# Single-Cell Transcriptomic and Regulatory Network Analyses Identify a Putative HNF4A–OXR1 Association Linked to Podocyte Mitochondrial Dysfunction in Diabetic Kidney Disease

**DOI:** 10.3390/ijms27219608

**Published:** 2026-10-27

**Authors:** Zhenkun Yang, Shimin Jiang, Wen Cui, Jiahui Zhou, Wenge Li

**Affiliations:** 1 Department of Nephrology, China-Japan Friendship Hospital, Beijing 100029, China; yzk1584772@163.com; 2 China-Japan Friendship Hospital (Institute of Clinical Medical Sciences), Chinese Academy of Medical Sciences & Peking Union Medical College, Beijing 100730, China; b2024023032@student.pumc.edu.cn; 3 Health Science Center, Peking University, Beijing 100191, China; 2211210680@pku.edu.cn

**Keywords:** diabetic kidney disease, podocyte, single-cell RNA sequencing, mitochondrial dysfunction, OXR1, HNF4A, network medicine

## Abstract

Podocyte injury is a central pathological event in diabetic kidney disease (DKD), but the mitochondrial regulatory mechanisms underlying podocyte dysfunction remain incompletely defined. We integrated single-nucleus RNA-seq data from human renal biopsies in GSE195460 with podocyte-specific differential expression, functional enrichment, mitochondrial gene annotation, co-expression network analysis, machine learning feature selection, pseudotime analysis, in silico knockout, transcription factor enrichment, motif analysis, and molecular docking; findings were externally evaluated in GSE209781. Podocytes showed prominent disease-associated transcriptomic perturbation, with 677 exploratory cell-level differentially expressed genes and enrichment of cytoskeletal, adhesion, developmental, oxidative phosphorylation, and metabolic pathways. Thirty-one mitochondrial-related differentially expressed genes were identified, and OXR1 was prioritized as a candidate regulatory hub based on consistent downregulation, network centrality, machine learning selection, and external validation. HNF4A was predicted as an upstream regulator linked to OXR1 and other mitochondrial targets. In silico OXR1 perturbation increased the predicted injured-state probability from 15% to 82%, while promoter motif analysis provided additional computational support for a putative HNF4A–OXR1 regulatory relationship. These findings highlight OXR1 as a candidate associated with podocyte mitochondrial dysfunction in DKD and suggest a putative HNF4A–OXR1 regulatory relationship.

## 1. Introduction

Diabetic kidney disease (DKD) is a major microvascular complication of diabetes and a leading cause of end-stage kidney disease worldwide [1]. Podocyte injury and loss are key pathological features of DKD and contribute directly to proteinuria and progressive renal dysfunction [2,3]. Although current therapies, including renin–angiotensin–aldosterone system blockade and sodium–glucose cotransporter 2 inhibitors, have improved renal outcomes, many patients remain at risk of disease progression [4]. Identifying podocyte-specific molecular mechanisms may therefore provide new opportunities for therapeutic intervention.

Mitochondrial dysfunction has emerged as an important contributor to podocyte injury in DKD. Because podocytes have high energetic demands, disturbances in oxidative phosphorylation, fatty acid oxidation, redox balance, and mitochondrial homeostasis may impair their structural and functional integrity [5,6]. However, the upstream regulatory mechanisms driving these mitochondrial alterations remain poorly understood. Moreover, bulk transcriptomic studies may obscure cell-type-specific signals within the heterogeneous kidney microenvironment, limiting the identification of podocyte-intrinsic regulatory networks.

Single-cell RNA sequencing enables high-resolution characterization of disease-associated transcriptional changes across specific renal cell populations [3,7]. Combined with mitochondrial gene annotation, machine learning approaches, and network analysis, single-cell data may help distinguish disease-associated markers from candidate regulatory nodes [8]. Nevertheless, the mitochondrial regulatory landscape of DKD podocytes and its potentially modifiable upstream pathways have not been fully defined.

In this study, we aimed to characterize podocyte-specific mitochondrial transcriptional alterations in diabetic kidney disease and identify potential regulatory genes and networks. We integrated human renal single-nucleus transcriptomic data with mitochondrial gene annotation, machine learning and network analyses, and independent dataset evaluation to provide a basis for future mechanistic investigations.

## 2. Results

### 2.1. Single-Cell Transcriptomic Profiling and Quality-Control Sensitivity Analysis

**Figure 1:**
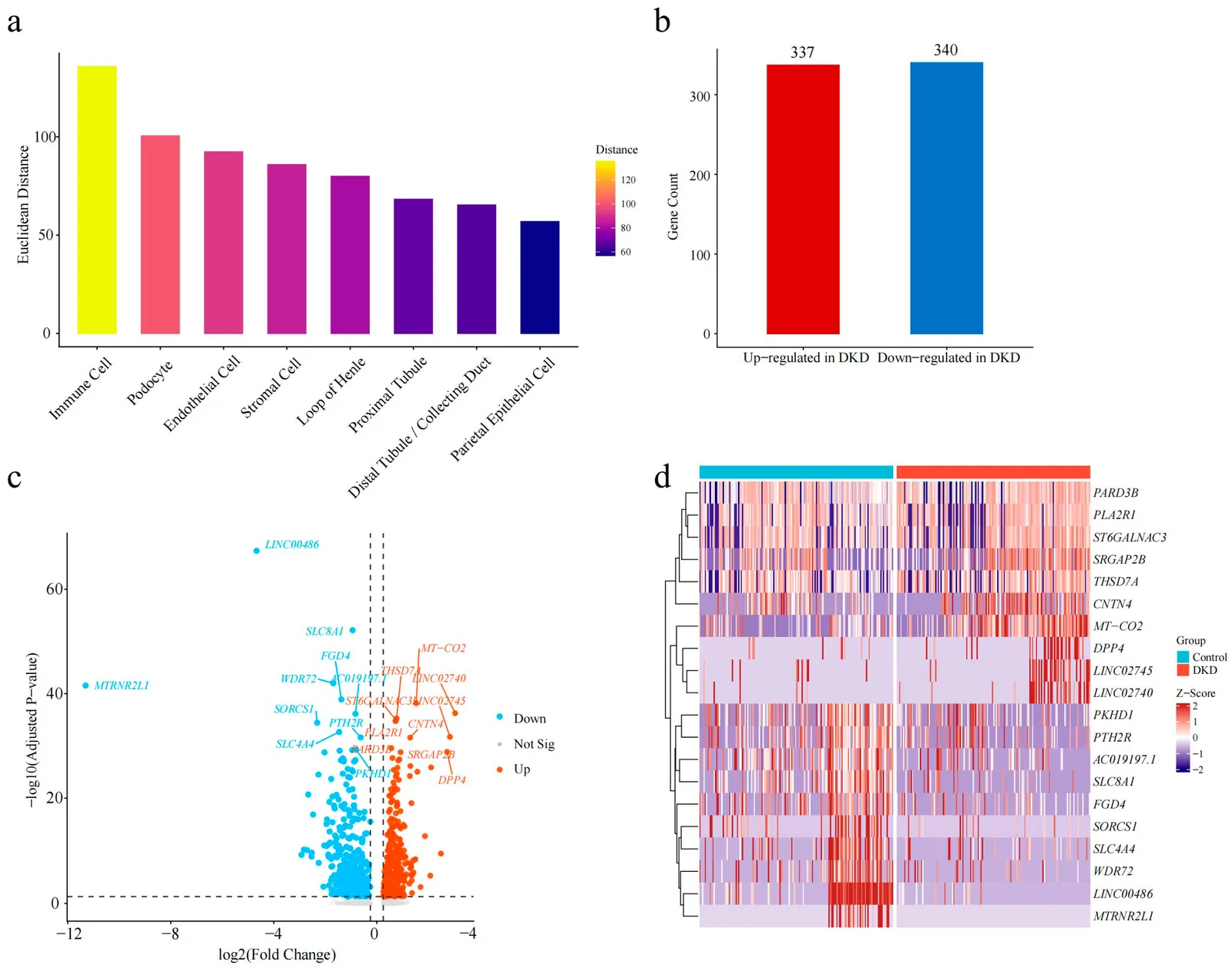
Transcriptomic differences across renal cell populations and differential gene expression in DKD podocytes. (**a**) Bar plot showing Euclidean distances between control and DKD transcriptomic profiles across eight major renal cell populations. (**b**) Numbers of differentially expressed genes (DEGs) in DKD podocytes, including 337 upregulated and 340 downregulated genes. (**c**) Volcano plot showing the distribution of DEGs between DKD and control podocytes. (**d**) Heatmap showing the expression patterns of representative DEGs in control and DKD podocytes. Columns are annotated by group, and colors indicate standardized expression levels (Z-scores). Abbreviations: DKD, diabetic kidney disease; DEG, differentially expressed gene; log2FC, log2 fold change.

Integrated analysis of GSE195460 identified eight major renal cell populations, including proximal tubular cells, distal tubular/collecting duct cells, loop of Henle cells, parietal epithelial cells, endothelial cells, immune cells, stromal cells, and podocytes (Supplementary Figure S1a). Cell-type distributions differed between control and DKD samples (Supplementary Figure S1b). Cell identities were supported by canonical lineage markers and cluster-enriched genes (Supplementary Figure S1c,d; Table S1). Transcriptomic distance analysis revealed substantial DKD-associated differences across renal cell populations, with podocytes among the prominently altered lineages (Figure 1a).

Quality-control sensitivity analysis showed that 61,792 (97.60%) and 62,631 (98.93%) of 63,309 nuclei were retained at mitochondrial transcript thresholds of 5% and 10%, respectively. Relaxing the threshold recovered 839 additional nuclei, including 86 control and 753 DKD nuclei. At the 5% threshold, mean OXR1 expression was 1.14 in control and 0.69 in DKD podocytes (log2FC = −0.724; *p* = 0.021). The corresponding values at the 10% threshold were 1.13 and 0.70, respectively (log2FC = −0.691; *p* = 0.028; Table S2).

### 2.2. Differential Expression and Functional Enrichment in DKD Podocytes

Cell-level differential expression analysis identified 677 DKD-associated genes in podocytes, including 337 upregulated and 340 downregulated genes (Figure 1b–d; Table S3). Patient-level pseudobulk analysis of six control and five DKD samples identified 18 significant genes (BH-FDR < 0.05 and |log2FC| > 0.25), comprising six upregulated and 12 downregulated genes (Supplementary Figure S2a; Table S4). OXR1 showed significant downregulation at the patient level (log2FC = −0.735; *p* = 1.21 × 10^−5^; BH-FDR = 0.0086; Supplementary Figure S2b).

KEGG enrichment analysis identified IgSF CAM signaling (34 genes; fold enrichment = 3.19; adjusted *p* = 4.97 × 10^−7^) and cytoskeleton-related pathways, including cytoskeleton in muscle cells (28 genes; fold enrichment = 3.37; adjusted *p* = 2.21 × 10^−6^) (Supplementary Figure S3a; Table S5). GO enrichment included regulation of actin filament-based processes (38 genes; fold enrichment = 3.22; adjusted *p* = 2.84 × 10^−7^) and cell–substrate adhesion (34 genes; fold enrichment = 3.15; adjusted *p* = 3.52 × 10^−6^) (Supplementary Figure S3b; Table S6). GSEA showed negative enrichment of the oxidative phosphorylation pathway (NES = −2.17; adjusted *p* = 1.53 × 10^−4^) and the broader metabolic-pathway gene set (NES = −1.97; adjusted *p* = 2.85 × 10^−8^) in DKD podocytes (Supplementary Figure S3c,d; Table S7).

### 2.3. Mitochondrial Gene Expression and Functional Module Scores in DKD Podocytes

**Figure 2:**
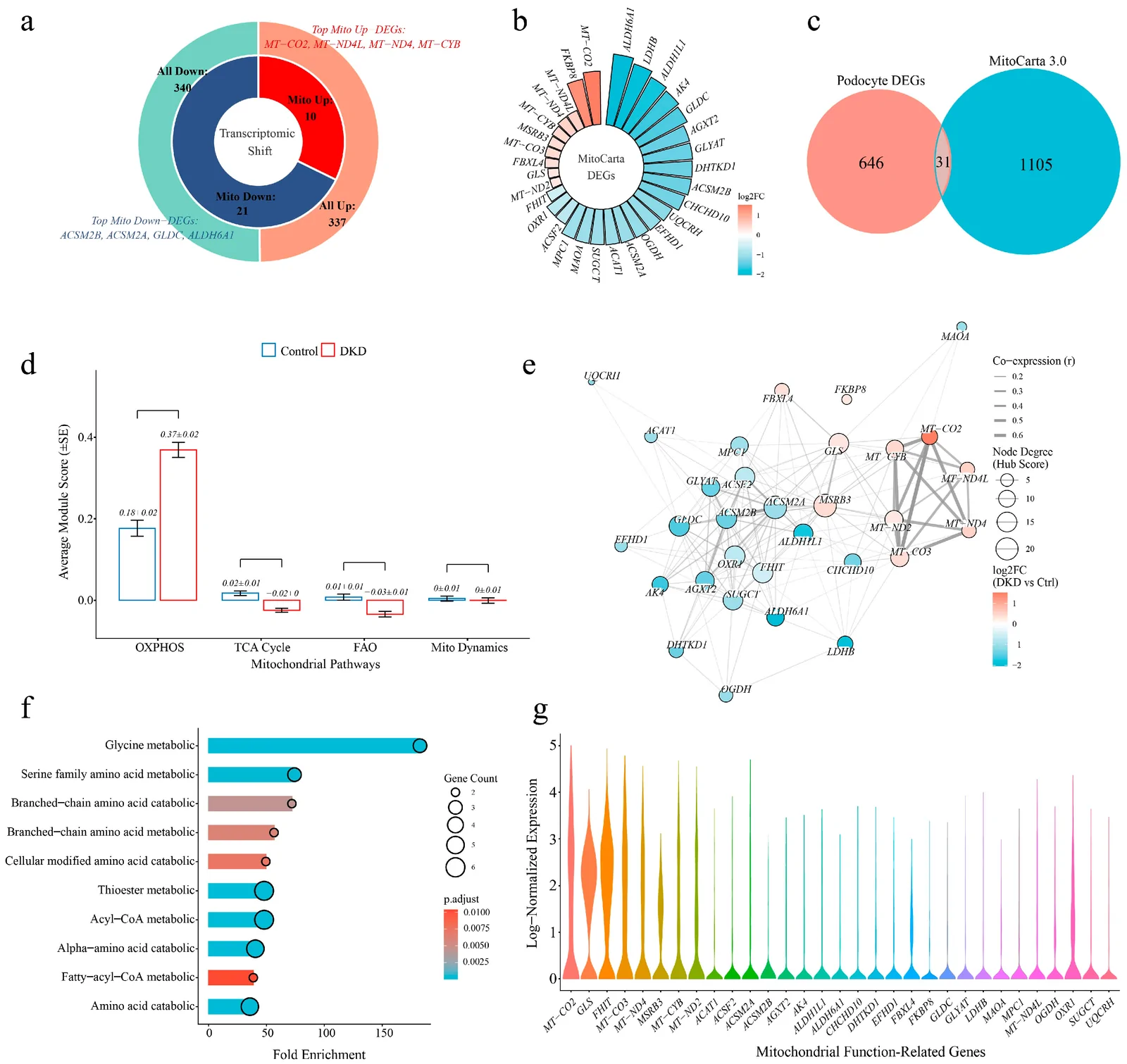
Mitochondrial DEG profiling defines a metabolic dysfunction signature in DKD podocytes. (**a**) Nested donut plot showing the distribution of podocyte DEGs and mitochondrial-related DEGs. (**b**) Circular heatmap showing log2FC values of mitochondrial-related DEGs. (**c**) Venn diagram showing the intersection between podocyte DEGs and the MitoCarta 3.0 mitochondrial gene inventory, identifying 31 mitochondrial DEGs. (**d**) Module-score analysis of mitochondrial functional programs, including OXPHOS, TCA cycle, fatty acid oxidation, and mitochondrial dynamics. (**e**) Co-expression network of mitochondrial DEGs. Node color indicates log2FC, node size indicates degree, and edge width represents co-expression strength. (**f**) GO enrichment analysis of mitochondrial DEGs. (**g**) Violin plots showing expression distributions of mitochondrial function-related genes in podocytes. Abbreviations: DKD, diabetic kidney disease; DEG, differentially expressed gene; Mito-DEG, mitochondrial-related differentially expressed gene; MitoCarta 3.0, mitochondrial protein inventory version 3.0; log2FC, log2 fold change; OXPHOS, oxidative phosphorylation; TCA cycle, tricarboxylic acid cycle; FAO, fatty acid oxidation; GO, Gene Ontology.

Intersection of the 677 podocyte DEGs with the MitoCarta 3.0 inventory identified 31 mitochondrial-related DEGs, comprising 10 upregulated and 21 downregulated genes (Figure 2a–c; Table S8). Among the upregulated genes, MT-CO2 showed an average log2FC of 1.529, followed by MT-ND4L (0.545) and MT-ND4 (0.534). Downregulated genes included ALDH6A1 (−2.037), GLDC (−1.568), ACSM2B (−1.393), ACSM2A (−1.041), and OXR1 (−0.717) (Figure 2b; Table S8). Their co-expression relationships and expression distributions are presented in Figure 2e,g.

Mitochondrial module-score analysis showed a higher mean OXPHOS score in DKD than in control podocytes (0.3689 ± 0.0188 vs. 0.1769 ± 0.0194; *p* = 8.88 × 10^−17^). In contrast, TCA-cycle scores were lower in DKD (−0.0247 vs. 0.0173; *p* = 6.92 × 10^−5^), as were FAO scores (−0.0341 vs. 0.0078; *p* = 1.28 × 10^−3^). Mitochondrial-dynamics scores did not differ significantly between groups (*p* = 0.869; Figure 2d; Table S9). GO analysis of mitochondrial-related DEGs identified enrichment of acyl-CoA metabolic processes (six genes; fold enrichment = 47.94; adjusted *p* = 4.04 × 10^−7^; Figure 2f; Table S10).

### 2.4. Machine Learning and Network Analysis of Mitochondrial-Related DEGs

**Figure 3:**
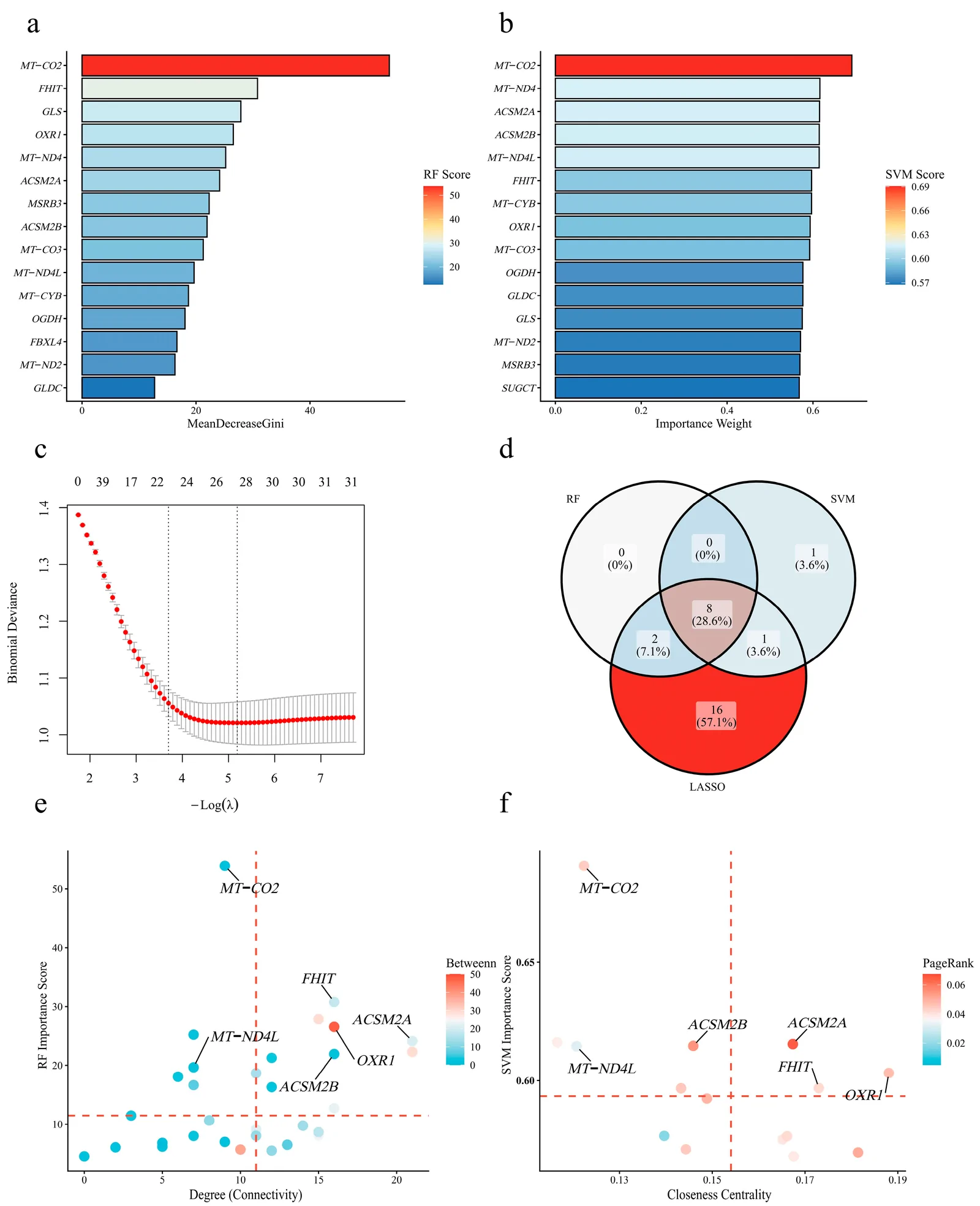
Machine learning and network topology prioritize OXR1 as a candidate mitochondrial hub in DKD podocytes. (**a**) Random Forest ranking of mitochondrial DEGs based on Mean Decrease Gini scores. (**b**) SVM-based feature importance ranking of mitochondrial DEGs. (**c**) LASSO regression with cross-validation for feature selection. (**d**) Venn diagram showing the intersection of the top 10 Random Forest-ranked genes, the top 10 SVM-RFE-ranked genes, and genes with nonzero LASSO coefficients at lambda.1se, identifying eight overlapping candidates. (**e**) Integration of Random Forest importance with degree and betweenness centrality. (**f**) Integration of SVM importance with closeness centrality and PageRank. Candidate hub genes, including OXR1, ACSM2A, ACSM2B, FHIT, and MT-CO2, are annotated. Abbreviations: DKD, diabetic kidney disease; RF, Random Forest; SVM, support vector machine; LASSO, least absolute shrinkage and selection operator; DEG, differentially expressed gene; Mean Decrease Gini, Random Forest-based feature importance score.

The intersection of Random Forest, SVM-RFE, and LASSO identified eight consensus candidate genes: ACSM2A, ACSM2B, FHIT, OXR1, MT-CO2, MT-CO3, MT-ND4, and MT-ND4L (Figure 3a–d; Tables S11 and S12). MT-CO2 ranked first in both Random Forest and SVM-RFE, with importance scores of 53.9201 and 0.690638, respectively. OXR1 ranked fourth in Random Forest (26.5416) and eighth in SVM-RFE (0.593450) and was also retained by LASSO. Network analysis showed that OXR1 had a degree of 16, betweenness centrality of 50, closeness centrality of 0.1881, and PageRank of 0.0361. Among the 31 mitochondrial-related DEGs, OXR1 had the highest betweenness and closeness centrality (Figure 3e,f; Tables S11 and S12).

### 2.5. OXR1 Expression in an Independent Single-Cell Cohort

The independent GSE209781 cohort comprised three DKD patients (one male and two females; aged 30–47 years) and three reference donors (two males and one female; aged 42–64 years). Clinical characteristics, including renal function, proteinuria, and pathological diagnoses, are summarized in Table S13. UMAP analysis showed the cellular distribution of the external dataset (Supplementary Figure S4a), and podocyte-enriched regions were identified by NPHS1, NPHS2, and SYNPO expression (Supplementary Figure S4b). In pooled-cell summaries, mean OXR1 expression was 0.504 in DKD and 0.713 in reference podocytes, representing approximately 29% lower expression in the DKD group (Supplementary Figure S4c; Table S14).

In GSE271188, donor-level OXR1 expression was lower in DKD podocytes, although the difference was not statistically significant when all donors were included (control, *n* = 7; DKD, *n* = 4; *p* = 0.0727). After excluding donors with fewer than 10 podocytes, mean OXR1 expression was 0.633 in DKD and 1.119 in controls (*p* = 0.0238; Supplementary Figure S5; Table S15).

### 2.6. Transcription Factor Enrichment and Predicted Regulatory Network Analysis

**Figure 4:**
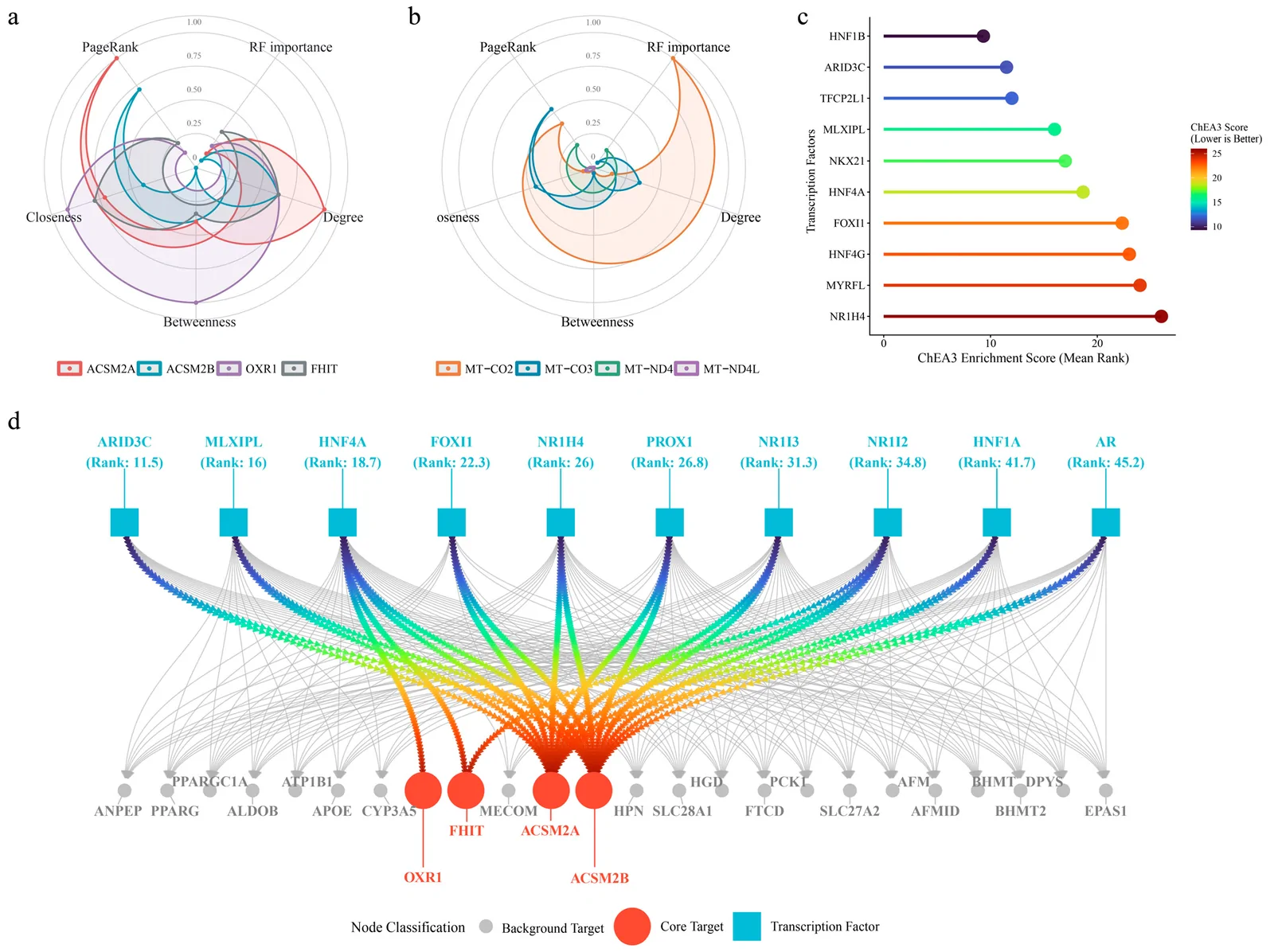
Machine learning and network-topological profiles of candidate mitochondrial-related genes and predicted transcriptional regulatory network in DKD podocytes. (**a**) Radar plot showing the normalized Random Forest feature importance and network-topological characteristics of four nuclear-encoded candidate genes (ACSM2A, ACSM2B, OXR1, and FHIT). (**b**) Radar plot showing the corresponding profiles of four mitochondrially encoded candidate genes (MT-CO2, MT-CO3, MT-ND4, and MT-ND4L). Five metrics were evaluated: Random Forest importance (MeanDecreaseGini), degree, betweenness, closeness, and PageRank. Each metric was independently min–max normalized to a 0–1 scale across all eight candidate genes. (**c**) ChEA3-based transcription factor enrichment analysis identifying putative upstream regulators associated with downregulated podocyte genes. (**d**) Predicted transcription factor–target network connecting candidate transcription factors to mitochondrial-related genes. OXR1, FHIT, ACSM2A, and ACSM2B are highlighted as candidate targets. Abbreviations: DKD, diabetic kidney disease; RF, Random Forest; TF, transcription factor; ChEA3, ChIP-X Enrichment Analysis version 3; HNF4A, hepatocyte nuclear factor 4 alpha; OXR1, oxidation resistance 1.

Radar plots showed differences in normalized Random Forest importance and network-topological characteristics among the eight candidate genes (Figure 4a,b). OXR1 exhibited the highest betweenness and closeness centrality among the nuclear-encoded candidates. ChEA3 analysis ranked HNF1B first (integrated mean-rank score = 9.33), whereas HNF4A ranked sixth (score = 18.67; Figure 4c; Table S16). The predicted transcription factor–target network included associations between HNF4A and OXR1, ACSM2A, ACSM2B, FHIT, and additional podocyte DEGs (Figure 4d; Table S17).

### 2.7. Podocyte Trajectories and Predicted Injury-Associated Effects of OXR1 Perturbation

**Figure 5:**
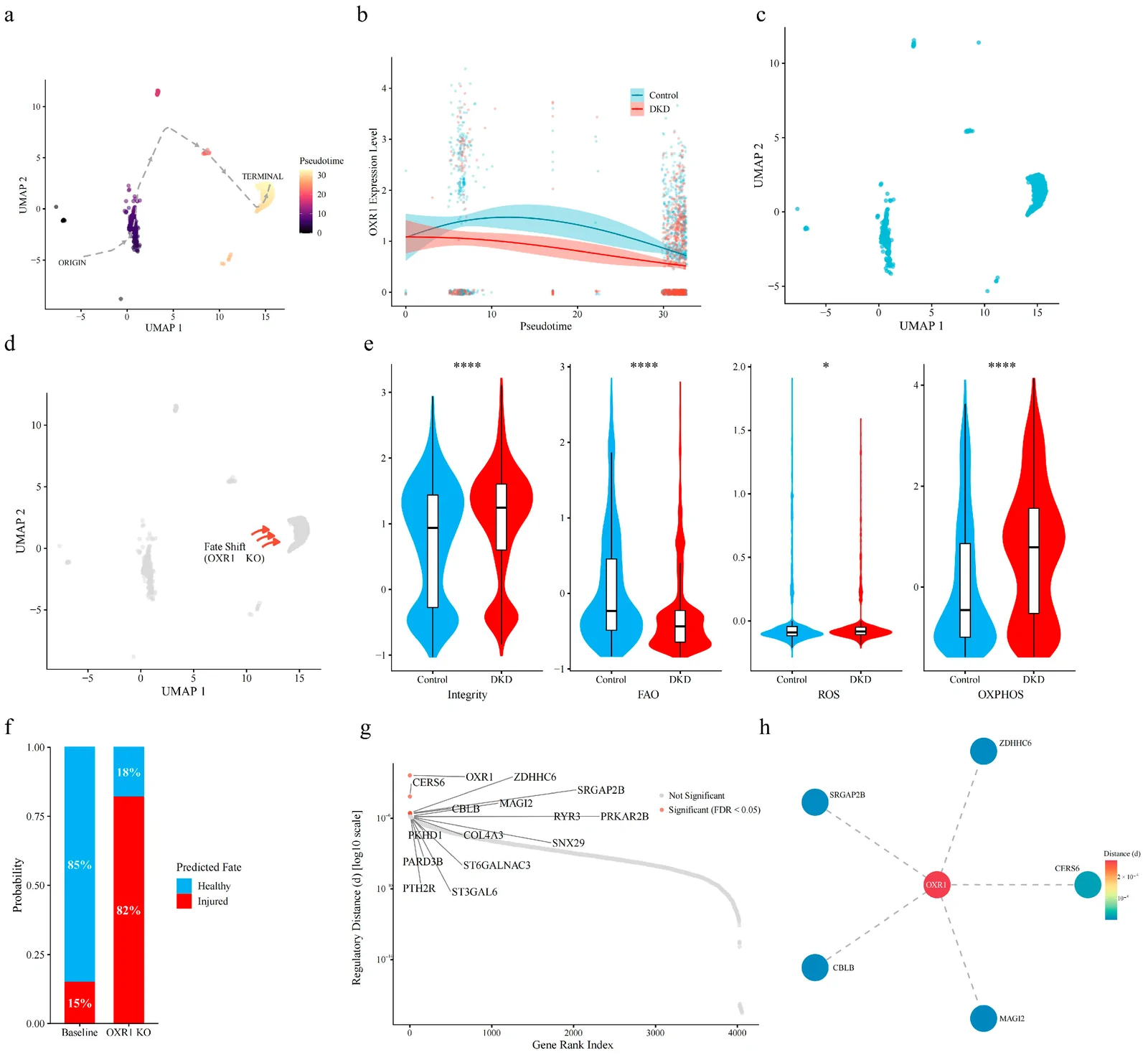
In silico OXR1 perturbation predicts transition toward an injured podocyte state. (**a**) Pseudotime trajectory analysis showing podocyte state progression from origin to terminal states. (**b**) Dynamic expression pattern of OXR1 along pseudotime in control and DKD podocytes. (**c**) Baseline UMAP of podocytes before perturbation. (**d**) Simulated fate-shift vector field after in silico OXR1 knockout. (**e**) Comparison of podocyte functional module scores between control and DKD groups, including integrity, FAO, ROS, and OXPHOS signatures. (**f**) Fate probability analysis showing an increase in predicted injured-state probability from 15% at baseline to 82% after OXR1 knockout. (**g**) Regulatory-distance analysis identifying genes most affected by OXR1 perturbation. (**h**) Network visualization of the major downstream genes affected by OXR1 perturbation, including CERS6, ZDHHC6, SRGAP2B, CBLB, and MAGI2. Statistical significance is indicated as follows: * *p* < 0.05 and **** *p* < 0.0001. Abbreviations: DKD, diabetic kidney disease; UMAP, Uniform Manifold Approximation and Projection; KO, knockout; OXR1, oxidation resistance 1; FAO, fatty acid oxidation; ROS, reactive oxygen species; OXPHOS, oxidative phosphorylation; FDR, false discovery rate.

Slingshot analysis included 1574 podocyte nuclei distributed across five subclusters (C0–C4). C0 contained 453 nuclei and exhibited the highest composite podocyte-marker score (1.0935). Two transcriptional lineages were inferred: Lineage 1 (C0–C1–C2–C3; 1409 nuclei assigned pseudotime) and Lineage 2 (C0–C4; 579 nuclei assigned pseudotime) (Figure 5a; Table S18).

OXR1 expression showed weak negative correlations with inferred pseudotime in Lineage 1 (Spearman’s ρ = −0.0829) and Lineage 2 (ρ = −0.0658). Mean OXR1 expression was lower in DKD than in control podocytes in both lineages (Lineage 1: 0.6505 vs. 0.8689; Lineage 2: 0.6554 vs. 0.9125; Figure 5b; Table S19). The two-lineage structure was retained in all 11 leave-one-library-out analyses, whereas using C4 as the alternative root produced a single lineage (C4–C0–C1–C2–C3).

Separate comparisons of control and DKD podocytes showed differences in the distributions of integrity, FAO, ROS, and OXPHOS-related scores (Figure 5e). In the computational OXR1 perturbation outputs, the modeled fate-shift visualization indicated a shift toward the injured-state region (Figure 5c,d). The predicted injured-state probability increased from 15% at baseline to 82% following perturbation (Figure 5f). Regulatory-distance analysis identified six genes with FDR < 0.05: OXR1, CERS6, ZDHHC6, SRGAP2B, CBLB, and MAGI2 (Figure 5g,h; Table S20). OXR1 showed the largest regulatory distance (d = 2.77 × 10^−4^; Z = 7.33), followed by CERS6 (d = 1.75 × 10^−5^; Z = 4.47).

### 2.8. Predicted HNF4A Motif and PGE1–EP2 Interactions

Motif analysis identified a putative HNF4A-binding sequence within the upstream regulatory region of OXR1. The predicted sequence CAAAGTCA matched the HNF4A matrix MA0114.5, with a motif score of 12.11742 and a relative score of 0.9098152 (Supplementary Figure S6a). Molecular docking positioned PGE1 within a predicted binding pocket of the human EP2 receptor, with modeled ligand–receptor contacts of approximately 2.8–3.2 Å (Supplementary Figure S6b).

## 3. Discussion

In this study, we integrated single-cell transcriptomics with mitochondrial gene annotation, machine learning feature selection, network analysis, transcription factor prediction, in silico perturbation, and molecular docking to investigate podocyte-specific mitochondrial dysregulation in DKD. Our analyses identified podocytes as a prominently altered renal cell population and revealed coordinated changes in cytoskeletal, adhesion, developmental, and metabolic pathways. Among mitochondrial-related genes, OXR1 emerged as a consistently prioritized candidate based on differential expression, network centrality, machine learning selection, and external validation. Upstream regulatory analysis further implicated HNF4A as a potential regulator of OXR1. Additional docking analysis was performed as an exploratory, literature-informed assessment of PGE1–EP2 structural compatibility. Together, these findings provide a network-based framework for understanding mitochondrial dysfunction in DKD podocytes.

Podocyte injury is a major contributor to proteinuria and progressive renal dysfunction in DKD [9,10]. Mitochondrial dysfunction has also been increasingly recognized as an important component of podocyte injury under diabetic stress [5,6]. Consistent with these observations, our single-cell analysis revealed broad transcriptional remodeling in DKD podocytes, involving oxidative phosphorylation, fatty acid oxidation, cytoskeletal organization, and cell adhesion. Recent multi-omics studies have similarly highlighted metabolic reprogramming in DKD [11], while mitochondrial quality control is known to be important for maintaining renal cellular homeostasis [12]. Because podocytes have high energetic demands and limited regenerative capacity, disruption of mitochondrial metabolism may reduce their ability to adapt to persistent metabolic stress. Experimental studies showing that modulation of metabolic pathways can influence diabetic nephropathy further support the relevance of mitochondrial and metabolic regulation in disease progression [13].

A central finding of our study was the prioritization of OXR1 as a candidate mitochondrial regulatory hub. OXR1 was not selected solely on the basis of differential expression but was supported by several independent analytical layers, including mitochondrial gene screening, consensus machine learning feature selection, network centrality analysis, and external single-cell evaluation. Patient-level pseudobulk analysis further supported OXR1 downregulation in DKD podocytes. This concordance strengthened the evidence supporting OXR1 prioritization. Nevertheless, the limited number of biological replicates and the computational nature of the study warrant cautious interpretation. Previous studies in other biological systems have linked OXR1 to oxidative stress resistance, mitochondrial protection, and cellular responses to metabolic stress [14,15]. Although these studies were not performed in DKD podocytes, they provide biological support for a potential role of OXR1 in maintaining cellular stress tolerance. The reduced expression of OXR1 observed in DKD podocytes may therefore reflect impaired stress-defense capacity, although this possibility requires direct experimental testing.

Pseudotime analysis identified two putative transcriptional lineages among podocyte subclusters, with OXR1 exhibiting weak negative associations with inferred pseudotime along both lineages. The inferred two-lineage structure was preserved across all 11 leave-one-library-out analyses but changed when an alternative reference root was selected, indicating sensitivity to root selection. Importantly, these trajectories reflect inferred transcriptional-state relationships rather than chronological DKD progression. Separately, in silico OXR1 perturbation using scTenifoldKnk identified regulatory displacement among several genes, suggesting potential network-level consequences of OXR1 loss. These findings are consistent with the concept that perturbation of highly connected network components may affect broader transcriptional programs [16]. However, the computational analyses remain hypothesis-generating and do not establish a causal role for OXR1 in podocyte injury. Experimental loss- and gain-of-function studies are required to determine whether OXR1 directly regulates mitochondrial homeostasis and podocyte survival under diabetic conditions.

Our analyses also identified HNF4A as a candidate upstream regulator connected to OXR1 and other mitochondrial-related genes. HNF4A is an established regulator of metabolic transcriptional programs, and previous studies have linked HNF4A-dependent signaling to cellular metabolism and interactions with metabolic co-regulators such as PGC-1α [17,18]. In our study, transcription factor enrichment and regulatory network analysis connected HNF4A with OXR1, FHIT, ACSM2A, and ACSM2B, while promoter analysis identified a putative HNF4A-binding motif upstream of OXR1. These convergent computational observations support a possible HNF4A–OXR1 regulatory relationship. Nevertheless, the current evidence does not establish direct transcriptional regulation. Chromatin immunoprecipitation, promoter-reporter assays, and HNF4A perturbation experiments will be required to determine whether HNF4A directly regulates OXR1 in podocytes.

Previous pharmacological studies have shown that PGE1 can interact with the human EP2 receptor and that EP2 is coupled to cAMP-generating signaling, providing a receptor-level rationale for examining PGE1–EP2 structural compatibility [19,20]. Structural studies have further characterized agonist recognition and Gs coupling of human EP2 [21]. However, prostanoid signaling in podocytes appears to be receptor- and context-dependent, with previous studies predominantly implicating EP4 and also demonstrating PKA-independent signaling [22,23]. Moreover, cAMP/PKA regulation of HNF4A is context-dependent [24]. Therefore, the PGE1–EP2 docking result should be interpreted as hypothesis-generating evidence of structural compatibility rather than evidence for a continuous PGE1–EP2–PKA–HNF4A–OXR1 pathway. Direct experimental assessment is required to determine whether these components are functionally connected in podocytes.

More broadly, previous models of DKD have emphasized oxidative stress and mitochondrial dysfunction as consequences of chronic metabolic injury [5,6,25]. Our findings suggest a complementary possibility in which disruption of upstream regulatory networks may contribute to the loss of mitochondrial homeostasis in podocytes. Rather than considering mitochondrial-related genes only as downstream biomarkers, the present analysis places OXR1 within an interconnected network involving oxidative stress defense, mitochondrial metabolism, and transcriptional regulation. Current DKD therapies have substantially improved renal outcomes but do not specifically target podocyte-intrinsic mitochondrial regulatory failure [26]. The predicted HNF4A–OXR1 regulatory relationship provides a hypothesis for future mechanistic studies rather than evidence of an established regulatory mechanism.

Several limitations should be considered. First, the study is primarily based on computational analyses of publicly available single-cell datasets, and the predicted HNF4A–OXR1 regulatory relationship requires experimental validation. Second, single-cell RNA-sequencing measures transcript abundance but does not directly assess protein expression, mitochondrial respiration, enzyme activity, or post-translational regulation. Third, the in silico OXR1 knockout predicts network-level consequences and cannot substitute for experimental loss- or gain-of-function studies. Fourth, the PGE1–EP2 docking analysis was literature-informed, which supports the structural feasibility of PGE1–EP2 interaction but does not demonstrate pathway activation or therapeutic efficacy. An additional limitation concerns the use of nephrectomy-derived reference kidney tissues. Although these tissues were selected as non-tumorous comparators, potential tumor-associated and tissue procurement-related confounding cannot be excluded. Further validation using well-characterized reference kidney tissues is therefore warranted. Furthermore, the small external cohort (three biological replicates per group) and unequal cellular contributions among donors limit statistical power and the robustness of the external comparison. Mitochondrial filtering also excluded a greater proportion of nuclei from certain DKD samples, potentially reducing the representation of disease-associated stressed cell populations. Finally, inter-individual heterogeneity, differences in disease stage, and dataset-specific technical effects may influence the generalizability of the findings. Future studies should therefore validate OXR1 and HNF4A in human kidney tissue and experimental DKD models, directly test HNF4A–OXR1 transcriptional regulation, and determine whether EP2 signaling and PKA activity are functionally linked to HNF4A/OXR1 regulation and mitochondrial responses in podocytes.

## 4. Materials and Methods

### 4.1. Single-Cell RNA Sequencing Data Processing and Integration

Publicly available human renal single-cell and single-nucleus RNA-sequencing datasets were obtained from the Gene Expression Omnibus (GEO) database. GSE195460 was used as the discovery cohort, whereas GSE209781 and GSE271188 were used for independent external evaluation. According to the original study by Wilson et al., renal cortical tissues in GSE195460 were obtained from patients undergoing nephrectomy for renal masses or from deceased organ donors. Histopathological assessment was performed in the original study, and reference kidney samples exhibited less than 10% global glomerulosclerosis and interstitial fibrosis/tubular atrophy. For GSE209781, DKD samples were obtained by needle biopsy, whereas reference samples were collected from non-DKD kidney tissues within tumor nephrectomy specimens.

Raw gene–barcode count matrices from GSE195460 were processed in R (v4.4.3) using Seurat (v4.2) [7]. Nuclei with more than 200 and fewer than 4000 detected genes and a mitochondrial transcript percentage below 5% were retained [27]. To evaluate the influence of mitochondrial transcript filtering, the original 5% cutoff was compared with a relaxed 10% cutoff while maintaining identical gene-count criteria. Nucleus retention was summarized separately for each sample. Normalization and variance stabilization were performed using SCTransform with the glmGamPoi method (vst.flavor = “v2”), with mitochondrial transcript percentage included as a regression covariate [28]. Batch effects were corrected using Harmony, with the original sample identifier (orig.ident) specified as the batch variable. The first 30 Harmony-corrected dimensions were used for Uniform Manifold Approximation and Projection (UMAP), nearest-neighbor graph construction, and graph-based clustering at a resolution of 0.5.

### 4.2. Cell-Type Annotation and Podocyte Differential Expression Analysis

Following quality control, normalization, and batch correction, cluster-specific marker genes were identified using the Seurat FindAllMarkers function with the Wilcoxon rank-sum test (only.pos = TRUE, min.pct = 0.25, and logfc.threshold = 0.25). Major renal cell populations were annotated according to established lineage-specific markers. The podocyte population was identified based on the expression of canonical podocyte markers, including NPHS1, NPHS2, WT1, PTPRO, and PODXL, and subsequently extracted using the Seurat subset function.

To characterize disease-associated transcriptional differences across renal cell populations, average normalized gene-expression profiles were calculated for each annotated cell type within individual samples. For each cell type, donor-level expression profiles were averaged within the DKD and control groups using a common set of detected genes. Euclidean distances were then calculated between the resulting group-level expression vectors.

For exploratory differential expression analysis, RNA counts were normalized using the Seurat LogNormalize method with a scale factor of 10,000. Differential expression between DKD and control podocytes was assessed using Seurat FindMarkers with the Wilcoxon rank-sum test (assay = “RNA”, slot = “data”, and min.pct = 0.1). Genes with a Bonferroni-adjusted *p* value < 0.05 and an absolute log2 fold change > 0.25 were considered exploratory differentially expressed genes (DEGs).

To account for the non-independence of nuclei originating from the same donor, patient-level pseudobulk differential expression analysis was additionally performed using the GSE195460 discovery cohort. Raw podocyte counts were aggregated within each donor to generate one pseudobulk expression profile per biological replicate, comprising six control and five DKD donors. Differential expression was assessed using DESeq2 with disease status as the design factor (design = ~ condition), with the control group as the reference. *p* values were adjusted using the Benjamini–Hochberg method, and genes with an adjusted *p* value < 0.05 and an absolute log2 fold change > 0.25 were considered significant.

### 4.3. Functional Enrichment and Mitochondrial Module Scoring

Functional enrichment of podocyte DEGs was performed using the clusterProfiler R package [29]. Gene Ontology (GO) and Kyoto Encyclopedia of Genes and Genomes (KEGG) enrichment analyses were performed using a hypergeometric test, with *p* < 0.05 considered significant. Gene set enrichment analysis (GSEA) was performed using all detected genes ranked according to log2FC and KEGG gene sets.

Mitochondrial-related DEGs were identified by intersecting significant podocyte DEGs with the human MitoCarta 3.0 mitochondrial gene inventory [30]. Four predefined mitochondrial functional programs—oxidative phosphorylation (OXPHOS), fatty acid oxidation (FAO), the tricarboxylic acid (TCA) cycle, and mitochondrial dynamics—were quantified at the single-cell level using the Seurat AddModuleScore function. For each program, predefined genes were intersected with genes detected in the podocyte expression matrix before module-score calculation. The gene sets used for module scoring are provided in Supplementary Table S21. Module scores were compared between DKD and control podocytes using the Wilcoxon rank-sum test.

### 4.4. Mitochondrial Co-Expression Network and Machine Learning Analysis

Pairwise Pearson correlations among mitochondrial-related DEGs were calculated using the rcorr function. Gene pairs with *p* < 0.05 and |r| > 0.1 were retained to construct a co-expression network. Network topology was analyzed using igraph, and degree, betweenness centrality, closeness centrality, and PageRank were calculated. Networks were visualized using ggraph.

Three machine learning methods were applied to the expression matrix of mitochondrial-related DEGs. Equal numbers of DKD and control nuclei were randomly sampled, with a maximum of 500 nuclei per group and a fixed random seed of 2026. Random Forest (RF) was performed using 1000 trees, with gene importance evaluated using MeanDecreaseGini [31]. Support vector machine–recursive feature elimination (SVM-RFE) was implemented using the caret package with a linear support vector machine and five-fold cross-validation. Least absolute shrinkage and selection operator (LASSO) logistic regression was performed using glmnet (family = “binomial”, alpha = 1) with ten-fold cross-validation. Genes with nonzero coefficients at lambda.1se were retained [32].

Consensus candidates were defined by the intersection of the top 10 RF-ranked genes, the top 10 SVM-RFE-ranked genes, and genes retained by LASSO. Their network-topological characteristics were subsequently evaluated. For radar-plot visualization, RF importance and four network metrics were independently min–max normalized across the consensus candidates using (x − xmin)/(xmax − xmin).

### 4.5. External Validation of Candidate Mitochondrial Genes

Candidate mitochondrial genes were evaluated using the independent single-cell dataset GSE209781, comprising three DKD patients and three reference donors. Podocytes were annotated using canonical markers, including NPHS1, NPHS2, and SYNPO. Candidate-gene expression was summarized across the annotated podocyte populations. Pooled-cell expression comparisons were considered descriptive and were not treated as independent donor-level statistical tests.

An additional independent dataset, GSE271188, comprising seven control and four DKD donors, was used for donor-level evaluation of OXR1 expression. Podocytes were extracted, and mean OXR1 expression was calculated for each donor. Between-group differences were assessed using two-sided exact Wilcoxon rank-sum tests. A sensitivity analysis was conducted after excluding donors with fewer than 10 podocytes.

### 4.6. Transcription Factor Enrichment and Regulatory Network Construction

Podocyte DEGs were submitted to ChEA3 for transcription factor enrichment analysis [33]. Transcription factors were ranked according to the ChEA3 integrated mean rank, which integrates multiple transcriptional regulatory resources. Predicted transcription factor–target relationships were subsequently intersected with mitochondrial-related candidate genes to construct a transcription factor–mitochondrial gene regulatory network and were visualized using ggraph. HNF4A was retained for further investigation because the integrated regulatory network connected it to OXR1 and several additional mitochondrial-associated candidates.

### 4.7. Slingshot-Based Trajectory Inference and scTenifoldKnk Perturbation Modeling

Podocyte transcriptional trajectories were inferred using Slingshot based on UMAP embeddings and five podocyte subclusters (C0–C4). The reference root was selected according to the mean standardized expression of five canonical podocyte markers (NPHS1, NPHS2, WT1, PTPRO, and PODXL). Cluster-level mean expression values were log1p-transformed, standardized across subclusters for each marker, and averaged to generate a marker score. The subcluster with the highest score (C0) was designated as the reference root. Slingshot was performed using subcluster identities as cluster labels and C0 as the starting cluster. The number and composition of inferred lineages were recorded, and lineage-specific pseudotime values were extracted. Trajectory sensitivity was assessed using C4 as an alternative root and through leave-one-library-out analyses. Associations between OXR1 expression and pseudotime were summarized using Spearman correlation coefficients.

The functional consequences of OXR1 perturbation were evaluated using the gene regulatory network-based scTenifoldKnk algorithm [34]. OXR1 was computationally suppressed, and the perturbed network was compared with the original network using nc_nNet = 3 and nc_nCells = 300. Regulatory distance was calculated to quantify transcriptomic displacement following OXR1 perturbation. Multiple testing was corrected using the Benjamini–Hochberg false discovery rate (FDR).

### 4.8. HNF4A Motif Screening and PGE1–EP2 Molecular Docking

The upstream regulatory region of OXR1 was screened for putative HNF4A-binding motifs using the JASPAR database and the human HNF4A position frequency matrix MA0114.5. Predicted binding sites were evaluated according to motif scores, relative scores, genomic positions, strand orientation, and nucleotide sequences.

Prostaglandin E1 (PGE1) was selected for an exploratory, literature-informed assessment of EP2 receptor binding. The three-dimensional structure of PGE1 (PubChem CID: 5280723) was obtained from PubChem, and the human EP2 receptor structure (PDB ID: 7CX2) was retrieved from the RCSB Protein Data Bank. The receptor was prepared by removing the co-crystallized ligand and non-receptor components. Blind molecular docking was performed using CB-Dock2 (http://cadd.labshare.cn/cb-dock2/, accessed on 17 September 2026) with AutoDock Vina 1.1.2 Vina-based scoring [35,36]. Predicted binding poses and ligand–receptor contacts were examined to assess structural compatibility.

### 4.9. Statistical Analysis and Visualization

Statistical analyses were performed in R (v4.4.3). The statistical unit was defined according to the analytical objective. Unless otherwise specified, other two-group comparisons were conducted using the Wilcoxon rank-sum test. Multiple testing was corrected using the Benjamini–Hochberg method, with FDR < 0.05 considered statistically significant.

## 5. Conclusions

OXR1 was identified as a candidate associated with mitochondrial dysfunction in DKD podocytes, with HNF4A predicted as a potential upstream regulator. Computational perturbation and promoter motif analyses provide hypothesis-generating support for the biological relevance of OXR1 and a putative HNF4A–OXR1 regulatory relationship. Further experimental studies are required to establish the functional roles and regulatory mechanisms of OXR1 in DKD podocytes.

## Data Availability

The datasets analyzed in this study are publicly available in the Gene Expression Omnibus (GEO) database under accession numbers GSE195460, GSE209781 and GSE271188. Data supporting the findings of this study are provided within the article and its Supplementary Materials. No new primary datasets were generated in this study.

## Supplementary Materials

The following supporting information can be downloaded at https://www.mdpi.com/article/10.3390/ijms27219608/s1.

## Abbreviations

The following abbreviations are used in this manuscript:

ChEA3	ChIP-X Enrichment Analysis 3
DEG	Differentially expressed gene
DKD	Diabetic kidney disease
EP2	Prostaglandin E receptor 2
FAO	Fatty acid oxidation
FDR	False discovery rate
GEO	Gene Expression Omnibus
GO	Gene Ontology
GSEA	Gene Set Enrichment Analysis
HNF4A	Hepatocyte nuclear factor 4 alpha
KEGG	Kyoto Encyclopedia of Genes and Genomes
LASSO	Least absolute shrinkage and selection operator
Mito-DEG	Mitochondrial-related differentially expressed gene
OXPHOS	Oxidative phosphorylation
OXR1	Oxidation resistance 1
PGE1	Prostaglandin E1
PKA	Protein kinase A
RAAS	Renin–angiotensin–aldosterone system
ROS	Reactive oxygen species
scRNA-seq	Single-cell RNA sequencing
SGLT2	Sodium–glucose cotransporter 2
SVM-RFE	Support vector machine–recursive feature elimination
TCA	Tricarboxylic acid
TF	Transcription factor
UMAP	Uniform Manifold Approximation and Projection
