# Supplementary material for: Single-Cell Transcriptomic and Regulatory Network Analyses Identify a Putative HNF4A–OXR1 Association Linked to Podocyte Mitochondrial Dysfunction in Diabetic Kidney Disease
Source: Int J Mol Sci. 2026 Oct 27;27(21):9608. doi: 10.3390/ijms27219608 (PMC13762632; doi:10.3390/ijms27219608)
Supplement: Supplementary file 1 [file ijms-27-09608-s001.zip › Supplementary_Figures.pdf]

Supplementary Figures

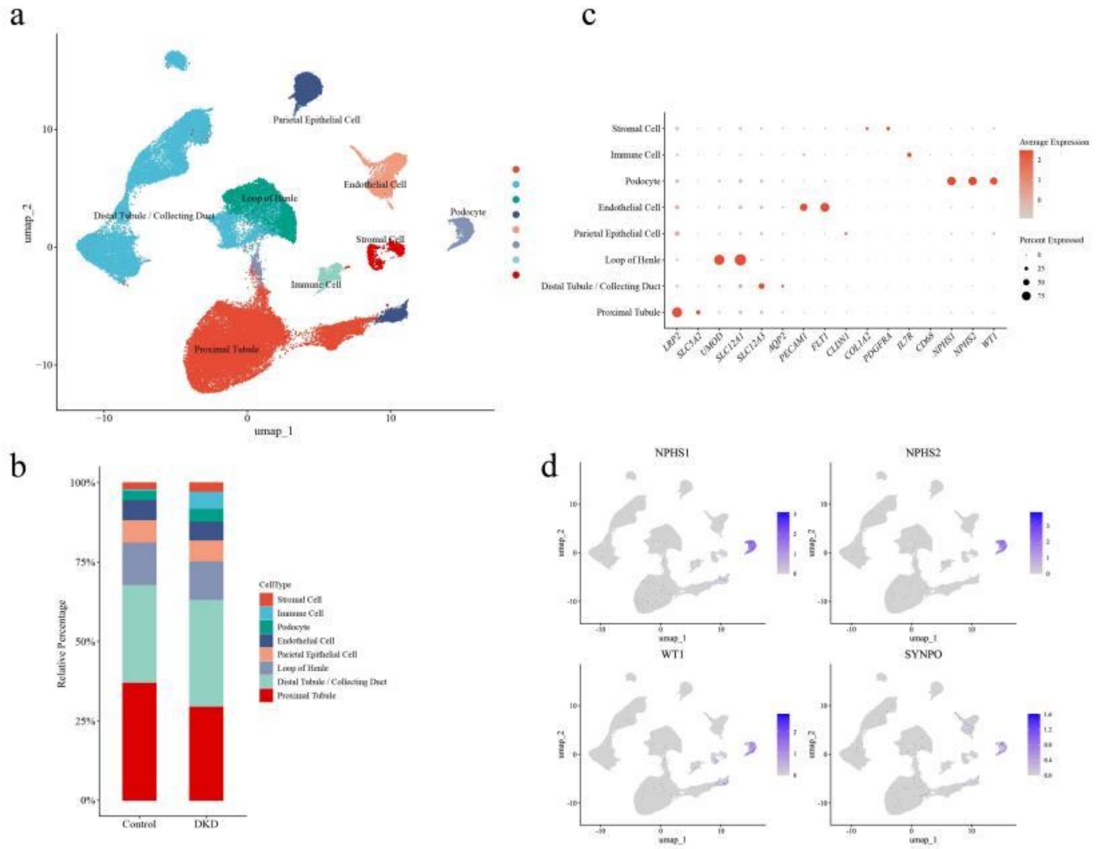

Supplementary Figure S1. Integrated single-cell atlas and marker-based annotation of renal cell lineages in DKD. (a) UMAP visualization of integrated single-cell transcriptomes from control and DKD renal biopsy samples. Cells are colored by major renal cell lineage. (b) Relative cell-type composition in control and DKD samples. (c) Dot plot showing canonical marker gene expression across annotated renal cell types. Dot size represents the percentage of expressing cells, and color indicates average expression. (d) Feature plots showing podocyte marker expression, including NPHS1, NPHS2, WT1, and SYNPO. Abbreviations: DKD, diabetic kidney disease; UMAP, Uniform Manifold Approximation and Projection; NPHS1, nephrin; NPHS2, podocin; WT1, Wilms tumor 1; SYNPO, synaptopodin.

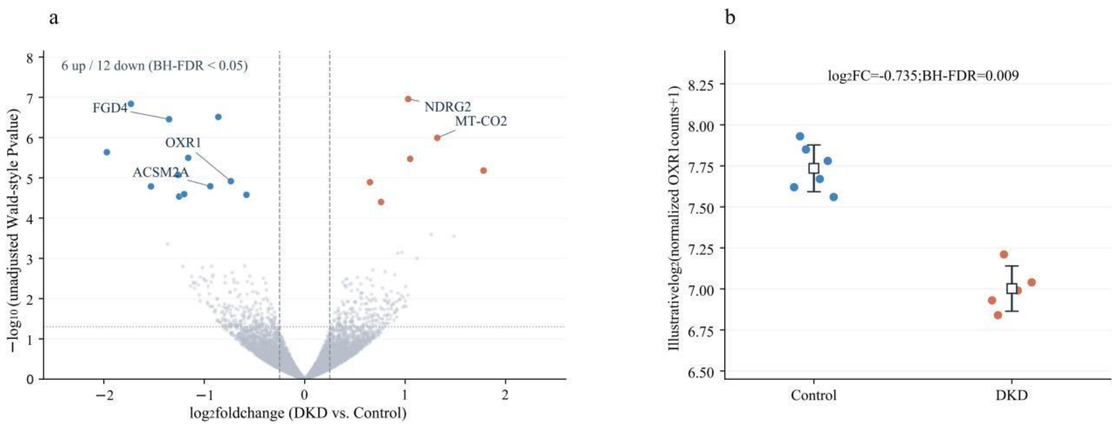

Supplementary Figure S2. Patient-level pseudobulk differential expression analysis of podocytes in the GSE195460 discovery cohort. (a) Volcano plot showing differential gene expression between DKD and control podocytes. (b) Donor-level OXR1 expression in six control and five DKD samples. Abbreviations: DKD, diabetic kidney disease; OXR1, oxidation resistance 1; BH-FDR, Benjamini–Hochberg false discovery rate; log2FC, log2 fold change.

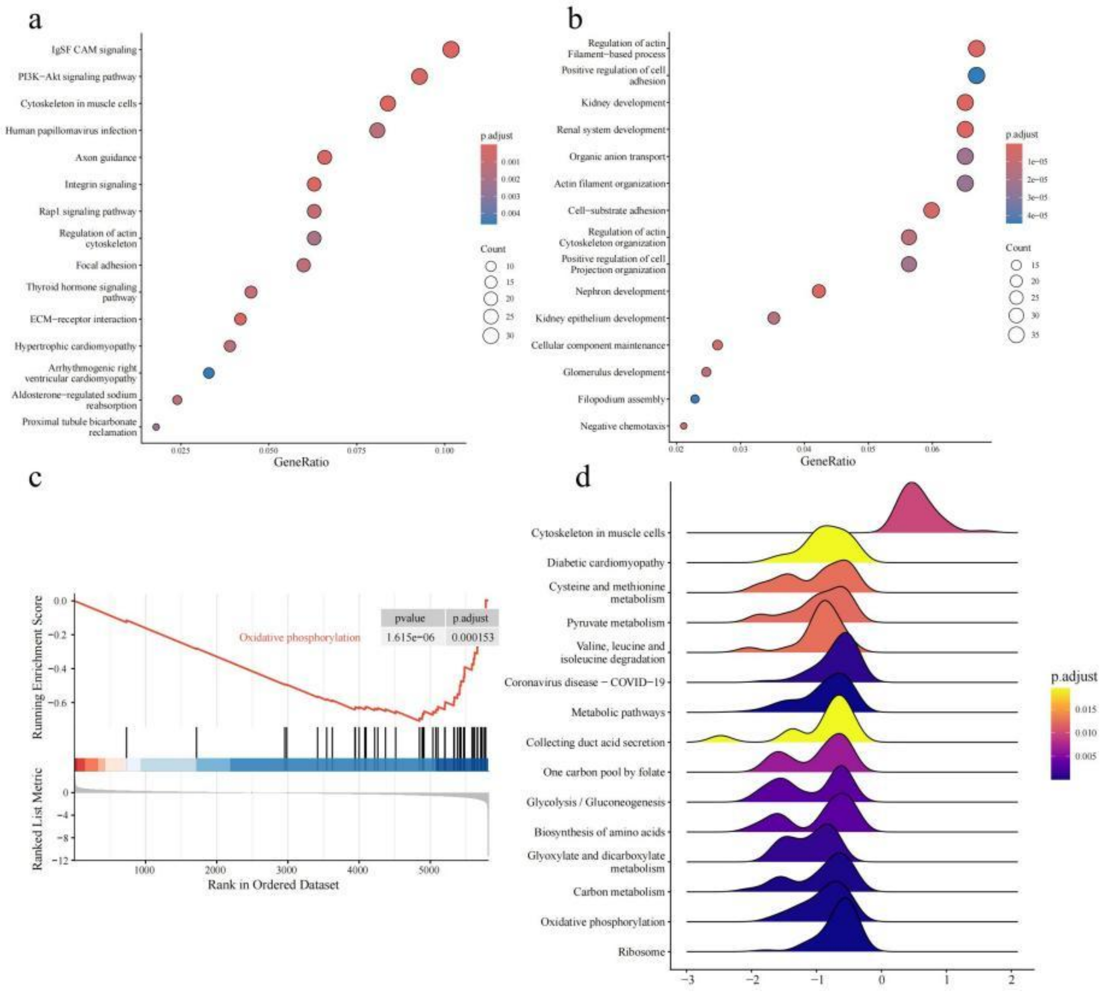

Supplementary Figure S3. Functional enrichment and gene set enrichment analyses of differentially expressed genes in DKD podocytes. (a) Kyoto Encyclopedia of Genes and Genomes (KEGG) pathway enrichment analysis of podocyte DEGs. (b) Gene Ontology (GO) enrichment analysis of podocyte DEGs. (c) Gene set enrichment analysis (GSEA) plot showing negative enrichment of the oxidative phosphorylation pathway in DKD podocytes. (d) Ridge plot showing the distributions of gene-level changes across representative KEGG pathways identified by GSEA. Abbreviations: DKD, diabetic kidney disease; DEG, differentially expressed gene; KEGG, Kyoto Encyclopedia of Genes and Genomes; GO, Gene Ontology; GSEA, gene set enrichment analysis.

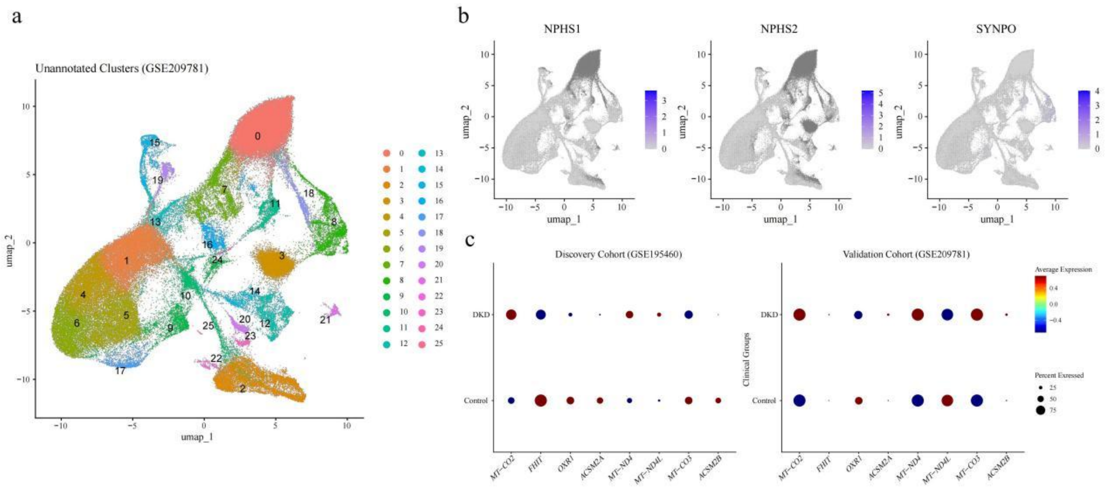

Supplementary Figure S4. External single-cell validation of mitochondrial hub gene expression in DKD podocytes. (a) UMAP visualization of the independent external single-cell cohort GSE209781. (b) Feature plots confirming podocyte-enriched regions based on NPHS1, NPHS2, and SYNPO expression. (c) Dot plot comparing selected mitochondrial hub genes between control and DKD groups in the discovery cohort GSE195460 and validation cohort GSE209781. Abbreviations: DKD, diabetic kidney disease; UMAP, Uniform Manifold Approximation and Projection; GSE, Gene Expression Omnibus Series; NPHS1, nephrin; NPHS2, podocin; SYNPO, synaptopodin.

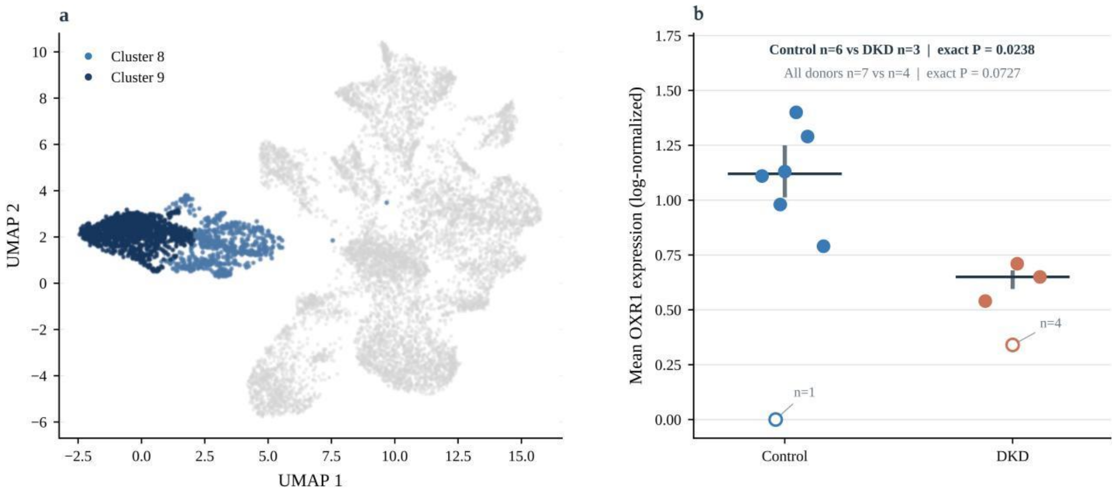

Supplementary Figure S5. External evaluation of OXR1 expression in podocytes from the independent GSE271188 cohort. (a) UMAP visualization showing the identification of podocytes corresponding to clusters 8 and 9. (b) Donor-level comparison of mean OXR1 expression between control and DKD samples. Abbreviations: DKD, diabetic kidney disease; OXR1, oxidation resistance 1; UMAP, Uniform Manifold Approximation and Projection.

a

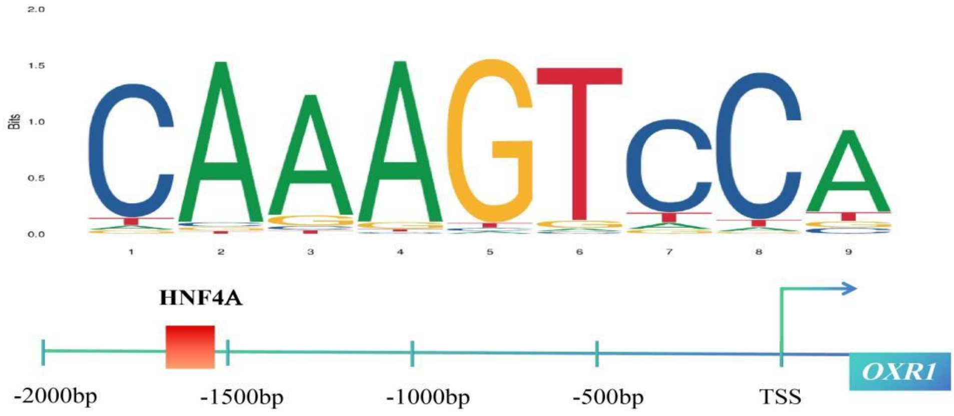

Matrix ID	Name	Score	Relative score	Sequence ID	Start	End	Strand	Predicted sequence
MA0114.5	MA0114.5.HNF4A	12.11742	0.9098152	hg38_dna	391	399	+	CAAAGTCTA

b

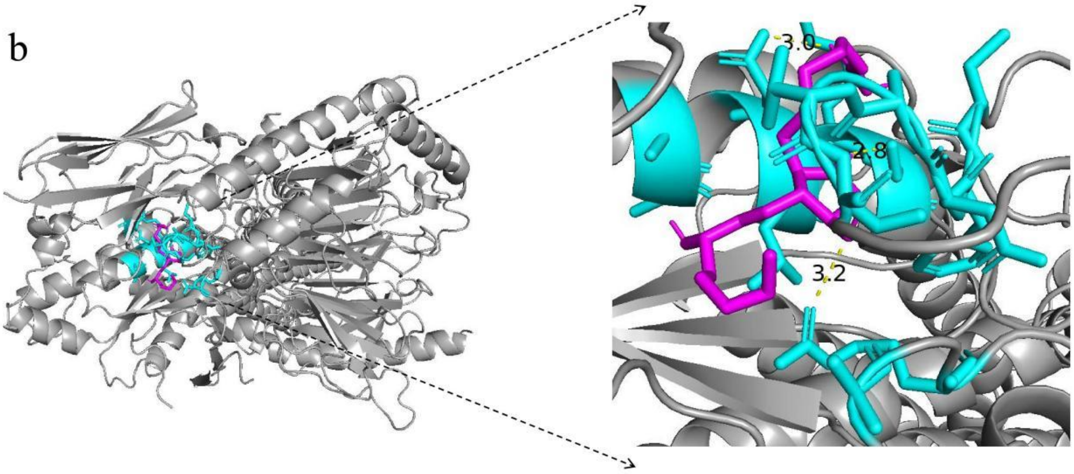

Supplementary Figure S6. Exploratory HNF4A motif analysis and PGE1–EP2 molecular docking. (a) Predicted HNF4A-binding motif within the upstream regulatory region of OXR1. The motif matches matrix MA0114.5, with a score of 12.11742, relative score of 0.9098152, and predicted sequence CAAAGTCA at positions 391–399 bp on the positive strand. (b) Exploratory molecular docking model of PGE1 with the human EP2 receptor. PGE1 is positioned within the predicted EP2 binding pocket with modeled short-range contacts of approximately 2.8–3.2 Å.
