# Supplementary material for: Single-Cell Transcriptomic and Regulatory Network Analyses Identify a Putative HNF4A–OXR1 Association Linked to Podocyte Mitochondrial Dysfunction in Diabetic Kidney Disease
Source: Int J Mol Sci. 2026 Oct 27;27(21):9608. doi: 10.3390/ijms27219608 (PMC13762632; doi:10.3390/ijms27219608)
Supplement: Supplementary file 1 [file ijms-27-09608-s001.zip › Supplementary_Tables.pdf]

Supplementary Tables

Table S1. All markers for each renal cell lineage identified in the integrated single-cell dataset.

gene	p_val	avg_log2FC	pct.1	pct.2	p_val_adj	cluster
ACSM2A	0	4.4298385634853	0.951	0.228	0	Proximal Tubule
ACSM2B	0	4.47994403165289	0.916	0.2	0	Proximal Tubule
LRP2	0	3.5072779433835	0.877	0.272	0	Proximal Tubule
SLC13A3	0	4.06787555821683	0.781	0.188	0	Proximal Tubule
FTCD	0	4.44540645789363	0.678	0.091	0	Proximal Tubule
SLC22A6	0	4.18522525437117	0.694	0.114	0	Proximal Tubule
AC096577.1	0	4.46817684581332	0.682	0.107	0	Proximal Tubule
CUBN	0	3.85845631581736	0.922	0.351	0	Proximal Tubule
SLC6A13	0	4.2101065493534	0.652	0.096	0	Proximal Tubule
SLC17A1	0	3.68924291285422	0.724	0.173	0	Proximal Tubule
AGXT2	0	4.29358197974534	0.629	0.083	0	Proximal Tubule
SLC5A12	0	3.87851166315371	0.719	0.183	0	Proximal Tubule
TINAG	0	3.60138612864886	0.693	0.157	0	Proximal Tubule
SLC2A9	0	3.61418139097412	0.697	0.162	0	Proximal Tubule
SLC16A9	0	3.84021312372383	0.681	0.152	0	Proximal Tubule
PAH	0	3.96230468674229	0.635	0.11	0	Proximal Tubule
GLYAT	0	4.05199454867457	0.609	0.085	0	Proximal Tubule
SLC13A1	0	3.99285431345297	0.63	0.115	0	Proximal Tubule
AC003044.1	0	3.81291970412689	0.639	0.127	0	Proximal Tubule
SLC4A4	0	3.22455872846246	0.847	0.339	0	Proximal Tubule
AC098588.3	0	3.53268141050421	0.684	0.185	0	Proximal Tubule
LINC01060	0	4.07448640182089	0.6	0.101	0	Proximal Tubule
SLC28A1	0	3.95436975954251	0.58	0.083	0	Proximal Tubule
AL589669.1	0	4.05261005607864	0.571	0.079	0	Proximal Tubule
Z93403.1	0	3.439373528	0.63	0.14	0	Proximal Tubule
AL391117.1	0	3.84061026895272	0.592	0.106	0	Proximal Tubule
MIOX	0	4.43212913607667	0.572	0.092	0	Proximal Tubule
PRODH2	0	4.65699218622638	0.543	0.066	0	Proximal Tubule
MME	0	2.63549879706803	0.642	0.169	0	Proximal Tubule
PDZD2	0	2.80302102620295	0.756	0.287	0	Proximal Tubule
LINC00871	0	3.64946433003058	0.599	0.136	0	Proximal Tubule
SMIM2-AS1	0	3.64246444564061	0.576	0.115	0	Proximal Tubule
DPYS	0	3.94651257751289	0.532	0.073	0	Proximal Tubule
ACSF2	0	3.59071853939544	0.584	0.126	0	Proximal Tubule
RASSF4	0	3.38620432477738	0.597	0.14	0	Proximal Tubule
ASS1	0	3.60757257781235	0.579	0.129	0	Proximal Tubule
AFM	0	4.36672773942939	0.51	0.061	0	Proximal Tubule
PLG	0	4.22574135576196	0.516	0.069	0	Proximal Tubule
ALDOB	0	3.53297918043858	0.57	0.124	0	Proximal Tubule

gene	p_val	avg_log2FC	pct.1	pct.2	p_val_adj	cluster
GPX3	0	2.73543872524065	0.658	0.214	0	Proximal Tubule
EBNA1BP2	0	3.40118766764167	0.573	0.135	0	Proximal Tubule
XYLB	0	3.46918686005993	0.535	0.106	0	Proximal Tubule
ENPEP	0	2.58560826925957	0.598	0.171	0	Proximal Tubule
RHOBTB1	0	3.38509970346925	0.564	0.137	0	Proximal Tubule
AC007364.1	0	3.16747368430276	0.564	0.138	0	Proximal Tubule
LINC01622	0	4.27827218509534	0.487	0.063	0	Proximal Tubule
AC087762.1	0	4.13785353466646	0.485	0.062	0	Proximal Tubule
SORCS1	0	3.87192093640923	0.567	0.145	0	Proximal Tubule
NOX4	0	2.93749812139564	0.647	0.227	0	Proximal Tubule
PTPRD	0	2.00677707236992	0.611	0.191	0	Proximal Tubule
SLC27A2	0	4.06587887639933	0.471	0.061	0	Proximal Tubule
PDZK1	0	3.3256063036619	0.51	0.106	0	Proximal Tubule
CYP3A5	0	4.11164419465151	0.498	0.095	0	Proximal Tubule
LINC00671	0	3.469412588	0.505	0.106	0	Proximal Tubule
ANPEP	0	3.93473014703791	0.456	0.059	0	Proximal Tubule
PRUNE2	0	2.85191557244534	0.516	0.121	0	Proximal Tubule
KCNJ15	0	2.60694116618965	0.595	0.201	0	Proximal Tubule
AL110292.1	0	3.25024230787955	0.516	0.123	0	Proximal Tubule
RAB11FIP3	0	2.75542914453676	0.851	0.459	0	Proximal Tubule
CYP4A22-AS1	0	4.34131395794831	0.448	0.058	0	Proximal Tubule
NCKAP5	0	2.35113505254372	0.768	0.378	0	Proximal Tubule
PCDH15	0	3.65374057144081	0.516	0.128	0	Proximal Tubule
HECW1	0	3.93812634887986	0.442	0.055	0	Proximal Tubule
AC009060.1	0	3.19542739034649	0.514	0.127	0	Proximal Tubule
BNC2	0	2.42218276953433	0.617	0.231	0	Proximal Tubule
NLGN1	0	2.23866057806862	0.656	0.272	0	Proximal Tubule
ACMSD	0	3.49015148558473	0.466	0.084	0	Proximal Tubule
SLC36A2	0	4.04004049881962	0.443	0.061	0	Proximal Tubule
SLC1A1	0	2.94898546699406	0.53	0.149	0	Proximal Tubule
AC027288.3	0	3.0568849654342	0.522	0.144	0	Proximal Tubule
CNTNAP3B	0	2.05076484685923	0.6	0.223	0	Proximal Tubule
PTH1R	0	1.9971193315461	0.843	0.471	0	Proximal Tubule
MIR100HG	0	2.67210792876631	0.524	0.154	0	Proximal Tubule
SUGCT	0	2.45852822521308	0.635	0.269	0	Proximal Tubule
SLC34A1	0	4.52308063557222	0.405	0.041	0	Proximal Tubule
DPEP1	0	4.5189696210563	0.393	0.034	0	Proximal Tubule
SLC47A1	0	3.47779394377381	0.452	0.093	0	Proximal Tubule
ADAMTS9-AS1	0	2.10452559002748	0.614	0.259	0	Proximal Tubule
GRAMD1B	0	2.18683678838027	0.612	0.257	0	Proximal Tubule
FGGY	0	2.40012904160333	0.776	0.422	0	Proximal Tubule
SLC17A3	0	3.94325478433616	0.401	0.052	0	Proximal Tubule

gene	p_val	avg_log2FC	pct.1	pct.2	p_val_adj	cluster
GLIS1	0	4.00645550261186	0.391	0.043	0	Proximal Tubule
KCNQ1OT1	0	1.91764005316161	0.763	0.415	0	Proximal Tubule
AK4	0	3.43958714624668	0.432	0.084	0	Proximal Tubule
L3MBTL4	0	2.06222328355517	0.751	0.404	0	Proximal Tubule
GDA	0	3.63092641615249	0.413	0.068	0	Proximal Tubule
BHMT2	0	2.58151968186839	0.552	0.211	0	Proximal Tubule
TRPM3	0	1.46093086096805	0.767	0.429	0	Proximal Tubule
UPB1	0	4.00645672178035	0.38	0.044	0	Proximal Tubule
KCNK5	0	3.88677117430704	0.391	0.056	0	Proximal Tubule
MSRA	0	2.43687493905785	0.632	0.299	0	Proximal Tubule
SLC22A8	0	4.44475169717607	0.368	0.036	0	Proximal Tubule
EHHADH	0	2.84602715364188	0.476	0.144	0	Proximal Tubule
PCSK5	0	2.18279120639011	0.48	0.151	0	Proximal Tubule
DLG2	0	1.58099159656373	0.837	0.508	0	Proximal Tubule
LINC01020	0	4.31620439657527	0.367	0.04	0	Proximal Tubule
FAM20C	0	2.83264599475782	0.459	0.132	0	Proximal Tubule
CLDN10	0	2.48481397077633	0.513	0.188	0	Proximal Tubule
CDHR3	0	2.7555866245527	0.446	0.123	0	Proximal Tubule
HNF4A	0	4.14849925085843	0.36	0.037	0	Proximal Tubule
AC018467.1	0	3.95752607953176	0.37	0.049	0	Proximal Tubule
AC087482.1	0	3.7017075476601	0.367	0.05	0	Proximal Tubule
CYP4A11	0	4.20886993694832	0.349	0.036	0	Proximal Tubule
ZNF804B	0	3.94980186845184	0.372	0.06	0	Proximal Tubule
BBOX1	0	1.97277448483712	0.445	0.133	0	Proximal Tubule
AC108519.1	0	2.22441199221347	0.586	0.274	0	Proximal Tubule
AC008554.1	0	2.813997235	0.454	0.144	0	Proximal Tubule
AC004053.1	0	4.02385068568795	0.364	0.054	0	Proximal Tubule
ABCC2	0	3.4968118385768	0.372	0.062	0	Proximal Tubule
SLC12A6	0	2.4582194707097	0.484	0.18	0	Proximal Tubule
ERRFI1	0	2.40581820293007	0.514	0.212	0	Proximal Tubule
COL27A1	0	2.32210226533032	0.46	0.159	0	Proximal Tubule
GPC6	0	1.44382537227326	0.606	0.305	0	Proximal Tubule
BHMT	0	3.31649509364043	0.379	0.079	0	Proximal Tubule
RNF212B	0	4.6683089820611	0.35	0.052	0	Proximal Tubule
SLC5A2	0	4.57563176032679	0.327	0.029	0	Proximal Tubule
ARSB	0	2.87481111358155	0.411	0.114	0	Proximal Tubule
TENM1	0	2.41680988221187	0.437	0.144	0	Proximal Tubule
MLXIPL	0	3.03162317071446	0.387	0.095	0	Proximal Tubule
ARHGAP10	0	1.66383281487773	0.436	0.145	0	Proximal Tubule
ASPA	0	2.86577637164302	0.395	0.104	0	Proximal Tubule
SLC7A7	0	3.81771422156481	0.333	0.044	0	Proximal Tubule
SLC5A10	0	4.26395047178796	0.317	0.032	0	Proximal Tubule

gene	p_val	avg_log2FC	pct.1	pct.2	p_val_adj	cluster
UGT2B7	0	2.45288525279095	0.413	0.128	0	Proximal Tubule
TPCN1	0	2.09031736370131	0.596	0.312	0	Proximal Tubule
SLC22A11	0	4.52834585633795	0.31	0.027	0	Proximal Tubule
ABCC6	0	3.1220907092582	0.357	0.075	0	Proximal Tubule
LINC01781	0	2.58224012926713	0.379	0.098	0	Proximal Tubule
CNTN4	0	1.49867256340869	0.519	0.241	0	Proximal Tubule
SLC47A2	0	4.06657151986001	0.313	0.035	0	Proximal Tubule
TMEM246-AS1	0	4.14835529447696	0.308	0.031	0	Proximal Tubule
RMST	0	3.36071149524728	0.335	0.06	0	Proximal Tubule
KIAA1549	0	2.47736677590854	0.415	0.14	0	Proximal Tubule
PPP1R16B	0	2.23525731054914	0.363	0.089	0	Proximal Tubule
FRMD4B	0	1.80370604248691	0.528	0.255	0	Proximal Tubule
CDH9	0	3.73475180015806	0.319	0.046	0	Proximal Tubule
ALDH4A1	0	3.05463105477755	0.353	0.081	0	Proximal Tubule
KHK	0	4.06424870938185	0.31	0.039	0	Proximal Tubule
PCK1	0	2.70747374901253	0.383	0.112	0	Proximal Tubule
FUT6	0	4.05838316599069	0.301	0.031	0	Proximal Tubule
BNIP3L	0	2.24649359925398	0.45	0.182	0	Proximal Tubule
CDH2	0	2.80742601353504	0.325	0.057	0	Proximal Tubule
SLC25A42	0	2.84954057737731	0.365	0.098	0	Proximal Tubule
USH1C	0	3.35760314223566	0.312	0.049	0	Proximal Tubule
P4HA2	0	3.5520744950156	0.314	0.053	0	Proximal Tubule
AMN	0	3.98911112330561	0.297	0.036	0	Proximal Tubule
HNF4A-AS1	0	4.27265454545751	0.287	0.028	0	Proximal Tubule
RHEX	0	2.48365434977214	0.358	0.101	0	Proximal Tubule
MAPT	0	3.778908932	0.299	0.042	0	Proximal Tubule
NPL	0	3.56970792796711	0.304	0.048	0	Proximal Tubule
SHMT1	0	2.83250047880915	0.34	0.086	0	Proximal Tubule
AGMO	0	3.30178795999628	0.301	0.048	0	Proximal Tubule
DOK6	0	2.25606495550002	0.33	0.077	0	Proximal Tubule
IL6R	0	2.82229583593436	0.336	0.084	0	Proximal Tubule
LINC02027	0	3.38280079212957	0.293	0.041	0	Proximal Tubule
AC004485.1	0	3.85348688207731	0.284	0.032	0	Proximal Tubule
SLC16A12	0	1.28593329872186	0.537	0.286	0	Proximal Tubule
CTSH	0	2.52673615797896	0.383	0.132	0	Proximal Tubule
SH3BP2	0	2.85909078685793	0.329	0.079	0	Proximal Tubule
CAPN12	0	3.99542687980029	0.281	0.032	0	Proximal Tubule
TTYH3	0	3.25409953912154	0.302	0.054	0	Proximal Tubule
MCF2L	0	1.83593650083433	0.523	0.275	0	Proximal Tubule
PEPD	0	2.65693411597686	0.357	0.109	0	Proximal Tubule
ATP8B4	0	3.09075140539669	0.294	0.047	0	Proximal Tubule
GALNT14	0	1.40775505681793	0.574	0.329	0	Proximal Tubule

gene	p_val	avg_log2FC	pct.1	pct.2	p_val_adj	cluster
CDH6	0	2.82819471545237	0.303	0.061	0	Proximal Tubule
PLEKHA6	0	1.95400719424404	0.501	0.26	0	Proximal Tubule
ACY1	0	3.30758152593742	0.298	0.057	0	Proximal Tubule
AL358232.2	0	3.55887229911143	0.283	0.042	0	Proximal Tubule
GADD45A	0	2.64714628845176	0.343	0.104	0	Proximal Tubule
MYO7B	0	4.00432039833881	0.265	0.026	0	Proximal Tubule
PAPLN	0	2.31826495240482	0.355	0.116	0	Proximal Tubule
SLC25A48	0	2.613845171	0.324	0.087	0	Proximal Tubule
PPARA	0	1.72568931347997	0.653	0.417	0	Proximal Tubule
GIPC2	0	2.66936448468816	0.318	0.082	0	Proximal Tubule
MIA2	0	1.74856664207597	0.598	0.363	0	Proximal Tubule
NQO2	0	2.35385344189635	0.392	0.158	0	Proximal Tubule
HPN	0	1.71312056192096	0.546	0.313	0	Proximal Tubule
SLC6A12	0	3.02950807138314	0.298	0.065	0	Proximal Tubule
CALD1	0	0.713200900676828	0.426	0.194	0	Proximal Tubule
ARHGAP42	0	1.90808845575797	0.358	0.126	0	Proximal Tubule
CHDH	0	3.60689424362912	0.269	0.038	0	Proximal Tubule
IGSF11	0	2.97139423820741	0.299	0.068	0	Proximal Tubule
MFSD4B	0	1.89127436204338	0.633	0.403	0	Proximal Tubule
MAF	0	2.65622849744339	0.303	0.073	0	Proximal Tubule
FKBP5	0	0.923813510911162	0.679	0.45	0	Proximal Tubule
CYP4F3	0	3.49024639173418	0.267	0.038	0	Proximal Tubule
AC113414.1	0	2.26979231729464	0.362	0.135	0	Proximal Tubule
LINC02147	0	3.09846170230196	0.27	0.044	0	Proximal Tubule
LINC01349	0	4.27410347876432	0.252	0.026	0	Proximal Tubule
SLC22A5	0	2.50952922292653	0.356	0.131	0	Proximal Tubule
AL591519.1	0	3.18607047680542	0.276	0.052	0	Proximal Tubule
PDZK1IP1	0	3.74091113598477	0.261	0.037	0	Proximal Tubule
SLC22A2	0	2.2821735905626	0.324	0.1	0	Proximal Tubule
TTC38	0	2.73987925682146	0.318	0.094	0	Proximal Tubule
ACO1	0	2.49462942396064	0.334	0.111	0	Proximal Tubule
LINC01515	0	1.83663019996404	0.414	0.192	0	Proximal Tubule
TNIK	0	1.54615233008377	0.354	0.134	0	Proximal Tubule
SORD	0	3.2588444414784	0.268	0.049	0	Proximal Tubule
ANK2	0	1.61069866082074	0.538	0.32	0	Proximal Tubule
LINC02275	0	3.42117874053876	0.261	0.043	0	Proximal Tubule
BAIAP2	0	2.23921899522019	0.343	0.127	0	Proximal Tubule
SLC66A2	0	2.31847855487793	0.359	0.144	0	Proximal Tubule
AC127526.2	0	2.45708599371787	0.31	0.096	0	Proximal Tubule
PTH2R	0	1.44373794281382	0.973	0.762	0	Proximal Tubule
AC079352.1	0	1.53739471719307	0.388	0.178	0	Proximal Tubule
PC	0	3.1393792128123	0.27	0.06	0	Proximal Tubule

gene	p_val	avg_log2FC	pct.1	pct.2	p_val_adj	cluster
PRR5	0	2.70434887330344	0.273	0.063	0	Proximal Tubule
GALM	0	2.47814019860896	0.289	0.081	0	Proximal Tubule
RGL1	0	1.66048401946117	0.329	0.123	0	Proximal Tubule
AFMID	0	1.86865389339001	0.396	0.191	0	Proximal Tubule
RNF152	0	1.30676494018303	0.622	0.417	0	Proximal Tubule
FBP1	0	3.34411108043096	0.256	0.052	0	Proximal Tubule
EPB41L3	0	1.47211819119468	0.589	0.385	0	Proximal Tubule
PCLO	0	1.39493326892818	0.622	0.42	0	Proximal Tubule
PDE7A	0	1.47858433760742	0.491	0.289	0	Proximal Tubule
NRP1	0	1.21509634630027	0.435	0.233	0	Proximal Tubule
LINC01277	0	2.82514472496261	0.276	0.076	0	Proximal Tubule
ABHD6	0	2.67252367368318	0.266	0.067	0	Proximal Tubule
HPD	0	3.00458293964278	0.25	0.051	0	Proximal Tubule
SNX30	0	2.11628801187343	0.359	0.16	0	Proximal Tubule
DHTKD1	0	2.0698664354759	0.35	0.151	0	Proximal Tubule
SDK1	0	1.50722700256491	0.539	0.341	0	Proximal Tubule
CRYL1	0	1.59073237217575	0.543	0.346	0	Proximal Tubule
ASTN2	0	1.43117787960077	0.483	0.288	0	Proximal Tubule
EPHA7	0	2.66394573027648	0.25	0.056	0	Proximal Tubule
SLC7A8	0	2.18084640704837	0.376	0.182	0	Proximal Tubule
CLIC4	0	1.42427855902913	0.336	0.144	0	Proximal Tubule
CELSR1	0	1.85676530707626	0.364	0.173	0	Proximal Tubule
PRLR	0	1.46197327984399	0.394	0.204	0	Proximal Tubule
ZBTB16	0	0.728669926455427	0.667	0.478	0	Proximal Tubule
LIN52	0	1.93122523253758	0.424	0.235	0	Proximal Tubule
SAMD5	0	1.08497499418774	0.484	0.298	0	Proximal Tubule
HGD	0	1.94420775805998	0.325	0.141	0	Proximal Tubule
ALDH1L1	0	1.62890126312153	0.451	0.268	0	Proximal Tubule
LINC01320	0	0.859066383829212	0.595	0.413	0	Proximal Tubule
TM7SF3	0	1.94048512160967	0.371	0.19	0	Proximal Tubule
DPF3	0	2.40770727069088	0.258	0.077	0	Proximal Tubule
EDA	0	1.71373739615427	0.343	0.163	0	Proximal Tubule
MAP3K13	0	1.50724220988889	0.523	0.343	0	Proximal Tubule
POR	0	2.06237670921259	0.316	0.137	0	Proximal Tubule
TRPV1	0	2.21615500918687	0.289	0.111	0	Proximal Tubule
ALCAM	0	1.19105281666899	0.388	0.212	0	Proximal Tubule
ABAT	0	2.35487870848581	0.264	0.088	0	Proximal Tubule
ACSS2	0	1.72023034176158	0.369	0.194	0	Proximal Tubule
TNFRSF14	0	2.04446068275355	0.274	0.1	0	Proximal Tubule
EYA2	0	1.53563182217713	0.354	0.18	0	Proximal Tubule
DNM1	0	2.12670796031438	0.266	0.093	0	Proximal Tubule
ALDH6A1	0	2.31163408629373	0.273	0.1	0	Proximal Tubule

gene	p_val	avg_log2FC	pct.1	pct.2	p_val_adj	cluster
CALM1	0	1.97985203861021	0.319	0.146	0	Proximal Tubule
NR1H4	0	1.69889357269066	0.318	0.146	0	Proximal Tubule
FP700111.1	0	2.31295728145372	0.267	0.096	0	Proximal Tubule
PARP10	0	2.13310920136272	0.267	0.097	0	Proximal Tubule
SPATS2L	0	1.37924895844382	0.444	0.274	0	Proximal Tubule
DIXDC1	0	1.76504979145166	0.342	0.173	0	Proximal Tubule
ZNF69	0	1.60953064934978	0.395	0.226	0	Proximal Tubule
RALYL	0	0.81925108707213	0.602	0.435	0	Proximal Tubule
SRRM2	0	1.32679935092058	0.687	0.523	0	Proximal Tubule
GPR155	0	2.23146111995985	0.255	0.091	0	Proximal Tubule
ZFAT	0	1.72637105393968	0.338	0.175	0	Proximal Tubule
TRABD2B	0	1.24581188344611	0.622	0.459	0	Proximal Tubule
PDGFC	0	1.41864610071788	0.418	0.256	0	Proximal Tubule
FOXP2	0	1.57206856478071	0.266	0.105	0	Proximal Tubule
DENND5B	0	1.24899553422473	0.534	0.374	0	Proximal Tubule
RTTN	0	1.82061894723557	0.376	0.216	0	Proximal Tubule
GLDC	0	1.44674123921898	0.445	0.286	0	Proximal Tubule
CAPN3	0	2.01434531030352	0.259	0.1	0	Proximal Tubule
TNS1	0	1.34562298365058	0.618	0.459	0	Proximal Tubule
ROR1	0	1.16871331365992	0.288	0.13	0	Proximal Tubule
FAT1	0	1.14447207689366	0.46	0.303	0	Proximal Tubule
MAST4	0	0.946349494616454	0.726	0.569	0	Proximal Tubule
CAMK2G	0	1.76210509757562	0.306	0.149	0	Proximal Tubule
MALT1	0	1.54949795927507	0.333	0.177	0	Proximal Tubule
NFIA	0	0.787847258901027	0.539	0.384	0	Proximal Tubule
INSR	0	1.249899373	0.698	0.543	0	Proximal Tubule
CNTN3	0	1.6574973932893	0.301	0.148	0	Proximal Tubule
CFI	0	1.83947277471211	0.295	0.142	0	Proximal Tubule
MARC2	0	1.78813695790746	0.356	0.204	0	Proximal Tubule
C11orf54	0	1.54265912694937	0.397	0.247	0	Proximal Tubule
ADCY9	0	1.28290347022794	0.365	0.215	0	Proximal Tubule
EVA1A	0	1.984468036	0.259	0.11	0	Proximal Tubule
PAX2	0	1.16594700115141	0.539	0.39	0	Proximal Tubule
GPR137B	0	1.61514948381859	0.25	0.101	0	Proximal Tubule
ULK4	0	1.60592942224051	0.563	0.415	0	Proximal Tubule
EVC	0	1.63396692791526	0.329	0.182	0	Proximal Tubule
FAM85B	0	1.48252062906328	0.396	0.249	0	Proximal Tubule
DOCK8	0	0.667545032211426	0.31	0.163	0	Proximal Tubule
D2HGDH	0	1.78042865109229	0.321	0.175	0	Proximal Tubule
EPS8L2	0	1.27576315937765	0.397	0.251	0	Proximal Tubule
OSBPL10	0	0.930839887436635	0.47	0.324	0	Proximal Tubule
SH3YL1	0	1.75574606739087	0.397	0.254	0	Proximal Tubule

gene	p_val	avg_log2FC	pct.1	pct.2	p_val_adj	cluster
TBC1D19	0	1.3796738185769	0.488	0.345	0	Proximal Tubule
SEMA5A	0	0.675059422487498	0.327	0.185	0	Proximal Tubule
PBLD	0	1.74160166676924	0.272	0.13	0	Proximal Tubule
ENTPD5	0	1.73148408574745	0.286	0.144	0	Proximal Tubule
SAT2	0	2.14911722000217	0.251	0.11	0	Proximal Tubule
LRP5	0	1.65903201412124	0.26	0.121	0	Proximal Tubule
ANKS1B	0	1.91835136845929	0.323	0.185	0	Proximal Tubule
GOLGB1	0	1.32045641475773	0.673	0.536	0	Proximal Tubule
PPP1R21	0	1.34440034862471	0.423	0.287	0	Proximal Tubule
GRB10	0	0.49503257214694	0.409	0.273	0	Proximal Tubule
PAX8	0	1.13301164952084	0.752	0.617	0	Proximal Tubule
KIF21A	0	1.21332208850211	0.366	0.231	0	Proximal Tubule
FTL	0	1.85464202533325	0.284	0.149	0	Proximal Tubule
TFPI	0	1.32233465724417	0.265	0.131	0	Proximal Tubule
CDK14	0	1.08162162952119	0.751	0.617	0	Proximal Tubule
PLEKHA5	0	1.25177561537985	0.816	0.682	0	Proximal Tubule
SGO1-AS1	0	0.810645097126313	0.466	0.334	0	Proximal Tubule
NHS	0	0.778789318285745	0.698	0.566	0	Proximal Tubule
TRPV4	0	1.49016589189273	0.284	0.152	0	Proximal Tubule
PRKCZ	0	1.55035026137834	0.308	0.177	0	Proximal Tubule
RERG	0	0.786553142688078	0.335	0.206	0	Proximal Tubule
CERS4	0	1.31219942472221	0.304	0.176	0	Proximal Tubule
VPS13A	0	1.25806233852513	0.555	0.429	0	Proximal Tubule
DISP1	0	1.3156298259243	0.367	0.241	0	Proximal Tubule
SLC22A23	0	1.02275711107212	0.363	0.237	0	Proximal Tubule
STX3	0	1.60511593154275	0.284	0.159	0	Proximal Tubule
PKHD1	0	0.553512067407099	0.93	0.807	0	Proximal Tubule
WIPF1	0	1.0900535137192	0.251	0.129	0	Proximal Tubule
DIRC3	0	1.14641275038039	0.296	0.174	0	Proximal Tubule
CCDC30	0	1.0530891742738	0.486	0.365	0	Proximal Tubule
FANCC	0	1.12937551554965	0.441	0.32	0	Proximal Tubule
PPP2R2B	0	1.67521211833723	0.29	0.17	0	Proximal Tubule
DMXL2	0	1.3621724572938	0.414	0.296	0	Proximal Tubule
AIG1	0	1.1877875913676	0.531	0.414	0	Proximal Tubule
LRMDA	0	0.376035683924664	0.852	0.735	0	Proximal Tubule
ATXN7L1	0	1.14461173634733	0.476	0.361	0	Proximal Tubule
TLN2	0	0.985735863323587	0.496	0.381	0	Proximal Tubule
TTC28	0	0.857944749475118	0.606	0.491	0	Proximal Tubule
LRRC8D	0	1.12237030406459	0.418	0.304	0	Proximal Tubule
GOLGA4	0	1.20402032391769	0.806	0.694	0	Proximal Tubule
SLC39A11	0	1.07798175602916	0.44	0.33	0	Proximal Tubule
HNF1A-AS1	0	0.859789342355667	0.528	0.42	0	Proximal Tubule

gene	p_val	avg_log2FC	pct.1	pct.2	p_val_adj	cluster
PUM3	0	1.18401585553788	0.453	0.346	0	Proximal Tubule
UBE2E2	0	0.856801570514633	0.711	0.604	0	Proximal Tubule
SYNE2	0	1.52865753209351	0.931	0.828	0	Proximal Tubule
WDR72	0	0.937159755305602	0.737	0.634	0	Proximal Tubule
NFIB	0	0.732632724358576	0.605	0.503	0	Proximal Tubule
GON4L	0	1.08869761112631	0.477	0.376	0	Proximal Tubule
ZHX3	0	0.88663576620623	0.567	0.47	0	Proximal Tubule
CMIP	0	0.925727437317243	0.514	0.427	0	Proximal Tubule
PTPRM	0	0.38365395834418	0.745	0.658	0	Proximal Tubule
SORBS2	0	0.844705586984429	0.597	0.513	0	Proximal Tubule
WDR60	0	0.936151807502639	0.551	0.468	0	Proximal Tubule
DIP2C	0	0.506849853810014	0.685	0.605	0	Proximal Tubule
PATJ	0	0.918968439508765	0.847	0.767	0	Proximal Tubule
CUX1	0	0.966173289303931	0.722	0.659	0	Proximal Tubule
FHIT	0	0.52586497239567	0.918	0.857	0	Proximal Tubule
GPHN	0	0.829903902902543	0.897	0.836	0	Proximal Tubule
PLEKHA7	0	0.833313651797786	0.61	0.55	0	Proximal Tubule
NEAT1	0	0.954056454050191	0.949	0.907	0	Proximal Tubule
FARP1	0	0.606807188969763	0.723	0.687	0	Proximal Tubule
SNX29	0	0.752836307033135	0.789	0.755	0	Proximal Tubule
WDFY3	0	0.756509724462675	0.623	0.593	0	Proximal Tubule
KIFC3	8.44243658051458e-308	1.08995580159316	0.46	0.363	2.44155265908482e-303	Proximal Tubule
RABGAP1L	8.9151668608064e-308	0.501522139445046	0.729	0.736	2.57826625614521e-303	Proximal Tubule
SLC2A11	2.14119388664784e-307	1.42458859454234	0.278	0.165	6.19233272018557e-303	Proximal Tubule
TMEM178B	7.12168011258428e-307	1.33503487494766	0.25	0.137	2.05958988855937e-302	Proximal Tubule
PLPPR1	6.74539647163924e-305	1.39489980824195	0.417	0.309	1.95076865959807e-300	Proximal Tubule
RFFL	1.90274569098696e-302	1.39623286137151	0.297	0.184	5.50274053833428e-298	Proximal Tubule
ATP11A	7.21537322592824e-299	1.06521868935203	0.35	0.228	2.08668593693845e-294	Proximal Tubule
KLHL20	2.9781482765994e-293	1.40977326110539	0.329	0.22	8.61280481592547e-289	Proximal Tubule
MACROD1	1.64255326258799e-286	1.45408581592422	0.26	0.153	4.75026403540446e-282	Proximal Tubule
ATP6V0A1	4.68911420873315e-282	1.33336530053079	0.318	0.211	1.35609182916563e-277	Proximal Tubule
EPHX2	6.96257158766078e-281	1.20098804824671	0.348	0.239	2.0135757031515e-276	Proximal Tubule
CTTNBP2	9.97954181510993e-279	1.06397712724986	0.273	0.16	2.88608349292979e-274	Proximal Tubule
ENOSF1	7.87623669531219e-277	0.929636049772045	0.543	0.479	2.27780765228428e-272	Proximal Tubule
AGPAT3	6.08649532792177e-276	1.1450193540898	0.427	0.336	1.76021444883498e-271	Proximal Tubule
COBL	6.84727899031422e-275	0.927021533002476	0.444	0.343	1.98023308399887e-270	Proximal Tubule
LINC02532	1.60052401310881e-274	0.572249346355248	0.596	0.493	4.62871544591067e-270	Proximal Tubule
PTGR1	4.49314183454652e-271	1.16454731135936	0.341	0.234	1.29941661855085e-266	Proximal Tubule
IQGAP2	1.34029436134475e-270	0.341649090376764	0.43	0.289	3.87613129300901e-266	Proximal Tubule
KIAA0825	2.73580515436262e-270	0.964513985962913	0.424	0.317	7.9119485064167e-266	Proximal Tubule
TUBGCP2	8.76279252504655e-266	1.2917057878223	0.323	0.216	2.53419959824346e-261	Proximal Tubule
OSBPL1A	4.81604900110751e-263	1.15469997561425	0.341	0.236	1.39280137112029e-258	Proximal Tubule

gene	p_val	avg_log2FC	pct.1	pct.2	p_val_adj	cluster
SFXN5	1.05921970320396e-262	1.14040027255372	0.353	0.251	3.06326338166584e-258	Proximal Tubule
DMXL1	4.87076715371698e-260	0.89792531214373	0.547	0.498	1.40862586085495e-255	Proximal Tubule
AL033504.1	1.05579426715968e-255	0.817732977393382	0.346	0.227	3.05335702062579e-251	Proximal Tubule
TGFBR3	2.0625320772101e-253	0.449102483884367	0.284	0.165	5.96484276729162e-249	Proximal Tubule
MAST2	2.96863588254682e-250	1.32753665010733	0.25	0.149	8.58529497232541e-246	Proximal Tubule
CDK10	1.09159875647476e-247	1.35360819756422	0.259	0.158	3.15690360372502e-243	Proximal Tubule
PLXDC2	1.26914654941078e-245	0.493941510342769	0.626	0.573	3.67037182089596e-241	Proximal Tubule
IGF1R	3.31367367516824e-245	0.568468690043753	0.666	0.649	9.58314426858654e-241	Proximal Tubule
DLEU2	1.58103304812149e-237	0.766765877129912	0.333	0.222	4.57234757516735e-233	Proximal Tubule
PTPRJ	1.64146930930585e-237	0.600228296692023	0.567	0.487	4.74712924251252e-233	Proximal Tubule
ACSL1	4.74021452512887e-235	1.077791131	0.371	0.278	1.37087004066727e-230	Proximal Tubule
AGAP1	9.54688820614997e-228	0.467540782169054	0.681	0.684	2.76096006921857e-223	Proximal Tubule
KANSL1L	9.93907912925365e-227	0.69854429873397	0.592	0.556	2.87438168418016e-222	Proximal Tubule
TRMT11	4.78291745023338e-226	0.885629386359727	0.452	0.367	1.38321972660749e-221	Proximal Tubule
GANC	5.00652998581718e-226	1.38049209713421	0.271	0.177	1.44788847189833e-221	Proximal Tubule
EHBP1	4.96994446879167e-223	0.711170894073612	0.591	0.543	1.43730794037455e-218	Proximal Tubule
PDE3B	1.08258793470536e-220	0.753870765816378	0.281	0.176	3.13084430716789e-216	Proximal Tubule
WWC1	1.30087687953592e-219	0.716957750239934	0.536	0.476	3.76213593561787e-215	Proximal Tubule
ACAD11	8.03332065940365e-216	1.02584726655691	0.381	0.292	2.32323633469954e-211	Proximal Tubule
AC034195.1	1.8325066866586e-214	1.07836524578127	0.334	0.238	5.29960933781668e-210	Proximal Tubule
MAN1C1	5.18174672459133e-214	1.49519075202918	0.267	0.179	1.49856115275181e-209	Proximal Tubule
AP3D1	1.06047704255699e-211	1.05856844995099	0.322	0.229	3.06689960707481e-207	Proximal Tubule
LINC00278	1.45952743252784e-211	1.72773981410909	0.284	0.209	4.22095333487051e-207	Proximal Tubule
RETREG1	1.33296599685049e-205	1.00805311569851	0.339	0.243	3.85493766289163e-201	Proximal Tubule
RNF213	1.09220423066768e-201	0.756334170768676	0.466	0.388	3.15865463509093e-197	Proximal Tubule
ANK3	6.53982978209683e-200	0.285982222810266	0.805	0.771	1.8913187729824e-195	Proximal Tubule
FBXO21	4.9433775141035e-195	1.13261918745092	0.398	0.325	1.42962477707873e-190	Proximal Tubule
BICDL1	9.14805567281639e-195	0.545564912353564	0.454	0.351	2.6456177005785e-190	Proximal Tubule
ST3GAL1	7.93901622759915e-192	0.841300797190099	0.26	0.165	2.29596349302167e-187	Proximal Tubule
CMYA5	4.91620150243486e-191	0.96479443175519	0.437	0.357	1.42176547450416e-186	Proximal Tubule
MYO15B	8.36896020430985e-190	1.04640443791649	0.335	0.244	2.42030329108641e-185	Proximal Tubule
GHR	8.33750685689408e-186	0.728895316110231	0.424	0.329	2.41120698301377e-181	Proximal Tubule
SLC16A10	2.96011116647828e-185	0.874522365848179	0.327	0.235	8.56064149345518e-181	Proximal Tubule
MST1	1.08168634824827e-184	1.17839407759036	0.291	0.206	3.128236919134e-180	Proximal Tubule
SAFB2	2.69405564280139e-184	0.99758266505707	0.383	0.309	7.79120891898162e-180	Proximal Tubule
HIBCH	7.06220104359627e-180	0.805049601190239	0.527	0.493	2.04238854180804e-175	Proximal Tubule
NEO1	1.40755327527757e-176	0.958392589097712	0.353	0.275	4.07064407210274e-172	Proximal Tubule
FTX	5.05551882019803e-172	0.30643487135339	0.86	0.871	1.46205604280127e-167	Proximal Tubule
GALNT2	7.90390498959826e-170	1.06440107335153	0.274	0.193	2.28580932299182e-165	Proximal Tubule
TNS3	2.34551045449606e-167	0.614499296996801	0.565	0.532	6.7832162344026e-163	Proximal Tubule
HPCAL1	1.50474207128161e-166	0.91073304142871	0.319	0.237	4.3517140701464e-162	Proximal Tubule
RBM47	6.47460561345198e-166	0.476349755929411	0.627	0.601	1.87245594341031e-161	Proximal Tubule

gene	p_val	avg_log2FC	pct.1	pct.2	p_val_adj	cluster
SLC3A1	1.16914592893847e-165	0.843893396603831	0.374	0.292	3.38117002649005e-161	Proximal Tubule
ARHGEF10L	1.60787455286441e-163	0.796740292070677	0.424	0.357	4.64997320688387e-159	Proximal Tubule
SUCLG1	1.80768084673863e-162	1.30516760475582	0.327	0.259	5.22781300876813e-158	Proximal Tubule
AC008014.1	2.18967478324523e-161	0.513153172770263	0.617	0.563	6.33253947314521e-157	Proximal Tubule
LARP4	8.2180805123027e-161	0.882975785876492	0.462	0.412	2.37666888415794e-156	Proximal Tubule
LPIN2	5.57932622485875e-160	1.0769876841892	0.291	0.211	1.61354114422915e-155	Proximal Tubule
ZNF75D	1.16237129321381e-159	1.04748401942411	0.307	0.229	3.36157777997433e-155	Proximal Tubule
CABLES1	1.85512387232642e-158	0.740798062378751	0.259	0.173	5.36501823876802e-154	Proximal Tubule
LARP1B	4.69783630555502e-157	0.885555590053805	0.389	0.32	1.35861425956651e-152	Proximal Tubule
YBX3	1.48833507884467e-153	0.973622247939088	0.327	0.254	4.30426504801879e-149	Proximal Tubule
GLG1	2.87007082237193e-153	0.717605825291062	0.535	0.514	8.30024481829961e-149	Proximal Tubule
ZKSCAN1	8.48091299482526e-150	0.972226655904301	0.339	0.269	2.45268003810347e-145	Proximal Tubule
FREM2	4.47747734869393e-148	0.802939493797505	0.313	0.231	1.29488644924228e-143	Proximal Tubule
MICAL3	1.69522359649486e-145	0.724547248000809	0.457	0.404	4.90258664106312e-141	Proximal Tubule
SHROOM4	3.55837528190786e-145	0.881465691074092	0.365	0.288	1.02908213152775e-140	Proximal Tubule
SPEF2	1.23633677276148e-142	1.15843091418899	0.31	0.239	3.5754859468262e-138	Proximal Tubule
LARP1	1.63576431593266e-141	0.849896546519173	0.4	0.341	4.73063040167727e-137	Proximal Tubule
DHRSX	4.4019198303619e-139	0.790788032552662	0.442	0.388	1.27303521494066e-134	Proximal Tubule
KIF12	1.16905484641303e-138	1.00126355176806	0.348	0.28	3.38090661582649e-134	Proximal Tubule
MT-CO1	1.31836803927178e-137	0.404794463646081	0.696	0.667	3.81272036957398e-133	Proximal Tubule
ZC3H12C	2.60727716022099e-131	0.96740472512584	0.277	0.206	7.5402455473591e-127	Proximal Tubule
RUFY3	3.40133008374248e-131	0.677075360978073	0.488	0.453	9.83664660218326e-127	Proximal Tubule
PARD3	7.65804185757245e-131	0.3466296376766	0.716	0.733	2.21470570520995e-126	Proximal Tubule
KIAA1671	1.87711124829593e-129	0.755158861160591	0.39	0.332	5.42860573007183e-125	Proximal Tubule
NPLOC4	7.98873648673605e-128	1.01255611370061	0.325	0.261	2.31034259196407e-123	Proximal Tubule
CLUH	1.70340575108277e-127	1.04339966585736	0.264	0.194	4.92624943213138e-123	Proximal Tubule
DMGDH	2.70822872622498e-127	0.858125694728724	0.359	0.293	7.83219747624264e-123	Proximal Tubule
GAK	3.5221967139301e-127	0.83182039711995	0.345	0.277	1.01861928966858e-122	Proximal Tubule
ARHGAP32	1.84652856313294e-125	0.758261358166608	0.43	0.377	5.34016060458047e-121	Proximal Tubule
NRG1	5.50758815851068e-125	0.830427767676953	0.26	0.187	1.59279449544129e-120	Proximal Tubule
FNIP1	1.25033115801887e-124	0.699900924977609	0.489	0.453	3.61595770899056e-120	Proximal Tubule
FP236383.3	2.22596268870724e-124	0.465676436721721	0.771	0.791	6.43748409574132e-120	Proximal Tubule
NDRG1	2.76410387521416e-121	0.493112359704477	0.423	0.36	7.99378840711936e-117	Proximal Tubule
PCAT1	2.04108668073786e-120	0.57580307193267	0.475	0.409	5.90282268069389e-116	Proximal Tubule
P3H2	1.12996848179734e-119	0.43012337520281	0.505	0.441	3.26786884935791e-115	Proximal Tubule
FRMD3	2.29597049256869e-119	0.299920822381088	0.428	0.339	6.63994666450866e-115	Proximal Tubule
AASS	2.11606220716486e-116	1.00746388600172	0.332	0.276	6.11965190312079e-112	Proximal Tubule
ANKHD1	4.88122268539004e-116	0.603071809292184	0.587	0.589	1.4116496006148e-111	Proximal Tubule
ZNF462	8.53205606950203e-116	0.858936967738119	0.284	0.214	2.46747061529999e-111	Proximal Tubule
CACNA2D1	4.28251171562177e-115	0.600279986299846	0.353	0.281	1.23850238815782e-110	Proximal Tubule
LRRK2	5.99448182717396e-115	0.804817115248483	0.328	0.262	1.73360414441871e-110	Proximal Tubule
FBXO17	2.15087622769919e-114	0.875605425556292	0.33	0.268	6.22033405050605e-110	Proximal Tubule

gene	p_val	avg_log2FC	pct.1	pct.2	p_val_adj	cluster
CNKSR3	2.72512948885227e-114	0.53015559044674	0.482	0.44	7.88107448176076e-110	Proximal Tubule
INVS	2.04262496934095e-109	0.707568707302315	0.463	0.423	5.90727141133401e-105	Proximal Tubule
TUBGCP3	2.31051485120817e-106	1.10069252140997	0.282	0.225	6.68200894969403e-102	Proximal Tubule
MAPK10	3.15395207088725e-106	0.333928233305966	0.726	0.697	9.12122938900592e-102	Proximal Tubule
CYB5A	1.15356322202322e-105	1.00515985544177	0.271	0.211	3.33610483809116e-101	Proximal Tubule
RREB1	2.19221473635732e-102	0.734414524050022	0.413	0.373	6.33988501754536e-98	Proximal Tubule
EIF1B-AS1	4.83930547062564e-101	0.820705961075255	0.264	0.2	1.39952714210494e-96	Proximal Tubule
TNRC6A	6.32919078857008e-100	0.502242542348028	0.641	0.672	1.83040197605447e-95	Proximal Tubule
COL18A1	1.00029788085848e-99	0.647740412759424	0.477	0.445	2.89286147144272e-95	Proximal Tubule
TTC21B	5.69133862221994e-98	1.05062076571313	0.296	0.243	1.64593512954601e-93	Proximal Tubule
SIPA1L3	1.24926576648114e-97	0.653100742766621	0.498	0.48	3.61287659666346e-93	Proximal Tubule
PCBP1-AS1	1.76159939325783e-97	0.873587279736567	0.31	0.254	5.09454544530163e-93	Proximal Tubule
MET	1.8103583047414e-97	0.744610939583292	0.272	0.208	5.23555621731212e-93	Proximal Tubule
ELL2	1.13434288860962e-96	0.691551259615309	0.319	0.26	3.28051963385902e-92	Proximal Tubule
AKAP13	1.352315811662e-96	0.401983867039037	0.591	0.593	3.9108973273265e-92	Proximal Tubule
VMP1	1.82814398047009e-95	0.627785245580038	0.438	0.4	5.2869923915195e-91	Proximal Tubule
NFATC3	5.15223152066498e-95	0.79445574076882	0.35	0.3	1.49002535577631e-90	Proximal Tubule
ZNF385B	2.79401655484345e-94	1.12717641883391	0.265	0.21	8.08029587660727e-90	Proximal Tubule
ADD1	7.25193222335959e-94	0.836885980229919	0.39	0.353	2.09725879899559e-89	Proximal Tubule
BEND7	8.31176983922895e-94	0.945313762118162	0.258	0.201	2.40376383750501e-89	Proximal Tubule
STON2	3.82036378461891e-92	0.835481953494827	0.297	0.241	1.10484920651179e-87	Proximal Tubule
RIN2	6.37477622915219e-90	0.548056805082261	0.471	0.437	1.84358528547081e-85	Proximal Tubule
AUTS2	1.91152071717365e-89	0.316242090042127	0.46	0.402	5.52811791406618e-85	Proximal Tubule
WDR6	7.51013873238111e-89	1.01250162072886	0.27	0.216	2.17193212140462e-84	Proximal Tubule
ZNF91	2.66948264336951e-87	0.697009620448441	0.511	0.501	7.72014380462463e-83	Proximal Tubule
ARHGEF28	6.50429495102778e-85	0.563359754067377	0.494	0.476	1.88104209983723e-80	Proximal Tubule
DENND1A	7.40640036371881e-83	0.528942862952971	0.537	0.544	2.14193098518748e-78	Proximal Tubule
TAF1D	5.94357410318296e-81	0.708056380108855	0.429	0.396	1.71888163064051e-76	Proximal Tubule
SPEN	6.40068678878707e-81	0.819066381131493	0.414	0.381	1.85107861931722e-76	Proximal Tubule
SLC43A2	1.19366412793065e-79	0.84498931512399	0.331	0.285	3.45207665797544e-75	Proximal Tubule
PPARG	1.89798591925466e-79	0.29331118121743	0.318	0.248	5.48897527848449e-75	Proximal Tubule
MSH3	3.53393372112232e-79	0.761301266129597	0.383	0.348	1.02201363214857e-74	Proximal Tubule
NCOA7	6.81319628029657e-79	0.338301565357636	0.4	0.335	1.97037636426177e-74	Proximal Tubule
N4BP2L2	1.7488313683264e-78	0.440563678716611	0.729	0.752	5.05762031719996e-74	Proximal Tubule
SELENOO	2.94274951341958e-78	0.911822408869107	0.253	0.202	8.51043159280942e-74	Proximal Tubule
ANKRD26	6.06564006762223e-78	0.607073983491503	0.497	0.473	1.75418310755635e-73	Proximal Tubule
ECHDC2	3.11146527001561e-77	0.728740507302893	0.442	0.426	8.99835756088515e-73	Proximal Tubule
SCP2	1.1739081585817e-76	0.895635740196845	0.308	0.262	3.39494239461826e-72	Proximal Tubule
XPR1	3.97635236964723e-76	0.61158084312648	0.425	0.398	1.14996110530198e-71	Proximal Tubule
OSBPL8	2.0817081376632e-74	0.602220803715111	0.418	0.386	6.02029993412198e-70	Proximal Tubule
IFT88	4.61349084424436e-72	0.744133261911237	0.398	0.37	1.33422155215547e-67	Proximal Tubule
NRIP1	8.23819198559955e-72	0.5965429982596	0.442	0.417	2.38248512223539e-67	Proximal Tubule

gene	p_val	avg_log2FC	pct.1	pct.2	p_val_adj	cluster
LINC00486	1.80097128862563e-70	0.59736967793566	0.304	0.253	5.20840896670531e-66	Proximal Tubule
ESF1	5.54516954657289e-70	0.885100291470161	0.282	0.234	1.60366303286888e-65	Proximal Tubule
SGK3	1.80553736200901e-66	0.718329805289678	0.376	0.348	5.22161405093005e-62	Proximal Tubule
NBEA	3.31602753404367e-66	0.555996886429455	0.472	0.453	9.5899516284543e-62	Proximal Tubule
OTUD7A	4.43411890026023e-66	0.735960379538785	0.291	0.245	1.28234718595526e-61	Proximal Tubule
ANKIB1	1.82674143665869e-65	0.623989976070153	0.449	0.435	5.28293623481693e-61	Proximal Tubule
CDR2	2.18028538631014e-65	0.616580051987784	0.404	0.372	6.30538533720891e-61	Proximal Tubule
CPM	5.83829612352794e-65	0.445412631852188	0.395	0.346	1.68843523892428e-60	Proximal Tubule
ACSS3	6.41645419247709e-63	0.694182313000461	0.327	0.284	1.85563855246437e-58	Proximal Tubule
KDM4B	6.444977009578e-63	0.7580578377416	0.316	0.276	1.86388735116996e-58	Proximal Tubule
GMDS-DT	2.63848107295935e-62	0.294732841583637	0.665	0.683	7.63048726299843e-58	Proximal Tubule
SH3D19	3.06278057855565e-62	0.410410835734374	0.409	0.369	8.85756143318294e-58	Proximal Tubule
ERI3	1.09275768259107e-61	0.736208050674948	0.299	0.261	3.16025521805339e-57	Proximal Tubule
MT-CO3	5.26787806438613e-61	0.380029662693421	0.482	0.44	1.52347033622047e-56	Proximal Tubule
MICU1	6.7925666787435e-61	0.735761555964256	0.375	0.349	1.96441028349262e-56	Proximal Tubule
PELI2	1.89302993384922e-60	0.590107611964186	0.449	0.42	5.47464256869196e-56	Proximal Tubule
ACVR1	9.59431869165801e-59	0.716398674426912	0.305	0.267	2.7746769656275e-54	Proximal Tubule
DHFR	5.47486125941611e-57	0.285927462667349	0.422	0.38	1.58332987622314e-52	Proximal Tubule
SIPA1L1	1.82150464633518e-56	0.412490222437956	0.502	0.491	5.26779143720133e-52	Proximal Tubule
SHANK2	3.93083852819654e-56	0.325611472463123	0.597	0.603	1.13679850235444e-51	Proximal Tubule
SETD3	5.97088221594301e-56	0.754098695703949	0.321	0.291	1.72677913685072e-51	Proximal Tubule
SMARCC1	8.6198489260052e-56	0.64003909781213	0.427	0.415	2.4928603094007e-51	Proximal Tubule
NINL	1.22319627974237e-55	0.529542695534507	0.45	0.432	3.53748364101493e-51	Proximal Tubule
HSP90B1	1.09646853491046e-54	0.687560414297903	0.268	0.227	3.17098700296105e-50	Proximal Tubule
PDZD8	2.61125968759408e-53	0.802180826655378	0.295	0.261	7.55176301652208e-49	Proximal Tubule
ASH1L	5.39314979249962e-53	0.343026872332291	0.646	0.688	1.55969891999089e-48	Proximal Tubule
FNIP2	2.89864895327181e-52	0.50498096837927	0.47	0.457	8.38289277286207e-48	Proximal Tubule
MT-CO2	1.27810984834605e-50	0.370609602676168	0.488	0.45	3.69629368141677e-46	Proximal Tubule
CFDP1	4.47131472047877e-50	0.613252811516441	0.407	0.391	1.29310421716246e-45	Proximal Tubule
TFDP2	2.24695400576797e-49	0.570657253401706	0.461	0.451	6.49819098468096e-45	Proximal Tubule
AC087564.1	2.64092972341955e-48	0.577117262536221	0.271	0.228	7.63756876012933e-44	Proximal Tubule
RFX7	5.39979442683654e-48	0.571937256040788	0.376	0.347	1.56162054824113e-43	Proximal Tubule
NR3C1	2.10300597717719e-46	0.365930995799485	0.271	0.223	6.08189328599643e-42	Proximal Tubule
CCDC144A	5.01719433515504e-46	0.599601183159329	0.311	0.276	1.45097260172684e-41	Proximal Tubule
RAI14	2.07238175216157e-45	0.477483852941971	0.411	0.381	5.99332802725125e-41	Proximal Tubule
CCDC198	2.10486600905417e-44	0.436862353328892	0.49	0.489	6.08727249818466e-40	Proximal Tubule
PRPF4B	2.7000812809009e-44	0.634603918728935	0.417	0.408	7.80863506436541e-40	Proximal Tubule
TTC39B	4.50529877752094e-44	0.66168739754209	0.276	0.24	1.30293240645906e-39	Proximal Tubule
DDAH1	5.61779123124974e-43	0.439936136041302	0.389	0.356	1.62466522407743e-38	Proximal Tubule
SLC25A16	1.62894259081365e-42	0.640894501074589	0.377	0.36	4.71090197263308e-38	Proximal Tubule
DIAPH2	5.52434473077611e-42	0.440844843575259	0.296	0.255	1.59764049614045e-37	Proximal Tubule
SASH1	1.75989615793885e-41	0.331179444609369	0.408	0.376	5.08961968875914e-37	Proximal Tubule

gene	p_val	avg_log2FC	pct.1	pct.2	p_val_adj	cluster
KANTR	8.18199846061503e-41	0.693762796621971	0.327	0.301	2.36623395480987e-36	Proximal Tubule
ZBTB38	3.37587587724883e-39	0.706509480830414	0.308	0.283	9.76303303700363e-35	Proximal Tubule
PPP2R2D	2.11113075635721e-38	0.697469931533345	0.339	0.32	6.10539014738505e-34	Proximal Tubule
WDPCP	3.27246161353515e-37	0.455532169136658	0.457	0.45	9.46395898634364e-33	Proximal Tubule
ANKRD12	9.0509933393437e-37	0.358697750023791	0.605	0.638	2.6175472737382e-32	Proximal Tubule
PDZRN3	4.34463221313478e-36	0.321376729432984	0.367	0.331	1.25646763603858e-31	Proximal Tubule
LCLAT1	5.17941734657028e-36	0.672235635350749	0.331	0.31	1.49788749662813e-31	Proximal Tubule
SPAG9	6.88695364834946e-36	0.443765797559091	0.495	0.505	1.99170699510267e-31	Proximal Tubule
HSPD1	1.52425515483963e-35	0.718870897860189	0.271	0.244	4.4081459077962e-31	Proximal Tubule
CHD9	2.38997669232834e-35	0.287239624351767	0.704	0.752	6.91181259421355e-31	Proximal Tubule
EZR	5.66303612895248e-35	0.479534780773633	0.336	0.309	1.63775004849306e-30	Proximal Tubule
CWF19L2	3.06723474242524e-34	0.639466527097798	0.376	0.362	8.8704428750938e-30	Proximal Tubule
TPT1-AS1	3.59913396111212e-34	0.578921841874154	0.395	0.388	1.04086954155362e-29	Proximal Tubule
AC009120.2	1.77436607531624e-33	0.567334670957575	0.412	0.403	5.13146668981455e-29	Proximal Tubule
MT-ND1	8.99804689878279e-33	0.451234595543755	0.355	0.328	2.60223516312798e-28	Proximal Tubule
TRIP11	1.2558266322735e-32	0.603039022508577	0.412	0.409	3.63185062053495e-28	Proximal Tubule
DNM2	1.31531950657691e-32	0.452102112249151	0.438	0.433	3.80390401302043e-28	Proximal Tubule
SNRNP70	3.75497633370272e-31	0.563962032159969	0.397	0.39	1.08593915570683e-26	Proximal Tubule
PPP2R3A	3.90663778037095e-31	0.250805232522628	0.662	0.68	1.12979964608328e-26	Proximal Tubule
STX7	4.86641968612874e-31	0.595999221361404	0.302	0.281	1.40736857322843e-26	Proximal Tubule
RRBP1	5.48989957219515e-30	0.460194646587758	0.339	0.319	1.58767895627884e-25	Proximal Tubule
TRPS1	5.79092962333155e-29	0.323573205409764	0.524	0.534	1.67473684706748e-24	Proximal Tubule
CRPPA	7.59314321054116e-29	0.56251127260195	0.282	0.255	2.1959370164885e-24	Proximal Tubule
PALM2-AKAP2	2.61938645125865e-28	0.311855404905143	0.33	0.302	7.57526561704e-24	Proximal Tubule
SPTLC3	3.44854865324458e-28	0.480841321759007	0.293	0.266	9.97320270518333e-24	Proximal Tubule
FBXO34	7.59401306858247e-28	0.412988416221592	0.411	0.401	2.19618857943405e-23	Proximal Tubule
HMGN3	1.69041729934029e-27	0.667787618371358	0.273	0.253	4.88868682969212e-23	Proximal Tubule
MT-ATP6	6.77822209113919e-26	0.453564691021749	0.333	0.308	1.96026182875745e-21	Proximal Tubule
PLIN5	3.14452018377626e-25	0.440274841928504	0.287	0.263	9.09395237148094e-21	Proximal Tubule
MT-ND4	1.68968831361673e-24	0.357233981436972	0.381	0.359	4.88657860297959e-20	Proximal Tubule
LUC7L3	3.58530927867402e-24	0.325356923731062	0.649	0.697	1.03687144339253e-19	Proximal Tubule
SCMH1	5.12812191879977e-24	0.390772147312396	0.507	0.522	1.48305285891689e-19	Proximal Tubule
XIST	6.13044351467616e-24	0.437714583149795	0.432	0.442	1.77292426444435e-19	Proximal Tubule
WDR59	9.66940490906029e-23	0.632998126253151	0.318	0.307	2.79639189970024e-18	Proximal Tubule
CSPP1	2.30641060581909e-22	0.549719769120217	0.369	0.363	6.67013947202881e-18	Proximal Tubule
TJP2	4.43953346265532e-21	0.527872991431908	0.347	0.34	1.28391307739992e-16	Proximal Tubule
MT-ND2	5.37448854219697e-21	0.393566337848629	0.327	0.305	1.55430208640336e-16	Proximal Tubule
PRRC2C	1.26265402481152e-20	0.402019354729386	0.558	0.601	3.65159543975491e-16	Proximal Tubule
CLYBL	1.0670947461521e-19	0.447142739264975	0.365	0.359	3.08603800587187e-15	Proximal Tubule
MGMT	4.38929824754179e-19	0.428837271670561	0.324	0.31	1.26938505318908e-14	Proximal Tubule
AHCTF1	9.90812263129561e-19	0.280431798873089	0.239	0.291	2.86542906497069e-14	Proximal Tubule
PTPRK	1.08493217105921e-18	0.327418563513468	0.488	0.512	3.13762383870324e-14	Proximal Tubule

gene	p_val	avg_log2FC	pct.1	pct.2	p_val_adj	cluster
STPG2	1.18751308739561e-18	0.381550715717643	0.407	0.398	3.43428784874812e-14	Proximal Tubule
MYO5B	1.20975612117564e-18	0.549071116328352	0.285	0.271	3.49861470243996e-14	Proximal Tubule
MAPKAP1	3.10808476787148e-18	0.544868703677954	0.408	0.427	8.98858114868433e-14	Proximal Tubule
DDX24	7.26578342854877e-18	0.26927449010689	0.246	0.298	2.10126456753631e-13	Proximal Tubule
DIP2B	1.82706439372049e-17	0.420423358811716	0.41	0.418	5.28387022663965e-13	Proximal Tubule
ITGB8	3.17353552950338e-17	0.605456093198784	0.303	0.287	9.17786475132379e-13	Proximal Tubule
MAPK14	3.67045350304871e-17	0.547756024069778	0.315	0.306	1.06149515308169e-12	Proximal Tubule
RDX	6.90670464174244e-17	1.07877546188661	0.322	0.327	1.99741898239191e-12	Proximal Tubule
USP8	8.9426635178874e-17	0.552980176653367	0.332	0.33	2.58621828937304e-12	Proximal Tubule
CFLAR	1.62602506615302e-16	0.337753757545994	0.483	0.498	4.70246449131454e-12	Proximal Tubule
ENTPD1-AS1	2.74321800010023e-16	0.391002736235454	0.319	0.302	7.93338645628986e-12	Proximal Tubule
RNF19A	2.90606575651341e-16	0.403835539306061	0.389	0.39	8.40434216783679e-12	Proximal Tubule
MAP7	3.78174688555436e-16	0.407323580836692	0.431	0.442	1.09368119930232e-11	Proximal Tubule
IGF2R	5.96632644173283e-16	0.447736820615626	0.451	0.473	1.72546160694913e-11	Proximal Tubule
PDXDC1	7.29467871832374e-16	0.536953025133837	0.362	0.369	2.10962108533923e-11	Proximal Tubule
AEBP2	8.0152901219389e-16	0.385715184295145	0.436	0.445	2.31802190326473e-11	Proximal Tubule
PLEKHA1	1.40102273979374e-15	0.514425378635611	0.314	0.309	4.0517577634835e-11	Proximal Tubule
THOC2	1.77608415668275e-15	0.536557785454697	0.404	0.419	5.1364353811265e-11	Proximal Tubule
LDAH	1.30588466057095e-14	0.523646068099895	0.28	0.27	3.7766184383712e-10	Proximal Tubule
GALK2	1.5202300407271e-14	0.523509687451552	0.295	0.287	4.39650527778278e-10	Proximal Tubule
KCNJ3	1.6171114258726e-14	0.258286062583473	0.344	0.323	4.67668624362357e-10	Proximal Tubule
PRKRIP1	1.93520969409788e-14	0.523860508967505	0.349	0.353	5.59662643533108e-10	Proximal Tubule
GSE1	2.04249417443339e-14	0.287503938468683	0.364	0.358	5.90689315246135e-10	Proximal Tubule
HSDL2	4.17157309426507e-14	0.607774998939421	0.261	0.251	1.20641893886146e-09	Proximal Tubule
TSPAN9	1.50830679551103e-13	0.412240009714846	0.474	0.511	4.3620232526179e-09	Proximal Tubule
WASHC1	1.35979707638652e-12	0.5359893826649	0.386	0.397	3.93253314490981e-08	Proximal Tubule
GAPVD1	1.38362577830292e-12	0.598787147135951	0.296	0.293	4.00144575085205e-08	Proximal Tubule
KRIT1	1.4335433117704e-12	0.312548030286807	0.248	0.295	4.14580725763998e-08	Proximal Tubule
CASC4	2.2695170185275e-12	0.450503597375476	0.391	0.399	6.56344321758153e-08	Proximal Tubule
SETD2	2.29156825782223e-12	0.372177073772204	0.552	0.6	6.62721540162188e-08	Proximal Tubule
SREK1	1.82414635615826e-11	0.267554192556919	0.335	0.396	5.27543126200969e-07	Proximal Tubule
PPM1H	7.86836377577559e-11	0.41708279175634	0.311	0.309	2.2755308039543e-06	Proximal Tubule
ALMS1	1.58086294556331e-10	0.269558612200739	0.28	0.328	4.57185563856908e-06	Proximal Tubule
SHLD2	2.18104491328154e-10	0.409887473946851	0.393	0.402	6.3075818892102e-06	Proximal Tubule
PDPK1	8.90510969966298e-10	0.501673396368301	0.253	0.246	2.57535772514253e-05	Proximal Tubule
SCYL2	9.519828922811e-10	0.282418485920603	0.236	0.276	2.75313452447694e-05	Proximal Tubule
CDK12	1.60128862474527e-09	0.290225309736186	0.314	0.366	4.63092670276331e-05	Proximal Tubule
DGLUCY	5.48976890463308e-09	0.400188486876572	0.366	0.373	0.000158764116721989	Proximal Tubule
CDKL1	5.82975337431252e-09	0.446154512128788	0.295	0.291	0.000168596467585118	Proximal Tubule
BOD1L1	6.05780238890093e-09	0.466169780068691	0.376	0.389	0.000175191645087015	Proximal Tubule
MT-CYB	6.74374166004505e-09	0.360691510441007	0.316	0.309	0.000195029008808503	Proximal Tubule
TOGARAM1	7.11665578794639e-09	0.261396146293085	0.26	0.3	0.00020581368538741	Proximal Tubule

gene	p_val	avg_log2FC	pct.1	pct.2	p_val_adj	cluster
CEP350	1.79999828624066e-08	0.26527955450476	0.384	0.449	0.000520559504380798	Proximal Tubule
FARP2	2.29239440284647e-08	0.259943607156677	0.244	0.281	0.000662960461303199	Proximal Tubule
VWA8	3.43071127997692e-08	0.25366439027311	0.558	0.6	0.000992161702169325	Proximal Tubule
PPP1R13B	5.11966680353246e-08	0.309661814318991	0.31	0.303	0.00148060763958159	Proximal Tubule
HIVEP1	5.22110667530512e-08	0.549628279538215	0.286	0.286	0.00150994405049824	Proximal Tubule
TRHDE	6.22044363572766e-08	0.269549297569879	0.41	0.41	0.00179895229945244	Proximal Tubule
EEF1A1	1.01421355303134e-07	0.470540290549071	0.269	0.265	0.00293310559536663	Proximal Tubule
RNMT	1.26060378136338e-07	0.287042942035965	0.296	0.341	0.00364566613570289	Proximal Tubule
SHTN1	1.27940561120014e-07	0.509798066305976	0.265	0.264	0.0037000410275908	Proximal Tubule
ADGRA3	1.87571808958908e-07	0.514324798231599	0.257	0.254	0.00542457671509163	Proximal Tubule
KIAA1958	2.9547505440838e-07	0.315955767080769	0.411	0.426	0.00854513857349036	Proximal Tubule
MGA	4.46900131444032e-07	0.271006264282411	0.252	0.288	0.0129243518013614	Proximal Tubule
ARID4A	4.89940389258216e-07	0.5633079069727	0.263	0.262	0.0141690760573476	Proximal Tubule
PIGL	4.95115328097172e-07	0.2893765762683	0.236	0.271	0.0143187352885702	Proximal Tubule
PPFIBP1	5.54179560333158e-07	0.394554697492099	0.426	0.45	0.0160268728848349	Proximal Tubule
CCNL1	5.93028695945814e-07	0.452301553202664	0.368	0.382	0.0171503898867529	Proximal Tubule
NPIPB5	1.69195467446026e-06	0.535023234715063	0.313	0.322	0.0489313291853907	Proximal Tubule
SLC25A13	3.22709205728558e-06	0.379604116463246	0.391	0.413	0.0933275022966989	Proximal Tubule
ZCCHC14	4.86769930801088e-06	0.263295080004665	0.237	0.269	0.140773863987675	Proximal Tubule
PHF3	5.37993434706057e-06	0.43714327383684	0.424	0.454	0.155587701316992	Proximal Tubule
CCDC14	6.3141535064141e-06	0.338371442648849	0.242	0.276	0.182605319405496	Proximal Tubule
NUMB	6.66564541708181e-06	0.317996447136836	0.464	0.507	0.192770465462006	Proximal Tubule
MDN1	8.13854595349321e-06	0.482746480621595	0.265	0.268	0.235366748975024	Proximal Tubule
ADD3	1.47949752310513e-05	0.398601180027758	0.403	0.425	0.427870683682004	Proximal Tubule
APPL2	1.93394468439618e-05	0.294002190670314	0.231	0.261	0.559296802727375	Proximal Tubule
KIF1B	2.36092699114912e-05	0.358203204868207	0.42	0.448	0.682780085840324	Proximal Tubule
BAZ1B	2.54397498714516e-05	0.315731418972188	0.314	0.359	0.735717566282381	Proximal Tubule
FBXL5	2.67927265198742e-05	0.525074839997602	0.311	0.323	0.774845650954763	Proximal Tubule
SON	2.88643330708089e-05	0.300814794120798	0.598	0.658	0.834756512407793	Proximal Tubule
ACO2	3.62419298707063e-05	0.468552826776345	0.292	0.297	1	Proximal Tubule
AP2A2	3.79334831821256e-05	0.386305160744072	0.386	0.409	1	Proximal Tubule
URGCP	4.71093029739601e-05	0.491642366707528	0.29	0.297	1	Proximal Tubule
SPG7	5.11003749812648e-05	0.337865688185785	0.287	0.326	1	Proximal Tubule
MT-ND5	0.00011055274179143	0.438372390744826	0.304	0.309	1	Proximal Tubule
NT5C2	0.000127299740191981	0.399331018042959	0.416	0.452	1	Proximal Tubule
ELP4	0.00013158103576915	0.337784059897729	0.396	0.415	1	Proximal Tubule
POM121	0.000140180448204921	0.284788099657539	0.224	0.251	1	Proximal Tubule
ZMAT1	0.000143544321382083	0.310233716317457	0.398	0.457	1	Proximal Tubule
USP40	0.000184324685425723	0.494633128034377	0.348	0.368	1	Proximal Tubule
MPHOSPH8	0.000187885993048042	0.393014131099784	0.367	0.384	1	Proximal Tubule
RAB1A	0.000216707555254083	0.284218722720334	0.367	0.42	1	Proximal Tubule
SUGP2	0.000228580788235857	0.472219892261669	0.273	0.28	1	Proximal Tubule

gene	p_val	avg_log2FC	pct.1	pct.2	p_val_adj	cluster
RPAP2	0.000239112858263787	0.324353879079832	0.311	0.353	1	Proximal Tubule
UPF2	0.000249314061271243	0.287740134397075	0.314	0.355	1	Proximal Tubule
PAQR5	0.000270206841853712	0.304030419986627	0.359	0.373	1	Proximal Tubule
GLUD1	0.000401263965073573	0.282171927744455	0.231	0.258	1	Proximal Tubule
HIPK3	0.00043750542943992	0.282547299775093	0.326	0.369	1	Proximal Tubule
ZC3H7A	0.000525506516400223	0.436328097581916	0.235	0.266	1	Proximal Tubule
IGF2BP2	0.000619487882522595	0.298075719558449	0.351	0.359	1	Proximal Tubule
AL050309.1	0.000626066524496713	0.268355019590565	0.423	0.441	1	Proximal Tubule
FAM185A	0.000848647796464771	0.359202872329213	0.231	0.258	1	Proximal Tubule
EFR3A	0.000862669111895121	0.298342707835769	0.315	0.354	1	Proximal Tubule
ACADVL	0.000919833967904819	0.51453571999694	0.272	0.28	1	Proximal Tubule
CHPT1	0.00129887837426138	0.319784248844147	0.25	0.25	1	Proximal Tubule
CEP290	0.00162518592613766	0.43073202320793	0.307	0.316	1	Proximal Tubule
TPD52	0.00480143545756647	0.32927592129796	0.313	0.349	1	Proximal Tubule
SEC31A	0.00582284947650213	0.427100278958481	0.38	0.414	1	Proximal Tubule
RAD50	0.00685459842342038	0.48269724296206	0.329	0.349	1	Proximal Tubule
MUC20	0.00741770420828887	0.371968832899853	0.272	0.28	1	Proximal Tubule
DERA	0.00796966925428046	0.301810793522655	0.235	0.26	1	Proximal Tubule
PIK3C2G	0	3.44200667065165	0.655	0.151	0	Distal Tubule / Collecting Duct
TEX41	0	2.83542885538407	0.669	0.223	0	Distal Tubule / Collecting Duct
SCN2A	0	2.27082514954473	0.624	0.224	0	Distal Tubule / Collecting Duct
TOX3	0	2.46135089950203	0.602	0.219	0	Distal Tubule / Collecting Duct
PRDM16	0	1.50785404187434	0.59	0.216	0	Distal Tubule / Collecting Duct
HSD11B2	0	2.25125910280277	0.588	0.219	0	Distal Tubule / Collecting Duct
TMEM52B	0	2.28538481247636	0.481	0.127	0	Distal Tubule / Collecting Duct
KLHL3	0	3.02689414881685	0.458	0.109	0	Distal Tubule / Collecting Duct
OSBPL3	0	1.3548425455728	0.633	0.284	0	Distal Tubule / Collecting Duct
KCNIP4	0	1.48801035961699	0.788	0.449	0	Distal Tubule / Collecting Duct
FREM1	0	1.443025491	0.624	0.285	0	Distal Tubule / Collecting Duct
EMX1	0	2.92492356291199	0.403	0.065	0	Distal Tubule / Collecting Duct
KAZN	0	1.24877998148577	0.807	0.471	0	Distal Tubule / Collecting Duct
**gene**	**p_val**	**avg_log2FC**	**pct.1**	**pct.2**	**p_val_adj**	**cluster**
AC019197.1	0	2.35685640498218	0.905	0.57	0	Distal Tubule / Collecting Duct
MYO1B	0	2.13112320979464	0.466	0.139	0	Distal Tubule / Collecting Duct
CACNB4	0	2.3602652277489	0.509	0.184	0	Distal Tubule / Collecting Duct
ICA1	0	1.87721131574359	0.552	0.228	0	Distal Tubule / Collecting Duct
RHOBTB3	0	1.05404737379359	0.621	0.3	0	Distal Tubule / Collecting Duct
CSGALNACT1	0	2.13780971807905	0.464	0.145	0	Distal Tubule / Collecting Duct
IVNS1ABP	0	1.78037822923616	0.575	0.256	0	Distal Tubule / Collecting Duct
WSB1	0	1.63148527486546	0.758	0.443	0	Distal Tubule / Collecting Duct
AC138305.1	0	1.23026709856673	0.645	0.336	0	Distal Tubule / Collecting Duct
ATP6V0A4	0	1.48030185340622	0.543	0.235	0	Distal Tubule / Collecting Duct

gene	p_val	avg_log2FC	pct.1	pct.2	p_val_adj	cluster
TRPM6	0	4.01551262396678	0.372	0.065	0	Distal Tubule / Collecting Duct
MECOM	0	1.72374357740902	0.941	0.635	0	Distal Tubule / Collecting Duct
EFNA5	0	1.0435926886836	0.664	0.362	0	Distal Tubule / Collecting Duct
GATA3	0	3.10162507805434	0.348	0.046	0	Distal Tubule / Collecting Duct
DCDC2	0	1.40233191830686	0.658	0.357	0	Distal Tubule / Collecting Duct
CLCNKB	0	1.36185379817888	0.546	0.246	0	Distal Tubule / Collecting Duct
HOXD3	0	1.51417865484614	0.53	0.23	0	Distal Tubule / Collecting Duct
ADAMTS16	0	2.05856440615949	0.456	0.156	0	Distal Tubule / Collecting Duct
MYO10	0	1.15084748839074	0.616	0.317	0	Distal Tubule / Collecting Duct
KCNQ1	0	1.37174762534536	0.561	0.264	0	Distal Tubule / Collecting Duct
FAM171A1	0	1.37358877518875	0.509	0.216	0	Distal Tubule / Collecting Duct
COL4A5	0	1.61184604602767	0.493	0.201	0	Distal Tubule / Collecting Duct
TBC1D9	0	1.94520613024328	0.464	0.173	0	Distal Tubule / Collecting Duct
SPIRE1	0	1.41434480400138	0.565	0.274	0	Distal Tubule / Collecting Duct
DPY19L2	0	1.52559084151971	0.528	0.239	0	Distal Tubule / Collecting Duct
SLC12A3	0	4.6756066138898	0.478	0.189	0	Distal Tubule / Collecting Duct
ACSL4	0	1.801274306	0.509	0.221	0	Distal Tubule / Collecting Duct
ASAP2	0	1.22692603225446	0.66	0.375	0	Distal Tubule / Collecting Duct
COBLL1	0	1.77264097259424	0.91	0.629	0	Distal Tubule / Collecting Duct
ADAMTS9-AS2	0	1.17677092679362	0.801	0.521	0	Distal Tubule / Collecting Duct
MYO3B	0	1.34728425154524	0.474	0.194	0	Distal Tubule / Collecting Duct
KSR2	0	2.67335850336024	0.374	0.095	0	Distal Tubule / Collecting Duct
SIM1	0	1.45535705085845	0.462	0.184	0	Distal Tubule / Collecting Duct
BLNK	0	1.75860071085828	0.421	0.143	0	Distal Tubule / Collecting Duct
GRIP1	0	1.65109274682145	0.536	0.26	0	Distal Tubule / Collecting Duct
WNK1	0	1.62005303049688	0.765	0.49	0	Distal Tubule / Collecting Duct
FMN1	0	2.19464135958018	0.463	0.189	0	Distal Tubule / Collecting Duct
TMEM72	0	1.34490090695807	0.455	0.183	0	Distal Tubule / Collecting Duct
MYO5A	0	1.52843427750679	0.475	0.203	0	Distal Tubule / Collecting Duct
NOS1AP	0	1.50473966405376	0.529	0.258	0	Distal Tubule / Collecting Duct
TPST1	0	1.17088630214178	0.558	0.288	0	Distal Tubule / Collecting Duct
C11orf80	0	1.66941581787163	0.46	0.19	0	Distal Tubule / Collecting Duct
AL355612.1	0	3.61499848121859	0.368	0.099	0	Distal Tubule / Collecting Duct
SCIN	0	1.69825815860066	0.444	0.176	0	Distal Tubule / Collecting Duct
GRHL2	0	1.47757136954358	0.422	0.155	0	Distal Tubule / Collecting Duct
B4GALNT3	0	2.62991717611628	0.347	0.08	0	Distal Tubule / Collecting Duct
CCSER1	0	1.10248855983019	0.934	0.668	0	Distal Tubule / Collecting Duct
TMPRSS2	0	1.65664325543886	0.418	0.152	0	Distal Tubule / Collecting Duct
ABTB2	0	1.80292129171672	0.596	0.33	0	Distal Tubule / Collecting Duct
BMPR1B	0	2.52125723819066	0.413	0.148	0	Distal Tubule / Collecting Duct
NEBL	0	0.533239034614816	0.625	0.36	0	Distal Tubule / Collecting Duct
OPHN1	0	1.36493223440601	0.553	0.289	0	Distal Tubule / Collecting Duct

gene	p_val	avg_log2FC	pct.1	pct.2	p_val_adj	cluster
ERBB4	0	1.01315568609724	0.911	0.647	0	Distal Tubule / Collecting Duct
NR3C2	0	1.21083317120575	0.855	0.591	0	Distal Tubule / Collecting Duct
AC011586.2	0	2.25982483361778	0.353	0.09	0	Distal Tubule / Collecting Duct
DACH1	0	0.590153885237104	0.572	0.309	0	Distal Tubule / Collecting Duct
MITF	0	1.30634793754092	0.623	0.361	0	Distal Tubule / Collecting Duct
NAALADL2	0	1.02712435478756	0.837	0.575	0	Distal Tubule / Collecting Duct
SKAP1	0	0.617302298606441	0.567	0.305	0	Distal Tubule / Collecting Duct
RGL3	0	1.05530107062477	0.531	0.27	0	Distal Tubule / Collecting Duct
FAAH2	0	1.10286941420873	0.547	0.287	0	Distal Tubule / Collecting Duct
RNPC3	0	1.90448090081795	0.745	0.485	0	Distal Tubule / Collecting Duct
KITLG	0	2.40684387251968	0.358	0.1	0	Distal Tubule / Collecting Duct
ME3	0	1.68811952900044	0.46	0.203	0	Distal Tubule / Collecting Duct
LHX1	0	2.69853928175312	0.321	0.064	0	Distal Tubule / Collecting Duct
SCNN1A	0	1.05487103424282	0.516	0.26	0	Distal Tubule / Collecting Duct
CADPS2	0	1.81990744068544	0.735	0.48	0	Distal Tubule / Collecting Duct
CA12	0	1.10574908694275	0.728	0.473	0	Distal Tubule / Collecting Duct
CASZ1	0	1.34714464642501	0.441	0.187	0	Distal Tubule / Collecting Duct
WDFY2	0	1.05063730949197	0.535	0.282	0	Distal Tubule / Collecting Duct
LINC01099	0	3.66514868283222	0.321	0.068	0	Distal Tubule / Collecting Duct
PWRN1	0	3.42680548638218	0.338	0.085	0	Distal Tubule / Collecting Duct
C4orf19	0	1.13664527052945	0.542	0.29	0	Distal Tubule / Collecting Duct
KCTD8	0	1.7415390777338	0.399	0.147	0	Distal Tubule / Collecting Duct
CNNM2	0	2.28637242922991	0.584	0.332	0	Distal Tubule / Collecting Duct
LINC01762	0	1.07368389405165	0.504	0.253	0	Distal Tubule / Collecting Duct
APCDD1L-DT	0	3.7386637168808	0.285	0.034	0	Distal Tubule / Collecting Duct
BACE2	0	1.31050395576524	0.488	0.237	0	Distal Tubule / Collecting Duct
ARHGAP8	0	1.01067913193833	0.52	0.269	0	Distal Tubule / Collecting Duct
ITGA2	0	1.76120124558794	0.442	0.193	0	Distal Tubule / Collecting Duct
FHOD3	0	1.17541884627865	0.743	0.496	0	Distal Tubule / Collecting Duct
GPRIN3	0	1.47391257032919	0.375	0.129	0	Distal Tubule / Collecting Duct
TMTC2	0	1.68456292197825	0.51	0.264	0	Distal Tubule / Collecting Duct
LIMCH1	0	1.02486866779088	0.699	0.453	0	Distal Tubule / Collecting Duct
HOXB3	0	1.298921793	0.427	0.184	0	Distal Tubule / Collecting Duct
ABCA5	0	1.38598549215188	0.589	0.347	0	Distal Tubule / Collecting Duct
AC040174.1	0	1.24291570477319	0.418	0.178	0	Distal Tubule / Collecting Duct
RAP1GAP2	0	1.25798615483294	0.415	0.175	0	Distal Tubule / Collecting Duct
SLC22A15	0	1.4113748191254	0.404	0.165	0	Distal Tubule / Collecting Duct
SCN7A	0	1.37451498210501	0.419	0.181	0	Distal Tubule / Collecting Duct
MFHAS1	0	1.08271785198071	0.515	0.278	0	Distal Tubule / Collecting Duct
SLC38A1	0	1.49859050872811	0.42	0.184	0	Distal Tubule / Collecting Duct
RHBG	0	1.32463741083621	0.34	0.105	0	Distal Tubule / Collecting Duct
PTGER3	0	0.418083422515694	0.639	0.404	0	Distal Tubule / Collecting Duct

gene	p_val	avg_log2FC	pct.1	pct.2	p_val_adj	cluster
WNK4	0	1.51359342217549	0.383	0.149	0	Distal Tubule / Collecting Duct
TRIM2	0	0.825927435200734	0.537	0.304	0	Distal Tubule / Collecting Duct
VAV3	0	1.10159675148359	0.599	0.366	0	Distal Tubule / Collecting Duct
USP24	0	1.12396103567357	0.607	0.375	0	Distal Tubule / Collecting Duct
FGF13	0	1.48006335578103	0.502	0.27	0	Distal Tubule / Collecting Duct
LAMB1	0	0.980751700319568	0.634	0.403	0	Distal Tubule / Collecting Duct
SAT1	0	1.12286612449359	0.592	0.361	0	Distal Tubule / Collecting Duct
ABCC5	0	1.10515769917493	0.444	0.215	0	Distal Tubule / Collecting Duct
RNF150	0	0.828652246625304	0.51	0.282	0	Distal Tubule / Collecting Duct
ACOT11	0	0.975931406060759	0.492	0.264	0	Distal Tubule / Collecting Duct
FAM49B	0	0.948439643468566	0.543	0.316	0	Distal Tubule / Collecting Duct
CLCNKA	0	1.41804407364366	0.387	0.16	0	Distal Tubule / Collecting Duct
LINC01606	0	0.70222682875718	0.457	0.23	0	Distal Tubule / Collecting Duct
RAD51B	0	1.13888023393968	0.651	0.425	0	Distal Tubule / Collecting Duct
ARHGEF3	0	0.614826190794551	0.548	0.324	0	Distal Tubule / Collecting Duct
NAV2	0	0.582586426131857	0.528	0.304	0	Distal Tubule / Collecting Duct
ITPR1	0	1.04851493491856	0.586	0.362	0	Distal Tubule / Collecting Duct
CRYBG1	0	1.3126015142408	0.407	0.183	0	Distal Tubule / Collecting Duct
IPCEF1	0	1.10493986711914	0.484	0.26	0	Distal Tubule / Collecting Duct
GRB14	0	0.953501027053562	0.54	0.317	0	Distal Tubule / Collecting Duct
TSC22D2	0	1.32566377427058	0.554	0.332	0	Distal Tubule / Collecting Duct
SGPP1	0	1.52297068069194	0.404	0.182	0	Distal Tubule / Collecting Duct
KCTD1	0	0.929316036909036	0.487	0.265	0	Distal Tubule / Collecting Duct
AGBL4	0	0.83992243469068	0.666	0.446	0	Distal Tubule / Collecting Duct
ADGRL2	0	0.914088199811939	0.536	0.317	0	Distal Tubule / Collecting Duct
XKR6	0	0.883884215658165	0.521	0.302	0	Distal Tubule / Collecting Duct
KCNJ16	0	0.964451459332435	0.732	0.513	0	Distal Tubule / Collecting Duct
MROH7	0	1.12092343646864	0.403	0.186	0	Distal Tubule / Collecting Duct
MSR1	0	1.73731489751788	0.32	0.104	0	Distal Tubule / Collecting Duct
DEFB1	0	1.4452066243615	0.456	0.241	0	Distal Tubule / Collecting Duct
EYA3	0	1.21420322992855	0.477	0.263	0	Distal Tubule / Collecting Duct
AC078980.1	0	3.26497658001377	0.268	0.055	0	Distal Tubule / Collecting Duct
GTF2IRD1	0	0.987968820643141	0.499	0.286	0	Distal Tubule / Collecting Duct
KNG1	0	1.57787260004499	0.373	0.161	0	Distal Tubule / Collecting Duct
MYO1E	0	0.898007920920703	0.555	0.344	0	Distal Tubule / Collecting Duct
ATP1B1	0	0.691658290715305	0.816	0.606	0	Distal Tubule / Collecting Duct
PTPN3	0	0.821154851194412	0.511	0.303	0	Distal Tubule / Collecting Duct
CCDC178	0	1.38473112854514	0.371	0.163	0	Distal Tubule / Collecting Duct
TMEM213	0	1.10367244661727	0.394	0.187	0	Distal Tubule / Collecting Duct
ATP1B3	0	1.95515905875135	0.339	0.133	0	Distal Tubule / Collecting Duct
RALGPS1	0	0.623784361458028	0.532	0.326	0	Distal Tubule / Collecting Duct
LINC02694	0	2.22033626923827	0.301	0.095	0	Distal Tubule / Collecting Duct

gene	p_val	avg_log2FC	pct.1	pct.2	p_val_adj	cluster
RALBP1	0	1.52659610545898	0.349	0.143	0	Distal Tubule / Collecting Duct
SUSD1	0	1.55369396614426	0.327	0.122	0	Distal Tubule / Collecting Duct
AK3	0	0.768215550946514	0.565	0.36	0	Distal Tubule / Collecting Duct
MID1	0	1.62980611862269	0.325	0.121	0	Distal Tubule / Collecting Duct
APBB2	0	0.990623740720189	0.695	0.491	0	Distal Tubule / Collecting Duct
CHRM3	0	0.476494891287501	0.338	0.135	0	Distal Tubule / Collecting Duct
FSTL4	0	2.41689033600551	0.274	0.071	0	Distal Tubule / Collecting Duct
BACH2	0	0.900121352211577	0.407	0.204	0	Distal Tubule / Collecting Duct
CLCN5	0	0.396813623378947	0.589	0.386	0	Distal Tubule / Collecting Duct
ZNF704	0	0.81831710858599	0.582	0.38	0	Distal Tubule / Collecting Duct
TFCP2L1	0	0.608908754141057	0.708	0.507	0	Distal Tubule / Collecting Duct
RBBP8	0	0.961991699587353	0.482	0.281	0	Distal Tubule / Collecting Duct
LHX1-DT	0	2.13001435662085	0.254	0.055	0	Distal Tubule / Collecting Duct
ERMP1	0	1.33861733950554	0.34	0.142	0	Distal Tubule / Collecting Duct
DOCK7	0	0.881861903545471	0.461	0.263	0	Distal Tubule / Collecting Duct
CARMIL1	0	0.769867052669121	0.519	0.321	0	Distal Tubule / Collecting Duct
PTBP3	0	0.959312811113244	0.49	0.292	0	Distal Tubule / Collecting Duct
SCML1	0	1.3963870595399	0.331	0.133	0	Distal Tubule / Collecting Duct
SLC8A1	0	3.27715733320707	0.678	0.482	0	Distal Tubule / Collecting Duct
CADM1	0	1.53589472759516	0.401	0.205	0	Distal Tubule / Collecting Duct
STK39	0	0.60464742055021	0.485	0.289	0	Distal Tubule / Collecting Duct
LRRFIP1	0	0.930326992602077	0.627	0.431	0	Distal Tubule / Collecting Duct
MLLT3	0	0.759338998053443	0.503	0.307	0	Distal Tubule / Collecting Duct
TMEM51	0	1.15564366612965	0.429	0.233	0	Distal Tubule / Collecting Duct
ADAMTS17	0	2.0603584870362	0.343	0.148	0	Distal Tubule / Collecting Duct
SPATS2	0	1.09013723610321	0.375	0.18	0	Distal Tubule / Collecting Duct
SLC16A7	0	0.881655475693655	0.476	0.281	0	Distal Tubule / Collecting Duct
DANT2	0	0.540015199491571	0.821	0.626	0	Distal Tubule / Collecting Duct
MAP4K3	0	0.807902291827173	0.633	0.439	0	Distal Tubule / Collecting Duct
HDAC4	0	1.10926302576123	0.45	0.256	0	Distal Tubule / Collecting Duct
CACNA2D3	0	0.563422016397842	0.506	0.312	0	Distal Tubule / Collecting Duct
ARHGEF38	0	1.05136879674613	0.372	0.178	0	Distal Tubule / Collecting Duct
EPB41L1	0	0.650754813028638	0.533	0.339	0	Distal Tubule / Collecting Duct
OGT	0	0.835619444491263	0.461	0.267	0	Distal Tubule / Collecting Duct
CNOT6L	0	1.06967454379721	0.42	0.226	0	Distal Tubule / Collecting Duct
LLGL2	0	0.611025304778761	0.572	0.378	0	Distal Tubule / Collecting Duct
GAREM1	0	1.1150814368179	0.565	0.371	0	Distal Tubule / Collecting Duct
EGF	0	1.24167295876308	0.546	0.353	0	Distal Tubule / Collecting Duct
TOX	0	1.72238096727888	0.425	0.232	0	Distal Tubule / Collecting Duct
MIR3936HG	0	0.58483726220072	0.601	0.409	0	Distal Tubule / Collecting Duct
SAMD12	0	0.623774112658228	0.669	0.478	0	Distal Tubule / Collecting Duct
RIMBP2	0	1.68643890015222	0.28	0.09	0	Distal Tubule / Collecting Duct

gene	p_val	avg_log2FC	pct.1	pct.2	p_val_adj	cluster
ITPKB	0	1.52792514306155	0.314	0.124	0	Distal Tubule / Collecting Duct
CDH16	0	0.958245303872185	0.439	0.249	0	Distal Tubule / Collecting Duct
NFE2L2	0	0.937643918041354	0.555	0.366	0	Distal Tubule / Collecting Duct
PPP2R3A	0	0.461740364150907	0.802	0.614	0	Distal Tubule / Collecting Duct
DAB1	0	0.832198281636475	0.472	0.286	0	Distal Tubule / Collecting Duct
LNX1	0	0.784453562638158	0.378	0.192	0	Distal Tubule / Collecting Duct
SREBF2	0	0.702884617310589	0.492	0.306	0	Distal Tubule / Collecting Duct
LRP1B	0	0.744074447440254	0.512	0.328	0	Distal Tubule / Collecting Duct
CDK6	0	1.03686658018241	0.426	0.242	0	Distal Tubule / Collecting Duct
CLMN	0	0.503213255276679	0.648	0.464	0	Distal Tubule / Collecting Duct
SLC25A29	0	1.28912046024702	0.376	0.192	0	Distal Tubule / Collecting Duct
ZNF208	0	1.44359860792842	0.322	0.138	0	Distal Tubule / Collecting Duct
MAL	0	1.15453103586356	0.369	0.186	0	Distal Tubule / Collecting Duct
TMEM117	0	0.395655510079209	0.597	0.414	0	Distal Tubule / Collecting Duct
LRCH1	0	0.84878152681455	0.619	0.436	0	Distal Tubule / Collecting Duct
SGPP2	0	0.870749235745875	0.365	0.183	0	Distal Tubule / Collecting Duct
ABCA10	0	1.40468059042778	0.343	0.163	0	Distal Tubule / Collecting Duct
MSI2	0	0.66841455844503	0.838	0.658	0	Distal Tubule / Collecting Duct
AHI1	0	0.584408443067299	0.605	0.426	0	Distal Tubule / Collecting Duct
FAM110B	0	1.47289715419516	0.317	0.138	0	Distal Tubule / Collecting Duct
KCNJ1	0	1.1653772999915	0.314	0.135	0	Distal Tubule / Collecting Duct
CYS1	0	1.10873029242264	0.35	0.171	0	Distal Tubule / Collecting Duct
LGR4	0	0.789215728072428	0.642	0.464	0	Distal Tubule / Collecting Duct
PPARGC1A	0	0.658753773238325	0.696	0.518	0	Distal Tubule / Collecting Duct
CTDSPL	0	0.596560299341363	0.533	0.356	0	Distal Tubule / Collecting Duct
ESRRG	0	0.865213952924792	0.946	0.769	0	Distal Tubule / Collecting Duct
PDE7B	0	0.724918936563812	0.572	0.395	0	Distal Tubule / Collecting Duct
TLK1	0	0.618346666754182	0.545	0.369	0	Distal Tubule / Collecting Duct
ANKRD28	0	0.562601788636683	0.548	0.372	0	Distal Tubule / Collecting Duct
SGSM1	0	1.45671759935616	0.278	0.102	0	Distal Tubule / Collecting Duct
SRGAP3	0	1.04113039911648	0.389	0.213	0	Distal Tubule / Collecting Duct
ANXA11	0	0.958898035897741	0.386	0.21	0	Distal Tubule / Collecting Duct
ATP1A1	0	0.608048639408892	0.681	0.506	0	Distal Tubule / Collecting Duct
SUPT3H	0	0.623892807359639	0.52	0.345	0	Distal Tubule / Collecting Duct
BICD1	0	0.461128097882746	0.43	0.255	0	Distal Tubule / Collecting Duct
DYNC2H1	0	0.716061916288627	0.579	0.404	0	Distal Tubule / Collecting Duct
TRAPPC9	0	0.578868061964286	0.68	0.506	0	Distal Tubule / Collecting Duct
YAP1	0	0.710074032080897	0.527	0.353	0	Distal Tubule / Collecting Duct
SOX5	0	1.03972507391053	0.412	0.238	0	Distal Tubule / Collecting Duct
FRAS1	0	0.946951356382757	0.716	0.542	0	Distal Tubule / Collecting Duct
PTPN13	0	0.708012801835513	0.634	0.461	0	Distal Tubule / Collecting Duct
ACTR3C	0	0.746363733359667	0.424	0.251	0	Distal Tubule / Collecting Duct

gene	p_val	avg_log2FC	pct.1	pct.2	p_val_adj	cluster
PRKAG2	0	1.11775039691305	0.6	0.427	0	Distal Tubule / Collecting Duct
RABGEF1	0	0.59224246647002	0.535	0.363	0	Distal Tubule / Collecting Duct
MPPED2	0	0.760565705613611	0.419	0.247	0	Distal Tubule / Collecting Duct
GBE1	0	0.678376394429503	0.522	0.351	0	Distal Tubule / Collecting Duct
CALB1	0	1.86377139045401	0.308	0.137	0	Distal Tubule / Collecting Duct
GPC5	0	0.52192050866753	0.594	0.423	0	Distal Tubule / Collecting Duct
MRTFA	0	0.578867496965324	0.523	0.353	0	Distal Tubule / Collecting Duct
KIAA1217	0	0.383140731862295	0.763	0.595	0	Distal Tubule / Collecting Duct
PIP5K1B	0	0.810421338271356	0.319	0.151	0	Distal Tubule / Collecting Duct
MTX2	0	0.707968327045631	0.434	0.266	0	Distal Tubule / Collecting Duct
CCDC171	0	0.668824188370331	0.424	0.256	0	Distal Tubule / Collecting Duct
TNFRSF19	0	1.66958904023693	0.254	0.087	0	Distal Tubule / Collecting Duct
PFKP	0	1.31127765460046	0.302	0.135	0	Distal Tubule / Collecting Duct
FNDC3B	0	0.715100242596796	0.742	0.576	0	Distal Tubule / Collecting Duct
ZNF98	0	0.929517058998414	0.32	0.154	0	Distal Tubule / Collecting Duct
KLHL13	0	0.644105514614689	0.496	0.33	0	Distal Tubule / Collecting Duct
TSC22D1	0	0.827307564973765	0.551	0.386	0	Distal Tubule / Collecting Duct
ATP8A1	0	1.01479649596164	0.336	0.171	0	Distal Tubule / Collecting Duct
CFAP221	0	0.853105314054924	0.363	0.198	0	Distal Tubule / Collecting Duct
UBE2H	0	0.616722894514046	0.627	0.463	0	Distal Tubule / Collecting Duct
NUP214	0	0.646404814280785	0.469	0.305	0	Distal Tubule / Collecting Duct
ARNT2	0	0.775969550315568	0.359	0.195	0	Distal Tubule / Collecting Duct
CLASP1	0	0.689546157908026	0.652	0.489	0	Distal Tubule / Collecting Duct
ARHGAP6	0	0.561925532859972	0.797	0.634	0	Distal Tubule / Collecting Duct
HIP1R	0	0.700790791010787	0.407	0.244	0	Distal Tubule / Collecting Duct
TP53BP1	0	0.648895070276103	0.422	0.259	0	Distal Tubule / Collecting Duct
MYO5C	0	1.18707343119052	0.3	0.137	0	Distal Tubule / Collecting Duct
PRDM16-DT	0	1.28930447435466	0.275	0.113	0	Distal Tubule / Collecting Duct
EXPH5	0	0.977706349702542	0.311	0.149	0	Distal Tubule / Collecting Duct
NRCAM	0	1.26267929886313	0.302	0.14	0	Distal Tubule / Collecting Duct
SORL1	0	0.850690018621698	0.307	0.146	0	Distal Tubule / Collecting Duct
KANK1	0	0.984751116360031	0.721	0.56	0	Distal Tubule / Collecting Duct
RPS6KA6	0	0.930054962263023	0.32	0.16	0	Distal Tubule / Collecting Duct
TMEM116	0	0.748077323034011	0.349	0.189	0	Distal Tubule / Collecting Duct
MAGI1	0	0.319901641315155	0.87	0.711	0	Distal Tubule / Collecting Duct
USP3	0	0.593643741063299	0.458	0.299	0	Distal Tubule / Collecting Duct
SLC38A11	0	1.74829176134768	0.253	0.094	0	Distal Tubule / Collecting Duct
SUSD4	0	1.18520114980239	0.282	0.123	0	Distal Tubule / Collecting Duct
ESRRB	0	0.501137159562639	0.351	0.192	0	Distal Tubule / Collecting Duct
ELOVL7	0	0.855862750762279	0.34	0.182	0	Distal Tubule / Collecting Duct
BICC1	0	0.622937132201627	0.903	0.745	0	Distal Tubule / Collecting Duct
ZNF10	0	0.855166569018565	0.337	0.179	0	Distal Tubule / Collecting Duct

gene	p_val	avg_log2FC	pct.1	pct.2	p_val_adj	cluster
CA10	0	0.980720600999444	0.35	0.192	0	Distal Tubule / Collecting Duct
CD2AP	0	0.571432550336816	0.56	0.403	0	Distal Tubule / Collecting Duct
ATXN1	0	0.607601647363692	0.697	0.54	0	Distal Tubule / Collecting Duct
TPD52L1	0	1.23912543604603	0.266	0.11	0	Distal Tubule / Collecting Duct
SH3RF1	0	0.823131052873999	0.612	0.456	0	Distal Tubule / Collecting Duct
ADK	0	0.471839372233177	0.683	0.528	0	Distal Tubule / Collecting Duct
TRAK1	0	0.624693739919502	0.523	0.369	0	Distal Tubule / Collecting Duct
VPS37B	0	0.9171383296707	0.357	0.203	0	Distal Tubule / Collecting Duct
MARK1	0	1.20344262449589	0.27	0.117	0	Distal Tubule / Collecting Duct
HIF1A	0	0.797013234937016	0.464	0.311	0	Distal Tubule / Collecting Duct
MTMR10	0	0.847126355448176	0.397	0.244	0	Distal Tubule / Collecting Duct
AC092535.4	0	1.21465776082297	0.294	0.141	0	Distal Tubule / Collecting Duct
ZNF385D	0	1.64432012880348	0.271	0.119	0	Distal Tubule / Collecting Duct
ADAM22	0	1.16088571369501	0.277	0.125	0	Distal Tubule / Collecting Duct
VAV2	0	0.973659508478099	0.394	0.242	0	Distal Tubule / Collecting Duct
TRPM7	0	1.71599626173301	0.644	0.493	0	Distal Tubule / Collecting Duct
RNF24	0	1.23192653801318	0.266	0.115	0	Distal Tubule / Collecting Duct
IL1RAPL2	0	2.20634083142918	0.258	0.107	0	Distal Tubule / Collecting Duct
CPEB4	0	1.13152418646153	0.617	0.466	0	Distal Tubule / Collecting Duct
BTBD9	0	0.520942651822257	0.722	0.572	0	Distal Tubule / Collecting Duct
GLIS3	0	1.11452221263568	0.776	0.626	0	Distal Tubule / Collecting Duct
SEPTIN9	0	0.799321225763281	0.447	0.297	0	Distal Tubule / Collecting Duct
MAL2	0	1.37028522692197	0.251	0.101	0	Distal Tubule / Collecting Duct
AC002460.2	0	0.961859037480716	0.368	0.219	0	Distal Tubule / Collecting Duct
LINC01138	0	0.886571975612945	0.335	0.187	0	Distal Tubule / Collecting Duct
SLC11A2	0	1.14595905578544	0.276	0.128	0	Distal Tubule / Collecting Duct
BTBD11	0	1.30784522050213	0.348	0.2	0	Distal Tubule / Collecting Duct
ASXL3	0	0.85763112871695	0.303	0.156	0	Distal Tubule / Collecting Duct
MRPS6	0	0.706535307035723	0.356	0.209	0	Distal Tubule / Collecting Duct
NFAT5	0	0.565836338762902	0.689	0.542	0	Distal Tubule / Collecting Duct
GLS	0	0.801132649879755	0.666	0.52	0	Distal Tubule / Collecting Duct
SHB	0	1.15679854227696	0.304	0.158	0	Distal Tubule / Collecting Duct
AIF1L	0	1.14365116396038	0.265	0.12	0	Distal Tubule / Collecting Duct
HK1	0	1.05493624911822	0.257	0.113	0	Distal Tubule / Collecting Duct
FBXL17	0	0.458372716482394	0.708	0.565	0	Distal Tubule / Collecting Duct
FAM27C	0	1.01880478158181	0.278	0.135	0	Distal Tubule / Collecting Duct
CDH1	0	0.964625874528498	0.299	0.158	0	Distal Tubule / Collecting Duct
SLC35B4	0	0.979622104582121	0.265	0.125	0	Distal Tubule / Collecting Duct
SLC45A4	0	1.2165926357628	0.337	0.197	0	Distal Tubule / Collecting Duct
DCUN1D1	0	0.883743083056526	0.311	0.171	0	Distal Tubule / Collecting Duct
LINC00472	0	0.841925826199476	0.89	0.754	0	Distal Tubule / Collecting Duct
ANKRD13A	0	1.03107870986078	0.27	0.136	0	Distal Tubule / Collecting Duct

gene	p_val	avg_log2FC	pct.1	pct.2	p_val_adj	cluster
PHTF2	0	0.660665817063156	0.283	0.149	0	Distal Tubule / Collecting Duct
SLC36A4	0	0.769427877571758	0.293	0.159	0	Distal Tubule / Collecting Duct
LRBA	0	0.538037626591995	0.827	0.695	0	Distal Tubule / Collecting Duct
SH3BP4	0	0.913673386207123	0.286	0.154	0	Distal Tubule / Collecting Duct
MACC1	0	0.935092439476321	0.296	0.164	0	Distal Tubule / Collecting Duct
STIM2	0	1.09542343142611	0.322	0.191	0	Distal Tubule / Collecting Duct
CHSY1	0	1.05189090476251	0.261	0.13	0	Distal Tubule / Collecting Duct
MED13L	0	0.529426589116633	0.741	0.611	0	Distal Tubule / Collecting Duct
PCCA	0	0.394146259462922	0.785	0.655	0	Distal Tubule / Collecting Duct
FOSL2	0	0.957732795567245	0.266	0.137	0	Distal Tubule / Collecting Duct
PIGQ	0	0.939839224266448	0.25	0.121	0	Distal Tubule / Collecting Duct
CTNNAL1	0	1.08165191975109	0.255	0.127	0	Distal Tubule / Collecting Duct
SIK3	0	0.439350672702448	0.754	0.626	0	Distal Tubule / Collecting Duct
SLC20A1	0	1.33558952102422	0.255	0.129	0	Distal Tubule / Collecting Duct
ARID1B	0	0.443767019630613	0.769	0.643	0	Distal Tubule / Collecting Duct
KL	0	1.17950619883855	0.279	0.154	0	Distal Tubule / Collecting Duct
PDE10A	0	1.7006661679812	0.593	0.476	0	Distal Tubule / Collecting Duct
MAML2	0	0.672671530568226	0.788	0.681	0	Distal Tubule / Collecting Duct
PKHD1	0	0.367760986990618	0.921	0.816	0	Distal Tubule / Collecting Duct
IMMP2L	0	0.502649410465295	0.861	0.762	0	Distal Tubule / Collecting Duct
PDE4D	0	1.1697842345478	0.966	0.924	0	Distal Tubule / Collecting Duct
MALAT1	0	0.400249937692307	1	1	0	Distal Tubule / Collecting Duct
PANTR1	2.34900741833281e-307	0.73141914395428	0.277	0.145	6.79332945381849e-303	Distal Tubule / Collecting Duct
CREM	1.07646210089634e-304	0.839167978175432	0.317	0.182	3.11312839579222e-300	Distal Tubule / Collecting Duct
PLCB4	4.02534561150212e-303	0.558592540069862	0.292	0.159	1.16412995084641e-298	Distal Tubule / Collecting Duct
MPC1	1.37084483836737e-301	0.536256117170565	0.464	0.298	3.96448327255844e-297	Distal Tubule / Collecting Duct
COL4A3	1.51457169559924e-301	0.552067793983898	0.519	0.37	4.38014134367299e-297	Distal Tubule / Collecting Duct
CMTM4	3.51251691078854e-301	0.567625107314145	0.443	0.284	1.01581989060005e-296	Distal Tubule / Collecting Duct
GPR39	3.70448855449308e-301	0.841369036901642	0.293	0.163	1.0713380899594e-296	Distal Tubule / Collecting Duct
PLCG2	9.17004889870119e-301	0.830329876036193	0.435	0.273	2.65197814150439e-296	Distal Tubule / Collecting Duct
EIF4G3	4.21739265662744e-300	0.46643578034714	0.636	0.484	1.21966995629666e-295	Distal Tubule / Collecting Duct
RTN3	1.00492467451698e-299	0.735897796357994	0.31	0.175	2.90624215870309e-295	Distal Tubule / Collecting Duct
STXBP4	3.73160989785391e-298	0.633650527816763	0.407	0.258	1.07918158245935e-293	Distal Tubule / Collecting Duct
TBX2	8.92114398226283e-298	0.881339746710117	0.254	0.132	2.57999483967041e-293	Distal Tubule / Collecting Duct
FLG-AS1	2.5815685388844e-297	0.704195066425723	0.306	0.171	7.46589621445369e-293	Distal Tubule / Collecting Duct
FLNB	2.50724409217162e-296	0.486944515840012	0.445	0.282	7.25094991456033e-292	Distal Tubule / Collecting Duct
LINC01182	2.02154312935782e-295	0.951661536154106	0.261	0.14	5.84630273010281e-291	Distal Tubule / Collecting Duct
MAN1A1	2.32287040351887e-294	0.620982600520431	0.488	0.339	6.71774120697658e-290	Distal Tubule / Collecting Duct
CASR	5.71750764351714e-292	0.654455713476772	0.412	0.267	1.65350321050516e-287	Distal Tubule / Collecting Duct
SPIN1	1.15997226337066e-291	0.493133950082608	0.514	0.351	3.35463978566795e-287	Distal Tubule / Collecting Duct
MUC1	3.25629926817832e-291	0.974858167176276	0.268	0.144	9.4172174835717e-287	Distal Tubule / Collecting Duct
CPEB2	1.20787050164755e-290	0.839313027292439	0.383	0.247	3.49316149076473e-286	Distal Tubule / Collecting Duct

gene	p_val	avg_log2FC	pct.1	pct.2	p_val_adj	cluster
SGMS2	1.67652548524003e-289	0.904941907677158	0.259	0.139	4.84851170331416e-285	Distal Tubule / Collecting Duct
TANC1	3.31974081929768e-289	0.32015288783456	0.486	0.314	9.60069044940889e-285	Distal Tubule / Collecting Duct
LINC00970	2.20945896842244e-287	0.739553773689224	0.434	0.289	6.38975533667769e-283	Distal Tubule / Collecting Duct
TBC1D4	6.47177409527077e-285	0.339165300214926	0.571	0.377	1.87163706835231e-280	Distal Tubule / Collecting Duct
DOCK1	9.82257918754673e-285	0.401545679601291	0.695	0.535	2.84068990103851e-280	Distal Tubule / Collecting Duct
MYH10	2.04894585047538e-284	0.602021076904129	0.409	0.262	5.92555139957481e-280	Distal Tubule / Collecting Duct
FAM107B	1.38950440787722e-281	0.717577256458763	0.432	0.285	4.01844674758092e-277	Distal Tubule / Collecting Duct
PRKCE	3.10331846641537e-280	0.405737593738984	0.635	0.476	8.97479700487325e-276	Distal Tubule / Collecting Duct
CACHD1	3.20938550522897e-280	0.730570183897304	0.324	0.19	9.28154288112219e-276	Distal Tubule / Collecting Duct
ATP9B	8.50856636278948e-280	0.467013135173359	0.618	0.462	2.46067739211872e-275	Distal Tubule / Collecting Duct
CTNND1	1.87753921606407e-276	0.384278520484778	0.724	0.581	5.42984341285729e-272	Distal Tubule / Collecting Duct
SNTG1	2.12927052142535e-275	2.56389007714524	0.258	0.156	6.15785034796212e-271	Distal Tubule / Collecting Duct
OBSCN	8.17615658602874e-273	0.715832866704856	0.287	0.161	2.36454448467951e-268	Distal Tubule / Collecting Duct
GAB1	1.27693718522322e-271	0.527742622754698	0.463	0.312	3.69290233966556e-267	Distal Tubule / Collecting Duct
CAAP1	2.71840567716328e-270	0.591846647386858	0.34	0.205	7.86162921835621e-266	Distal Tubule / Collecting Duct
WWTR1	1.61886619164841e-269	0.707734574117849	0.508	0.371	4.68176102624719e-265	Distal Tubule / Collecting Duct
PDE9A	4.34947319080169e-269	0.683838716468128	0.335	0.2	1.25786764677985e-264	Distal Tubule / Collecting Duct
MTURN	6.30538205364512e-269	0.719828697185566	0.259	0.138	1.82351648991417e-264	Distal Tubule / Collecting Duct
DTNB	1.25845193731497e-268	0.619576557143423	0.394	0.253	3.6394430027149e-264	Distal Tubule / Collecting Duct
ATRN	9.10325823474661e-268	0.534460051985457	0.462	0.311	2.63266228148872e-263	Distal Tubule / Collecting Duct
TMEM161B-AS1	3.2225477189362e-267	0.540192261852511	0.493	0.342	9.3196080031635e-263	Distal Tubule / Collecting Duct
ARL15	3.45657369854146e-266	1.10823123190934	0.678	0.664	9.99641113618191e-262	Distal Tubule / Collecting Duct
ZNF44	2.40885538947664e-264	0.477855663270327	0.459	0.305	6.96640978636643e-260	Distal Tubule / Collecting Duct
RTN4	2.14003320391206e-261	0.76619284863418	0.47	0.323	6.18897602571368e-257	Distal Tubule / Collecting Duct
TRHDE	5.35834051755642e-261	0.475555913803224	0.516	0.36	1.54963207767732e-256	Distal Tubule / Collecting Duct
MYO6	3.82502393193014e-260	0.430077493950002	0.712	0.58	1.1061969211142e-255	Distal Tubule / Collecting Duct
SH3BGRL	4.05617651809999e-260	0.62506877425607	0.342	0.208	1.17304624903452e-255	Distal Tubule / Collecting Duct
BCAR3	4.28561320397702e-260	0.74550620821692	0.318	0.191	1.23939933859015e-255	Distal Tubule / Collecting Duct
ACVR1B	2.80777158244317e-259	0.615242487693958	0.325	0.194	8.12007541642563e-255	Distal Tubule / Collecting Duct
OGDHL	1.54783981190208e-258	0.591831292770504	0.388	0.245	4.47635273602082e-254	Distal Tubule / Collecting Duct
ZFYVE16	3.23482891938254e-258	0.543921433850404	0.478	0.327	9.35512523485431e-254	Distal Tubule / Collecting Duct
IGFBP7	6.50700397074388e-258	0.59056959781659	0.6	0.457	1.88182554833913e-253	Distal Tubule / Collecting Duct
KIZ	6.85679986183902e-258	0.869254862850289	0.291	0.171	1.98298652004384e-253	Distal Tubule / Collecting Duct
SEMA6D	2.30951746115679e-257	0.763716562900052	0.276	0.157	6.67912449766544e-253	Distal Tubule / Collecting Duct
MTR	4.06217216511647e-255	0.403626573809986	0.677	0.531	1.17478019015168e-250	Distal Tubule / Collecting Duct
SSBP3	4.32598532527753e-255	0.543934181889781	0.394	0.253	1.25107495607026e-250	Distal Tubule / Collecting Duct
NNT	1.70294884939408e-254	0.570180849778661	0.431	0.289	4.92492807244769e-250	Distal Tubule / Collecting Duct
BRAF	7.24538000274571e-254	0.414970701991507	0.656	0.512	2.09536389679406e-249	Distal Tubule / Collecting Duct
CLINT1	3.29458703701175e-253	0.534010821074504	0.432	0.288	9.52794571103798e-249	Distal Tubule / Collecting Duct
TBC1D1	3.11665975360709e-252	0.409313196863737	0.596	0.444	9.01338000743169e-248	Distal Tubule / Collecting Duct
PPP1R9A	2.29446122155324e-251	0.441133289375351	0.588	0.437	6.63558185273198e-247	Distal Tubule / Collecting Duct
MINDY2	1.22978935781713e-249	0.58673294966216	0.369	0.235	3.55655082280713e-245	Distal Tubule / Collecting Duct

gene	p_val	avg_log2FC	pct.1	pct.2	p_val_adj	cluster
DENND1B	8.49880325963166e-249	0.460733813041426	0.454	0.308	2.45785390268547e-244	Distal Tubule / Collecting Duct
EXOC6B	1.12965694883034e-248	0.396253419220212	0.667	0.524	3.26696789601734e-244	Distal Tubule / Collecting Duct
CAB39	1.29044445601913e-248	0.391835943955797	0.48	0.328	3.73196536680733e-244	Distal Tubule / Collecting Duct
AF117829.1	1.99897974279487e-248	0.454955480739102	0.517	0.365	5.78104941616275e-244	Distal Tubule / Collecting Duct
CBLB	5.373538452382e-247	0.272788755921968	0.509	0.362	1.55402732042887e-242	Distal Tubule / Collecting Duct
HNF1B	1.25111334813274e-246	0.513960434936407	0.367	0.229	3.61821980279988e-242	Distal Tubule / Collecting Duct
PPP4R1	3.04360612474862e-246	0.660019646030764	0.287	0.168	8.80210891277302e-242	Distal Tubule / Collecting Duct
MYO1D	5.98434063786796e-244	0.29860816774988	0.449	0.289	1.73067131247141e-239	Distal Tubule / Collecting Duct
ZDHHC21	2.19646095388961e-243	0.521241301096173	0.4	0.264	6.35216507864876e-239	Distal Tubule / Collecting Duct
TANC2	4.8110410197852e-243	0.530185618737204	0.461	0.325	1.39135306292188e-238	Distal Tubule / Collecting Duct
ST6GAL1	8.75419692023033e-243	0.647621399567214	0.376	0.243	2.53171374933061e-238	Distal Tubule / Collecting Duct
STX18-AS1	3.11992473217666e-242	0.656795817572411	0.321	0.197	9.02282232545491e-238	Distal Tubule / Collecting Duct
RCOR1	5.74412912204682e-242	0.571984980728315	0.368	0.235	1.66120214209594e-237	Distal Tubule / Collecting Duct
RALGPS2	8.19571177528744e-242	0.361501316810587	0.48	0.33	2.37019984541313e-237	Distal Tubule / Collecting Duct
DHX15	8.75731918690387e-242	0.511428570098175	0.384	0.247	2.5326167088526e-237	Distal Tubule / Collecting Duct
PRKG1	1.12625404497798e-241	0.528221028281108	0.618	0.505	3.25712669807633e-237	Distal Tubule / Collecting Duct
RNF213-AS1	1.72307253300413e-241	0.538591196083181	0.462	0.314	4.98312576544795e-237	Distal Tubule / Collecting Duct
SOX6	4.9048173938366e-241	0.408623906572424	0.719	0.582	1.41847319029754e-236	Distal Tubule / Collecting Duct
MIR646HG	1.18611641938687e-239	1.33269928860977	0.294	0.189	3.43024868486684e-235	Distal Tubule / Collecting Duct
BABAM2	3.52158901776596e-239	0.410036410045524	0.717	0.588	1.01844354393792e-234	Distal Tubule / Collecting Duct
CCDC148	4.41048137740617e-239	0.431693956723046	0.497	0.344	1.27551121434587e-234	Distal Tubule / Collecting Duct
NUP88	6.76890542117069e-239	0.58523861862256	0.311	0.187	1.95756744780256e-234	Distal Tubule / Collecting Duct
ARFGEF1	1.36331802438869e-237	0.411739884501471	0.61	0.467	3.94271572653208e-233	Distal Tubule / Collecting Duct
STRBP	1.48544644001445e-237	0.427958218882275	0.449	0.302	4.2959111045218e-233	Distal Tubule / Collecting Duct
UNC13B	1.54863377759438e-237	0.454561069986442	0.482	0.336	4.47864888480295e-233	Distal Tubule / Collecting Duct
STAM2	3.07311960512445e-237	0.600346248860047	0.311	0.188	8.88746189801992e-233	Distal Tubule / Collecting Duct
BMERB1	4.75732035483095e-237	0.51301937620587	0.353	0.221	1.37581704661711e-232	Distal Tubule / Collecting Duct
MAPK8	1.61377197864902e-236	0.365452559511136	0.679	0.523	4.66702856225296e-232	Distal Tubule / Collecting Duct
CHCHD3	2.71501080166132e-236	0.464269241966863	0.558	0.418	7.85181123840453e-232	Distal Tubule / Collecting Duct
HECTD2	9.02705684813625e-236	0.647212897202868	0.274	0.158	2.610624840481e-231	Distal Tubule / Collecting Duct
PHACTR2	1.610815192639e-234	0.517139390785557	0.405	0.271	4.65847753711197e-230	Distal Tubule / Collecting Duct
SYTL2	8.69814656714738e-234	0.670862626267355	0.365	0.235	2.51550398721902e-229	Distal Tubule / Collecting Duct
MEF2A	1.61344213003459e-232	0.338863623172212	0.631	0.491	4.66607464006004e-228	Distal Tubule / Collecting Duct
PRKAA2	3.67033260550464e-232	0.521041173865786	0.438	0.298	1.06146018951194e-227	Distal Tubule / Collecting Duct
PRICKLE2	6.67794902105187e-232	0.480324461518182	0.536	0.39	1.9312628568882e-227	Distal Tubule / Collecting Duct
MFN1	9.30009444973227e-232	0.632511088781117	0.259	0.147	2.68958731486257e-227	Distal Tubule / Collecting Duct
SRSF5	6.21415623579371e-231	0.469795245391062	0.462	0.317	1.79713398339154e-226	Distal Tubule / Collecting Duct
KIF13B	1.42290971557133e-228	0.467571667951231	0.515	0.371	4.11505489743228e-224	Distal Tubule / Collecting Duct
ABCB7	2.03823622198877e-227	0.643691282004276	0.283	0.169	5.89457915399151e-223	Distal Tubule / Collecting Duct
STX18	1.04765492856002e-226	0.554056935559399	0.4	0.267	3.02981805339558e-222	Distal Tubule / Collecting Duct
FRY	2.69970031262167e-226	0.251071178393273	0.358	0.224	7.80753330410187e-222	Distal Tubule / Collecting Duct
ZDHHC14	2.96999402489989e-226	0.655350203322109	0.418	0.289	8.58922272001048e-222	Distal Tubule / Collecting Duct

gene	p_val	avg_log2FC	pct.1	pct.2	p_val_adj	cluster
PKM	1.87910350742536e-224	0.630352947672016	0.3	0.181	5.43436734347415e-220	Distal Tubule / Collecting Duct
GABARAPL1	1.71966746019662e-223	0.518271727625769	0.305	0.184	4.97327829488862e-219	Distal Tubule / Collecting Duct
MICU3	2.39180322887049e-223	0.634631114333529	0.268	0.156	6.91709493789346e-219	Distal Tubule / Collecting Duct
ETNK1	4.96683088407157e-223	0.477567989384744	0.418	0.283	1.4364074916735e-218	Distal Tubule / Collecting Duct
TRMT9B	2.41148903159811e-222	0.676282092915533	0.285	0.17	6.97402627938172e-218	Distal Tubule / Collecting Duct
NAP1L4	1.06627313201554e-220	0.437889937273297	0.466	0.325	3.08366189778894e-216	Distal Tubule / Collecting Duct
CPEB3	9.2429731251867e-219	0.272335745219124	0.571	0.406	2.67306782780399e-214	Distal Tubule / Collecting Duct
MFF	2.03579767523409e-218	0.619662911227708	0.271	0.16	5.88752687677698e-214	Distal Tubule / Collecting Duct
NLK	3.27600159402594e-218	0.483843072591537	0.492	0.36	9.47419660992303e-214	Distal Tubule / Collecting Duct
NPAS2	4.00329556347692e-217	0.503769851832212	0.267	0.153	1.15775307695752e-212	Distal Tubule / Collecting Duct
SLC35F5	2.15558601787704e-214	0.519081237985297	0.394	0.266	6.23395476370041e-210	Distal Tubule / Collecting Duct
ATP6V1H	5.40914251154749e-214	0.443203673833142	0.509	0.367	1.56432401433954e-209	Distal Tubule / Collecting Duct
RAI1	1.10277907445917e-213	0.714045865420619	0.256	0.15	3.18923708333593e-209	Distal Tubule / Collecting Duct
CDC42BPB	3.96168038567207e-213	0.498931391341905	0.435	0.302	1.14571796753636e-208	Distal Tubule / Collecting Duct
SLC25A12	9.00202828269056e-213	0.495607566787589	0.395	0.265	2.60338657935411e-208	Distal Tubule / Collecting Duct
TOM1L2	1.18476040790963e-212	0.465202106261649	0.461	0.324	3.42632709967466e-208	Distal Tubule / Collecting Duct
IQCK	1.49931481254665e-212	0.609378646731232	0.306	0.191	4.33601843788491e-208	Distal Tubule / Collecting Duct
DDX17	3.9319906392682e-212	0.308474900973355	0.807	0.683	1.13713169287636e-207	Distal Tubule / Collecting Duct
BCL2L1	3.55333592052355e-210	0.621582706044847	0.395	0.271	1.02762474821541e-205	Distal Tubule / Collecting Duct
HERC3	3.56531878163985e-210	0.400335419299851	0.405	0.27	1.03109019165024e-205	Distal Tubule / Collecting Duct
PISD	1.16886658657112e-209	0.601144975660935	0.323	0.204	3.38036216836368e-205	Distal Tubule / Collecting Duct
ITGB5	2.5752838045835e-209	0.613983669208654	0.342	0.222	7.44772076285549e-205	Distal Tubule / Collecting Duct
NR6A1	1.36395648793914e-208	0.435709817954636	0.49	0.351	3.94456216312e-204	Distal Tubule / Collecting Duct
STARD13	2.53254329369622e-208	0.613078397773599	0.495	0.373	7.32411520536947e-204	Distal Tubule / Collecting Duct
CDYL	3.35206188403881e-207	0.514853798569545	0.343	0.221	9.69416296864025e-203	Distal Tubule / Collecting Duct
TMEM164	6.9762714720526e-207	0.629031363853412	0.285	0.174	2.01753770971761e-202	Distal Tubule / Collecting Duct
SNX10	3.10888747170691e-206	0.674163051990344	0.323	0.209	8.99090256817637e-202	Distal Tubule / Collecting Duct
NR4A1	3.75568201905809e-206	0.461249719832286	0.276	0.165	1.0861432399116e-201	Distal Tubule / Collecting Duct
CLCN3	1.39023849764647e-205	0.665776116062059	0.315	0.2	4.0205697351936e-201	Distal Tubule / Collecting Duct
C20orf194	2.41203358949388e-204	0.401007946353853	0.661	0.527	6.9756011408163e-200	Distal Tubule / Collecting Duct
MIR4435-2HG	2.83855748363175e-204	0.83594398228655	0.261	0.157	8.20910824266303e-200	Distal Tubule / Collecting Duct
PREPL	1.67816419150437e-203	0.477396057489006	0.374	0.249	4.85325084183063e-199	Distal Tubule / Collecting Duct
RBFOX2	4.60149425135943e-203	0.324023097119179	0.537	0.392	1.33075213749315e-198	Distal Tubule / Collecting Duct
GRAMD1C	5.24110957533251e-203	0.489924955021046	0.318	0.199	1.51572888918616e-198	Distal Tubule / Collecting Duct
ANKRD10	5.99693686315891e-203	0.435303472173057	0.413	0.283	1.73431414082556e-198	Distal Tubule / Collecting Duct
SGMS1	1.22545727480623e-202	0.46996494220082	0.497	0.366	3.54402243873963e-198	Distal Tubule / Collecting Duct
PHLDB2	2.79647901603785e-202	0.384011218142939	0.602	0.464	8.08741731438148e-198	Distal Tubule / Collecting Duct
PKP4	1.10372336156144e-201	0.391951430597825	0.768	0.656	3.19196796163569e-197	Distal Tubule / Collecting Duct
TBL1X	3.43013405867192e-201	0.464634508410296	0.565	0.429	9.91994769767919e-197	Distal Tubule / Collecting Duct
MRPL1	1.05139199114483e-200	0.661513591702832	0.257	0.154	3.04062563839084e-196	Distal Tubule / Collecting Duct
FAM160A1	1.96325928808465e-200	0.357282486181386	0.571	0.423	5.6777458611408e-196	Distal Tubule / Collecting Duct
SLC25A27	3.87602431046162e-199	0.517623599111656	0.313	0.198	1.1209462305855e-194	Distal Tubule / Collecting Duct

gene	p_val	avg_log2FC	pct.1	pct.2	p_val_adj	cluster
RNF10	1.45164224975462e-198	0.572731065394945	0.34	0.222	4.19814938629038e-194	Distal Tubule / Collecting Duct
VEPH1	3.12049942699407e-198	0.715250273402756	0.348	0.233	9.02448434286686e-194	Distal Tubule / Collecting Duct
NRSN2-AS1	1.14237208592049e-197	0.613932930885345	0.318	0.203	3.30374007248207e-193	Distal Tubule / Collecting Duct
MCU	4.24383749200546e-197	0.579541077501501	0.419	0.298	1.22731780268798e-192	Distal Tubule / Collecting Duct
LINC01725	4.03973981933343e-195	0.440212284701537	0.479	0.345	1.16829275575123e-190	Distal Tubule / Collecting Duct
MOB1B	4.53105056794422e-195	0.372926015234499	0.408	0.282	1.31037982424947e-190	Distal Tubule / Collecting Duct
MTUS1	5.32510188103298e-195	0.346292759100001	0.443	0.309	1.54001946399474e-190	Distal Tubule / Collecting Duct
MAGED1	2.13242325930031e-194	0.496041455375593	0.392	0.268	6.16696806589649e-190	Distal Tubule / Collecting Duct
HOOK1	4.62778905773653e-193	0.477491771674063	0.268	0.161	1.33835659549741e-188	Distal Tubule / Collecting Duct
PDE1C	5.26100971024543e-193	0.517167639362093	0.268	0.163	1.52148400820298e-188	Distal Tubule / Collecting Duct
COL4A4	5.65338390338019e-193	0.460888738346933	0.439	0.313	1.63495862485755e-188	Distal Tubule / Collecting Duct
CLASP2	3.0888755777647e-192	0.38822499973235	0.507	0.375	8.93302817089552e-188	Distal Tubule / Collecting Duct
REV3L	2.27654750571759e-191	0.340957855584682	0.511	0.373	6.58377538653528e-187	Distal Tubule / Collecting Duct
IQGAP1	2.90613366136422e-190	0.38255728335191	0.385	0.259	8.40453854866534e-186	Distal Tubule / Collecting Duct
PCNX2	5.06350397422893e-190	0.408533084201584	0.363	0.24	1.46436534934701e-185	Distal Tubule / Collecting Duct
FRK	2.14483571352097e-189	0.433313445086668	0.419	0.291	6.20286488350264e-185	Distal Tubule / Collecting Duct
IDH2	2.50908948423044e-188	0.618042742814983	0.267	0.163	7.25628678839444e-184	Distal Tubule / Collecting Duct
UQCRC2	8.67405939670844e-188	0.507308589775426	0.322	0.208	2.50853797752808e-183	Distal Tubule / Collecting Duct
ARFGEF2	9.17903040472823e-188	0.393248279121367	0.471	0.337	2.6545755930474e-183	Distal Tubule / Collecting Duct
TSC1	9.84521860355823e-188	0.504440863663341	0.315	0.202	2.84723722014904e-183	Distal Tubule / Collecting Duct
USP25	4.46856037151421e-187	0.369170257248577	0.597	0.461	1.29230765944191e-182	Distal Tubule / Collecting Duct
DNM1L	2.77139067841994e-186	0.478659172454437	0.339	0.222	8.01486184199046e-182	Distal Tubule / Collecting Duct
RAB11FIP2	1.74861150936126e-185	0.508525665526109	0.306	0.197	5.05698448507278e-181	Distal Tubule / Collecting Duct
CBFA2T2	2.15897341548824e-185	0.365829777945336	0.511	0.378	6.243751117592e-181	Distal Tubule / Collecting Duct
ERBB2	2.65234506615543e-185	0.465689592421373	0.311	0.198	7.6705819313215e-181	Distal Tubule / Collecting Duct
POGZ	2.77988496821197e-185	0.352791045228005	0.565	0.425	8.03942732806901e-181	Distal Tubule / Collecting Duct
FYB2	2.70517834406973e-184	0.551469743658568	0.264	0.162	7.82337577104967e-180	Distal Tubule / Collecting Duct
WDR91	4.07641517378508e-184	0.57174555031655	0.264	0.161	1.17889926825864e-179	Distal Tubule / Collecting Duct
ATP2C1	6.61596938508447e-184	0.415452071694534	0.36	0.239	1.91333834616643e-179	Distal Tubule / Collecting Duct
RIC1	6.74594075008421e-184	0.401334503718462	0.383	0.262	1.95092606492435e-179	Distal Tubule / Collecting Duct
REEP3	7.82192645010868e-184	0.501035260534886	0.285	0.179	2.26210112937143e-179	Distal Tubule / Collecting Duct
MAP3K21	7.89279065203202e-184	0.672703640572112	0.322	0.214	2.28259505656766e-179	Distal Tubule / Collecting Duct
ARHGEF18	1.27781236133084e-183	0.569917801159941	0.287	0.181	3.6954333489688e-179	Distal Tubule / Collecting Duct
ANK3	1.43793638752171e-183	0.314451843432741	0.845	0.753	4.15851203271278e-179	Distal Tubule / Collecting Duct
OIP5-AS1	6.39573754338723e-183	0.463243805736523	0.336	0.22	1.84964729754759e-178	Distal Tubule / Collecting Duct
GALNT11	8.83582983983592e-182	0.522665542422911	0.362	0.246	2.55532198968055e-177	Distal Tubule / Collecting Duct
LINC01266	1.67697123615463e-181	0.648051016532562	0.288	0.185	4.84980081495918e-177	Distal Tubule / Collecting Duct
COG5	3.26805550732298e-181	0.388081771577917	0.509	0.376	9.45121652717805e-177	Distal Tubule / Collecting Duct
ADAMTS9	1.42586637681706e-180	0.380923520383184	0.423	0.301	4.12360556175494e-176	Distal Tubule / Collecting Duct
PTK2	1.87227250301082e-180	0.28475660889751	0.737	0.621	5.41461207870729e-176	Distal Tubule / Collecting Duct
ARHGAP5	7.04306528114087e-178	0.34593349269416	0.537	0.406	2.03685447930594e-173	Distal Tubule / Collecting Duct
ADAM10	7.35919690689977e-178	0.354515745690094	0.47	0.341	2.12827974547541e-173	Distal Tubule / Collecting Duct

gene	p_val	avg_log2FC	pct.1	pct.2	p_val_adj	cluster
ARIH1	2.58069836778093e-177	0.325398657808425	0.566	0.438	7.46337967962245e-173	Distal Tubule / Collecting Duct
MAP4K3-DT	1.92151063848075e-175	0.426960395349119	0.485	0.359	5.55700876648632e-171	Distal Tubule / Collecting Duct
CDK8	1.19468941178347e-174	0.371053478336571	0.468	0.339	3.45504177887779e-170	Distal Tubule / Collecting Duct
MRTFB	1.3247098764976e-174	0.32643649548553	0.496	0.362	3.83106096283106e-170	Distal Tubule / Collecting Duct
CD24	8.54590621394876e-174	0.609116855406349	0.251	0.154	2.47147607707398e-169	Distal Tubule / Collecting Duct
KANSL3	1.97996394075807e-173	0.480648735271589	0.318	0.209	5.72605571667233e-169	Distal Tubule / Collecting Duct
RBPMS	6.62976301420655e-173	0.514644927807056	0.756	0.66	1.91732746370853e-168	Distal Tubule / Collecting Duct
SNX9	7.77481257707174e-172	0.410645775432405	0.436	0.315	2.24847579728915e-167	Distal Tubule / Collecting Duct
RALGAPA1	9.70303064234764e-171	0.311648077371255	0.607	0.475	2.80611646176694e-166	Distal Tubule / Collecting Duct
KCNJ3	3.01292776958796e-170	0.473661848978062	0.409	0.293	8.71338710964838e-166	Distal Tubule / Collecting Duct
DYRK1A	3.51824535485172e-170	0.297144852478418	0.598	0.467	1.01747655662312e-165	Distal Tubule / Collecting Duct
TMCC1	6.09811255156641e-170	0.329154204892097	0.58	0.45	1.763574149913e-165	Distal Tubule / Collecting Duct
VCL	2.04802004572404e-168	0.737803874407716	0.331	0.225	5.92287397223392e-164	Distal Tubule / Collecting Duct
RERE	1.03048656335455e-167	0.268623431769625	0.808	0.704	2.98016714122136e-163	Distal Tubule / Collecting Duct
SSH1	1.9071784073181e-167	0.516180746884524	0.254	0.157	5.51555995396393e-163	Distal Tubule / Collecting Duct
ACSL3	3.87589976361453e-167	0.499640676914582	0.315	0.209	1.12091021163732e-162	Distal Tubule / Collecting Duct
MACROD2	4.38359532950261e-167	0.37016378632819	0.64	0.525	1.26773576929215e-162	Distal Tubule / Collecting Duct
ATXN7	1.8745847044468e-166	0.451273164631065	0.365	0.252	5.42129896526016e-162	Distal Tubule / Collecting Duct
ATP5F1C	3.52400760899401e-166	0.420783458085084	0.307	0.2	1.01914300052107e-161	Distal Tubule / Collecting Duct
DNAH14	5.93777630067593e-166	0.326568159441941	0.599	0.463	1.71720490615548e-161	Distal Tubule / Collecting Duct
GSPT1	1.066024115352e-165	0.454293825458784	0.297	0.192	3.08294174159799e-161	Distal Tubule / Collecting Duct
USP12	2.39801391108663e-165	0.426839110687522	0.31	0.204	6.93505623086253e-161	Distal Tubule / Collecting Duct
ACVR2A	4.38535986059351e-165	0.501906026225049	0.269	0.171	1.26824607168364e-160	Distal Tubule / Collecting Duct
SPPL2A	4.46814109550836e-165	0.556946348766576	0.335	0.23	1.29218640482102e-160	Distal Tubule / Collecting Duct
DIAPH1	2.41404229831494e-164	0.365587477927297	0.437	0.315	6.98141032672681e-160	Distal Tubule / Collecting Duct
SMIM8	6.53840251891218e-164	0.496420707858142	0.263	0.166	1.8909060084694e-159	Distal Tubule / Collecting Duct
VAPA	4.00280531504685e-162	0.389963895424049	0.398	0.282	1.15761129711155e-157	Distal Tubule / Collecting Duct
RAB3IP	7.44829616327942e-162	0.291409573093486	0.496	0.363	2.15404725042041e-157	Distal Tubule / Collecting Duct
MAP7	2.19680116955426e-161	0.272537393077834	0.533	0.394	6.35314898235093e-157	Distal Tubule / Collecting Duct
PWWP2A	2.31781900185378e-161	0.4772668533529	0.261	0.163	6.70313255336114e-157	Distal Tubule / Collecting Duct
NEDD4L	9.41448183728774e-161	0.317974550517977	0.772	0.655	2.72266814734361e-156	Distal Tubule / Collecting Duct
MAP3K1	2.5674386594039e-160	0.282252736662566	0.36	0.245	7.42503260299607e-156	Distal Tubule / Collecting Duct
PLS3	1.1929742434119e-159	0.550539727581789	0.3	0.199	3.45008151194722e-155	Distal Tubule / Collecting Duct
SNX24	1.33263701151927e-159	0.41553645104022	0.391	0.277	3.85398623731374e-155	Distal Tubule / Collecting Duct
HS6ST2	1.4488416107265e-159	0.448188979886877	0.27	0.173	4.19004993822103e-155	Distal Tubule / Collecting Duct
HIVEP2	2.56170290815252e-159	0.37207593022534	0.452	0.335	7.40844481037708e-155	Distal Tubule / Collecting Duct
SENP6	2.21352174830586e-158	0.283898187655857	0.613	0.48	6.40150489610055e-154	Distal Tubule / Collecting Duct
ZFAND3	3.55867744646464e-158	0.278902014075482	0.77	0.681	1.02916951751757e-153	Distal Tubule / Collecting Duct
DNAJB6	4.70728047263593e-158	0.418668188997385	0.291	0.19	1.36134551268631e-153	Distal Tubule / Collecting Duct
RASA2	1.090158416319e-157	0.285732312208213	0.367	0.253	3.15273813999454e-153	Distal Tubule / Collecting Duct
MRPS5	1.38121957008099e-157	0.448019277622734	0.325	0.218	3.99448699667422e-153	Distal Tubule / Collecting Duct
TOP2B	5.81068865846541e-157	0.330008062411935	0.342	0.23	1.6804511600282e-152	Distal Tubule / Collecting Duct

gene	p_val	avg_log2FC	pct.1	pct.2	p_val_adj	cluster
HLCS	9.24007440743367e-157	0.453823454835537	0.375	0.265	2.67222951862982e-152	Distal Tubule / Collecting Duct
MEGF9	1.57356373860791e-156	0.513637082586632	0.3	0.198	4.55074633205407e-152	Distal Tubule / Collecting Duct
ARHGAP24	5.27149144477197e-156	0.32805725484085	0.622	0.502	1.52451532582805e-151	Distal Tubule / Collecting Duct
PLXNB1	1.26633560269026e-155	0.468698617300935	0.356	0.246	3.66224256298022e-151	Distal Tubule / Collecting Duct
LINC02241	2.10968495714415e-155	0.474999990409232	0.258	0.164	6.10120889606087e-151	Distal Tubule / Collecting Duct
BMPR1A	2.24692130436703e-155	0.347240527354972	0.398	0.281	6.49809641222945e-151	Distal Tubule / Collecting Duct
TBC1D32	2.73355198890702e-155	0.434865503173119	0.334	0.227	7.9054323519191e-151	Distal Tubule / Collecting Duct
UBAC2	1.15550654124857e-154	0.287260872450963	0.474	0.351	3.34172491729086e-150	Distal Tubule / Collecting Duct
PPP1CB	1.33601084836788e-154	0.480640716518466	0.324	0.22	3.86374337347991e-150	Distal Tubule / Collecting Duct
SPAG16	1.12964565085093e-153	0.531588711517456	0.441	0.335	3.26693522226088e-149	Distal Tubule / Collecting Duct
NPEPPS	1.40168857005735e-153	0.2939353218876	0.558	0.43	4.05368334460585e-149	Distal Tubule / Collecting Duct
NFYC	3.01656720710976e-153	0.387119939692793	0.399	0.285	8.72391236296144e-149	Distal Tubule / Collecting Duct
MAP4	1.62843873723838e-152	0.32130225157592	0.607	0.493	4.70944482809341e-148	Distal Tubule / Collecting Duct
TVP23C	6.01262757637066e-152	0.365084841395751	0.341	0.232	1.7388518950864e-147	Distal Tubule / Collecting Duct
MYOF	8.28759856769372e-152	0.503335170993121	0.281	0.182	2.39677350577702e-147	Distal Tubule / Collecting Duct
TACC2	1.99502835423969e-151	0.460609846885962	0.452	0.339	5.76962200046119e-147	Distal Tubule / Collecting Duct
TOM1L1	2.54411596449647e-151	0.336437091824316	0.385	0.272	7.3575833693238e-147	Distal Tubule / Collecting Duct
CCDC50	4.33379954642128e-151	0.366563474100648	0.387	0.272	1.25333482882503e-146	Distal Tubule / Collecting Duct
ATAD1	6.04723631089449e-151	0.404832186205519	0.283	0.184	1.74886074111069e-146	Distal Tubule / Collecting Duct
NUDT4	6.48872350073195e-150	0.354717693181914	0.308	0.204	1.87653883641168e-145	Distal Tubule / Collecting Duct
FOCAD	9.06463171661828e-150	0.348689710064673	0.399	0.286	2.62149149244601e-145	Distal Tubule / Collecting Duct
SLMAP	1.72561510251998e-149	0.294017551690771	0.499	0.376	4.99047887648778e-145	Distal Tubule / Collecting Duct
TMEM245	3.00112792238062e-149	0.364335360776133	0.449	0.334	8.67926195152475e-145	Distal Tubule / Collecting Duct
CYSTM1	5.55099141471078e-149	0.471214993363587	0.359	0.252	1.60534671713436e-144	Distal Tubule / Collecting Duct
AAGAB	1.48768939497786e-148	0.44695028795428	0.26	0.167	4.30239773027597e-144	Distal Tubule / Collecting Duct
CTBP2	1.94461397165766e-148	0.322993994817199	0.559	0.434	5.62382360603395e-144	Distal Tubule / Collecting Duct
RAB28	2.78837726384268e-148	0.329410116183928	0.476	0.354	8.06398704703302e-144	Distal Tubule / Collecting Duct
ZNF516	3.43848316569091e-148	0.412850822795048	0.359	0.253	9.9440933151781e-144	Distal Tubule / Collecting Duct
NDUFA10	3.41891968925179e-147	0.39769547621162	0.342	0.235	9.88751574131618e-143	Distal Tubule / Collecting Duct
PAQR5	6.11570262408235e-147	0.380784443368524	0.451	0.33	1.76866119888461e-142	Distal Tubule / Collecting Duct
SLX4IP	8.31396103519481e-147	0.369320704420126	0.369	0.26	2.40439753137834e-142	Distal Tubule / Collecting Duct
MAP2K4	9.93528858154653e-147	0.315491364180012	0.429	0.314	2.87328545778326e-142	Distal Tubule / Collecting Duct
FBXW11	1.01677475042933e-146	0.2869211550005	0.493	0.369	2.94051257824163e-142	Distal Tubule / Collecting Duct
STAT3	1.96622518664465e-146	0.315409707486446	0.504	0.382	5.68632323977634e-142	Distal Tubule / Collecting Duct
XPO4	2.19248143773018e-146	0.34423415432353	0.327	0.222	6.34065631791568e-142	Distal Tubule / Collecting Duct
REV1	2.99240266870146e-146	0.300820341810895	0.499	0.376	8.65402851788463e-142	Distal Tubule / Collecting Duct
IP6K1	1.17297580185554e-145	0.433199327219321	0.285	0.188	3.39224601896621e-141	Distal Tubule / Collecting Duct
ZNF493	2.02717872163381e-145	0.408772944459245	0.297	0.197	5.86260086296497e-141	Distal Tubule / Collecting Duct
PITPNM2	2.8410671179807e-145	0.43720321208233	0.326	0.224	8.21636610520017e-141	Distal Tubule / Collecting Duct
OXSR1	4.03808867805336e-145	0.378811443616447	0.348	0.242	1.16781524569303e-140	Distal Tubule / Collecting Duct
OLA1	8.48067930148961e-145	0.338162767157924	0.384	0.273	2.45261245399079e-140	Distal Tubule / Collecting Duct
ITGAV	1.34479470387095e-144	0.303113623962738	0.401	0.284	3.8891462835948e-140	Distal Tubule / Collecting Duct

gene	p_val	avg_log2FC	pct.1	pct.2	p_val_adj	cluster
RAPGEF6	1.51794007882934e-144	0.30185015803499	0.402	0.287	4.38988270797447e-140	Distal Tubule / Collecting Duct
ARHGAP12	1.77908010497918e-144	0.312810621011095	0.391	0.277	5.14509966359977e-140	Distal Tubule / Collecting Duct
PDS5B	4.24262794830977e-144	0.324258392663974	0.414	0.301	1.22696800265118e-139	Distal Tubule / Collecting Duct
ZNF618	7.4840635774268e-144	0.369963624529992	0.422	0.309	2.16439118659183e-139	Distal Tubule / Collecting Duct
SMAD3	1.38174437080704e-143	0.730762696633256	0.427	0.323	3.99600472037395e-139	Distal Tubule / Collecting Duct
LCORL	1.97633435278021e-143	0.353871374570004	0.405	0.293	5.71555894824038e-139	Distal Tubule / Collecting Duct
RLF	2.18161698083863e-143	0.364379153606632	0.331	0.227	6.30923630858533e-139	Distal Tubule / Collecting Duct
SLC44A3	2.73158141408824e-143	0.454611942985269	0.289	0.192	7.89973344954318e-139	Distal Tubule / Collecting Duct
ATP6V1A	1.05847182393431e-141	0.365374588673863	0.39	0.278	3.06110051481802e-137	Distal Tubule / Collecting Duct
FASTKD1	1.00624377538176e-140	0.456334742020524	0.257	0.167	2.91005699840405e-136	Distal Tubule / Collecting Duct
TRIM24	2.66110159047153e-140	0.480811980690548	0.272	0.18	7.69590579964367e-136	Distal Tubule / Collecting Duct
IFT80	7.58874586842317e-140	0.444104807256067	0.255	0.166	2.19466530514798e-135	Distal Tubule / Collecting Duct
SMAD1	1.12815261714283e-139	0.492828193767657	0.252	0.164	3.26261736877707e-135	Distal Tubule / Collecting Duct
ZMYM2	1.2744651626609e-139	0.264748110782892	0.56	0.437	3.68575325041531e-135	Distal Tubule / Collecting Duct
GPI	1.56019385788497e-139	0.400644537088689	0.356	0.25	4.51208063700332e-135	Distal Tubule / Collecting Duct
DAPK1	3.24177128304309e-139	0.486100075401674	0.463	0.354	9.37520255056061e-135	Distal Tubule / Collecting Duct
DOP1A	6.17822571297409e-139	0.340269627453365	0.413	0.302	1.78674287619211e-134	Distal Tubule / Collecting Duct
RYBP	1.11427603532744e-138	0.458141131460762	0.272	0.18	3.22248629416696e-134	Distal Tubule / Collecting Duct
RNF149	2.23670218403416e-138	0.327974903407726	0.345	0.24	6.46854271622679e-134	Distal Tubule / Collecting Duct
ATP6V0D2	4.04827674291879e-138	0.392552997288487	0.28	0.185	1.17076163405211e-133	Distal Tubule / Collecting Duct
AL008633.1	8.66166997996674e-138	0.507108038911349	0.31	0.215	2.50495495820638e-133	Distal Tubule / Collecting Duct
ZMYND11	1.34742662221993e-137	0.301391988848919	0.475	0.357	3.89675779146004e-133	Distal Tubule / Collecting Duct
CBFB	1.89391513238637e-137	0.37627492268676	0.332	0.23	5.47720256286138e-133	Distal Tubule / Collecting Duct
AGL	3.2704306682928e-137	0.356219653499906	0.263	0.171	9.45808549270279e-133	Distal Tubule / Collecting Duct
LATS2	4.24847062528253e-137	0.40792914331581	0.291	0.196	1.22865770483171e-132	Distal Tubule / Collecting Duct
FER	8.13831444108108e-137	0.289109676814993	0.524	0.407	2.35360053636065e-132	Distal Tubule / Collecting Duct
PDZRN3	1.14888077794043e-136	0.358713311586571	0.419	0.308	3.32256320980373e-132	Distal Tubule / Collecting Duct
UBE2K	3.19103670569018e-136	0.258664542169693	0.516	0.395	9.22847815285599e-132	Distal Tubule / Collecting Duct
GALNT7	8.42215836147369e-136	0.468410006443559	0.264	0.174	2.43568819813819e-131	Distal Tubule / Collecting Duct
HGSNAT	3.55122320524191e-135	0.405393437181118	0.296	0.201	1.02701375095596e-130	Distal Tubule / Collecting Duct
CBR4	5.54062350849403e-135	0.28943892313683	0.509	0.39	1.60234831865647e-130	Distal Tubule / Collecting Duct
STRN	6.19048482306464e-135	0.369780612960461	0.347	0.243	1.79028821083029e-130	Distal Tubule / Collecting Duct
FGFR2	6.34116325546808e-135	0.494186680021211	0.266	0.177	1.83386441348137e-130	Distal Tubule / Collecting Duct
AFG3L2	1.47924604338918e-134	0.410157808642991	0.306	0.209	4.2779795574815e-130	Distal Tubule / Collecting Duct
ATP6V0E1	1.72275373291649e-134	0.464221517015028	0.279	0.187	4.98220379559449e-130	Distal Tubule / Collecting Duct
PAWR	9.77960034168359e-134	0.269783270123524	0.419	0.306	2.8282604188149e-129	Distal Tubule / Collecting Duct
ZNF544	1.28113369171496e-133	0.379876239566783	0.323	0.223	3.70503863643966e-129	Distal Tubule / Collecting Duct
PDHX	1.29652896475878e-133	0.406494558606349	0.284	0.19	3.74956176608239e-129	Distal Tubule / Collecting Duct
FAM13B	1.33578880727368e-133	0.279488420970348	0.419	0.305	3.86310123063547e-129	Distal Tubule / Collecting Duct
MTERF1	2.05820616012172e-133	0.455215741582437	0.335	0.237	5.95233221507202e-129	Distal Tubule / Collecting Duct
MBOAT2	1.5174639221283e-132	0.614629992163022	0.287	0.196	4.38850566279503e-128	Distal Tubule / Collecting Duct
SMC5	1.54818737238477e-132	0.290722425494102	0.393	0.283	4.47735788093677e-128	Distal Tubule / Collecting Duct

gene	p_val	avg_log2FC	pct.1	pct.2	p_val_adj	cluster
ATG5	1.72913078439337e-132	0.346201713213536	0.316	0.217	5.00064622846563e-128	Distal Tubule / Collecting Duct
UMAD1	1.82119692213683e-132	0.293476705457288	0.5	0.384	5.2669014988197e-128	Distal Tubule / Collecting Duct
AGO2	1.96999621928227e-132	0.44050936889622	0.295	0.201	5.69722906616432e-128	Distal Tubule / Collecting Duct
SMAP1	2.27411651702582e-132	0.319008275817377	0.453	0.341	6.57674496723868e-128	Distal Tubule / Collecting Duct
COG7	3.0503895389121e-132	0.490771846581324	0.25	0.163	8.82172654653379e-128	Distal Tubule / Collecting Duct
PPP6R3	4.97964710269368e-132	0.257152388793474	0.607	0.492	1.44011394209901e-127	Distal Tubule / Collecting Duct
CSAD	8.2099039470752e-132	0.409634862422974	0.34	0.238	2.37430422149415e-127	Distal Tubule / Collecting Duct
ZNF141	2.03317223819132e-131	0.519614578853871	0.256	0.169	5.8799341128493e-127	Distal Tubule / Collecting Duct
ZFYVE9	2.21037276627695e-131	0.316881762722521	0.354	0.249	6.39239804007295e-127	Distal Tubule / Collecting Duct
NDUFS4	3.34518337010171e-131	0.321786965724674	0.329	0.228	9.67427030633413e-127	Distal Tubule / Collecting Duct
SRSF10	1.67747884838112e-130	0.336414253080604	0.377	0.27	4.8512688295182e-126	Distal Tubule / Collecting Duct
CEP95	1.74640616694087e-130	0.27880644455595	0.376	0.269	5.05060663479301e-126	Distal Tubule / Collecting Duct
TCAIM	3.63518466410282e-130	0.367557384249857	0.409	0.302	1.05129540485853e-125	Distal Tubule / Collecting Duct
NISCH	4.73266541931443e-129	0.463863226731832	0.262	0.174	1.36868683926573e-124	Distal Tubule / Collecting Duct
LINC02256	8.49827940955503e-129	0.325556594099093	0.337	0.236	2.45770240524331e-124	Distal Tubule / Collecting Duct
ATL2	9.56331471141429e-128	0.467672933241484	0.356	0.258	2.76571061454101e-123	Distal Tubule / Collecting Duct
JARID2	1.22162322361385e-127	0.31343968438195	0.447	0.338	3.53293436269126e-123	Distal Tubule / Collecting Duct
CMC1	1.59446112077907e-127	0.367840131259677	0.3	0.206	4.61118156129307e-123	Distal Tubule / Collecting Duct
SNX6	1.92221096110596e-127	0.290856315849035	0.351	0.247	5.55903409951844e-123	Distal Tubule / Collecting Duct
SLC10A7	2.97261957888276e-127	0.382436838630329	0.272	0.183	8.59681582212896e-123	Distal Tubule / Collecting Duct
CMC2	4.48372390315591e-127	0.38867328447325	0.25	0.164	1.29669295279269e-122	Distal Tubule / Collecting Duct
RANBP9	1.25193276319347e-126	0.302110477752847	0.317	0.22	3.62058955115552e-122	Distal Tubule / Collecting Duct
MYSM1	2.40916502912646e-126	0.327528406909669	0.326	0.228	6.96730526423371e-122	Distal Tubule / Collecting Duct
AZIN1-AS1	3.07137738617199e-126	0.379707370350766	0.353	0.253	8.8824234008094e-122	Distal Tubule / Collecting Duct
ANO10	3.46685411161325e-126	0.333054037770125	0.459	0.349	1.00261420907855e-121	Distal Tubule / Collecting Duct
ZRANB2	4.41229068178902e-126	0.255578415283572	0.59	0.457	1.27603446517339e-121	Distal Tubule / Collecting Duct
YWHAZ	4.45957398612042e-126	0.326732830070628	0.37	0.267	1.28970879678603e-121	Distal Tubule / Collecting Duct
MCCC1	1.32268511219142e-125	0.367855758298747	0.34	0.243	3.82520534445758e-121	Distal Tubule / Collecting Duct
PTPN14	1.78451790492278e-125	0.286367823862926	0.455	0.341	5.16082578103667e-121	Distal Tubule / Collecting Duct
PARD6B	3.53518180127933e-125	0.429041217174373	0.311	0.218	1.02237457692998e-120	Distal Tubule / Collecting Duct
TOP1	5.71709484616061e-125	0.305535030761836	0.415	0.308	1.65338382950965e-120	Distal Tubule / Collecting Duct
BTRC	7.1887217792694e-125	0.287211575889923	0.402	0.295	2.07897833856471e-120	Distal Tubule / Collecting Duct
ZMIZ1	7.30768787128527e-125	0.322868885371452	0.443	0.332	2.1133833323757e-120	Distal Tubule / Collecting Duct
METTL25	1.01093512037003e-124	0.374707368078729	0.301	0.208	2.92362436811012e-120	Distal Tubule / Collecting Duct
CAMKMT	1.42596726964026e-123	0.263214862487422	0.59	0.477	4.12389734379963e-119	Distal Tubule / Collecting Duct
OXNAD1	1.55017694551689e-123	0.297081981077621	0.332	0.235	4.48311172643484e-119	Distal Tubule / Collecting Duct
AP2B1	1.70528148750823e-123	0.276206509731674	0.355	0.253	4.93167406187381e-119	Distal Tubule / Collecting Duct
ATF6	5.94528796263246e-123	0.262340460205472	0.409	0.302	1.71937727879331e-118	Distal Tubule / Collecting Duct
FBXL2	2.61694646982347e-122	0.440839976793454	0.274	0.187	7.56820919072948e-118	Distal Tubule / Collecting Duct
TFCP2	5.21568139900455e-122	0.281240496253251	0.421	0.313	1.50837506059212e-117	Distal Tubule / Collecting Duct
CRTC1	6.09339536225965e-122	0.468893637413852	0.277	0.188	1.76220993876549e-117	Distal Tubule / Collecting Duct
MAP4K5	4.00811430411701e-121	0.334240643361296	0.351	0.254	1.15914665675064e-116	Distal Tubule / Collecting Duct

gene	p_val	avg_log2FC	pct.1	pct.2	p_val_adj	cluster
FERMT2	3.95524173652609e-120	0.344942873955784	0.426	0.324	1.14385591020335e-115	Distal Tubule / Collecting Duct
KDM7A	2.5656444170405e-119	0.362974136014823	0.258	0.173	7.41984365408112e-115	Distal Tubule / Collecting Duct
SF1	3.2730405959802e-119	0.363412193741242	0.322	0.228	9.46563340357474e-115	Distal Tubule / Collecting Duct
RALGAPB	4.4244530064853e-119	0.285111370217575	0.346	0.247	1.27955180947555e-114	Distal Tubule / Collecting Duct
WDR27	6.95879040058927e-119	0.329497174200071	0.352	0.255	2.01248218385042e-114	Distal Tubule / Collecting Duct
LINC00271	1.05483263054234e-118	0.358368441860261	0.399	0.299	3.05057596752845e-114	Distal Tubule / Collecting Duct
TTN-AS1	2.89167381581572e-118	0.364546328195012	0.397	0.297	8.36272067533907e-114	Distal Tubule / Collecting Duct
ABCD3	3.01913818700028e-118	0.276319207574013	0.412	0.306	8.73134763680481e-114	Distal Tubule / Collecting Duct
STK33	5.25754838926136e-118	0.327943483970984	0.464	0.359	1.52048299417438e-113	Distal Tubule / Collecting Duct
DICER1	6.19860306137431e-118	0.264082768906491	0.448	0.336	1.79263600534945e-113	Distal Tubule / Collecting Duct
SMC5-AS1	2.8505068402909e-117	0.356276942986623	0.347	0.249	8.24366578212127e-113	Distal Tubule / Collecting Duct
COX10	6.10901544636438e-117	0.380954197906215	0.282	0.194	1.76672726708858e-112	Distal Tubule / Collecting Duct
CFAP70	7.25137447847195e-117	0.284535969275739	0.342	0.244	2.09709749917409e-112	Distal Tubule / Collecting Duct
CAPN2	8.6167236928419e-117	0.333689572251998	0.329	0.231	2.49195649196988e-112	Distal Tubule / Collecting Duct
DDAH1	2.0838121751213e-116	0.305861787156014	0.44	0.334	6.02638481045079e-112	Distal Tubule / Collecting Duct
MRPS25	2.10187021705213e-116	0.30372138208266	0.281	0.192	6.07860866771475e-112	Distal Tubule / Collecting Duct
NUP98	2.6052740109712e-116	0.252196499531454	0.354	0.255	7.5344524397287e-112	Distal Tubule / Collecting Duct
CDC73	3.74558812618157e-116	0.262297307559424	0.37	0.269	1.08322408609171e-111	Distal Tubule / Collecting Duct
EMX2OS	5.80995620913239e-116	0.398089675253143	0.33	0.236	1.68023933568109e-111	Distal Tubule / Collecting Duct
TMEM260	7.3606998511485e-116	0.310498343642608	0.309	0.217	2.12871439695215e-111	Distal Tubule / Collecting Duct
PXK	9.48935644735648e-116	0.293579098448164	0.263	0.176	2.74432188457549e-111	Distal Tubule / Collecting Duct
PLEKHH2	2.01786782859389e-115	0.399898296287837	0.258	0.174	5.83567376029354e-111	Distal Tubule / Collecting Duct
SPATA6	3.27289603594687e-115	0.253345152323077	0.383	0.28	9.46521533595834e-111	Distal Tubule / Collecting Duct
DNASE1	3.51580917656343e-115	0.468716023168431	0.31	0.22	1.01677201386214e-110	Distal Tubule / Collecting Duct
ATG10	4.2516801631424e-115	0.322279066785767	0.38	0.281	1.22958590318078e-110	Distal Tubule / Collecting Duct
C11orf49	8.59967918002624e-115	0.261365136962119	0.45	0.344	2.48702721886359e-110	Distal Tubule / Collecting Duct
AGFG1	2.68962549232483e-114	0.269009753669199	0.36	0.262	7.77839692380342e-110	Distal Tubule / Collecting Duct
CRTC3	1.83618781315982e-113	0.263410182720353	0.299	0.209	5.31025515565819e-109	Distal Tubule / Collecting Duct
CCDC84	4.26421214635353e-113	0.278224442602706	0.275	0.188	1.23321015272544e-108	Distal Tubule / Collecting Duct
CMSS1	4.6397784742336e-113	0.405282276126417	0.312	0.223	1.34182393474836e-108	Distal Tubule / Collecting Duct
SETBP1	1.1489276928039e-112	0.268117138029456	0.729	0.622	3.32269888758889e-108	Distal Tubule / Collecting Duct
UTY	3.77777189495518e-112	0.358572322083965	0.323	0.232	1.09253163202104e-107	Distal Tubule / Collecting Duct
RBM41	4.85386597151247e-112	0.389061417792569	0.291	0.203	1.40373803896141e-107	Distal Tubule / Collecting Duct
UBE2W	7.71516861155467e-112	0.253152226149942	0.368	0.271	2.23122676246161e-107	Distal Tubule / Collecting Duct
UBL3	1.19973663620732e-111	0.319864126121532	0.36	0.263	3.46963835191158e-107	Distal Tubule / Collecting Duct
DUSP1	1.69048391311549e-111	0.329035438597046	0.254	0.173	4.88887947673001e-107	Distal Tubule / Collecting Duct
ZNF532	1.79043822354165e-111	0.357200642475481	0.294	0.206	5.17794734248245e-107	Distal Tubule / Collecting Duct
UBE2E1	2.01938389764012e-110	0.261164851219064	0.369	0.271	5.84005823197521e-106	Distal Tubule / Collecting Duct
LNPEP	2.81541175450317e-110	0.295963034212435	0.33	0.236	8.14217079402318e-106	Distal Tubule / Collecting Duct
PIAS2	4.39939137120307e-110	0.260533631923539	0.376	0.277	1.27230398455193e-105	Distal Tubule / Collecting Duct
TNPO1	7.95532609372569e-110	0.289801883505479	0.364	0.268	2.30068030630547e-105	Distal Tubule / Collecting Duct
SDHA	8.1941839010523e-110	0.331798526022582	0.288	0.2	2.36975798418433e-105	Distal Tubule / Collecting Duct

gene	p_val	avg_log2FC	pct.1	pct.2	p_val_adj	cluster
GCFC2	1.69265071563892e-109	0.333251864479279	0.267	0.183	4.89514586962776e-105	Distal Tubule / Collecting Duct
RAB6A	5.85593844237622e-109	0.279225066788926	0.381	0.285	1.6935373975352e-104	Distal Tubule / Collecting Duct
AL589935.1	5.96335983673896e-109	0.299921779031259	0.366	0.269	1.72460366478491e-104	Distal Tubule / Collecting Duct
AP1G1	7.44583313477236e-109	0.269156034209893	0.374	0.277	2.15333494257617e-104	Distal Tubule / Collecting Duct
PCGF5	9.1083013836659e-109	0.27742282679707	0.392	0.293	2.63412076015618e-104	Distal Tubule / Collecting Duct
CAPS2	1.05361188187195e-108	0.392367023830299	0.259	0.177	3.04704556237369e-104	Distal Tubule / Collecting Duct
KIAA1958	1.06230813911014e-108	0.256373230058079	0.496	0.386	3.07219513830651e-104	Distal Tubule / Collecting Duct
GPCPD1	2.29371911330922e-108	0.265700764143205	0.312	0.222	6.63343567569027e-104	Distal Tubule / Collecting Duct
VPS45	4.01590089768664e-108	0.278469544220315	0.305	0.215	1.16139853961098e-103	Distal Tubule / Collecting Duct
MAN2A1	6.23654878388425e-108	0.332552558005186	0.42	0.318	1.80360990829933e-103	Distal Tubule / Collecting Duct
CRYBG3	8.97542130454788e-108	0.375272184496729	0.286	0.2	2.59569184127525e-103	Distal Tubule / Collecting Duct
UBA6	1.19048468920267e-107	0.320857258914277	0.254	0.172	3.44288172117412e-103	Distal Tubule / Collecting Duct
GNAS	1.94523731005828e-107	0.265911014277477	0.349	0.253	5.62562630068854e-103	Distal Tubule / Collecting Duct
MROH1	4.24792665710931e-107	0.360682066775382	0.28	0.195	1.22850038923601e-102	Distal Tubule / Collecting Duct
NPTN	9.29825670354053e-107	0.300843121923759	0.281	0.196	2.68905583866392e-102	Distal Tubule / Collecting Duct
SMAD2	2.40890343922627e-106	0.293639133908154	0.412	0.313	6.96654874624238e-102	Distal Tubule / Collecting Duct
FNDC3A	7.00279172335994e-106	0.570787411330452	0.575	0.49	2.02520736639569e-101	Distal Tubule / Collecting Duct
ARFIP1	7.59922757829267e-106	0.250320406638877	0.375	0.277	2.19769661564224e-101	Distal Tubule / Collecting Duct
TMCO4	1.07814150825329e-105	0.360239814118234	0.266	0.184	3.11798524186852e-101	Distal Tubule / Collecting Duct
SMARCA2	1.71199205310898e-105	0.609587909474387	0.412	0.322	4.95108101759116e-101	Distal Tubule / Collecting Duct
MIPOL1	3.00342885046697e-105	0.293289088939425	0.373	0.278	8.68591623555047e-101	Distal Tubule / Collecting Duct
FAM168B	3.52722625155071e-105	0.336471652366887	0.26	0.179	1.02007383194846e-100	Distal Tubule / Collecting Duct
ZDHHC20	4.46856059951517e-105	0.267680648069544	0.334	0.242	1.29230772537979e-100	Distal Tubule / Collecting Duct
CACNA1D	1.50231772099261e-104	0.741558783237977	0.407	0.337	4.34470284911063e-100	Distal Tubule / Collecting Duct
ORC4	1.39925014533529e-103	0.287856923238885	0.301	0.214	4.04663142030964e-99	Distal Tubule / Collecting Duct
ERICH1	1.91141012656278e-103	0.333255309081308	0.441	0.34	5.52779808601956e-99	Distal Tubule / Collecting Duct
IBTK	2.01482304759745e-103	0.271060598534583	0.342	0.25	5.82686825365183e-99	Distal Tubule / Collecting Duct
TPP2	2.87958880819106e-103	0.281296039926147	0.32	0.231	8.32777083328855e-99	Distal Tubule / Collecting Duct
XPO6	4.74948666853314e-103	0.299613570942872	0.252	0.172	1.37355154453979e-98	Distal Tubule / Collecting Duct
ESYT2	7.82689121843041e-103	0.405348179700362	0.395	0.302	2.26353694037007e-98	Distal Tubule / Collecting Duct
INPP5A	7.72287697062278e-102	0.269691212299366	0.298	0.21	2.23345601990411e-97	Distal Tubule / Collecting Duct
PACRG	1.97743005791287e-101	0.254764178112075	0.39	0.291	5.71872772748401e-97	Distal Tubule / Collecting Duct
NDFIP1	6.7277151884081e-101	0.25740262955751	0.285	0.2	1.94565523248762e-96	Distal Tubule / Collecting Duct
SNRK	7.19623869447784e-101	0.2795450090937	0.286	0.203	2.08115223044299e-96	Distal Tubule / Collecting Duct
CEP57	1.04132283687908e-100	0.273483554899108	0.302	0.215	3.01150564425429e-96	Distal Tubule / Collecting Duct
VRK2	1.6105321173744e-100	0.333049749272126	0.271	0.191	4.65765888344677e-96	Distal Tubule / Collecting Duct
RAB3GAP2	3.15780541398891e-100	0.253733749058701	0.395	0.297	9.13237325725591e-96	Distal Tubule / Collecting Duct
ILF3	4.5282704561664e-100	0.317551601098295	0.314	0.226	1.30957581592332e-95	Distal Tubule / Collecting Duct
USP22	1.0299322079142e-99	0.286523354607162	0.302	0.216	2.97856394528785e-95	Distal Tubule / Collecting Duct
DAAM1	8.49611944929574e-99	0.289873584021891	0.348	0.257	2.45707774473633e-94	Distal Tubule / Collecting Duct
SEPTIN11	3.6284290871454e-98	0.319368797990204	0.341	0.253	1.04934169200245e-93	Distal Tubule / Collecting Duct
GOT1	6.48162624944234e-98	0.27388127723625	0.329	0.237	1.87448631133872e-93	Distal Tubule / Collecting Duct

gene	p_val	avg_log2FC	pct.1	pct.2	p_val_adj	cluster
MPRIP	8.10394239149205e-98	0.283648477040092	0.369	0.276	2.3436601396195e-93	Distal Tubule / Collecting Duct
NDRG1	1.57255124421674e-97	0.450993150923659	0.445	0.352	4.5478181982748e-93	Distal Tubule / Collecting Duct
DSTN	2.91434585408702e-97	0.356825721454885	0.257	0.18	8.42828821001967e-93	Distal Tubule / Collecting Duct
STXBP5	7.84874172888504e-97	0.261509520582671	0.387	0.292	2.26985610799356e-92	Distal Tubule / Collecting Duct
MTF2	1.00578226961487e-96	0.25000138227761	0.312	0.225	2.90872232372619e-92	Distal Tubule / Collecting Duct
SLC44A1	9.75764019821178e-95	0.503850773094584	0.291	0.213	2.82190954532285e-90	Distal Tubule / Collecting Duct
EIPR1	3.78704510436996e-93	0.390604710727972	0.274	0.196	1.09521344418379e-88	Distal Tubule / Collecting Duct
HMG20A	2.72690545785319e-92	0.331953417804218	0.25	0.175	7.88621058411142e-88	Distal Tubule / Collecting Duct
PTPN2	4.87612591337696e-92	0.281171467867976	0.299	0.217	1.41017561414862e-87	Distal Tubule / Collecting Duct
TTC7A	1.77522663970108e-90	0.274173979942461	0.262	0.185	5.13395544201554e-86	Distal Tubule / Collecting Duct
AFF1	1.61137178122588e-89	0.263961736032641	0.686	0.602	4.66008719130524e-85	Distal Tubule / Collecting Duct
LINC01578	1.23887099541782e-88	0.258537566930042	0.303	0.222	3.58281491874833e-84	Distal Tubule / Collecting Duct
PAPPA	2.00814871735733e-88	1.03564467888081	0.277	0.217	5.80756609059741e-84	Distal Tubule / Collecting Duct
ATF7IP2	1.12767067175254e-86	0.322215631568433	0.265	0.19	3.26122358270833e-82	Distal Tubule / Collecting Duct
LTN1	1.29692016910407e-84	0.251530570522494	0.259	0.184	3.75069312904896e-80	Distal Tubule / Collecting Duct
SPP1	2.1972574701309e-83	0.823958004180361	0.315	0.246	6.35446860361857e-79	Distal Tubule / Collecting Duct
ZNF248	1.55463230250137e-82	0.250311250986088	0.303	0.225	4.49599661883395e-78	Distal Tubule / Collecting Duct
ERC1	3.17461705039131e-82	0.315670313917926	0.631	0.547	9.18099250973166e-78	Distal Tubule / Collecting Duct
RAD54L2	6.99105959304789e-82	0.286782115478451	0.281	0.205	2.02181443430945e-77	Distal Tubule / Collecting Duct
IFRD1	8.02958736491913e-82	0.472564409092258	0.272	0.201	2.32215666593461e-77	Distal Tubule / Collecting Duct
FAM117B	1.59366134931864e-81	0.323160347704527	0.251	0.181	4.6088686222295e-77	Distal Tubule / Collecting Duct
DPH6-DT	1.7168571119493e-76	0.33283334366873	0.397	0.318	4.96515076775738e-72	Distal Tubule / Collecting Duct
ZNF717	2.30453994716218e-74	0.257832958798211	0.267	0.196	6.66472952719301e-70	Distal Tubule / Collecting Duct
ZRANB2-AS2	1.15389848667813e-73	0.300384934641196	0.297	0.225	3.33707442347316e-69	Distal Tubule / Collecting Duct
PPP2R5A	1.59609971815562e-73	0.273816108244259	0.311	0.234	4.61592038490604e-69	Distal Tubule / Collecting Duct
THSD7A	2.46715985663265e-73	0.306446873529168	0.659	0.583	7.13502630538162e-69	Distal Tubule / Collecting Duct
MYO19	5.53517395641115e-73	0.29119060891237	0.256	0.188	1.6007723081941e-68	Distal Tubule / Collecting Duct
CECR2	2.54189313395959e-71	0.362229289938575	0.284	0.215	7.35115494341115e-67	Distal Tubule / Collecting Duct
MAGI3	4.38775615332013e-69	0.390458447580935	0.615	0.52	1.26893907954018e-64	Distal Tubule / Collecting Duct
ARID5B	5.03974660277846e-69	0.359958403804645	0.485	0.411	1.45749471752353e-64	Distal Tubule / Collecting Duct
NUP160	1.71547179673815e-68	0.251947184275062	0.251	0.185	4.96114443616674e-64	Distal Tubule / Collecting Duct
RANBP2	3.07231785155643e-68	0.454046849858987	0.378	0.295	8.8851432267012e-64	Distal Tubule / Collecting Duct
NELL1	1.23638365874597e-66	0.521082942809464	0.298	0.237	3.57562154109333e-62	Distal Tubule / Collecting Duct
SESTD1	3.24174814943555e-63	0.327950296816784	0.302	0.236	9.37513564816761e-59	Distal Tubule / Collecting Duct
NDEL1	4.62054618252229e-62	0.25552736637128	0.268	0.202	1.33626195598545e-57	Distal Tubule / Collecting Duct
TMEM120B	1.75870469821042e-61	0.264152264296399	0.27	0.204	5.08617398722454e-57	Distal Tubule / Collecting Duct
ACTB	1.47372454791377e-60	0.455812028732638	0.319	0.256	4.26201139256662e-56	Distal Tubule / Collecting Duct
FREM2	1.37364197918147e-57	0.289137347966219	0.304	0.238	3.9725726037928e-53	Distal Tubule / Collecting Duct
AC092683.1	6.56410723273495e-49	0.308369264944439	0.312	0.251	1.89833981170695e-44	Distal Tubule / Collecting Duct
GCNT2	3.16418328283429e-30	0.301589775499921	0.408	0.359	9.15081805395678e-26	Distal Tubule / Collecting Duct
FRMD3	1.57664645431964e-21	0.356400975112175	0.393	0.358	4.55966154589239e-17	Distal Tubule / Collecting Duct
UMOD	0	4.11417464280646	0.898	0.129	0	Loop of Henle

gene	p_val	avg_log2FC	pct.1	pct.2	p_val_adj	cluster
SLC12A1	0	4.19500899427366	0.999	0.256	0	Loop of Henle
RP1	0	4.15864692550572	0.806	0.091	0	Loop of Henle
GP2	0	4.1548874555406	0.736	0.047	0	Loop of Henle
CASR	0	2.58183810325098	0.897	0.227	0	Loop of Henle
PLCB1	0	2.94065661882739	0.919	0.273	0	Loop of Henle
CACNA2D3	0	2.61151718161411	0.934	0.291	0	Loop of Henle
SIM2	0	3.70061301190376	0.632	0.052	0	Loop of Henle
LINC01606	0	2.48767317967586	0.801	0.228	0	Loop of Henle
LINC01762	0	2.2079771070963	0.831	0.259	0	Loop of Henle
ESRRB	0	2.79998793388703	0.738	0.169	0	Loop of Henle
PDE1A	0	2.29776165211623	0.911	0.365	0	Loop of Henle
HS6ST2	0	2.86054817305253	0.674	0.134	0	Loop of Henle
ENOX1	0	3.25806450922521	0.664	0.134	0	Loop of Henle
AL159156.1	0	4.43091864041998	0.565	0.037	0	Loop of Henle
RBFOX1	0	2.24865219374296	0.694	0.171	0	Loop of Henle
SGIP1	0	2.29676076848164	0.666	0.165	0	Loop of Henle
EGF	0	2.09354824876361	0.851	0.35	0	Loop of Henle
HIP1	0	2.26268993243215	0.885	0.394	0	Loop of Henle
PCDH9	0	2.11958922696125	0.796	0.308	0	Loop of Henle
CLDN10-AS1	0	3.48524289892246	0.538	0.056	0	Loop of Henle
GPC5	0	2.5625687798047	0.89	0.416	0	Loop of Henle
KLHL13	0	1.91080029540371	0.787	0.323	0	Loop of Henle
CLCN5	0	2.10683315437557	0.853	0.39	0	Loop of Henle
LINC02343	0	2.41491819555514	0.622	0.165	0	Loop of Henle
SLC16A12	0	1.54573589979085	0.769	0.313	0	Loop of Henle
STK32B	0	1.7299970172018	0.717	0.267	0	Loop of Henle
RANBP3L	0	2.52184123817825	0.572	0.127	0	Loop of Henle
NPSR1-AS1	0	2.81116226620228	0.553	0.116	0	Loop of Henle
PHACTR1	0	2.90851488360911	0.576	0.144	0	Loop of Henle
UGT8	0	2.28619696872678	0.593	0.164	0	Loop of Henle
CCDC148	0	1.83848612095964	0.763	0.338	0	Loop of Henle
TMEM72	0	1.70863136966996	0.638	0.215	0	Loop of Henle
AC092078.2	0	3.20722853023585	0.529	0.108	0	Loop of Henle
KNG1	0	1.5795167263554	0.593	0.175	0	Loop of Henle
WNK4	0	1.76814260131744	0.587	0.169	0	Loop of Henle
FAM184A	0	2.29857578322028	0.56	0.143	0	Loop of Henle
SLC4A7	0	2.56027534557379	0.582	0.166	0	Loop of Henle
CA10	0	1.66060277967973	0.598	0.19	0	Loop of Henle
NELL1	0	1.99855444680163	0.61	0.204	0	Loop of Henle
SCHLAP1	0	3.2664463216137	0.464	0.059	0	Loop of Henle
THSD4	0	1.79192619709173	0.668	0.264	0	Loop of Henle
NRK	0	2.51091330250332	0.517	0.113	0	Loop of Henle

gene	p_val	avg_log2FC	pct.1	pct.2	p_val_adj	cluster
INPP4B	0	1.8009659094227	0.639	0.236	0	Loop of Henle
NTRK2	0	3.54939196513319	0.447	0.048	0	Loop of Henle
EFHD1	0	1.76269760131224	0.659	0.262	0	Loop of Henle
OSBPL3	0	1.26604340001887	0.741	0.344	0	Loop of Henle
CACNB2	0	1.43697862340852	0.586	0.192	0	Loop of Henle
ACPP	0	3.32715437325686	0.437	0.048	0	Loop of Henle
LINC00970	0	1.58364815181791	0.67	0.285	0	Loop of Henle
SUSD4	0	2.15565832801399	0.508	0.124	0	Loop of Henle
MYO3B	0	1.65112923793228	0.616	0.234	0	Loop of Henle
SCD5	0	2.07314277654605	0.552	0.172	0	Loop of Henle
SEMA6D	0	2.17044674318934	0.526	0.146	0	Loop of Henle
EFNA5	0	1.31800289380999	0.784	0.41	0	Loop of Henle
TBC1D4	0	1.41332037972963	0.764	0.391	0	Loop of Henle
USP53	0	1.65852488536056	0.754	0.383	0	Loop of Henle
PRDM16	0	1.23723919338297	0.657	0.288	0	Loop of Henle
RHOBTB3	0	1.74933743755899	0.722	0.355	0	Loop of Henle
TFAP2B	0	2.40734708334032	0.448	0.083	0	Loop of Henle
CLCNKB	0	1.13272129339076	0.658	0.294	0	Loop of Henle
CPEB3	0	1.59397401508314	0.775	0.412	0	Loop of Henle
CYFIP2	0	1.43938841836977	0.797	0.436	0	Loop of Henle
MGAT5	0	1.4877498661176	0.613	0.255	0	Loop of Henle
AC068631.1	0	3.09087000167673	0.414	0.057	0	Loop of Henle
PTGER3	0	1.11414386536029	0.788	0.433	0	Loop of Henle
SKAP1	0	1.11115722787633	0.698	0.343	0	Loop of Henle
PRKX	0	2.15291331423862	0.457	0.104	0	Loop of Henle
PRKD1	0	1.84546682080559	0.814	0.462	0	Loop of Henle
NR2F2-AS1	0	1.29017234143242	0.831	0.48	0	Loop of Henle
KCTD1	0	1.55906119618182	0.638	0.291	0	Loop of Henle
PPM1L	0	1.83188729802533	0.637	0.292	0	Loop of Henle
ARHGEF38	0	1.60054651190334	0.538	0.196	0	Loop of Henle
LRP1B	0	1.19648599713067	0.684	0.343	0	Loop of Henle
CLDN16	0	3.80338757819426	0.376	0.035	0	Loop of Henle
ATP1A1	0	1.39069223401222	0.854	0.519	0	Loop of Henle
DEPTOR	0	1.38806217392115	0.649	0.318	0	Loop of Henle
ADGRV1	0	2.0027785442258	0.438	0.109	0	Loop of Henle
LMO7	0	1.62976121912712	0.803	0.475	0	Loop of Henle
TENM4	0	2.05299162588114	0.462	0.134	0	Loop of Henle
GALNT18	0	1.1098345848527	0.679	0.352	0	Loop of Henle
SLC16A7	0	1.1786320054687	0.628	0.301	0	Loop of Henle
NUDT4	0	1.69773653006054	0.522	0.195	0	Loop of Henle
CKB	0	1.82398233112191	0.479	0.152	0	Loop of Henle
CREB3L2	0	1.8724030570363	0.533	0.207	0	Loop of Henle

gene	p_val	avg_log2FC	pct.1	pct.2	p_val_adj	cluster
LINC01182	0	1.91578513015232	0.461	0.136	0	Loop of Henle
LNX1	0	1.47845314889911	0.533	0.209	0	Loop of Henle
STK39	0	1.2218722353509	0.631	0.31	0	Loop of Henle
PANTR1	0	1.94098475585541	0.463	0.146	0	Loop of Henle
WDFY2	0	1.36538185647313	0.639	0.322	0	Loop of Henle
AK3	0	1.13267344969396	0.701	0.385	0	Loop of Henle
RNF150	0	1.35730968319536	0.627	0.314	0	Loop of Henle
MPC1	0	1.17522861034757	0.623	0.31	0	Loop of Henle
RALGPS1	0	1.12718851807077	0.663	0.351	0	Loop of Henle
NAV2	0	1.32574330515791	0.646	0.335	0	Loop of Henle
AC011246.1	0	3.43888834721291	0.453	0.143	0	Loop of Henle
MALRD1	0	2.06910827866674	0.441	0.131	0	Loop of Henle
MYO10	0	0.829390916027281	0.68	0.373	0	Loop of Henle
FXYD2	0	0.957770260482025	0.709	0.403	0	Loop of Henle
AC138305.1	0	1.13312239334672	0.701	0.395	0	Loop of Henle
DNM3	0	0.890599610459668	0.544	0.239	0	Loop of Henle
MROH7	0	1.38534754415902	0.519	0.216	0	Loop of Henle
CXCL12	0	3.62664698694878	0.326	0.024	0	Loop of Henle
PPFIA2	0	2.28285976026996	0.388	0.088	0	Loop of Henle
F11	0	2.19120611883186	0.382	0.083	0	Loop of Henle
KAZN	0	0.874795554124446	0.838	0.539	0	Loop of Henle
AC040174.1	0	1.36559087553445	0.513	0.216	0	Loop of Henle
SCNN1A	0	0.987167673201681	0.6	0.303	0	Loop of Henle
ITPR1	0	0.650598266004094	0.691	0.396	0	Loop of Henle
TRIM2	0	1.09786294734148	0.634	0.341	0	Loop of Henle
MTURN	0	1.76008338765432	0.432	0.139	0	Loop of Henle
DNASE1	0	1.34897626358627	0.503	0.211	0	Loop of Henle
FAM171A1	0	0.99327321003115	0.563	0.271	0	Loop of Henle
ATP1B1	0	1.38217409935241	0.924	0.636	0	Loop of Henle
CFAP221	0	1.40448469285767	0.501	0.213	0	Loop of Henle
KCNJ1	0	1.54137277135244	0.443	0.155	0	Loop of Henle
BICDL1	0	1.22812791115253	0.637	0.349	0	Loop of Henle
ADAMTS17	0	0.849829849400472	0.46	0.173	0	Loop of Henle
PRDM16-DT	0	1.7705972124392	0.414	0.127	0	Loop of Henle
AL139383.1	0	1.85546210646931	0.414	0.128	0	Loop of Henle
COL4A3	0	0.516234780621509	0.665	0.38	0	Loop of Henle
ACOT11	0	1.09207805940363	0.585	0.3	0	Loop of Henle
ERBB4	0	1.85881444309767	0.979	0.695	0	Loop of Henle
TMEM72-AS1	0	2.19456706194152	0.389	0.105	0	Loop of Henle
CGNL1	0	1.4503597625885	0.916	0.632	0	Loop of Henle
ARHGAP6	0	1.41538323557314	0.934	0.65	0	Loop of Henle
NMD3	0	1.45209398240924	0.496	0.214	0	Loop of Henle

gene	p_val	avg_log2FC	pct.1	pct.2	p_val_adj	cluster
SLC2A13	0	1.24706085320013	0.493	0.212	0	Loop of Henle
KCNQ1	0	0.822550827960803	0.604	0.323	0	Loop of Henle
ADAMTS9-AS2	0	0.612653656461995	0.854	0.574	0	Loop of Henle
KCNIP4	0	0.902256821787661	0.799	0.521	0	Loop of Henle
PPARGC1A	0	1.06315196020261	0.817	0.539	0	Loop of Henle
ZNF98	0	1.69099916271839	0.448	0.171	0	Loop of Henle
LINC01725	0	0.970187031794974	0.627	0.352	0	Loop of Henle
CCND3	0	1.05859343774485	0.462	0.187	0	Loop of Henle
STXBP4	0	1.33644264505138	0.545	0.27	0	Loop of Henle
CCDC178	0	1.36426859654873	0.468	0.194	0	Loop of Henle
PTPN4	0	1.21005073785989	0.721	0.447	0	Loop of Henle
PRKCQ	0	1.99016450506048	0.357	0.083	0	Loop of Henle
CPM	0	0.670644343577587	0.601	0.327	0	Loop of Henle
SLC25A33	0	1.62178141374458	0.459	0.186	0	Loop of Henle
FGF13	0	0.815085546300789	0.582	0.309	0	Loop of Henle
CCSER1	0	1.59688446952976	0.989	0.718	0	Loop of Henle
SMC5-AS1	0	1.11543130400667	0.516	0.245	0	Loop of Henle
AC024022.1	0	1.96978851758635	0.355	0.084	0	Loop of Henle
AC124017.1	0	4.54787083291702	0.283	0.012	0	Loop of Henle
ZDHHC14	0	0.814743868743369	0.566	0.295	0	Loop of Henle
GNAI1	0	1.65166072863656	0.41	0.139	0	Loop of Henle
GUCY1A1	0	2.25169367232615	0.338	0.068	0	Loop of Henle
MRPS6	0	1.53907440758887	0.49	0.221	0	Loop of Henle
FBXL4	0	1.37500659123261	0.503	0.235	0	Loop of Henle
PDE4DIP	0	0.932428167094266	0.608	0.34	0	Loop of Henle
AGPAT4	0	1.96237904347173	0.355	0.091	0	Loop of Henle
CFAP70	0	1.12718465255234	0.505	0.241	0	Loop of Henle
COL28A1	0	1.8523211068998	0.39	0.127	0	Loop of Henle
ACACB	0	0.874394058549802	0.74	0.477	0	Loop of Henle
ALDH1A2	0	0.443725872810082	0.412	0.149	0	Loop of Henle
CECR2	0	1.29401458282531	0.466	0.203	0	Loop of Henle
SSBP2	0	0.865808384231248	0.571	0.308	0	Loop of Henle
PDE7B	0	0.639177238191038	0.679	0.417	0	Loop of Henle
TMEM161B-AS1	0	1.01254023092251	0.616	0.357	0	Loop of Henle
AL132780.1	0	1.73526857466981	0.392	0.133	0	Loop of Henle
ZFAND5	0	0.888738335291273	0.561	0.303	0	Loop of Henle
GSAP	0	1.22565985109902	0.447	0.189	0	Loop of Henle
ZNF385B	0	0.788592684745916	0.453	0.196	0	Loop of Henle
FKBP5	0	1.02031735073277	0.753	0.496	0	Loop of Henle
NAIP	0	1.08069556746086	0.52	0.264	0	Loop of Henle
ABHD17C	0	1.99827319383373	0.353	0.097	0	Loop of Henle
MGLL	0	1.10944578173598	0.473	0.218	0	Loop of Henle

gene	p_val	avg_log2FC	pct.1	pct.2	p_val_adj	cluster
HIBADH	0	1.16699864599119	0.625	0.371	0	Loop of Henle
CADM1	0	0.805757844785581	0.489	0.235	0	Loop of Henle
DSCAML1	0	1.12121467633457	0.545	0.292	0	Loop of Henle
MECOM	0	0.707943104033137	0.953	0.7	0	Loop of Henle
TLE1	0	1.72252347819155	0.364	0.113	0	Loop of Henle
IVNS1ABP	0	0.545087867213684	0.575	0.325	0	Loop of Henle
KATNAL2	0	1.0552749636193	0.502	0.253	0	Loop of Henle
DENND2A	0	1.78853844085973	0.343	0.095	0	Loop of Henle
ACSS1	0	1.5708821392353	0.396	0.148	0	Loop of Henle
WLS	0	0.947895312396149	0.517	0.269	0	Loop of Henle
CHL1	0	1.11816817972329	0.412	0.165	0	Loop of Henle
SULT1C2	0	0.672309016358196	0.743	0.497	0	Loop of Henle
PRICKLE2	0	0.957239467482683	0.651	0.405	0	Loop of Henle
NAALADL2	0	0.847850758546861	0.873	0.627	0	Loop of Henle
RNF213-AS1	0	0.884555783257894	0.576	0.33	0	Loop of Henle
ARHGAP8	0	0.759306793012389	0.56	0.317	0	Loop of Henle
USP2	0	1.10507477055249	0.393	0.15	0	Loop of Henle
ETNK1	0	1.16903672164827	0.537	0.295	0	Loop of Henle
WWC2	0	0.836912019395925	0.662	0.421	0	Loop of Henle
AC138123.1	0	1.54822116797851	0.399	0.158	0	Loop of Henle
LINC00476	0	1.18893672565518	0.44	0.199	0	Loop of Henle
DNAH14	0	0.643238672958715	0.715	0.475	0	Loop of Henle
TFCP2L1	0	0.509770625319788	0.779	0.54	0	Loop of Henle
P3H2	0	0.932822931961683	0.67	0.432	0	Loop of Henle
FAM160A1	0	0.642250862136844	0.677	0.439	0	Loop of Henle
LINC00645	0	1.37115443648705	0.339	0.101	0	Loop of Henle
GRHL2	0	0.997070286430912	0.446	0.209	0	Loop of Henle
PDE9A	0	1.10812963227725	0.45	0.213	0	Loop of Henle
KCTD16	0	2.34455655675411	0.292	0.055	0	Loop of Henle
ARHGAP18	0	0.458663948427007	0.588	0.351	0	Loop of Henle
SYTL4	0	2.29084092667247	0.299	0.062	0	Loop of Henle
SGK2	0	0.743082397338425	0.566	0.329	0	Loop of Henle
LRP6	0	0.819983137223059	0.55	0.314	0	Loop of Henle
SLC25A12	0	0.932040480588022	0.512	0.276	0	Loop of Henle
TAPT1-AS1	0	0.82352741940993	0.64	0.404	0	Loop of Henle
CMTM4	0	0.980237658557941	0.539	0.304	0	Loop of Henle
PHF21B	0	1.52127632247013	0.34	0.105	0	Loop of Henle
CLCNKA	0	1.16124556923962	0.437	0.202	0	Loop of Henle
C2CD5	0	0.954761445230312	0.504	0.269	0	Loop of Henle
DZIP1	0	1.20560284069776	0.394	0.16	0	Loop of Henle
RAP1GAP2	0	0.940238325942221	0.454	0.221	0	Loop of Henle
FGF14	0	2.58328891630457	0.318	0.086	0	Loop of Henle

gene	p_val	avg_log2FC	pct.1	pct.2	p_val_adj	cluster
GNG7	0	1.38101765555413	0.449	0.217	0	Loop of Henle
MFSD4A	0	2.21377357589244	0.294	0.062	0	Loop of Henle
TANC2	0	0.607933041876866	0.571	0.339	0	Loop of Henle
PLCL1	0	1.11798820716755	0.92	0.689	0	Loop of Henle
NHS	0	0.616434788964987	0.812	0.581	0	Loop of Henle
MFHAS1	0	0.747216783603389	0.555	0.324	0	Loop of Henle
ARHGAP24	0	0.723354257680591	0.742	0.511	0	Loop of Henle
MAPK4	0	1.75388961674334	0.34	0.11	0	Loop of Henle
TCIM	0	2.04295068537476	0.298	0.068	0	Loop of Henle
AZIN1-AS1	0	1.0726521101931	0.485	0.255	0	Loop of Henle
CLMN	0	0.619438692822961	0.723	0.493	0	Loop of Henle
SIM1	0	0.747047868973061	0.472	0.243	0	Loop of Henle
LAMA1	0	1.68503732847983	0.339	0.111	0	Loop of Henle
CEP70	0	1.05125881023643	0.475	0.247	0	Loop of Henle
AC245297.3	0	0.817144936065479	0.54	0.313	0	Loop of Henle
PKP4	0	1.20358896490101	0.888	0.662	0	Loop of Henle
PLEKHH2	0	1.17737381248358	0.397	0.172	0	Loop of Henle
SEC14L1	0	0.753509463127935	0.467	0.242	0	Loop of Henle
PIK3R1	0	0.900518381366719	0.443	0.218	0	Loop of Henle
NOS1	0	1.75073289055736	0.324	0.099	0	Loop of Henle
MERTK	0	1.49052963178415	0.371	0.147	0	Loop of Henle
AL353151.2	0	2.09249418491218	0.302	0.078	0	Loop of Henle
ABLIM1	0	0.616921078906463	0.807	0.584	0	Loop of Henle
LIFR	0	0.731749794773552	0.597	0.374	0	Loop of Henle
BTBD11	0	0.738693088909127	0.44	0.218	0	Loop of Henle
SGPP2	0	1.02367513125545	0.434	0.213	0	Loop of Henle
HOXB3	0	0.825764562882728	0.454	0.233	0	Loop of Henle
GOT1	0	1.00055132456103	0.457	0.238	0	Loop of Henle
HOMER1	0	1.05921908079658	0.378	0.16	0	Loop of Henle
AC087564.1	0	1.11022752317769	0.432	0.215	0	Loop of Henle
MAMDC2-AS1	0	1.51574823952908	0.345	0.128	0	Loop of Henle
GPI	0	0.810166230252925	0.472	0.256	0	Loop of Henle
DNAH7	0	0.74545630159565	0.5	0.285	0	Loop of Henle
MYO9A	0	0.795780940051993	0.863	0.648	0	Loop of Henle
AL645568.1	0	1.44932089565766	0.337	0.123	0	Loop of Henle
MYLK	0	1.00915511386128	0.385	0.171	0	Loop of Henle
UBE2E3	0	0.88333207434168	0.495	0.281	0	Loop of Henle
ERICH1	0	0.892856053206757	0.557	0.345	0	Loop of Henle
CALCR	0	1.31877731603648	0.331	0.119	0	Loop of Henle
CCDC171	0	0.772100113125776	0.494	0.282	0	Loop of Henle
MYOF	0	1.05120956692835	0.397	0.186	0	Loop of Henle
RIMBP2	0	1.45647956124563	0.334	0.123	0	Loop of Henle

gene	p_val	avg_log2FC	pct.1	pct.2	p_val_adj	cluster
AKAP6	0	0.895749652225623	0.452	0.241	0	Loop of Henle
COBLL1	0	0.285495447095377	0.901	0.691	0	Loop of Henle
CAPN2	0	0.867287391032649	0.445	0.235	0	Loop of Henle
RBM20	0	2.11775017663125	0.273	0.063	0	Loop of Henle
FAM227B	0	1.10742035744308	0.375	0.165	0	Loop of Henle
MEF2C	0	0.562874593426122	0.364	0.155	0	Loop of Henle
OXR1	0	0.882123808045813	0.837	0.628	0	Loop of Henle
AC008269.1	0	0.949392341212876	0.415	0.207	0	Loop of Henle
XPA	0	1.41545576045681	0.339	0.132	0	Loop of Henle
RPS6KA6	0	1.16646759541834	0.391	0.184	0	Loop of Henle
BDH1	0	1.29103613003721	0.339	0.133	0	Loop of Henle
MOB3B	0	0.974202785381426	0.441	0.235	0	Loop of Henle
AMMECR1	0	1.48328272662943	0.339	0.133	0	Loop of Henle
ATP2C1	0	0.874630016330731	0.456	0.251	0	Loop of Henle
TRMT9B	0	1.0147043980374	0.384	0.18	0	Loop of Henle
CTIF	0	0.910204857317703	0.427	0.223	0	Loop of Henle
PXDNL	0	2.27973854394079	0.284	0.08	0	Loop of Henle
FGF9	0	1.44164105927905	0.319	0.116	0	Loop of Henle
SPAG17	0	1.23254639852702	0.347	0.145	0	Loop of Henle
SLC22A15	0	0.88882049926182	0.416	0.215	0	Loop of Henle
USP13	0	1.85590256845379	0.265	0.065	0	Loop of Henle
LPIN1	0	1.19076378751714	0.365	0.165	0	Loop of Henle
MUC1	0	1.08273812858996	0.357	0.158	0	Loop of Henle
SH3BP4	0	1.12567724134288	0.369	0.17	0	Loop of Henle
NRCAM	0	0.919565226740912	0.365	0.166	0	Loop of Henle
SLCO3A1	0	0.847449776047885	0.356	0.159	0	Loop of Henle
LIPA	0	0.955982416002264	0.351	0.157	0	Loop of Henle
RRAGD	0	1.80655745175669	0.273	0.08	0	Loop of Henle
TMEM164	0	1.07209324068301	0.376	0.184	0	Loop of Henle
PCCA-DT	0	1.54363915867485	0.284	0.093	0	Loop of Henle
SFRP1	0	1.88754326359467	0.256	0.066	0	Loop of Henle
AC009879.4	0	1.30470493247673	0.326	0.136	0	Loop of Henle
OCLN	0	1.13868451184829	0.337	0.148	0	Loop of Henle
MGME1	0	1.24671645199709	0.308	0.119	0	Loop of Henle
TTC7A	0	1.0227232237735	0.372	0.185	0	Loop of Henle
U91319.1	0	1.82284298351767	0.259	0.072	0	Loop of Henle
KHDRBS3	0	0.924057857859335	0.344	0.158	0	Loop of Henle
MSI2	0	0.51715973321293	0.876	0.691	0	Loop of Henle
GAD1	0	1.56712135877278	0.252	0.067	0	Loop of Henle
NR2F1-AS1	0	1.05894442281262	0.299	0.115	0	Loop of Henle
FGD4	0	0.593161156263273	0.84	0.656	0	Loop of Henle
HDAC9	0	1.26561611388482	0.266	0.085	0	Loop of Henle

gene	p_val	avg_log2FC	pct.1	pct.2	p_val_adj	cluster
OXCT1	0	1.05701196683237	0.304	0.125	0	Loop of Henle
SLC22A3	0	1.65472263588671	0.262	0.083	0	Loop of Henle
AC016705.2	0	1.51314840557034	0.277	0.1	0	Loop of Henle
AL078600.1	0	1.31019772344987	0.298	0.122	0	Loop of Henle
GTF2H2C	0	1.31154482414555	0.301	0.126	0	Loop of Henle
ESRRG	0	1.21134927247594	0.977	0.803	0	Loop of Henle
MAGI1	0	0.340002740773392	0.913	0.74	0	Loop of Henle
RNF24	0	1.09935860249769	0.312	0.141	0	Loop of Henle
GSTO2	0	1.1651520884777	0.308	0.137	0	Loop of Henle
SHISA9	0	1.5119953090413	0.254	0.083	0	Loop of Henle
FARP1	0	0.519356155150402	0.848	0.678	0	Loop of Henle
FAM83B	0	1.21873176412503	0.285	0.116	0	Loop of Henle
BDKRB2	0	1.52539170867705	0.25	0.081	0	Loop of Henle
PPM1K	0	1.23758908572896	0.287	0.12	0	Loop of Henle
AC004870.4	0	1.24648636192038	0.262	0.096	0	Loop of Henle
SLC6A16	0	1.46081574288479	0.251	0.085	0	Loop of Henle
AP1S3	0	1.28035157426195	0.269	0.104	0	Loop of Henle
UPP2	0	0.971462077290354	0.281	0.117	0	Loop of Henle
RPL34-AS1	0	1.23708826964116	0.272	0.108	0	Loop of Henle
LRBA	0	0.653128730289476	0.879	0.716	0	Loop of Henle
AC104596.1	0	1.31461128348681	0.271	0.109	0	Loop of Henle
DOCK3	0	1.13422266662941	0.284	0.124	0	Loop of Henle
AC083843.3	0	1.35882580261334	0.262	0.103	0	Loop of Henle
AC025159.1	0	1.45858926624317	0.254	0.096	0	Loop of Henle
GOLM1	0	1.12043180086535	0.26	0.105	0	Loop of Henle
ARRDC3	0	1.01008088517738	0.266	0.112	0	Loop of Henle
IMMP2L	0	0.764370860360699	0.914	0.776	0	Loop of Henle
RAP1GAP	6.32743124865458e-308	1.04436445528035	0.33	0.157	1.8298931171109e-303	Loop of Henle
BACE2	7.16882370269973e-305	0.795332926573366	0.499	0.29	2.07322381482076e-300	Loop of Henle
MAL	1.27079451563864e-303	0.721086930741022	0.42	0.218	3.67513773922694e-299	Loop of Henle
ARNT2	4.30463585530652e-303	0.805883875774277	0.421	0.222	1.24490068935465e-298	Loop of Henle
CLINT1	3.60843293731709e-302	0.744838949944053	0.515	0.307	1.0435588054721e-297	Loop of Henle
AHCYL1	1.3600004175004e-300	0.923098672798709	0.413	0.226	3.93312120741115e-296	Loop of Henle
MAGED1	1.8535357743979e-300	0.818841076697708	0.483	0.282	5.36042545955872e-296	Loop of Henle
TMEM232	2.56716428605133e-292	0.730130816963717	0.524	0.316	7.42423911526044e-288	Loop of Henle
ASB3	1.00444790721579e-291	0.836118737586369	0.42	0.23	2.90486334766806e-287	Loop of Henle
LINC01594	8.33322544066129e-291	1.22168959724284	0.252	0.106	2.40996879743924e-286	Loop of Henle
USP25	1.02056697803279e-290	0.605648060083915	0.686	0.477	2.95147970047082e-286	Loop of Henle
GCNT2	7.90562856499187e-290	0.644465407975418	0.568	0.346	2.28630778099565e-285	Loop of Henle
PDE8B	3.49436311619591e-289	1.10640106895654	0.285	0.13	1.01056981320386e-284	Loop of Henle
PTPN14	8.73474688582678e-289	0.717788565255105	0.559	0.351	2.5260887993811e-284	Loop of Henle
KLF12	1.6196103694416e-288	0.643299228813849	0.647	0.444	4.6839131884251e-284	Loop of Henle

gene	p_val	avg_log2FC	pct.1	pct.2	p_val_adj	cluster
PXN	8.18760024621322e-288	0.77636872171613	0.34	0.165	2.36785399120486e-283	Loop of Henle
KCNJ16	3.58140108944266e-287	0.480668644918953	0.783	0.553	1.03574119506682e-282	Loop of Henle
CDKL1	4.80623970602252e-285	0.695860940783878	0.471	0.266	1.38996452298171e-280	Loop of Henle
TPST1	3.16642145652916e-283	0.657441488035183	0.566	0.346	9.15729085228232e-279	Loop of Henle
EPM2A	2.50136900744613e-281	1.03721648029075	0.313	0.151	7.23395916953421e-277	Loop of Henle
ST3GAL6	1.18283309774256e-280	0.438595737826132	0.378	0.189	3.42075331867148e-276	Loop of Henle
TAOK3	7.58297110339397e-279	0.642305579468947	0.598	0.388	2.19299524310154e-274	Loop of Henle
PBX3	6.42174167988549e-277	0.86476271255558	0.371	0.194	1.85716769382288e-272	Loop of Henle
PCCA	3.93260431297614e-275	0.519937092349511	0.832	0.676	1.1373091673127e-270	Loop of Henle
OIP5-AS1	5.83263873192853e-275	0.773816103975596	0.419	0.233	1.68679912127373e-270	Loop of Henle
SOX2-OT	6.00964470660906e-274	1.03016987192054	0.263	0.118	1.73798924915134e-269	Loop of Henle
AL035530.2	1.04497983309354e-271	1.09912304410343	0.263	0.118	3.02208167730652e-267	Loop of Henle
FSIP2-AS1	2.62092959144443e-271	1.13163381862501	0.278	0.129	7.5797283784573e-267	Loop of Henle
AL049828.1	9.1932166255741e-269	0.81202090125324	0.556	0.361	2.65867824811603e-264	Loop of Henle
TTC14	4.34930822349134e-265	0.669700350304913	0.533	0.331	1.25781993823369e-260	Loop of Henle
FOXO3	1.91834281371271e-263	0.631729174923527	0.599	0.401	5.54784741725715e-259	Loop of Henle
ACSS3	1.28689298102215e-262	0.627554049479581	0.47	0.273	3.72169450111605e-258	Loop of Henle
ITGA2	2.39682307541286e-262	0.40431089451713	0.445	0.246	6.93161233409401e-258	Loop of Henle
CCDC146	3.90939002566916e-262	0.753908323918039	0.39	0.21	1.13059559542352e-257	Loop of Henle
SEPTIN6	8.73752636105852e-262	0.830705386402594	0.289	0.135	2.52689262361812e-257	Loop of Henle
KRBA2	1.7230293411702e-261	1.04440244252556	0.273	0.126	4.98300085466423e-257	Loop of Henle
PLA2G12A	1.09372084271649e-260	0.897225304153662	0.357	0.19	3.1630406771361e-256	Loop of Henle
STRBP	1.70669178572808e-260	0.668729543795846	0.521	0.323	4.93575264432561e-256	Loop of Henle
SLC15A2	1.96882570073062e-259	0.783764761039591	0.261	0.116	5.69384392651296e-255	Loop of Henle
SRSF10	4.27703773203164e-259	0.686801036572666	0.472	0.279	1.23691931210355e-254	Loop of Henle
LLGL2	1.28775298523466e-258	0.596255297245526	0.626	0.413	3.72418163329864e-254	Loop of Henle
MYO5C	2.37747174792751e-258	0.920880577819168	0.33	0.168	6.87564829500636e-254	Loop of Henle
RIC8B	8.8079755592045e-258	0.704111075455069	0.405	0.224	2.54726653172194e-253	Loop of Henle
PFKP	5.37558157623012e-257	0.731301126905787	0.333	0.167	1.55461819184575e-252	Loop of Henle
NR3C2	7.85407491290289e-257	0.390999117106396	0.855	0.648	2.27139846481152e-252	Loop of Henle
NFYC	1.16931700135805e-256	0.702872395828558	0.489	0.297	3.38166476792749e-252	Loop of Henle
GNGT1	1.63745384598298e-256	1.0536185052693	0.355	0.19	4.73551652258278e-252	Loop of Henle
ZRANB2	3.38159426451407e-255	0.570376469105459	0.682	0.472	9.77957061297468e-251	Loop of Henle
SLC3A1	5.44951267729369e-255	0.6402954064453	0.495	0.294	1.57599906627333e-250	Loop of Henle
CFLAR	2.13031458733017e-254	0.505937895404078	0.673	0.466	6.16086978655886e-250	Loop of Henle
TPST2	6.77625386225732e-253	0.93609437182855	0.273	0.127	1.95969261696482e-248	Loop of Henle
PIAS2	7.44070059756097e-252	0.718789885158817	0.471	0.284	2.15185061281463e-247	Loop of Henle
ITPRID2	3.66012668189173e-251	0.759116633390393	0.328	0.165	1.05850863640309e-246	Loop of Henle
ATF7IP2	1.90568771546577e-250	0.788727907395339	0.362	0.192	5.511248873127e-246	Loop of Henle
DANT2	2.5921735099356e-249	0.458769318325857	0.851	0.664	7.49656579073376e-245	Loop of Henle
SLCO4C1	2.95244074535498e-249	0.926657775307638	0.255	0.115	8.53845863556662e-245	Loop of Henle
LUC7L2	3.31516682083239e-248	0.64087422547083	0.562	0.372	9.58746244584728e-244	Loop of Henle

gene	p_val	avg_log2FC	pct.1	pct.2	p_val_adj	cluster
SPATA13	9.64986680955805e-248	0.345944724493034	0.632	0.414	2.79074148132419e-243	Loop of Henle
SGCE	8.16707271884511e-244	0.996019304846141	0.25	0.114	2.36191743029001e-239	Loop of Henle
LINC01117	5.20017165191394e-243	0.996421664172554	0.268	0.126	1.50388964173351e-238	Loop of Henle
PALM	1.41830756727215e-242	0.885695190164052	0.267	0.124	4.10174548455105e-238	Loop of Henle
MIR29B2CHG	2.43758087100738e-242	0.610749637367039	0.553	0.36	7.04948387895333e-238	Loop of Henle
ANK2	2.91036703423148e-242	0.308178290330041	0.59	0.366	8.41678146299743e-238	Loop of Henle
MICU3	6.90853445280844e-241	0.770706098635483	0.331	0.171	1.9979481637522e-236	Loop of Henle
FAM13A	1.81150708268768e-240	0.686388619411172	0.544	0.36	5.23887848313276e-236	Loop of Henle
STPG2	1.31486619420274e-239	0.659266521341065	0.568	0.376	3.80259303363432e-235	Loop of Henle
CASK	2.56376034903059e-239	0.59045990100698	0.485	0.298	7.41439492939648e-235	Loop of Henle
LINC01320	3.23667483176348e-238	0.33409247566093	0.676	0.446	9.36046361345997e-234	Loop of Henle
IDH2	2.1724093671765e-235	0.794848129789019	0.336	0.176	6.28260788987442e-231	Loop of Henle
RAP2C-AS1	2.54205294913286e-235	0.789818164020668	0.428	0.253	7.35161712889223e-231	Loop of Henle
CMC1	8.52420451521959e-234	0.793694545693406	0.38	0.214	2.46519994580151e-229	Loop of Henle
SIL1	1.00871899160291e-233	0.607300192364828	0.574	0.378	2.91721532371562e-229	Loop of Henle
LGR4	2.45593211121671e-231	0.553426159449573	0.687	0.496	7.10255566563874e-227	Loop of Henle
WDR91	3.48042845162288e-231	0.730208343196881	0.331	0.173	1.00653990820934e-226	Loop of Henle
ARMCX4	8.64432317869063e-231	0.678447225564719	0.492	0.307	2.49993826327733e-226	Loop of Henle
RGL3	1.27577828819737e-230	0.596147513268452	0.525	0.328	3.68955080946679e-226	Loop of Henle
TDRD3	9.17625999844728e-230	0.589922580773989	0.567	0.378	2.65377439155095e-225	Loop of Henle
HNF1A-AS1	1.19245399227547e-228	0.484119276840673	0.65	0.428	3.44857694566065e-224	Loop of Henle
ETFDH	1.94542305008774e-228	0.877405672996426	0.312	0.163	5.62616346085374e-224	Loop of Henle
LINC02256	1.18469089586578e-227	0.737113217137688	0.417	0.246	3.42612607084383e-223	Loop of Henle
HPRT1	1.50495543220744e-227	0.964641442182867	0.272	0.133	4.3523311099439e-223	Loop of Henle
MBNL2	1.7531163124679e-227	0.531896227527003	0.644	0.452	5.07001237565717e-223	Loop of Henle
PLIN5	2.60972497176634e-227	0.695290058829029	0.428	0.248	7.54732461834826e-223	Loop of Henle
NNT	2.70457941556049e-227	0.615117792252245	0.499	0.31	7.82164366980093e-223	Loop of Henle
SVIL	9.57351068997703e-227	0.78921571275446	0.567	0.387	2.76865929154136e-222	Loop of Henle
CYS1	1.21709729320562e-226	0.682588949414714	0.373	0.206	3.51984537195064e-222	Loop of Henle
GALNT10	1.41203654115978e-226	0.787233325925713	0.336	0.179	4.08360967703408e-222	Loop of Henle
ZKSCAN7-AS1	2.3989386992741e-225	0.935754455817266	0.261	0.126	6.93773071830069e-221	Loop of Henle
AC021231.1	2.5122691800068e-225	0.947009408906012	0.31	0.163	7.26548246857967e-221	Loop of Henle
UQCRC2	4.62185640660969e-225	0.727820878558548	0.389	0.223	1.33664087279152e-220	Loop of Henle
BLNK	6.34061934640709e-225	0.647041604504347	0.381	0.21	1.83370711498093e-220	Loop of Henle
Z93930.2	8.69241359021717e-225	0.845616617249609	0.34	0.184	2.5138460102908e-220	Loop of Henle
CALM2	2.34659904031623e-223	0.787258496675975	0.305	0.157	6.78636442459455e-219	Loop of Henle
ANKRD28	7.276685203634e-223	0.55404623388869	0.597	0.403	2.10441736089095e-218	Loop of Henle
DMGDH	1.06541251611417e-222	0.590046064207705	0.477	0.292	3.08117299660219e-218	Loop of Henle
DAPK1	3.07120107746267e-221	0.496835962014261	0.56	0.363	8.88191351602205e-217	Loop of Henle
NOS1AP	5.83402857502551e-221	0.416592250738639	0.521	0.318	1.68720106389738e-216	Loop of Henle
GPATCH2L	1.51269541534157e-220	0.637591558349785	0.476	0.301	4.37471514116781e-216	Loop of Henle
GABPB1-AS1	5.75285113219824e-220	0.615903732851722	0.427	0.252	1.66372454743173e-215	Loop of Henle

gene	p_val	avg_log2FC	pct.1	pct.2	p_val_adj	cluster
TOM1L2	9.8490755827267e-219	0.512891045326121	0.539	0.342	2.84835265852456e-214	Loop of Henle
ME3	1.29607842944093e-218	0.371193476625008	0.451	0.26	3.74825881794316e-214	Loop of Henle
PRKAA2	4.80077554857191e-218	0.564967918195065	0.506	0.319	1.388384288647e-213	Loop of Henle
CHKA	1.0744034211056e-216	0.656455673696099	0.425	0.253	3.10717469383739e-212	Loop of Henle
TBC1D32	1.00893923520293e-215	0.665138040554112	0.411	0.239	2.91785226820688e-211	Loop of Henle
MSANTD2	2.62349624885622e-214	0.857586081494713	0.291	0.151	7.58715115169218e-210	Loop of Henle
INTS6	5.49464409705278e-212	0.759816746229507	0.435	0.27	1.58905107286767e-207	Loop of Henle
ASXL3	3.62446336271612e-211	0.817699109603625	0.335	0.183	1.0481948044975e-206	Loop of Henle
SLC20A2	1.28483934712299e-210	0.643754011182502	0.513	0.335	3.71575539187969e-206	Loop of Henle
ZNF493	3.27495319217842e-210	0.718458254817219	0.367	0.209	9.47116463177999e-206	Loop of Henle
HOXD3	2.29863271204505e-209	0.503179314085574	0.491	0.301	6.64764580323429e-205	Loop of Henle
MPDZ	4.96519939175299e-209	0.670579968928602	0.427	0.259	1.43593566409496e-204	Loop of Henle
EPN1	1.78165463220544e-208	0.863323923402244	0.261	0.129	5.15254519633814e-204	Loop of Henle
COL4A4	5.80642818197398e-208	0.412998857615408	0.526	0.327	1.67921903022687e-203	Loop of Henle
TFEB	6.28431675020797e-208	0.796340574354385	0.285	0.146	1.81742440416014e-203	Loop of Henle
ANKRD6	3.39837848854727e-207	0.784179608856331	0.328	0.179	9.82811058887872e-203	Loop of Henle
DOCK1	2.82687773227301e-206	0.476194959342733	0.744	0.563	8.17533040173355e-202	Loop of Henle
MBOAT2	1.51489669919128e-205	0.725197077145335	0.36	0.205	4.38108125406118e-201	Loop of Henle
TMEM116	3.82687907858559e-205	0.616278105530426	0.384	0.218	1.10673342952695e-200	Loop of Henle
MIR3936HG	9.53174150405651e-205	0.491096348986426	0.646	0.444	2.75657964297314e-200	Loop of Henle
ZRANB2-AS2	2.80739491733618e-204	0.743517603665161	0.385	0.228	8.11898610093623e-200	Loop of Henle
DSE	1.82654233631017e-203	0.731557704364363	0.383	0.226	5.28236043660902e-199	Loop of Henle
LINC01473	5.98044565938642e-203	0.47741023602632	0.731	0.554	1.72954488469455e-198	Loop of Henle
MTR	1.36862902059546e-202	0.449137625243964	0.744	0.553	3.95807512756207e-198	Loop of Henle
ADHFE1	1.88359310714995e-201	0.661258045751049	0.331	0.182	5.44735126587764e-197	Loop of Henle
AC053527.2	2.26298946175815e-201	0.681158121065772	0.393	0.233	6.54456552340458e-197	Loop of Henle
DTWD1	2.47722784435528e-201	0.817423376154795	0.272	0.139	7.16414292587546e-197	Loop of Henle
DMTF1	6.10878097947266e-201	0.595996777827576	0.456	0.285	1.76665945926349e-196	Loop of Henle
ZHX3	2.84103497769397e-199	0.455621055888314	0.671	0.479	8.21627315549097e-195	Loop of Henle
FRS2	6.4312778476907e-199	0.64325016975675	0.438	0.273	1.85992555355215e-194	Loop of Henle
HNRNPA2B1	2.37220840951851e-198	0.477585964435306	0.737	0.557	6.86042672032754e-194	Loop of Henle
LETMD1	2.57552839078233e-198	0.786041431033501	0.31	0.169	7.4484281061425e-194	Loop of Henle
MIOS	5.0221904091291e-198	0.820144713017102	0.286	0.15	1.45241746632014e-193	Loop of Henle
DAAM1	7.8334072670664e-198	0.563076088573873	0.434	0.264	2.2654213816356e-193	Loop of Henle
PPP1CB	4.01404905112618e-196	0.57771418862859	0.393	0.232	1.16086298558569e-191	Loop of Henle
IFRD1	4.08188971887974e-196	0.682592389885498	0.354	0.204	1.18048250670002e-191	Loop of Henle
ATP6V0A4	4.58585808470043e-196	0.299779331827726	0.505	0.308	1.32623015809537e-191	Loop of Henle
SRGAP3	7.6669678984602e-196	0.561936986199858	0.415	0.247	2.21728711623469e-191	Loop of Henle
ZNF618	9.94359839866531e-196	0.585912651715473	0.499	0.322	2.87568865689401e-191	Loop of Henle
SLC36A4	2.7850360400667e-194	0.703175406174122	0.329	0.183	8.05432422787291e-190	Loop of Henle
RORA	6.32270714644783e-193	0.351242310744825	0.893	0.8	1.82852690675271e-188	Loop of Henle
THRB	7.27854643931252e-193	0.565450319333471	0.633	0.461	2.10495563024918e-188	Loop of Henle

gene	p_val	avg_log2FC	pct.1	pct.2	p_val_adj	cluster
ITPR2	9.12262099909755e-193	0.305961219822561	0.839	0.662	2.63826199293901e-188	Loop of Henle
CYP7B1	2.13964451684596e-192	0.774482331728165	0.307	0.168	6.18785194271851e-188	Loop of Henle
WWOX	4.33442634178758e-192	0.39661553251053	0.877	0.776	1.25351609804497e-187	Loop of Henle
AC092535.4	6.59187164692273e-192	0.75552440051555	0.312	0.171	1.90636928029005e-187	Loop of Henle
GLCCI1	1.2220407981772e-191	0.438822234841591	0.453	0.277	3.53414198832847e-187	Loop of Henle
DICER1	4.34659056496632e-191	0.522077731568962	0.526	0.349	1.25703399138826e-186	Loop of Henle
TCAIM	4.55652646215014e-191	0.617822220030242	0.486	0.314	1.31774745285382e-186	Loop of Henle
TSC22D2	8.29264619857224e-191	0.395364172649976	0.564	0.379	2.39823328062709e-186	Loop of Henle
TFCP2	3.21093957030211e-190	0.589805997380498	0.498	0.325	9.28603723731371e-186	Loop of Henle
CCNH	7.49726239524783e-190	0.535540300387343	0.538	0.367	2.16820828470567e-185	Loop of Henle
MTX2	1.56894881281868e-189	0.588268268477301	0.466	0.298	4.53739996667162e-185	Loop of Henle
CACNA1D	4.07067713235241e-189	0.34392412978453	0.531	0.334	1.17723982667632e-184	Loop of Henle
FAM27C	1.08093212710381e-188	0.795220744043185	0.3	0.163	3.12605571158421e-184	Loop of Henle
ZMYND8	1.5239284062913e-188	0.604626533233553	0.515	0.345	4.40720095099445e-184	Loop of Henle
DECR1	3.35358432015366e-188	0.555972273970976	0.414	0.253	9.69856585388438e-184	Loop of Henle
COX10	6.64420121335167e-187	0.683082907617768	0.351	0.203	1.9215029909013e-182	Loop of Henle
ZNF385D	8.96155752691371e-187	0.53088506318183	0.285	0.15	2.59168243678344e-182	Loop of Henle
ZMIZ1	1.0170190852295e-186	0.515120737179045	0.523	0.344	2.94121919448371e-182	Loop of Henle
SREBF2	1.67830431655934e-186	0.566169412248032	0.517	0.343	4.85365608348962e-182	Loop of Henle
AUH	3.2595337055377e-186	0.501549660518694	0.625	0.447	9.42657147641503e-182	Loop of Henle
GOLGA8B	4.98581143068694e-186	0.591857907530234	0.383	0.227	1.44189666575466e-181	Loop of Henle
LDHB	5.62999479712222e-186	0.556672533939288	0.328	0.183	1.62819449532775e-181	Loop of Henle
CRTC1	9.02188731774601e-186	0.646059405928978	0.346	0.197	2.60912981229215e-181	Loop of Henle
MYEF2	1.82617873479205e-185	0.725642466342575	0.308	0.171	5.2813089010186e-181	Loop of Henle
DHX15	9.65163026429685e-185	0.585767546153324	0.431	0.27	2.79125147243465e-180	Loop of Henle
SLC19A2	1.25755729725341e-184	0.754941657078536	0.293	0.158	3.63685570365687e-180	Loop of Henle
DPH6	3.74315318781347e-183	0.481164161525307	0.678	0.497	1.08251990191565e-178	Loop of Henle
CARMIL1	5.69883659036831e-183	0.407805197716203	0.545	0.36	1.64810354193452e-178	Loop of Henle
DNMT3A	7.79291783158665e-183	0.643317245369218	0.398	0.242	2.25371183689486e-178	Loop of Henle
SUCLG2-AS1	9.00667361243382e-183	0.591023951622288	0.494	0.325	2.60473000871586e-178	Loop of Henle
PCNX2	1.14256181001261e-182	0.573802448939889	0.42	0.258	3.30428875455646e-178	Loop of Henle
COL6A1	2.21443601420106e-182	0.701504988182036	0.305	0.167	6.40414895306947e-178	Loop of Henle
ZNF138	2.70880412917606e-182	0.815488427309774	0.261	0.136	7.83386154157717e-178	Loop of Henle
PSME4	1.95574102597662e-181	0.564242716670663	0.486	0.318	5.65600304712438e-177	Loop of Henle
APP	1.67021818511393e-180	0.297791404801581	0.667	0.492	4.83027099134948e-176	Loop of Henle
B3GLCT	4.98554683894372e-180	0.753159127433836	0.279	0.149	1.44182014582252e-175	Loop of Henle
ABCC5	3.64379497601494e-179	0.48823998531321	0.435	0.266	1.05378550706352e-174	Loop of Henle
AP3B1	8.80030276339476e-179	0.471117668537358	0.691	0.516	2.54504755917377e-174	Loop of Henle
HMGA1P4	1.7411468817644e-178	0.702488162213486	0.273	0.144	5.03539678206264e-174	Loop of Henle
TRA2A	2.78726109917827e-177	0.500293701945138	0.555	0.385	8.06075909882357e-173	Loop of Henle
VAV3	4.77967052301885e-177	0.29848591179322	0.611	0.415	1.38228071525705e-172	Loop of Henle
PITPNM2	5.32083944931458e-177	0.540239128830874	0.392	0.236	1.53878676874178e-172	Loop of Henle

gene	p_val	avg_log2FC	pct.1	pct.2	p_val_adj	cluster
UST	6.43233358953312e-177	0.404209290371864	0.289	0.153	1.86023087409298e-172	Loop of Henle
EEA1	7.24184652514213e-177	0.634121507479505	0.443	0.286	2.0943420150711e-172	Loop of Henle
NINL	1.01226701920907e-176	0.408400414920848	0.6	0.414	2.92747621955262e-172	Loop of Henle
ZNF532	1.41969902997233e-176	0.614410030482291	0.364	0.215	4.10576959467999e-172	Loop of Henle
MNAT1	3.56229004765716e-176	0.48443213047958	0.604	0.43	1.03021428178245e-171	Loop of Henle
VWA8	4.85718020920452e-176	0.406126941224094	0.726	0.564	1.40469651650195e-171	Loop of Henle
ZNF680	3.87322161522629e-175	0.738043951317434	0.257	0.135	1.12013569112344e-170	Loop of Henle
ATG10	4.6394738982287e-175	0.609755945447869	0.451	0.292	1.34173585136774e-170	Loop of Henle
SPIRE1	7.23609256833981e-175	0.409379232429345	0.529	0.343	2.09267797076387e-170	Loop of Henle
TBC1D8	1.40438933488802e-174	0.351216848518569	0.459	0.29	4.06149395649616e-170	Loop of Henle
EPB41L1	5.99045119699346e-174	0.485654073728691	0.554	0.378	1.73243848617051e-169	Loop of Henle
ITGB5	6.45870944555642e-174	0.536429199874895	0.396	0.24	1.86785877165492e-169	Loop of Henle
DNAJC11	8.91678196090418e-174	0.531285808647806	0.435	0.272	2.57873334309349e-169	Loop of Henle
MITF	9.52959862123073e-174	0.275162214741727	0.618	0.418	2.75595992125993e-169	Loop of Henle
GRAMD2B	2.21066882699829e-173	0.485682982478879	0.514	0.347	6.39325424767906e-169	Loop of Henle
WARS2-AS1	1.23671107580679e-171	0.592842991194286	0.405	0.253	3.57656843123325e-167	Loop of Henle
NAA16	2.2801388533692e-171	0.637716921074726	0.353	0.209	6.59416156394373e-167	Loop of Henle
ZNF559	3.87543372831396e-171	0.743027777485287	0.269	0.144	1.1207754342284e-166	Loop of Henle
ZNF737	5.56570096227461e-171	0.649818841588227	0.27	0.144	1.60960071828982e-166	Loop of Henle
PIP5K1B	6.2933541746652e-171	0.422119967143966	0.328	0.187	1.82003802731318e-166	Loop of Henle
BCKDHB	9.12492903939853e-170	0.514653442784696	0.54	0.376	2.63892947819405e-165	Loop of Henle
SNX10	2.10257595173327e-169	0.49404866517969	0.379	0.225	6.08064965241261e-165	Loop of Henle
EPHX2	2.3779016448563e-169	0.381902105893026	0.421	0.255	6.87689155692442e-165	Loop of Henle
SCN7A	3.09248084957956e-169	0.921079667370351	0.376	0.239	8.94345461698409e-165	Loop of Henle
RASSF8	3.86295727530951e-169	0.528170662206258	0.49	0.327	1.11716724401951e-164	Loop of Henle
RBBP8	1.95379426048539e-168	0.355164818732442	0.496	0.322	5.65037300132373e-164	Loop of Henle
ACSL4	1.08930182335285e-167	0.599148852781431	0.452	0.292	3.15026087313644e-163	Loop of Henle
SCGB2B2	1.19830842399474e-166	0.736181995303947	0.289	0.16	3.46550796219279e-162	Loop of Henle
PDK4	2.21523881181919e-166	0.603524576438336	0.436	0.278	6.40647064378111e-162	Loop of Henle
ZMAT1	1.09900647901014e-165	0.362692679491097	0.598	0.413	3.17832673729731e-161	Loop of Henle
AC002070.1	8.35500117760333e-165	0.659229712210585	0.261	0.139	2.41626634056288e-160	Loop of Henle
SORL1	3.76193431202475e-164	0.576773959867593	0.315	0.18	1.08795140303756e-159	Loop of Henle
CCDC66	6.21979670646081e-164	0.526180926238532	0.392	0.242	1.79876520750847e-159	Loop of Henle
SORT1	7.62510661986663e-164	0.695998575975265	0.319	0.185	2.20518083446543e-159	Loop of Henle
ACSL3	5.51602864543786e-163	0.535684629063895	0.369	0.224	1.59523548426063e-158	Loop of Henle
LNPEP	7.37612171933992e-162	0.508835253200121	0.396	0.247	2.1331744012331e-157	Loop of Henle
SGO1-AS1	3.80398439375244e-161	0.651461094689378	0.512	0.359	1.1001122866732e-156	Loop of Henle
ARHGEF18	4.99930111774185e-161	0.557461633964301	0.335	0.197	1.44579788325094e-156	Loop of Henle
ITFG2	3.84325103411641e-160	0.581502362749039	0.333	0.196	1.11146819906647e-155	Loop of Henle
ARFGEF2	2.00101494673088e-159	0.467364305805053	0.524	0.359	5.78693522594572e-155	Loop of Henle
ATP6AP1L	2.24003682963333e-159	0.687318396	0.29	0.164	6.4781865112996e-155	Loop of Henle
TTC28-AS1	3.27742182711235e-159	0.688571284407004	0.299	0.171	9.47830392400891e-155	Loop of Henle

gene	p_val	avg_log2FC	pct.1	pct.2	p_val_adj	cluster
USP24	5.77782631807345e-159	0.3495022498661	0.602	0.426	1.67094737118684e-154	Loop of Henle
AC011346.1	9.75075987991855e-159	0.79528311908069	0.275	0.154	2.81991975727244e-154	Loop of Henle
FRK	3.56607388441265e-158	0.481319564501625	0.472	0.311	1.03130856737214e-153	Loop of Henle
IMMP1L	3.03855289688936e-157	0.541618291715186	0.381	0.238	8.78749497780402e-153	Loop of Henle
LMF1	8.21762392990231e-157	0.616065232385698	0.37	0.227	2.37653684052775e-152	Loop of Henle
AGBL4	3.44360665316842e-156	0.299850928328556	0.685	0.491	9.95891044096308e-152	Loop of Henle
LINC00882	5.15770818479618e-156	0.66467512490752	0.256	0.139	1.49160920704306e-151	Loop of Henle
VEPH1	9.21747751867016e-156	0.396574975276527	0.404	0.249	2.66569449839941e-151	Loop of Henle
AF117829.1	9.57765668916638e-156	0.471502664544002	0.557	0.392	2.76985831450692e-151	Loop of Henle
DAB1	2.92228801730781e-155	0.577070101826384	0.478	0.326	8.45125694605418e-151	Loop of Henle
DNM1L	3.11251786646926e-155	0.53380881740811	0.385	0.241	9.00140166982909e-151	Loop of Henle
MYSM1	5.57810238264972e-155	0.542224402858738	0.385	0.241	1.6131872090623e-150	Loop of Henle
CD24	8.2781005417043e-155	0.48498928624111	0.298	0.168	2.39402667666088e-150	Loop of Henle
SLC45A4	1.06031309380938e-154	0.314081835551742	0.37	0.223	3.06642546729671e-150	Loop of Henle
BDNF-AS	3.62650502038115e-154	0.635665399372009	0.323	0.191	1.04878525189423e-149	Loop of Henle
CEP95	6.46874619003197e-154	0.495283368429707	0.435	0.283	1.87076139815724e-149	Loop of Henle
SPIN1	7.94882431174929e-154	0.45727109449766	0.545	0.382	2.2987999909579e-149	Loop of Henle
AL356124.1	8.11058404348215e-154	0.383237594603824	0.313	0.181	2.34558090537504e-149	Loop of Henle
RNF10	1.06681387651403e-153	0.493907711552025	0.386	0.241	3.08522573087857e-149	Loop of Henle
AC084198.2	2.00285906561519e-153	0.57314759526999	0.326	0.194	5.79226841775912e-149	Loop of Henle
TTC3	2.37246813797369e-153	0.372263416949843	0.691	0.514	6.86117785501992e-149	Loop of Henle
FAM126B	6.12091088356675e-153	0.537587096885266	0.371	0.23	1.7701674275275e-148	Loop of Henle
EIF3K	3.12309095143122e-152	0.628187012414597	0.286	0.162	9.03197903153909e-148	Loop of Henle
ZNF518A	1.31812745946508e-151	0.445678385694364	0.568	0.405	3.81202461277302e-147	Loop of Henle
LINC02241	1.15718851536947e-150	0.741568270266802	0.301	0.178	3.34658918644851e-146	Loop of Henle
FLNB	7.05199130353962e-150	0.39907717427306	0.476	0.313	2.03943588498366e-145	Loop of Henle
SAMD12	1.11030102958577e-149	0.345013482371086	0.694	0.515	3.21099057756204e-145	Loop of Henle
GCFC2	1.19291605096285e-149	0.57409797078714	0.325	0.193	3.44991321938457e-145	Loop of Henle
C2CD3	2.26535340478373e-149	0.65866548876641	0.276	0.156	6.55140204663454e-145	Loop of Henle
NUP88	2.88697524108869e-149	0.600830634971435	0.345	0.209	8.3491323972285e-145	Loop of Henle
GNAS	6.35314555288522e-149	0.385635764965364	0.414	0.264	1.8373296938944e-144	Loop of Henle
HSD17B4	7.98409546026188e-149	0.595362564891757	0.332	0.201	2.30900040710774e-144	Loop of Henle
ASPH	1.27474620204953e-148	0.501214245025699	0.36	0.222	3.68656601632726e-144	Loop of Henle
LINC00571	1.91801716707083e-148	0.539223822501482	0.399	0.254	5.54690564716885e-144	Loop of Henle
MSH2	3.46600786852628e-148	0.685994943317051	0.27	0.152	1.0023694755778e-143	Loop of Henle
CCDC7	1.09671908987023e-146	0.481755461374605	0.466	0.314	3.17171160790472e-142	Loop of Henle
LINC01266	1.46791388533927e-146	0.718816951164591	0.329	0.201	4.24520695640118e-142	Loop of Henle
CTBP2	1.56976381816505e-146	0.421029938823279	0.615	0.453	4.53975696213334e-142	Loop of Henle
MAP3K3	1.63019614100825e-146	0.609658276466549	0.324	0.194	4.71452723979586e-142	Loop of Henle
TSPAN14	3.05603361639347e-146	0.516661215584937	0.269	0.148	8.83804921860993e-142	Loop of Henle
RHBDD1	6.58200054955048e-146	0.496270413756499	0.422	0.275	1.90351455893e-141	Loop of Henle
HCG17	8.93433864007315e-146	0.464432824167629	0.469	0.313	2.58381073470915e-141	Loop of Henle

gene	p_val	avg_log2FC	pct.1	pct.2	p_val_adj	cluster
NRSN2-AS1	9.10272603252441e-146	0.553726534263768	0.36	0.222	2.63250836860606e-141	Loop of Henle
SLC39A10	4.83150634697929e-145	0.620731478833525	0.275	0.156	1.39727163554641e-140	Loop of Henle
TSGA10	6.11526291004159e-145	0.428149127125742	0.484	0.325	1.76853403358403e-140	Loop of Henle
COL4A2	7.46901185154343e-145	0.258843961694877	0.522	0.349	2.16003822746636e-140	Loop of Henle
gene	p_val	avg_log2FC	pct.1	pct.2	p_val_adj	cluster
TFB1M	1.01862820013542e-144	0.651239781751616	0.263	0.147	2.94587275479164e-140	Loop of Henle
MPHOSPH9	2.66554618894613e-144	0.510692960600763	0.305	0.178	7.7087595784322e-140	Loop of Henle
AL589935.1	1.37634854368552e-143	0.593469999979518	0.423	0.282	3.98039998833854e-139	Loop of Henle
MTOR	1.98978447203446e-143	0.576978481601583	0.323	0.195	5.75445669312365e-139	Loop of Henle
ZNF808	2.6637842768453e-143	0.623807831289335	0.262	0.146	7.7036641286366e-139	Loop of Henle
CASC2	2.9545399486011e-143	0.669789973261834	0.297	0.175	8.54452953135438e-139	Loop of Henle
PANK1	2.96053061884084e-143	0.480539979094114	0.253	0.138	8.5618545496877e-139	Loop of Henle
LTN1	3.08005365640871e-143	0.618426253508883	0.319	0.191	8.90751517433399e-139	Loop of Henle
SPPL2A	6.22504071194477e-143	0.493238539429813	0.388	0.245	1.80028177389443e-138	Loop of Henle
RIC3	6.43093535269042e-143	0.620077855105188	0.257	0.143	1.85982650399807e-138	Loop of Henle
AC139887.2	1.54896385931552e-142	0.47153780199132	0.501	0.344	4.47960348114049e-138	Loop of Henle
MIRLET7BHG	1.58563921513845e-142	0.442913845798313	0.383	0.243	4.58566861018039e-138	Loop of Henle
AGL	1.68538694251409e-142	0.594868404391995	0.31	0.184	4.87413903775075e-138	Loop of Henle
SMIM8	3.32420268479705e-142	0.59671287084363	0.305	0.181	9.61359416443306e-138	Loop of Henle
SOX6	1.84377206916685e-141	0.333383907002045	0.767	0.605	5.33218882403052e-137	Loop of Henle
NDUFV3	7.67723475804883e-141	0.513157370008586	0.332	0.201	2.22025629202772e-136	Loop of Henle
MOK	1.26211055135961e-140	0.50922088275206	0.358	0.223	3.65002371453199e-136	Loop of Henle
CCDC50	7.78639195884671e-140	0.469819248145995	0.437	0.29	2.25182455449847e-135	Loop of Henle
LINC01004	1.93589478170986e-139	0.463570303934216	0.427	0.283	5.59860770870492e-135	Loop of Henle
BBS9	1.06195474942938e-138	0.386327784507835	0.674	0.517	3.07117313534978e-134	Loop of Henle
KIAA0232	1.17709477580159e-138	0.398576611786874	0.576	0.416	3.40415809161819e-134	Loop of Henle
ZNF717	1.50329054518706e-138	0.570561465782147	0.329	0.203	4.34751625668098e-134	Loop of Henle
WWP2	4.37450021168987e-138	0.449336677942156	0.482	0.329	1.26510546122071e-133	Loop of Henle
MKLN1	4.57341899962599e-138	0.345491680720576	0.696	0.536	1.32263277469184e-133	Loop of Henle
STX18-AS1	3.7718850078179e-137	0.545949190440695	0.351	0.22	1.09082914426094e-132	Loop of Henle
PDXK	4.09718916566579e-137	0.447773740064104	0.394	0.252	1.18490710671055e-132	Loop of Henle
RUNX1	4.94499550031009e-137	0.673430380329532	0.338	0.213	1.43009269868968e-132	Loop of Henle
AP002495.1	9.74515326653998e-137	0.534365213072231	0.343	0.214	2.81829832468336e-132	Loop of Henle
TVP23C	1.2485230590516e-136	0.464057761601222	0.387	0.249	3.61072868677721e-132	Loop of Henle
SLX4IP	4.23176829402674e-136	0.501715832071632	0.418	0.276	1.22382739063253e-131	Loop of Henle
FXR1	4.42767334724956e-136	0.423373999409737	0.442	0.296	1.28048313202457e-131	Loop of Henle
GABPB2	7.15725357189361e-136	0.551626360139933	0.328	0.201	2.06987773299163e-131	Loop of Henle
POGZ	7.57253699148032e-136	0.362397522305408	0.611	0.449	2.18997769793611e-131	Loop of Henle
MRPS25	5.49379385735152e-135	0.501456987541462	0.331	0.204	1.58880518354606e-130	Loop of Henle
MYO19	1.2037510845039e-134	0.548724732534044	0.319	0.193	3.48124813638528e-130	Loop of Henle
GOLGA8A	2.57012533509663e-134	0.524282679416562	0.257	0.145	7.43280246909946e-130	Loop of Henle
PPTC7	2.93884916492743e-134	0.593938851985154	0.288	0.171	8.49915178497011e-130	Loop of Henle

gene	p_val	avg_log2FC	pct.1	pct.2	p_val_adj	cluster
TENT2	3.21971576354915e-134	0.389032063606035	0.571	0.417	9.31141798818414e-130	Loop of Henle
KCNMB2	1.09393118241479e-133	0.344095320770159	0.287	0.167	3.16364897954357e-129	Loop of Henle
URI1	1.28923596811367e-133	0.51223780900817	0.383	0.249	3.72847041978472e-129	Loop of Henle
KIF16B	1.67191675552139e-133	0.419402097751745	0.555	0.4	4.83518325696787e-129	Loop of Henle
WDR27	1.84055097523345e-133	0.474918529672992	0.407	0.268	5.32287342037515e-129	Loop of Henle
MFF	2.32090748031794e-133	0.541007313782906	0.3	0.18	6.71206443307949e-129	Loop of Henle
CYB5A	3.38490435586165e-133	0.367146661210745	0.349	0.214	9.7891433971519e-129	Loop of Henle
MAN1A1	3.9840838324755e-133	0.438931299556776	0.519	0.367	1.15219704435192e-128	Loop of Henle
EIF1B-AS1	1.40272131288921e-132	0.491890008589396	0.335	0.205	4.05667003687559e-128	Loop of Henle
RAP1A	3.31663985344157e-132	0.379115456511635	0.437	0.292	9.59172245615303e-128	Loop of Henle
TMEM161B	6.40693982342036e-132	0.61673286396741	0.289	0.172	1.85288699693317e-127	Loop of Henle
ATP5F1C	7.54031949051067e-132	0.509258465714284	0.345	0.218	2.18066039665569e-127	Loop of Henle
LYPLAL1	1.52240151866294e-131	0.345604191708441	0.554	0.399	4.40278519197321e-127	Loop of Henle
SYTL2	2.05408210722739e-131	0.327742066038106	0.403	0.258	5.9404054541016e-127	Loop of Henle
AC046134.2	9.04478593308196e-131	0.515299181106693	0.368	0.236	2.6157520918473e-126	Loop of Henle
PCMTD1	1.1232885118457e-130	0.360631141252045	0.521	0.367	3.24855037625776e-126	Loop of Henle
AFTPH	1.50122945442376e-130	0.400178806861302	0.39	0.253	4.34155558219352e-126	Loop of Henle
AC079142.1	2.44235997311151e-130	0.53494357661122	0.482	0.342	7.06330504223849e-126	Loop of Henle
CDH16	2.61943681429094e-130	0.347994012975085	0.441	0.29	7.5754112669294e-126	Loop of Henle
ATAD1	7.53135238699361e-130	0.51539244526268	0.323	0.2	2.17806711031855e-125	Loop of Henle
6-Mar	1.62516195440074e-129	0.382620044358269	0.53	0.38	4.69996837212693e-125	Loop of Henle
LINC00174	2.9644869712858e-129	0.518499063893003	0.344	0.217	8.57329632095855e-125	Loop of Henle
RBMX	2.76720700067335e-128	0.441731532787403	0.406	0.267	8.00276264594733e-124	Loop of Henle
COA1	6.07202616667887e-128	0.378869118218453	0.53	0.377	1.75602996740353e-123	Loop of Henle
DDX17	1.85203480497646e-127	0.290724804229822	0.844	0.705	5.35608465599191e-123	Loop of Henle
SMARCAD1	4.6000521183756e-127	0.485512923538932	0.356	0.228	1.33033507263422e-122	Loop of Henle
MLLT3	7.59722770985755e-127	0.329614741383556	0.503	0.35	2.1971182536908e-122	Loop of Henle
AC104123.1	9.10769593023651e-127	0.688473594416606	0.252	0.145	2.6339456630244e-122	Loop of Henle
WDR78	1.63860560504032e-126	0.584740279864585	0.261	0.151	4.73884740977661e-122	Loop of Henle
CDKL5	5.3626593385189e-126	0.592852376685926	0.282	0.169	1.55088108069967e-121	Loop of Henle
TMCC1	7.52964256518675e-126	0.37036964789442	0.628	0.471	2.17757262985201e-121	Loop of Henle
CPT1A	7.78847024022711e-126	0.440805217877723	0.403	0.267	2.25242559347368e-121	Loop of Henle
MGAT4A	1.44238372845106e-125	0.265958779810532	0.326	0.197	4.17137374268046e-121	Loop of Henle
ITPKB	3.0093003800992e-125	0.376593755531714	0.29	0.169	8.70289669924688e-121	Loop of Henle
MLLT6	2.40288821738823e-124	0.503306562189958	0.317	0.196	6.94915272468677e-120	Loop of Henle
IQCH-AS1	2.61358021597414e-124	0.541778490679105	0.283	0.169	7.55847398459721e-120	Loop of Henle
CMC2	2.75326479280382e-124	0.533525667751193	0.291	0.176	7.96244178078865e-120	Loop of Henle
GNA13	5.38245135708815e-124	0.494561029402856	0.259	0.151	1.55660493246989e-119	Loop of Henle
NDUFS4	7.54733182571874e-124	0.4561626356041	0.373	0.244	2.18268836399786e-119	Loop of Henle
PPP1R13B	1.02099046214848e-123	0.33054897085082	0.433	0.287	2.95270441653339e-119	Loop of Henle
FIP1L1	1.22535163380141e-123	0.474246139115418	0.358	0.23	3.54371692495367e-119	Loop of Henle
GNPTAB	1.23316275409706e-123	0.644672215310611	0.256	0.149	3.5663066848487e-119	Loop of Henle

gene	p_val	avg_log2FC	pct.1	pct.2	p_val_adj	cluster
FGFR2	1.86965542888173e-122	0.522223928603947	0.309	0.19	5.40704350032596e-118	Loop of Henle
CFAP69	2.35862866363432e-122	0.422703068485749	0.316	0.195	6.82115409523046e-118	Loop of Henle
DYNC2LI1	5.98444936371466e-122	0.518554732336955	0.281	0.168	1.73070275598628e-117	Loop of Henle
RAB30-DT	8.43849973611157e-122	0.478930103340599	0.323	0.203	2.44041412368347e-117	Loop of Henle
SHLD2	2.80139929838676e-121	0.384896345660567	0.529	0.379	8.1016467709345e-117	Loop of Henle
DENND1B	3.15686663105647e-121	0.391563880966977	0.48	0.336	9.12965829701532e-117	Loop of Henle
LINC01578	7.37435093440005e-121	0.426900681908265	0.359	0.232	2.13266229022849e-116	Loop of Henle
LRCH1	1.30752532513509e-120	0.327271007481856	0.626	0.475	3.78136324029069e-116	Loop of Henle
KIDINS220	1.72297723466954e-120	0.40053570433736	0.472	0.328	4.98285016266431e-116	Loop of Henle
LINC00271	3.751892932676e-120	0.421406719350157	0.452	0.312	1.0850474361299e-115	Loop of Henle
MESD	9.75541273007652e-120	0.643809784304049	0.25	0.145	2.82126536153813e-115	Loop of Henle
CTDSPL	1.04748727122378e-119	0.257584307245626	0.557	0.391	3.02933318837918e-115	Loop of Henle
ARMC2	1.28358055547029e-119	0.626013214038372	0.254	0.149	3.71211496642007e-115	Loop of Henle
ANXA11	7.24183240085388e-119	0.397229786386722	0.379	0.249	2.09433793032694e-114	Loop of Henle
CAPS2	2.16493106471465e-118	0.586414185320896	0.302	0.189	6.26098063915478e-114	Loop of Henle
RFX3-AS1	9.87337947731547e-118	0.473138630930757	0.4	0.27	2.85538134483963e-113	Loop of Henle
STAM2	1.20447318712413e-117	0.496385255941722	0.332	0.212	3.48333645716299e-113	Loop of Henle
MIER1	2.66482946529744e-117	0.510433178312151	0.332	0.212	7.7066868136402e-113	Loop of Henle
IMMT	8.5766162469668e-117	0.445273128089094	0.313	0.196	2.4803574186228e-112	Loop of Henle
ACVR1B	9.4794907650055e-117	0.456864648713987	0.342	0.22	2.74146872923959e-112	Loop of Henle
MCCC1	1.25865101949994e-116	0.416794392980989	0.388	0.257	3.64001874839382e-112	Loop of Henle
INTS10	1.33222487455103e-116	0.409826797809178	0.301	0.185	3.85279433720156e-112	Loop of Henle
PREPL	5.27164803342488e-116	0.403679533857768	0.402	0.272	1.52456061126648e-111	Loop of Henle
GIGYF2	9.93377275607803e-116	0.341783781577999	0.611	0.461	2.87284708105777e-111	Loop of Henle
SIK3	2.66693986953084e-115	0.27310100169312	0.789	0.648	7.71279010268318e-111	Loop of Henle
SRSF1	2.79601803491012e-115	0.503540591057774	0.294	0.181	8.08608415696006e-111	Loop of Henle
IMPA2	2.11472745806541e-114	0.484356145085432	0.294	0.181	6.11579180872518e-110	Loop of Henle
PHLDB2	4.79602020609287e-114	0.321813139701391	0.641	0.488	1.38700904360206e-109	Loop of Henle
METTL25	6.23950259757517e-114	0.500949553998124	0.343	0.222	1.80446415121874e-109	Loop of Henle
RRBP1	2.58464077358522e-113	0.385167586407086	0.449	0.308	7.47478111720845e-109	Loop of Henle
CPSF6	3.07969945230591e-113	0.406096631295474	0.389	0.26	8.90649081606869e-109	Loop of Henle
TNIP1	3.39267601752265e-113	0.502472979999252	0.251	0.148	9.81161904267551e-109	Loop of Henle
CLEC16A	5.76894850761002e-113	0.358258192984576	0.502	0.356	1.66837990840082e-108	Loop of Henle
CEP192	6.17858560841744e-113	0.373772950639423	0.41	0.276	1.78684695795432e-108	Loop of Henle
SUCLA2	7.62764496093801e-113	0.442675476196441	0.33	0.211	2.20591492270327e-108	Loop of Henle
HADHB	1.56692862380461e-111	0.531067323690299	0.283	0.175	4.53155758004294e-107	Loop of Henle
PRKAR1B	1.5964869474691e-111	0.562009838209647	0.273	0.167	4.61704025208065e-107	Loop of Henle
ZNF655	2.14011882667111e-111	0.353393220388469	0.391	0.263	6.18922364673285e-107	Loop of Henle
DSTN	2.37286429169139e-111	0.458292888376509	0.304	0.19	6.8623235315715e-107	Loop of Henle
USO1	9.60714337725816e-111	0.450641504290708	0.369	0.246	2.77838586470306e-106	Loop of Henle
ABHD12	1.06851662927181e-110	0.5260243244548	0.252	0.149	3.09015009185407e-106	Loop of Henle
RAB28	1.20489458685458e-110	0.357786561678416	0.519	0.375	3.48455514518345e-106	Loop of Henle

gene	p_val	avg_log2FC	pct.1	pct.2	p_val_adj	cluster
PHF8	1.5864485854596e-110	0.552503122475795	0.255	0.153	4.58800930914916e-106	Loop of Henle
HEATR5A	2.40473646423247e-110	0.484429474803984	0.313	0.199	6.95449785456031e-106	Loop of Henle
AMFR	1.02649861332598e-109	0.486473560219447	0.297	0.186	2.96863398973872e-105	Loop of Henle
SF1	1.09721830836871e-109	0.356756551166069	0.365	0.242	3.17315534780232e-105	Loop of Henle
GRAMD1C	3.15244642494909e-109	0.398129931766122	0.342	0.222	9.11687506095278e-105	Loop of Henle
SDC4	3.54921161141042e-109	0.386739451196721	0.289	0.178	1.02643199801989e-104	Loop of Henle
LINC01184	3.55933002629477e-109	0.449977464344626	0.366	0.243	1.02935824360445e-104	Loop of Henle
MIR4435-2HG	4.64612426220942e-109	0.58344518311218	0.282	0.177	1.34365913663096e-104	Loop of Henle
MTERF1	5.11525733362422e-109	0.470817908558046	0.374	0.252	1.47933242088413e-104	Loop of Henle
OXNAD1	5.38855695095291e-109	0.373973377641358	0.375	0.25	1.55837067021558e-104	Loop of Henle
BCL2	9.83658078227873e-109	0.33328228735826	0.566	0.427	2.84473916223501e-104	Loop of Henle
ZNF420	2.74127839281302e-108	0.602710312423127	0.268	0.166	7.92777711201527e-104	Loop of Henle
PAPOLA	2.90744779981514e-108	0.343171676660264	0.433	0.301	8.40833903706537e-104	Loop of Henle
PLOD2	3.29885545937834e-108	0.447970416293721	0.27	0.165	9.54028998852215e-104	Loop of Henle
BCR	1.60137340744195e-107	0.368434882620686	0.408	0.275	4.63117189432211e-103	Loop of Henle
NCOA2	1.70693807779692e-107	0.359123905785641	0.683	0.549	4.9364649209887e-103	Loop of Henle
TMEM120B	2.64339384244097e-107	0.553165492776386	0.323	0.211	7.64469499233929e-103	Loop of Henle
IFT74	9.7962176853419e-107	0.480181340567005	0.328	0.213	2.83306615460088e-102	Loop of Henle
PIGN	2.50621566443525e-106	0.436125967599725	0.352	0.234	7.24797570154674e-102	Loop of Henle
SS18	3.29972101697137e-106	0.406747736456528	0.326	0.212	9.5427931810812e-102	Loop of Henle
NR6A1	3.30193033406013e-106	0.341676705182768	0.521	0.376	9.5491825261019e-102	Loop of Henle
FUS	1.35810984835296e-105	0.296202086922446	0.445	0.31	3.92765368143677e-101	Loop of Henle
MRRF	3.31676482540696e-105	0.461373801267965	0.283	0.177	9.59208387507693e-101	Loop of Henle
SETDB2	3.98570558662359e-105	0.459785542659562	0.292	0.183	1.15266605565154e-100	Loop of Henle
GSPT1	4.94591635578323e-105	0.447370354501875	0.325	0.211	1.43035901009251e-100	Loop of Henle
RMDN1	5.05067651615711e-105	0.444479478578101	0.281	0.175	1.46065564847264e-100	Loop of Henle
TRMT11	1.20756013450649e-104	0.279481781176505	0.522	0.378	3.49226390899276e-100	Loop of Henle
CTNNAL1	2.718893443867e-104	0.517779330164764	0.255	0.155	7.86303983966338e-100	Loop of Henle
ARFGAP3	6.28330604226389e-104	0.535278427656018	0.263	0.162	1.81713210742272e-99	Loop of Henle
ATP5F1A	1.14553149514933e-103	0.393505925633304	0.281	0.174	3.31287708397187e-99	Loop of Henle
MPP7	1.47241351560715e-103	0.342943756585656	0.331	0.213	4.25821988713586e-99	Loop of Henle
KANSL3	1.4839155587863e-103	0.377185118214414	0.347	0.228	4.29148379600999e-99	Loop of Henle
ANAPC16	2.98224496710473e-103	0.365307956729502	0.384	0.261	8.62465244486688e-99	Loop of Henle
CLCN3	1.24692295924002e-102	0.365321086146267	0.337	0.221	3.60610119812213e-98	Loop of Henle
SLC25A27	1.93989740074012e-102	0.412353039511722	0.335	0.219	5.61018328294044e-98	Loop of Henle
ANKRD17	4.87445891943917e-102	0.29054107726042	0.716	0.58	1.40969351950181e-97	Loop of Henle
UBQLN1	6.56560860635904e-102	0.4823798213742	0.317	0.207	1.89877400895903e-97	Loop of Henle
SFPQ	1.68955637537705e-101	0.300580174499723	0.561	0.417	4.88619703759042e-97	Loop of Henle
GTF2IRD2B	2.23543145677061e-101	0.465543155052942	0.283	0.178	6.46486777298061e-97	Loop of Henle
CSRNP3	7.29878560070062e-101	0.459894062022479	0.306	0.198	2.11080879572262e-96	Loop of Henle
HNRNPA3	1.6311527766753e-100	0.346612008446538	0.43	0.3	4.71729383014498e-96	Loop of Henle
PKM	1.8382274324897e-100	0.401755171590534	0.317	0.204	5.31615373476022e-96	Loop of Henle

gene	p_val	avg_log2FC	pct.1	pct.2	p_val_adj	cluster
MIR646HG	5.41338864218475e-100	0.547599158898724	0.314	0.209	1.56555199531983e-95	Loop of Henle
LINC01278	6.10261232737369e-100	0.324043930267524	0.618	0.479	1.76487548507647e-95	Loop of Henle
SENP6	6.16629218302305e-100	0.290054649486106	0.65	0.503	1.78329169933027e-95	Loop of Henle
HNRNPD	1.0924833622706e-99	0.430350242088761	0.379	0.261	3.15946188368657e-95	Loop of Henle
ZNF544	1.15579164943489e-99	0.382662792811315	0.356	0.239	3.34254945016571e-95	Loop of Henle
ENSA	1.86259376191368e-99	0.390404345683422	0.385	0.264	5.38662115945437e-95	Loop of Henle
CAAP1	4.03161404967635e-99	0.434499167591374	0.347	0.233	1.1659427831664e-94	Loop of Henle
CPEB2	4.31309949145902e-99	0.349759465841719	0.399	0.274	1.24734837292995e-94	Loop of Henle
FAM135A	1.79059095026245e-98	0.293624148350161	0.463	0.329	5.17838902815901e-94	Loop of Henle
AL008633.1	2.38978113954069e-98	0.62020198944379	0.338	0.232	6.91124705555167e-94	Loop of Henle
SSBP3	2.43501785507796e-98	0.343032803723257	0.406	0.282	7.04207163688546e-94	Loop of Henle
NCOA1	2.65248476209661e-98	0.28422311441438	0.684	0.544	7.67098593198339e-94	Loop of Henle
THUMPD3-AS1	3.37242750603046e-98	0.45035285450593	0.27	0.17	9.75306034744009e-94	Loop of Henle
SHPRH	6.85279893919896e-98	0.282649949665045	0.55	0.409	1.98182945321634e-93	Loop of Henle
SGMS1	1.64391432541253e-97	0.307011724367766	0.529	0.39	4.75420022909303e-93	Loop of Henle
PRKCI	1.68336095625261e-97	0.409496669366904	0.332	0.219	4.86827988548256e-93	Loop of Henle
ATP6V0E1	4.34043937615296e-97	0.478675972301899	0.31	0.203	1.25525506758343e-92	Loop of Henle
PPA2	2.09969722295991e-96	0.350630147021581	0.413	0.289	6.07232436880006e-92	Loop of Henle
RAB3IP	2.42171511397747e-96	0.296246942781921	0.528	0.387	7.00360010962283e-92	Loop of Henle
UBA6-AS1	2.6090890793747e-96	0.366571212502978	0.501	0.369	7.54548561755164e-92	Loop of Henle
TNRC6B	3.05409214752797e-96	0.251010049736831	0.793	0.666	8.8324344906509e-92	Loop of Henle
STAT3	3.88919902579876e-96	0.316875727099472	0.539	0.403	1.124756358261e-91	Loop of Henle
PTAR1	6.9491837645962e-96	0.33896459309038	0.354	0.239	2.00970394472122e-91	Loop of Henle
ADIPOR2	8.47700558805238e-96	0.331310122245221	0.342	0.228	2.45155001606475e-91	Loop of Henle
IRAK1BP1	1.88239474024491e-95	0.418945594673663	0.304	0.197	5.44388558878827e-91	Loop of Henle
XPO1	4.16920473305198e-95	0.271421313900083	0.525	0.386	1.20573400879863e-90	Loop of Henle
UBE3D	6.70302619207194e-95	0.507701074560213	0.304	0.199	1.9385151747472e-90	Loop of Henle
AFG3L2	6.71784728704489e-95	0.367964830442396	0.337	0.225	1.94280143541338e-90	Loop of Henle
BABAM2	9.70778144803299e-95	0.254980869216131	0.754	0.611	2.80749039477114e-90	Loop of Henle
GOSR1	2.84477081609559e-94	0.364168123677761	0.308	0.201	8.22707720014844e-90	Loop of Henle
HMG20A	3.07905446769696e-94	0.428399602453023	0.288	0.186	8.9046255205796e-90	Loop of Henle
THSD7A	7.95524137296033e-94	0.277177396808592	0.723	0.59	2.30065580506013e-89	Loop of Henle
PRRG1	1.05320045336994e-93	0.481512650224085	0.261	0.164	3.04585571114587e-89	Loop of Henle
AC011586.2	1.09672707661914e-93	0.516959765015489	0.259	0.162	3.17173470558256e-89	Loop of Henle
AL158152.1	2.36130422120396e-93	0.424231128046875	0.286	0.184	6.82889180772187e-89	Loop of Henle
MIPEP	2.55326617906078e-93	0.469955643664954	0.312	0.206	7.38404578984377e-89	Loop of Henle
USP9Y	2.91097465017662e-93	0.519758811657261	0.322	0.216	8.41853868831078e-89	Loop of Henle
TASOR	4.48413410815845e-93	0.336039804652292	0.373	0.257	1.29681158407942e-88	Loop of Henle
STAT5B	1.12219345758985e-92	0.359539018307582	0.358	0.245	3.24538347934986e-88	Loop of Henle
KLHDC10	1.20264033037007e-92	0.359967138672022	0.374	0.257	3.47803583543024e-88	Loop of Henle
MRPS31	1.28372285829274e-92	0.393554286245968	0.313	0.206	3.71252650618259e-88	Loop of Henle
FASTKD1	1.72964968786281e-92	0.447722677966091	0.284	0.182	5.00214689729926e-88	Loop of Henle

gene	p_val	avg_log2FC	pct.1	pct.2	p_val_adj	cluster
KAT2B	2.3531208672165e-92	0.40158981932671	0.266	0.168	6.80522554799011e-88	Loop of Henle
BRAF	4.52811878618866e-92	0.259709085991741	0.681	0.54	1.30953195296576e-87	Loop of Henle
UBXN2A	9.63979260718051e-92	0.39405233195036	0.266	0.167	2.7878280219966e-87	Loop of Henle
PNPLA8	1.78646314274883e-91	0.345279581242224	0.298	0.194	5.16645140882961e-87	Loop of Henle
BRWD1	1.92465106881567e-91	0.26396445636643	0.623	0.479	5.56609089101492e-87	Loop of Henle
ZMYM6	3.25310777360875e-91	0.389227489829488	0.297	0.194	9.40798768127651e-87	Loop of Henle
DNM2	5.58701504595709e-91	0.264855816136121	0.56	0.416	1.61576475129079e-86	Loop of Henle
EFCAB6	1.61075500005386e-90	0.408004818645648	0.329	0.221	4.65830346015575e-86	Loop of Henle
NEK1	1.61130555213182e-90	0.271210735247037	0.526	0.389	4.65989565676523e-86	Loop of Henle
FAM172A	1.84965630236404e-90	0.272581930730518	0.644	0.507	5.34920602643679e-86	Loop of Henle
ELL2	2.99427634224039e-90	0.268903346292655	0.384	0.265	8.65944718175921e-86	Loop of Henle
ICE1	3.13670243747898e-90	0.339626785344948	0.381	0.265	9.07134344918921e-86	Loop of Henle
ZNF875	3.33475260967209e-90	0.444419658386216	0.259	0.164	9.64410454717169e-86	Loop of Henle
TMEM65	7.15128478011838e-90	0.377522839630948	0.291	0.189	2.06815155841023e-85	Loop of Henle
FEZ2	8.05730853289963e-90	0.360746652327359	0.333	0.224	2.33017362771457e-85	Loop of Henle
SDHB	9.54220242262622e-90	0.437907865414832	0.256	0.161	2.7596049406235e-85	Loop of Henle
YWHAQ	2.34258831867127e-89	0.392302918102475	0.271	0.174	6.77476541759732e-85	Loop of Henle
ZNF277	3.04839740986363e-89	0.353742368747847	0.386	0.27	8.81596530932563e-85	Loop of Henle
SUPT3H	3.23818933416034e-89	0.346144724851355	0.513	0.384	9.3648435543917e-85	Loop of Henle
PCM1	3.33419470105868e-88	0.258276686093445	0.675	0.53	9.6424910754617e-84	Loop of Henle
GTF2IRD1	3.69981554873754e-88	0.272783278849084	0.47	0.337	1.0699866566949e-83	Loop of Henle
SMG6	3.94447266811147e-88	0.283142147766658	0.529	0.395	1.14074149561784e-83	Loop of Henle
SENP7	5.02603993482025e-88	0.315810949293506	0.428	0.305	1.45353074915002e-83	Loop of Henle
ASCC3	5.97415300487586e-88	0.254887947804603	0.458	0.328	1.7277250490101e-83	Loop of Henle
GOLPH3	5.99632304732067e-88	0.336147583692789	0.336	0.228	1.73413662528514e-83	Loop of Henle
CUL1	7.06219737871622e-88	0.353121621514627	0.359	0.248	2.04238748192473e-83	Loop of Henle
ZBTB44	1.31679417385023e-87	0.376314323144094	0.318	0.213	3.80816875077486e-83	Loop of Henle
CASD1	2.7431045378067e-87	0.448140295205524	0.252	0.16	7.93305832333697e-83	Loop of Henle
FAAH2	3.72103670310097e-87	0.260126799371941	0.487	0.353	1.0761238145368e-82	Loop of Henle
SOS2	4.97692256038042e-87	0.28813386560782	0.528	0.397	1.43932600446202e-82	Loop of Henle
NUCKS1	1.02546867877009e-86	0.279951471602862	0.423	0.301	2.96565541900309e-82	Loop of Henle
NDUFS1	1.77121926674957e-86	0.33297679396336	0.384	0.268	5.12236611943975e-82	Loop of Henle
CAMKMT	4.22735707129748e-86	0.300673383074313	0.628	0.496	1.22255166501923e-81	Loop of Henle
UBXN4	4.70216237959304e-86	0.265334135162041	0.445	0.32	1.35986536017831e-81	Loop of Henle
ARFIP1	5.3263699641457e-86	0.301619073831252	0.413	0.293	1.54038619363094e-81	Loop of Henle
AP2B1	7.53289103296003e-86	0.342294143579644	0.383	0.271	2.17851208673204e-81	Loop of Henle
PDHX	1.56218644344684e-85	0.352809748823979	0.31	0.207	4.51784319444827e-81	Loop of Henle
CCDC18-AS1	2.01552051728333e-85	0.27419879307548	0.505	0.376	5.8288853359834e-81	Loop of Henle
CHP1	7.9454847771589e-85	0.763839333687938	0.277	0.188	2.29783419755435e-80	Loop of Henle
UHRF2	8.4882472567816e-85	0.338794587328646	0.313	0.21	2.45480110666124e-80	Loop of Henle
COG6	1.06979775274153e-84	0.364869831627478	0.322	0.218	3.09385510092851e-80	Loop of Henle
USP54	1.97355198023632e-84	0.486304687645113	0.271	0.178	5.70751232684342e-80	Loop of Henle

gene	p_val	avg_log2FC	pct.1	pct.2	p_val_adj	cluster
SACM1L	2.46880638146023e-84	0.374312073964769	0.277	0.181	7.13978805518299e-80	Loop of Henle
ZSCAN5A	3.23765160173039e-84	0.44604422065164	0.33	0.226	9.3632884322043e-80	Loop of Henle
VGLL4	6.87199606968753e-84	0.253720970104097	0.518	0.389	1.98738126335363e-79	Loop of Henle
DPH6-DT	9.92947511934075e-84	0.357849735678159	0.448	0.328	2.87160420451335e-79	Loop of Henle
MIPOL1	9.97961816358847e-84	0.364244236709967	0.409	0.294	2.88610557290979e-79	Loop of Henle
CHD6	1.32251948006869e-83	0.276085579490261	0.545	0.413	3.82472633635865e-79	Loop of Henle
SMAD4	5.2054652580301e-83	0.359093037705346	0.329	0.224	1.50542055262231e-78	Loop of Henle
KIF27	8.24610402629377e-83	0.326956903156329	0.292	0.193	2.38477328440416e-78	Loop of Henle
IP6K1	8.64726021200047e-83	0.365603758977805	0.306	0.206	2.50078765331054e-78	Loop of Henle
MTDH	1.44908881488368e-82	0.274712847476006	0.454	0.33	4.19076485264361e-78	Loop of Henle
YTHDF3	4.21350910681677e-82	0.345298794956249	0.265	0.172	1.21854683369141e-77	Loop of Henle
ZNF586	4.52776916437081e-82	0.342235361527959	0.353	0.245	1.30943084233604e-77	Loop of Henle
SDHA	6.31775968842392e-82	0.317501018114167	0.317	0.215	1.8270961018922e-77	Loop of Henle
ZC3H6	7.08436108438204e-82	0.368196953208285	0.288	0.191	2.04879722560329e-77	Loop of Henle
SEM1	7.63482389140789e-82	0.501576788472488	0.267	0.175	2.20799106939516e-77	Loop of Henle
HSDL2	1.10510696519574e-81	0.289337990895372	0.35	0.24	3.19596934334609e-77	Loop of Henle
ACYP2	2.89400290319847e-81	0.260459397416492	0.529	0.402	8.36945639604996e-77	Loop of Henle
PIK3C3	2.71678432144885e-80	0.306274625083713	0.368	0.258	7.85694025763006e-76	Loop of Henle
TOM1L1	8.60697060669738e-80	0.28768813888126	0.408	0.293	2.48913589945688e-75	Loop of Henle
MRPS5	8.6164164666991e-80	0.36241833093387	0.345	0.238	2.49186764216938e-75	Loop of Henle
SCAF8	1.16191000922779e-79	0.267247387853062	0.523	0.393	3.36024374668678e-75	Loop of Henle
VAPA	1.49995826906825e-79	0.273093998376274	0.422	0.303	4.33787931414537e-75	Loop of Henle
HMGB1	2.09658035697777e-79	0.269874328518715	0.522	0.393	6.0633103923797e-75	Loop of Henle
PLXNB1	2.77077055859661e-79	0.260718886731294	0.379	0.266	8.01306845546141e-75	Loop of Henle
HULC	2.81845459734851e-79	0.455468043685947	0.37	0.266	8.15097069553188e-75	Loop of Henle
NPHP3	4.86481710219506e-79	0.348081836339062	0.353	0.246	1.40690510595481e-74	Loop of Henle
SLC49A4	5.67820997229216e-79	0.297675248935453	0.252	0.162	1.64213832398689e-74	Loop of Henle
ZNF254	6.70924207212542e-79	0.345868610210459	0.349	0.244	1.94031280725867e-74	Loop of Henle
TAX1BP1	1.55702565089651e-78	0.252090919345062	0.53	0.401	4.50291818239269e-74	Loop of Henle
TTC19	1.74183932278139e-78	0.305091744915812	0.319	0.218	5.03739932148377e-74	Loop of Henle
UMAD1	1.80590561764475e-78	0.268758413799947	0.532	0.405	5.22267904622863e-74	Loop of Henle
EMC2	5.75364340702865e-78	0.368891460670775	0.259	0.17	1.66395367331268e-73	Loop of Henle
WNK1	2.55120861247191e-77	0.398404913891673	0.672	0.564	7.37809530726877e-73	Loop of Henle
DAZAP1	5.7556331602268e-77	0.311224632610066	0.307	0.208	1.66452910993759e-72	Loop of Henle
TFG	6.00948053640143e-77	0.32756383823332	0.3	0.202	1.73794177112729e-72	Loop of Henle
ZNF506	7.47903531763149e-77	0.347461731250256	0.302	0.206	2.16293701385903e-72	Loop of Henle
MACROD2	1.26241368523775e-76	0.279468980489104	0.665	0.546	3.65090037770758e-72	Loop of Henle
R3HDM1	2.24073919032327e-76	0.286438680037789	0.403	0.291	6.4802177384149e-72	Loop of Henle
EEF1AKMT2	3.05869982820943e-76	0.405650364804205	0.253	0.165	8.84575990318168e-72	Loop of Henle
ERCC8	1.35310254481417e-75	0.313262153283921	0.301	0.205	3.91317255960259e-71	Loop of Henle
MIB1	3.92815368990842e-74	0.250061837149526	0.413	0.299	1.13602204712152e-69	Loop of Henle
TMEM260	7.99409352077725e-74	0.255599208287266	0.336	0.233	2.31189184620878e-69	Loop of Henle

gene	p_val	avg_log2FC	pct.1	pct.2	p_val_adj	cluster
U2SURP	9.8790252820145e-74	0.277704222144182	0.409	0.297	2.85701411155859e-69	Loop of Henle
TNPO1	1.13313212988867e-73	0.282795040127169	0.394	0.284	3.27701811963803e-69	Loop of Henle
GPR39	2.20396359122787e-73	0.442090626027294	0.284	0.193	6.37386270583101e-69	Loop of Henle
ICE2	4.09367113164686e-73	0.318739870415234	0.29	0.196	1.18388969127227e-68	Loop of Henle
ORC2	4.88626024444399e-73	0.296501774645018	0.294	0.2	1.4131064626932e-68	Loop of Henle
STX18	7.67591545565465e-73	0.256959574645647	0.406	0.295	2.21987474977533e-68	Loop of Henle
COQ4	1.27800073458566e-72	0.252739548454706	0.312	0.213	3.69597812442172e-68	Loop of Henle
ADNP	1.40674896553423e-72	0.25113833368937	0.483	0.364	4.06831800832499e-68	Loop of Henle
KLHL24	1.47110542666407e-72	0.349823343931489	0.332	0.234	4.25443689391248e-68	Loop of Henle
ZNF611	1.47125519549312e-72	0.298224082606872	0.332	0.23	4.25487002536611e-68	Loop of Henle
ATP2A2	1.93739745220194e-72	0.278208553078379	0.356	0.251	5.60295343176801e-68	Loop of Henle
HOOK1	4.43873851469722e-72	0.347653814469867	0.274	0.184	1.28368317845044e-67	Loop of Henle
AFG1L	6.79643875829548e-72	0.279890712395332	0.355	0.252	1.96553008889905e-67	Loop of Henle
TULP3	1.59445028145221e-71	0.300758926233509	0.296	0.202	4.61115021395979e-67	Loop of Henle
MYH10	3.41048326930722e-71	0.294747543939538	0.404	0.295	9.86311761483649e-67	Loop of Henle
NAA35	3.8846299221362e-71	0.296510235940091	0.371	0.267	1.12343497348179e-66	Loop of Henle
UTY	4.18680043271166e-71	0.333035245033424	0.351	0.248	1.21082268514021e-66	Loop of Henle
HNRNPDL	5.16273708845361e-71	0.291676469259419	0.45	0.336	1.49306356598078e-66	Loop of Henle
KCTD3	6.11828527952981e-71	0.254519651726702	0.298	0.204	1.76940810284002e-66	Loop of Henle
VPS45	9.18503945361802e-71	0.302399320797953	0.329	0.231	2.65631340998633e-66	Loop of Henle
ST7L	2.42380504983411e-70	0.377621733731336	0.269	0.182	7.00964420412024e-66	Loop of Henle
SECISBP2L	6.84597394942921e-70	0.265999377219099	0.409	0.3	1.97985566617493e-65	Loop of Henle
TMEM14B	7.50598489872413e-70	0.275644658231994	0.388	0.282	2.17073083271102e-65	Loop of Henle
IFT80	1.55690318689801e-69	0.329092326335424	0.272	0.183	4.50256401650903e-65	Loop of Henle
ZBTB1	2.57024651812484e-69	0.322578312070047	0.293	0.201	7.43315293041704e-65	Loop of Henle
ZNF280D	3.33692873232372e-69	0.252441053672268	0.409	0.3	9.65039789388021e-65	Loop of Henle
ZDHHC21	3.48479293580194e-69	0.266910274412894	0.402	0.293	1.00780211703392e-64	Loop of Henle
OPA1	5.66562751564024e-68	0.255168306310538	0.374	0.27	1.63849947752316e-63	Loop of Henle
SLC44A3	7.61903360625752e-68	0.301537948141759	0.306	0.211	2.20342451892968e-63	Loop of Henle
TBCA	1.10053231902323e-67	0.277331014939336	0.347	0.248	3.18273946661517e-63	Loop of Henle
ETFA	1.3207865988402e-67	0.290242683746373	0.321	0.226	3.81971484384586e-63	Loop of Henle
CASZ1	1.47975260193224e-67	0.260017729808697	0.36	0.254	4.27944452478804e-63	Loop of Henle
CAPRIN1	1.60549667164664e-67	0.292605345377877	0.271	0.183	4.6430963744021e-63	Loop of Henle
AC005670.3	2.84389344219585e-67	0.29235296740118	0.274	0.186	8.22453983483039e-63	Loop of Henle
TNRC6C	8.64937087019257e-67	0.277269409387446	0.331	0.236	2.50139805565969e-62	Loop of Henle
SLAIN2	1.08063744223118e-66	0.333407007225109	0.286	0.196	3.12520348293258e-62	Loop of Henle
LATS1	1.20705850786329e-66	0.266292965766128	0.288	0.198	3.49081320474064e-62	Loop of Henle
ZNF146	3.09863872400347e-66	0.29454914555633	0.252	0.169	8.96126318981804e-62	Loop of Henle
MBTPS1	6.32441402972536e-66	0.335446847947609	0.253	0.17	1.82902053739658e-61	Loop of Henle
FLG-AS1	7.13590965046257e-66	0.335857125120274	0.293	0.203	2.06370507091378e-61	Loop of Henle
ARMC8	1.05946427010884e-65	0.276546043697692	0.352	0.255	3.06397066915476e-61	Loop of Henle
BICRAL	1.20467679907685e-65	0.254514744017348	0.337	0.24	3.48392530293024e-61	Loop of Henle

gene	p_val	avg_log2FC	pct.1	pct.2	p_val_adj	cluster
LARS2	1.76817780106334e-65	0.379695307496204	0.264	0.18	5.11357020067518e-61	Loop of Henle
SPAG16	5.12572680158463e-65	0.280237597624299	0.466	0.354	1.48236019101828e-60	Loop of Henle
SMARCA4	6.22502490107035e-65	0.284615116205227	0.37	0.27	1.80027720138955e-60	Loop of Henle
TTN-AS1	6.30170331854133e-65	0.288343407065223	0.418	0.316	1.82245259972215e-60	Loop of Henle
COG7	7.38056695104569e-65	0.351536105923075	0.264	0.18	2.13445996224241e-60	Loop of Henle
TP53BP1	1.36948095027546e-64	0.271192225145013	0.402	0.298	3.96053890819664e-60	Loop of Henle
S100PBP	2.71793940911796e-64	0.33834705528159	0.278	0.192	7.86028077116914e-60	Loop of Henle
RBM41	3.3218657524256e-64	0.277647600416104	0.312	0.219	9.60683575601483e-60	Loop of Henle
TAPT1	6.92422463927061e-64	0.306906866163452	0.279	0.193	2.00248576567706e-59	Loop of Henle
ZNF248	8.80756364772085e-64	0.299732069296655	0.331	0.238	2.54714740692087e-59	Loop of Henle
ZNF10	9.48856684528064e-64	0.270963953441481	0.309	0.218	2.74409353165516e-59	Loop of Henle
XPO4	1.43056869298094e-63	0.267747208844125	0.338	0.243	4.13720466010089e-59	Loop of Henle
HLCS	1.90108412260186e-63	0.311199516303289	0.387	0.287	5.49793528256459e-59	Loop of Henle
BMPR1A	4.40137163233036e-63	0.252419193149201	0.41	0.305	1.27287667606994e-58	Loop of Henle
HADHA	7.65397139671218e-63	0.263592065298518	0.288	0.2	2.21352852792916e-58	Loop of Henle
RYBP	1.04903421736193e-62	0.252038090711289	0.285	0.198	3.03380695661071e-58	Loop of Henle
KIFAP3	2.39575763897559e-62	0.292895172029248	0.272	0.187	6.9285310919174e-58	Loop of Henle
CSTF3	4.68876281098144e-62	0.360326428208139	0.251	0.172	1.35599020493583e-57	Loop of Henle
CBFB	5.23964635788901e-62	0.254789162762911	0.347	0.25	1.5153057267015e-57	Loop of Henle
gene	p_val	avg_log2FC	pct.1	pct.2	p_val_adj	cluster
PPM1B	2.02713773631716e-61	0.257549438956095	0.367	0.268	5.86248233342922e-57	Loop of Henle
SLC35D1	4.48599305911009e-61	0.309949070418837	0.253	0.171	1.29734919269464e-56	Loop of Henle
SUPT20H	1.43138782701537e-60	0.258448320488936	0.296	0.209	4.13957359572846e-56	Loop of Henle
ACVR2A	2.40492850218973e-60	0.264033726998023	0.276	0.192	6.95505322833269e-56	Loop of Henle
SEC22A	3.19107714598052e-60	0.271580103891148	0.279	0.194	9.22859510617565e-56	Loop of Henle
ALCAM	6.91402286271812e-60	0.309837966173662	0.355	0.26	1.99953541189808e-55	Loop of Henle
CHM	9.13971069024406e-60	0.294284852850227	0.291	0.206	2.64320433161858e-55	Loop of Henle
TERF2IP	1.52689660058006e-59	0.315841422201403	0.266	0.185	4.41578496887752e-55	Loop of Henle
KDM7A	1.99183611887673e-59	0.251267195151489	0.273	0.189	5.7603900557915e-55	Loop of Henle
RHOT1	1.29017283883794e-58	0.263605434169498	0.298	0.211	3.73117984991933e-54	Loop of Henle
DTWD2	1.4640314215566e-57	0.252896829500441	0.27	0.189	4.23397887114169e-53	Loop of Henle
IQCB1	2.12741182142984e-56	0.27953425555319	0.289	0.206	6.15247498757509e-52	Loop of Henle
METTL16	1.01974296171258e-55	0.290354265054361	0.272	0.192	2.94909664527279e-51	Loop of Henle
NOP58	1.2301357154662e-55	0.29347886128686	0.269	0.189	3.55755248912825e-51	Loop of Henle
TRAK2	1.50499970084312e-55	0.312328277795309	0.259	0.181	4.35245913483831e-51	Loop of Henle
MAVS	7.20882759610492e-53	0.293057522147249	0.261	0.183	2.08479294079354e-48	Loop of Henle
TIAM2	2.25691621832453e-47	0.25080917667982	0.374	0.286	6.52700170339454e-43	Loop of Henle
AC002460.2	1.89892599847215e-44	0.37995968330974	0.332	0.257	5.49169398758146e-40	Loop of Henle
TEX9	6.16094472202798e-44	0.259101994166025	0.294	0.218	1.78174521361049e-39	Loop of Henle
RBM27	6.35317560885171e-44	0.260887656032287	0.257	0.186	1.83733838607992e-39	Loop of Henle
PLS3	4.41325568132124e-38	0.259745183741482	0.293	0.222	1.2763135430381e-33	Loop of Henle
TMEM52B	5.96908818678266e-29	0.482181400102516	0.285	0.233	1.72626030361755e-24	Loop of Henle

gene	p_val	avg_log2FC	pct.1	pct.2	p_val_adj	cluster
SNTB1	0	3.17091994617645	0.723	0.168	0	Parietal Epithelial Cell
CLNK	0	4.62525852363571	0.682	0.136	0	Parietal Epithelial Cell
ATP6V1C2	0	4.44405484195502	0.598	0.054	0	Parietal Epithelial Cell
GALNT17	0	4.22940125585721	0.629	0.098	0	Parietal Epithelial Cell
ADGRF5	0	4.20456976413315	0.661	0.14	0	Parietal Epithelial Cell
RCAN2	0	2.9765717893297	0.703	0.19	0	Parietal Epithelial Cell
NRXN3	0	3.77437661271832	0.587	0.077	0	Parietal Epithelial Cell
PDE1C	0	3.41746647386165	0.666	0.162	0	Parietal Epithelial Cell
PACRG	0	3.29812347758575	0.789	0.288	0	Parietal Epithelial Cell
SLC26A7	0	4.99286330143537	0.703	0.202	0	Parietal Epithelial Cell
LINC01187	0	3.87287211143383	0.536	0.041	0	Parietal Epithelial Cell
TSPAN5	0	3.09754070902866	0.603	0.121	0	Parietal Epithelial Cell
PSD3	0	2.78930736865571	0.629	0.149	0	Parietal Epithelial Cell
HS6ST3	0	4.97366178984999	0.523	0.044	0	Parietal Epithelial Cell
PDE1A	0	1.89235419535488	0.88	0.402	0	Parietal Epithelial Cell
IL18	0	3.12392885933788	0.533	0.063	0	Parietal Epithelial Cell
SHOC1	0	5.12843592289502	0.521	0.057	0	Parietal Epithelial Cell
AC026333.3	0	5.6190631754669	0.475	0.019	0	Parietal Epithelial Cell
TMEM101	0	3.71891108018272	0.565	0.112	0	Parietal Epithelial Cell
ABCC4	0	3.02564485348519	0.678	0.233	0	Parietal Epithelial Cell
ADAMTSL1	0	3.30652171453785	0.523	0.084	0	Parietal Epithelial Cell
ATP6V0D2	0	3.04845624144041	0.614	0.186	0	Parietal Epithelial Cell
DMRT2	0	5.74892722447289	0.439	0.011	0	Parietal Epithelial Cell
KIT	0	4.66294765042453	0.452	0.025	0	Parietal Epithelial Cell
LY86-AS1	0	4.78616796041763	0.462	0.036	0	Parietal Epithelial Cell
NXPH2	0	5.37118204681287	0.449	0.023	0	Parietal Epithelial Cell
SLC4A9	0	1.72092559623888	0.503	0.084	0	Parietal Epithelial Cell
SIPA1L2	0	3.0891407972668	0.479	0.068	0	Parietal Epithelial Cell
CA8	0	3.52456198236644	0.475	0.07	0	Parietal Epithelial Cell
DOCK10	0	2.88478178065899	0.478	0.075	0	Parietal Epithelial Cell
CACNB2	0	2.75614140608849	0.616	0.215	0	Parietal Epithelial Cell
CELF2	0	1.55235860289306	0.523	0.124	0	Parietal Epithelial Cell
LEF1	0	4.1546622196748	0.422	0.023	0	Parietal Epithelial Cell
SEMA3C	0	2.90972918922228	0.505	0.107	0	Parietal Epithelial Cell
ADGRF1	0	3.26813134870247	0.48	0.092	0	Parietal Epithelial Cell
SMIM34B	0	4.86597081124363	0.408	0.021	0	Parietal Epithelial Cell
RHBG	0	2.61189857594611	0.537	0.153	0	Parietal Epithelial Cell
FYB2	0	2.49220310019288	0.549	0.169	0	Parietal Epithelial Cell
EPS8	0	2.22029584997366	0.68	0.311	0	Parietal Epithelial Cell
MPPED2	0	2.22113440651424	0.643	0.277	0	Parietal Epithelial Cell
AC092422.1	0	4.59252073131427	0.39	0.026	0	Parietal Epithelial Cell
THRB-AS1	0	5.21267883497382	0.375	0.016	0	Parietal Epithelial Cell

gene	p_val	avg_log2FC	pct.1	pct.2	p_val_adj	cluster
ITIH5	0	3.0523513999367	0.406	0.048	0	Parietal Epithelial Cell
INPP4A	0	1.97166273656188	0.534	0.177	0	Parietal Epithelial Cell
VWA5B1	0	3.95679286256516	0.379	0.037	0	Parietal Epithelial Cell
AP004608.1	0	2.34099461723602	0.429	0.09	0	Parietal Epithelial Cell
GRB14	0	1.71320220852256	0.702	0.364	0	Parietal Epithelial Cell
CTTNBP2	0	1.93476222426238	0.511	0.176	0	Parietal Epithelial Cell
EFCAB13	0	2.00330296158476	0.528	0.193	0	Parietal Epithelial Cell
PALLD	0	1.85053779886146	0.553	0.22	0	Parietal Epithelial Cell
CNTNAP5	0	5.36684272807152	0.356	0.023	0	Parietal Epithelial Cell
GULP1	0	1.53550410767171	0.694	0.362	0	Parietal Epithelial Cell
SYT17	0	3.42728100406789	0.377	0.048	0	Parietal Epithelial Cell
C12orf75	0	2.26515585539264	0.439	0.112	0	Parietal Epithelial Cell
ITGA6	0	2.57137415331347	0.677	0.352	0	Parietal Epithelial Cell
ARHGAP18	0	2.33920079742197	0.682	0.36	0	Parietal Epithelial Cell
PCNX4	0	1.65312078748119	0.655	0.333	0	Parietal Epithelial Cell
UAP1	0	3.7348628042069	0.39	0.071	0	Parietal Epithelial Cell
MOB1B	0	1.9839688207736	0.619	0.3	0	Parietal Epithelial Cell
SLIT2	0	2.73752291469603	0.719	0.402	0	Parietal Epithelial Cell
CASC15	0	1.65943267764947	0.478	0.161	0	Parietal Epithelial Cell
ARNT2	0	1.53693582849611	0.541	0.226	0	Parietal Epithelial Cell
LIMS1	0	1.55203066606263	0.69	0.375	0	Parietal Epithelial Cell
PTPRJ	0	1.31870970210298	0.807	0.493	0	Parietal Epithelial Cell
NTN1	0	3.04927799721981	0.368	0.054	0	Parietal Epithelial Cell
GLCCI1	0	1.56078276891251	0.592	0.278	0	Parietal Epithelial Cell
AL450352.1	0	3.13697297197891	0.345	0.032	0	Parietal Epithelial Cell
GPAT3	0	1.96337858731192	0.602	0.29	0	Parietal Epithelial Cell
GMPR	0	2.7438301957847	0.396	0.086	0	Parietal Epithelial Cell
AL356124.1	0	2.56594050860884	0.483	0.177	0	Parietal Epithelial Cell
FAM13C	0	4.33289116090912	0.323	0.017	0	Parietal Epithelial Cell
STIM1	0	1.40996096342883	0.483	0.177	0	Parietal Epithelial Cell
PLCG2	0	1.87138261286175	0.608	0.304	0	Parietal Epithelial Cell
UST	0	2.43032007612958	0.453	0.15	0	Parietal Epithelial Cell
EFNA5	0	0.87114011415771	0.74	0.437	0	Parietal Epithelial Cell
MYO1D	0	1.1282612771305	0.622	0.319	0	Parietal Epithelial Cell
PXK	0	2.08777656508003	0.485	0.183	0	Parietal Epithelial Cell
LINC01091	0	2.05631194555013	0.404	0.103	0	Parietal Epithelial Cell
TBC1D14	0	1.81433758761654	0.62	0.32	0	Parietal Epithelial Cell
SCIN	0	1.34519004691662	0.54	0.241	0	Parietal Epithelial Cell
KBTBD12	0	3.67646782930076	0.33	0.032	0	Parietal Epithelial Cell
AL691403.1	0	4.02706853736965	0.321	0.023	0	Parietal Epithelial Cell
TMEM213	0	1.58939062024401	0.53	0.233	0	Parietal Epithelial Cell
STAP1	0	4.81302500285749	0.308	0.013	0	Parietal Epithelial Cell

gene	p_val	avg_log2FC	pct.1	pct.2	p_val_adj	cluster
DSCAML1	0	1.5633619265474	0.598	0.304	0	Parietal Epithelial Cell
KIAA1328	0	1.21276544791003	0.659	0.367	0	Parietal Epithelial Cell
GRIP1	0	1.13725389222448	0.62	0.328	0	Parietal Epithelial Cell
SMURF1	0	1.50118575839977	0.582	0.293	0	Parietal Epithelial Cell
THRB	0	1.34553845199831	0.751	0.464	0	Parietal Epithelial Cell
STK33	0	0.93474616629856	0.658	0.373	0	Parietal Epithelial Cell
EGFR	0	1.13200148182989	0.609	0.324	0	Parietal Epithelial Cell
RHCG	0	2.28259867180702	0.402	0.119	0	Parietal Epithelial Cell
TBC1D4	0	1.18042637999742	0.702	0.42	0	Parietal Epithelial Cell
PLCB4	0	1.2352972684027	0.462	0.182	0	Parietal Epithelial Cell
LINC01435	0	4.47526436712343	0.336	0.058	0	Parietal Epithelial Cell
DSG2	0	2.19664649595984	0.364	0.086	0	Parietal Epithelial Cell
SHROOM3	0	1.56431134639988	0.778	0.501	0	Parietal Epithelial Cell
LITAF	0	1.80273286393583	0.441	0.164	0	Parietal Epithelial Cell
RERG	0	1.71128071856566	0.506	0.231	0	Parietal Epithelial Cell
ITGB3	0	3.09292741693389	0.329	0.055	0	Parietal Epithelial Cell
GNG12	0	1.61952212723198	0.554	0.281	0	Parietal Epithelial Cell
DOCK8	0	1.45886889436108	0.466	0.194	0	Parietal Epithelial Cell
SLC4A1	0	2.80470452509809	0.35	0.079	0	Parietal Epithelial Cell
PRLR	0	1.38354715143364	0.521	0.251	0	Parietal Epithelial Cell
TMPRSS2	0	1.52427015283373	0.488	0.218	0	Parietal Epithelial Cell
PPP1R12B	0	1.20027714189052	0.635	0.366	0	Parietal Epithelial Cell
TMEM117	0	2.01041817145106	0.723	0.454	0	Parietal Epithelial Cell
GALNT10	0	1.5398164712896	0.448	0.18	0	Parietal Epithelial Cell
MYRIP	0	1.87272062051152	0.401	0.134	0	Parietal Epithelial Cell
ABCA5	0	1.34494010658905	0.673	0.406	0	Parietal Epithelial Cell
BAIAP2L1	0	1.72050086862565	0.407	0.14	0	Parietal Epithelial Cell
ATP6V0A4	0	1.35877051849935	0.582	0.315	0	Parietal Epithelial Cell
PTBP3	0	1.0425564442369	0.604	0.337	0	Parietal Epithelial Cell
AC107223.1	0	1.72784477751073	0.426	0.16	0	Parietal Epithelial Cell
CDH1	0	1.33492763776608	0.451	0.185	0	Parietal Epithelial Cell
MPP7	0	1.53563243345011	0.474	0.21	0	Parietal Epithelial Cell
DMD	0	1.52639434334077	0.388	0.124	0	Parietal Epithelial Cell
NRDC	0	1.20774558722713	0.586	0.322	0	Parietal Epithelial Cell
AC092894.1	0	2.69921854365258	0.299	0.036	0	Parietal Epithelial Cell
PCED1B	0	1.24883789414367	0.38	0.117	0	Parietal Epithelial Cell
ATP6V1G3	0	3.09941438930174	0.289	0.026	0	Parietal Epithelial Cell
AP000282.1	0	2.49642879179156	0.339	0.077	0	Parietal Epithelial Cell
TPM1	0	1.50813073816186	0.484	0.226	0	Parietal Epithelial Cell
PPP2R5A	0	1.40376668947354	0.498	0.241	0	Parietal Epithelial Cell
AL157911.1	0	1.8596551572705	0.351	0.097	0	Parietal Epithelial Cell
FNDC3A	0	1.08932146655031	0.751	0.5	0	Parietal Epithelial Cell

gene	p_val	avg_log2FC	pct.1	pct.2	p_val_adj	cluster
CFH	0	4.72607384874242	0.274	0.025	0	Parietal Epithelial Cell
SYK	0	1.51889928056294	0.375	0.127	0	Parietal Epithelial Cell
ATP9A	0	1.62954507384559	0.382	0.134	0	Parietal Epithelial Cell
ZSWIM6	0	1.25160316718089	0.791	0.544	0	Parietal Epithelial Cell
CDYL2	0	1.9756796318932	0.35	0.104	0	Parietal Epithelial Cell
SDC2	0	1.93727447377498	0.353	0.108	0	Parietal Epithelial Cell
NPC1	0	1.42155737965847	0.428	0.184	0	Parietal Epithelial Cell
LAMA3	0	3.50597009836192	0.271	0.028	0	Parietal Epithelial Cell
CCBE1	0	1.93513982799588	0.293	0.051	0	Parietal Epithelial Cell
ZFPM2	0	2.94784920356848	0.319	0.078	0	Parietal Epithelial Cell
TBC1D1	0	1.07532300077187	0.716	0.476	0	Parietal Epithelial Cell
AL031123.1	0	4.30485694142081	0.253	0.014	0	Parietal Epithelial Cell
ELOVL7	0	1.46258482182476	0.453	0.216	0	Parietal Epithelial Cell
TENM3	0	2.36780658952422	0.314	0.078	0	Parietal Epithelial Cell
HIPK2	0	0.91805504683544	0.773	0.537	0	Parietal Epithelial Cell
SUSD1	0	1.17794092310287	0.406	0.171	0	Parietal Epithelial Cell
DCLK2	0	1.27224747017486	0.366	0.132	0	Parietal Epithelial Cell
SYT10	0	3.35651354512484	0.258	0.025	0	Parietal Epithelial Cell
CAB39	0	1.53561368929764	0.593	0.361	0	Parietal Epithelial Cell
PTGER3	0	1.96015317740312	0.687	0.463	0	Parietal Epithelial Cell
RARB	0	1.39749822584862	0.387	0.163	0	Parietal Epithelial Cell
FOXP1	0	1.36031724486302	0.843	0.619	0	Parietal Epithelial Cell
FOXO1	0	1.18868303919244	0.677	0.457	0	Parietal Epithelial Cell
AHCYL2	0	1.44806786482312	0.667	0.449	0	Parietal Epithelial Cell
MAP2	0	2.55449742239342	0.261	0.046	0	Parietal Epithelial Cell
ABHD2	0	1.42619505744556	0.341	0.128	0	Parietal Epithelial Cell
IRS1	0	2.30274061160066	0.291	0.078	0	Parietal Epithelial Cell
MAGI3	0	1.09886469107147	0.748	0.536	0	Parietal Epithelial Cell
OXR1	0	0.883698450054487	0.85	0.64	0	Parietal Epithelial Cell
PARVB	0	1.53018682336265	0.281	0.072	0	Parietal Epithelial Cell
PPFIBP2	0	1.31478274282195	0.331	0.123	0	Parietal Epithelial Cell
MAPRE3	0	2.15244648448886	0.272	0.067	0	Parietal Epithelial Cell
DLC1	0	1.03264000673192	0.312	0.108	0	Parietal Epithelial Cell
LAMA2	0	2.66673938545206	0.25	0.049	0	Parietal Epithelial Cell
ARAP1	0	1.49021841984425	0.276	0.085	0	Parietal Epithelial Cell
LONRF2	0	2.36992046186734	0.26	0.071	0	Parietal Epithelial Cell
DGKI	0	1.14899999662448	0.259	0.07	0	Parietal Epithelial Cell
EXOSC7	0	1.89189459796527	0.266	0.079	0	Parietal Epithelial Cell
ATP6AP2	0	1.53436684361625	0.256	0.076	0	Parietal Epithelial Cell
NEDD4L	0	1.37491184431164	0.841	0.681	0	Parietal Epithelial Cell
MAGI1	0	1.93609856941784	0.909	0.751	0	Parietal Epithelial Cell
ZBTB20	0	0.57423542188317	0.957	0.902	0	Parietal Epithelial Cell

gene	p_val	avg_log2FC	pct.1	pct.2	p_val_adj	cluster
PDE4D	0	0.689981084959245	0.977	0.935	0	Parietal Epithelial Cell
UHMK1	9.91064298616332e-306	1.61954300584534	0.301	0.11	2.86615795159843e-301	Parietal Epithelial Cell
PRKG1	6.58639044830863e-304	0.646120565528826	0.783	0.523	1.90478411765086e-299	Parietal Epithelial Cell
PLPP4	2.10689393040828e-297	1.49409017436637	0.256	0.083	6.09313724674074e-293	Parietal Epithelial Cell
AC016705.2	3.27969767826713e-296	1.56311651632299	0.301	0.11	9.48488568554853e-292	Parietal Epithelial Cell
ALDH1A2	4.30252311385525e-295	3.78583989061446	0.356	0.17	1.24428968452694e-290	Parietal Epithelial Cell
ERC2	4.44483068642172e-292	1.40209852072072	0.332	0.13	1.28544503451316e-287	Parietal Epithelial Cell
CHL1	1.61327860666792e-291	1.40663040084769	0.403	0.182	4.66560173048362e-287	Parietal Epithelial Cell
SHROOM2	5.36349360020631e-289	1.3240041154647	0.302	0.111	1.55112234917967e-284	Parietal Epithelial Cell
SLC22A23	6.57006285837219e-286	1.1282481651476	0.507	0.263	1.90006217864124e-281	Parietal Epithelial Cell
TCF7L1	2.40903672503259e-285	0.941021436821876	0.333	0.124	6.96693420879424e-281	Parietal Epithelial Cell
ITGAV	2.80380071443068e-285	1.24354451591884	0.547	0.305	8.10859166613353e-281	Parietal Epithelial Cell
MAPRE2	5.58967308765407e-284	1.35724318022606	0.307	0.115	1.61653345694956e-279	Parietal Epithelial Cell
CRYBG3	3.6493443490237e-280	1.11434357673364	0.445	0.212	1.05539038573765e-275	Parietal Epithelial Cell
IGFBP5	4.84481384763292e-280	1.18740912424376	0.286	0.102	1.40112016473544e-275	Parietal Epithelial Cell
IQSEC1	9.70014851800196e-279	1.51843191315689	0.367	0.16	2.80528295140617e-274	Parietal Epithelial Cell
ATP6V1A	2.04487720716814e-278	1.24280648170072	0.523	0.298	5.91378488313026e-274	Parietal Epithelial Cell
PLCH1	9.58550383216927e-278	1.19158069256469	0.354	0.145	2.77212770826335e-273	Parietal Epithelial Cell
DAP	1.50110478104617e-276	1.26171024337478	0.389	0.174	4.34119502678553e-272	Parietal Epithelial Cell
LINC00623	4.07302556319476e-274	1.23873430600612	0.374	0.162	1.17791899287592e-269	Parietal Epithelial Cell
LRMDA	2.17726911027339e-272	0.618410219456222	0.934	0.763	6.29666226691065e-268	Parietal Epithelial Cell
WNK3	4.74149049435906e-272	1.39102829921574	0.291	0.109	1.37123905096864e-267	Parietal Epithelial Cell
FGF9	1.19524598092886e-271	1.47703722803867	0.32	0.129	3.45665137684628e-267	Parietal Epithelial Cell
PLEKHH1	2.33247678321656e-270	1.37189613836665	0.27	0.096	6.74552285706228e-266	Parietal Epithelial Cell
ARHGEF28	2.03402591627562e-269	0.817819276411295	0.709	0.466	5.88240294986908e-265	Parietal Epithelial Cell
LIMA1	2.49917146068659e-269	0.992033504544905	0.457	0.222	7.22760386430561e-265	Parietal Epithelial Cell
NCOA7	7.27131621257796e-269	0.972920955625562	0.591	0.34	2.10286464867754e-264	Parietal Epithelial Cell
PLCL1	1.44413370902699e-267	0.862731689215944	0.86	0.708	4.17643468650606e-263	Parietal Epithelial Cell
CCDC200	5.5885509882562e-267	1.49681177683067	0.257	0.09	1.61620894580369e-262	Parietal Epithelial Cell
MAP4K3	1.17056361454117e-264	0.967513412093867	0.697	0.486	3.38526997325305e-260	Parietal Epithelial Cell
PTPN21	3.20276606209873e-264	1.11982719295785	0.388	0.173	9.26239945158954e-260	Parietal Epithelial Cell
SPTLC2	2.88684750787556e-263	1.07990520171063	0.45	0.222	8.34876299277611e-259	Parietal Epithelial Cell
LINC02343	8.77424019302578e-263	1.13371534020175	0.434	0.208	2.53751026382306e-258	Parietal Epithelial Cell
SGPP2	5.09130977814573e-260	1.27683146410497	0.449	0.226	1.47240678783974e-255	Parietal Epithelial Cell
ARL15	2.34282844947893e-259	0.452405793972428	0.853	0.655	6.77545987589305e-255	Parietal Epithelial Cell
PLCE1	2.78035533282473e-259	0.804049491733099	0.268	0.095	8.04078762252912e-255	Parietal Epithelial Cell
FAM110B	2.83488364330958e-254	1.03236511928106	0.398	0.18	8.19848349645131e-250	Parietal Epithelial Cell
UBL3	6.0686263829938e-251	1.02955393138976	0.507	0.278	1.75504674996181e-246	Parietal Epithelial Cell
DIP2C	6.89026075334082e-251	1.50554019347198	0.789	0.621	1.99266340986617e-246	Parietal Epithelial Cell
MOB3B	3.80939035703525e-250	1.1260406289474	0.474	0.245	1.10167569125459e-245	Parietal Epithelial Cell
NSUN7	1.24915532274486e-249	1.25085869932086	0.332	0.14	3.61255719337812e-245	Parietal Epithelial Cell
TXNIP	8.11399809995864e-248	1.03105993200338	0.605	0.371	2.34656825050804e-243	Parietal Epithelial Cell

gene	p_val	avg_log2FC	pct.1	pct.2	p_val_adj	cluster
LMO7	8.62930882989202e-248	0.751996701152216	0.736	0.501	2.49559611360477e-243	Parietal Epithelial Cell
CAMSAP2	8.71422179082648e-248	0.950129689557766	0.509	0.276	2.52015294190702e-243	Parietal Epithelial Cell
C11orf80	3.97208758648023e-247	0.856757842597406	0.502	0.26	1.14872773001008e-242	Parietal Epithelial Cell
RALGPS2	3.78047172534947e-246	0.866721386874607	0.596	0.362	1.09331242297107e-241	Parietal Epithelial Cell
AMFR	1.31661126586506e-245	1.17310312545855	0.393	0.186	3.80763978088176e-241	Parietal Epithelial Cell
MBOAT1	1.51509409856986e-245	1.33305805065291	0.288	0.112	4.38165213306404e-241	Parietal Epithelial Cell
TMCO4	2.5346536212371e-242	1.19775151746186	0.404	0.195	7.33021827261768e-238	Parietal Epithelial Cell
GAS6	4.03586026132421e-241	1.09266609151387	0.32	0.131	1.16717078757496e-236	Parietal Epithelial Cell
ZDHHC3	2.86293144120509e-240	1.35552738660754	0.351	0.158	8.27959772796513e-236	Parietal Epithelial Cell
PRKCA	6.91760794096265e-240	0.62373598142352	0.802	0.586	2.0005722165264e-235	Parietal Epithelial Cell
PHLPP1	2.5590163025128e-239	0.997276712286925	0.493	0.267	7.40067514686702e-235	Parietal Epithelial Cell
MUC20	1.10654285789934e-238	1.19513667454224	0.484	0.262	3.2001219450449e-234	Parietal Epithelial Cell
PICALM	1.74862419559428e-236	0.798714204169074	0.751	0.566	5.05702117365865e-232	Parietal Epithelial Cell
MTHFD1L	4.5486384614296e-236	1.09600712578761	0.337	0.145	1.31546624304544e-231	Parietal Epithelial Cell
STK17A	3.06206455690304e-235	1.09878676835925	0.267	0.101	8.8554906985636e-231	Parietal Epithelial Cell
ABR	1.077363934594e-233	1.00043682026412	0.33	0.138	3.11573649884584e-229	Parietal Epithelial Cell
KIF21A	1.1416501953839e-232	0.970581802000234	0.489	0.262	3.30165236505025e-228	Parietal Epithelial Cell
ANKRD44	8.14348310467621e-232	0.581415649401844	0.386	0.176	2.35509531387236e-227	Parietal Epithelial Cell
ABCC5	3.73360238210593e-230	1.07514720358379	0.489	0.273	1.07975780890504e-225	Parietal Epithelial Cell
MEGF9	3.85932436536074e-230	1.06537603304732	0.429	0.216	1.11611660646233e-225	Parietal Epithelial Cell
ITPR2	1.01491162137148e-228	1.041559847	0.761	0.679	2.93512440900631e-224	Parietal Epithelial Cell
PNPLA8	5.26328781575444e-228	1.18005407315219	0.393	0.194	1.52214283631618e-223	Parietal Epithelial Cell
ADIPOR2	5.82219184931507e-227	1.06742070601671	0.438	0.228	1.68377788282192e-222	Parietal Epithelial Cell
MET	6.52963276217923e-227	0.767384073762244	0.437	0.215	1.88836979482223e-222	Parietal Epithelial Cell
DOCK4	1.13490587207811e-226	0.647006794265181	0.569	0.332	3.28214778204988e-222	Parietal Epithelial Cell
TMEM116	3.09504497247193e-226	1.23368849836611	0.425	0.226	8.95087006038881e-222	Parietal Epithelial Cell
JADE1	1.28239850895438e-225	1.15770468435972	0.43	0.224	3.70869648789606e-221	Parietal Epithelial Cell
EXOC6	2.44473824656336e-225	0.885786554248093	0.425	0.215	7.07018300906124e-221	Parietal Epithelial Cell
ASXL1	5.77296949144019e-225	0.889647145203347	0.569	0.343	1.6695427769245e-220	Parietal Epithelial Cell
GNG12-AS1	2.89228967739289e-224	1.63170282422905	0.274	0.111	8.36450174702024e-220	Parietal Epithelial Cell
SLIT3	8.51724642621166e-224	1.80341228440284	0.28	0.117	2.46318766646041e-219	Parietal Epithelial Cell
MCU	1.32529517158762e-219	0.843800011332043	0.552	0.32	3.83275363623141e-215	Parietal Epithelial Cell
CDC42SE2	4.31888618229981e-218	0.86813570193455	0.532	0.309	1.2490218839211e-213	Parietal Epithelial Cell
STK3	1.06245578944412e-217	0.697109560307936	0.755	0.567	3.07262214307239e-213	Parietal Epithelial Cell
AC019197.1	5.70999062781806e-216	0.365520579093283	0.865	0.662	1.65132928956498e-211	Parietal Epithelial Cell
GLIS3	9.21663456935491e-214	0.448149116321445	0.861	0.66	2.66545071745744e-209	Parietal Epithelial Cell
CAMTA1	2.16865827732165e-213	0.817125273713071	0.65	0.43	6.27175973801421e-209	Parietal Epithelial Cell
FER	1.48510089490738e-212	0.76645262979038	0.656	0.428	4.29491178807215e-208	Parietal Epithelial Cell
SRGAP1	5.61471204444459e-209	0.711952621729001	0.491	0.263	1.62377472325338e-204	Parietal Epithelial Cell
TMEM65	3.26688475895667e-208	1.12872447508882	0.38	0.189	9.44783072290268e-204	Parietal Epithelial Cell
CRIM1	4.58720310110452e-206	1.22825254053154	0.62	0.405	1.32661913683943e-201	Parietal Epithelial Cell
PBX3	1.16852130039375e-204	0.92256183994005	0.405	0.203	3.37936360073872e-200	Parietal Epithelial Cell

gene	p_val	avg_log2FC	pct.1	pct.2	p_val_adj	cluster
TBL1X	1.69755791769174e-204	0.737131462898854	0.684	0.457	4.90933749796452e-200	Parietal Epithelial Cell
RAP1GAP	8.25932552946855e-203	1.10968377708265	0.353	0.166	2.38859694312231e-198	Parietal Epithelial Cell
ATP10D	1.06510388518773e-202	0.957432501493565	0.427	0.224	3.08028043596292e-198	Parietal Epithelial Cell
STK39	1.94968882681934e-202	0.845413700195701	0.556	0.336	5.63850008716153e-198	Parietal Epithelial Cell
EGLN1	1.08594018334495e-201	1.17726355145612	0.384	0.196	3.14053901023358e-197	Parietal Epithelial Cell
ACADL	2.74787582090256e-201	0.98817415740714	0.277	0.115	7.94685687405021e-197	Parietal Epithelial Cell
KIF13A	1.03798078944697e-200	0.869848125560501	0.516	0.303	3.00184044308064e-196	Parietal Epithelial Cell
IL1R1	1.14260038555332e-200	1.73004447084008	0.256	0.106	3.3044003150202e-196	Parietal Epithelial Cell
NR4A1	1.4540746207512e-199	1.10239035852947	0.379	0.187	4.20518380321246e-195	Parietal Epithelial Cell
SPOPL	2.54078850715135e-197	1.28749924791598	0.279	0.12	7.34796036268172e-193	Parietal Epithelial Cell
SH3BP4	6.6760919430748e-197	0.934804490885685	0.374	0.183	1.93072578993723e-192	Parietal Epithelial Cell
SHTN1	1.28836140857163e-196	0.837647396151384	0.458	0.25	3.72594119358917e-192	Parietal Epithelial Cell
GPD2	7.23030495202305e-195	1.0179929009429	0.321	0.148	2.09100419212507e-190	Parietal Epithelial Cell
MAML3	1.88439075023865e-194	0.913094360337888	0.584	0.364	5.44965804969018e-190	Parietal Epithelial Cell
DUSP1	3.40880030975506e-193	1.25232564406115	0.37	0.186	9.85825049581163e-189	Parietal Epithelial Cell
TSPAN12	4.8373320871108e-193	1.53244564228867	0.259	0.11	1.39895643959244e-188	Parietal Epithelial Cell
SPATA13	2.99174039878139e-192	0.68390801255187	0.641	0.427	8.65211323327577e-188	Parietal Epithelial Cell
PRKAA2	1.12243678785347e-188	0.785968003092038	0.538	0.328	3.24608719047225e-184	Parietal Epithelial Cell
UBR3	3.94172465313175e-188	0.8085704177846	0.667	0.481	1.1399467696857e-183	Parietal Epithelial Cell
SPTAN1	1.13516025209459e-187	0.838625660984967	0.478	0.269	3.28288344905756e-183	Parietal Epithelial Cell
ST3GAL6	1.25708813556004e-185	0.829519350645318	0.389	0.201	3.63549888803965e-181	Parietal Epithelial Cell
LIN7A	1.29389277613093e-185	1.34282879204828	0.324	0.159	3.74193790857066e-181	Parietal Epithelial Cell
ENOX2	1.96982208637902e-185	1.12950315079482	0.346	0.169	5.69672547380812e-181	Parietal Epithelial Cell
UGT8	5.92376313184074e-185	0.807137251676766	0.399	0.206	1.71315229772834e-180	Parietal Epithelial Cell
CLIP1	6.23942680863899e-184	0.729373511252937	0.595	0.384	1.8044422330584e-179	Parietal Epithelial Cell
PLOD2	8.82544684877536e-183	1.33845343523979	0.338	0.167	2.55231922866583e-178	Parietal Epithelial Cell
ANXA4	5.35075979444823e-181	0.844363189824734	0.514	0.31	1.54743973255443e-176	Parietal Epithelial Cell
TTC39B	6.8299862391363e-179	0.8123681579812	0.436	0.239	1.97523202035822e-174	Parietal Epithelial Cell
RAPGEF2	1.48046184171338e-178	0.748715896866719	0.658	0.464	4.28149564623511e-174	Parietal Epithelial Cell
SPRED2	5.60288160641752e-177	0.879446348106762	0.464	0.271	1.62035336057595e-172	Parietal Epithelial Cell
MAP3K5	5.11773586992583e-176	0.676773102430372	0.519	0.306	1.48004921358255e-171	Parietal Epithelial Cell
WASHC3	1.11283325780352e-175	1.08445510691331	0.281	0.128	3.21831378156778e-171	Parietal Epithelial Cell
DPYD	2.15607589109795e-175	0.630918439098829	0.585	0.369	6.23537147705526e-171	Parietal Epithelial Cell
COL6A1	1.43640826878651e-174	0.941982337816813	0.347	0.172	4.15409271333059e-170	Parietal Epithelial Cell
RAP1B	6.15790704839456e-173	0.757850598440168	0.5	0.302	1.78086671839571e-168	Parietal Epithelial Cell
ST3GAL1	1.0799483399526e-172	0.812839874704167	0.369	0.185	3.12321059914293e-168	Parietal Epithelial Cell
CCDC198	2.1657930726024e-172	0.782337465782708	0.68	0.475	6.26347356596615e-168	Parietal Epithelial Cell
SKAP1	1.46767322394946e-171	0.709963414743954	0.579	0.375	4.24451096366183e-167	Parietal Epithelial Cell
EZR	1.79701915486712e-171	0.861352136560026	0.504	0.305	5.1969793958757e-167	Parietal Epithelial Cell
ITPR1	5.08121295516322e-170	0.72059257524969	0.622	0.42	1.4694867866332e-165	Parietal Epithelial Cell
WWOX	3.61320933349438e-169	0.579274024287864	0.895	0.781	1.04494013924658e-164	Parietal Epithelial Cell
MAL	8.42321198152543e-168	0.88358920539983	0.418	0.231	2.43599290505715e-163	Parietal Epithelial Cell

gene	p_val	avg_log2FC	pct.1	pct.2	p_val_adj	cluster
YAF2	5.41519721069401e-165	0.834364238860273	0.449	0.261	1.56607503333271e-160	Parietal Epithelial Cell
DPY19L2	2.69070053004933e-164	0.693111053935661	0.52	0.318	7.78150593290268e-160	Parietal Epithelial Cell
MAP4K3-DT	1.33907168063865e-163	0.771052624373649	0.578	0.386	3.87259530040697e-159	Parietal Epithelial Cell
SINHCAF	1.38772721349241e-162	1.01168544947742	0.312	0.151	4.01330710142006e-158	Parietal Epithelial Cell
VPS37B	2.3932379874609e-162	0.721356614439295	0.424	0.239	6.92124425973692e-158	Parietal Epithelial Cell
FREM1	4.17869300965533e-162	0.737798611982674	0.577	0.379	1.20847801839232e-157	Parietal Epithelial Cell
NAV2	1.72517163215191e-161	0.520965933058273	0.576	0.36	4.98919636018333e-157	Parietal Epithelial Cell
EXT1	4.57285839473968e-161	0.690334820265835	0.701	0.522	1.32247064775872e-156	Parietal Epithelial Cell
STOX2	1.61549003074571e-160	0.668612182410336	0.52	0.315	4.6719971689166e-156	Parietal Epithelial Cell
RNF169	1.94716487309694e-160	0.817042704423396	0.429	0.243	5.63120081299636e-156	Parietal Epithelial Cell
BMPR1B	1.054162381907e-159	0.325462073413721	0.419	0.219	3.04863760847504e-155	Parietal Epithelial Cell
USP54	1.37470167957695e-159	1.01270177510022	0.343	0.178	3.97563725733654e-155	Parietal Epithelial Cell
THSD4	1.66191624105124e-159	0.465267479817241	0.516	0.301	4.80626176912018e-155	Parietal Epithelial Cell
MACF1	3.72572571249878e-159	0.433759587598157	0.873	0.727	1.07747987605465e-154	Parietal Epithelial Cell
IPCEF1	2.19406606088725e-158	0.898318604912774	0.502	0.318	6.34523904808592e-154	Parietal Epithelial Cell
MSI2	2.77068279855088e-158	0.525638758565355	0.846	0.706	8.01281465340914e-154	Parietal Epithelial Cell
PARD3B	7.42334795301919e-157	0.497140320731935	0.843	0.676	2.14683222801315e-152	Parietal Epithelial Cell
FMNL2	4.66550077181463e-156	0.675365546295729	0.581	0.377	1.34926282320879e-151	Parietal Epithelial Cell
MYO10	5.07172078558665e-156	0.702300334143662	0.595	0.399	1.46674165119166e-151	Parietal Epithelial Cell
AC016831.7	9.29210148321005e-153	0.721265752082663	0.407	0.228	2.68727574894435e-148	Parietal Epithelial Cell
FBXO34	9.71210327298044e-153	0.973289829359862	0.565	0.393	2.80874026654594e-148	Parietal Epithelial Cell
FBXL7	1.11491056668261e-152	1.65497855366031	0.269	0.132	3.22432135884611e-148	Parietal Epithelial Cell
KLF6	3.81649998279055e-152	1.19014684231795	0.385	0.216	1.10373179502303e-147	Parietal Epithelial Cell
GRB10	1.39125638456286e-150	0.252948951456469	0.527	0.304	4.02351346415578e-146	Parietal Epithelial Cell
FLNB	2.96600888225011e-150	0.684223757844238	0.518	0.32	8.57769768746733e-146	Parietal Epithelial Cell
BCL2	3.03938282613452e-150	0.649471217148316	0.639	0.43	8.78989513318104e-146	Parietal Epithelial Cell
OSBPL10	7.04595159781197e-150	1.08057287283477	0.54	0.362	2.03768920208722e-145	Parietal Epithelial Cell
PTPN2	1.32003385778824e-149	0.779702899628643	0.406	0.231	3.8175379167236e-145	Parietal Epithelial Cell
SEPTIN9	4.61297948206168e-148	0.608879813778947	0.533	0.331	1.33407366621224e-143	Parietal Epithelial Cell
PPARG	3.95223681990706e-147	0.647483314841508	0.45	0.258	1.14298688831712e-142	Parietal Epithelial Cell
EPB41L5	3.98558647453039e-146	0.716691556385073	0.693	0.5	1.15263160843419e-141	Parietal Epithelial Cell
NFAT5	6.5893940791213e-146	0.519510437547801	0.75	0.576	1.90565276768188e-141	Parietal Epithelial Cell
NNT	4.60918735124861e-145	0.803442056707874	0.499	0.322	1.3329769819811e-140	Parietal Epithelial Cell
ULK1	7.50188415810362e-145	0.887190942077878	0.278	0.133	2.16954489852357e-140	Parietal Epithelial Cell
gene	p_val	avg_log2FC	pct.1	pct.2	p_val_adj	cluster
ST5	1.91186964257006e-144	0.804209725349012	0.311	0.156	5.5291270063126e-140	Parietal Epithelial Cell
PITPNB	8.16267734509544e-144	0.752917891933451	0.395	0.224	2.3606462882016e-139	Parietal Epithelial Cell
ABCA10	8.64078383107981e-144	0.798574096644592	0.379	0.209	2.49891468394828e-139	Parietal Epithelial Cell
CASZ1	7.84827677480793e-143	0.872160997259312	0.43	0.256	2.26972164327445e-138	Parietal Epithelial Cell
LGR4	2.62560130893747e-142	0.641349674933126	0.698	0.508	7.59323898544717e-138	Parietal Epithelial Cell
ARHGAP8	1.40220184971184e-141	0.731961548093518	0.522	0.336	4.05516774936666e-137	Parietal Epithelial Cell
SCAMP1	9.34728245432883e-141	0.89354355413091	0.307	0.158	2.7032340857919e-136	Parietal Epithelial Cell

gene	p_val	avg_log2FC	pct.1	pct.2	p_val_adj	cluster
FAM107B	2.85521424079956e-140	0.724005668331627	0.503	0.319	8.25727958439234e-136	Parietal Epithelial Cell
SERGEF	8.28082284748665e-140	0.875924778044272	0.322	0.169	2.39481396749314e-135	Parietal Epithelial Cell
FAM13B	1.62170518653088e-139	0.64030848302232	0.514	0.328	4.6899713994473e-135	Parietal Epithelial Cell
HMBOX1	2.80128095105588e-139	0.673076107652175	0.467	0.291	8.1013045104536e-135	Parietal Epithelial Cell
DDX52	3.98942757761309e-139	0.878068727738253	0.254	0.119	1.1537424554457e-134	Parietal Epithelial Cell
ZNRF3	8.40551507234962e-139	0.615875555571734	0.535	0.34	2.43087495892351e-134	Parietal Epithelial Cell
RBBP8	8.85779796344491e-139	0.725248144822007	0.526	0.331	2.56167517102827e-134	Parietal Epithelial Cell
PAX8	4.01113399478942e-138	0.428014381218655	0.863	0.649	1.1600199512931e-133	Parietal Epithelial Cell
TLE1	5.52765905706828e-138	0.712426525232277	0.282	0.135	1.59859899930415e-133	Parietal Epithelial Cell
SCN2A	1.29542894493913e-137	0.440440940062947	0.547	0.337	3.74638050876395e-133	Parietal Epithelial Cell
NPNT	1.82248601455478e-137	0.591153310457116	0.364	0.199	5.27062955409242e-133	Parietal Epithelial Cell
AC010210.1	2.55460119408792e-137	0.785689193627225	0.341	0.184	7.38790665330225e-133	Parietal Epithelial Cell
CD9	4.58892452542388e-136	0.735299847089974	0.254	0.119	1.32711697275259e-131	Parietal Epithelial Cell
SBF2	7.3293945207647e-135	0.462096824356482	0.816	0.672	2.11966089540515e-130	Parietal Epithelial Cell
RNF144B	1.15394166477564e-134	0.729517901865776	0.254	0.119	3.33719929453115e-130	Parietal Epithelial Cell
CLIP4	3.81889099284063e-134	0.890710705314833	0.313	0.164	1.10442327512951e-129	Parietal Epithelial Cell
ACTR3	3.46703314703609e-133	0.775770239741015	0.412	0.243	1.00266598612284e-128	Parietal Epithelial Cell
ARHGAP28	7.79507619911957e-133	0.371075105184144	0.253	0.116	2.25433603678538e-128	Parietal Epithelial Cell
LPP	1.89862571306216e-132	0.455318616922344	0.859	0.742	5.49082556217578e-128	Parietal Epithelial Cell
KCNMB2	7.13009549564506e-132	1.28754637381878	0.313	0.173	2.06202361734055e-127	Parietal Epithelial Cell
PDLIM5	9.16479086508497e-132	0.442234073846323	0.584	0.395	2.65045751818257e-127	Parietal Epithelial Cell
INTS9	9.29628087337362e-132	0.788694780918025	0.298	0.153	2.68848442857965e-127	Parietal Epithelial Cell
RICTOR	5.67064864239421e-131	0.62088136745856	0.563	0.386	1.63995158738041e-126	Parietal Epithelial Cell
DST	6.42237224828556e-131	0.499816231739498	0.68	0.48	1.85735005420419e-126	Parietal Epithelial Cell
SELENOI	6.74805824250991e-129	0.906451094866188	0.28	0.142	1.95153844373386e-124	Parietal Epithelial Cell
BACE2	5.53165624743667e-128	0.782177080690745	0.485	0.304	1.59975498675868e-123	Parietal Epithelial Cell
SPPL3	1.43124466730762e-127	0.625791819285976	0.576	0.401	4.13915957785362e-123	Parietal Epithelial Cell
RAD54L2	2.27154556138258e-126	0.693759343099512	0.379	0.218	6.56930976351842e-122	Parietal Epithelial Cell
ANKRD33B	1.17215019669619e-125	0.662642579462395	0.306	0.158	3.38985836884539e-121	Parietal Epithelial Cell
PPP3CA	1.41388106390188e-125	0.674947875830187	0.571	0.399	4.08894403680424e-121	Parietal Epithelial Cell
GNAQ	1.79426258521976e-125	0.481519680282018	0.696	0.527	5.18900739645554e-121	Parietal Epithelial Cell
RUFY3	2.32747930430167e-125	0.604848500936563	0.624	0.454	6.73107014804044e-121	Parietal Epithelial Cell
THSD7A	4.44335978796885e-125	0.476855317410017	0.768	0.595	1.28501965068059e-120	Parietal Epithelial Cell
PRKN	5.60906781955935e-125	0.544184393366246	0.81	0.692	1.62214241341656e-120	Parietal Epithelial Cell
AC006504.5	7.22569363084064e-125	0.880817566464335	0.312	0.168	2.08967059803911e-120	Parietal Epithelial Cell
ITGA1	1.04205596048847e-124	0.724286027656314	0.372	0.211	3.01362583773266e-120	Parietal Epithelial Cell
MRPS5	1.03493000770007e-123	0.739663758215836	0.404	0.241	2.9930175822686e-119	Parietal Epithelial Cell
CAPN2	2.06372472177176e-123	0.632992792469532	0.422	0.25	5.96829189536392e-119	Parietal Epithelial Cell
UMAD1	6.565960884627e-123	0.649225418675018	0.58	0.409	1.89887588783413e-118	Parietal Epithelial Cell
VAPA	4.19699585890069e-122	0.716557506441562	0.474	0.307	1.21377120239408e-117	Parietal Epithelial Cell
FAM49B	7.03915310209855e-122	0.503595882421436	0.563	0.375	2.0357230771269e-117	Parietal Epithelial Cell
BACH2	1.02144885559189e-120	0.727306508582075	0.421	0.258	2.95403009037174e-116	Parietal Epithelial Cell

gene	p_val	avg_log2FC	pct.1	pct.2	p_val_adj	cluster
ELP3	1.95213030090969e-120	0.901434483086392	0.312	0.17	5.64556083023082e-116	Parietal Epithelial Cell
PARP8	6.35866725127539e-120	0.44070055260066	0.27	0.137	1.83892656906884e-115	Parietal Epithelial Cell
MCC	1.70139488766887e-119	0.715745223785963	0.348	0.196	4.92043401513837e-115	Parietal Epithelial Cell
CEP152	6.9295398728761e-119	0.775856117815687	0.288	0.151	2.00402293123577e-114	Parietal Epithelial Cell
COL4A4	1.51524906215873e-118	0.547497072599677	0.52	0.341	4.38210028776306e-114	Parietal Epithelial Cell
CDC42BPB	2.46514748237698e-118	0.52054354344048	0.511	0.332	7.12920651903422e-114	Parietal Epithelial Cell
LPGAT1	8.68428269364653e-118	0.663308795011168	0.332	0.184	2.51149455500258e-113	Parietal Epithelial Cell
NHLRC2	1.08329188834505e-117	0.604594877520778	0.356	0.203	3.1328801410939e-113	Parietal Epithelial Cell
MYO19	1.14053951468802e-117	0.838078512815861	0.347	0.199	3.29844027647777e-113	Parietal Epithelial Cell
TLE4	5.5159483766213e-116	0.941596841601385	0.276	0.144	1.59521227051888e-111	Parietal Epithelial Cell
WRN	7.18133303900296e-116	0.788825361664227	0.34	0.192	2.07684151487966e-111	Parietal Epithelial Cell
EML4	8.05239004438348e-116	0.435821035549647	0.598	0.415	2.3287512008357e-111	Parietal Epithelial Cell
PHACTR2	9.04306845054469e-116	0.606962430694653	0.472	0.302	2.61525539589752e-111	Parietal Epithelial Cell
SLC38A1	1.30092401099172e-115	0.318158771373277	0.426	0.247	3.76227223978807e-111	Parietal Epithelial Cell
TFRC	2.40414258676924e-115	1.00707608327947	0.264	0.136	6.95278036093663e-111	Parietal Epithelial Cell
PKM	8.83971109718579e-115	0.720005854440775	0.359	0.208	2.55644444930613e-110	Parietal Epithelial Cell
LNPEP	2.71196242982746e-114	0.64533612293925	0.416	0.255	7.84299534706103e-110	Parietal Epithelial Cell
FOXN3	8.04955069817273e-114	0.509846200607653	0.568	0.407	2.32793006191155e-109	Parietal Epithelial Cell
SLC16A7	8.90084301616912e-114	0.568527224217417	0.506	0.331	2.57412380027611e-109	Parietal Epithelial Cell
SRSF5	1.09315436323379e-113	0.542459302314862	0.528	0.351	3.16140241847211e-109	Parietal Epithelial Cell
MACROD2	1.20630124033497e-113	0.527617954953131	0.714	0.55	3.48862318704873e-109	Parietal Epithelial Cell
LRRFIP2	1.78790605318982e-113	0.627252550028728	0.532	0.37	5.17062430582496e-109	Parietal Epithelial Cell
GPRIN3	1.93577939466885e-113	0.781236989685218	0.343	0.198	5.59827400938232e-109	Parietal Epithelial Cell
MYEF2	4.61625860833265e-113	0.757943790375286	0.32	0.179	1.3350219895298e-108	Parietal Epithelial Cell
TRIM2	6.13281928060495e-112	0.597040760552608	0.534	0.367	1.77361133595095e-107	Parietal Epithelial Cell
TBC1D12	1.29312472621394e-111	0.840531666768084	0.255	0.131	3.73971670821072e-107	Parietal Epithelial Cell
PDE7B	1.83791001986943e-111	0.438307626154535	0.641	0.437	5.3152357774624e-107	Parietal Epithelial Cell
IGFBP7	2.68414107250502e-111	0.577593757574457	0.671	0.49	7.76253598168453e-107	Parietal Epithelial Cell
GPATCH8	2.73516928223359e-111	0.489295875874351	0.62	0.45	7.91010956421956e-107	Parietal Epithelial Cell
FRY	3.44242226767825e-111	0.921019798322539	0.41	0.256	9.95548519812551e-107	Parietal Epithelial Cell
WDR1	4.2995468699103e-111	0.759514084742124	0.257	0.131	1.24342895477806e-106	Parietal Epithelial Cell
WARS2-AS1	2.43687235224214e-110	0.647980547799069	0.422	0.261	7.04743484268426e-106	Parietal Epithelial Cell
PACS1	2.75353872028962e-110	0.461919675529609	0.598	0.419	7.96323397907757e-106	Parietal Epithelial Cell
PISD	5.30813241911044e-110	0.63359531718729	0.386	0.232	1.53511189560674e-105	Parietal Epithelial Cell
YWHAQ	1.65587359622741e-109	0.667987522895266	0.317	0.177	4.78878644028968e-105	Parietal Epithelial Cell
CLEC16A	3.35390755163356e-109	0.65202118083162	0.528	0.363	9.69950063932427e-105	Parietal Epithelial Cell
AL008633.1	7.58754737131509e-109	0.669295270202919	0.391	0.235	2.19431869978432e-104	Parietal Epithelial Cell
LENG8	2.45532806377938e-108	0.618148725363582	0.433	0.27	7.10080876044997e-104	Parietal Epithelial Cell
FNBP1L	4.27578367951776e-107	0.606472816210287	0.657	0.513	1.23655664011654e-102	Parietal Epithelial Cell
FBXL2	1.09659244111972e-106	0.765735555161067	0.349	0.205	3.17134533971823e-102	Parietal Epithelial Cell
MTMR12	1.23620299746835e-106	0.771149046184322	0.338	0.197	3.57509906867846e-102	Parietal Epithelial Cell
LIFR	2.75350873441706e-106	0.412250534787502	0.567	0.39	7.96314725993414e-102	Parietal Epithelial Cell

gene	p_val	avg_log2FC	pct.1	pct.2	p_val_adj	cluster
NFKB1	3.63215087473261e-105	0.897293621546619	0.368	0.224	1.05041803297267e-100	Parietal Epithelial Cell
STRN	5.72635267591974e-105	0.568272906325617	0.421	0.266	1.65606119387599e-100	Parietal Epithelial Cell
OXCT1	1.88998690338637e-104	0.857822120056586	0.263	0.14	5.46584212459337e-100	Parietal Epithelial Cell
HADHB	2.29501771377354e-104	0.621630897417943	0.318	0.18	6.63719122823307e-100	Parietal Epithelial Cell
CPEB4	6.49809316298601e-104	0.339993160176656	0.675	0.502	1.87924854273555e-99	Parietal Epithelial Cell
SMARCA1	7.05946393733179e-104	0.646446652702343	0.294	0.162	2.04159697067635e-99	Parietal Epithelial Cell
PSME4	7.86478999348929e-104	0.625904155112138	0.487	0.329	2.2744972661171e-99	Parietal Epithelial Cell
ZMIZ1	1.40157727298884e-103	0.578174432330675	0.523	0.356	4.05336147348373e-99	Parietal Epithelial Cell
RNF217	6.76356555004699e-103	0.743672393756127	0.254	0.133	1.95602315707359e-98	Parietal Epithelial Cell
TNS3	2.33990996336859e-102	0.406645992292968	0.717	0.53	6.76701961406196e-98	Parietal Epithelial Cell
ATL2	2.78138208268069e-102	0.543875157900884	0.437	0.278	8.04375698311255e-98	Parietal Epithelial Cell
OSMR-AS1	1.19963973217409e-101	0.78767433206681	0.277	0.152	3.46935810544748e-97	Parietal Epithelial Cell
ANKS1A	8.89812304155656e-101	0.336139010253555	0.559	0.393	2.57333718361816e-96	Parietal Epithelial Cell
STK24	2.11437404083113e-100	0.553805741005508	0.462	0.304	6.11476972608364e-96	Parietal Epithelial Cell
NECTIN2	2.57019722511917e-100	0.726083222639675	0.306	0.173	7.43301037504465e-96	Parietal Epithelial Cell
SGMS1	1.8189735540419e-99	0.523682357515107	0.559	0.397	5.26047151828919e-95	Parietal Epithelial Cell
SMAD2	1.86645416818032e-99	0.5503261157997	0.496	0.334	5.39778545437748e-95	Parietal Epithelial Cell
PARP4	3.72694182696105e-99	0.431527332119003	0.321	0.181	1.07783157635714e-94	Parietal Epithelial Cell
CMSS1	1.19293162282552e-98	0.575538389253708	0.39	0.241	3.4499582532114e-94	Parietal Epithelial Cell
DYRK1A	6.40125442616694e-97	0.441322900818744	0.66	0.497	1.85124278004748e-92	Parietal Epithelial Cell
FAM120B	8.61899574842569e-97	0.449742258298933	0.575	0.41	2.49261357044471e-92	Parietal Epithelial Cell
FBXL17	9.82356728824752e-97	0.411985539874421	0.75	0.6	2.84097565976118e-92	Parietal Epithelial Cell
COG5	1.41355469172436e-96	0.437138974511013	0.571	0.407	4.08800016846685e-92	Parietal Epithelial Cell
PARVA	1.3138589614591e-95	0.605610264578627	0.384	0.237	3.7996801165397e-91	Parietal Epithelial Cell
RALA	1.37220563718119e-95	0.70150694382737	0.268	0.147	3.968418702728e-91	Parietal Epithelial Cell
GABARAPL1	1.49237562151059e-95	0.557909337690326	0.354	0.213	4.31595029740861e-91	Parietal Epithelial Cell
PRDM4	1.50450513176658e-95	0.64660215201818	0.254	0.136	4.35102884106895e-91	Parietal Epithelial Cell
RBFOX2	2.32587670698564e-95	0.445611556753647	0.59	0.427	6.72643543660247e-91	Parietal Epithelial Cell
BCAM	2.44345903292011e-95	0.981153693380388	0.261	0.145	7.06648352320496e-91	Parietal Epithelial Cell
WWP1	3.07891732858969e-95	0.547819873631065	0.551	0.4	8.90422891428137e-91	Parietal Epithelial Cell
ATP6V1H	7.38961626880089e-95	0.517440640324837	0.556	0.401	2.13707702493722e-90	Parietal Epithelial Cell
PRKCI	1.04665437777783e-94	0.826577549694177	0.36	0.224	3.0269244605335e-90	Parietal Epithelial Cell
SNX24	1.58420864872754e-94	0.553905997598029	0.454	0.303	4.58153141212004e-90	Parietal Epithelial Cell
WBP1L	1.84784039735609e-94	0.45926995379404	0.376	0.228	5.34395442915381e-90	Parietal Epithelial Cell
RALGAPA1	2.47189177453438e-94	0.419480212299443	0.666	0.506	7.14871101195342e-90	Parietal Epithelial Cell
AC079781.5	4.06486372565654e-94	0.563450225933846	0.344	0.205	1.17555858945987e-89	Parietal Epithelial Cell
PTK2	4.29321758905029e-94	0.362353284101295	0.802	0.647	1.24159852675334e-89	Parietal Epithelial Cell
GOLPH3	7.76969983818429e-94	0.560849940831879	0.373	0.232	2.2469971932029e-89	Parietal Epithelial Cell
NR3C2	8.04978221926493e-94	0.352673329056834	0.806	0.665	2.32799701781142e-89	Parietal Epithelial Cell
AL033504.1	8.72676913645902e-94	0.616620550618408	0.405	0.257	2.52378163426395e-89	Parietal Epithelial Cell
ANKH	8.78068309121316e-94	0.936255850286755	0.265	0.148	2.53937354997885e-89	Parietal Epithelial Cell
ADAM22	1.33005573289681e-93	0.704454597505471	0.291	0.165	3.84652117953759e-89	Parietal Epithelial Cell

gene	p_val	avg_log2FC	pct.1	pct.2	p_val_adj	cluster
GOT1	2.92620565696243e-93	0.734508174065111	0.396	0.257	8.46258675993536e-89	Parietal Epithelial Cell
MAN2A1	3.04250380302438e-93	0.519261315448297	0.494	0.339	8.79892099834649e-89	Parietal Epithelial Cell
TSPAN14	1.52713048354384e-92	0.501992787561494	0.28	0.155	4.41646135840878e-88	Parietal Epithelial Cell
SHANK2	2.20854798492329e-92	0.389101599863033	0.766	0.588	6.38712077239814e-88	Parietal Epithelial Cell
TRAF3	7.87797929853385e-92	0.51579053027808	0.312	0.18	2.27831161313599e-87	Parietal Epithelial Cell
CA12	8.11412800849442e-92	0.845140517982205	0.654	0.547	2.34660582005659e-87	Parietal Epithelial Cell
MROH1	8.82376531818026e-92	0.624019526175397	0.349	0.212	2.55183293001773e-87	Parietal Epithelial Cell
RHEB	1.73234928446104e-91	0.707454692537837	0.32	0.191	5.00995413066132e-87	Parietal Epithelial Cell
PLEKHB2	2.00567249896797e-91	0.721122179326796	0.273	0.154	5.80040486701538e-87	Parietal Epithelial Cell
CLCNKB	2.50053229796545e-91	0.456051774391341	0.493	0.33	7.23153940571608e-87	Parietal Epithelial Cell
SPTLC3	2.6589878764815e-91	0.399426883017481	0.421	0.265	7.68979293878451e-87	Parietal Epithelial Cell
NOVA1	3.65085338523529e-91	0.739079291375886	0.271	0.152	1.05582679901005e-86	Parietal Epithelial Cell
ALPK3	5.98542403286557e-91	0.723492890056835	0.256	0.14	1.73098463030472e-86	Parietal Epithelial Cell
AC138305.1	9.94560791867503e-91	0.680747672895781	0.56	0.425	2.87626981008082e-86	Parietal Epithelial Cell
AMMECR1	3.21961807820964e-90	0.64770562229986	0.273	0.151	9.31113548218227e-86	Parietal Epithelial Cell
WIPF1	1.09377392530166e-89	0.398915546021996	0.292	0.162	3.1631941919724e-85	Parietal Epithelial Cell
SLC43A2	2.98890624565326e-89	0.633472848286943	0.432	0.291	8.64391686242924e-85	Parietal Epithelial Cell
CAPZB	6.0387844563696e-89	0.48374451622376	0.491	0.337	1.74641646478209e-84	Parietal Epithelial Cell
TBL1XR1	2.76137813951983e-88	0.552233969073876	0.594	0.443	7.98590557949134e-84	Parietal Epithelial Cell
CDYL	4.96996700290636e-88	0.504460033761361	0.394	0.25	1.43731445724052e-83	Parietal Epithelial Cell
RERE	6.45119112877926e-88	0.33541720867011	0.845	0.729	1.86568447444296e-83	Parietal Epithelial Cell
BTAF1	1.27102997160051e-87	0.47644231497332	0.413	0.268	3.67581867786868e-83	Parietal Epithelial Cell
SIM1	1.29544048721377e-87	0.667201482448895	0.402	0.263	3.74641388902222e-83	Parietal Epithelial Cell
ARHGAP21	3.70716715952899e-87	0.438037770924859	0.453	0.3	1.07211274253578e-82	Parietal Epithelial Cell
XPR1	3.78739112898902e-87	0.432340631358476	0.551	0.397	1.09531351450362e-82	Parietal Epithelial Cell
PRIM2	7.93418763274683e-87	0.463503760613276	0.32	0.189	2.29456706339038e-82	Parietal Epithelial Cell
ARIH1	1.13955540432035e-86	0.49648705224159	0.615	0.469	3.29559422929445e-82	Parietal Epithelial Cell
SREBF2	1.2081119314491e-86	0.515343743670579	0.509	0.355	3.4938597057508e-82	Parietal Epithelial Cell
UBA6	2.0271609576831e-86	0.61621447547902	0.316	0.189	5.86254948961951e-82	Parietal Epithelial Cell
KIF3B	4.12745660560697e-86	0.581897460687158	0.3	0.175	1.19366045034153e-81	Parietal Epithelial Cell
ATP11A	5.98514670649503e-86	0.302311158887235	0.417	0.259	1.73090442751836e-81	Parietal Epithelial Cell
KLF7	7.4797591898998e-86	0.340119348148258	0.297	0.168	2.16314635771902e-81	Parietal Epithelial Cell
FAR1	3.07120312209705e-85	0.520490384547333	0.362	0.226	8.88191942910466e-81	Parietal Epithelial Cell
SUPT3H	7.69840150410068e-85	0.471823019903751	0.548	0.39	2.22637771498592e-80	Parietal Epithelial Cell
RIC1	9.73787160930503e-84	0.531112701427031	0.436	0.291	2.81619246941101e-79	Parietal Epithelial Cell
ZFAND6	1.18273402059989e-83	0.452721275357787	0.512	0.362	3.42046678757489e-79	Parietal Epithelial Cell
YPEL2	7.54527952563618e-83	0.759906642454241	0.313	0.189	2.18209483881398e-78	Parietal Epithelial Cell
LRCH3	2.73218040319412e-82	0.476822205685733	0.437	0.293	7.9014657260374e-78	Parietal Epithelial Cell
MRTFB	3.66244102657457e-82	0.438254342840309	0.548	0.394	1.05917794488537e-77	Parietal Epithelial Cell
RIC8B	1.60118372672553e-81	0.850098503641174	0.365	0.239	4.63062333769022e-77	Parietal Epithelial Cell
MKLN1	2.39595445554726e-81	0.403897826458703	0.689	0.547	6.92910028544266e-77	Parietal Epithelial Cell
RTN4	2.75495380686354e-81	0.309987027036083	0.513	0.359	7.96732640944936e-77	Parietal Epithelial Cell

gene	p_val	avg_log2FC	pct.1	pct.2	p_val_adj	cluster
IQGAP1	3.17394416593318e-81	0.501150795321419	0.432	0.289	9.17904652787875e-77	Parietal Epithelial Cell
ZNF608	3.97093849741234e-81	0.661542168998778	0.384	0.25	1.14839541345165e-76	Parietal Epithelial Cell
SAMD4A	4.11089147482726e-81	0.568192535310192	0.439	0.299	1.18886981452005e-76	Parietal Epithelial Cell
PSEN1	4.59837090525834e-81	0.481948052277577	0.362	0.228	1.32984886580071e-76	Parietal Epithelial Cell
RNF152	6.54678975762447e-81	0.667287547777528	0.602	0.479	1.893331597905e-76	Parietal Epithelial Cell
USP9X	8.58194522578727e-81	0.395316695435601	0.544	0.392	2.48189855929768e-76	Parietal Epithelial Cell
STX18	8.15141103199536e-80	0.443420056196369	0.443	0.299	2.35738807045306e-75	Parietal Epithelial Cell
MED27	9.25284877654541e-80	0.524348965211086	0.374	0.241	2.67592386617693e-75	Parietal Epithelial Cell
FNBP1	1.095507587195e-79	0.504334352084022	0.42	0.28	3.16820794216795e-75	Parietal Epithelial Cell
RPH3AL	1.40123187581504e-79	0.891650058342276	0.294	0.178	4.05236258485708e-75	Parietal Epithelial Cell
SIMC1	3.38204726476564e-79	0.557731432265857	0.286	0.167	9.78088068970222e-75	Parietal Epithelial Cell
TMLHE	4.3934616992328e-79	0.679235479891535	0.296	0.179	1.27058912341812e-74	Parietal Epithelial Cell
ZNF420	5.70251534552015e-79	0.584639324341702	0.288	0.171	1.64916743792443e-74	Parietal Epithelial Cell
LINC01138	2.15301875767356e-78	0.438019046527854	0.36	0.225	6.22653024719194e-74	Parietal Epithelial Cell
AKT3	2.4524758688661e-78	0.259516717971577	0.602	0.427	7.09256021276075e-74	Parietal Epithelial Cell
RHEX	2.63138352438746e-78	1.08340979378067	0.29	0.182	7.60996115252854e-74	Parietal Epithelial Cell
FAM160A1	3.5280210361323e-78	0.389583488662565	0.616	0.459	1.02030368364946e-73	Parietal Epithelial Cell
C1GALT1	3.69379574210083e-78	0.446967474187708	0.322	0.196	1.06824572861556e-73	Parietal Epithelial Cell
SAMD5	5.23367233496421e-78	0.481927504678928	0.498	0.352	1.51357803927165e-73	Parietal Epithelial Cell
PHKB	7.28494056707007e-78	0.42514855043177	0.535	0.387	2.10680481199666e-73	Parietal Epithelial Cell
KDM5B	1.19938268375223e-77	0.518301834384461	0.296	0.178	3.46861472141144e-73	Parietal Epithelial Cell
TMEM87A	1.4670184643123e-77	0.43599007467712	0.388	0.252	4.24261739879116e-73	Parietal Epithelial Cell
NPTN	3.22441668312379e-77	0.483984510587985	0.342	0.214	9.325013047594e-73	Parietal Epithelial Cell
CKB	3.51190005875697e-77	0.550478666705846	0.307	0.186	1.01564149699252e-72	Parietal Epithelial Cell
CCNI	3.52814215307541e-77	0.567120310549722	0.32	0.198	1.02033871066941e-72	Parietal Epithelial Cell
VCL	3.40397586038585e-76	0.473713042288958	0.384	0.25	9.84429818823587e-72	Parietal Epithelial Cell
MINDY2	4.4603500962019e-76	0.478562193224548	0.404	0.268	1.28993324782159e-71	Parietal Epithelial Cell
REEP3	5.85533225619599e-76	0.515216767410982	0.329	0.205	1.69336208849188e-71	Parietal Epithelial Cell
SPAG9	6.77859601863026e-76	0.358274664606772	0.642	0.491	1.96036996858787e-71	Parietal Epithelial Cell
PER1	6.94809834552935e-76	0.530330384273887	0.368	0.236	2.00939004152709e-71	Parietal Epithelial Cell
ZRANB2	7.73605827792137e-76	0.334607985983296	0.648	0.489	2.23726805397486e-71	Parietal Epithelial Cell
SCAI	1.0421337346047e-75	0.611234496517107	0.252	0.145	3.01385076047679e-71	Parietal Epithelial Cell
SRSF1	1.48479576491782e-75	0.544526958107504	0.307	0.188	4.29402935214233e-71	Parietal Epithelial Cell
ZNF532	1.05863794153914e-74	0.460305328972709	0.355	0.225	3.0615809269312e-70	Parietal Epithelial Cell
HOMER1	1.38117656234893e-74	1.29412518113755	0.292	0.181	3.9943626183131e-70	Parietal Epithelial Cell
CCNT2	3.69122773046413e-74	0.474946200939824	0.392	0.26	1.06750305965023e-69	Parietal Epithelial Cell
FUBP3	6.07451359966687e-74	0.489186357971631	0.3	0.183	1.75674933302366e-69	Parietal Epithelial Cell
RHBDD1	9.12845976933184e-74	0.452419264225039	0.422	0.284	2.63995056529077e-69	Parietal Epithelial Cell
GRAMD2B	1.01418133659078e-73	0.435516906214911	0.503	0.359	2.93301242542054e-69	Parietal Epithelial Cell
ZNF846	4.85658555724278e-73	0.810441298458572	0.275	0.168	1.40452454315461e-68	Parietal Epithelial Cell
FAM135A	7.01325826317658e-73	0.438015388558231	0.478	0.337	2.02823428971067e-68	Parietal Epithelial Cell
DENND4A	1.35404134605029e-72	0.380930386463915	0.522	0.374	3.91588757277743e-68	Parietal Epithelial Cell

gene	p_val	avg_log2FC	pct.1	pct.2	p_val_adj	cluster
NUP160	3.0459777489836e-72	0.473403563767027	0.318	0.198	8.80896765006058e-68	Parietal Epithelial Cell
REV1	3.48289670090331e-72	0.383766537621113	0.55	0.405	1.00725372590124e-67	Parietal Epithelial Cell
MYOF	4.24557591881942e-72	0.724534189050709	0.322	0.206	1.22782055572258e-67	Parietal Epithelial Cell
PRDM2	4.82028955472631e-72	0.386130776403062	0.352	0.224	1.39402773922685e-67	Parietal Epithelial Cell
NOS1AP	1.43978291752333e-71	0.423582711537484	0.479	0.334	4.16385219747747e-67	Parietal Epithelial Cell
THEM4	1.58607269703395e-71	0.52875965535299	0.275	0.165	4.58692223982217e-67	Parietal Epithelial Cell
MYO5B	1.89630651750634e-71	0.488909428963817	0.4	0.267	5.48411844862834e-67	Parietal Epithelial Cell
FOSL2	2.58966119500703e-71	0.792342303091808	0.28	0.171	7.48930017596034e-67	Parietal Epithelial Cell
CTDSPL	6.50254656843344e-71	0.291556793703498	0.558	0.402	1.88053646759095e-66	Parietal Epithelial Cell
NSD2	6.82860001490576e-71	0.385635876016354	0.38	0.248	1.97483112431074e-66	Parietal Epithelial Cell
TAF3	1.13544648901558e-70	0.439735870937003	0.413	0.279	3.28371124623306e-66	Parietal Epithelial Cell
TFCP2L1	3.16339242316037e-70	0.475612283365701	0.652	0.565	9.1485308877798e-66	Parietal Epithelial Cell
FBXW4	3.41269882464591e-70	0.475134873971294	0.387	0.257	9.86952500087598e-66	Parietal Epithelial Cell
B4GALNT3	8.47793948890177e-70	0.342533789503963	0.269	0.158	2.45182010019039e-65	Parietal Epithelial Cell
TRA2A	1.0436041491496e-69	0.363567947162789	0.546	0.397	3.01810319934064e-65	Parietal Epithelial Cell
MTSS1	1.30367396797476e-69	0.351408472068657	0.586	0.444	3.770225115383e-65	Parietal Epithelial Cell
KCTD20	1.32815073322017e-69	0.522436962178559	0.265	0.158	3.84101192047274e-65	Parietal Epithelial Cell
VGLL4	1.64173008523703e-69	0.378302756796492	0.542	0.395	4.74788340650549e-65	Parietal Epithelial Cell
MLLT6	2.04370950957595e-69	0.59302068934023	0.32	0.203	5.91040790169366e-65	Parietal Epithelial Cell
KATNBL1	3.39632352803382e-69	0.40908772556715	0.37	0.242	9.82216764307379e-65	Parietal Epithelial Cell
USO1	7.93593638762828e-69	0.425175800942352	0.38	0.254	2.2950728033021e-64	Parietal Epithelial Cell
AOPEP	8.12918056354475e-69	0.430709495423352	0.749	0.614	2.35095901897714e-64	Parietal Epithelial Cell
UGCG	1.54041152116064e-68	1.03888431446555	0.336	0.223	4.45487011919656e-64	Parietal Epithelial Cell
CDK5RAP2	2.00014851282603e-68	0.433232190741876	0.312	0.195	5.78442949909289e-64	Parietal Epithelial Cell
SLC22A15	2.04571876663909e-68	0.496812377180893	0.357	0.233	5.91621867312025e-64	Parietal Epithelial Cell
USP22	2.5406178351744e-68	0.440274656365784	0.361	0.235	7.34746677932438e-64	Parietal Epithelial Cell
SAMD4B	2.54322663566159e-68	0.42706099399067	0.289	0.177	7.35501143033333e-64	Parietal Epithelial Cell
DCAF6	2.78099452482801e-68	0.406732146431789	0.486	0.35	8.04263616580259e-64	Parietal Epithelial Cell
SFPQ	3.68753871788834e-68	0.33484011726245	0.577	0.425	1.06643619721331e-63	Parietal Epithelial Cell
RIMKLB	4.11287976102709e-68	0.383548215976568	0.312	0.194	1.18944482688903e-63	Parietal Epithelial Cell
ZNF431	4.49521648983979e-68	0.47991595999858	0.289	0.178	1.30001660886167e-63	Parietal Epithelial Cell
CPPED1	7.16751261655472e-68	0.407200377174174	0.347	0.223	2.07284464870763e-63	Parietal Epithelial Cell
GK5	9.49420468371472e-68	0.562470739466512	0.26	0.155	2.7457239945303e-63	Parietal Epithelial Cell
PCMTD2	1.21948784860702e-67	0.498697808291127	0.273	0.164	3.5267588581715e-63	Parietal Epithelial Cell
MECP2	1.41826283317276e-67	0.352052520813066	0.392	0.26	4.10161611353562e-63	Parietal Epithelial Cell
AC040174.1	1.43259065888068e-67	0.506069220421659	0.372	0.246	4.14305218548292e-63	Parietal Epithelial Cell
MBP	2.06458903408825e-67	0.459903078215478	0.404	0.276	5.97079148658322e-63	Parietal Epithelial Cell
CCDC50	4.73612008349745e-67	0.386510603676792	0.431	0.3	1.36968592814746e-62	Parietal Epithelial Cell
GMDS	4.78889196552322e-67	0.412516526911233	0.522	0.378	1.38494755642932e-62	Parietal Epithelial Cell
DIAPH1	8.55924391270693e-67	0.416771732574795	0.478	0.344	2.47533333955484e-62	Parietal Epithelial Cell
TM9SF3	8.90471630867429e-67	0.405841907822353	0.411	0.281	2.5752439564686e-62	Parietal Epithelial Cell
KIDINS220	1.4325157156601e-66	0.349407793694565	0.473	0.337	4.14283544968901e-62	Parietal Epithelial Cell

gene	p_val	avg_log2FC	pct.1	pct.2	p_val_adj	cluster
ASCC3	1.82834067471385e-66	0.328034231810446	0.477	0.335	5.28756123127244e-62	Parietal Epithelial Cell
VPS41	1.84925667788409e-66	0.53503969342937	0.404	0.28	5.3480503124408e-62	Parietal Epithelial Cell
TUSC3	2.42821957067041e-66	0.792480382895262	0.268	0.165	7.02241099837884e-62	Parietal Epithelial Cell
RNF213-AS1	2.70225235285336e-66	0.393983287288889	0.492	0.352	7.81491380445193e-62	Parietal Epithelial Cell
NRG3	3.30526933243442e-66	1.34469761657196	0.288	0.194	9.55883890940034e-62	Parietal Epithelial Cell
ZNF208	3.5863794290076e-66	0.419742627461742	0.303	0.188	1.037180930869e-61	Parietal Epithelial Cell
SEPTIN11	3.88028169443391e-66	0.425618614525639	0.404	0.272	1.12217746603029e-61	Parietal Epithelial Cell
PDZD8	6.24280370507195e-66	0.386987331935859	0.393	0.264	1.80541883150681e-61	Parietal Epithelial Cell
NF1	8.10812511557383e-66	0.32527012398824	0.71	0.565	2.34486978342395e-61	Parietal Epithelial Cell
PAFAH1B1	1.15796696611156e-65	0.326968006884194	0.615	0.473	3.34884046599464e-61	Parietal Epithelial Cell
KIF1B	1.93716458586173e-65	0.350698546756769	0.567	0.429	5.60227998231212e-61	Parietal Epithelial Cell
FOCAD	1.98364061846716e-65	0.424302422155246	0.446	0.313	5.73668866860703e-61	Parietal Epithelial Cell
ATP2B1	2.04554292961959e-65	0.469411395994821	0.41	0.281	5.91571015245984e-61	Parietal Epithelial Cell
PTBP2	2.83828032974708e-65	0.351400509708307	0.49	0.349	8.20830671362855e-61	Parietal Epithelial Cell
ASXL3	4.57555132348669e-65	0.499587628102439	0.308	0.195	1.32324944275235e-60	Parietal Epithelial Cell
TLE5	5.8925972501814e-65	0.549059191134216	0.264	0.159	1.70413912475246e-60	Parietal Epithelial Cell
STT3B	6.50160569722083e-65	0.423628840556009	0.388	0.262	1.88026436763626e-60	Parietal Epithelial Cell
AFDN	6.97542297251151e-65	0.387751739016235	0.704	0.561	2.01729232365033e-60	Parietal Epithelial Cell
KTN1	6.99294443783658e-65	0.275751574938903	0.638	0.486	2.02235953142234e-60	Parietal Epithelial Cell
NUP98	7.88450797874555e-65	0.359724919239538	0.409	0.277	2.28019970745321e-60	Parietal Epithelial Cell
PAPOLA	1.14733784392626e-64	0.387031667860759	0.44	0.309	3.31810104463474e-60	Parietal Epithelial Cell
STXBP3	1.9128407490839e-64	0.501625264796164	0.283	0.176	5.53193544635065e-60	Parietal Epithelial Cell
TP53BP1	2.13777846815841e-64	0.328241583995819	0.439	0.302	6.18245532991412e-60	Parietal Epithelial Cell
HECTD1	2.58931533412516e-64	0.257280840513443	0.565	0.418	7.48829994628995e-60	Parietal Epithelial Cell
DCBLD2	4.19647077066217e-64	0.600258543457451	0.327	0.214	1.2136193468755e-59	Parietal Epithelial Cell
MED13L	4.76172843327797e-64	0.285923747048423	0.779	0.643	1.37709186290399e-59	Parietal Epithelial Cell
PPM1B	5.31221659221288e-64	0.412238641233389	0.401	0.272	1.53629303846797e-59	Parietal Epithelial Cell
XRN2	5.47646604055852e-64	0.432812764327559	0.335	0.218	1.58379397892952e-59	Parietal Epithelial Cell
ELF1	7.4357365754561e-64	0.396435312534681	0.547	0.411	2.1504150176219e-59	Parietal Epithelial Cell
ECPAS	7.60780230730736e-64	0.302249128369982	0.514	0.374	2.20017642727329e-59	Parietal Epithelial Cell
SCML1	1.01715230509513e-63	0.434748584188529	0.3	0.188	2.9416044663351e-59	Parietal Epithelial Cell
FBXW11	1.22544055927515e-63	0.378219302811284	0.532	0.4	3.54397409742373e-59	Parietal Epithelial Cell
POGZ	2.62482950838279e-63	0.317554504549008	0.61	0.46	7.59100693824303e-59	Parietal Epithelial Cell
ILF3	6.13553377911513e-63	0.382479882318941	0.369	0.245	1.7743963689201e-58	Parietal Epithelial Cell
MNAT1	6.60161637947329e-63	0.294749026091905	0.588	0.442	1.90918745694367e-58	Parietal Epithelial Cell
CUEDC1	1.98386590585028e-62	0.531394132163723	0.266	0.164	5.73734019971902e-58	Parietal Epithelial Cell
RLF	2.02611013112523e-62	0.371730262163028	0.374	0.252	5.85951049921417e-58	Parietal Epithelial Cell
EPC2	3.9693239746151e-62	0.302831769154695	0.515	0.375	1.14792849345869e-57	Parietal Epithelial Cell
MARK3	6.0425019802014e-62	0.270091011018891	0.627	0.477	1.74749157267424e-57	Parietal Epithelial Cell
SETDB2	1.1073190591003e-61	0.417504316162813	0.3	0.19	3.20236671891806e-57	Parietal Epithelial Cell
DLG1	1.95480161105397e-61	0.308066878065762	0.676	0.537	5.65328625916809e-57	Parietal Epithelial Cell
EPN2	2.94457419997355e-61	0.367888705902836	0.409	0.281	8.51570858632352e-57	Parietal Epithelial Cell

gene	p_val	avg_log2FC	pct.1	pct.2	p_val_adj	cluster
RNGTT	4.18266201577053e-61	0.383961988742549	0.446	0.318	1.20962585496084e-56	Parietal Epithelial Cell
ST3GAL3	5.23400650663987e-61	0.469727690026487	0.322	0.209	1.51367468172025e-56	Parietal Epithelial Cell
ZSWIM5	1.87628465356145e-60	0.446657490041968	0.317	0.205	5.42621521809971e-56	Parietal Epithelial Cell
SLC35F5	2.77820740550257e-60	0.478293529716938	0.423	0.298	8.03457581671343e-56	Parietal Epithelial Cell
LAMC1	1.08021260078843e-59	0.552392008869636	0.283	0.18	3.12397484148013e-55	Parietal Epithelial Cell
ARAP2	2.64358225136438e-59	0.339181164650921	0.376	0.254	7.64523987094579e-55	Parietal Epithelial Cell
ARIH2	3.50908183286494e-59	0.358305452427733	0.353	0.235	1.01482646606454e-54	Parietal Epithelial Cell
CACUL1	4.18729438779463e-59	0.367544056117501	0.351	0.235	1.21096553695021e-54	Parietal Epithelial Cell
RALGPS1	4.44981406358778e-59	0.375618434872979	0.513	0.383	1.28688622718959e-54	Parietal Epithelial Cell
GTF2F2	5.38767848541203e-59	0.468185515469799	0.266	0.166	1.55811661798116e-54	Parietal Epithelial Cell
DNAJC3	6.72700679297354e-59	0.443535438067172	0.333	0.221	1.94545036452795e-54	Parietal Epithelial Cell
KLC1	8.63890875183495e-59	0.276090988203758	0.468	0.334	2.49837241103067e-54	Parietal Epithelial Cell
SCAF8	1.08009140237991e-58	0.339734805205856	0.535	0.4	3.12362433568271e-54	Parietal Epithelial Cell
DEPDC5	1.08622769599037e-58	0.4549593006682	0.276	0.174	3.14137049680416e-54	Parietal Epithelial Cell
INPP5A	1.16642389158481e-58	0.360431563385047	0.347	0.23	3.37329789446326e-54	Parietal Epithelial Cell
SGMS2	1.18849977845385e-58	0.483880146274161	0.272	0.17	3.43714135928852e-54	Parietal Epithelial Cell
HNF1B	1.19323067874232e-58	0.418628507017596	0.386	0.265	3.4508231229228e-54	Parietal Epithelial Cell
LINC00894	1.38311505002767e-58	0.359683605289215	0.481	0.348	3.99996872468001e-54	Parietal Epithelial Cell
TFEB	3.92794662196933e-58	0.484223294905108	0.255	0.157	1.13596216307353e-53	Parietal Epithelial Cell
HIVEP2	3.98132954801609e-58	0.475072331652333	0.495	0.363	1.15140050528625e-53	Parietal Epithelial Cell
JARID2	4.10065784710164e-58	0.379842186744353	0.49	0.364	1.18591024938179e-53	Parietal Epithelial Cell
KMT5B	9.52241152380076e-58	0.29857837869901	0.425	0.297	2.75388141268318e-53	Parietal Epithelial Cell
ATP8A1	9.75618689784096e-58	0.439383447208424	0.326	0.216	2.82148925085561e-53	Parietal Epithelial Cell
CD46	1.07059307498044e-57	0.283789567777688	0.408	0.281	3.09615517284343e-53	Parietal Epithelial Cell
SUN1	1.13142147698043e-57	0.356048602761054	0.345	0.23	3.27207091142739e-53	Parietal Epithelial Cell
SGK3	1.26509259109564e-57	0.332185516185484	0.478	0.349	3.65864777344859e-53	Parietal Epithelial Cell
GSPT1	1.67745206273356e-57	0.43781324146743	0.328	0.218	4.85119136542544e-53	Parietal Epithelial Cell
NASP	3.45284961347377e-57	0.494325514158719	0.279	0.176	9.98564108216615e-53	Parietal Epithelial Cell
SNX13	3.99947599761778e-57	0.330507993278821	0.437	0.312	1.15664845851106e-52	Parietal Epithelial Cell
PPP2R2A	4.15019841056221e-57	0.346884745757024	0.454	0.329	1.20023738033459e-52	Parietal Epithelial Cell
FEZ2	4.82081948814185e-57	0.363321744098332	0.345	0.23	1.39418099597062e-52	Parietal Epithelial Cell
UVRAG	5.24917725551131e-57	0.283426095196267	0.535	0.396	1.51806206229387e-52	Parietal Epithelial Cell
FAM27C	5.47486481050003e-57	0.489942349877772	0.275	0.174	1.58333090319661e-52	Parietal Epithelial Cell
YWHAZ	5.48621649924438e-57	0.409945215832811	0.412	0.292	1.58661381158147e-52	Parietal Epithelial Cell
ZNF236	7.17020181563077e-57	0.483802431686628	0.293	0.188	2.07362236508042e-52	Parietal Epithelial Cell
DENR	7.60723527793413e-57	0.478604759730507	0.265	0.166	2.20001244237855e-52	Parietal Epithelial Cell
KDM6A	8.38726704504166e-57	0.309388939253083	0.576	0.441	2.42559762942605e-52	Parietal Epithelial Cell
KDM1A	8.45568429926332e-57	0.489694344466681	0.254	0.157	2.44538389934695e-52	Parietal Epithelial Cell
SACM1L	1.36569769237914e-56	0.431899772581455	0.289	0.187	3.94959772636046e-52	Parietal Epithelial Cell
DNAH14	1.38686449209498e-56	0.285938879594705	0.649	0.496	4.01081211113868e-52	Parietal Epithelial Cell
MTR	1.45552879852544e-56	0.352300321651005	0.696	0.569	4.20938928533556e-52	Parietal Epithelial Cell
BCL2L13	3.90371851332117e-56	0.384043156265942	0.363	0.249	1.12895539405248e-51	Parietal Epithelial Cell

gene	p_val	avg_log2FC	pct.1	pct.2	p_val_adj	cluster
EIF3H	4.18175463790893e-56	0.446123742578706	0.287	0.185	1.20936344128326e-51	Parietal Epithelial Cell
PEX14	5.86739827689103e-56	0.445105821362004	0.375	0.258	1.69685158167689e-51	Parietal Epithelial Cell
EVI5	5.94483631904643e-56	0.29650707918902	0.555	0.416	1.71924666346823e-51	Parietal Epithelial Cell
PPM1L	1.30231795354571e-55	0.38952028324721	0.449	0.328	3.7663035216542e-51	Parietal Epithelial Cell
PRRG1	1.30603521313387e-55	0.461502031257996	0.269	0.17	3.77705383638314e-51	Parietal Epithelial Cell
TACC2	1.80506365230127e-55	0.379523133029673	0.498	0.366	5.22024408245527e-51	Parietal Epithelial Cell
TMEM120B	1.91497600253398e-55	0.435726076498393	0.327	0.218	5.53811059932827e-51	Parietal Epithelial Cell
gene	p_val	avg_log2FC	pct.1	pct.2	p_val_adj	cluster
DIDO1	2.39700815301728e-55	0.310697976979568	0.335	0.222	6.93214757852597e-51	Parietal Epithelial Cell
ZNF10	3.4064018828619e-55	0.372922234556228	0.331	0.222	9.8513142452366e-51	Parietal Epithelial Cell
DDX39B	3.68917004695236e-55	0.302595853404419	0.379	0.259	1.06690797757862e-50	Parietal Epithelial Cell
ESYT2	5.86326184670831e-55	0.430826518797279	0.447	0.323	1.69565532606804e-50	Parietal Epithelial Cell
FAM168B	1.38631047489587e-54	0.358989028319895	0.303	0.197	4.00920989339886e-50	Parietal Epithelial Cell
HLCS	2.04249871894782e-54	0.389119504903176	0.413	0.292	5.9069062951971e-50	Parietal Epithelial Cell
VRK2	2.23015122422832e-54	0.360700957160631	0.318	0.209	6.4495973404683e-50	Parietal Epithelial Cell
MIB1	2.40305175510821e-54	0.276443856642768	0.431	0.305	6.94962567577296e-50	Parietal Epithelial Cell
RSRC1	3.27399398978683e-54	0.270907284097791	0.471	0.342	9.4683906184635e-50	Parietal Epithelial Cell
PPP4R1	6.26048144376806e-54	0.411571657251367	0.302	0.199	1.81053123353772e-49	Parietal Epithelial Cell
LINC02256	6.52696449366101e-54	0.385668039102377	0.375	0.26	1.88759813156676e-49	Parietal Epithelial Cell
N4BP1	6.58849153865284e-54	0.320837542254125	0.337	0.226	1.9053917529784e-49	Parietal Epithelial Cell
TMCC1	7.19603765280458e-54	0.322056355881255	0.612	0.483	2.08109408919108e-49	Parietal Epithelial Cell
UHRF1BP1L	8.01448814820323e-54	0.388297521794477	0.254	0.158	2.31778997246037e-49	Parietal Epithelial Cell
LEMD3	9.78271986344169e-54	0.458642644171601	0.281	0.182	2.82916258450734e-49	Parietal Epithelial Cell
OSBPL9	1.77036900013e-53	0.274863180059918	0.535	0.402	5.11990714837595e-49	Parietal Epithelial Cell
LCOR	2.52647114885842e-53	0.309326357158817	0.564	0.434	7.30655456249856e-49	Parietal Epithelial Cell
PTPN9	2.57433495883664e-53	0.348242638149234	0.3	0.195	7.44497670095557e-49	Parietal Epithelial Cell
POLA1	4.50395850730124e-53	0.443282272920481	0.267	0.17	1.30254480031152e-48	Parietal Epithelial Cell
MRPS28	5.99160766542274e-53	0.399432744179413	0.297	0.194	1.73277293684026e-48	Parietal Epithelial Cell
GRAMD1C	9.71838195020291e-53	0.385283700324258	0.339	0.23	2.81055605999868e-48	Parietal Epithelial Cell
NCOA2	1.09058116231033e-52	0.34262762024874	0.675	0.558	3.15396072140149e-48	Parietal Epithelial Cell
QKI	1.69196662544947e-52	0.295021822353211	0.617	0.488	4.89316748079988e-48	Parietal Epithelial Cell
KPNA1	1.96779857642144e-52	0.310858352885218	0.334	0.224	5.69087348301079e-48	Parietal Epithelial Cell
PAWR	2.842425991702e-52	0.860917210234082	0.441	0.335	8.22029596800218e-48	Parietal Epithelial Cell
TMBIM4	3.18042610488127e-52	0.407630438143709	0.298	0.196	9.19779229531664e-48	Parietal Epithelial Cell
IQCK	3.43176708115936e-52	0.399151310581174	0.329	0.22	9.92467039871287e-48	Parietal Epithelial Cell
ACACA	3.89496568065518e-52	0.334524794971225	0.412	0.291	1.12642407484548e-47	Parietal Epithelial Cell
RNF38	4.4873852670858e-52	0.273767907621268	0.535	0.407	1.29775181924121e-47	Parietal Epithelial Cell
CDK8	6.35216458114611e-52	0.334649254095235	0.494	0.372	1.83704599686746e-47	Parietal Epithelial Cell
HYDIN	1.02976510509932e-51	0.560038189521926	0.285	0.189	2.97808068394724e-47	Parietal Epithelial Cell
TLK2	1.03381029666301e-51	0.303123182825932	0.43	0.309	2.98977937794942e-47	Parietal Epithelial Cell
FRK	1.09510755088528e-51	0.367023944945022	0.44	0.324	3.16705103716023e-47	Parietal Epithelial Cell
METTL16	1.12446112118082e-51	0.362102837886439	0.298	0.195	3.25194156245492e-47	Parietal Epithelial Cell

gene	p_val	avg_log2FC	pct.1	pct.2	p_val_adj	cluster
USP7	1.25745597451326e-51	0.398680530462156	0.258	0.164	3.63656267829234e-47	Parietal Epithelial Cell
CPT1A	1.36613961359442e-51	0.393798784455818	0.389	0.277	3.95087576251505e-47	Parietal Epithelial Cell
DPP6	1.38121684870564e-51	0.567240294078394	0.387	0.278	3.9944791264567e-47	Parietal Epithelial Cell
MPC1	1.3897987688394e-51	0.38501661335553	0.463	0.342	4.01929803948354e-47	Parietal Epithelial Cell
NBAS	2.24067868375534e-51	0.302040384456913	0.526	0.394	6.48004275342045e-47	Parietal Epithelial Cell
STRBP	2.41052226712533e-51	0.33367326144358	0.462	0.34	6.97123039652647e-47	Parietal Epithelial Cell
SORL1	2.45249799674429e-51	0.28187633249618	0.293	0.19	7.09262420658449e-47	Parietal Epithelial Cell
THOC1	3.71948955425588e-51	0.351118010179094	0.3	0.198	1.0756763790908e-46	Parietal Epithelial Cell
RAI1	4.95974829872246e-51	0.520814809434585	0.273	0.177	1.43435920799053e-46	Parietal Epithelial Cell
STX8	8.8446696359084e-51	0.291738957891641	0.516	0.388	2.55787845870471e-46	Parietal Epithelial Cell
PPP4R2	9.61896232417619e-51	0.337751196840463	0.326	0.22	2.78180390415176e-46	Parietal Epithelial Cell
PCNX2	1.06365741384024e-50	0.337809968011744	0.386	0.271	3.07609724082597e-46	Parietal Epithelial Cell
KAZN	1.09852889370078e-50	0.262463804131197	0.723	0.567	3.17694556058265e-46	Parietal Epithelial Cell
CTTN	1.23624805104477e-50	0.336254516930837	0.303	0.2	3.57522936362148e-46	Parietal Epithelial Cell
PPP2CA	1.56532912484322e-50	0.409288493090557	0.27	0.175	4.5269318290466e-46	Parietal Epithelial Cell
FUS	1.68687789256774e-50	0.289302395012634	0.443	0.319	4.8784508653059e-46	Parietal Epithelial Cell
MAP2K4	1.79524462910666e-50	0.304726344022008	0.465	0.342	5.19184746737647e-46	Parietal Epithelial Cell
STIM2	3.10262149235803e-50	0.256707120693685	0.333	0.225	8.97278135589941e-46	Parietal Epithelial Cell
PWWP2A	4.0087381609052e-50	0.409060265764189	0.284	0.188	1.15932707613378e-45	Parietal Epithelial Cell
CD24	4.15775690026847e-50	0.482368132423767	0.272	0.178	1.20242329555764e-45	Parietal Epithelial Cell
ANKRD10	4.87400459407504e-50	0.315676044188988	0.434	0.316	1.4095621286065e-45	Parietal Epithelial Cell
CUL5	5.36570381033718e-50	0.296671621525794	0.459	0.336	1.55176154194951e-45	Parietal Epithelial Cell
CNOT4	5.70606018913259e-50	0.253679808442918	0.494	0.368	1.65019260669714e-45	Parietal Epithelial Cell
FARS2	5.91429418658526e-50	0.274882685367408	0.58	0.449	1.71041387876046e-45	Parietal Epithelial Cell
MLXIP	6.59488862111745e-50	0.409605939704824	0.289	0.191	1.90724178922717e-45	Parietal Epithelial Cell
CPQ	6.70365187063015e-50	0.266605709554792	0.608	0.478	1.93869612098624e-45	Parietal Epithelial Cell
DNAJB6	7.04861518222073e-50	0.345897836025227	0.319	0.215	2.03845951069823e-45	Parietal Epithelial Cell
ATP11B	7.1614038823751e-50	0.300922983223378	0.388	0.272	2.07107800278288e-45	Parietal Epithelial Cell
KDM3A	7.86655134987564e-50	0.447947817399772	0.25	0.16	2.27500665038403e-45	Parietal Epithelial Cell
ATG5	1.408994796666e-49	0.364704366879726	0.347	0.241	4.07481295195806e-45	Parietal Epithelial Cell
FGFR2	1.53143005480447e-49	0.499449739543991	0.297	0.199	4.42889571849451e-45	Parietal Epithelial Cell
MTF2	1.53445645364024e-49	0.328597912867065	0.355	0.245	4.43764806392758e-45	Parietal Epithelial Cell
DLGAP4	2.21177329982268e-49	0.361245387469209	0.273	0.178	6.3964483830872e-45	Parietal Epithelial Cell
GOLGA8B	2.31592419787151e-49	0.387135304731735	0.347	0.24	6.6976527802444e-45	Parietal Epithelial Cell
ROCK2	2.62710262043481e-49	0.274882147764569	0.527	0.398	7.59758077829748e-45	Parietal Epithelial Cell
GTF2IRD2B	2.75568662952944e-49	0.358519654912693	0.282	0.185	7.96944573259915e-45	Parietal Epithelial Cell
ATF2	3.54069608915077e-49	0.329960014219875	0.304	0.203	1.0239693089824e-44	Parietal Epithelial Cell
WWP2	4.185664538497e-49	0.264671789953129	0.465	0.34	1.21049418453333e-44	Parietal Epithelial Cell
FAM189A1	6.55814181428731e-49	1.15168949449285	0.26	0.18	1.89661461269189e-44	Parietal Epithelial Cell
MUC1	8.82055889522852e-49	0.38330610183035	0.272	0.177	2.55090563250009e-44	Parietal Epithelial Cell
SDHA	1.12234891277815e-48	0.388516057548602	0.323	0.222	3.24583305575442e-44	Parietal Epithelial Cell
PPP3CB	1.17554951866984e-48	0.272295343105465	0.367	0.255	3.39968920799318e-44	Parietal Epithelial Cell

gene	p_val	avg_log2FC	pct.1	pct.2	p_val_adj	cluster
RNF130	1.29350055041445e-48	0.262808066629561	0.498	0.371	3.74080359179858e-44	Parietal Epithelial Cell
SCLT1	1.34317330528587e-48	0.295899389068982	0.413	0.296	3.88445719888674e-44	Parietal Epithelial Cell
RBFOX1	1.97682619208512e-48	1.51788249595091	0.312	0.234	5.71698134751016e-44	Parietal Epithelial Cell
ESRRG	2.20644339839143e-48	0.300426766326992	0.873	0.822	6.38103430814802e-44	Parietal Epithelial Cell
DAB2IP	2.63130644145196e-48	0.368866400500393	0.31	0.209	7.60973822867908e-44	Parietal Epithelial Cell
CBFB	2.91716741251553e-48	0.297639066104637	0.366	0.255	8.43644815699492e-44	Parietal Epithelial Cell
CHP1	4.71622201970315e-48	0.349661228763115	0.29	0.192	1.36393140809815e-43	Parietal Epithelial Cell
PDHX	6.68098028492295e-48	0.371004390439551	0.313	0.213	1.93213949839972e-43	Parietal Epithelial Cell
SMG7	7.19285068880219e-48	0.340353150449432	0.384	0.272	2.08017241920159e-43	Parietal Epithelial Cell
STX17	2.03887918139212e-47	0.433636021373741	0.262	0.172	5.89643859258602e-43	Parietal Epithelial Cell
SUZ12	2.39869628910489e-47	0.284639360999061	0.32	0.217	6.93702966809134e-43	Parietal Epithelial Cell
MT-ND6	4.8925287591781e-47	0.323854526094074	0.267	0.176	1.41491931715431e-42	Parietal Epithelial Cell
SLAIN2	5.88295574702622e-47	0.314488046362356	0.299	0.201	1.70135080203998e-42	Parietal Epithelial Cell
SASH1	5.9055103624653e-47	0.323985899502985	0.51	0.378	1.70787359682497e-42	Parietal Epithelial Cell
IRF3	5.95647840763356e-47	0.366822472424425	0.271	0.177	1.72261355548762e-42	Parietal Epithelial Cell
USP16	1.36162601108349e-46	0.379084549206805	0.256	0.165	3.93782242405346e-42	Parietal Epithelial Cell
TM9SF2	2.31016697795844e-46	0.387154867244366	0.263	0.173	6.6810029002558e-42	Parietal Epithelial Cell
CLYBL	3.00088049008788e-46	0.339827964952164	0.467	0.353	8.67854637733414e-42	Parietal Epithelial Cell
CEP192	3.71308556056845e-46	0.282985456239995	0.398	0.286	1.0738243441164e-41	Parietal Epithelial Cell
RSRC2	7.76137901814972e-46	0.300244635067364	0.333	0.23	2.2445908120489e-41	Parietal Epithelial Cell
C20orf194	8.33683225118473e-46	0.252410438207221	0.699	0.56	2.41101188704262e-41	Parietal Epithelial Cell
ELF2	1.08328627778844e-45	0.254470505089093	0.554	0.43	3.13286391536416e-41	Parietal Epithelial Cell
TFG	1.23264177189212e-45	0.375391845935007	0.305	0.208	3.564800004312e-41	Parietal Epithelial Cell
PPP2R1B	2.546384702221e-45	0.338746432359071	0.268	0.176	7.36414455882314e-41	Parietal Epithelial Cell
ZCCHC14	2.86178316121282e-45	0.284679996810526	0.357	0.251	8.27627690222747e-41	Parietal Epithelial Cell
ZHX2	2.96707338472858e-45	0.31978905963032	0.508	0.386	8.58077622863505e-41	Parietal Epithelial Cell
TTC28-AS1	3.14426864120266e-45	0.449170132277173	0.272	0.181	9.09322491035809e-41	Parietal Epithelial Cell
SF1	4.12520789530423e-45	0.309378291411429	0.355	0.251	1.19301012332198e-40	Parietal Epithelial Cell
EIF2AK3	4.788012442379e-45	0.498951343355722	0.263	0.174	1.38469319833601e-40	Parietal Epithelial Cell
ZCCHC7	5.10165789704329e-45	0.250706239050896	0.636	0.514	1.47539946382492e-40	Parietal Epithelial Cell
TRAPPC6B	1.17502406866078e-44	0.395436028360661	0.25	0.163	3.39816960656698e-40	Parietal Epithelial Cell
USP3	1.28337979806372e-44	0.347905307506607	0.451	0.342	3.71153437600028e-40	Parietal Epithelial Cell
PDE9A	1.36600714732576e-44	0.360042668103472	0.337	0.236	3.95049267006611e-40	Parietal Epithelial Cell
MTREX	1.57869308915918e-44	0.282602844732468	0.331	0.229	4.56558041384836e-40	Parietal Epithelial Cell
ZFX	2.0880200116152e-44	0.349652414866605	0.302	0.205	6.03855387359117e-40	Parietal Epithelial Cell
TMEM165	2.38149646414337e-44	0.461458446239718	0.354	0.254	6.88728777430263e-40	Parietal Epithelial Cell
TIAM2	2.51460901075702e-44	0.291574483574056	0.401	0.29	7.27224925910931e-40	Parietal Epithelial Cell
FBXW7	3.05320595134836e-44	0.271758461703203	0.482	0.364	8.82987161129944e-40	Parietal Epithelial Cell
PPIL2	3.11701891417647e-44	0.424627269704084	0.254	0.167	9.01441869979834e-40	Parietal Epithelial Cell
ZNF277	4.79874002521776e-44	0.282393206167259	0.386	0.278	1.38779561529298e-39	Parietal Epithelial Cell
UBE4B	8.98035035130384e-44	0.281638053557121	0.425	0.311	2.59711732159707e-39	Parietal Epithelial Cell
WASL	1.18352269054173e-43	0.301911197598922	0.327	0.228	3.42274762104667e-39	Parietal Epithelial Cell

gene	p_val	avg_log2FC	pct.1	pct.2	p_val_adj	cluster
ZNF148	2.9585452473439e-43	0.328365140291832	0.571	0.45	8.55611285531856e-39	Parietal Epithelial Cell
R3HCC1L	3.46184909208067e-43	0.317147381896732	0.298	0.204	1.00116675742973e-38	Parietal Epithelial Cell
SEC23A	3.55333112912022e-43	0.303459680567243	0.333	0.235	1.02762336254157e-38	Parietal Epithelial Cell
LINC01572	5.36241109781644e-43	0.271473578393313	0.258	0.169	1.55080928948851e-38	Parietal Epithelial Cell
BICD1	5.57558021759535e-43	1.33169250390564	0.372	0.306	1.61245779892857e-38	Parietal Epithelial Cell
RAD23B	1.74465430317175e-42	0.357802652507786	0.311	0.215	5.04554024477271e-38	Parietal Epithelial Cell
BRWD3	1.8551032740159e-42	0.297052796743481	0.287	0.196	5.36495866845397e-38	Parietal Epithelial Cell
ZNF131	1.87657936257264e-42	0.260794752210417	0.395	0.286	5.42706751656007e-38	Parietal Epithelial Cell
ZDHHC17	1.93316351859973e-42	0.28585261658987	0.318	0.22	5.59070889579042e-38	Parietal Epithelial Cell
ANKRD13A	2.21402722043883e-42	0.325607776892041	0.26	0.172	6.40296672150911e-38	Parietal Epithelial Cell
HNRNPD	2.61073210275319e-42	0.281897297254843	0.374	0.269	7.55023724116222e-38	Parietal Epithelial Cell
SIN3A	3.8901509758506e-42	0.287663900875836	0.268	0.179	1.12503166221599e-37	Parietal Epithelial Cell
DAAM1	4.4213931099856e-42	0.259992814538562	0.387	0.279	1.27866688740784e-37	Parietal Epithelial Cell
FAM117B	4.64904824604597e-42	0.321901804093979	0.291	0.197	1.34450475275649e-37	Parietal Epithelial Cell
AC079142.1	8.60680241121595e-42	0.395853212606808	0.459	0.353	2.48908725732365e-37	Parietal Epithelial Cell
PRKCE	1.29021864968674e-41	0.284559418327553	0.626	0.519	3.73131233489405e-37	Parietal Epithelial Cell
OBSCN	1.43604264820853e-41	0.423049041293726	0.283	0.195	4.15303533861906e-37	Parietal Epithelial Cell
HECTD2	2.31392328141725e-41	0.412479801460245	0.277	0.189	6.69186612985868e-37	Parietal Epithelial Cell
AHNAK	8.41857969004772e-41	0.259593978820046	0.328	0.231	2.4346532463618e-36	Parietal Epithelial Cell
PITPNA	8.89862853595735e-41	0.376341263797195	0.287	0.197	2.57348337259886e-36	Parietal Epithelial Cell
MAPKAPK2	1.31335397109682e-40	0.345771858843689	0.273	0.185	3.798219684412e-36	Parietal Epithelial Cell
WDR26	1.66626109011359e-40	0.28476004250227	0.351	0.251	4.81882707260851e-36	Parietal Epithelial Cell
CRTC1	1.95974777507966e-40	0.359224746921177	0.303	0.21	5.66759056553039e-36	Parietal Epithelial Cell
SUSD6	2.46467889710028e-40	0.306967449479287	0.339	0.24	7.127851370414e-36	Parietal Epithelial Cell
LINC01004	3.09319876315019e-40	0.283572891018893	0.399	0.294	8.94553082303034e-36	Parietal Epithelial Cell
CLCNKA	4.12327342644123e-40	0.289885339734595	0.324	0.226	1.1924506749268e-35	Parietal Epithelial Cell
MORC3	4.14866471153201e-40	0.253290102943086	0.303	0.21	1.19979383457506e-35	Parietal Epithelial Cell
BCL9	5.16010220599513e-40	0.374047558246116	0.254	0.171	1.49230155797379e-35	Parietal Epithelial Cell
TMEM168	6.86897205009395e-40	0.34690904932905	0.254	0.17	1.98650671688717e-35	Parietal Epithelial Cell
SEC11A	1.04882033842545e-39	0.255021797763285	0.284	0.194	3.03318841872639e-35	Parietal Epithelial Cell
AC021231.1	1.84438449012161e-39	0.38678066775815	0.261	0.176	5.33395994543169e-35	Parietal Epithelial Cell
CHIC2	1.97771570963596e-39	0.458720283979446	0.305	0.215	5.71955383226721e-35	Parietal Epithelial Cell
ZNF875	2.25059943014734e-39	0.267648252310347	0.255	0.171	6.50873355198612e-35	Parietal Epithelial Cell
AAGAB	2.59381757655567e-39	0.277199073666446	0.279	0.19	7.50132043139901e-35	Parietal Epithelial Cell
GLUD1	3.18876724279782e-39	0.265880428484708	0.339	0.242	9.2219148661713e-35	Parietal Epithelial Cell
WTAP	5.9265227751923e-39	0.384716838994967	0.275	0.189	1.71395038658561e-34	Parietal Epithelial Cell
CAPZA2	7.48717030659371e-39	0.31225595317868	0.283	0.196	2.1652896526669e-34	Parietal Epithelial Cell
CALM2	1.03952405689296e-38	0.399867805607969	0.253	0.17	3.00630357253444e-34	Parietal Epithelial Cell
HDLBP	1.36128833655827e-38	0.260403169946473	0.345	0.247	3.93684586932652e-34	Parietal Epithelial Cell
MIPOL1	1.50471046506672e-38	0.286671275003615	0.408	0.301	4.35162266497297e-34	Parietal Epithelial Cell
IFT43	4.30103976084604e-38	0.260604316790631	0.341	0.244	1.24386069883667e-33	Parietal Epithelial Cell
TMEM51	4.76865905343335e-38	0.363775419508107	0.388	0.289	1.37909619825292e-33	Parietal Epithelial Cell

gene	p_val	avg_log2FC	pct.1	pct.2	p_val_adj	cluster
AL158152.1	4.95640605310163e-38	0.434241825336465	0.275	0.191	1.43339263055699e-33	Parietal Epithelial Cell
CCDC18-AS1	5.77849753745373e-38	0.251428302238613	0.502	0.384	1.67114148783162e-33	Parietal Epithelial Cell
SCNN1A	4.2117691748306e-37	0.299687371955199	0.438	0.334	1.21804364536101e-32	Parietal Epithelial Cell
RGL3	7.01054776721708e-37	0.309119091203971	0.448	0.346	2.02745041427918e-32	Parietal Epithelial Cell
AGO2	7.43520296724368e-37	0.258514007570954	0.316	0.225	2.15026069812687e-32	Parietal Epithelial Cell
HOXB3	1.0336192859679e-36	0.273790923351918	0.352	0.255	2.98922697501917e-32	Parietal Epithelial Cell
IMMT	2.81964599125217e-36	0.304606477372933	0.292	0.205	8.15441620670127e-32	Parietal Epithelial Cell
ADAM10	2.88773515900644e-36	0.35444141956543	0.485	0.375	8.35133007984664e-32	Parietal Epithelial Cell
KPNA4	3.34296528029773e-36	0.276386160711789	0.338	0.246	9.66785559062104e-32	Parietal Epithelial Cell
RAPGEF3	3.35576621055953e-36	0.823846321839885	0.331	0.249	9.70487588093817e-32	Parietal Epithelial Cell
TANK	3.44826992588867e-36	0.316491090173451	0.344	0.252	9.97239662567003e-32	Parietal Epithelial Cell
NUP153	3.92996522528968e-36	0.288081444568859	0.317	0.227	1.13654594315377e-31	Parietal Epithelial Cell
FRMD4A	3.94204664823243e-36	2.12614506515199	0.351	0.322	1.14003989066882e-31	Parietal Epithelial Cell
AGFG1	4.09412324377522e-36	0.288549276927947	0.383	0.287	1.18402044209979e-31	Parietal Epithelial Cell
ANXA11	4.57964560318185e-36	0.305950847277037	0.355	0.259	1.32443350844019e-31	Parietal Epithelial Cell
TVP23C	4.7645856239667e-36	0.295817824566165	0.355	0.26	1.37791816245117e-31	Parietal Epithelial Cell
ENSA	8.92472280643024e-36	0.2805244231097	0.369	0.273	2.58102983561962e-31	Parietal Epithelial Cell
DLG5	1.06180727082023e-35	0.326510152299988	0.259	0.178	3.07074662721209e-31	Parietal Epithelial Cell
CAST	1.6244630076756e-35	0.252310364369817	0.45	0.343	4.69794701819782e-31	Parietal Epithelial Cell
SUCO	1.66540810019724e-35	0.31133877334611	0.262	0.181	4.81636022577043e-31	Parietal Epithelial Cell
PARD6B	1.87294320349712e-35	0.331088954548451	0.332	0.242	5.41655174451368e-31	Parietal Epithelial Cell
MTOR	2.35538398443265e-35	0.251394548140221	0.292	0.205	6.81177048297923e-31	Parietal Epithelial Cell
AUTS2	2.60654603214898e-35	0.57208500967328	0.507	0.416	7.53813112497485e-31	Parietal Epithelial Cell
KLHL22	4.74652582120153e-35	0.342063334616327	0.274	0.193	1.37269526749148e-30	Parietal Epithelial Cell
MRPL1	6.4026379994221e-35	0.328110727306187	0.262	0.181	1.85164290943287e-30	Parietal Epithelial Cell
AC002460.2	7.79087402827664e-35	0.458395362510813	0.349	0.261	2.2531207689776e-30	Parietal Epithelial Cell
SULT1C2	9.04974913899829e-35	0.272477228325094	0.636	0.521	2.61718745099831e-30	Parietal Epithelial Cell
ZNF544	1.14455019392328e-34	0.271849332013753	0.34	0.248	3.31003916082614e-30	Parietal Epithelial Cell
TBC1D16	1.32565780932734e-34	0.308908057214454	0.257	0.177	3.83380238457467e-30	Parietal Epithelial Cell
YME1L1	1.46341028520411e-34	0.27171746361607	0.278	0.195	4.23218254481029e-30	Parietal Epithelial Cell
HGSNAT	2.00154391271662e-34	0.312247546850837	0.313	0.225	5.78846499557645e-30	Parietal Epithelial Cell
MAPK1IP1L	2.4333802076469e-34	0.312417444184582	0.283	0.199	7.03733556051485e-30	Parietal Epithelial Cell
TULP4	2.90744697460963e-34	0.273253807337576	0.338	0.248	8.40833665057106e-30	Parietal Epithelial Cell
GABPB2	4.83234576659365e-34	0.30439487407407	0.297	0.212	1.39751439569888e-29	Parietal Epithelial Cell
DAB1	8.33105670310907e-34	0.417917855622122	0.44	0.338	2.40934159853914e-29	Parietal Epithelial Cell
CARS2	1.83632967737315e-33	0.294257755649882	0.253	0.175	5.31066542696314e-29	Parietal Epithelial Cell
RANBP9	2.41101674412453e-33	0.307476984176557	0.333	0.245	6.97266042400815e-29	Parietal Epithelial Cell
HIF1A	8.92779142158287e-33	0.340437297166351	0.455	0.353	2.58191727912177e-28	Parietal Epithelial Cell
SIK2	9.11060468383117e-33	0.4260921687305	0.617	0.505	2.63478687456397e-28	Parietal Epithelial Cell
DYNC2LI1	1.02500238837489e-32	0.478329372206646	0.251	0.178	2.96430690718019e-28	Parietal Epithelial Cell
DCAF10	1.21103783599589e-32	0.276535837173076	0.341	0.252	3.50232142170012e-28	Parietal Epithelial Cell
SARNP	1.9602030926953e-32	0.254436043797201	0.31	0.225	5.66890734407481e-28	Parietal Epithelial Cell

gene	p_val	avg_log2FC	pct.1	pct.2	p_val_adj	cluster
ORC4	1.98748750327417e-32	0.25710073326396	0.323	0.236	5.7478138594689e-28	Parietal Epithelial Cell
TTC27	2.56506628097666e-32	0.298312389716813	0.253	0.176	7.41817168458449e-28	Parietal Epithelial Cell
MTRNR2L12	2.75581569271499e-32	0.310428937387922	0.485	0.394	7.96981898333175e-28	Parietal Epithelial Cell
STPG2	5.4715624178672e-32	0.272438367639078	0.491	0.394	1.58237585124719e-27	Parietal Epithelial Cell
KHDRBS1	7.20899848686483e-32	0.268532777214055	0.305	0.221	2.08484236240131e-27	Parietal Epithelial Cell
MYO5C	1.09835132201002e-31	0.32283549288376	0.261	0.183	3.17643202325297e-27	Parietal Epithelial Cell
UQCC1	2.48486548610379e-30	0.254388116754182	0.305	0.223	7.18623098581217e-26	Parietal Epithelial Cell
MICU3	2.56014461826107e-30	0.336396079373695	0.262	0.186	7.403938236011e-26	Parietal Epithelial Cell
MAD1L1	3.31503307915061e-30	0.443060250169944	0.254	0.18	9.58707566490355e-26	Parietal Epithelial Cell
GTDC1	1.06281582530432e-29	0.365771649586243	0.29	0.214	3.0736633667801e-25	Parietal Epithelial Cell
PTPN1	1.4328362343525e-29	0.834129747409227	0.352	0.272	4.14376238974743e-25	Parietal Epithelial Cell
CPSF6	4.29090079387611e-28	0.269965145312128	0.356	0.271	1.24092850958897e-23	Parietal Epithelial Cell
GALNT7	4.97865162104291e-28	0.27388624340164	0.272	0.198	1.43982604880561e-23	Parietal Epithelial Cell
CAND1	5.54480902056215e-28	0.252113111222699	0.27	0.196	1.60355876874657e-23	Parietal Epithelial Cell
ARHGAP29	3.64353335889907e-27	0.450533310229658	0.491	0.405	1.05370984739361e-22	Parietal Epithelial Cell
PIK3CA	7.95807767874282e-27	0.267698776677359	0.273	0.2	2.30147606469242e-22	Parietal Epithelial Cell
Z93930.2	8.33352030295967e-27	0.258515043242054	0.272	0.199	2.41005407161594e-22	Parietal Epithelial Cell
GPCPD1	3.94696958628187e-26	0.268973927625019	0.323	0.245	1.14146360435272e-21	Parietal Epithelial Cell
TTLL5	5.22928575992402e-26	0.270777094025953	0.333	0.255	1.51230944177003e-21	Parietal Epithelial Cell
ITGB8	6.85026442851163e-25	0.771395377046484	0.348	0.289	1.98109647272556e-20	Parietal Epithelial Cell
GNGT1	3.15753283821146e-24	0.263880106623132	0.278	0.206	9.13158496810755e-20	Parietal Epithelial Cell
LMCD1-AS1	6.3527741417039e-24	0.272225660062694	0.31	0.236	1.83722228178077e-19	Parietal Epithelial Cell
HNRNPH3	1.09618075604157e-23	0.25536438438131	0.261	0.192	3.17015474647223e-19	Parietal Epithelial Cell
NAA50	1.48499264432533e-23	0.251219563984776	0.251	0.185	4.29459872738885e-19	Parietal Epithelial Cell
UTY	1.72982453354187e-23	0.326335811936854	0.327	0.257	5.00265255100309e-19	Parietal Epithelial Cell
TGFBR2	2.03195784482784e-22	0.640209222911573	0.327	0.262	5.87642208724212e-18	Parietal Epithelial Cell
SEMA4D	2.05068463964127e-22	0.278724392642285	0.311	0.242	5.93057997784254e-18	Parietal Epithelial Cell
NAP1L1	4.88188037326246e-22	0.266516173922061	0.285	0.218	1.4118398039475e-17	Parietal Epithelial Cell
LINC02241	2.89309903573737e-21	0.267344117255246	0.253	0.189	8.36684241135248e-17	Parietal Epithelial Cell
NCEH1	1.96134612981206e-20	0.278631908164522	0.251	0.188	5.67221300741647e-16	Parietal Epithelial Cell
VMP1	3.93968357905642e-20	0.352935121973491	0.498	0.407	1.13935649106312e-15	Parietal Epithelial Cell
ARHGAP10	2.16375430626137e-18	0.95889571361368	0.287	0.241	6.25757745370789e-14	Parietal Epithelial Cell
PLCL2	2.80012762298721e-17	0.324972050421329	0.375	0.311	8.097969085679e-13	Parietal Epithelial Cell
SORT1	6.9564756662121e-17	0.30671535094532	0.254	0.199	2.01181276266854e-12	Parietal Epithelial Cell
ZFP36L1	8.08239541941664e-16	0.524323447434135	0.286	0.234	2.33742875529529e-11	Parietal Epithelial Cell
DCDC2	9.24512046809543e-16	0.712185353671241	0.505	0.449	2.6736888393732e-11	Parietal Epithelial Cell
SOD2	1.29998987996625e-14	0.387746712432191	0.281	0.224	3.7595707328624e-10	Parietal Epithelial Cell
ERRFI1	6.00915556888422e-07	0.285720007243611	0.283	0.318	0.0173784779052132	Parietal Epithelial Cell
NEDD9	0.000615856943304338	0.717010789759162	0.284	0.277	1	Parietal Epithelial Cell
LDB2	0	6.35383561086398	0.869	0.081	0	Endothelial Cell
MEIS2	0	5.45638773796228	0.874	0.101	0	Endothelial Cell
PTPRB	0	6.87641828247084	0.806	0.034	0	Endothelial Cell

gene	p_val	avg_log2FC	pct.1	pct.2	p_val_adj	cluster
EMCN	0	6.49406734785164	0.824	0.076	0	Endothelial Cell
FLT1	0	6.73628397822188	0.769	0.043	0	Endothelial Cell
SLCO2A1	0	5.98865089915721	0.743	0.054	0	Endothelial Cell
ST6GALNAC3	0	2.89195178258725	0.795	0.124	0	Endothelial Cell
TEK	0	6.65837142204396	0.677	0.02	0	Endothelial Cell
EGFL7	0	6.43747203262863	0.671	0.019	0	Endothelial Cell
RBMS3	0	3.40530268120077	0.828	0.199	0	Endothelial Cell
ZEB1	0	4.00949353132791	0.795	0.179	0	Endothelial Cell
EPAS1	0	4.83523036846638	0.768	0.155	0	Endothelial Cell
PECAM1	0	5.97682385532182	0.626	0.023	0	Endothelial Cell
SHANK3	0	4.43237075522798	0.66	0.064	0	Endothelial Cell
RAPGEF4	0	5.9357708321876	0.628	0.034	0	Endothelial Cell
PLPP1	0	4.79894789699103	0.865	0.272	0	Endothelial Cell
ELMO1	0	3.87547553711075	0.673	0.09	0	Endothelial Cell
SH3RF3	0	4.740834316	0.641	0.067	0	Endothelial Cell
SNED1	0	4.07674366539092	0.674	0.114	0	Endothelial Cell
ADGRF5	0	2.23142953957245	0.7	0.141	0	Endothelial Cell
GNA14	0	5.01750701676741	0.587	0.042	0	Endothelial Cell
ERG	0	5.56932912734617	0.554	0.018	0	Endothelial Cell
TCF4	0	4.43014303536646	0.633	0.099	0	Endothelial Cell
TMTC1	0	3.71685571667467	0.622	0.107	0	Endothelial Cell
ARHGAP31	0	4.26949561650633	0.583	0.072	0	Endothelial Cell
NOSTRIN	0	4.41118063672248	0.572	0.071	0	Endothelial Cell
PRKCH	0	3.31057235303927	0.617	0.118	0	Endothelial Cell
ENG	0	5.23161886938653	0.522	0.028	0	Endothelial Cell
FBXL7	0	2.93947299753084	0.598	0.111	0	Endothelial Cell
DOCK4	0	3.12892492756481	0.804	0.319	0	Endothelial Cell
PREX2	0	6.29190384773819	0.492	0.014	0	Endothelial Cell
TIMP3	0	3.87090080635518	0.663	0.192	0	Endothelial Cell
EBF1	0	3.30661366891209	0.52	0.05	0	Endothelial Cell
SEC14L1	0	3.27254735824354	0.709	0.243	0	Endothelial Cell
EML1	0	4.49343682874742	0.522	0.066	0	Endothelial Cell
ASAP1	0	3.07807455108906	0.752	0.297	0	Endothelial Cell
XAF1	0	4.71551312715773	0.48	0.032	0	Endothelial Cell
LIMS2	0	5.04450973929352	0.474	0.03	0	Endothelial Cell
MEF2C	0	2.95176418799671	0.594	0.155	0	Endothelial Cell
ADGRL4	0	6.25765174659458	0.447	0.014	0	Endothelial Cell
CEACAM1	0	4.28810116212435	0.487	0.057	0	Endothelial Cell
KALRN	0	3.65326316295538	0.551	0.123	0	Endothelial Cell
GRB10	0	2.75165247508575	0.712	0.294	0	Endothelial Cell
HECW2	0	6.20445463559502	0.439	0.022	0	Endothelial Cell
LDLRAD4	0	2.33240893308507	0.645	0.232	0	Endothelial Cell

gene	p_val	avg_log2FC	pct.1	pct.2	p_val_adj	cluster
HSPG2	0	3.76784196650368	0.523	0.112	0	Endothelial Cell
DLC1	0	3.22174688810727	0.506	0.097	0	Endothelial Cell
AC119674.1	0	3.71554972310813	0.528	0.12	0	Endothelial Cell
ST6GAL1	0	2.51112968627048	0.661	0.261	0	Endothelial Cell
ETS1	0	3.00673137095309	0.482	0.083	0	Endothelial Cell
HEG1	0	4.48053923033895	0.435	0.037	0	Endothelial Cell
FLI1	0	3.87438013430227	0.427	0.03	0	Endothelial Cell
PREX1	0	4.05050789654999	0.429	0.035	0	Endothelial Cell
PLAT	0	6.63027085522785	0.41	0.018	0	Endothelial Cell
FGD5	0	3.6562006745876	0.45	0.059	0	Endothelial Cell
RASAL2	0	2.79027684307901	0.774	0.384	0	Endothelial Cell
ZBTB46	0	3.06115807278754	0.522	0.133	0	Endothelial Cell
CACNA1C	0	3.25779916392283	0.464	0.077	0	Endothelial Cell
RAPGEF5	0	3.76713939697191	0.452	0.066	0	Endothelial Cell
CDH13	0	5.4261475358541	0.413	0.028	0	Endothelial Cell
BTNL9	0	5.57538306022947	0.401	0.023	0	Endothelial Cell
DNM3	0	2.54370990774943	0.633	0.256	0	Endothelial Cell
NOTCH4	0	6.72512513388061	0.383	0.006	0	Endothelial Cell
NAV1	0	4.28564722601072	0.423	0.048	0	Endothelial Cell
HIVEP3	0	2.94963243907807	0.531	0.158	0	Endothelial Cell
DYSF	0	4.74626979121449	0.406	0.033	0	Endothelial Cell
FYN	0	2.43320849401178	0.448	0.075	0	Endothelial Cell
ARHGAP26	0	2.7053648795477	0.671	0.302	0	Endothelial Cell
RARB	0	3.14614142417649	0.525	0.156	0	Endothelial Cell
PITPNC1	0	2.67449878288388	0.677	0.309	0	Endothelial Cell
KLF7	0	2.69755702680314	0.522	0.154	0	Endothelial Cell
PIK3R3	0	3.59208342135864	0.495	0.13	0	Endothelial Cell
RAMP3	0	5.71033495833798	0.382	0.018	0	Endothelial Cell
ARAP3	0	5.92627497551211	0.372	0.011	0	Endothelial Cell
NRG3	0	3.26514550302641	0.536	0.178	0	Endothelial Cell
RUNX1T1	0	4.6929042517701	0.383	0.027	0	Endothelial Cell
PDGFD	0	4.45006213359254	0.406	0.051	0	Endothelial Cell
DOCK9	0	2.96016711984966	0.583	0.228	0	Endothelial Cell
CALCRL	0	4.57816526127794	0.387	0.037	0	Endothelial Cell
ITGA9	0	4.36741486674174	0.403	0.053	0	Endothelial Cell
HIF3A	0	5.00651904366912	0.374	0.024	0	Endothelial Cell
GFOD1	0	5.40499942635288	0.369	0.02	0	Endothelial Cell
IL4R	0	3.97051170936061	0.393	0.046	0	Endothelial Cell
IRAK3	0	4.28568157090457	0.371	0.027	0	Endothelial Cell
SPTBN1	0	2.30413901150971	0.808	0.466	0	Endothelial Cell
SEMA6A	0	3.09649010114217	0.449	0.11	0	Endothelial Cell
PLCB1	0	0.911991667547042	0.673	0.335	0	Endothelial Cell

gene	p_val	avg_log2FC	pct.1	pct.2	p_val_adj	cluster
UTRN	0	2.05892048478384	0.816	0.48	0	Endothelial Cell
PODXL	0	2.20274375606425	0.449	0.114	0	Endothelial Cell
SH2D3C	0	5.64679598233404	0.337	0.009	0	Endothelial Cell
CHST11	0	2.62618869561889	0.393	0.066	0	Endothelial Cell
SASH1	0	1.98128318793036	0.694	0.367	0	Endothelial Cell
PLPP3	0	3.1682734983023	0.608	0.282	0	Endothelial Cell
TGFBR2	0	2.6869835190623	0.572	0.247	0	Endothelial Cell
PLEKHG1	0	2.74627511204792	0.517	0.193	0	Endothelial Cell
PIP4K2A	0	2.52041691202312	0.575	0.254	0	Endothelial Cell
ABR	0	2.20048313471445	0.449	0.132	0	Endothelial Cell
ELK3	0	4.23682092209489	0.348	0.032	0	Endothelial Cell
CNTNAP3B	0	1.55062628940494	0.648	0.333	0	Endothelial Cell
PEAK1	0	2.28635425833737	0.571	0.259	0	Endothelial Cell
AKT3	0	1.93695572794687	0.731	0.42	0	Endothelial Cell
TACC1	0	1.96006772965353	0.774	0.463	0	Endothelial Cell
CDK17	0	2.74730985169084	0.493	0.185	0	Endothelial Cell
CMTM8	0	2.84327718276446	0.463	0.156	0	Endothelial Cell
TCF7L1	0	2.3671083195058	0.426	0.119	0	Endothelial Cell
ADCY4	0	6.12704620961362	0.315	0.008	0	Endothelial Cell
PCED1B	0	2.61698280960752	0.423	0.117	0	Endothelial Cell
NCALD	0	3.18798373669812	0.379	0.074	0	Endothelial Cell
RBMS2	0	2.65946200304998	0.476	0.171	0	Endothelial Cell
SLC9A3R2	0	5.19296019399723	0.319	0.016	0	Endothelial Cell
EXOC3L2	0	6.2729051416068	0.309	0.008	0	Endothelial Cell
IGFBP5	0	2.9938548098501	0.395	0.097	0	Endothelial Cell
SERPINB6	0	2.76736487563496	0.426	0.128	0	Endothelial Cell
C22orf34	0	4.94729159651361	0.307	0.011	0	Endothelial Cell
EVA1C	0	2.66049821770635	0.41	0.117	0	Endothelial Cell
CHN1	0	4.04495550208881	0.335	0.043	0	Endothelial Cell
NLRP1	0	4.33133395976286	0.308	0.017	0	Endothelial Cell
KDR	0	6.09616556457143	0.295	0.008	0	Endothelial Cell
FAR2	0	4.39261660947643	0.312	0.027	0	Endothelial Cell
EFNB2	0	4.72672436726681	0.314	0.029	0	Endothelial Cell
CD34	0	6.22662957937615	0.291	0.007	0	Endothelial Cell
OSMR	0	2.70206759951061	0.414	0.13	0	Endothelial Cell
SLCO3A1	0	1.9305377642904	0.45	0.167	0	Endothelial Cell
LHFPL6	0	2.6434124451351	0.348	0.065	0	Endothelial Cell
SRGAP2	0	2.01878152968906	0.393	0.111	0	Endothelial Cell
CHRM3	0	3.52461426095338	0.464	0.183	0	Endothelial Cell
SH3BP5	0	2.63744430500686	0.387	0.107	0	Endothelial Cell
BMPR2	0	2.03150514412549	0.684	0.406	0	Endothelial Cell
NXN	0	3.01991861661372	0.356	0.078	0	Endothelial Cell

gene	p_val	avg_log2FC	pct.1	pct.2	p_val_adj	cluster
ROBO4	0	6.70467300234961	0.278	0.004	0	Endothelial Cell
AFAP1L1	0	5.19999375490367	0.287	0.013	0	Endothelial Cell
IL6ST	0	2.21286627210029	0.542	0.269	0	Endothelial Cell
PLXND1	0	4.95871996217376	0.283	0.012	0	Endothelial Cell
NPDC1	0	5.203399923	0.284	0.013	0	Endothelial Cell
SYNE1	0	2.93675463171717	0.844	0.574	0	Endothelial Cell
ZBTB16	0	1.35499095788061	0.796	0.526	0	Endothelial Cell
SMAD6	0	3.61595749976599	0.34	0.071	0	Endothelial Cell
PBX1	0	2.3644765184319	0.798	0.531	0	Endothelial Cell
ADGRB3	0	4.27628391126644	0.292	0.025	0	Endothelial Cell
PTPRM	0	2.35047695797019	0.938	0.671	0	Endothelial Cell
SP100	0	1.87509207783625	0.53	0.265	0	Endothelial Cell
PARP14	0	1.96271939495111	0.49	0.225	0	Endothelial Cell
KLF2	0	4.86214273837833	0.279	0.015	0	Endothelial Cell
APP	0	2.14112690975008	0.763	0.499	0	Endothelial Cell
MAP4K4	0	1.96313436773299	0.488	0.227	0	Endothelial Cell
TOX2	0	5.09099387111434	0.269	0.01	0	Endothelial Cell
HLA-E	0	2.89575238419526	0.329	0.071	0	Endothelial Cell
GSE1	0	1.8383988122222	0.602	0.344	0	Endothelial Cell
EPB41L2	0	2.33232483485974	0.35	0.095	0	Endothelial Cell
IFI16	0	3.12702555987802	0.292	0.038	0	Endothelial Cell
FAT4	0	2.72867772836268	0.351	0.099	0	Endothelial Cell
CYYR1	0	3.72011981632291	0.3	0.048	0	Endothelial Cell
KLHL5	0	2.08547439087722	0.465	0.215	0	Endothelial Cell
ITGA8	0	4.44879765938511	0.274	0.024	0	Endothelial Cell
A2M	0	3.25574978040817	0.272	0.024	0	Endothelial Cell
LIMCH1	0	1.44406747645705	0.762	0.516	0	Endothelial Cell
SWAP70	0	2.50907444613518	0.37	0.124	0	Endothelial Cell
CD109	0	3.82558491006971	0.275	0.032	0	Endothelial Cell
FLT4	0	5.52080460333292	0.256	0.013	0	Endothelial Cell
GRASP	0	5.10343771141409	0.256	0.013	0	Endothelial Cell
TBC1D8	0	2.17562067148135	0.536	0.298	0	Endothelial Cell
UACA	0	1.40411089416052	0.617	0.381	0	Endothelial Cell
PDIA5	0	2.91869984583092	0.321	0.086	0	Endothelial Cell
ITGA1	0	1.81984740371347	0.443	0.208	0	Endothelial Cell
PALD1	0	4.44435375513347	0.251	0.016	0	Endothelial Cell
PTPN12	0	1.97568613206104	0.455	0.221	0	Endothelial Cell
CAMK1D	0	2.06883146894909	0.306	0.073	0	Endothelial Cell
DOCK6	0	2.65832700949726	0.342	0.11	0	Endothelial Cell
SGK1	0	2.59033043135892	0.33	0.098	0	Endothelial Cell
FCHSD2	0	1.65327404572576	0.587	0.355	0	Endothelial Cell
AKAP12	0	2.62224662035494	0.34	0.112	0	Endothelial Cell

gene	p_val	avg_log2FC	pct.1	pct.2	p_val_adj	cluster
NHSL1	0	2.5995232889305	0.412	0.184	0	Endothelial Cell
TBCD	0	1.97552301225897	0.423	0.195	0	Endothelial Cell
PDLIM1	0	4.09964219949513	0.255	0.028	0	Endothelial Cell
PPFIBP1	0	1.51116038684444	0.654	0.428	0	Endothelial Cell
IFI44	0	4.02952269377403	0.252	0.028	0	Endothelial Cell
SORBS1	0	2.15924789076453	0.425	0.201	0	Endothelial Cell
ETS2	0	2.88254280678328	0.299	0.076	0	Endothelial Cell
PIK3C2A	0	1.83590863735269	0.613	0.391	0	Endothelial Cell
PIK3C2B	0	3.42350593054854	0.257	0.036	0	Endothelial Cell
JAM3	0	3.32956428928949	0.266	0.048	0	Endothelial Cell
RNF144A	0	2.66994009278514	0.3	0.082	0	Endothelial Cell
TTC28	0	1.26662036638823	0.734	0.517	0	Endothelial Cell
RASGRP3	0	2.89741354316006	0.295	0.079	0	Endothelial Cell
ARGLU1	0	1.50014320895131	0.621	0.412	0	Endothelial Cell
ADARB1	0	2.17080957233802	0.321	0.112	0	Endothelial Cell
RBMS1	0	1.78447294668762	0.655	0.448	0	Endothelial Cell
PELI1	0	2.60935850242735	0.321	0.115	0	Endothelial Cell
GRK5	0	2.40363131210274	0.266	0.06	0	Endothelial Cell
DISC1	0	2.57781282947867	0.262	0.057	0	Endothelial Cell
EPB41L4A	0	1.47103902097896	0.649	0.445	0	Endothelial Cell
CD9	0	2.44952512058824	0.32	0.116	0	Endothelial Cell
AC007319.1	0	2.82130936528504	0.286	0.086	0	Endothelial Cell
TNS2	0	2.23403422148519	0.293	0.1	0	Endothelial Cell
ANKS1A	0	2.20498155653874	0.585	0.393	0	Endothelial Cell
DENND3	0	2.61402772157298	0.259	0.072	0	Endothelial Cell
EHD4	0	2.89029952023804	0.27	0.086	0	Endothelial Cell
GNAQ	0	1.44934019317505	0.707	0.527	0	Endothelial Cell
AMOTL1	0	2.44755228457625	0.265	0.086	0	Endothelial Cell
ZNF521	0	2.72868414054345	0.254	0.077	0	Endothelial Cell
PTPRG	0	1.78492413101997	0.899	0.733	0	Endothelial Cell
MEF2A	2.23137573981596e-307	1.31407917212374	0.686	0.525	6.45313863954776e-303	Endothelial Cell
NCOA3	3.23437523657062e-303	1.72571512828743	0.524	0.313	9.35381318416225e-299	Endothelial Cell
PLXNA2	7.26472942993435e-299	2.0235014333887	0.33	0.129	2.10095975113701e-294	Endothelial Cell
EPHA4	1.3960789318808e-297	2.38283852566942	0.26	0.086	4.03746027099928e-293	Endothelial Cell
NFIA	1.6432913521781e-285	1.33109883896334	0.641	0.424	4.75239859049906e-281	Endothelial Cell
MACF1	6.68863097106485e-278	0.831434407728197	0.83	0.731	1.93435207683195e-273	Endothelial Cell
DHRS3	8.93389581819868e-274	1.9808310761491	0.435	0.223	2.58368267062306e-269	Endothelial Cell
LIFR	8.871060580489e-266	1.82211943030854	0.572	0.391	2.56551071987742e-261	Endothelial Cell
ABCC1	6.09154980729178e-265	1.97888872257335	0.341	0.149	1.76167620426878e-260	Endothelial Cell
HLA-B	1.07237616787533e-264	2.06470860363248	0.264	0.094	3.10131187749546e-260	Endothelial Cell
HDAC7	1.64725848359416e-262	2.15776050923763	0.253	0.089	4.7638715345543e-258	Endothelial Cell
LIMA1	1.6194933713565e-260	1.64920190889297	0.435	0.226	4.68357482996299e-256	Endothelial Cell

gene	p_val	avg_log2FC	pct.1	pct.2	p_val_adj	cluster
CEP112	4.53449447463407e-257	1.53276509912477	0.523	0.319	1.31137580206417e-252	Endothelial Cell
WWC3	7.12074861882707e-257	2.2787686370513	0.251	0.09	2.05932050056479e-252	Endothelial Cell
STOX2	2.56810379002417e-252	1.46090466915174	0.525	0.316	7.4269561607499e-248	Endothelial Cell
ADGRL2	3.0972103561742e-252	1.88967959072828	0.551	0.376	8.95713235005578e-248	Endothelial Cell
RPGR	1.14516470247898e-250	2.3956357776439	0.274	0.107	3.31181631956922e-246	Endothelial Cell
TANC1	1.24678467803272e-250	1.46418082881398	0.548	0.358	3.60570128887062e-246	Endothelial Cell
ATP8B1	8.6056137008426e-250	1.9606161955861	0.362	0.17	2.48874348228368e-245	Endothelial Cell
TSPAN14	1.39694773110916e-247	1.80817558440153	0.34	0.152	4.0399728383677e-243	Endothelial Cell
CTNNB1	5.35775386363966e-245	1.88650965107051	0.418	0.226	1.54946241736459e-240	Endothelial Cell
PALM2-AKAP2	6.68835244284193e-245	1.70432515686082	0.502	0.299	1.93427152646988e-240	Endothelial Cell
PLSCR4	4.43078202523453e-237	2.09327677434563	0.267	0.104	1.28138216169783e-232	Endothelial Cell
PTK2	6.71074805279065e-237	0.870086092110858	0.761	0.651	1.94074833686706e-232	Endothelial Cell
MCC	6.02651738053705e-236	1.79473161333279	0.386	0.195	1.74286882645132e-231	Endothelial Cell
ZNF438	2.90211130969261e-234	1.90532356075442	0.405	0.218	8.39290590763102e-230	Endothelial Cell
DPYD	6.27590515398334e-234	1.32100551747642	0.557	0.373	1.81499177053198e-229	Endothelial Cell
DLEU2	6.39073620492433e-233	1.44075296559809	0.452	0.248	1.84820091046412e-228	Endothelial Cell
KIAA1671	1.00225662229398e-227	1.42033182393851	0.524	0.341	2.89852615167418e-223	Endothelial Cell
CTTNBP2NL	2.71008922092603e-227	1.97672905562508	0.319	0.144	7.83757802691809e-223	Endothelial Cell
RASGRF2	1.43027399908101e-225	1.91211026336494	0.379	0.194	4.13635240534229e-221	Endothelial Cell
CARD8	1.39353050778599e-224	1.96962981889362	0.286	0.12	4.03009022851709e-220	Endothelial Cell
ARHGAP29	2.27358293352967e-218	1.18339820480576	0.583	0.399	6.57520184376781e-214	Endothelial Cell
ANO6	1.28799460737075e-216	1.78597261795242	0.462	0.282	3.72488040451621e-212	Endothelial Cell
NCOA7	1.35314848411727e-216	1.31617920344201	0.542	0.345	3.91330541606716e-212	Endothelial Cell
CLIC4	2.7940385739029e-216	1.27656113187179	0.398	0.198	8.0803595557272e-212	Endothelial Cell
GAS6	4.97474401456865e-215	1.94240646919771	0.302	0.134	1.43869596901325e-210	Endothelial Cell
FGFR1	9.74349205866441e-215	2.20781971668977	0.284	0.122	2.81781790336575e-210	Endothelial Cell
CNN3	7.89374232030993e-210	1.97963848909076	0.399	0.226	2.28287027903363e-205	Endothelial Cell
JMJD1C	2.35360069008763e-208	0.92793263887756	0.789	0.707	6.80661319573342e-204	Endothelial Cell
RPS6KA2	8.27898897410634e-208	1.7816817132977	0.405	0.227	2.39428361131155e-203	Endothelial Cell
PIAS1	5.16774103739452e-207	1.52148911994405	0.562	0.416	1.49451070801449e-202	Endothelial Cell
LIMK2	3.87655883632855e-206	2.03119115566572	0.256	0.104	1.12110081546622e-201	Endothelial Cell
ATP2B4	3.572269422968e-205	1.89215341910504	0.308	0.142	1.03310031712235e-200	Endothelial Cell
CPNE8	9.41546945364644e-204	1.42818117028343	0.472	0.293	2.72295376599455e-199	Endothelial Cell
MSN	1.36120615290373e-202	1.64463988866081	0.344	0.17	3.9366081941976e-198	Endothelial Cell
ANKRD44	1.48476368227969e-202	1.48640629460919	0.355	0.179	4.29393656915287e-198	Endothelial Cell
RIN2	1.3951747755305e-200	1.31390344665537	0.586	0.44	4.03484545083422e-196	Endothelial Cell
HMBOX1	5.25704961905172e-198	1.68848505254582	0.459	0.293	1.52033874982976e-193	Endothelial Cell
NEDD4	3.79602409814205e-195	1.63941961097571	0.298	0.135	1.09781016918268e-190	Endothelial Cell
AFF1	3.32797454345653e-194	0.879224051211828	0.736	0.622	9.62450237967628e-190	Endothelial Cell
MYO1C	8.96293909490171e-193	2.02405683291965	0.255	0.108	2.59208198624557e-188	Endothelial Cell
RBFOX2	1.92183596345905e-189	1.23955730654934	0.572	0.429	5.55794960632356e-185	Endothelial Cell
ZFHX3	2.61776319246856e-186	1.31594209281838	0.479	0.312	7.57057115261906e-182	Endothelial Cell

gene	p_val	avg_log2FC	pct.1	pct.2	p_val_adj	cluster
CDC42BPA	3.93526910534824e-182	1.00914006821095	0.666	0.551	1.13807982526671e-177	Endothelial Cell
FOXN3	3.38103479378766e-181	1.12181519211205	0.564	0.408	9.77795262363391e-177	Endothelial Cell
PPP1R16B	2.77722437472972e-179	1.17396094027007	0.352	0.172	8.03173289171835e-175	Endothelial Cell
DUSP1	8.17624219772286e-179	1.62434378641331	0.358	0.189	2.36456924358145e-174	Endothelial Cell
PRKCE	8.41265083656125e-178	1.14241217601816	0.647	0.519	2.43293862193351e-173	Endothelial Cell
RALGAPA2	2.41737768376152e-176	1.30743278207088	0.576	0.443	6.99105626143832e-172	Endothelial Cell
VOPP1	1.98202242000093e-175	1.45859847154383	0.387	0.218	5.7320088386427e-171	Endothelial Cell
RFX3	2.10760880968826e-175	1.43171632764954	0.485	0.328	6.09520467761844e-171	Endothelial Cell
ARL15	3.94449728791432e-175	0.862337053187067	0.792	0.661	1.14074861566482e-170	Endothelial Cell
QKI	5.38444816733169e-174	1.05998796080877	0.614	0.489	1.55718240999233e-169	Endothelial Cell
RAPGEF1	2.36713776297893e-171	1.46490712689711	0.478	0.325	6.84576241053507e-167	Endothelial Cell
COL4A2	2.20517968195128e-169	1.16841249914586	0.527	0.361	6.3773796402031e-165	Endothelial Cell
IFNAR2	3.18288352738865e-165	1.59946626611752	0.256	0.114	9.20489916120799e-161	Endothelial Cell
CALD1	3.32036511571229e-165	0.780465527721797	0.452	0.262	9.60249591463995e-161	Endothelial Cell
PIEZO1	1.66405702601574e-163	1.73348720675217	0.252	0.112	4.81245291923753e-159	Endothelial Cell
PHLPP1	2.19836438760288e-163	1.46819752698168	0.429	0.273	6.35766980894752e-159	Endothelial Cell
KIAA0355	1.56584861580719e-162	1.47674743153631	0.422	0.269	4.5284341969144e-158	Endothelial Cell
TGFBR3	1.7968691346046e-159	1.24157416313851	0.363	0.196	5.1965455372765e-155	Endothelial Cell
CNST	1.09752535778709e-157	1.64081679571988	0.321	0.174	3.17404333472027e-153	Endothelial Cell
RAP1B	2.29075658532463e-157	1.43462729913134	0.456	0.307	6.62486804475883e-153	Endothelial Cell
NRP1	4.99611040467427e-156	1.0437227458772	0.471	0.292	1.4448751290318e-151	Endothelial Cell
ETV6	2.98639965054564e-155	1.21239835849833	0.508	0.362	8.63666778937799e-151	Endothelial Cell
DNAJC1	4.61859732391811e-155	1.26229874378805	0.526	0.396	1.33569834607712e-150	Endothelial Cell
YES1	8.31494712457879e-155	1.78597639663524	0.351	0.204	2.40468270842818e-150	Endothelial Cell
HIPK2	1.34143354998353e-154	1.02329113943372	0.649	0.547	3.87942582655236e-150	Endothelial Cell
KLF9	3.05497043699434e-154	1.70995440740155	0.331	0.184	8.83497450378763e-150	Endothelial Cell
CADPS2	5.96661898358564e-153	0.475710682352974	0.712	0.552	1.72554621005297e-148	Endothelial Cell
FZD4	1.56494867934139e-152	1.65461111479513	0.256	0.12	4.5258315806553e-148	Endothelial Cell
WWTR1	2.36774677090955e-152	1.28207729333956	0.546	0.406	6.84752366147042e-148	Endothelial Cell
DGKH	1.10613414610712e-151	1.19238141259026	0.446	0.281	3.19893995054179e-147	Endothelial Cell
PICALM	5.85414025114121e-151	0.869014909376506	0.678	0.572	1.69301736063004e-146	Endothelial Cell
B2M	2.94806397805843e-147	1.27125040308869	0.343	0.189	8.52580102454499e-143	Endothelial Cell
FNBP1	8.30177175900104e-147	1.22438128007635	0.434	0.28	2.4008723927031e-142	Endothelial Cell
MTSS1	7.58052469021299e-144	1.12169862582687	0.57	0.446	2.1922877404096e-139	Endothelial Cell
SNRK	4.73363352266442e-142	1.61235485197787	0.363	0.221	1.36896681475455e-137	Endothelial Cell
ARHGEF3	6.30809686687799e-142	1.15061118025608	0.528	0.387	1.82430161390112e-137	Endothelial Cell
FRMD4B	2.04378956635493e-140	0.964268044354308	0.508	0.338	5.91063942589845e-136	Endothelial Cell
KAT6A	2.69062341967543e-140	1.00771662213579	0.561	0.44	7.78128292970134e-136	Endothelial Cell
FUT8	9.47118283651196e-139	1.5757723602981	0.36	0.214	2.73906607631926e-134	Endothelial Cell
ECE1	9.40406361403182e-138	1.60674768739847	0.264	0.131	2.719655197178e-133	Endothelial Cell
APBB2	8.06647190278644e-137	0.721551927025341	0.673	0.548	2.33282367428584e-132	Endothelial Cell
CCNY	5.90059161152596e-133	1.09109614641866	0.575	0.478	1.70645109405331e-128	Endothelial Cell

gene	p_val	avg_log2FC	pct.1	pct.2	p_val_adj	cluster
STARD13	8.16804349733398e-132	1.01286460972155	0.544	0.404	2.36219817942899e-127	Endothelial Cell
PPP1R13B	8.4295221624582e-132	1.21589111881915	0.44	0.297	2.43781780938291e-127	Endothelial Cell
RB1	3.70064183706904e-130	1.13478910086073	0.491	0.36	1.07022561928037e-125	Endothelial Cell
PXN	4.1351388041065e-130	1.58890369723923	0.315	0.179	1.1958821421476e-125	Endothelial Cell
ATP11C	1.29705627340504e-129	1.44710599023934	0.307	0.17	3.75108674268737e-125	Endothelial Cell
PLXDC2	1.15794003529363e-127	0.881523350001877	0.675	0.586	3.34876258206918e-123	Endothelial Cell
ARHGEF10	2.7398728504665e-127	1.53351608761624	0.272	0.143	7.92371228354913e-123	Endothelial Cell
AC002070.1	1.55838660675978e-126	1.67194725538567	0.276	0.147	4.50685406674929e-122	Endothelial Cell
BMS1P14	1.91116340006915e-125	1.60187355572292	0.276	0.146	5.52708455299997e-121	Endothelial Cell
KIF26B	7.45050687431134e-125	1.49042582144776	0.276	0.144	2.15468658805084e-120	Endothelial Cell
MED13L	9.54697648593085e-125	0.773602697700089	0.72	0.648	2.7609855997312e-120	Endothelial Cell
NFKBIA	2.06202772606852e-121	1.47526962397878	0.263	0.136	5.96338418379015e-117	Endothelial Cell
C5orf56	6.65750834066124e-121	1.58651772442594	0.292	0.164	1.92535141211923e-116	Endothelial Cell
NKTR	1.20750598491997e-120	0.656381792518575	0.713	0.649	3.49210730838854e-116	Endothelial Cell
FKBP5	1.28871084620962e-120	0.551070246238447	0.687	0.518	3.72695176723822e-116	Endothelial Cell
CYTH3	9.17698988151433e-120	1.69115789469543	0.27	0.147	2.65398547373394e-115	Endothelial Cell
RNF115	2.06625955609536e-118	1.23669353695034	0.458	0.335	5.97562263622779e-114	Endothelial Cell
KIF13A	3.2003038113086e-118	1.30524163655923	0.435	0.31	9.25527862230447e-114	Endothelial Cell
NR3C1	7.59910078553515e-117	1.18942185515735	0.369	0.231	2.19765994717677e-112	Endothelial Cell
ZDHHC14	1.95947707825248e-115	1.04760748240336	0.465	0.321	5.66680771030617e-111	Endothelial Cell
KLC1	2.72770964784825e-114	1.10568766304304	0.46	0.335	7.88853630157715e-110	Endothelial Cell
MDFIC	2.60504955131936e-113	1.41533239194436	0.269	0.146	7.5338033024156e-109	Endothelial Cell
RCAN2	7.06609420389546e-112	0.757987567978671	0.363	0.216	2.04351444376657e-107	Endothelial Cell
FRYL	1.67661127759616e-111	1.00091091328592	0.539	0.442	4.84875981480809e-107	Endothelial Cell
SKIL	9.66811715768602e-111	1.44295099723299	0.288	0.165	2.7960194820028e-106	Endothelial Cell
RGL1	1.06830372225018e-110	1.43805606689074	0.316	0.186	3.08953436474752e-106	Endothelial Cell
JAZF1	1.17179794443564e-110	1.27354914237224	0.271	0.147	3.38883965530786e-106	Endothelial Cell
SOX13	2.01340498499937e-109	1.42031177556342	0.251	0.133	5.82276721661817e-105	Endothelial Cell
TJP1	5.1691538686213e-108	0.881175966909619	0.502	0.376	1.49491929880528e-103	Endothelial Cell
SBF2	2.47466197291674e-107	0.608862023395275	0.742	0.678	7.1567224256752e-103	Endothelial Cell
HERC1	5.14788624103116e-107	0.828596445253608	0.611	0.524	1.48876870090621e-102	Endothelial Cell
GSN	5.6308754896657e-107	1.61161904587025	0.285	0.167	1.62844919161132e-102	Endothelial Cell
CFLAR	3.80592875333988e-106	0.805184619371929	0.588	0.487	1.10067459546589e-101	Endothelial Cell
FMNL2	1.27869218051003e-105	1.11455701717848	0.498	0.384	3.697977786035e-101	Endothelial Cell
RESF1	2.40742147486106e-105	1.15595976794399	0.345	0.215	6.96226290529819e-101	Endothelial Cell
RANBP17	1.01041890610959e-103	1.30296079222049	0.311	0.186	2.92213147646894e-99	Endothelial Cell
MAML3	4.20890659556921e-103	1.09765212054454	0.493	0.372	1.21721578743862e-98	Endothelial Cell
IKBKB	3.94511833862028e-102	1.48707034858731	0.289	0.172	1.14092822352899e-97	Endothelial Cell
RIMKLB	8.19872241347446e-101	1.46847541283478	0.312	0.195	2.37107052197681e-96	Endothelial Cell
SH3D19	5.93494720506296e-99	1.0975044078468	0.485	0.376	1.71638673170421e-94	Endothelial Cell
ANKRD36C	8.01266307737236e-95	0.91867352910235	0.409	0.278	2.31726216197609e-90	Endothelial Cell
STAG1	7.92261681617255e-94	0.659476849275476	0.668	0.6	2.2912207832371e-89	Endothelial Cell

gene	p_val	avg_log2FC	pct.1	pct.2	p_val_adj	cluster
LRCH1	6.8839343743307e-93	0.701632505803309	0.591	0.488	1.99083382105644e-88	Endothelial Cell
SHROOM4	2.55501846749204e-92	0.810904510492366	0.436	0.307	7.38911340798699e-88	Endothelial Cell
ITSN2	5.99332559541071e-92	1.12820730072755	0.492	0.403	1.73326976219278e-87	Endothelial Cell
WASF2	1.42754664336664e-91	1.11720003698364	0.396	0.285	4.12846489261631e-87	Endothelial Cell
CD58	1.67735342210744e-91	1.26316890939145	0.262	0.151	4.85090609673473e-87	Endothelial Cell
TFPI	1.79370428035622e-90	1.33954072737699	0.285	0.17	5.18739277879018e-86	Endothelial Cell
ATXN3	2.62295401947293e-90	1.34293636711849	0.282	0.171	7.58558302431571e-86	Endothelial Cell
TCF12	1.95283645163765e-89	0.587086916948419	0.707	0.651	5.64760301813609e-85	Endothelial Cell
COL4A1	5.92624745734647e-85	0.94394576727574	0.381	0.262	1.7138707646646e-80	Endothelial Cell
GNB1	1.15581932984276e-84	0.905536288862833	0.483	0.381	3.34262950190527e-80	Endothelial Cell
SMURF2	1.33255291523676e-84	1.18223810737148	0.392	0.288	3.8537430308647e-80	Endothelial Cell
ODF2L	4.48791461305388e-84	1.23277485357784	0.372	0.265	1.29790490609518e-79	Endothelial Cell
MIR99AHG	8.14716436507291e-83	1.30969856206266	0.551	0.484	2.35615993437909e-78	Endothelial Cell
GSK3B	1.78104327044372e-82	0.81252927273853	0.553	0.477	5.15077713812324e-78	Endothelial Cell
DOK6	5.35929893292859e-80	1.41045111941243	0.263	0.158	1.54990925140295e-75	Endothelial Cell
SMYD3	6.88370826833621e-80	0.681407057830419	0.676	0.621	1.99076843120283e-75	Endothelial Cell
STIM1	1.51020307221249e-79	1.15134249918635	0.301	0.191	4.36750728483852e-75	Endothelial Cell
CHSY1	1.55759924399524e-79	1.466695594	0.268	0.165	4.50457701363424e-75	Endothelial Cell
TANC2	3.3974692625323e-79	1.21387933632066	0.454	0.363	9.82548110724341e-75	Endothelial Cell
ADIPOR2	7.24196239104657e-79	1.34280552307769	0.338	0.236	2.09437552349067e-74	Endothelial Cell
NTN4	1.20965436689572e-78	1.58215154046304	0.386	0.293	3.49832042906242e-74	Endothelial Cell
SOX5	6.56955458066052e-78	1.15420992173435	0.4	0.287	1.89991518472702e-73	Endothelial Cell
FCHO2	1.10174222800691e-77	1.1556704159845	0.355	0.252	3.18623852339597e-73	Endothelial Cell
DDX5	4.31947045144757e-76	0.744156234088294	0.555	0.482	1.24919085455864e-71	Endothelial Cell
PLSCR1	4.74211602029099e-76	1.55653225308285	0.26	0.164	1.37141995306815e-71	Endothelial Cell
SFMBT2	8.20495826926918e-76	1.1617495380058	0.312	0.207	2.37287393147265e-71	Endothelial Cell
STIM2	9.89387572243725e-74	0.845465469493158	0.337	0.226	2.86130885892885e-69	Endothelial Cell
NEDD9	3.48176463644191e-72	0.883912623304616	0.382	0.271	1.006926332859e-67	Endothelial Cell
HIP1	4.45142980176528e-70	0.298274132526497	0.579	0.449	1.28735349867052e-65	Endothelial Cell
PCSK5	2.56775298686324e-68	1.0985540296335	0.371	0.257	7.42594163800849e-64	Endothelial Cell
RAPGEF2	6.40940428704343e-68	0.783060272959133	0.543	0.473	1.85359971981296e-63	Endothelial Cell
TBC1D5	7.45705207973043e-68	0.517090312028301	0.713	0.682	2.15657946145804e-63	Endothelial Cell
ST3GAL1	2.72894850405025e-67	0.90195553651361	0.297	0.191	7.89211907371333e-63	Endothelial Cell
ZSWIM6	3.84150273358398e-67	0.587685055647605	0.633	0.556	1.11096259055249e-62	Endothelial Cell
ELF2	1.57707745694242e-66	0.832542503509948	0.507	0.434	4.56090800547747e-62	Endothelial Cell
RNF213	3.24361319983214e-65	0.662108090970159	0.505	0.409	9.38052937391456e-61	Endothelial Cell
LINC00342	1.95280146119538e-64	0.860721795277353	0.378	0.274	5.64750182577705e-60	Endothelial Cell
EXOC6B	2.93511987833075e-64	0.664112431441536	0.615	0.567	8.48836668813253e-60	Endothelial Cell
EIF4G3	6.81841816685716e-64	0.660683777584336	0.591	0.529	1.97188653385509e-59	Endothelial Cell
PDLIM5	1.82603334615246e-62	0.771962967943255	0.482	0.403	5.28088843707291e-58	Endothelial Cell
DENND5A	1.83216550844568e-62	1.1269081116477	0.292	0.199	5.2986226504249e-58	Endothelial Cell
RGS12	3.07302291397481e-62	1.04116370715784	0.281	0.187	8.88718226721514e-58	Endothelial Cell

gene	p_val	avg_log2FC	pct.1	pct.2	p_val_adj	cluster
SENP7	3.16906769788902e-62	0.940035677061789	0.402	0.316	9.16494378229504e-58	Endothelial Cell
CYTH1	5.65526049033599e-62	0.941289823448196	0.386	0.294	1.63550133380517e-57	Endothelial Cell
RPH3AL	1.73388788372854e-61	1.29848591108158	0.27	0.181	5.01440375974293e-57	Endothelial Cell
VGLL4	2.61926032296835e-60	1.06439228527073	0.47	0.401	7.57490085402448e-56	Endothelial Cell
SSBP2	7.51134529013746e-60	0.805576847264078	0.429	0.337	2.17228105790775e-55	Endothelial Cell
CYFIP1	9.34752421701616e-60	1.0745311193474	0.308	0.218	2.70330400356107e-55	Endothelial Cell
MYH9	1.1826155619281e-59	0.843985050407352	0.412	0.321	3.42012420509606e-55	Endothelial Cell
MYO15B	1.72283997079245e-59	0.810376823657232	0.368	0.269	4.98245319553175e-55	Endothelial Cell
BAZ2B	1.27870750884562e-58	0.514572964010538	0.679	0.638	3.69802211558153e-54	Endothelial Cell
RHOA	2.61698643126606e-57	1.036029215	0.274	0.186	7.56832475922144e-53	Endothelial Cell
BBS9	4.34980571876752e-57	0.697960822810211	0.583	0.534	1.25796381386757e-52	Endothelial Cell
ANKRD36	1.32751689474468e-56	0.719509941864251	0.439	0.348	3.83917885960162e-52	Endothelial Cell
MID1	1.47359933687908e-56	0.825562763831544	0.275	0.18	4.2616492822543e-52	Endothelial Cell
PHACTR1	2.25125868594732e-56	0.472934947582679	0.297	0.194	6.51064011975966e-52	Endothelial Cell
MAP4	3.04560376837156e-55	0.677751303488377	0.578	0.526	8.80788609813055e-51	Endothelial Cell
ARHGEF12	4.85835647736832e-55	0.704017163716664	0.554	0.498	1.40503669325492e-50	Endothelial Cell
SMCHD1	7.23231454749565e-55	0.762380698897728	0.465	0.397	2.09158536713574e-50	Endothelial Cell
NCKAP1	1.20117560396812e-54	0.94553805164457	0.412	0.343	3.47379984667581e-50	Endothelial Cell
TBC1D15	1.20978181161022e-54	1.23624228707783	0.275	0.192	3.49868899917676e-50	Endothelial Cell
ACAP2	1.25061306329099e-54	0.851061936194183	0.425	0.353	3.61677297903754e-50	Endothelial Cell
CEP170	1.92310887352814e-54	1.05141386466165	0.253	0.167	5.56163086224338e-50	Endothelial Cell
NUMB	4.3797539988305e-54	0.670720919496024	0.541	0.489	1.26662485646178e-49	Endothelial Cell
LRRFIP1	2.66608786376094e-53	0.591458557655442	0.562	0.489	7.71032610199663e-49	Endothelial Cell
ERC1	3.37484902246408e-53	0.56786055758011	0.615	0.571	9.76006337296611e-49	Endothelial Cell
CDC14A	7.83504690799303e-51	0.533188638132916	0.275	0.185	2.26589556579159e-46	Endothelial Cell
EXOC6	1.78508264823637e-49	1.97944195485224	0.297	0.225	5.16245901869958e-45	Endothelial Cell
MGAT4A	6.60769895494694e-49	1.0624427019977	0.288	0.209	1.91094653777066e-44	Endothelial Cell
MT-CO1	1.31843663449178e-48	0.284277923688323	0.737	0.673	3.81291874695022e-44	Endothelial Cell
NUTM2A-AS1	6.51673534266928e-48	0.873075850731366	0.427	0.367	1.88463986109996e-43	Endothelial Cell
PPP1R12A	7.96164849091232e-48	0.659351744410718	0.492	0.438	2.30250874357184e-43	Endothelial Cell
SPRED2	1.39161509990622e-47	1.17621570373228	0.347	0.28	4.0245508689288e-43	Endothelial Cell
ADAM10	3.67120569979948e-47	0.719585166865652	0.448	0.378	1.06171268838201e-42	Endothelial Cell
AKAP13	5.25576650478876e-47	0.594904285025776	0.619	0.591	1.51996767318491e-42	Endothelial Cell
TBC1D22A	6.99418204183453e-47	0.597359790619248	0.552	0.502	2.02271744649855e-42	Endothelial Cell
ADD1	1.55639074042831e-46	0.757062372541069	0.429	0.362	4.50108202131867e-42	Endothelial Cell
KLHL2	2.81140756096776e-46	1.19649969579979	0.255	0.18	8.13059066631876e-42	Endothelial Cell
PPP3CC	5.20466460759553e-46	1.02060655531206	0.335	0.263	1.50518900451663e-41	Endothelial Cell
DOCK1	9.17892905452852e-46	0.492609969487724	0.629	0.583	2.65454628256965e-41	Endothelial Cell
HPCAL1	1.24276032700722e-45	0.891687789892157	0.342	0.26	3.59406286570489e-41	Endothelial Cell
PDS5B	2.7479567474311e-45	0.839376791914856	0.402	0.333	7.94709091357074e-41	Endothelial Cell
RDX	4.93295615104363e-45	0.561110521122447	0.396	0.32	1.42661091888182e-40	Endothelial Cell
DENND6A	8.256414259407e-45	1.13648677934945	0.253	0.18	2.3877550038205e-40	Endothelial Cell

gene	p_val	avg_log2FC	pct.1	pct.2	p_val_adj	cluster
LUZP1	3.97845587536822e-44	0.770437909311123	0.374	0.301	1.15056943915649e-39	Endothelial Cell
UPF3A	5.17476314870527e-43	1.01722734609655	0.296	0.224	1.49654150260556e-38	Endothelial Cell
SETX	4.50690929088065e-42	0.621425790009475	0.493	0.431	1.30339816692268e-37	Endothelial Cell
MBNL1	1.12857116443602e-41	0.513874396815903	0.636	0.608	3.26382780754897e-37	Endothelial Cell
PLCL2	1.38218448363963e-41	0.548070900689363	0.393	0.31	3.99727752668581e-37	Endothelial Cell
CTBP2	4.34757734751465e-41	0.660417618664635	0.518	0.471	1.25731936890124e-36	Endothelial Cell
ARHGEF7	9.02510964314215e-41	0.830729324561375	0.431	0.378	2.61006170879671e-36	Endothelial Cell
TMOD3	1.3032039557386e-40	0.984280244302479	0.309	0.24	3.76886583999604e-36	Endothelial Cell
NFIB	2.93096393685901e-40	0.563887443053558	0.594	0.535	8.47634770539625e-36	Endothelial Cell
SUN1	5.50212882170063e-40	1.16520989881799	0.299	0.234	1.59121565523582e-35	Endothelial Cell
BICD1	1.45760229446021e-39	0.573174181228925	0.385	0.306	4.21538583557894e-35	Endothelial Cell
PPP2R5A	2.82019614594417e-39	0.855726913973857	0.325	0.254	8.15600725407055e-35	Endothelial Cell
ALG13	1.11076064948865e-38	1.09396122489607	0.266	0.199	3.21231979832116e-34	Endothelial Cell
SORBS2	1.1271882953672e-38	0.571272109319856	0.579	0.54	3.25982855020193e-34	Endothelial Cell
UVRAG	1.2654460576565e-38	0.674297787014003	0.454	0.402	3.6596699987426e-34	Endothelial Cell
HIPK3	1.22984180561371e-37	0.784737137462537	0.409	0.35	3.55670250183486e-33	Endothelial Cell
FER	2.59388484412009e-36	0.641135211053077	0.488	0.441	7.50151496919529e-32	Endothelial Cell
CDC27	5.29196626750075e-36	1.00060840474807	0.28	0.216	1.53043664456122e-31	Endothelial Cell
PHF14	6.10969413874645e-36	0.760652430425893	0.393	0.335	1.76692354492547e-31	Endothelial Cell
CACNA2D1	1.29845798407449e-35	0.842146114854937	0.365	0.302	3.75514048994343e-31	Endothelial Cell
MAN2A1	2.65354785324069e-34	0.818468127276295	0.399	0.347	7.67406039157208e-30	Endothelial Cell
ADAM17	3.42598164034429e-34	1.05350718560042	0.251	0.189	9.9079389038757e-30	Endothelial Cell
MAP4K5	1.12242067542418e-33	0.83470308325584	0.339	0.281	3.24604059332673e-29	Endothelial Cell
ATP13A3	1.23109205301687e-33	1.67037822858858	0.287	0.233	3.5603182173248e-29	Endothelial Cell
SPRED1	3.07812894947149e-33	1.03865975025654	0.282	0.221	8.90194892187156e-29	Endothelial Cell
ZNF800	3.85948374028396e-33	0.951068078110893	0.259	0.195	1.11616269769012e-28	Endothelial Cell
MYCBP2	3.24729038725957e-31	0.523949618631409	0.494	0.46	9.39116379995468e-27	Endothelial Cell
SLK	8.61723881911126e-30	0.790137499143939	0.312	0.253	2.49210546648698e-25	Endothelial Cell
KIAA1217	9.50055367320834e-30	0.54297469803848	0.649	0.649	2.74756012229185e-25	Endothelial Cell
ARHGAP17	1.14301196104982e-29	0.934277467319998	0.266	0.208	3.30559059135607e-25	Endothelial Cell
TIAM2	9.32538230034264e-29	0.838300958653602	0.344	0.294	2.69690056125909e-24	Endothelial Cell
COP1	1.18094787878159e-28	0.541971522032791	0.492	0.468	3.41530126543635e-24	Endothelial Cell
SMARCA2	1.29434035689674e-28	0.603978531115134	0.398	0.348	3.74323231214537e-24	Endothelial Cell
ITGB1	1.51577976365133e-28	0.776206306262508	0.289	0.233	4.38363507647964e-24	Endothelial Cell
TRIO	1.69386468322646e-28	0.450151732222421	0.57	0.541	4.89865666389092e-24	Endothelial Cell
SUSD6	1.77835523769401e-28	0.923075201377405	0.297	0.244	5.14300334741106e-24	Endothelial Cell
NLK	1.8837251537678e-28	0.616563059583304	0.444	0.399	5.44773314469648e-24	Endothelial Cell
RAPGEF3	3.25682492283627e-28	0.489148343960453	0.318	0.251	9.4187376768425e-24	Endothelial Cell
PPARG	8.70915233506607e-28	0.753495093350738	0.329	0.268	2.51868685530111e-23	Endothelial Cell
CCNH	1.39838003893462e-27	0.746843868625691	0.424	0.387	4.04411507259891e-23	Endothelial Cell
FOXO1	1.83862827410062e-27	0.484640506220309	0.514	0.47	5.31731296869899e-23	Endothelial Cell
CAMSAP2	8.51936984250996e-27	0.725965694644729	0.34	0.289	2.46380175845388e-22	Endothelial Cell

gene	p_val	avg_log2FC	pct.1	pct.2	p_val_adj	cluster
CNTLN	9.09538153032061e-27	0.767561846028994	0.293	0.236	2.63038433856872e-22	Endothelial Cell
RAB2A	1.56291118900333e-26	0.647201984488021	0.421	0.384	4.51993915859762e-22	Endothelial Cell
IQGAP1	1.93547310369925e-26	0.734639089568842	0.347	0.296	5.59738821589822e-22	Endothelial Cell
PTBP2	2.24470044655096e-26	0.709019405214924	0.396	0.356	6.49167369142537e-22	Endothelial Cell
ITM2B	2.96601350741619e-26	0.631680157539083	0.377	0.329	8.57771106344763e-22	Endothelial Cell
SVIL	3.89814759151755e-26	0.517038084762341	0.457	0.407	1.12734428346687e-21	Endothelial Cell
CDC42BPB	9.42305284366811e-26	0.663834121122504	0.388	0.341	2.72514688238882e-21	Endothelial Cell
CTNNA1	1.63287753275998e-25	0.506321721247464	0.51	0.483	4.72228182474185e-21	Endothelial Cell
ROCK1	7.71507107198859e-24	0.582772406924956	0.443	0.411	2.2311985540191e-19	Endothelial Cell
MKLN1	1.82884121282092e-23	0.442038447357159	0.571	0.556	5.28900878747811e-19	Endothelial Cell
VPS13C	2.38045997435583e-23	0.467653029698439	0.475	0.44	6.88429024583707e-19	Endothelial Cell
SKAP2	2.51441390052936e-23	0.601704125421752	0.362	0.313	7.27168500033091e-19	Endothelial Cell
CCND3	2.63443570357166e-23	0.662257692791912	0.274	0.219	7.61878805472925e-19	Endothelial Cell
REEP3	3.27700690986692e-23	1.06200445694962	0.257	0.21	9.47710398333514e-19	Endothelial Cell
AGTPBP1	4.481701834015e-23	0.934180964750851	0.261	0.214	1.29610817039714e-18	Endothelial Cell
ZNF532	2.02934465709252e-22	0.863613438944066	0.278	0.232	5.86886474831158e-18	Endothelial Cell
MAP3K3	2.65993069007238e-22	0.805986643407685	0.258	0.208	7.69251955568932e-18	Endothelial Cell
PPP6R3	4.72657862441452e-22	0.438210418356662	0.544	0.528	1.36692653818068e-17	Endothelial Cell
ATF7	9.82132613783455e-22	0.752265037566894	0.28	0.234	2.84032751906175e-17	Endothelial Cell
ARID4B	1.07499988469118e-21	0.448445972974602	0.511	0.492	3.10889966652688e-17	Endothelial Cell
ZFAND3	1.34154584557368e-21	0.280023361812532	0.722	0.708	3.87975058539908e-17	Endothelial Cell
CEP350	2.10687096404065e-21	0.49407693888085	0.449	0.425	6.09307082800556e-17	Endothelial Cell
MYO1E	2.328424038647e-21	0.407489193709025	0.45	0.409	6.73380231976713e-17	Endothelial Cell
SOS1	3.46014715577525e-21	0.55516526748309	0.484	0.471	1.0006745574502e-16	Endothelial Cell
FBXO11	3.70813844562919e-21	0.612035788587491	0.43	0.404	1.07239363847596e-16	Endothelial Cell
CDKAL1	6.76947284593862e-21	0.42991970638719	0.561	0.55	1.95773154704545e-16	Endothelial Cell
UBR1	8.56609143310945e-21	0.692868730277136	0.354	0.32	2.47731364245525e-16	Endothelial Cell
TSPAN9	4.64446051566834e-20	0.396831407344022	0.517	0.497	1.34317798113128e-15	Endothelial Cell
PBX3	5.80877459048891e-20	0.821469968168167	0.262	0.214	1.67989761156939e-15	Endothelial Cell
KCNIP4	8.65323213074675e-20	0.402972378750414	0.477	0.562	2.50251473221196e-15	Endothelial Cell
SPAG9	1.12630130682548e-19	0.379973899556293	0.526	0.5	3.25726337933929e-15	Endothelial Cell
CHIC2	1.13913154263345e-19	0.910470996237776	0.26	0.218	3.29436842129593e-15	Endothelial Cell
ATG7	1.56897701225608e-19	0.690022547163551	0.402	0.378	4.53748151944458e-15	Endothelial Cell
SPECC1L	1.72689345233496e-19	0.59296677021256	0.388	0.354	4.99417586415271e-15	Endothelial Cell
SLC25A37	1.97075263019657e-19	0.897578702390373	0.266	0.224	5.69941660652848e-15	Endothelial Cell
MT-CO3	4.25752512891088e-19	0.309675037439611	0.496	0.452	1.23127626728103e-14	Endothelial Cell
FAM120B	2.43602788951394e-18	0.534920860212247	0.441	0.42	7.04499265647432e-14	Endothelial Cell
PITPNM2	1.18661181410555e-17	0.834374851204753	0.292	0.254	3.43168136639326e-13	Endothelial Cell
RTN4	1.23572853433906e-17	0.533398567007192	0.396	0.368	3.57372692130856e-13	Endothelial Cell
WAC	1.69817077979121e-17	0.514272522553787	0.464	0.451	4.91110989515619e-13	Endothelial Cell
CNOT2	2.14631375458608e-17	0.707261607997258	0.353	0.328	6.20713937826295e-13	Endothelial Cell
EXOC4	5.93518354650921e-17	0.302327645581355	0.666	0.657	1.71645508165046e-12	Endothelial Cell

gene	p_val	avg_log2FC	pct.1	pct.2	p_val_adj	cluster
GOLPH3	9.66327481289965e-17	0.78040483948641	0.275	0.24	2.79461907589058e-12	Endothelial Cell
PAM	1.5625586916172e-16	0.517883595782281	0.447	0.425	4.51891973615696e-12	Endothelial Cell
DAB2IP	2.77775206460667e-16	0.792156219740111	0.253	0.214	8.03325897084248e-12	Endothelial Cell
NIPBL	6.45348765371477e-16	0.358491221370128	0.575	0.575	1.86634862945431e-11	Endothelial Cell
LINC00486	8.05072162465941e-16	0.609669228161446	0.309	0.268	2.3282686938515e-11	Endothelial Cell
GMDS	9.78465452860375e-16	0.557916280659666	0.411	0.386	2.82972208967220e-11	Endothelial Cell
PHKB	1.64995631251067e-15	0.572749402927654	0.41	0.396	4.77167365578086e-11	Endothelial Cell
FBXW7	1.66243366779364e-15	0.568654176188649	0.391	0.371	4.8077581672592e-11	Endothelial Cell
PAN3	1.79452090238738e-15	0.394742715552516	0.538	0.534	5.18975444970431e-11	Endothelial Cell
AFDN	2.15570623607922e-15	0.342265412637269	0.568	0.571	6.2343024347411e-11	Endothelial Cell
FAM214A	2.28153511934908e-15	0.758898676076466	0.329	0.304	6.59819956515753e-11	Endothelial Cell
PCM1	2.63491349020434e-15	0.390510309775204	0.552	0.549	7.62016981367095e-11	Endothelial Cell
XPR1	4.42671622250816e-15	0.536515902343569	0.425	0.406	1.28020633154936e-10	Endothelial Cell
SPIDR	4.47167072292123e-15	0.318496091057388	0.613	0.611	1.29320717306882e-10	Endothelial Cell
RAP1A	5.20521656306625e-15	0.597166255594924	0.337	0.309	1.50534863003876e-10	Endothelial Cell
ATP11A	6.43996541156482e-15	0.433210270827999	0.312	0.267	1.86243799702455e-10	Endothelial Cell
DYNC1H1	8.26653337727385e-15	0.598265017655956	0.294	0.258	2.3906814527076e-10	Endothelial Cell
NCOA1	1.8094698471519e-14	0.377815626473555	0.562	0.562	5.23298679796329e-10	Endothelial Cell
INPP5A	6.02422476396061e-14	0.670887258357974	0.271	0.236	1.74220580173741e-09	Endothelial Cell
EPS15	8.5688814037246e-14	0.502078382738343	0.432	0.42	2.47812050195715e-09	Endothelial Cell
ANKRD11	9.83526715543588e-14	0.304438193714274	0.593	0.591	2.84435926135206e-09	Endothelial Cell
PPP3CA	1.27659054514041e-13	0.644573370433495	0.424	0.41	3.69189985654605e-09	Endothelial Cell
PITPNB	1.496743186247e-13	0.794650835003597	0.263	0.234	4.32858129462632e-09	Endothelial Cell
NFKB1	2.39149360662412e-13	0.736659207607764	0.264	0.232	6.91619951035694e-09	Endothelial Cell
RPS6KA3	3.92971031327054e-13	0.605351293518378	0.258	0.223	1.13647222259784e-08	Endothelial Cell
CTNND1	4.22966341300331e-13	0.268124219323885	0.633	0.626	1.22321865904056e-08	Endothelial Cell
DENND4C	1.52542155457213e-12	0.623537538528501	0.386	0.379	4.41151913582259e-08	Endothelial Cell
KMT2E	1.55590828957437e-12	0.346699343501745	0.518	0.516	4.49968677344909e-08	Endothelial Cell
MLLT3	1.66621189992165e-12	0.597629208639276	0.388	0.368	4.8186848145734e-08	Endothelial Cell
TMEM135	2.17505724772062e-12	0.629159052262092	0.338	0.319	6.29026556040804e-08	Endothelial Cell
HERC4	4.7928710455188e-12	0.407182178639969	0.496	0.498	1.38609830636404e-07	Endothelial Cell
SPTAN1	5.90140931622134e-12	0.57150012739525	0.307	0.281	1.70668757425121e-07	Endothelial Cell
UBE2D3	6.69711245453197e-12	0.571284682318806	0.413	0.404	1.93680492185065e-07	Endothelial Cell
JAK2	2.56105645810242e-11	0.461183219422198	0.309	0.281	7.40657527683221e-07	Endothelial Cell
SETD5	3.03973392872475e-11	0.474546044392162	0.432	0.43	8.79091052187199e-07	Endothelial Cell
RPRD1A	2.04155883863078e-10	0.836006235509143	0.256	0.235	5.9041881613202e-06	Endothelial Cell
MT-ND1	2.19744729864033e-10	0.300252759126173	0.368	0.335	6.35501758766782e-06	Endothelial Cell
POLK	2.34702994922687e-10	0.595405000292155	0.301	0.281	6.7876106131641e-06	Endothelial Cell
CMIP	4.13144637292352e-10	0.364609537118289	0.469	0.456	1.19481429104948e-05	Endothelial Cell
TASOR2	5.4481689857599e-10	0.546476865117183	0.34	0.325	1.57561047068176e-05	Endothelial Cell
NFAT5	5.5015370434522e-10	0.253055213452543	0.588	0.588	1.59104451296638e-05	Endothelial Cell
BMERB1	1.32958941180499e-09	0.83574366746152	0.279	0.262	3.84517257894004e-05	Endothelial Cell

gene	p_val	avg_log2FC	pct.1	pct.2	p_val_adj	cluster
NCOR2	1.39358968774650e-09	0.573073530172839	0.317	0.296	4.03026137696286e-05	Endothelial Cell
TBC1D1	2.10777756043978e-09	0.346983285714002	0.496	0.492	6.09569270479183e-05	Endothelial Cell
ICA1	2.20344770918212e-09	0.273247532614272	0.362	0.33	6.3723707749547e-05	Endothelial Cell
ZNF407	2.46479445613401e-09	0.453793416155223	0.452	0.453	7.12818556713957e-05	Endothelial Cell
PPP2R2A	2.67274822560103e-09	0.557618412926763	0.346	0.337	7.72958786843819e-05	Endothelial Cell
MT-ND5	2.74600654816346e-09	0.277363011363046	0.336	0.305	7.94145093728874e-05	Endothelial Cell
AHNAK	2.8611525589533e-09	0.480682357295235	0.264	0.235	8.27445320049295e-05	Endothelial Cell
DYRK1A	2.95264706497951e-09	0.396393625262145	0.502	0.509	8.53905531192074e-05	Endothelial Cell
ACER3	4.03118454642892e-09	0.657491125404688	0.256	0.234	0.000116581857082724	Endothelial Cell
UNC13B	5.17289778626401e-09	0.493812135724082	0.387	0.382	0.000149600203978755	Endothelial Cell
PCNX1	6.59982457888326e-09	0.473600274668716	0.424	0.427	0.000190866926821304	Endothelial Cell
CAST	8.72009477084397e-09	0.454972101659509	0.36	0.35	0.000252185140772808	Endothelial Cell
MIRLET7BHG	1.08235827413281e-08	0.591768780817874	0.279	0.26	0.000313018012879209	Endothelial Cell
SEPTIN7	1.29866231650484e-08	0.61822346879445	0.282	0.265	0.000375573141933201	Endothelial Cell
LYST	1.43788132012712e-08	0.634806058129963	0.311	0.3	0.000415835277780763	Endothelial Cell
FTO	1.60771635166774e-08	0.356439179125218	0.485	0.488	0.000464951568902309	Endothelial Cell
NSRP1	2.20446715386767e-08	0.512946300565648	0.346	0.338	0.00063753190089853	Endothelial Cell
SMC5	2.23489517211421e-08	0.556320238228548	0.327	0.317	0.000646331683775428	Endothelial Cell
CAMK2D	3.72088614799489e-08	0.507527100411986	0.327	0.313	0.00107608027400012	Endothelial Cell
BIRC6	5.15937583979938e-08	0.260811794110464	0.59	0.602	0.00149209149286998	Endothelial Cell
CSNK1A1	6.94783084733239e-08	0.358826556887849	0.487	0.493	0.00200931268104853	Endothelial Cell
SEC24B	8.09573621984571e-08	0.560777410955013	0.321	0.313	0.00234128691477938	Endothelial Cell
TMEM165	9.20749809887114e-08	0.649831773373539	0.274	0.26	0.00266280845019353	Endothelial Cell
LRCH3	1.04378670456625e-07	0.614029961783266	0.311	0.302	0.00301863114960559	Endothelial Cell
PHF21A	1.39983581243583e-07	0.552192692664835	0.358	0.354	0.00404832516956443	Endothelial Cell
CRPPA	1.58432192514901e-07	0.689589080050344	0.28	0.263	0.00458185900753093	Endothelial Cell
RASA1	1.6243416335339e-07	0.521546011167151	0.331	0.324	0.00469759600418004	Endothelial Cell
MARK3	1.729603178267e-07	0.377090018442755	0.474	0.488	0.00500201239154817	Endothelial Cell
FAM172A	1.99073877063565e-07	0.343115025112879	0.511	0.526	0.00575721652467831	Endothelial Cell
SH3BGRL	3.24698158491572e-07	0.433780865008324	0.271	0.249	0.00939027074357626	Endothelial Cell
WASHC4	3.46756556383917e-07	0.557028547413824	0.308	0.298	0.0100281996106229	Endothelial Cell
ASXL1	3.52678350406119e-07	0.433350803183093	0.365	0.358	0.0101994578937449	Endothelial Cell
EPC1	4.96399521737858e-07	0.452909211591626	0.381	0.382	0.0143558741686589	Endothelial Cell
PDCD6IP	5.12232878017157e-07	0.625191133601776	0.284	0.274	0.0148137748322562	Endothelial Cell
PPP3R1	7.57268793899356e-07	0.582118507623013	0.298	0.286	0.0219002135195694	Endothelial Cell
RRBP1	1.1576566039485e-06	0.409005871187161	0.341	0.325	0.0334794289861907	Endothelial Cell
KDM6A	1.58856526261112e-06	0.400351231447507	0.442	0.451	0.0459413073947137	Endothelial Cell
LONP2	2.4780924261851e-06	0.468497305563009	0.38	0.387	0.0716664329652731	Endothelial Cell
ARIH1	2.52828538689189e-06	0.371019132940147	0.469	0.48	0.0731180133889134	Endothelial Cell
COMMD10	3.21436438724843e-06	0.287128437187448	0.539	0.56	0.0929594180792247	Endothelial Cell
SPTLC2	3.41713966335026e-06	0.541889476232515	0.252	0.237	0.0988236790640896	Endothelial Cell
DYM	3.5811016329156e-06	0.3521794214122	0.47	0.482	0.103565459223919	Endothelial Cell

gene	p_val	avg_log2FC	pct.1	pct.2	p_val_adj	cluster
ZNF280D	3.82710515811537e-06	0.443702239996146	0.32	0.313	0.110679881172697	Endothelial Cell
SRSF11	4.12541258094037e-06	0.290444074000952	0.485	0.493	0.119306931840795	Endothelial Cell
UBR5	4.73421692019271e-06	0.30090574632819	0.474	0.488	0.136913553331973	Endothelial Cell
EEF1A1	4.82489473203699e-06	0.39683914461844	0.283	0.266	0.13953595565051	Endothelial Cell
ARID2	4.86630548882103e-06	0.412360860075019	0.411	0.421	0.140733554736704	Endothelial Cell
7-Mar	5.22050847754445e-06	0.54055393386136	0.323	0.32	0.150977105170585	Endothelial Cell
CCNL2	7.61630495709108e-06	0.45330424657067	0.321	0.31	0.220263539359074	Endothelial Cell
RPS6KC1	1.02648669829864e-05	0.534905322996526	0.318	0.315	0.296859953147966	Endothelial Cell
FAM120A	1.12598274486994e-05	0.591964349149759	0.276	0.269	0.325634209816387	Endothelial Cell
B4GALT5	1.12709629970267e-05	0.545091404790859	0.283	0.276	0.325956249874013	Endothelial Cell
TBL1XR1	1.3854570497746e-05	0.280123545918642	0.448	0.454	0.400674178794815	Endothelial Cell
NPHP3	1.58171237407812e-05	0.510206604579968	0.27	0.259	0.457431218583391	Endothelial Cell
PSPC1	1.63682657583003e-05	0.48020184592266	0.344	0.345	0.473370245730043	Endothelial Cell
PRKCA	1.69346165914085e-05	0.52970354464042	0.513	0.606	0.489749111823533	Endothelial Cell
PHACTR2	2.65644019177963e-05	0.372697709847749	0.323	0.313	0.768242503462668	Endothelial Cell
EHMT1	3.7867623760736e-05	0.322285012954481	0.438	0.449	1	Endothelial Cell
TAF1	3.91901420227445e-05	0.585405027822234	0.259	0.25	1	Endothelial Cell
MCPH1	4.03342257502327e-05	0.521508567117278	0.305	0.301	1	Endothelial Cell
MTUS1	4.2346640582158e-05	0.617402671499191	0.346	0.352	1	Endothelial Cell
JAK1	5.11237866520144e-05	0.263485119763091	0.504	0.516	1	Endothelial Cell
ZNF462	6.75744297827256e-05	0.471086821740714	0.25	0.237	1	Endothelial Cell
TLK2	7.61947771861219e-05	0.466402519186244	0.317	0.318	1	Endothelial Cell
YAF2	9.88539104986731e-05	0.559001838828396	0.278	0.274	1	Endothelial Cell
FLNB	0.000108082409341898	0.531342534001455	0.332	0.334	1	Endothelial Cell
VPS54	0.000137515874096709	0.441599138020804	0.346	0.349	1	Endothelial Cell
CMSS1	0.000169577734813062	0.269648528371839	0.21	0.254	1	Endothelial Cell
ASCC3	0.000177286639883958	0.457671212025247	0.341	0.345	1	Endothelial Cell
RAB3GAP1	0.000196277754038046	0.462405675761365	0.338	0.344	1	Endothelial Cell
SPATA5	0.000237711878326465	0.527111068624434	0.275	0.27	1	Endothelial Cell
RSRC1	0.000251989132388538	0.471354940979657	0.345	0.352	1	Endothelial Cell
EXT1	0.000277659453321434	0.619618993761564	0.504	0.536	1	Endothelial Cell
ZNF207	0.000286432363114945	0.443448319713613	0.351	0.359	1	Endothelial Cell
NAA15	0.000321715273415716	0.261741878049889	0.22	0.266	1	Endothelial Cell
NEK7	0.00032897504504949	0.464617294592098	0.345	0.353	1	Endothelial Cell
CTIF	0.0003516622604804	0.512984676613774	0.256	0.249	1	Endothelial Cell
STXBP5	0.00037813159071811	0.460146502411305	0.321	0.322	1	Endothelial Cell
WDR7	0.000398305222103676	0.5245628403234	0.318	0.329	1	Endothelial Cell
ZMIZ1	0.000420690799664096	0.426458848442992	0.363	0.368	1	Endothelial Cell
RYK	0.000494192092333663	0.616723916101856	0.266	0.265	1	Endothelial Cell
RSF1	0.000537983087897102	0.313390350554289	0.398	0.412	1	Endothelial Cell
RANBP9	0.000572361627408171	0.274708784876927	0.21	0.253	1	Endothelial Cell
PHF20L1	0.000647488841420124	0.487779586045591	0.296	0.297	1	Endothelial Cell

gene	p_val	avg_log2FC	pct.1	pct.2	p_val_adj	cluster
UBE2E1	0.000694119816508311	0.541398246384056	0.298	0.303	1	Endothelial Cell
LNX1	0.000813221613459176	0.706144735528712	0.206	0.254	1	Endothelial Cell
CCSER2	0.000895471276687633	0.377283403575503	0.375	0.385	1	Endothelial Cell
PTPRA	0.00123634788889722	0.47919992777471	0.311	0.317	1	Endothelial Cell
MAP2K5	0.00161954590990105	0.441699745730869	0.324	0.331	1	Endothelial Cell
KPNA4	0.00178259921720464	0.272537798834954	0.214	0.254	1	Endothelial Cell
PHACTR4	0.00192011607660026	0.304632035243764	0.394	0.405	1	Endothelial Cell
FNBP4	0.00194815328190285	0.376806481656785	0.339	0.346	1	Endothelial Cell
MECP2	0.00220763898340864	0.559344178773134	0.267	0.269	1	Endothelial Cell
STAT3	0.00276139474726736	0.372302092042932	0.407	0.422	1	Endothelial Cell
ATG5	0.00322032814445736	0.265741738963256	0.213	0.251	1	Endothelial Cell
GNAS	0.00328735217143763	0.403495566725438	0.283	0.284	1	Endothelial Cell
WAPL	0.00407557734558455	0.454241316460256	0.302	0.312	1	Endothelial Cell
NUTM2B-AS1	0.00456849043925611	0.478550575682439	0.326	0.338	1	Endothelial Cell
MPDZ	0.00480227100063998	0.486995220105451	0.277	0.281	1	Endothelial Cell
CRIM1	0.00480771478120002	0.329331202340685	0.411	0.421	1	Endothelial Cell
SRSF4	0.0053292218537485	0.413068916985026	0.394	0.413	1	Endothelial Cell
CDK6	0.00605012937312906	0.257340985983324	0.306	0.3	1	Endothelial Cell
SRRM1	0.00660942419597309	0.342659310741613	0.286	0.288	1	Endothelial Cell
XRN1	0.00676632978635496	0.281386089597321	0.416	0.437	1	Endothelial Cell
AKAP10	0.00694521216893376	0.285379316594437	0.232	0.272	1	Endothelial Cell
RNF38	0.00754868690831419	0.34664456190324	0.397	0.417	1	Endothelial Cell
UBE4B	0.00934198037983867	0.414484227017138	0.311	0.32	1	Endothelial Cell
NPHS1	0	5.994507472	0.735	0.037	0	Podocyte
PTPRO	0	7.493724026	0.779	0.09	0	Podocyte
NPHS2	0	6.68934876503678	0.727	0.039	0	Podocyte
PTPRQ	0	7.37217676043471	0.775	0.091	0	Podocyte
NTNG1	0	7.09890724631591	0.723	0.039	0	Podocyte
CLIC5	0	5.76441449596063	0.758	0.078	0	Podocyte
FMN2	0	7.00971097765076	0.698	0.022	0	Podocyte
ATP10A	0	5.47778104600657	0.707	0.038	0	Podocyte
PLCE1	0	5.13412568731034	0.739	0.085	0	Podocyte
FGF1	0	6.62393281635417	0.68	0.032	0	Podocyte
ADAMTS19	0	6.93980694359359	0.667	0.021	0	Podocyte
PLA2R1	0	6.32701975019928	0.782	0.141	0	Podocyte
TARID	0	7.02223499348561	0.653	0.015	0	Podocyte
NFASC	0	4.81941558449498	0.675	0.041	0	Podocyte
ALS2CL	0	5.34499668053362	0.687	0.054	0	Podocyte
ST6GALNAC3	0	5.16476023225526	0.775	0.144	0	Podocyte
ABLIM2	0	5.17328105958006	0.671	0.043	0	Podocyte
CR1	0	6.30858731745429	0.633	0.012	0	Podocyte
RYR3	0	4.95323043518684	0.648	0.032	0	Podocyte

gene	p_val	avg_log2FC	pct.1	pct.2	p_val_adj	cluster
PODXL	0	4.08840931094041	0.726	0.114	0	Podocyte
WT1	0	5.24556246753876	0.636	0.026	0	Podocyte
SPOCK1	0	4.34345371072153	0.692	0.092	0	Podocyte
CCBE1	0	4.62959909213557	0.635	0.048	0	Podocyte
TENM2	0	4.61120747863703	0.63	0.046	0	Podocyte
AC092813.2	0	7.67816345579745	0.59	0.007	0	Podocyte
AC109466.1	0	5.90182314044337	0.609	0.027	0	Podocyte
ARHGAP28	0	4.26199431473281	0.685	0.106	0	Podocyte
SRGAP2	0	3.99796645188557	0.68	0.109	0	Podocyte
DACH2	0	6.60374528343518	0.583	0.013	0	Podocyte
MAGI2-AS3	0	4.3826809796092	0.715	0.146	0	Podocyte
FYN	0	3.25635747326727	0.645	0.079	0	Podocyte
KLHL29	0	4.20365312152149	0.68	0.116	0	Podocyte
ZDHHC6	0	4.30946819798396	0.65	0.092	0	Podocyte
NPAS3	0	4.49416094236882	0.751	0.2	0	Podocyte
SRGAP2C	0	4.28329907043162	0.628	0.078	0	Podocyte
SRGAP2B	0	4.09280483869957	0.625	0.085	0	Podocyte
SULF1	0	3.66172848858779	0.581	0.043	0	Podocyte
LINC02798	0	5.88346739978348	0.55	0.013	0	Podocyte
RIPOR1	0	3.47316992956829	0.658	0.121	0	Podocyte
ARHGEF26	0	4.8674320626518	0.575	0.039	0	Podocyte
ZBTB7C	0	4.08126375270305	0.57	0.041	0	Podocyte
LRRC2	0	5.02720985298459	0.551	0.027	0	Podocyte
ARMH4	0	3.85007589490636	0.694	0.17	0	Podocyte
CDC14A	0	4.07877814890556	0.696	0.173	0	Podocyte
KIRREL1	0	3.2701979638411	0.63	0.11	0	Podocyte
TGFBR3	0	3.26543177228626	0.707	0.189	0	Podocyte
PCOLCE2	0	5.75798378058206	0.527	0.013	0	Podocyte
UNC5C	0	2.8170217504874	0.631	0.119	0	Podocyte
SEMA5A	0	3.49693021036293	0.728	0.217	0	Podocyte
ST3GAL6	0	3.51191826552344	0.706	0.196	0	Podocyte
TCF7L1	0	2.77651820596408	0.63	0.121	0	Podocyte
NECAB1	0	5.24197804601391	0.525	0.017	0	Podocyte
WIPF3	0	4.40179495585804	0.543	0.037	0	Podocyte
VEGFA	0	3.20859379102856	0.736	0.233	0	Podocyte
DOCK5	0	3.47757949571992	0.702	0.199	0	Podocyte
TYRO3	0	5.41051005477931	0.515	0.013	0	Podocyte
TENM3	0	3.24808657906284	0.578	0.078	0	Podocyte
ROBO2	0	2.58715868121994	0.574	0.079	0	Podocyte
GRK5	0	3.33683651191861	0.55	0.056	0	Podocyte
NPNT	0	3.47084348316791	0.687	0.194	0	Podocyte
FBXL7	0	2.4686282131988	0.613	0.125	0	Podocyte

gene	p_val	avg_log2FC	pct.1	pct.2	p_val_adj	cluster
IQCJ-SCHIP1	0	4.40813113457811	0.66	0.175	0	Podocyte
PCED1B	0	2.59375463961996	0.604	0.119	0	Podocyte
DPP6	0	3.93660217109435	0.753	0.269	0	Podocyte
WDR49	0	6.73781889515112	0.488	0.008	0	Podocyte
LINC00839	0	5.94672950349006	0.49	0.011	0	Podocyte
INTS12	0	3.64388661131344	0.594	0.117	0	Podocyte
ZNF804A	0	5.95201845336602	0.491	0.015	0	Podocyte
PARD6G	0	4.24793347971624	0.503	0.031	0	Podocyte
FRY	0	2.84678027562911	0.72	0.251	0	Podocyte
LINC02149	0	6.70106346901763	0.469	0.006	0	Podocyte
MSRB3	0	3.08838083160705	0.51	0.049	0	Podocyte
AL136456.1	0	3.80039691316068	0.491	0.031	0	Podocyte
KIAA1211	0	4.70877193357043	0.476	0.018	0	Podocyte
WT1-AS	0	5.46108199076132	0.469	0.011	0	Podocyte
RBFOX1	0	2.41930769165677	0.68	0.224	0	Podocyte
TSPAN2	0	4.86866613310304	0.47	0.016	0	Podocyte
MME	0	2.17154297048355	0.765	0.316	0	Podocyte
ITGA9-AS1	0	2.66051520063309	0.604	0.155	0	Podocyte
EXPH5	0	2.6769280228101	0.633	0.185	0	Podocyte
ANKRD33B	0	2.36067342601118	0.599	0.153	0	Podocyte
CERS6	0	2.81610053316771	0.647	0.204	0	Podocyte
LMX1B	0	6.14571370125798	0.449	0.006	0	Podocyte
SRGAP1	0	2.78699715773384	0.707	0.264	0	Podocyte
MPP5	0	2.75986221887321	0.645	0.203	0	Podocyte
KLF7	0	2.2677167908593	0.602	0.162	0	Podocyte
AC008415.1	0	5.11962670504575	0.46	0.021	0	Podocyte
SERINC5	0	2.52808487591343	0.599	0.16	0	Podocyte
NDNF	0	4.96406463499734	0.457	0.019	0	Podocyte
PTPRD	0	2.46662036531367	0.758	0.32	0	Podocyte
GRIK2	0	3.54888772706301	0.486	0.051	0	Podocyte
SFMBT2	0	2.31147115607759	0.632	0.199	0	Podocyte
CLIP4	0	2.77563614342122	0.593	0.16	0	Podocyte
IQGAP2	0	2.71702374000177	0.755	0.323	0	Podocyte
PTGDS	0	3.97733370128648	0.466	0.036	0	Podocyte
SGIP1	0	2.01275999226949	0.642	0.216	0	Podocyte
MYO1B	0	1.90748849481668	0.653	0.229	0	Podocyte
CORO2B	0	3.83195689588125	0.461	0.037	0	Podocyte
NKD1	0	5.73654232007291	0.431	0.007	0	Podocyte
DYNC1I1	0	4.48147008633709	0.448	0.025	0	Podocyte
ANXA2	0	2.77317877705985	0.514	0.092	0	Podocyte
RERG	0	2.05570151470328	0.657	0.236	0	Podocyte
FRMD1	0	2.2754412782885	0.478	0.062	0	Podocyte

gene	p_val	avg_log2FC	pct.1	pct.2	p_val_adj	cluster
MGAT5	0	2.67038960223962	0.703	0.288	0	Podocyte
ITGB8	0	2.06796198375567	0.691	0.279	0	Podocyte
FIGN	0	2.17949255593375	0.539	0.128	0	Podocyte
PSD3	0	1.60753454913474	0.579	0.168	0	Podocyte
CACNB2	0	1.39971838435834	0.637	0.229	0	Podocyte
DACH1	0	3.66958807851255	0.787	0.379	0	Podocyte
ROBO1	0	1.7201048775615	0.5	0.093	0	Podocyte
ENPEP	0	1.75439747412293	0.711	0.304	0	Podocyte
ARRDC4	0	3.33429663252413	0.481	0.078	0	Podocyte
AC023154.1	0	6.81284028428075	0.405	0.003	0	Podocyte
RETREG1	0	1.84921008383452	0.661	0.262	0	Podocyte
CA10	0	1.84431896945792	0.626	0.229	0	Podocyte
PRDM5	0	2.72917214684912	0.5	0.105	0	Podocyte
CTTNBP2	0	1.49414602813002	0.58	0.185	0	Podocyte
ZFPM2	0	1.96638644442731	0.476	0.081	0	Podocyte
SNX25	0	2.49557948098652	0.615	0.221	0	Podocyte
TRPC6	0	4.05829746817225	0.427	0.033	0	Podocyte
KLHL42	0	2.73448464426114	0.488	0.094	0	Podocyte
C1orf21	0	2.9937896387999	0.449	0.057	0	Podocyte
LINC02006	0	4.90070756534085	0.418	0.026	0	Podocyte
HTRA1	0	3.66542071924281	0.424	0.034	0	Podocyte
TMEM266	0	4.86680064067753	0.406	0.016	0	Podocyte
MAP6	0	4.52826928875704	0.41	0.021	0	Podocyte
PAK1	0	2.05615479113771	0.571	0.183	0	Podocyte
SPOCK2	0	3.79585594512041	0.418	0.032	0	Podocyte
PPFIA4	0	6.12682622240448	0.387	0.004	0	Podocyte
MYH9	0	1.93035620245672	0.695	0.313	0	Podocyte
GOLIM4	0	2.15504471449597	0.557	0.176	0	Podocyte
NEBL	0	3.13429453723096	0.81	0.431	0	Podocyte
TJP1	0	2.47107484243761	0.75	0.371	0	Podocyte
AL024495.1	0	5.38404532527107	0.393	0.015	0	Podocyte
CDC42EP3	0	3.37794703471115	0.416	0.039	0	Podocyte
ANKRD44	0	1.42467946162516	0.551	0.177	0	Podocyte
SYNJ2	0	2.91215861114731	0.429	0.055	0	Podocyte
ITGAV	0	2.03951263968042	0.68	0.309	0	Podocyte
COL4A3	0	2.70972087148298	0.774	0.405	0	Podocyte
MYO1D	0	1.9336062856079	0.695	0.328	0	Podocyte
TMCC3	0	2.42691596243382	0.455	0.089	0	Podocyte
SBSPON	0	3.57302351019908	0.398	0.033	0	Podocyte
MYO1E	0	2.08271809294828	0.763	0.399	0	Podocyte
ATP8B1	0	2.06242970743578	0.534	0.17	0	Podocyte
USP46	0	2.35181644512334	0.466	0.104	0	Podocyte

gene	p_val	avg_log2FC	pct.1	pct.2	p_val_adj	cluster
TUSC3	0	2.15491662393308	0.522	0.16	0	Podocyte
AC008011.2	0	8.09582928611611	0.363	0.001	0	Podocyte
ITIH5	0	2.66913878929899	0.421	0.06	0	Podocyte
SORT1	0	1.81529403633832	0.55	0.19	0	Podocyte
ADAMTS9	0	2.12635690515498	0.685	0.328	0	Podocyte
NES	0	3.80893464042694	0.378	0.021	0	Podocyte
PARVA	0	1.88033910170801	0.592	0.235	0	Podocyte
COL4A4	0	1.97178151067412	0.697	0.341	0	Podocyte
TMEM150C	0	2.73266154280736	0.435	0.08	0	Podocyte
XKR4	0	3.78834510247919	0.385	0.03	0	Podocyte
UACA	0	1.95107662598755	0.739	0.384	0	Podocyte
PDE4DIP	0	1.97628914441167	0.717	0.363	0	Podocyte
ITGA3	0	1.73248154003924	0.489	0.137	0	Podocyte
CEP85L	0	1.87301244183702	0.507	0.156	0	Podocyte
JAZF1	0	1.75189700914182	0.494	0.143	0	Podocyte
CBLB	0	2.73058090842267	0.745	0.397	0	Podocyte
TRAM2	0	3.12846814155158	0.387	0.04	0	Podocyte
TNNT2	0	3.95866788242067	0.362	0.018	0	Podocyte
AL365295.1	0	2.6039521480264	0.431	0.087	0	Podocyte
IGFBP2	0	3.19540635037536	0.377	0.034	0	Podocyte
AL022068.1	0	2.4547829200634	0.426	0.084	0	Podocyte
MCC	0	1.63810688780333	0.534	0.195	0	Podocyte
ITGB3	0	2.23971327241939	0.402	0.063	0	Podocyte
APBA1	0	2.62766647027013	0.391	0.054	0	Podocyte
SMAD6	0	2.1925034228051	0.413	0.076	0	Podocyte
LARGE1	0	2.36278563736055	0.739	0.405	0	Podocyte
NOTCH2	0	1.83360699661305	0.473	0.14	0	Podocyte
CPNE8	0	1.8519856879465	0.624	0.292	0	Podocyte
PHF21B	0	1.69750972365022	0.456	0.124	0	Podocyte
SPATA13	0	2.72670526736989	0.762	0.431	0	Podocyte
INF2	0	2.65044530439662	0.381	0.05	0	Podocyte
DST	0	1.86129341845741	0.812	0.482	0	Podocyte
ARHGEF3	0	2.04532762428318	0.714	0.384	0	Podocyte
BCAM	0	2.08273475844106	0.472	0.142	0	Podocyte
CCDC91	0	2.93681568031458	0.741	0.413	0	Podocyte
MAP1B	0	3.40717450151565	0.354	0.026	0	Podocyte
CRIM1	0	2.13148823909574	0.736	0.409	0	Podocyte
F5	0	4.25981712549762	0.338	0.012	0	Podocyte
PDLIM2	0	3.38929372941609	0.348	0.022	0	Podocyte
MFSD6	0	2.00095248012724	0.426	0.101	0	Podocyte
SYNPO	0	3.21337023115951	0.356	0.031	0	Podocyte
GRK3	0	2.18929398778561	0.42	0.097	0	Podocyte

gene	p_val	avg_log2FC	pct.1	pct.2	p_val_adj	cluster
ZNRF3	0	1.76414825287078	0.664	0.342	0	Podocyte
CCN2	0	2.785774189	0.396	0.079	0	Podocyte
SOX2-OT	0	2.94985633561173	0.441	0.126	0	Podocyte
NXN	0	1.75937483874699	0.399	0.084	0	Podocyte
ACTN4	0	1.47263383212273	0.733	0.418	0	Podocyte
TSC22D3	0	1.44138716675692	0.452	0.137	0	Podocyte
F3	0	4.36731863275793	0.326	0.013	0	Podocyte
CTDSPL	0	1.93051528282717	0.712	0.402	0	Podocyte
TLE4	0	1.56594129214969	0.45	0.143	0	Podocyte
SERPINI2	0	6.75325385059447	0.31	0.003	0	Podocyte
PDGFRB	0	3.0313062117562	0.324	0.018	0	Podocyte
FLRT3	0	2.13517367632265	0.379	0.073	0	Podocyte
PDLIM5	0	1.7548017902732	0.703	0.397	0	Podocyte
SNCA	0	3.96214919608108	0.324	0.019	0	Podocyte
AC112722.1	0	3.07104295414725	0.346	0.042	0	Podocyte
PTGER4	0	4.52973104998035	0.312	0.008	0	Podocyte
ADORA1	0	5.27231692025398	0.31	0.007	0	Podocyte
VIM	0	2.51968047408146	0.349	0.046	0	Podocyte
CDKN1C	0	2.98601790691211	0.344	0.044	0	Podocyte
TNS2	0	1.57807411401417	0.401	0.102	0	Podocyte
PRKAR2B	0	4.22515893550211	0.312	0.013	0	Podocyte
LHFPL6	0	1.64099562342873	0.368	0.072	0	Podocyte
NLK	0	1.80200134184091	0.687	0.392	0	Podocyte
TMEM178A	0	1.61350713336596	0.397	0.104	0	Podocyte
MTSS1	0	1.93925476003723	0.734	0.444	0	Podocyte
IQSEC2	0	2.09551656894461	0.366	0.077	0	Podocyte
DCN	0	3.37590033468557	0.309	0.022	0	Podocyte
SLC7A14-AS1	0	4.86051348625459	0.302	0.016	0	Podocyte
ZNF521	0	1.62993851210042	0.363	0.078	0	Podocyte
CD47	0	1.752859929	0.397	0.113	0	Podocyte
HS3ST3A1	0	3.81109275270633	0.295	0.011	0	Podocyte
ARRB1	0	1.98919498714495	0.35	0.067	0	Podocyte
PRKG1	0	1.2331008504882	0.814	0.531	0	Podocyte
FAM89A	0	4.39331914390048	0.291	0.009	0	Podocyte
ADAMTS19-AS1	0	7.63654849074579	0.282	0.001	0	Podocyte
ARHGAP19	0	2.75576377987509	0.314	0.035	0	Podocyte
ILDR2	0	2.55003623383207	0.319	0.042	0	Podocyte
LNX2	0	1.90269919167605	0.397	0.12	0	Podocyte
FAM81A	0	2.1483943294571	0.366	0.089	0	Podocyte
MAFB	0	4.36095064860627	0.288	0.011	0	Podocyte
TCF21	0	5.1737110491815	0.281	0.005	0	Podocyte
TBXAS1	0	1.8136069023156	0.341	0.065	0	Podocyte

gene	p_val	avg_log2FC	pct.1	pct.2	p_val_adj	cluster
HPGD	0	2.99367505845704	0.326	0.053	0	Podocyte
MCTP2	0	1.63706300413427	0.367	0.095	0	Podocyte
LGALS8	0	2.27115538702455	0.332	0.063	0	Podocyte
TRIB2	0	2.40433783011072	0.318	0.051	0	Podocyte
JAKMIP2	0	3.81000597040079	0.278	0.011	0	Podocyte
MAP3K20	0	2.28318924872705	0.315	0.05	0	Podocyte
TMEM241	0	1.60658652073341	0.358	0.093	0	Podocyte
TSHZ3	0	3.03306653992709	0.289	0.024	0	Podocyte
DPYSL2	0	1.81455166844752	0.333	0.07	0	Podocyte
SPTB	0	4.16280289396848	0.274	0.012	0	Podocyte
MXRA8	0	4.25976356451748	0.271	0.01	0	Podocyte
SPSB1	0	1.37798336211344	0.348	0.087	0	Podocyte
AFAP1L2	0	1.99185578582589	0.324	0.063	0	Podocyte
GPSM1	0	3.30703186375096	0.285	0.024	0	Podocyte
FAM126A	0	1.99705770020267	0.312	0.058	0	Podocyte
FLRT2	0	2.33668887878984	0.278	0.025	0	Podocyte
SH3BGRL2	0	2.06422585295098	0.33	0.078	0	Podocyte
TTLL7	0	1.99170875398925	0.304	0.053	0	Podocyte
ARHGEF12	0	1.68219456573376	0.743	0.493	0	Podocyte
KCND3	0	2.38966075965811	0.291	0.042	0	Podocyte
EPB41L5	0	1.52284107288391	0.753	0.505	0	Podocyte
IL1RAP	0	2.85112675888619	0.282	0.034	0	Podocyte
AC124852.1	0	2.64946257136316	0.279	0.033	0	Podocyte
FOXC1	0	3.23711551905871	0.266	0.023	0	Podocyte
SPATA18	0	3.08571213497191	0.265	0.026	0	Podocyte
ARHGAP23	0	2.86781826679969	0.26	0.022	0	Podocyte
MYL9	0	2.45096562674152	0.279	0.041	0	Podocyte
AC018742.1	0	3.55656203137607	0.262	0.025	0	Podocyte
SPARC	0	2.22919950628412	0.277	0.04	0	Podocyte
VTI1A	0	2.6932421303952	0.763	0.528	0	Podocyte
MYL3	0	1.84898076791207	0.298	0.066	0	Podocyte
ZSWIM6	0	1.43254633849571	0.785	0.553	0	Podocyte
LIMS2	0	1.50097429725521	0.281	0.05	0	Podocyte
CCDC85C	0	2.10557070103039	0.287	0.058	0	Podocyte
SYT9	0	2.0698089274006	0.272	0.044	0	Podocyte
ARHGEF37	0	1.88859392310852	0.279	0.052	0	Podocyte
PAX8-AS1	0	2.48127344455833	0.269	0.047	0	Podocyte
THSD7A	0	1.59726864361707	0.81	0.6	0	Podocyte
MAGI2	0	5.1619565794873	0.868	0.675	0	Podocyte
PARD3B	0	2.34615731384524	0.834	0.682	0	Podocyte
GPC6	1.18246019777435e-307	1.72808918316366	0.709	0.398	3.41967489196342e-303	Podocyte
ZBTB46	5.12249906138605e-306	1.15067748589712	0.462	0.146	1.48142672855285e-301	Podocyte

gene	p_val	avg_log2FC	pct.1	pct.2	p_val_adj	cluster
FRMD3	3.46880405522321e-302	1.41652175722767	0.694	0.358	1.00317813277055e-297	Podocyte
GULP1	2.38280960686303e-298	1.50921505941386	0.686	0.374	6.89108538304788e-294	Podocyte
CHD3	1.73102807531493e-293	1.92171572391241	0.34	0.092	5.00613319381078e-289	Podocyte
TANC1	5.71125581671556e-293	1.67266039605427	0.654	0.359	1.65169518219414e-288	Podocyte
CHN1	1.10586484427911e-290	1.44288343099629	0.254	0.054	3.19816112965519e-286	Podocyte
PALLD	5.59623673804582e-290	1.27101140194166	0.576	0.232	1.61843166464285e-285	Podocyte
CAPN2	1.01645552148643e-287	1.45031253118626	0.588	0.251	2.93958936813877e-283	Podocyte
PLEKHG1	7.64601010465467e-286	1.12275792562838	0.539	0.202	2.21122612226613e-281	Podocyte
KCNT2	1.67148487245493e-285	1.25595652727425	0.262	0.058	4.83393425113964e-281	Podocyte
MIB1	5.69570136922456e-285	1.60043754502339	0.618	0.303	1.64719683597974e-280	Podocyte
SEPTIN11	1.72315799663025e-284	1.57364262464752	0.598	0.27	4.98337292625469e-280	Podocyte
LEPR	1.72634663106211e-284	1.74560366059543	0.391	0.122	4.99259445703161e-280	Podocyte
DMD	7.43927634609647e-283	1.76755425846087	0.406	0.133	2.1514387192911e-278	Podocyte
XYLT1	8.92639594606527e-280	1.32801114490592	0.273	0.063	2.58151370760208e-275	Podocyte
MDFIC	9.21159721330554e-279	1.41313319694425	0.434	0.144	2.66399391408796e-274	Podocyte
MAML3	7.93881359222338e-275	1.47658399720388	0.677	0.369	2.295904890871e-270	Podocyte
GSN	7.6064327463453e-274	1.38587735956765	0.465	0.164	2.19978035024306e-269	Podocyte
CNTN1	2.89958495412803e-273	1.85545794570543	0.283	0.07	8.38559968733827e-269	Podocyte
PARD3	4.05150552782748e-273	1.14055567391008	0.825	0.724	1.17169539864771e-268	Podocyte
TET2	6.11651249422231e-272	1.51964298695427	0.652	0.35	1.76889541332909e-267	Podocyte
TRAF5	2.73768084653975e-269	1.55472960347514	0.302	0.079	7.91737300819296e-265	Podocyte
DHRS3	2.78424794694441e-268	1.25779284427875	0.553	0.225	8.05204506256324e-264	Podocyte
TNNI3K	7.58327361786564e-267	1.69209434179694	0.255	0.059	2.19308273028674e-262	Podocyte
GMDS	4.94929145534036e-265	1.5282676453249	0.671	0.378	1.43133508888443e-260	Podocyte
EFCAB13	1.05611437949026e-261	1.59225439076842	0.511	0.206	3.05428278548584e-257	Podocyte
EIF3M	4.56524444316761e-260	1.52160856668584	0.339	0.099	1.32026869296407e-255	Podocyte
NPR3	3.91940459926539e-258	1.28397439897148	0.455	0.163	1.13349181010755e-253	Podocyte
HFM1	3.4198401696994e-256	1.97500615177445	0.259	0.063	9.89017777077068e-252	Podocyte
PTPN13	4.86900875202619e-251	1.28993605449872	0.755	0.508	1.40811733108597e-246	Podocyte
SLK	1.03970388387826e-250	1.38060520689317	0.556	0.246	3.00682363217593e-246	Podocyte
AC245297.3	1.95579511148461e-250	1.37948050591181	0.639	0.332	5.65615946241348e-246	Podocyte
AC098829.1	9.21459276260634e-249	1.40549751587738	0.414	0.143	2.66486022694575e-244	Podocyte
TNIK	2.84629952753275e-248	1.25214605838075	0.508	0.199	8.23149823362471e-244	Podocyte
WASL	6.66747318314999e-248	1.55807746656019	0.519	0.225	1.92823324456698e-243	Podocyte
STN1	2.33396975896723e-247	1.65853857700331	0.26	0.065	6.74984054293322e-243	Podocyte
NBPF14	1.1268454118826e-246	1.2584436247354	0.321	0.091	3.25883693116448e-242	Podocyte
GTDC1	2.46782976225382e-245	1.53765975686192	0.504	0.21	7.13696367243804e-241	Podocyte
SLC44A2	3.12716971842612e-245	1.41317859626103	0.329	0.096	9.04377482568835e-241	Podocyte
RIPOR2	1.22868260728844e-242	0.403260222268572	0.259	0.062	3.55335010027816e-238	Podocyte
ZEB2	2.73646666422531e-238	0.897877896190496	0.406	0.134	7.9138615929396e-234	Podocyte
LPCAT2	6.83440921657473e-236	1.4271440725368	0.326	0.098	1.97651114543341e-231	Podocyte
SLC6A6	6.85215857088888e-235	1.0918954576498	0.319	0.092	1.98164425870107e-230	Podocyte

gene	p_val	avg_log2FC	pct.1	pct.2	p_val_adj	cluster
MTSS2	5.26508684205132e-234	1.6337591331202	0.251	0.063	1.52266311472124e-229	Podocyte
BACH1	3.86731872099462e-233	1.52257224410596	0.637	0.359	1.11842857411164e-228	Podocyte
BCAR3	4.50086864197151e-233	1.23741517541034	0.526	0.221	1.30165121125816e-228	Podocyte
NAPEPLD	5.15638570985773e-232	1.36675248471947	0.443	0.167	1.49122674729085e-227	Podocyte
TOM1L2	9.32211251483603e-232	1.45271726727947	0.64	0.358	2.69595493929058e-227	Podocyte
RBMS1	1.21802269161549e-231	1.31684275755564	0.692	0.453	3.52252162415201e-227	Podocyte
TET3	3.15704050717877e-231	1.38559222358499	0.384	0.13	9.130161146761e-227	Podocyte
UNC5D	1.85486663930504e-229	2.39258968819448	0.303	0.092	5.36427432087016e-225	Podocyte
DPY19L3	3.07356160713841e-229	1.49177503528456	0.382	0.131	8.88874016784429e-225	Podocyte
APBB2	6.05823220612867e-229	1.19914010130572	0.757	0.549	1.75204075401241e-224	Podocyte
PHACTR4	1.28395565833997e-226	1.26280280454876	0.677	0.395	3.7131997639192e-222	Podocyte
RNF145	1.22954018007424e-225	1.26295241639373	0.398	0.139	3.5558302007747e-221	Podocyte
CMYA5	1.85900360507307e-221	1.02489183972941	0.693	0.374	5.37623842587132e-217	Podocyte
COL4A5	1.89202466863688e-221	1.05790449608829	0.605	0.283	5.47173534169787e-217	Podocyte
TMCO3	1.27257896909689e-220	1.45591803304339	0.278	0.078	3.68029837862822e-216	Podocyte
PRKCI	1.63305271000262e-217	1.27616181012197	0.514	0.224	4.72278843732759e-213	Podocyte
AL589935.1	2.63192927224802e-215	1.52505185115218	0.573	0.291	7.61153945534129e-211	Podocyte
PAPSS1	1.87994281191953e-212	1.36144044366689	0.371	0.129	5.43679461207128e-208	Podocyte
ZNF496	2.35202899592399e-209	1.54891066183954	0.25	0.067	6.80206785621217e-205	Podocyte
TMSB4X	2.2511789517526e-207	1.45298807145266	0.29	0.085	6.51040952846853e-203	Podocyte
CASZ1	2.59159590807462e-207	1.19944339778324	0.558	0.258	7.4948953661518e-203	Podocyte
SSBP2	3.21824250566981e-207	1.13580916324901	0.633	0.332	9.30715732639708e-203	Podocyte
AKT3	1.35925358090631e-203	1.00349249603092	0.703	0.43	3.93096135598104e-199	Podocyte
PTPN14	3.3855368840109e-203	1.23796656840135	0.641	0.368	9.79097266855952e-199	Podocyte
NEDD9	1.92938985923049e-202	1.06644004067267	0.566	0.268	5.57979547289459e-198	Podocyte
SULF2	9.13983523542595e-198	1.23285600217525	0.417	0.159	2.64324035008519e-193	Podocyte
PLEKHH2	9.18963445488031e-197	1.11485038326794	0.467	0.192	2.65764228435139e-192	Podocyte
HPS5	1.19512720605378e-196	1.44737367563662	0.267	0.079	3.45630787990753e-192	Podocyte
RASSF8	3.07875300223835e-196	1.29958347435644	0.613	0.339	8.9037536824733e-192	Podocyte
CTNNBIP1	3.80737544401668e-193	1.3128983431744	0.319	0.105	1.10109297840962e-188	Podocyte
DOCK4	4.18146321554543e-191	0.814872843233543	0.638	0.339	1.20927916193574e-186	Podocyte
DTNA	1.6359481219227e-189	1.22332464670118	0.312	0.102	4.73116196860046e-185	Podocyte
PCMTD2	2.63541863510087e-188	1.2010370121624	0.415	0.163	7.62163069271172e-184	Podocyte
FAT1	3.62018660273543e-187	0.949491259644598	0.641	0.347	1.04695796551109e-182	Podocyte
LINC00472	5.39320669247985e-187	0.770662089458505	0.873	0.794	1.55971537546517e-182	Podocyte
PPP2R2B	2.09387992706075e-186	0.987626938102442	0.477	0.202	6.05550074905969e-182	Podocyte
KCNQ1OT1	1.63496927509555e-184	1.07400782119381	0.755	0.527	4.72833114357632e-180	Podocyte
LRP11	1.4375988738926e-181	1.35492724661332	0.281	0.089	4.15753594329741e-177	Podocyte
PTBP3	3.68950627167873e-181	1.00037494308321	0.629	0.346	1.06700521376949e-176	Podocyte
UTRN	3.6708192867787e-179	0.825635903772222	0.73	0.492	1.0616009377364e-174	Podocyte
DLG2	9.37291640336657e-175	1.00463559904179	0.815	0.614	2.71064742385361e-170	Podocyte
NUDCD1	6.48528858945545e-172	1.38496011544322	0.253	0.077	1.87554546007052e-167	Podocyte

gene	p_val	avg_log2FC	pct.1	pct.2	p_val_adj	cluster
ARL13B	2.44943333234265e-171	1.25299331292106	0.322	0.114	7.08376119713493e-167	Podocyte
SDC2	2.35674357837457e-170	1.13553378827239	0.329	0.118	6.81570242865925e-166	Podocyte
PAWR	9.7546016562633e-170	1.13955856845927	0.597	0.333	2.82103079899135e-165	Podocyte
FNBP1	5.13831384700829e-168	0.915203145060828	0.561	0.28	1.4860003645548e-163	Podocyte
AL139383.1	1.68748952772386e-167	1.13796757463088	0.393	0.157	4.8802197141774e-163	Podocyte
ITGA1	4.9112548292863e-167	0.957499352946442	0.474	0.214	1.4203348966296e-162	Podocyte
IQSEC1	1.5416344988337e-164	1.08597812900805	0.407	0.166	4.45840697062707e-160	Podocyte
CALM2	3.37483173444204e-163	1.14423586433895	0.404	0.168	9.76001337600637e-159	Podocyte
ST3GAL1	2.02180561169022e-162	0.900149159703385	0.446	0.189	5.84706182900811e-158	Podocyte
PTPN21	2.63262345223976e-162	1.09900049317423	0.422	0.18	7.6135470238774e-158	Podocyte
ZNF462	2.72807245940805e-162	0.945309819199925	0.497	0.229	7.88958555260808e-158	Podocyte
ZNF124	1.47095669278487e-161	1.14808202594793	0.332	0.123	4.25400675553384e-157	Podocyte
TXNRD1	1.93946729219524e-160	1.18720391849066	0.35	0.134	5.60893940902864e-156	Podocyte
NECTIN2	4.78519607887453e-160	1.1341103770511	0.416	0.174	1.38387870601051e-155	Podocyte
ZC2HC1A	6.37418398064473e-160	1.2265143311304	0.295	0.103	1.84341400720246e-155	Podocyte
TMOD3	1.57664168005661e-159	1.11762394421242	0.489	0.235	4.55964773872371e-155	Podocyte
SLC7A8	6.05841332158393e-159	0.753443587289972	0.516	0.24	1.75209313260207e-154	Podocyte
KATNAL1	1.62252980798882e-157	1.15774970094116	0.263	0.085	4.69235620470365e-153	Podocyte
DCBLD2	5.20410443213831e-156	0.865143897998636	0.47	0.213	1.5050270017744e-151	Podocyte
KANK1	1.10235667961002e-155	0.888206745349013	0.765	0.606	3.18801551743218e-151	Podocyte
LAMA5	2.41196378220317e-154	1.01992005785347	0.406	0.168	6.97539925813157e-150	Podocyte
SPTAN1	7.98746462521379e-153	0.916945699057635	0.546	0.274	2.30997476961183e-148	Podocyte
EML4	3.39430630052667e-152	0.891595193980868	0.665	0.42	9.81633382112312e-148	Podocyte
STAT1	8.10205404064336e-152	1.32441384910148	0.302	0.112	2.34311402855406e-147	Podocyte
LRRC1	4.46416249053558e-150	1.08240122876762	0.334	0.129	1.29103579226289e-145	Podocyte
HMGN1	2.58007535340155e-149	1.28832764730763	0.283	0.1	7.46157792203728e-145	Podocyte
REPS1	3.26004136678269e-149	1.04091020617779	0.52	0.272	9.42803963273552e-145	Podocyte
SLC25A37	5.34479256427324e-146	0.982488564809366	0.468	0.218	1.54571400958782e-141	Podocyte
AHNAK	1.87699137519758e-145	0.892611376119855	0.486	0.229	5.42825905707141e-141	Podocyte
NCK2	8.78391813828627e-145	0.928390697079183	0.369	0.154	2.54030912559239e-140	Podocyte
ABI1	1.26369259923588e-143	1.20612433291536	0.508	0.275	3.65459899699017e-139	Podocyte
ZFYVE9	3.8504793877232e-143	1.02588690454667	0.524	0.274	1.11355863892955e-138	Podocyte
PLCG2	2.32124168631363e-141	0.699073825447658	0.577	0.316	6.713030956819e-137	Podocyte
FNDC3B	8.55884408765957e-141	0.791859353934365	0.767	0.624	2.47521771015115e-136	Podocyte
CDS1	1.38604593128393e-140	1.44231080793375	0.308	0.121	4.00844483327313e-136	Podocyte
TSHZ1	1.21035306782374e-139	1.13370011639339	0.355	0.147	3.50034107214626e-135	Podocyte
BICD1	3.80442018672208e-139	0.803893883297822	0.564	0.302	1.10023831800003e-134	Podocyte
ZSWIM5	2.39313397770148e-138	0.995029888074511	0.438	0.205	6.92094346351268e-134	Podocyte
ARHGEF10	1.67133009090695e-137	0.833147977487402	0.354	0.143	4.83348662290291e-133	Podocyte
DLC1	3.57948231496388e-136	0.455218182177661	0.311	0.116	1.03518628548756e-131	Podocyte
PABPN1	8.24803311585785e-136	1.03371244888957	0.364	0.155	2.38533117710609e-131	Podocyte
FANCL	2.10111504843531e-130	0.950722061375703	0.355	0.151	6.07642472007491e-126	Podocyte

gene	p_val	avg_log2FC	pct.1	pct.2	p_val_adj	cluster
FCHSD2	2.24307824063665e-130	0.768858415921253	0.605	0.361	6.48698227192118e-126	Podocyte
GHR	3.96468526568466e-129	0.872163542258738	0.603	0.353	1.146586978836e-124	Podocyte
ITM2B	7.86570560514293e-129	1.18797770565028	0.557	0.324	2.27476206100734e-124	Podocyte
MAL2	7.03168114077292e-127	0.923118418855316	0.339	0.142	2.03356218591153e-122	Podocyte
MT-ND6	9.77006064574716e-127	2.52320835462973	0.376	0.175	2.82550153875008e-122	Podocyte
TRPC1	7.46246534167741e-126	0.70124150050195	0.383	0.167	2.15814497681311e-121	Podocyte
KIF13A	1.25418012384556e-124	0.862770780175096	0.553	0.309	3.62708891816135e-120	Podocyte
MACF1	1.36784911858997e-124	0.632532539587095	0.831	0.733	3.95581965096219e-120	Podocyte
HSPA12A	1.54887968602664e-124	0.79413421861919	0.542	0.294	4.47936005198904e-120	Podocyte
GAB2	1.47556076832844e-123	0.960716138844516	0.357	0.156	4.26732174200584e-119	Podocyte
ERC2	3.26213998875307e-123	1.09212918510294	0.327	0.137	9.43410884747387e-119	Podocyte
HMGN3	2.51492518718107e-122	0.924173371877639	0.485	0.252	7.27316364132767e-118	Podocyte
HNRNPLL	2.70460755399635e-122	0.986917494174023	0.325	0.136	7.82172504615745e-118	Podocyte
FBXO21	8.63143342537973e-121	0.596845925775101	0.604	0.342	2.49621054661982e-116	Podocyte
EVL	4.40894397831964e-120	0.861032569963164	0.393	0.182	1.27506659853004e-115	Podocyte
AASS	8.96660532952044e-118	0.78463544661218	0.531	0.287	2.59314226129731e-113	Podocyte
PLOD2	5.10264968044658e-117	0.991956872117437	0.373	0.172	1.47568628758515e-112	Podocyte
FMNL2	2.94970530097113e-116	0.852856273015956	0.614	0.383	8.5305477304085e-112	Podocyte
ANKS1B	7.48263306323523e-116	0.460482279899796	0.463	0.224	2.16397748188763e-111	Podocyte
DCLK2	6.60279651182326e-115	0.726264086332093	0.335	0.142	1.90952875121929e-110	Podocyte
CEMIP2	2.46962009210222e-113	0.84692030651946	0.403	0.196	7.14214130635962e-109	Podocyte
SH3RF1	8.00096992347059e-113	0.77003235358194	0.694	0.499	2.31388050186769e-108	Podocyte
RAD23B	1.3135440020209e-112	0.959002971586825	0.427	0.214	3.79876925384444e-108	Podocyte
PDE3A	1.22555596355432e-111	0.639084915775805	0.377	0.175	3.5443078465991e-107	Podocyte
QKI	3.49007372058033e-111	0.788810857816306	0.682	0.49	1.00932931999183e-106	Podocyte
DOCK3	4.24743316623363e-110	0.809458637618023	0.323	0.138	1.22835767167476e-105	Podocyte
ATP10D	2.81301220674712e-109	0.941278322738839	0.444	0.231	8.13523130191267e-105	Podocyte
LIMK2	5.67038794450034e-109	0.807667909443559	0.274	0.108	1.6398761935495e-104	Podocyte
CAST	4.92033561374036e-107	0.771681074522474	0.564	0.343	1.42296105949371e-102	Podocyte
DGKD	1.01323047431529e-106	0.83235807563787	0.274	0.111	2.93026253171983e-102	Podocyte
RBPMS	2.72040402702496e-106	0.535114645083649	0.81	0.687	7.86740844615617e-102	Podocyte
ZNF609	6.04386296164844e-106	0.759524937080136	0.63	0.413	1.74788516850873e-101	Podocyte
WASHC1	9.67844004721046e-106	0.72655911583293	0.621	0.385	2.79900486165327e-101	Podocyte
FERMT2	5.47862459789503e-105	0.773866046156006	0.574	0.349	1.58441823371124e-100	Podocyte
RASSF8-AS1	1.93288695422122e-104	0.694998402496125	0.366	0.168	5.58990907160776e-100	Podocyte
WDR26	5.15414538595657e-104	0.824059091693637	0.467	0.251	1.49057884561864e-99	Podocyte
ARHGAP29	1.69067439312241e-103	0.611891921610062	0.635	0.403	4.88943034491e-99	Podocyte
WWP1	2.84615943810917e-103	0.74788162018041	0.621	0.403	8.23109309501173e-99	Podocyte
SH3BP5	3.82915499368354e-103	0.472606890185355	0.292	0.118	1.10739162417328e-98	Podocyte
AL359232.1	4.07195424464832e-103	0.883670000619398	0.39	0.191	1.17760916755229e-98	Podocyte
CAND1	4.18292411095064e-103	0.80535605341477	0.394	0.194	1.20970165288692e-98	Podocyte
CSPP1	4.3093581310625e-103	0.830369862844037	0.571	0.358	1.24626637150327e-98	Podocyte

gene	p_val	avg_log2FC	pct.1	pct.2	p_val_adj	cluster
ZBTB1	6.3494048200412e-103	0.7680722889799	0.412	0.206	1.83624787395592e-98	Podocyte
RDX	2.34969558193268e-102	0.523672022518662	0.546	0.317	6.79531962294931e-98	Podocyte
SLC16A10	4.79901000686514e-102	0.858694842089405	0.476	0.259	1.3878736939854e-97	Podocyte
DAPK1	7.64472739428497e-102	0.702392211412005	0.606	0.381	2.21085516242721e-97	Podocyte
ADD3	8.00376703808126e-102	0.759788208833632	0.617	0.41	2.3146894274131e-97	Podocyte
AKT2	1.1075920748664e-101	0.823364552212116	0.329	0.148	3.20315628051364e-97	Podocyte
PDE7B	2.1195148803951e-101	0.824195223204073	0.666	0.444	6.12963703410264e-97	Podocyte
CPEB4	3.04244243388117e-100	0.5194008205244	0.712	0.507	8.79874351878435e-96	Podocyte
CEP112	3.33574801433946e-100	0.664303600004366	0.551	0.324	9.64698325746972e-96	Podocyte
PAM	5.37225948488093e-100	0.725774263357843	0.629	0.419	1.55365744302756e-95	Podocyte
CCDC6	2.43702293789227e-99	0.841214797539841	0.392	0.197	7.04787033638444e-95	Podocyte
PON2	8.73313901890775e-99	0.745060688173625	0.342	0.159	2.52562380426812e-94	Podocyte
SOBP	1.0604923222098e-98	0.766529702223371	0.36	0.169	3.06694379583073e-94	Podocyte
CLEC16A	1.62682253544018e-98	0.811206595936084	0.575	0.367	4.70477077249299e-94	Podocyte
PICALM	1.56998282493849e-97	0.665539188157344	0.717	0.574	4.5403903297221e-93	Podocyte
AL591519.1	3.35798948293739e-94	0.682573126552548	0.286	0.123	9.71130558465494e-90	Podocyte
CAMSAP2	4.79374550796794e-94	0.708337319674949	0.503	0.285	1.38635120090433e-89	Podocyte
CECR2	6.11663159398343e-94	0.779261583969181	0.435	0.23	1.76892985698001e-89	Podocyte
PAN3	2.7112391468581e-93	0.715526056743885	0.695	0.529	7.84090361271361e-89	Podocyte
MED27	6.17689259856405e-93	0.880534492966112	0.441	0.243	1.78635733950472e-88	Podocyte
RFC1	1.36951391647886e-92	0.693384376359801	0.402	0.205	3.96063424645688e-88	Podocyte
GLS	1.62853090428567e-92	0.534543121478809	0.724	0.561	4.70971137519416e-88	Podocyte
SENP7	7.7027200763738e-92	0.644361791298748	0.532	0.314	2.2276266460873e-87	Podocyte
RABGAP1L	3.04844679153617e-91	0.656281272496187	0.802	0.732	8.81610812112261e-87	Podocyte
ANKH	3.37173387291094e-91	0.912838145998538	0.32	0.15	9.75105436045844e-87	Podocyte
PLCB4	7.63157224332518e-91	0.540507176520862	0.391	0.195	2.20705069276964e-86	Podocyte
SBF2	9.17583578152886e-91	0.575753772550902	0.784	0.678	2.65365170801815e-86	Podocyte
RHOQ	3.74778712284736e-90	0.850387581644909	0.276	0.12	1.08386003592746e-85	Podocyte
PCMTD1	5.57454132145514e-90	0.851431755534416	0.575	0.381	1.61215735016483e-85	Podocyte
FSD1L	1.55772058263033e-89	0.691555349047175	0.368	0.182	4.50492792496692e-85	Podocyte
PWWP3A	1.64581937653897e-89	0.721253521209752	0.309	0.141	4.75970963695071e-85	Podocyte
RAPGEF3	3.51574778415063e-89	0.309242037076356	0.477	0.247	1.01675425917636e-84	Podocyte
SEMA3B	9.3823541865373e-89	0.567487903028475	0.33	0.152	2.71337683074659e-84	Podocyte
VEZT	2.26437473555532e-88	0.734628877824199	0.485	0.277	6.54857173522598e-84	Podocyte
BNIP3L	5.31442564341212e-88	0.300286956490392	0.498	0.266	1.53693189607479e-83	Podocyte
ZNF736	5.38511623503378e-88	0.80838406083729	0.323	0.154	1.55737561517177e-83	Podocyte
RAPGEF6	7.33279601910666e-88	0.763573204921161	0.522	0.317	2.12064460872565e-83	Podocyte
TSEN15	7.00575958332503e-87	0.907501658516667	0.285	0.13	2.0260656714976e-82	Podocyte
TSPAN5	9.49044305329436e-87	0.561258283853226	0.319	0.148	2.74463613101273e-82	Podocyte
TNS3	1.2379994723192e-86	0.670315392482686	0.714	0.537	3.58029447394713e-82	Podocyte
TEAD1	2.19334714467565e-86	0.683248090755115	0.696	0.533	6.34315994240197e-82	Podocyte
AP3S1	4.70814101270239e-85	0.867501668275048	0.304	0.142	1.36159438087353e-80	Podocyte

gene	p_val	avg_log2FC	pct.1	pct.2	p_val_adj	cluster
TERF2IP	1.56396601270611e-84	0.802840637041558	0.368	0.189	4.52298970874608e-80	Podocyte
MYO1C	2.60243036506553e-84	0.963539763485789	0.256	0.112	7.52622861576951e-80	Podocyte
WDR41	2.72892930100967e-84	0.865070619347833	0.335	0.167	7.89206353851997e-80	Podocyte
MBNL3	1.3187057219401e-83	0.819048327550891	0.258	0.113	3.81369694785078e-79	Podocyte
MBD2	1.33651833052394e-83	0.815633654896802	0.385	0.202	3.86521101187522e-79	Podocyte
AC079781.5	2.91909798561413e-83	0.625150242437258	0.397	0.208	8.44203137439607e-79	Podocyte
DAG1	3.15332430365381e-83	0.933351240099855	0.301	0.143	9.11941388616682e-79	Podocyte
RAI14	6.14730950664836e-83	0.546405934199915	0.6	0.384	1.77780190932271e-78	Podocyte
DLG5	7.26608675844919e-83	0.82833919010334	0.352	0.178	2.1013522905435e-78	Podocyte
WBP1L	8.23292597228299e-83	0.502500296222745	0.432	0.232	2.38096219118424e-78	Podocyte
CDK6	9.62650835889692e-83	0.707959333975446	0.492	0.294	2.78398621739299e-78	Podocyte
TSTD3	1.97634338851372e-82	0.791593005272845	0.343	0.172	5.71558507958167e-78	Podocyte
SPATS2L	3.76981318488779e-82	0.538840323711542	0.547	0.325	1.09022997306955e-77	Podocyte
SCD5	1.5713202965231 8e-81	0.467780844885277	0.413	0.215	4.54425829754504e-77	Podocyte
S100A6	4.38457690759042e-81	1.03029181887775	0.286	0.134	1.26801964167515e-76	Podocyte
NOS1AP	1.88337387246047e-80	0.396851477228933	0.562	0.337	5.44671723915568e-76	Podocyte
APPBP2	4.36432865253143e-80	0.606154169791246	0.516	0.31	1.26216384631209e-75	Podocyte
USO1	2.66572504790463e-79	0.683599304776894	0.453	0.256	7.70927683854018e-75	Podocyte
CHD7	1.27338121733523e-78	0.626098738364167	0.558	0.351	3.68261848053348e-74	Podocyte
MEGF9	1.53538779833523e-77	0.748345226732819	0.408	0.225	4.44034151278548e-73	Podocyte
UBAP2	1.93031978865405e-76	0.69448520007913	0.456	0.266	5.58248482878752e-72	Podocyte
MED4	3.84579495759739e-76	0.668261490907612	0.322	0.159	1.11220390173717e-71	Podocyte
ALDH7A1	6.00431751929297e-76	0.60014934335609	0.301	0.144	1.73644862657953e-71	Podocyte
ADAMTS9-AS2	1.04296109178136e-75	0.848952120992911	0.762	0.605	3.0162434774317e-71	Podocyte
PCNX4	2.46959333886474e-75	0.497159987796853	0.559	0.348	7.14206393599684e-71	Podocyte
LINC00894	2.48455387919401e-75	0.656837915916982	0.549	0.35	7.18532981862909e-71	Podocyte
AL158152.1	2.49293387836957e-75	0.779641119592955	0.361	0.191	7.2095647762448e-71	Podocyte
AC087564.1	2.96674344454366e-75	0.580955499143324	0.423	0.237	8.57982204162027e-71	Podocyte
ERBIN	4.18033250177381e-75	0.625992506950335	0.67	0.503	1.20895215951298e-70	Podocyte
ZNF277	2.29340556138131e-74	0.58727639907367	0.474	0.279	6.63252888351474e-70	Podocyte
STXBP3	5.17500925419639e-74	0.805692374797777	0.341	0.178	1.49661267631359e-69	Podocyte
ZNF846	6.33669392817484e-74	0.563092704839466	0.337	0.17	1.83257188402816e-69	Podocyte
ARHGAP21	1.38649775808084e-73	0.581005083758094	0.504	0.304	4.0097515163698e-69	Podocyte
TAF15	1.56688927464134e-73	0.688998141625486	0.504	0.312	4.53144378226276e-69	Podocyte
PLEKHA2	4.47172182357036e-73	0.484448843229833	0.367	0.189	1.29322195137655e-68	Podocyte
SEPTIN7	3.0483515142018e-72	0.607785826140539	0.448	0.26	8.81583257907161e-68	Podocyte
CDK5RAP2	3.63249623239603e-72	0.610481672990122	0.372	0.197	1.05051791040893e-67	Podocyte
GNPTAB	2.91792505429988e-71	0.697708929823621	0.313	0.158	8.43863925703524e-67	Podocyte
ZNF608	2.0916585731779e-70	0.592846425514808	0.442	0.253	6.04907659363048e-66	Podocyte
KAT7	2.33138506865356e-70	0.65256446309323	0.252	0.117	6.74236561854611e-66	Podocyte
HECTD2	1.45592794477213e-69	0.472729709046266	0.359	0.189	4.210543616281e-65	Podocyte
ITGB5	5.38433642627404e-69	0.590046673672303	0.441	0.254	1.55715009447845e-64	Podocyte

gene	p_val	avg_log2FC	pct.1	pct.2	p_val_adj	cluster
CCDC122	3.56762705826016e-68	0.785333420909572	0.252	0.119	1.03175774524884e-63	Podocyte
TRAPPC10	6.02089848853742e-68	0.62765791299046	0.482	0.294	1.74124384288502e-63	Podocyte
RBMS2	6.38608064055452e-68	0.336021926547708	0.353	0.184	1.84685452124837e-63	Podocyte
VGLL4	6.79432822297451e-68	0.550905556882042	0.591	0.399	1.96491972208423e-63	Podocyte
WWOX	9.94224554738105e-68	0.63118027712403	0.841	0.787	2.8752974123026e-63	Podocyte
PLS3	3.13877425135698e-66	0.597182299041903	0.4	0.225	9.07733513492438e-62	Podocyte
FAM228B	3.7414558041255e-66	0.469558070298478	0.304	0.152	1.08202901855309e-61	Podocyte
PRMT2	4.73842685619814e-66	0.48765236959044	0.386	0.212	1.3703530468125e-61	Podocyte
KAT2B	3.32476117245084e-65	0.59291948876693	0.331	0.176	9.61520931072783e-61	Podocyte
JAK2	6.06399827144581e-65	0.510083821002536	0.458	0.277	1.75370830010213e-60	Podocyte
ROCK1	6.97001315500485e-65	0.455320739731617	0.594	0.407	2.0157278044274e-60	Podocyte
SEM1	8.01754136162573e-65	0.774509891470869	0.336	0.182	2.31867296178216e-60	Podocyte
WASF2	2.40354750869089e-64	0.511381861399667	0.471	0.285	6.95105939513406e-60	Podocyte
ARHGAP24	3.44220547356294e-64	0.516785759677957	0.694	0.535	9.95485822954402e-60	Podocyte
FGFR1	3.44969339863569e-64	0.437113407858286	0.266	0.127	9.97651330885443e-60	Podocyte
MT-CO2	4.5322927237031e-64	0.869828153380787	0.612	0.458	1.31073905569494e-59	Podocyte
AC006504.5	7.09874619540048e-64	0.767896177150121	0.323	0.173	2.05295739970982e-59	Podocyte
HIVEP2	1.38716612149447e-63	0.556976268935988	0.55	0.366	4.01168442336202e-59	Podocyte
NFATC3	1.49814630407619e-63	0.558979062336167	0.501	0.311	4.33263911138835e-59	Podocyte
SASH1	2.09795504954347e-63	0.334487505632717	0.592	0.38	6.06728600327971e-59	Podocyte
NRIP1	2.37286363554943e-63	0.71301265455658	0.587	0.42	6.86232163400895e-59	Podocyte
NEAT1	3.59024792622945e-63	0.302795178989458	0.931	0.921	1.03829970026556e-58	Podocyte
ERC1	4.96013971953042e-63	0.515200851408477	0.711	0.569	1.4344724068882e-58	Podocyte
XKR6	3.2797315159325e-62	0.497069240641288	0.564	0.365	9.4849835440768e-58	Podocyte
CNIH3	5.04816427697291e-62	0.651005094840607	0.291	0.149	1.45992910890056e-57	Podocyte
EEA1	1.04615011973819e-61	0.517753788913889	0.486	0.3	3.02546614628284e-57	Podocyte
IGF1R	1.5406617127353e-61	0.483747648172275	0.771	0.651	4.45559367323048e-57	Podocyte
PPHLN1	3.47673926347793e-61	0.578041533143084	0.437	0.263	1.00547299499782e-56	Podocyte
MYOF	9.64345288296482e-61	0.52827983219575	0.373	0.208	2.78888657375342e-56	Podocyte
VCL	1.50648475880416e-60	0.31072743969405	0.435	0.253	4.35675392246162e-56	Podocyte
SMAD5	6.2722944480331e-60	0.553756429129384	0.349	0.194	1.81394755437117e-55	Podocyte
RERE	9.71011395205126e-60	0.437889728072216	0.803	0.735	2.80816495493322e-55	Podocyte
PARD6B	1.38409075605123e-59	0.472786775236044	0.416	0.242	4.00279046650017e-55	Podocyte
FAM13B	1.44558432077655e-59	0.503677299317651	0.519	0.335	4.18062985568578e-55	Podocyte
P3H2	2.46982876809852e-59	0.438271629098895	0.645	0.457	7.14274479734093e-55	Podocyte
LINC01376	6.36961716457512e-59	0.540025495530157	0.333	0.181	1.84209328399512e-54	Podocyte
FRS2	8.34389081079539e-59	0.581396188012614	0.459	0.289	2.41305322248203e-54	Podocyte
IARS	1.47772783748808e-58	0.537631412038218	0.354	0.199	4.27358890601553e-54	Podocyte
DSTN	1.58309020006696e-58	0.802301297703904	0.352	0.2	4.57829685859365e-54	Podocyte
MIR4435-2HG	3.42114107734333e-58	0.395569726616874	0.34	0.185	9.89393999567692e-54	Podocyte
RIC3	1.67362589257477e-57	0.460872335279508	0.292	0.153	4.84012608132624e-53	Podocyte
IP6K2	2.42685587686686e-57	0.592694730187855	0.351	0.196	7.01846719589895e-53	Podocyte

gene	p_val	avg_log2FC	pct.1	pct.2	p_val_adj	cluster
EPC1	8.54191848101474e-57	0.501238367720581	0.556	0.376	2.47032282470946e-52	Podocyte
LENG8	2.68278148235342e-56	0.501369979003573	0.45	0.275	7.7586040469661e-52	Podocyte
DPYD	2.71908612805001e-56	0.429381581515435	0.56	0.378	7.86359708232062e-52	Podocyte
CCDC88A	3.56385782754194e-56	0.406581341542618	0.274	0.139	1.03066768372513e-51	Podocyte
ACTR3	1.17270895861398e-55	0.543540082031117	0.417	0.249	3.39147430831163e-51	Podocyte
MYEF2	1.19494113926296e-55	0.526214951994036	0.333	0.183	3.45576977474848e-51	Podocyte
SOX6	1.35469920132952e-55	0.481114277011939	0.747	0.621	3.91779009024496e-51	Podocyte
INTS3	2.60990702461894e-55	0.553697985668931	0.316	0.171	7.54785111519797e-51	Podocyte
ELF1	3.21542947632792e-55	0.494262965305797	0.595	0.414	9.29902204554034e-51	Podocyte
MTHFS	5.67306519061762e-55	0.518109260385844	0.264	0.134	1.64065045312662e-50	Podocyte
SUSD6	1.38372260771633e-54	0.481995254205162	0.404	0.242	4.00172578151563e-50	Podocyte
STX7	1.7222300323743e-54	0.458678600389506	0.456	0.282	4.98068925362646e-50	Podocyte
CCDC18-AS1	2.54439064546937e-54	0.520584278804669	0.558	0.386	7.35837774669741e-50	Podocyte
PHLDB1	3.11780167054525e-54	0.510005496727994	0.257	0.13	9.01668243121685e-50	Podocyte
FTX	7.70899044839488e-54	0.29310163287664	0.878	0.867	2.2294400376758e-49	Podocyte
MBOAT2	1.80409558803006e-53	0.394132285002256	0.379	0.22	5.21744444058293e-49	Podocyte
ARAP2	2.37642344820931e-53	0.48786898675378	0.422	0.257	6.87261661222132e-49	Podocyte
GNAI1	3.15133600190286e-53	0.81836826434444	0.302	0.17	9.11366371750307e-49	Podocyte
RCAN1	1.30037886628168e-52	0.866906824420563	0.271	0.146	3.76069568128661e-48	Podocyte
SLC2A13	1.80343312353455e-52	0.620777002578288	0.399	0.243	5.21552859326192e-48	Podocyte
ARGLU1	1.98396239599122e-52	0.444024721664001	0.604	0.419	5.73761924920661e-48	Podocyte
ZNF280D	2.10551178846e-52	0.462540774207124	0.48	0.308	6.08914009222633e-48	Podocyte
RAB18	2.16453583131338e-52	0.5537890702993	0.304	0.166	6.2598376241583e-48	Podocyte
PTPN9	2.70683610668629e-52	0.498477847450714	0.346	0.198	7.82817002053676e-48	Podocyte
SLF2	4.15829607568851e-52	0.400353548201158	0.361	0.208	1.20257922508912e-47	Podocyte
MT-CO1	3.25938399882021e-51	1.07969659167607	0.797	0.673	9.42613852458806e-47	Podocyte
WDSUB1	7.82792024974606e-51	0.565271730154753	0.341	0.198	2.26383453622656e-46	Podocyte
ATP9A	7.97306269776677e-51	0.532954850653678	0.276	0.146	2.30580973219415e-46	Podocyte
RSPH3	1.32465194970936e-50	0.429782393583376	0.269	0.142	3.83089343855946e-46	Podocyte
PLXNB2	1.55306558555169e-50	0.59388484076471	0.252	0.13	4.49146567341549e-46	Podocyte
CREB3L2	2.15534263001725e-50	0.333879374277379	0.409	0.243	6.23325088600988e-46	Podocyte
PHF21A	3.64302795692842e-50	0.450014306259199	0.522	0.349	1.0535636851437e-45	Podocyte
MT-ND2	4.01261519186507e-50	0.863619385270634	0.461	0.308	1.16044831348738e-45	Podocyte
DDX17	6.1827618943688e-50	0.349039955348429	0.822	0.719	1.78805473985146e-45	Podocyte
NUP58	1.19250904657348e-49	0.526061891928216	0.308	0.173	3.44873616269049e-45	Podocyte
JARID2	1.22921076814394e-49	0.446546885610538	0.543	0.367	3.55487754147228e-45	Podocyte
MYLK	2.16077211166363e-49	0.251117541819805	0.348	0.193	6.24895294693121e-45	Podocyte
TNPO3	2.56090363765139e-49	0.504556008332494	0.392	0.238	7.40613332008781e-45	Podocyte
C5orf56	2.96348988766989e-49	0.431280407610258	0.304	0.167	8.57041275514132e-45	Podocyte
ACTG1	3.34633148082115e-49	0.682550671160881	0.309	0.174	9.67759064253475e-45	Podocyte
LINC01278	6.21170384023276e-49	0.392993458736295	0.662	0.491	1.79642475059531e-44	Podocyte
SPATA6	7.89916199707571e-49	0.400842988773476	0.477	0.307	2.28443764955429e-44	Podocyte

gene	p_val	avg_log2FC	pct.1	pct.2	p_val_adj	cluster
SMAD1	2.99835053582934e-48	0.503392587378659	0.325	0.187	8.67122974961846e-44	Podocyte
WRN	3.31934715367213e-48	0.435276990932032	0.34	0.198	9.5995519684198e-44	Podocyte
USP53	4.80687195191251e-48	0.353070898232667	0.591	0.425	1.3901473684931e-43	Podocyte
ARNT	4.81695554696033e-48	0.472481195884795	0.305	0.17	1.39306354418093e-43	Podocyte
DIAPH2	5.69050583407642e-48	0.254656682782182	0.433	0.263	1.6456942872149e-43	Podocyte
ELOVL5	9.08056338258539e-48	0.33492108487828	0.293	0.16	2.6260989302437e-43	Podocyte
PSAP	1.55287515733711e-47	0.729509205996293	0.321	0.187	4.49091495501891e-43	Podocyte
MTRNR2L12	2.31219817313583e-47	3.19771579777243	0.515	0.396	6.68687711670882e-43	Podocyte
MT-CO3	3.13292830822858e-47	0.851684712213596	0.591	0.45	9.06042866739705e-43	Podocyte
LINC01006	3.35377359230523e-47	0.428651144566581	0.278	0.152	9.69911322894673e-43	Podocyte
MCPH1	3.6264619512254e-47	0.379419945414334	0.46	0.296	1.04877279629439e-42	Podocyte
PHIP	3.92326548871182e-47	0.437321342919611	0.618	0.458	1.13460837933546e-42	Podocyte
MT-ND5	5.46634357936723e-47	0.973574031437043	0.456	0.302	1.580866563153e-42	Podocyte
MT-ND4L	1.05240978538426e-46	0.903643171627017	0.302	0.176	3.04356909933129e-42	Podocyte
SCARB2	1.05458029766251e-46	0.626759124911303	0.279	0.154	3.04984622083998e-42	Podocyte
ODF2L	1.49832740860362e-46	0.309562525991444	0.429	0.266	4.33316286568166e-42	Podocyte
SEL1L	4.34547012707339e-46	0.676188766253861	0.254	0.137	1.25670996074962e-41	Podocyte
ABI2	4.98647402418335e-46	0.457879943381998	0.447	0.287	1.44208828779383e-41	Podocyte
RHEB	5.87952483498606e-46	0.592294602222798	0.331	0.195	1.70035858227797e-41	Podocyte
AL035530.2	8.17384835060551e-46	0.435484301245427	0.25	0.132	2.36387694299511e-41	Podocyte
MT-CYB	1.14430630804342e-45	0.930578131715259	0.444	0.307	3.30933384286156e-41	Podocyte
FSIP2-AS1	1.8005670172637e-45	0.555225136646245	0.263	0.144	5.20723981392663e-41	Podocyte
ZNF516	2.17304170863983e-45	0.523659566014251	0.435	0.281	6.28443662138638e-41	Podocyte
ATXN3	5.10588535523453e-45	0.465998846723983	0.304	0.173	1.47662204473383e-40	Podocyte
CDC42BPA	5.1510249422078e-45	0.387098166718912	0.694	0.553	1.4896764132865e-40	Podocyte
COX10-AS1	5.74814235837398e-45	0.524978006511085	0.258	0.141	1.66236277004175e-40	Podocyte
TNPO1	8.28463735111785e-45	0.504786590296891	0.452	0.293	2.39591712194328e-40	Podocyte
PDK4	1.39905296125054e-44	0.427891370497409	0.45	0.293	4.04606116393655e-40	Podocyte
SSBP3	1.019794492779e-43	0.43958198021761	0.451	0.292	2.94924567311688e-39	Podocyte
TMBIM4	1.17437873458334e-43	0.484182247765819	0.333	0.198	3.39630330041501e-39	Podocyte
SPAG16	1.58717908796948e-43	0.418041245907842	0.523	0.363	4.59012192240773e-39	Podocyte
TIAL1	2.47850929843425e-43	0.359061703303773	0.345	0.205	7.16784889107185e-39	Podocyte
ACTB	4.13790086549961e-43	0.659722583468118	0.423	0.271	1.19668093030249e-38	Podocyte
CNTLN	5.36676153282793e-42	0.334285059574442	0.38	0.234	1.55206743529384e-37	Podocyte
NT5C2	7.42784202000645e-42	0.379775242250959	0.594	0.434	2.14813191218587e-37	Podocyte
CEP83	1.04016116530363e-41	0.467073873263934	0.334	0.203	3.0081460900581e-37	Podocyte
NEK11	1.45289130598104e-41	0.401997844476988	0.311	0.183	4.20176165689717e-37	Podocyte
PLD1	1.46810876912099e-41	0.307931110249577	0.304	0.175	4.2457705602979e-37	Podocyte
AC046134.2	2.77528339404112e-41	0.417470815306216	0.393	0.248	8.02611957556692e-37	Podocyte
CPSF6	3.74286865186012e-41	0.414538825265052	0.424	0.272	1.08243761411795e-36	Podocyte
CTNNA1	6.55222266556452e-41	0.420993221286041	0.634	0.479	1.89490279488126e-36	Podocyte
RFX3	7.51813660538818e-41	0.497415100587915	0.485	0.333	2.17424510627826e-36	Podocyte

gene	p_val	avg_log2FC	pct.1	pct.2	p_val_adj	cluster
SHTN1	8.16852771831006e-41	0.350097535254236	0.411	0.259	2.36233821613527e-36	Podocyte
NPHP3	8.25834937401001e-41	0.360313903021252	0.404	0.255	2.38831463896369e-36	Podocyte
FNBP1L	1.11886900335828e-40	0.422992065141527	0.66	0.518	3.23576915771213e-36	Podocyte
HIPK3	1.54867225747173e-40	0.344608848672927	0.51	0.349	4.47876016860826e-36	Podocyte
ABCC1	1.69417641638951e-39	0.317867410084159	0.276	0.157	4.89955819619846e-35	Podocyte
TAF3	2.74123597599097e-39	0.423175517704458	0.432	0.283	7.92765444256588e-35	Podocyte
MFSD14C	2.92902526788992e-39	0.374910338625234	0.477	0.323	8.47074107473766e-35	Podocyte
CDC27	3.87405074851239e-39	0.481251029122874	0.351	0.216	1.12037547646978e-34	Podocyte
DENND6A	5.16605816576409e-39	0.415578347592605	0.304	0.18	1.49402402153897e-34	Podocyte
SRSF10	8.09604374870408e-39	0.387176530765353	0.456	0.299	2.34137585212522e-34	Podocyte
YEATS2	1.19893501892584e-38	0.322956881953347	0.267	0.152	3.46732007473354e-34	Podocyte
MARK1	1.21549222129234e-38	0.51195181704664	0.279	0.162	3.51520350397744e-34	Podocyte
DUSP16	1.29823227201446e-38	0.436510090726774	0.298	0.177	3.75448773066583e-34	Podocyte
HIPK2	1.34308033839495e-38	0.269393886375593	0.696	0.549	3.8841883386382e-34	Podocyte
SMAD4	1.87541786239282e-38	0.338370459369413	0.371	0.233	5.42370845804002e-34	Podocyte
LATS2	1.96054473687966e-38	0.281234694830292	0.36	0.222	5.66989537905597e-34	Podocyte
APC	2.71527400512588e-38	0.341504871823385	0.408	0.262	7.85257242282405e-34	Podocyte
KPNA5	3.85011439395582e-38	0.427707960794198	0.256	0.145	1.11345308273202e-33	Podocyte
OSBPL9	5.88295993778255e-38	0.363128189157929	0.561	0.406	1.70135201400671e-33	Podocyte
MT-ND4	1.06209958194621e-37	0.739917426717464	0.487	0.362	3.07159199098845e-33	Podocyte
POLK	1.21067298582618e-37	0.306286622884031	0.426	0.277	3.50126627500931e-33	Podocyte
TRAF3	1.21729396978685e-37	0.353603009229535	0.309	0.185	3.52041416062356e-33	Podocyte
TTC14	3.41924969125318e-37	0.324458087534886	0.512	0.352	9.88847010710419e-33	Podocyte
STXBP5	4.2046098345566e-37	0.359396288581401	0.468	0.317	1.21597316415377e-32	Podocyte
PJA2	4.50136161272107e-37	0.26876671128566	0.385	0.243	1.30179377839893e-32	Podocyte
TNRC6C	5.48922639360779e-37	0.294557774706648	0.384	0.243	1.58748427303137e-32	Podocyte
PDCD6IP	1.02695838755873e-36	0.347628724727616	0.413	0.27	2.96996365681985e-32	Podocyte
KRCC1	1.1881603739935e-36	0.342759115615246	0.289	0.17	3.43615980158921e-32	Podocyte
NAP1L1	1.78794204929634e-36	0.430886462031005	0.351	0.218	5.17072840656501e-32	Podocyte
VPS13C	2.75472831603984e-36	0.287325034151085	0.599	0.437	7.96667428998721e-32	Podocyte
AUTS2	3.91466775159284e-36	0.303993263130622	0.565	0.417	1.13212191376065e-31	Podocyte
RNF150	1.31722086974641e-35	0.275295546980284	0.508	0.349	3.80940275530662e-31	Podocyte
TMEM168	1.50964324461442e-35	0.394184460374332	0.288	0.172	4.36588826342491e-31	Podocyte
ITGB1	2.80688511205058e-35	0.344894666868451	0.364	0.232	8.11751174405029e-31	Podocyte
TMEM14B	3.70517788582267e-35	0.420684936532266	0.432	0.291	1.07153744457992e-30	Podocyte
CNTN4	4.93353563747179e-35	0.644239987157905	0.452	0.332	1.42677850635684e-30	Podocyte
MT-ND1	6.0221157012851e-35	0.866403101317043	0.467	0.333	1.74159586081165e-30	Podocyte
EXOC5	6.58373720455469e-35	0.541267674158499	0.254	0.147	1.90401679955722e-30	Podocyte
Z93930.2	7.60552388006617e-35	0.357115104187242	0.322	0.2	2.19951750611514e-30	Podocyte
ALG13	8.82862362882982e-35	0.295881224942816	0.324	0.199	2.55323795345758e-30	Podocyte
ICE2	1.22568008637538e-34	0.309499141269913	0.329	0.204	3.54466680979759e-30	Podocyte
FAR1	1.82592384622424e-34	0.421067828701635	0.36	0.231	5.28057176328049e-30	Podocyte

gene	p_val	avg_log2FC	pct.1	pct.2	p_val_adj	cluster
ATXN2	1.95685989162039e-34	0.365285669784342	0.647	0.513	5.65923880656616e-30	Podocyte
MTMR3	2.05591771370399e-34	0.331474160772192	0.507	0.357	5.94571402803193e-30	Podocyte
BTBD1	2.38530399672616e-34	0.400058886467839	0.254	0.147	6.89829915853205e-30	Podocyte
PSMD1	3.81333215139183e-34	0.36797367564767	0.402	0.265	1.10281565818252e-29	Podocyte
PBX3	5.07517943803696e-34	0.441769058174097	0.335	0.213	1.46774189348029e-29	Podocyte
MT-ATP6	6.87301240182702e-34	0.770970481025288	0.439	0.312	1.98767518660837e-29	Podocyte
MAP3K2	9.66985449231061e-34	0.294027682472654	0.492	0.338	2.79652191917623e-29	Podocyte
DHFR	1.19034260853859e-33	2.50592754085031	0.493	0.391	3.44247082389361e-29	Podocyte
SMAD2	3.6674427829536e-33	0.315676480734835	0.488	0.34	1.06062445283018e-28	Podocyte
ASCC3	3.75245643864434e-33	0.290717673243593	0.49	0.34	1.08521040205594e-28	Podocyte
NFE2L1	4.26027949504657e-33	0.39389040150696	0.258	0.151	1.23207282996747e-28	Podocyte
MROH1	7.79354793131099e-33	0.421612127175902	0.343	0.218	2.25389406173514e-28	Podocyte
CD2AP	2.38336912521723e-32	0.307182531623586	0.595	0.448	6.89270351012822e-28	Podocyte
RNF115	3.19478440052706e-32	0.33489185807839	0.483	0.338	9.23931648632426e-28	Podocyte
MMP24OS	5.64154904800532e-32	0.346809226498366	0.253	0.149	1.63153598468314e-27	Podocyte
MYO5C	8.75903511075336e-32	0.294429350976296	0.3	0.185	2.53311295402987e-27	Podocyte
ZNF718	1.2242419584579e-31	0.372727726531701	0.279	0.169	3.54050774386025e-27	Podocyte
TRAF3IP2-AS1	1.53664657349489e-31	0.388812256317827	0.29	0.18	4.44398189054723e-27	Podocyte
ZMYM4	7.29007940437236e-31	0.311962811744246	0.591	0.441	2.10829096374449e-26	Podocyte
FER	1.13467786673274e-30	0.289414364636692	0.586	0.439	3.28148839059109e-26	Podocyte
KCTD20	1.1425938208527e-30	0.316809450942566	0.265	0.161	3.30438132990602e-26	Podocyte
NFIC	1.71511578451732e-30	0.375822644057786	0.48	0.335	4.96011484882409e-26	Podocyte
LONRF1	4.08961987417571e-30	0.333629327567527	0.258	0.155	1.18271806761161e-25	Podocyte
GOLGA8B	4.5488613476641e-30	0.288524406852275	0.37	0.243	1.31553070174446e-25	Podocyte
EPS15	6.16904760790133e-30	0.25378859064039	0.563	0.416	1.78408856820507e-25	Podocyte
TENT2	8.11712963343424e-30	0.309842594122302	0.58	0.432	2.34747388998918e-25	Podocyte
CRYZL1	1.40587561428074e-29	0.394165659993461	0.251	0.151	4.0657922764999e-25	Podocyte
AOPEP	1.4440280860572e-29	0.326523163496618	0.721	0.62	4.17612922487744e-25	Podocyte
FBXL2	1.58850381907141e-29	0.362720606861691	0.325	0.211	4.59395304475452e-25	Podocyte
RBM4	1.78251372692728e-29	0.299431119771526	0.25	0.15	5.15502969827369e-25	Podocyte
NBEAL1	1.8436459148395e-29	0.288206369511047	0.655	0.52	5.33182398571582e-25	Podocyte
ZDHHC21	2.6341182936852e-29	0.330874657212241	0.436	0.303	7.6178701053376e-25	Podocyte
LINC00271	8.85522012819123e-29	0.321224592145688	0.462	0.326	2.5609296610729e-24	Podocyte
KIF1B	1.05502977164641e-28	0.27985388418474	0.578	0.434	3.05114609960142e-24	Podocyte
CASD1	6.3970756044511e-28	0.400571274199368	0.27	0.169	1.85003426480726e-23	Podocyte
ATF2	7.54759933834562e-28	0.354326048025772	0.318	0.206	2.18276572864955e-23	Podocyte
TASOR	1.61867848224576e-27	0.28462244605844	0.396	0.268	4.68121817065473e-23	Podocyte
C18orf25	2.38397398259626e-27	0.375274787191872	0.26	0.162	6.89445275766838e-23	Podocyte
SLC38A2	2.77388087267711e-27	0.296538185215344	0.27	0.168	8.02206348378219e-23	Podocyte
KIF5B	3.04388790716394e-27	0.308547879844042	0.332	0.217	8.80292382751812e-23	Podocyte
NCKAP1	3.73621631606176e-27	0.308714090502133	0.475	0.343	1.08051375860506e-22	Podocyte
LUC7L2	4.63200901497434e-27	0.283363334752485	0.534	0.392	1.33957700713058e-22	Podocyte

gene	p_val	avg_log2FC	pct.1	pct.2	p_val_adj	cluster
TARSL2	5.69180213775468e-27	0.279693202073429	0.279	0.177	1.64606917823865e-22	Podocyte
NOVA1	7.4907506926486e-27	0.312691964131399	0.254	0.157	2.16632510031398e-22	Podocyte
BCKDHB	7.65478252792889e-27	0.457059341801309	0.515	0.393	2.21376310707704e-22	Podocyte
DDX5	1.18961464372315e-26	0.349621043111741	0.617	0.482	3.44036554964735e-22	Podocyte
CHIC2	1.49142156173275e-26	0.259065077900999	0.331	0.217	4.31319115653112e-22	Podocyte
LINC01004	1.65473837421159e-26	0.254701858993704	0.426	0.297	4.78550337821993e-22	Podocyte
B2M	4.86227125361578e-26	0.70455680241994	0.297	0.195	1.40616884654568e-21	Podocyte
ZRANB2	5.63583260170246e-26	0.274440528167627	0.64	0.495	1.62988278841235e-21	Podocyte
MTMR2	5.93757297995994e-26	0.26688220721337	0.274	0.175	1.71714610580442e-21	Podocyte
FAM126B	6.23019826607427e-26	0.343398526852766	0.36	0.244	1.80177333854868e-21	Podocyte
TM9SF2	6.3587398814665e-26	0.351839794482077	0.277	0.176	1.83894757372011e-21	Podocyte
METTL15	8.44223759858442e-26	0.278423662411271	0.533	0.395	2.44149511351061e-21	Podocyte
TCF7L2	1.35228720752454e-25	0.369991978749296	0.556	0.424	3.91081460416098e-21	Podocyte
TMEM165	1.53355787142892e-25	0.25284961700772	0.378	0.257	4.43504936417243e-21	Podocyte
SRSF5	1.54512856006932e-25	0.250887456317779	0.496	0.359	4.46851179572049e-21	Podocyte
SEPTIN10	4.09089330985713e-25	0.292029716951172	0.301	0.196	1.18308634521068e-20	Podocyte
CDC42	8.02216060106986e-25	0.379375683189325	0.253	0.159	2.3200088458294e-20	Podocyte
FBXW7	8.94653868145365e-25	0.274643965654803	0.501	0.368	2.5873389866764e-20	Podocyte
RIOK3	2.75847065691662e-24	0.274169294008127	0.316	0.207	7.97749713980286e-20	Podocyte
MT-ND3	5.13010992248332e-24	0.660279034030714	0.296	0.203	1.48362778958218e-19	Podocyte
ZFAND6	2.72111728640444e-23	0.253376688938232	0.502	0.368	7.86947119228165e-19	Podocyte
DLGAP4	3.15204969380327e-23	0.278516949968775	0.279	0.181	9.11572771447906e-19	Podocyte
ST5	5.07133341784806e-23	0.268469905191904	0.255	0.163	1.46662962444166e-18	Podocyte
PAPOLA	7.11485620213101e-23	0.258932196856028	0.438	0.313	2.05761641365629e-18	Podocyte
UBA6-AS1	8.33842232025719e-22	0.293302401750683	0.496	0.382	2.41147173501838e-17	Podocyte
MORF4L1	1.15928691658309e-21	0.301138633538706	0.289	0.192	3.35265776275829e-17	Podocyte
YWHAZ	1.43200899377702e-21	0.295541468298601	0.415	0.296	4.14137001000314e-17	Podocyte
PPP4R2	2.06041172186166e-21	0.258765799982702	0.327	0.224	5.95871069962391e-17	Podocyte
CTSD	6.80434805407953e-21	0.751874269275782	0.272	0.185	1.9678174572398e-16	Podocyte
CSTF3	2.51705155845517e-20	0.30283962467973	0.268	0.179	7.27931310705235e-16	Podocyte
CNTN3	4.46014803428485e-20	0.299838038767937	0.291	0.197	1.28987481151518e-15	Podocyte
NCOA1	8.89756435913909e-20	0.252787200872717	0.67	0.558	2.57317561266302e-15	Podocyte
DUSP1	3.18277090710008e-17	0.32520576241223	0.279	0.196	9.20457346333342e-13	Podocyte
CCNI	3.7283589703176e-17	0.302108664501813	0.292	0.204	1.07824141421585e-12	Podocyte
CANX	1.59039836510918e-16	0.297060202240928	0.299	0.213	4.59943207189575e-12	Podocyte
CIRBP	1.72756014354577e-16	0.263443126461262	0.537	0.414	4.99610393513438e-12	Podocyte
HEATR5A	3.72887846133105e-16	0.257766541692741	0.297	0.211	1.07839165101694e-11	Podocyte
HSP90B1	1.23805710791444e-13	0.283346717123819	0.319	0.238	3.58046115608855e-09	Podocyte
PTMA	4.77949357661423e-11	0.350894308320815	0.285	0.214	1.38222954235683e-06	Podocyte
APLP2	7.94345890282676e-08	0.271014224044403	0.29	0.227	0.0022972483146975	Podocyte
FP236383.3	8.8621464473302e-07	0.828530239260363	0.848	0.781	0.0256293275256789	Podocyte
EEF1A1	1.61297133138446e-06	0.458295168032704	0.322	0.265	0.0466471309036386	Podocyte

gene	p_val	avg_log2FC	pct.1	pct.2	p_val_adj	cluster
NAV2	0.00478599865596512	0.273037451389671	0.395	0.375	1	Podocyte
ARHGAP15	0	7.43393644297796	0.8	0.025	0	Immune Cell
PTPRC	0	7.03504746366252	0.719	0.024	0	Immune Cell
CELF2	0	3.65245782043601	0.816	0.136	0	Immune Cell
CHST11	0	4.99623285982917	0.702	0.072	0	Immune Cell
ELMO1	0	3.91535741755442	0.725	0.111	0	Immune Cell
DOCK2	0	7.54156431987666	0.614	0.009	0	Immune Cell
PRKCB	0	7.36081436812245	0.598	0.013	0	Immune Cell
IKZF1	0	8.15512019867672	0.59	0.006	0	Immune Cell
ANKRD44	0	4.2067716422715	0.75	0.176	0	Immune Cell
DOCK10	0	4.03979215376511	0.602	0.091	0	Immune Cell
RIPOR2	0	5.14305734679413	0.566	0.057	0	Immune Cell
INPP5D	0	5.35784051419952	0.537	0.029	0	Immune Cell
SMAP2	0	5.06060316248731	0.586	0.084	0	Immune Cell
FLI1	0	4.42625361681232	0.539	0.043	0	Immune Cell
CAMK1D	0	4.27592334476631	0.566	0.076	0	Immune Cell
ITGA4	0	7.7184396793052	0.481	0.006	0	Immune Cell
FYN	0	4.20853485563355	0.533	0.088	0	Immune Cell
FYB1	0	6.14005664303761	0.467	0.023	0	Immune Cell
DOCK8	0	2.9253183192706	0.638	0.203	0	Immune Cell
STK17B	0	5.34235713090758	0.458	0.034	0	Immune Cell
TNFAIP8	0	4.39344904212523	0.503	0.079	0	Immune Cell
RUNX1	0	2.72304789378978	0.642	0.219	0	Immune Cell
CD44	0	4.97467876066358	0.434	0.03	0	Immune Cell
ETS1	0	4.31122580966553	0.501	0.098	0	Immune Cell
PREX1	0	4.05351368544324	0.439	0.05	0	Immune Cell
APBB1IP	0	4.44333000459961	0.425	0.041	0	Immune Cell
STK4	0	3.5813568597178	0.511	0.133	0	Immune Cell
PRKCH	0	3.76804256563782	0.517	0.14	0	Immune Cell
PIP4K2A	0	3.17695715901466	0.641	0.265	0	Immune Cell
CD53	0	7.30379199407927	0.38	0.005	0	Immune Cell
SH3KBP1	0	3.61699557875766	0.482	0.107	0	Immune Cell
PARP8	0	4.37939875841673	0.504	0.137	0	Immune Cell
CD96	0	6.67877994127206	0.377	0.012	0	Immune Cell
CARD11	0	6.7619503370055	0.373	0.009	0	Immune Cell
CYTIP	0	4.6286607451683	0.408	0.045	0	Immune Cell
TMEM131L	0	3.57501051996567	0.478	0.117	0	Immune Cell
RCSD1	0	6.23848710911937	0.368	0.011	0	Immune Cell
DOCK11	0	3.65488627209527	0.424	0.068	0	Immune Cell
RHOH	0	8.16540503007064	0.356	0.003	0	Immune Cell
AOAH	0	6.43673153258735	0.373	0.023	0	Immune Cell
CLEC2D	0	4.00976895608082	0.441	0.096	0	Immune Cell

gene	p_val	avg_log2FC	pct.1	pct.2	p_val_adj	cluster
EMB	0	6.60839371237732	0.352	0.009	0	Immune Cell
IL7R	0	8.41934620814843	0.345	0.004	0	Immune Cell
SLC9A9	0	3.91844067238865	0.407	0.069	0	Immune Cell
CCND3	0	2.90862124536654	0.551	0.215	0	Immune Cell
CAMK4	0	6.88073925388748	0.347	0.012	0	Immune Cell
ITK	0	8.41497346345886	0.334	0.003	0	Immune Cell
FOXN3	0	2.4827189921247	0.74	0.41	0	Immune Cell
IFI16	0	3.98463791448148	0.373	0.045	0	Immune Cell
THEMIS	0	8.05908805529965	0.333	0.005	0	Immune Cell
ARHGAP25	0	4.86414099315049	0.356	0.031	0	Immune Cell
AFF3	0	4.80066199473898	0.395	0.076	0	Immune Cell
STK17A	0	3.73636838088919	0.419	0.104	0	Immune Cell
BCL11B	0	6.66499862781191	0.326	0.015	0	Immune Cell
DISC1	0	3.96858616959027	0.371	0.062	0	Immune Cell
PAG1	0	4.04061635490053	0.373	0.064	0	Immune Cell
IKZF3	0	7.73286914454963	0.311	0.003	0	Immune Cell
CD247	0	7.45875410486919	0.313	0.007	0	Immune Cell
PIK3R5	0	7.66720615945972	0.309	0.003	0	Immune Cell
SLA	0	6.808312045	0.308	0.005	0	Immune Cell
AKNA	0	5.26690079989335	0.323	0.021	0	Immune Cell
LINC01934	0	6.37734403268075	0.317	0.017	0	Immune Cell
STK10	0	3.92793955146996	0.353	0.057	0	Immune Cell
XYLT1	0	3.68111088407126	0.359	0.063	0	Immune Cell
STAT4	0	6.15696244596771	0.308	0.014	0	Immune Cell
SP140	0	6.56901995996666	0.285	0.007	0	Immune Cell
LCP1	0	6.86960229270687	0.281	0.006	0	Immune Cell
RASA3	0	4.32864543446505	0.314	0.041	0	Immune Cell
SEL1L3	0	3.77923984312993	0.334	0.064	0	Immune Cell
NFATC2	0	3.80161460137591	0.312	0.043	0	Immune Cell
RASSF3	0	4.0529649300164	0.314	0.046	0	Immune Cell
TMC8	0	7.95583522824872	0.271	0.003	0	Immune Cell
SRGN	0	5.32061309167719	0.285	0.018	0	Immune Cell
LCP2	0	6.58074220167627	0.272	0.007	0	Immune Cell
MCUB	0	5.46669550472494	0.283	0.019	0	Immune Cell
TRAF3IP3	0	5.76310054943716	0.274	0.011	0	Immune Cell
SAMSN1	0	6.28001000344882	0.268	0.009	0	Immune Cell
NLRP1	0	3.99796418895233	0.287	0.028	0	Immune Cell
P2RY8	0	4.9529146467539	0.267	0.013	0	Immune Cell
GNG2	0	5.08199101220213	0.272	0.02	0	Immune Cell
SLFN12L	0	7.12541243745922	0.256	0.004	0	Immune Cell
NCKAP1L	0	7.58414628060519	0.253	0.002	0	Immune Cell
HCLS1	0	5.41622364380195	0.258	0.011	0	Immune Cell

gene	p_val	avg_log2FC	pct.1	pct.2	p_val_adj	cluster
PVT1	0	3.76784377423007	0.311	0.065	0	Immune Cell
ST8SIA4	0	5.76092369516387	0.252	0.009	0	Immune Cell
MBNL1	0	2.49956996714885	0.844	0.604	0	Immune Cell
CIITA	0	4.37178200370599	0.277	0.043	0	Immune Cell
HLA-DRB1	0	4.32488461279852	0.263	0.03	0	Immune Cell
RNASET2	0	3.920134093	0.279	0.047	0	Immune Cell
PDE4B	0	4.71729991510449	0.259	0.028	0	Immune Cell
TRIM22	0	3.76093058570061	0.273	0.045	0	Immune Cell
NLRC5	0	3.83735473542994	0.258	0.042	0	Immune Cell
CD74	5.35154598497278e-296	3.74985057945619	0.286	0.059	1.54766709885413e-291	Immune Cell
TTC39C	3.3697263633523e-286	3.81011936957271	0.367	0.101	9.74524864281486e-282	Immune Cell
FNBP1	1.21177051752849e-278	2.5052252190734	0.6	0.282	3.50444033669238e-274	Immune Cell
ATM	6.4909068277041e-265	2.55129911092632	0.607	0.307	1.87717025457202e-260	Immune Cell
RESF1	5.79083799075452e-261	2.64539095232811	0.526	0.216	1.67471034692621e-256	Immune Cell
CEP85L	6.62601890512249e-260	3.02555701517691	0.45	0.161	1.91624466736142e-255	Immune Cell
LRRC8C	3.54940283953715e-258	3.49531312816592	0.253	0.052	1.02648730119414e-253	Immune Cell
WIPF1	5.73742860162435e-255	2.62796444978426	0.458	0.164	1.65926435158976e-250	Immune Cell
CDC42SE2	9.98300048885286e-255	2.52889013554581	0.609	0.317	2.88708374137625e-250	Immune Cell
UTRN	3.31911820850816e-251	1.80023709583158	0.74	0.494	9.59888985900559e-247	Immune Cell
MGAT5	1.18923550205836e-247	2.64364328869065	0.592	0.294	3.43926907195277e-243	Immune Cell
SMCHD1	9.09596557470081e-239	2.43955725350297	0.645	0.395	2.63055324420348e-234	Immune Cell
FOXP1	1.03298672217461e-237	1.77696029340978	0.789	0.631	2.98739760052898e-233	Immune Cell
LDLRAD4	7.92829524604044e-236	2.42394730900683	0.555	0.25	2.2928629851549e-231	Immune Cell
TBXAS1	1.18271311840858e-232	4.22658627030864	0.28	0.069	3.42040633843762e-228	Immune Cell
CCDC88C	1.1028975971261e-223	2.95317522849824	0.338	0.1	3.18957985088869e-219	Immune Cell
MT-CO2	4.13475778926454e-213	1.90181619599614	0.708	0.457	1.1957719526553e-208	Immune Cell
JAZF1	1.94407201930712e-212	3.19091502006423	0.403	0.149	5.62225627983619e-208	Immune Cell
SEPTIN6	6.6650145502042e-212	2.84147168665875	0.403	0.149	1.92752220791905e-207	Immune Cell
ST6GAL1	2.7421843161517e-202	1.92875012505043	0.57	0.279	7.93039704231072e-198	Immune Cell
SSH2	6.13100728258624e-195	1.91929524772297	0.69	0.494	1.77308730612394e-190	Immune Cell
TC2N	3.84084441995399e-193	3.86013216419465	0.301	0.091	1.11077220625069e-188	Immune Cell
RFTN1	1.46369045710751e-192	2.67726550520835	0.253	0.063	4.23299280195491e-188	Immune Cell
FCHSD2	1.49222541934998e-192	2.45875746434764	0.599	0.364	4.31551591276015e-188	Immune Cell
CYTH1	1.22092112819696e-190	2.33224305514401	0.546	0.294	3.5309039027456e-186	Immune Cell
LYN	1.44354630796648e-184	3.7906988400699	0.27	0.076	4.17473592263906e-180	Immune Cell
MT-CO3	1.56821464242477e-182	1.82868167846357	0.679	0.449	4.53527674589242e-178	Immune Cell
PTK2B	4.7081800178342e-181	2.97706317924743	0.33	0.112	1.36160566115765e-176	Immune Cell
MT-ND4	1.32484903744314e-176	1.89201175224952	0.616	0.36	3.83146341628557e-172	Immune Cell
PDCD4	1.79454330957357e-173	3.02834827492559	0.342	0.124	5.18981925128676e-169	Immune Cell
CNTRL	2.12052963206254e-172	2.78187576578473	0.323	0.11	6.13257169592486e-168	Immune Cell
SP110	2.03401955344209e-163	2.85180391895259	0.278	0.086	5.88238454855452e-159	Immune Cell
ZC3HAV1	1.8899624274774e-162	2.65175024709269	0.345	0.129	5.46577134026464e-158	Immune Cell

gene	p_val	avg_log2FC	pct.1	pct.2	p_val_adj	cluster
DPYD	8.61032242589595e-162	2.23566986205732	0.581	0.379	2.49010524556911e-157	Immune Cell
HLA-E	1.89636885582087e-161	2.56652142455724	0.273	0.083	5.48429873103397e-157	Immune Cell
NCK2	2.53860143973673e-160	3.05021410943964	0.373	0.156	7.34163536371861e-156	Immune Cell
PDE7A	4.53866458718191e-158	2.00367634091901	0.576	0.353	1.31258179861301e-153	Immune Cell
HLA-B	1.67808984485435e-149	2.4350001429298	0.295	0.1	4.85303583131877e-145	Immune Cell
MT-CYB	9.09038603138912e-149	1.89079764495198	0.542	0.306	2.62893964027773e-144	Immune Cell
HDAC9	5.12526640100274e-147	3.02823046856344	0.296	0.103	1.48222704316999e-142	Immune Cell
RPS19	1.47618050483342e-142	2.68414456367214	0.274	0.091	4.26911401997826e-138	Immune Cell
ZFP36L2	4.61719978605682e-140	2.49063361609126	0.314	0.117	1.33529417812763e-135	Immune Cell
MYCBP2	1.84490585818998e-134	1.65004591649946	0.616	0.458	5.33546774188542e-130	Immune Cell
PDE3B	2.82704159396745e-131	2.67228989911463	0.417	0.207	8.17580428975387e-127	Immune Cell
AKAP13	2.94176048519582e-131	1.44320847884901	0.7	0.59	8.50757132318631e-127	Immune Cell
EVL	2.89271927130934e-129	2.48230341732052	0.386	0.184	8.3657441326266e-125	Immune Cell
GRK3	3.43150771895807e-125	3.20349628653153	0.277	0.104	9.92392032322674e-121	Immune Cell
PCED1B	8.24656790949046e-124	2.2394781156743	0.325	0.131	2.38490743942464e-119	Immune Cell
RPS6KA3	8.2540921402832e-124	2.36309696881664	0.419	0.221	2.3870834469699e-119	Immune Cell
CDC14A	4.8424304663284e-121	2.18834486164752	0.386	0.186	1.40043089086217e-116	Immune Cell
ETV6	7.44715353796344e-121	2.16214786601051	0.533	0.367	2.15371680317903e-116	Immune Cell
TBC1D22A	2.00222179279072e-120	1.69308668248596	0.627	0.502	5.79042542475076e-116	Immune Cell
PPP2R5C	6.16381958795291e-120	2.28795055781523	0.498	0.323	1.78257662483598e-115	Immune Cell
CD47	2.278677753892e-119	2.72426440518217	0.295	0.119	6.58993606425567e-115	Immune Cell
MT-CO1	3.46550443256033e-118	0.906706406638658	0.802	0.674	1.00222388189645e-113	Immune Cell
TPK1	4.82733411592327e-118	2.53148487569155	0.312	0.13	1.39606502632501e-113	Immune Cell
SERINC5	3.13096957969781e-117	2.89385575560139	0.357	0.171	9.05476402448607e-113	Immune Cell
PACS1	4.98252545861931e-117	1.80310544505494	0.583	0.428	1.4409463626327e-112	Immune Cell
RAPGEF1	2.40293795925162e-116	2.01118146827468	0.507	0.33	6.9492965781557e-112	Immune Cell
IFNAR2	1.08904282376918e-115	2.42714412101674	0.296	0.118	3.14951184634046e-111	Immune Cell
RPS6KA5	3.89517779196961e-115	2.43476467033593	0.297	0.12	1.12648541743761e-110	Immune Cell
MT-ATP6	2.98116618416633e-114	1.62369965800728	0.519	0.312	8.62153260460903e-110	Immune Cell
SARAF	4.25507912494177e-112	2.82015872961642	0.269	0.104	1.23056888293316e-107	Immune Cell
SP100	1.31069914280865e-111	1.90406223643561	0.47	0.277	3.79054192100261e-107	Immune Cell
TNRC6B	2.42612078902686e-111	1.15171229146266	0.732	0.681	7.01634132186569e-107	Immune Cell
STIM1	1.71630057294818e-110	2.18753986048788	0.384	0.193	4.96354125696612e-106	Immune Cell
SFMBT2	1.93959047131182e-109	2.5832910502564	0.394	0.209	5.60929564303378e-105	Immune Cell
MEF2C	5.24588342693926e-109	2.5794169801728	0.362	0.178	1.51710948707083e-104	Immune Cell
CNOT6L	5.53654393036241e-109	2.39874331666949	0.463	0.283	1.60116850466081e-104	Immune Cell
FAM49B	2.87280148591498e-107	1.7046932660293	0.544	0.384	8.30814189726612e-103	Immune Cell
RASA2	6.98387154685019e-107	2.21106023798823	0.458	0.285	2.01973565134908e-102	Immune Cell
ZEB2	1.69029095931895e-106	2.83641605961544	0.314	0.139	4.88832145435041e-102	Immune Cell
INPP4A	2.83391038760767e-106	2.16468782654677	0.383	0.197	8.19566884096138e-102	Immune Cell
ELOVL5	6.73418884073035e-104	2.34546752368952	0.337	0.16	1.94752741273922e-99	Immune Cell
CYLD	3.51450180966271e-102	2.51850500857733	0.272	0.111	1.01639392335445e-97	Immune Cell

gene	p_val	avg_log2FC	pct.1	pct.2	p_val_adj	cluster
NIN	7.69806094639596e-102	2.27282632087434	0.325	0.151	2.22627922569771e-97	Immune Cell
TRERF1	3.93976349202097e-101	2.66470148214041	0.258	0.102	1.13937960189246e-96	Immune Cell
MT-ND3	5.75503488414932e-101	1.76843390452219	0.395	0.202	1.66435608849598e-96	Immune Cell
HLA-A	5.73524496303004e-100	2.23237687366143	0.269	0.108	1.65863284330829e-95	Immune Cell
TGFBR2	3.37168376006957e-98	1.71374341024926	0.446	0.262	9.7509094341212e-94	Immune Cell
SLC38A1	5.57835971892194e-98	1.92390236489094	0.434	0.255	1.61326163071223e-93	Immune Cell
MDFIC	8.99364678632278e-98	2.57441375581385	0.317	0.149	2.60096265060455e-93	Immune Cell
MYO9B	1.73829506025325e-97	2.31430625656846	0.319	0.149	5.02714931425239e-93	Immune Cell
CCM2	5.71837725793854e-96	2.22050620548357	0.342	0.17	1.65375470299582e-91	Immune Cell
CASK	2.76088216375176e-95	2.24420921016633	0.471	0.319	7.9844712175701e-91	Immune Cell
SKAP1	3.09724885460604e-95	2.25220044612711	0.512	0.386	8.95724368752066e-91	Immune Cell
RAB8B	5.05303676393921e-95	2.59052524569232	0.255	0.104	1.46133823213122e-90	Immune Cell
PCNX1	5.29431185552074e-95	1.79151966588881	0.548	0.424	1.5311149886166e-90	Immune Cell
B2M	1.46596776300985e-94	1.90022741360112	0.371	0.194	4.23957877062448e-90	Immune Cell
MAP4K4	6.6638659693171e-94	2.07837547418353	0.41	0.239	1.9271900383265e-89	Immune Cell
PPP1R16B	7.45578315679867e-93	1.82669005104738	0.362	0.179	2.15621248894618e-88	Immune Cell
DIPK1A	2.31423071303533e-92	2.37977169476239	0.253	0.102	6.69275522209818e-88	Immune Cell
EML4	2.40740026535887e-91	1.71506143956278	0.555	0.425	6.96220156741786e-87	Immune Cell
SORL1	5.1076027791526e-89	2.47643480540036	0.354	0.193	1.47711872373093e-84	Immune Cell
VOPP1	1.71798634507589e-85	2.15339222201193	0.388	0.225	4.96841650995948e-81	Immune Cell
TRAF3	2.08577979083348e-84	2.19385805210579	0.345	0.185	6.03207515509043e-80	Immune Cell
DGKD	4.52719067126871e-83	2.53541217213515	0.257	0.113	1.30926354213091e-78	Immune Cell
DLEU2	2.90269009734714e-82	1.78986998703232	0.421	0.256	8.39457976152794e-78	Immune Cell
TXNIP	3.53853126194438e-80	1.58338129341385	0.527	0.384	1.02334324095432e-75	Immune Cell
PRKCQ	2.33226645054086e-77	2.66116981347648	0.253	0.115	6.74491457496418e-73	Immune Cell
ARAP2	2.52786860445487e-76	2.03439395487728	0.407	0.259	7.31059600408348e-72	Immune Cell
TNIK	1.89028774880482e-75	2.11667154312226	0.358	0.206	5.46671216954354e-71	Immune Cell
ZEB1	2.80282603141406e-75	1.19922904082626	0.386	0.213	8.10577288284946e-71	Immune Cell
LINC00623	1.60487775245211e-74	2.20032294129722	0.319	0.173	4.6413064600915e-70	Immune Cell
MT-ND4L	1.03920975292787e-73	1.58074562436774	0.338	0.177	3.0053946054674e-69	Immune Cell
MGAT4A	3.5288481335069e-73	2.13544218045573	0.356	0.21	1.02054288021019e-68	Immune Cell
NUP210	6.49188101013246e-73	2.24855523262207	0.278	0.137	1.87745198813031e-68	Immune Cell
IQGAP2	2.32684153692907e-72	1.45236388131132	0.485	0.334	6.72922572479888e-68	Immune Cell
PARP14	1.75763873951517e-70	1.75648046692852	0.39	0.237	5.08309123467788e-66	Immune Cell
NR3C1	8.52442851805311e-70	1.84445007500173	0.384	0.236	2.46526472742096e-65	Immune Cell
USP15	1.85567917429335e-68	1.55108050270772	0.511	0.409	5.36662417205636e-64	Immune Cell
GPRIN3	2.17893183249153e-67	1.99129848315478	0.348	0.204	6.30147085956552e-63	Immune Cell
ATF7IP	2.43796113839182e-67	1.72396353123438	0.463	0.344	7.05058361222916e-63	Immune Cell
FUT8	3.82989925614356e-66	2.09803486085572	0.36	0.22	1.10760686487672e-61	Immune Cell
CEMIP2	2.86961822652562e-65	2.15397160134322	0.34	0.2	8.29893591111209e-61	Immune Cell
MT-ND2	4.59248514924388e-65	1.30496542132562	0.462	0.309	1.32814670516133e-60	Immune Cell
TANK	1.06750101283459e-64	1.99053154398917	0.385	0.255	3.08721292911762e-60	Immune Cell

gene	p_val	avg_log2FC	pct.1	pct.2	p_val_adj	cluster
RAP1A	5.56524293113103e-64	1.75720279463436	0.432	0.308	1.60946825568309e-59	Immune Cell
BAZ1A	5.71958663595122e-64	1.99790881503296	0.254	0.123	1.65410445511709e-59	Immune Cell
PHTF2	1.05624966044049e-63	2.14206271925662	0.323	0.189	3.0546740179939e-59	Immune Cell
NOTCH2	1.88217910697847e-62	2.4880895965201	0.276	0.148	5.44326197738173e-58	Immune Cell
EPB41	2.54815331693611e-62	1.73914939418589	0.438	0.321	7.36925939257924e-58	Immune Cell
PLCL2	2.73297795244502e-62	1.310899437	0.454	0.312	7.903772238471e-58	Immune Cell
ANKRD11	2.55707297618775e-60	0.963773867271946	0.641	0.59	7.39505504713496e-56	Immune Cell
RNF213	5.74518543588218e-60	1.25750352299224	0.524	0.412	1.66150762805713e-55	Immune Cell
SEMA4D	6.39679508609779e-59	1.86576534751901	0.371	0.244	1.84995313889948e-54	Immune Cell
SLC4A7	1.50712037799209e-58	1.30838168544358	0.364	0.217	4.35859213315313e-54	Immune Cell
KLF12	2.11287328091922e-56	1.52128328189095	0.535	0.469	6.11042952841838e-52	Immune Cell
BACH2	8.88723786696789e-55	2.42348553323112	0.382	0.266	2.57018919112711e-50	Immune Cell
BCL2	1.14011506200817e-54	1.66083206362196	0.525	0.443	3.29721275932763e-50	Immune Cell
KAT2B	1.13387366451717e-53	2.28274175882945	0.297	0.178	3.27916263778365e-49	Immune Cell
VPS13C	1.25622913318368e-53	1.22798949088029	0.518	0.441	3.6330146531672e-49	Immune Cell
ZCCHC7	1.99022843281624e-53	1.41948092206625	0.561	0.522	5.75574062770456e-49	Immune Cell
INPP4B	2.28209393676815e-53	1.95097436707113	0.399	0.285	6.5998156651335e-49	Immune Cell
PITPNC1	3.24574945253485e-53	1.43278952908588	0.447	0.329	9.38670741673078e-49	Immune Cell
MAP3K1	2.16968432151699e-52	1.71807488901015	0.393	0.279	6.27472705782712e-48	Immune Cell
VPS13B	2.55491135465332e-52	0.909126567287925	0.636	0.607	7.38880363765741e-48	Immune Cell
UBAC2	1.72620044790181e-50	1.53517533170484	0.467	0.389	4.99217169533205e-46	Immune Cell
KDM4C	2.15309539313533e-50	1.44352674573997	0.488	0.42	6.22675187694736e-46	Immune Cell
FBXW7	2.39391344005751e-50	1.64301472120302	0.457	0.37	6.92319766864631e-46	Immune Cell
CAMK2D	5.35300462742742e-50	1.80533481838877	0.414	0.312	1.54808893825201e-45	Immune Cell
NCOA3	7.8060727174656e-50	1.72217234838136	0.422	0.324	2.25751622989105e-45	Immune Cell
ABR	1.06353028693075e-49	1.67055708759716	0.271	0.148	3.07572958980374e-45	Immune Cell
NEDD9	7.79060698894191e-47	1.46303141538591	0.392	0.275	2.253043541202e-42	Immune Cell
CCDC91	2.68980284754455e-46	1.10693509391496	0.504	0.422	7.77890983509884e-42	Immune Cell
STIM2	7.80107658606901e-45	1.41979409379034	0.348	0.23	2.25607134869116e-40	Immune Cell
RPL13	1.00365118344804e-44	1.61103536565213	0.323	0.209	2.90255922253172e-40	Immune Cell
DIAPH2	2.56156991800337e-43	1.57098276689004	0.373	0.266	7.40806020286575e-39	Immune Cell
USP3	3.240832254915e-43	1.44731418673012	0.432	0.348	9.37248688121418e-39	Immune Cell
MBP	3.52727040478979e-43	1.61849267067373	0.382	0.282	1.02008660106521e-38	Immune Cell
ATP2B1	1.56223468506464e-42	1.74828843388132	0.382	0.288	4.51798270920695e-38	Immune Cell
ARID1B	3.18415643933397e-41	0.726527582076625	0.699	0.683	9.20858042255383e-37	Immune Cell
ASAP1	3.3355716177408e-41	1.32499307261358	0.421	0.323	9.6464731185064e-37	Immune Cell
GRB2	5.75650551522265e-40	1.84664999503354	0.3	0.199	1.66478139500239e-35	Immune Cell
RABGAP1L	9.80324883642791e-40	0.781595862235252	0.722	0.734	2.83509956349495e-35	Immune Cell
MANBA	1.10243761637231e-39	1.73582574669025	0.288	0.183	3.18824958654872e-35	Immune Cell
ARPC2	1.57300213047318e-39	1.86320158565577	0.291	0.191	4.54912216132843e-35	Immune Cell
MT-ND1	2.07686280596712e-39	1.05620763496956	0.443	0.335	6.00628723485692e-35	Immune Cell
ATXN1	1.1999805319902e-38	1.12373244551626	0.598	0.59	3.47034369851566e-34	Immune Cell

gene	p_val	avg_log2FC	pct.1	pct.2	p_val_adj	cluster
EEF1A1	5.64285666071318e-38	1.23396338877171	0.369	0.264	1.63191414627825e-33	Immune Cell
XPO6	6.15139128046644e-37	1.87692049252388	0.29	0.195	1.7789823583109e-32	Immune Cell
ITPKB	9.87891176592743e-37	1.58639865438194	0.286	0.183	2.85698128270621e-32	Immune Cell
MYH9	1.38943445358012e-36	1.24421287734875	0.411	0.324	4.0182444397537e-32	Immune Cell
UVRAG	4.23353867552009e-36	1.5648906801315	0.452	0.404	1.22433938496041e-31	Immune Cell
MSN	4.413562708087e-36	1.73271836593864	0.277	0.178	1.27640233517876e-31	Immune Cell
HIVEP2	4.69296027062591e-36	1.39198061776977	0.44	0.37	1.35720411026501e-31	Immune Cell
PIK3R1	5.12210248480437e-36	1.56969674781835	0.339	0.245	1.48131203860542e-31	Immune Cell
KANSL1	6.46256197553649e-36	0.800410649752261	0.635	0.633	1.86897292332515e-31	Immune Cell
ANKRD36	6.36746524162592e-35	1.19199801505848	0.433	0.352	1.84147094787822e-30	Immune Cell
FAM172A	1.56600751613269e-34	1.05631107951873	0.544	0.525	4.52889373665574e-30	Immune Cell
ARHGAP26	1.75617848773076e-34	1.14259527961969	0.412	0.323	5.07886818651736e-30	Immune Cell
RAPGEF6	2.6756480361114e-34	1.45692136673387	0.397	0.322	7.73797412043418e-30	Immune Cell
PLXDC2	3.46946720810727e-34	0.784054133419081	0.321	0.598	1.00336991658462e-29	Immune Cell
LINC01138	4.483363147024e-34	1.6879877034972	0.322	0.232	1.29658862211934e-29	Immune Cell
LYST	4.27414933907501e-32	1.7482162650426	0.37	0.299	1.23608398886049e-27	Immune Cell
ANKRD12	6.95025618993476e-32	0.756697618845855	0.623	0.627	2.01001409012913e-27	Immune Cell
RAP1B	8.68370946275074e-32	1.32793283676097	0.389	0.314	2.51132877662752e-27	Immune Cell
FAM53B	1.07716268107959e-31	1.80268395181305	0.275	0.187	3.11515447368218e-27	Immune Cell
SETX	2.65702964380455e-31	1.03836624947159	0.48	0.433	7.68412972988275e-27	Immune Cell
PAN3	3.24241044875769e-31	0.92439448774265	0.547	0.534	9.37705101780723e-27	Immune Cell
MAP3K5	6.93075460402641e-31	1.21404273519827	0.399	0.319	2.00437423148444e-26	Immune Cell
TBL1XR1	1.14163247877027e-30	1.11997705600495	0.491	0.453	3.30160112860362e-26	Immune Cell
PLEKHA2	2.01090563898716e-30	1.63121088965287	0.282	0.193	5.81553910795087e-26	Immune Cell
ELF1	2.08034738325248e-30	0.985658243354788	0.478	0.419	6.01636463236617e-26	Immune Cell
SMARCA2	2.56868710995808e-30	1.1963907357924	0.417	0.349	7.42864312199876e-26	Immune Cell
KAT6A	5.50629170334741e-29	1.07112659800271	0.484	0.446	1.59241956060807e-24	Immune Cell
STK38	5.72378322132444e-29	1.68836484441471	0.317	0.24	1.65531810760703e-24	Immune Cell
PHACTR2	6.16449438242244e-29	1.73191184709874	0.375	0.312	1.78277177539657e-24	Immune Cell
ST3GAL1	8.80417699546063e-29	1.51646481430082	0.284	0.196	2.54616798708721e-24	Immune Cell
COP1	9.65169324299213e-29	1.112031405	0.489	0.469	2.79126968587333e-24	Immune Cell
TBC1D5	1.56589594784755e-28	0.786565712447731	0.649	0.685	4.5285710811751e-24	Immune Cell
ACAP2	7.17789083647746e-28	1.26671171343485	0.41	0.357	2.07584602990928e-23	Immune Cell
UST	2.44312114729543e-27	1.66430808412468	0.252	0.169	7.06550635797839e-23	Immune Cell
STT3B	5.30849423158458e-27	1.61021955968213	0.336	0.269	1.53521653177426e-22	Immune Cell
DOCK4	1.80836107935791e-26	0.475374219433985	0.183	0.353	5.22978024150308e-22	Immune Cell
RNF149	3.24016108183077e-26	1.59760081767151	0.334	0.272	9.37054584865458e-22	Immune Cell
RICTOR	7.92088131592091e-26	1.17308360273845	0.436	0.397	2.29071887656433e-21	Immune Cell
CDK17	8.71694399869741e-26	1.42730340458424	0.282	0.202	2.52094020442329e-21	Immune Cell
MYO5A	9.6100050140649e-26	1.31173277645999	0.359	0.288	2.77921345006757e-21	Immune Cell
SIPA1L1	1.10214800624147e-25	1.33561993683884	0.503	0.494	3.18741203405032e-21	Immune Cell
PRDM2	1.36287800112162e-25	1.65967643716357	0.303	0.232	3.94144317924373e-21	Immune Cell

gene	p_val	avg_log2FC	pct.1	pct.2	p_val_adj	cluster
CBLB	3.31871524291993e-25	1.26230474678947	0.451	0.408	9.59772448252443e-21	Immune Cell
HERC1	8.29309088467015e-25	0.842002872950407	0.533	0.529	2.39836188384661e-20	Immune Cell
ATP8A1	9.23070881668171e-25	1.66750231245039	0.293	0.222	2.66952098978435e-20	Immune Cell
PPP3CA	1.4889497632151e-24	1.14570153368396	0.45	0.41	4.30604271521808e-20	Immune Cell
SH3BGRL	4.66933455746951e-24	1.35106225207589	0.325	0.249	1.35037155402018e-19	Immune Cell
GPCPD1	5.35907092783788e-24	1.52078660267307	0.315	0.249	1.54984331233071e-19	Immune Cell
OSBPL10	1.86967342015558e-23	0.424296391787846	0.201	0.378	5.40709553108993e-19	Immune Cell
SUSD6	4.14340764062639e-23	1.59712647135202	0.31	0.246	1.19827348966915e-18	Immune Cell
TAOK3	7.53813180014566e-23	1.14251722849989	0.443	0.415	2.18002771660213e-18	Immune Cell
PRKACB	8.23685045427624e-23	1.50204726296049	0.288	0.218	2.38209715137669e-18	Immune Cell
AKT3	1.15474435994756e-22	0.893919674013601	0.478	0.438	3.33952068896835e-18	Immune Cell
JARID2	1.33068593206714e-22	1.36398755398895	0.406	0.372	3.84834371553817e-18	Immune Cell
JAK1	1.66997637744247e-22	0.865072904297308	0.52	0.515	4.82957168356364e-18	Immune Cell
PARP4	1.99717219241515e-22	1.3945189177513	0.261	0.189	5.77582198046461e-18	Immune Cell
EPC1	2.3899087027974e-22	1.23140277252337	0.412	0.381	6.91161596849008e-18	Immune Cell
PICALM	3.44752316883135e-22	0.730755924299358	0.579	0.579	9.97023700426026e-18	Immune Cell
RBL2	3.69006916700991e-22	1.57193199660696	0.306	0.243	1.06716800309927e-17	Immune Cell
ITSN2	4.45808741979256e-22	1.03585156261645	0.44	0.408	1.28927888180401e-17	Immune Cell
PRMT2	5.4953843616013e-22	1.54899678950218	0.283	0.216	1.5892651573751e-17	Immune Cell
PCNX2	2.25796685380353e-21	1.56456039201679	0.332	0.278	6.53004014119981e-17	Immune Cell
MED13L	2.81182333659291e-21	0.626901819845809	0.642	0.653	8.1317930894267e-17	Immune Cell
CDKAL1	3.1040464606528e-21	0.811883282166529	0.538	0.551	8.97690236420791e-17	Immune Cell
GLCCI1	3.24979502749571e-21	1.32863961826645	0.36	0.298	9.39840721951761e-17	Immune Cell
LNPEP	4.49048258191531e-21	1.4029663795417	0.321	0.265	1.29864756268991e-16	Immune Cell
ANKRD36C	4.65195013511703e-21	1.04359324661235	0.351	0.285	1.34534397907584e-16	Immune Cell
CRYBG1	6.19215863678551e-20	1.34629289793547	0.317	0.252	1.79077227775837e-15	Immune Cell
ROCK1	7.82486238476576e-20	1.0152668026074	0.435	0.412	2.26295020167426e-15	Immune Cell
ACTR3	2.2303341558883e-19	1.39232066711005	0.309	0.253	6.45012637882898e-15	Immune Cell
PRIM2	4.5172014185168e-19	1.63455118357285	0.259	0.197	1.30637465023506e-14	Immune Cell
PBX3	1.60680892072153e-18	1.47185288154037	0.277	0.215	4.64689139872668e-14	Immune Cell
DENND4A	1.62287949293372e-18	1.09628829181796	0.41	0.384	4.69336749356432e-14	Immune Cell
ATF7IP2	2.75273884605764e-18	1.49407592953143	0.272	0.213	7.9609207427987e-14	Immune Cell
KDM6A	3.43402929262022e-18	1.02571202623805	0.456	0.45	9.93121271425766e-14	Immune Cell
MAML2	6.00899628330717e-18	0.511809367168663	0.706	0.716	1.73780172513243e-13	Immune Cell
FKBP5	1.0581885460628e-17	0.442286629075054	0.585	0.527	3.06028127521362e-13	Immune Cell
SND1	1.20506927043389e-17	1.1298526930547	0.41	0.399	3.48506033009481e-13	Immune Cell
BIRC6	1.33026117673477e-17	0.675336537323579	0.563	0.602	3.84711532311696e-13	Immune Cell
SRGAP1	1.80073528402062e-17	0.44257560518425	0.154	0.281	5.20772644138763e-13	Immune Cell
BBX	2.17084683138417e-17	0.964619397852505	0.458	0.459	6.27808903636303e-13	Immune Cell
SAT1	3.20730209711027e-17	0.474955055756681	0.268	0.439	9.27551766484289e-13	Immune Cell
KMT2E	4.00683220813276e-17	0.814196397804687	0.502	0.517	1.15877587459199e-12	Immune Cell
SENP7	4.155043513265e-17	1.18425463004928	0.359	0.32	1.20163858403624e-12	Immune Cell

gene	p_val	avg_log2FC	pct.1	pct.2	p_val_adj	cluster
EPS15	5.75254307047909e-17	1.06473180730789	0.428	0.421	1.66363545598255e-12	Immune Cell
CAPZB	1.7430117820889e-16	1.17483889523196	0.374	0.347	5.04079007380109e-12	Immune Cell
SUCLG2	3.49714904406839e-16	1.04085699912232	0.456	0.463	1.01137550354458e-11	Immune Cell
NIPBL	6.67899696413544e-16	0.691448241627979	0.54	0.576	1.93156592202797e-11	Immune Cell
ZFHX3	2.77750350832953e-15	0.279010175355618	0.191	0.325	8.03254014608899e-11	Immune Cell
XKR6	3.78658348697944e-15	1.14787603780846	0.39	0.372	1.09507994443445e-10	Immune Cell
TOX	7.438015260721e-15	1.44459098902807	0.336	0.292	2.15107401340051e-10	Immune Cell
NCOA2	8.43807574277657e-15	0.669735510853016	0.542	0.566	2.44029150481098e-10	Immune Cell
ARID4B	9.78150442887905e-15	0.848416599484926	0.478	0.494	2.82881108083182e-10	Immune Cell
MT-ND5	1.19700206182666e-14	0.673391486745226	0.362	0.306	3.4617299628027e-10	Immune Cell
PPP3CC	1.39957312279465e-14	1.37139964037007	0.305	0.266	4.04756547112214e-10	Immune Cell
FRYL	3.43605588986262e-14	0.918019216616226	0.441	0.448	9.93707363348269e-10	Immune Cell
ARHGEF18	3.5161272627471e-14	1.50961018003134	0.261	0.213	1.01686400438646e-09	Immune Cell
FOCAD	6.47403973441274e-14	0.267737109624122	0.194	0.325	1.87229229119216e-09	Immune Cell
BMP2K	7.86353950026975e-14	1.96683306371612	0.268	0.228	2.27413562347801e-09	Immune Cell
TAB2	9.64733621832836e-14	1.39208096211113	0.323	0.295	2.79000963434056e-09	Immune Cell
SMYD3	9.65306511649834e-14	0.780673147081854	0.577	0.625	2.79166643169132e-09	Immune Cell
NFKB1	1.57427350463545e-13	1.36327629766291	0.277	0.233	4.55279897540572e-09	Immune Cell
WAC	1.64253140917942e-13	0.919686911705554	0.443	0.452	4.75020083534688e-09	Immune Cell
OSBPL8	2.07385977961148e-13	0.983576334776619	0.408	0.397	5.99760248263640e-09	Immune Cell
STAG1	3.82134507572893e-13	0.603574485087233	0.564	0.605	1.10513299590081e-08	Immune Cell
SNX9	4.1435743915615e-13	0.373816088009436	0.217	0.357	1.19832171403959e-08	Immune Cell
CHD2	5.87131615646486e-13	0.750175522264782	0.5	0.518	1.69798463244964e-08	Immune Cell
RAD51B	5.97741611888465e-13	0.783836485026191	0.496	0.497	1.72866874158144e-08	Immune Cell
EXOC4	7.10632434401832e-13	0.538164101737281	0.607	0.659	2.0551490002901e-08	Immune Cell
CMIP	1.10969815082284e-12	0.925484326385018	0.452	0.457	3.20924705217966e-08	Immune Cell
ZFAND3	1.64551633954443e-12	0.48015091744494	0.666	0.71	4.7588332539625e-08	Immune Cell
SIK3	2.09218774796684e-12	0.536000050516807	0.63	0.667	6.05060696712011e-08	Immune Cell
NEK7	2.3940336235768e-12	1.2355361087108	0.365	0.352	6.9235452393841e-08	Immune Cell
AGTPBP1	4.5810233898737e-12	1.39108108414156	0.257	0.216	1.32483196435148e-07	Immune Cell
OXNAD1	6.9974330738511e-12	1.59725872040411	0.291	0.265	2.02365764495774e-07	Immune Cell
PUM2	7.68762465092183e-12	0.842974322029622	0.446	0.468	2.22326104904659e-07	Immune Cell
NSF	1.07013160439002e-11	1.28860226626921	0.329	0.311	3.09482059989594e-07	Immune Cell
GBF1	1.10607651409802e-11	0.313999169292553	0.244	0.392	3.19877327877146e-07	Immune Cell
SYNRG	1.16414339248869e-11	1.15934226683907	0.315	0.288	3.36670269107728e-07	Immune Cell
PIAS1	1.31973842772449e-11	0.86451623089274	0.417	0.425	3.81668353297922e-07	Immune Cell
OGT	1.78572014484385e-11	1.17057490233983	0.346	0.329	5.16430265888842e-07	Immune Cell
MAN1A1	3.19408543412166e-11	0.583110634162695	0.25	0.39	9.23729507547985e-07	Immune Cell
ABI1	4.84170588257258e-11	1.18825916886575	0.306	0.282	1.40022134123999e-06	Immune Cell
MAGED1	4.85664730995849e-11	0.250692245803979	0.197	0.31	1.40454240204e-06	Immune Cell
ASCC3	6.22865147684611e-11	1.08859723035016	0.356	0.345	1.8013260071039e-06	Immune Cell
PHF21A	8.43694820403817e-11	1.11202179901834	0.363	0.354	2.43996542060784e-06	Immune Cell

gene	p_val	avg_log2FC	pct.1	pct.2	p_val_adj	cluster
TRAPPC9	9.05496463358624e-11	0.71872264872107	0.518	0.563	2.61869577203314e-06	Immune Cell
EXOC2	9.55151159267273e-11	1.31233359047736	0.293	0.268	2.76229715260095e-06	Immune Cell
MECP2	1.10959400183362e-10	1.2355739208917	0.295	0.269	3.20894585330284e-06	Immune Cell
NAA15	1.27342860727844e-10	0.257702851702397	0.163	0.266	3.68275553224926e-06	Immune Cell
LPIN2	1.50645408397267e-10	1.0819397210779	0.277	0.238	4.35666521084895e-06	Immune Cell
UBE2R2	2.17065441141672e-10	0.290064809971645	0.225	0.351	6.27753255781715e-06	Immune Cell
ARID5B	3.86807508528204e-10	0.863793584732701	0.434	0.434	1.11864731466356e-05	Immune Cell
AP2B1	4.27549120274597e-10	0.402875701569473	0.178	0.288	1.23647205583413e-05	Immune Cell
DYRK1A	4.50321265325459e-10	0.779348555931436	0.48	0.509	1.30232909932123e-05	Immune Cell
TET2	5.77530917471713e-10	0.25798448101191	0.238	0.363	1.67021941332819e-05	Immune Cell
PHC3	6.47277001360732e-10	1.01443935765637	0.376	0.381	1.87192508793524e-05	Immune Cell
STX7	6.89602160570901e-10	1.32360248928883	0.307	0.288	1.99432944837105e-05	Immune Cell
SMAD3	8.96217933748491e-10	0.883766192971772	0.367	0.356	2.59186226440063e-05	Immune Cell
ZHX2	9.45630857049213e-10	0.939925342502808	0.393	0.395	2.73476443858632e-05	Immune Cell
MOB3B	1.13590290749648e-09	0.340714237452001	0.168	0.263	3.28503120847981e-05	Immune Cell
CWC27	1.28258983502453e-09	0.260447425863506	0.172	0.271	3.70924980289093e-05	Immune Cell
FMN1	1.28725432074944e-09	0.261520736771336	0.184	0.279	3.72273949560737e-05	Immune Cell
RAB3GAP2	1.84705571765646e-09	0.254406296200739	0.215	0.331	5.34168513546248e-05	Immune Cell
MAML3	2.64050294795266e-09	0.511754230204345	0.255	0.383	7.63633452547911e-05	Immune Cell
HERC4	2.71639025730991e-09	0.81409342844182	0.466	0.498	7.85580062414025e-05	Immune Cell
TYW1	2.91135645011026e-09	0.38079302769319	0.198	0.307	8.41964285371886e-05	Immune Cell
YTHDC1	3.42216477787613e-09	0.327720704757848	0.214	0.327	9.89690053761776e-05	Immune Cell
LCORL	3.98808913898145e-09	0.271850341275031	0.218	0.331	0.000115335537899344	Immune Cell
SEPTIN7	4.43701133541574e-09	1.31124669130299	0.283	0.266	0.000128318367820223	Immune Cell
RBM33	4.43755186063237e-09	0.878380329398321	0.408	0.422	0.000128333999809488	Immune Cell
NPIPB5	4.4415138157878e-09	0.843835246548693	0.338	0.318	0.000128448579552583	Immune Cell
TULP4	5.94607039964758e-09	0.31598834656202	0.163	0.257	0.000171960355957808	Immune Cell
RAF1	6.09566490615462e-09	0.298973677290701	0.16	0.252	0.000176286629085992	Immune Cell
SMAP1	6.33649941186537e-09	0.26702221280609	0.251	0.379	0.000183251562991146	Immune Cell
AGFG1	6.88792302205161e-09	0.368050672370917	0.19	0.296	0.000199198733797733	Immune Cell
TTC37	7.3240015838439e-09	0.305569701048605	0.185	0.286	0.000211810125804766	Immune Cell
NFATC3	8.04912168968613e-09	1.03012905298368	0.33	0.317	0.000232780599265723	Immune Cell
ERBIN	8.43993631407684e-09	0.754772665945726	0.467	0.51	0.000244082958203102	Immune Cell
CUL1	8.97806425870874e-09	0.2772250872731	0.17	0.264	0.000259645618361857	Immune Cell
TCERG1	9.97591441724233e-09	0.320435884079685	0.183	0.283	0.000288503444946648	Immune Cell
XPO4	1.0630016610538e-08	0.408948512650449	0.165	0.258	0.000307420080376759	Immune Cell
FUS	1.22573914067959e-08	1.04825746808815	0.335	0.327	0.000354483759484537	Immune Cell
RSRC1	1.34477498719863e-08	1.00376615595744	0.351	0.351	0.000388908926297844	Immune Cell
VTI1A	1.39944039844497e-08	0.539947699384698	0.495	0.537	0.000404718163230287	Immune Cell
EMSY	1.4802361557717e-08	0.274008153411863	0.187	0.285	0.000428084296249176	Immune Cell
NT5DC1	1.87760406138122e-08	0.344065997134038	0.251	0.376	0.000543003094551449	Immune Cell
DNAJC1	2.09422661068844e-08	0.262077012534267	0.274	0.407	0.000605650335811097	Immune Cell

gene	p_val	avg_log2FC	pct.1	pct.2	p_val_adj	cluster
LMBR1	2.14452297568129e-08	0.313399020457493	0.22	0.331	0.000620196044567028	Immune Cell
KMT2C	2.14817205613524e-08	0.469749743998503	0.579	0.649	0.000621251358634311	Immune Cell
XRCC5	2.38626625233225e-08	1.06049015000348	0.338	0.334	0.000690108200174485	Immune Cell
FP236383.3	2.63838464350202e-08	0.26984559115721	0.753	0.784	0.000763020838900784	Immune Cell
HNRNPM	3.38480166004583e-08	0.390048664799282	0.179	0.275	0.000978884640085255	Immune Cell
NUBPL	3.4383898794414e-08	0.311205810395285	0.242	0.358	0.000994382353134452	Immune Cell
PTAR1	3.61881051830124e-08	0.371601778638052	0.165	0.256	0.00104656000189272	Immune Cell
UBE2W	3.73765975151211e-08	0.276390535906047	0.201	0.305	0.0010809312001373	Immune Cell
gene	p_val	avg_log2FC	pct.1	pct.2	p_val_adj	cluster
STRN3	3.86280422676408e-08	0.25495561778156	0.285	0.421	0.00111712298238017	Immune Cell
CAMKMT	4.48787271039261e-08	0.264398064034342	0.36	0.517	0.00129789278784554	Immune Cell
CREB3L2	5.05276833627157e-08	0.583961085781429	0.165	0.251	0.00146126060284974	Immune Cell
CCDC66	5.19505183598182e-08	0.298083409291176	0.174	0.263	0.00150240899096594	Immune Cell
PTBP2	5.8226046728822e-08	0.309785382905871	0.242	0.362	0.00168389727139753	Immune Cell
RNGTT	6.07176163881861e-08	1.1318358545987	0.327	0.327	0.00175595346594634	Immune Cell
MAP2K4	7.17273053581318e-08	0.317318238007447	0.237	0.353	0.00207435367095717	Immune Cell
PTPN1	9.04189564275268e-08	1.10623261653844	0.292	0.277	0.00261491621988408	Immune Cell
FARS2	9.05790983517911e-08	0.888296432392606	0.424	0.459	0.0026195475243338	Immune Cell
STAG2	1.21365439569625e-07	1.03781691495709	0.326	0.324	0.00350988851235356	Immune Cell
STK39	1.24418666837521e-07	0.939500487008696	0.356	0.351	0.0035981878449411	Immune Cell
FAM168A	1.31998244463437e-07	0.585639976193018	0.172	0.264	0.0038173892298826	Immune Cell
SUPT3H	1.44951167678949e-07	0.331931437106916	0.277	0.404	0.00419198776927519	Immune Cell
FBXO42	1.67530617403182e-07	0.387497154316703	0.187	0.283	0.00484498545530002	Immune Cell
SDCCAG8	1.83961004232674e-07	0.288263216775273	0.299	0.435	0.00532015224240894	Immune Cell
FBXL4	2.25152095948289e-07	0.398291999137646	0.183	0.272	0.00651139861482453	Immune Cell
DCAF5	2.29096760172896e-07	0.260822257528102	0.233	0.344	0.00662547830420016	Immune Cell
PHF20	2.3136014605763e-07	1.06891405868305	0.301	0.291	0.00669093542398667	Immune Cell
FOXO1	2.50839308421938e-07	0.72286156612756	0.448	0.473	0.00725427279956244	Immune Cell
MAN2A1	2.62447581087911e-07	0.953574833633777	0.349	0.35	0.00758998404506238	Immune Cell
RNF115	2.93296375439449e-07	0.462318753541167	0.234	0.346	0.00848213117770885	Immune Cell
BTRC	3.27222814856748e-07	0.293102311378629	0.225	0.332	0.00946328380565714	Immune Cell
DGLUCY	3.32326670594417e-07	0.251859031333843	0.259	0.373	0.00961088731359054	Immune Cell
RFX3	3.56400772415883e-07	0.991277443465612	0.34	0.338	0.0103071103382673	Immune Cell
PPP3R1	4.71751786310617e-07	1.25813882069894	0.294	0.287	0.0136430616601031	Immune Cell
ARHGAP24	4.9613752292737e-07	0.998178334251543	0.362	0.545	0.0143482971630595	Immune Cell
ARMC8	5.16078287388002e-07	0.387481957054195	0.181	0.27	0.014924984071261	Immune Cell
XIST	5.27730537305253e-07	0.295334085085306	0.498	0.437	0.0152619671388679	Immune Cell
CSGALNACT1	5.98907789431197e-07	1.02894320310613	0.272	0.246	0.0173204132703502	Immune Cell
COG5	6.1678491931037e-07	0.821945208327896	0.394	0.419	0.0178374198664559	Immune Cell
ZNF131	6.69469222727002e-07	0.286609885338405	0.202	0.295	0.0193610499212649	Immune Cell
RLF	8.56821669877449e-07	0.361592144281411	0.177	0.262	0.0247792826928558	Immune Cell
AMBRA1	1.12821431439592e-06	0.324324134546809	0.251	0.36	0.03262795797233	Immune Cell

gene	p_val	avg_log2FC	pct.1	pct.2	p_val_adj	cluster
EP400	1.16308185874546e-06	0.279970034682977	0.184	0.269	0.0336363273549186	Immune Cell
MTMR3	1.1947101243565e-06	0.426110439476361	0.25	0.364	0.0345510167963901	Immune Cell
APPL2	1.29284852909183e-06	0.350602736883033	0.172	0.252	0.0373891794613357	Immune Cell
PPA2	1.39686351270025e-06	0.377456766516522	0.209	0.308	0.0403972927872913	Immune Cell
ATP11B	1.60017919409275e-06	1.08540146179737	0.288	0.28	0.0462771822931625	Immune Cell
PTEN	1.64346181547098e-06	0.971086344174359	0.356	0.372	0.0475289157034207	Immune Cell
AC016831.7	1.79548580312696e-06	1.2314162048428	0.255	0.24	0.0519254494264317	Immune Cell
RASA1	2.0152729293657e-06	1.13858586497918	0.318	0.324	0.058281693117256	Immune Cell
FAM214A	2.19043834745319e-06	1.11785935358206	0.306	0.306	0.0633474770083463	Immune Cell
USP32	2.20635524782075e-06	0.255757383637425	0.235	0.336	0.0638077937669762	Immune Cell
PAM	2.41521154909881e-06	0.467917278825276	0.298	0.429	0.0698479179999376	Immune Cell
UBE2G1	2.47844728162597e-06	0.477998071511394	0.183	0.268	0.0716766953846231	Immune Cell
SPIDR	2.55694145210365e-06	0.544970499063332	0.553	0.613	0.0739467467948375	Immune Cell
RCOR1	2.7202461649959e-06	0.414158637290944	0.193	0.28	0.0786695190916815	Immune Cell
MAPK1	2.80455638297965e-06	1.15412350337682	0.294	0.288	0.0811077705957716	Immune Cell
TMEM165	2.95785164433734e-06	0.402526154622992	0.18	0.263	0.0855410695542359	Immune Cell
NSMCE2	3.13613577583572e-06	0.276660464352972	0.367	0.518	0.0906970466371692	Immune Cell
PPP1R12A	4.21118528914513e-06	0.748665173453968	0.41	0.442	0.121787478562077	Immune Cell
MALT1	4.65959547697857e-06	1.00807423910605	0.251	0.23	0.13475550119422	Immune Cell
LARS	4.97218269059367e-06	0.416008572259409	0.172	0.25	0.143795523411969	Immune Cell
MGA	5.42292597547101e-06	0.402661541602353	0.194	0.278	0.156831019210622	Immune Cell
YAF2	6.75190409847993e-06	0.414989062810331	0.195	0.276	0.195265066528039	Immune Cell
MAP4K5	6.98962874353504e-06	0.377348841958211	0.2	0.287	0.202140063263033	Immune Cell
TM9SF3	7.24171149612126e-06	0.358031363090114	0.203	0.292	0.209430296467827	Immune Cell
CHD7	7.9635219480985e-06	0.333980592201237	0.253	0.36	0.230305054739009	Immune Cell
TPR	8.06286410759065e-06	0.276389475042497	0.23	0.323	0.233178029991521	Immune Cell
TRPC4AP	8.59842052038684e-06	0.37936179264974	0.238	0.341	0.248666321449587	Immune Cell
ATRX	8.64095558610179e-06	0.361328858399305	0.563	0.637	0.249896435550064	Immune Cell
WASF2	8.65640644178135e-06	1.16413646394774	0.292	0.291	0.250343274296317	Immune Cell
DTNB	8.73902814251813e-06	0.428217598535124	0.209	0.3	0.252732693881624	Immune Cell
WWP2	8.97429910611907e-06	0.251087641793856	0.251	0.351	0.259536730148964	Immune Cell
SHOC2	9.28259326221002e-06	0.312367678634795	0.207	0.294	0.268452597143114	Immune Cell
TRAPPC12	1.00760289106857e-05	0.305065189678439	0.209	0.297	0.29139875609703	Immune Cell
AHCTF1	1.02222940216049e-05	0.325398381607462	0.192	0.275	0.295628743104814	Immune Cell
APC	1.03175332157367e-05	0.398225826562612	0.187	0.268	0.298383060599106	Immune Cell
EXOC1	1.06941254093936e-05	0.325621780509213	0.185	0.264	0.309274106839663	Immune Cell
ACBD6	1.10613481069249e-05	0.34016347278976	0.259	0.371	0.319894187252268	Immune Cell
CLASP2	1.14308557951459e-05	0.283718605107885	0.299	0.419	0.330580349595619	Immune Cell
SEPTIN2	1.14612637090833e-05	0.367134084061701	0.231	0.33	0.331459746466689	Immune Cell
HS2ST1	1.16880900666676e-05	0.599574897775241	0.175	0.253	0.338019564728027	Immune Cell
ITCH	1.30660018896338e-05	0.276373102649129	0.312	0.437	0.377868774648209	Immune Cell
AUTS2	1.31229027896106e-05	1.27059729029442	0.384	0.423	0.37951434867554	Immune Cell

gene	p_val	avg_log2FC	pct.1	pct.2	p_val_adj	cluster
LENG8	1.35435451407075e-05	0.310782921520133	0.2	0.283	0.39167932546926	Immune Cell
PPP2R5A	1.4295383885046e-05	0.286985327383962	0.185	0.26	0.413422501955529	Immune Cell
SFSWAP	1.6394134104342e-05	0.317893330861486	0.21	0.299	0.474118358297572	Immune Cell
REV3L	1.67336009646367e-05	0.372890425257028	0.298	0.42	0.483935739897294	Immune Cell
FRMD4A	1.89652508919703e-05	0.711213955590636	0.233	0.326	0.548475055795781	Immune Cell
HDLBP	2.27178033409122e-05	0.53773944023699	0.176	0.255	0.656998872619182	Immune Cell
USP33	2.2867276567126e-05	0.346789383643264	0.25	0.352	0.661321638321285	Immune Cell
MFSD14C	2.41827684981094e-05	0.383207076463235	0.235	0.331	0.699365664965324	Immune Cell
PI4KA	2.46912786012505e-05	0.275742441021027	0.247	0.346	0.714071777148165	Immune Cell
R3HDM1	2.77206762317414e-05	0.393353775668097	0.22	0.308	0.801681956621961	Immune Cell
PPP6R3	2.77521189496834e-05	0.632109217069178	0.473	0.53	0.802591280024843	Immune Cell
DENND1B	3.10058014593884e-05	1.0607717365122	0.335	0.355	0.896687778205512	Immune Cell
ZNF644	3.17820602054713e-05	0.743390696377754	0.413	0.453	0.919137181142231	Immune Cell
ACTB	3.37741935059539e-05	0.579477976774211	0.292	0.275	0.976749676192186	Immune Cell
AAK1	3.52531878845304e-05	0.722345956596176	0.466	0.525	1	Immune Cell
ZDHHC21	3.64978759281486e-05	0.488294088018613	0.218	0.309	1	Immune Cell
PSPC1	3.73322649585912e-05	0.913730710672157	0.331	0.345	1	Immune Cell
CCSER2	4.20772245854137e-05	0.390899485428613	0.275	0.387	1	Immune Cell
RAP1GDS1	4.24507062015839e-05	1.03061578644463	0.307	0.312	1	Immune Cell
TRA2B	5.43616228145486e-05	0.459188296949273	0.197	0.276	1	Immune Cell
RANBP2	5.45761204208223e-05	0.257807801821338	0.233	0.324	1	Immune Cell
PIK3C3	5.5574701442906e-05	0.435153798535432	0.194	0.274	1	Immune Cell
KMT2A	5.57339478693795e-05	0.669839747387841	0.371	0.39	1	Immune Cell
GNAS	6.53269790325105e-05	0.34505574112708	0.205	0.286	1	Immune Cell
MICU2	6.82172009369794e-05	0.373922799468908	0.279	0.392	1	Immune Cell
CCDC18-AS1	7.41900619588133e-05	0.444707029582978	0.286	0.395	1	Immune Cell
MTF2	7.55460934685134e-05	0.579376630134455	0.18	0.254	1	Immune Cell
NCOR2	7.74531217225728e-05	0.353204128501198	0.217	0.3	1	Immune Cell
RPTOR	7.86104116236436e-05	0.462282319214983	0.278	0.393	1	Immune Cell
MED13	8.36334653271049e-05	0.319491219407323	0.343	0.478	1	Immune Cell
ATXN7	8.56579158719212e-05	1.00196283409735	0.287	0.288	1	Immune Cell
TUT4	8.75317616211586e-05	0.67552597182841	0.43	0.481	1	Immune Cell
MED27	0.000106832634165482	0.621211954160138	0.178	0.252	1	Immune Cell
TOP1	0.000111223082496483	0.474112254981887	0.248	0.344	1	Immune Cell
JAK2	0.000111609889270318	1.13711067341889	0.283	0.283	1	Immune Cell
C2CD5	0.000125460244065677	0.478576957773272	0.218	0.302	1	Immune Cell
OGA	0.000126962987978653	0.641842160750132	0.408	0.447	1	Immune Cell
NAIP	0.00013440159896881	0.491363548606292	0.215	0.299	1	Immune Cell
ADNP	0.000137751936708311	0.324332367424078	0.279	0.382	1	Immune Cell
ERICH1	0.000151106666920109	0.255265339358323	0.279	0.374	1	Immune Cell
LINC01578	0.000152286710677491	0.516729170642064	0.178	0.25	1	Immune Cell
PEAK1	0.000154586445835247	0.591889500377297	0.202	0.28	1	Immune Cell

gene	p_val	avg_log2FC	pct.1	pct.2	p_val_adj	cluster
THADA	0.000167462311094456	0.85207671606317	0.369	0.403	1	Immune Cell
UPF2	0.000170781760788128	0.281335703388887	0.255	0.343	1	Immune Cell
WDR37	0.000177434143588725	0.456607850366484	0.194	0.266	1	Immune Cell
VAV3	0.000183078709130715	0.864034471346828	0.406	0.441	1	Immune Cell
6-Mar	0.000185876081238807	0.33468088726814	0.288	0.402	1	Immune Cell
RALGAPB	0.000186356200027498	0.484567601481156	0.2	0.28	1	Immune Cell
STAU1	0.000205379834911958	0.514784689251097	0.183	0.254	1	Immune Cell
SCFD1	0.000212741060457267	0.447176047843546	0.222	0.308	1	Immune Cell
PDPK1	0.000224802405632599	0.490463093448294	0.181	0.25	1	Immune Cell
CDK6	0.00023867023575834	0.318929042451728	0.226	0.303	1	Immune Cell
POU2F1	0.00023969982368224	0.392193884394449	0.254	0.345	1	Immune Cell
CAB39	0.000257326767997033	0.703769901057174	0.358	0.377	1	Immune Cell
CCNT2	0.000288355581200728	0.531267170726885	0.195	0.27	1	Immune Cell
RC3H1	0.000316466871243568	0.468349718552896	0.205	0.284	1	Immune Cell
MEF2A	0.000366784568574569	0.78179957866789	0.459	0.537	1	Immune Cell
FBXW11	0.000382546270974564	0.392828907349287	0.299	0.411	1	Immune Cell
FBXO11	0.000413228667542325	0.763626246973527	0.371	0.406	1	Immune Cell
PCF11	0.00041339800876616	0.322125741524418	0.186	0.251	1	Immune Cell
QKI	0.000416115759340877	0.486853079402029	0.361	0.5	1	Immune Cell
IQGAP1	0.000432253209181115	0.960125533978933	0.292	0.299	1	Immune Cell
USP34	0.000432856399003861	0.329105074544473	0.568	0.655	1	Immune Cell
ARHGAP35	0.000438386462273769	0.430718866757348	0.266	0.366	1	Immune Cell
NEMF	0.000479585043005232	0.262639359385254	0.317	0.429	1	Immune Cell
PAFAH1B1	0.000556820439745464	0.259958249426539	0.358	0.486	1	Immune Cell
ATG7	0.00058714056164697	1.12902267715287	0.342	0.381	1	Immune Cell
DIP2B	0.000608647968826058	0.790243466677371	0.375	0.416	1	Immune Cell
GPATCH8	0.000630397265338913	0.306175304950338	0.341	0.465	1	Immune Cell
PDSS2	0.00064288957019035	0.429442902270235	0.293	0.404	1	Immune Cell
SMARCC1	0.000677990368961068	0.265904812314111	0.314	0.422	1	Immune Cell
HNRNPD	0.00068403817175352	0.538296390424282	0.202	0.278	1	Immune Cell
CNOT4	0.000710253638431941	0.342157530163696	0.278	0.379	1	Immune Cell
UBE3C	0.000738421749788571	0.305263886343617	0.31	0.421	1	Immune Cell
PRR14L	0.000762240190748042	0.351422923294161	0.212	0.283	1	Immune Cell
CLTC	0.000766396344516647	0.37818474661971	0.243	0.33	1	Immune Cell
FTO	0.000776494194737314	0.311910475367735	0.362	0.491	1	Immune Cell
GNB1	0.000787722747422726	0.831650688213126	0.358	0.388	1	Immune Cell
SEC63	0.000803866373268208	0.378140766387149	0.207	0.278	1	Immune Cell
RELCH	0.000821138971004298	1.04960501987879	0.264	0.271	1	Immune Cell
RNF111	0.000840034631932323	0.51968522105583	0.205	0.278	1	Immune Cell
FAM13B	0.000840453747270063	0.904407923286824	0.319	0.342	1	Immune Cell
YLPM1	0.000848428471615038	0.374116072164225	0.234	0.318	1	Immune Cell
TTC39B	0.000871649092237318	0.487912597191724	0.188	0.254	1	Immune Cell

gene	p_val	avg_log2FC	pct.1	pct.2	p_val_adj	cluster
RSF1	0.000939726430425493	0.722718430301181	0.373	0.412	1	Immune Cell
ZMYND8	0.00104447865077079	0.336676201529102	0.277	0.369	1	Immune Cell
YTHDC2	0.00105892115615123	0.475951382931222	0.218	0.296	1	Immune Cell
HNRNPC	0.00108565036067957	0.589916360433387	0.456	0.522	1	Immune Cell
UBR1	0.00108577410756595	0.438244419555323	0.239	0.324	1	Immune Cell
ARID4A	0.0011835857836634	0.37103372198878	0.198	0.264	1	Immune Cell
PRKCA	0.00132940818646805	0.343863109941374	0.456	0.604	1	Immune Cell
GMDS	0.00149224421358784	0.912704635280519	0.356	0.388	1	Immune Cell
RNF13	0.00150796005675047	1.10920968645519	0.261	0.27	1	Immune Cell
PPP2R5E	0.00156116177957316	0.51273955958925	0.266	0.36	1	Immune Cell
VPS37B	0.0016206772792463	0.563321061154981	0.19	0.254	1	Immune Cell
SNX6	0.00167662400686307	0.461991881738204	0.211	0.282	1	Immune Cell
AC245297.3	0.0017261452824195	0.75204979114387	0.329	0.342	1	Immune Cell
DDX39B	0.00176576863257676	0.558581974779258	0.201	0.269	1	Immune Cell
AVL9	0.00179688130760316	0.50910827387809	0.24	0.326	1	Immune Cell
PCMTD1	0.00181365261758279	0.357199719305296	0.291	0.39	1	Immune Cell
KAT6B	0.001961141307058	0.408397941188486	0.304	0.411	1	Immune Cell
MAPK14	0.00196704383322367	0.475795729457916	0.233	0.311	1	Immune Cell
TFCP2	0.0021087094819991	0.417413713560131	0.261	0.35	1	Immune Cell
ANAPC16	0.00229434721765481	0.514775071817203	0.207	0.278	1	Immune Cell
RAP1GAP2	0.00232630303192223	0.701566066252176	0.189	0.253	1	Immune Cell
STXBP5	0.00234122960500245	0.451220331813814	0.243	0.324	1	Immune Cell
VPS53	0.00246943822578035	0.438386249525664	0.28	0.373	1	Immune Cell
NUP98	0.00248059636383377	0.492416851510221	0.216	0.288	1	Immune Cell
RPS6KC1	0.00289287703908585	0.509231397816913	0.239	0.317	1	Immune Cell
EHMT1	0.00298072252052994	0.779735605940101	0.395	0.449	1	Immune Cell
OFD1	0.00306345538759477	0.512090540109856	0.216	0.288	1	Immune Cell
TLK2	0.00316114556801984	0.524319995175491	0.236	0.32	1	Immune Cell
VPS8	0.00328388079986737	0.978925853665067	0.287	0.305	1	Immune Cell
HIVEP1	0.00338519662991426	0.470352142203024	0.218	0.287	1	Immune Cell
SMG7	0.00359076669143956	0.551012331096072	0.211	0.281	1	Immune Cell
LONP2	0.00359534366102497	0.388203958149781	0.29	0.389	1	Immune Cell
SMC5	0.00361006241474454	0.409650019724387	0.242	0.32	1	Immune Cell
CWF19L2	0.00375000871862586	0.289931925811116	0.284	0.369	1	Immune Cell
NBAS	0.00416831784124195	0.454554992536108	0.303	0.405	1	Immune Cell
ATG5	0.00444115065786112	0.567045400573755	0.188	0.25	1	Immune Cell
NRF1	0.0045213771887125	0.592478479239514	0.228	0.305	1	Immune Cell
DYM	0.00453882087979422	0.639179982568725	0.42	0.483	1	Immune Cell
TTC14	0.00466685771822753	0.401520350226712	0.272	0.36	1	Immune Cell
TMEM131	0.00479944010606978	0.701487773833795	0.39	0.447	1	Immune Cell
ADK	0.0049094444968923	0.686069605561676	0.49	0.579	1	Immune Cell
PLCG2	0.00519780115160452	0.657251338075786	0.31	0.325	1	Immune Cell

gene	p_val	avg_log2FC	pct.1	pct.2	p_val_adj	cluster
DOCK9	0.00522517881436069	1.032121779	0.245	0.25	1	Immune Cell
TCF12	0.00535825915165933	0.352532766084648	0.581	0.656	1	Immune Cell
TENT2	0.00551600757018686	0.675429696024091	0.386	0.438	1	Immune Cell
TRIP12	0.00553055883667492	0.376184284873324	0.333	0.447	1	Immune Cell
KCMF1	0.00563964273310853	0.517149297974808	0.2	0.262	1	Immune Cell
NRDC	0.00570608038355217	0.530643896398209	0.256	0.342	1	Immune Cell
PAPOLA	0.00575349375013258	0.519435044854873	0.24	0.32	1	Immune Cell
STAT5B	0.00593110087782359	0.986153139350774	0.252	0.26	1	Immune Cell
IL6ST	0.00617081523519929	0.532024976166422	0.22	0.287	1	Immune Cell
DHX36	0.00638120669566255	0.447839688020615	0.229	0.299	1	Immune Cell
STRBP	0.00651732757078093	0.777042515345754	0.259	0.351	1	Immune Cell
RABEP1	0.00669998821064701	0.578734154281042	0.242	0.323	1	Immune Cell
BRD4	0.00674995802933715	0.39820202721906	0.3	0.396	1	Immune Cell
PCM1	0.00683175991842669	0.476240998546239	0.476	0.551	1	Immune Cell
ARHGEF7	0.00698312582763402	0.467027858850773	0.288	0.384	1	Immune Cell
PRPF40A	0.00709260815767183	0.37117505545814	0.223	0.288	1	Immune Cell
TVP23C	0.00711613507136195	0.684274777762211	0.202	0.268	1	Immune Cell
ODF2L	0.00735543403744869	0.550071230142698	0.208	0.273	1	Immune Cell
ZFAND6	0.00753008269727159	0.483017987851045	0.283	0.374	1	Immune Cell
SECISBP2	0.00858450725816549	0.385593714266612	0.261	0.341	1	Immune Cell
EWSR1	0.00861688235119634	0.520605157043887	0.212	0.279	1	Immune Cell
RNF216	0.00914387704535305	0.85560209704157	0.301	0.324	1	Immune Cell
TRIM33	0.00936364892292985	0.506175571982929	0.262	0.347	1	Immune Cell
SCFD2	0.00937634311651731	0.845012888267537	0.332	0.362	1	Immune Cell
DMTF1	0.00939684366503164	0.376944568436419	0.238	0.309	1	Immune Cell
RHBDD1	0.00987936162566464	1.03597262384179	0.279	0.294	1	Immune Cell
EBF1	0	4.8046314986712	0.732	0.064	0	Stromal Cell
CACNA1C	0	4.83716067331858	0.74	0.086	0	Stromal Cell
RBMS3	0	3.72430879324958	0.869	0.224	0	Stromal Cell
SLIT3	0	4.31119052558793	0.732	0.115	0	Stromal Cell
CALD1	0	4.05287247773258	0.867	0.26	0	Stromal Cell
MEIS2	0	2.47786150163738	0.731	0.135	0	Stromal Cell
CARMN	0	8.32313569742617	0.537	0.004	0	Stromal Cell
TMTC1	0	3.29220966596703	0.647	0.127	0	Stromal Cell
MEIS1	0	4.26600673447366	0.588	0.09	0	Stromal Cell
PDE3A	0	3.70342915400697	0.643	0.172	0	Stromal Cell
DLC1	0	3.51447803933228	0.572	0.112	0	Stromal Cell
EPB41L2	0	3.46049404206669	0.535	0.101	0	Stromal Cell
KALRN	0	3.27676007819959	0.555	0.14	0	Stromal Cell
CPED1	0	5.60466395683459	0.427	0.015	0	Stromal Cell
RAPGEF5	0	3.45154167709085	0.484	0.081	0	Stromal Cell
MSRB3	0	3.92595745558167	0.456	0.055	0	Stromal Cell

gene	p_val	avg_log2FC	pct.1	pct.2	p_val_adj	cluster
FBXL7	0	2.44331176601553	0.532	0.132	0	Stromal Cell
ANO3	0	7.16442377172205	0.405	0.011	0	Stromal Cell
LHFPL6	0	4.33913019001276	0.467	0.073	0	Stromal Cell
ITGA1	0	3.04707885759282	0.607	0.214	0	Stromal Cell
TPM1	0	2.4287399154922	0.626	0.235	0	Stromal Cell
ITGA8	0	4.21690243226114	0.421	0.031	0	Stromal Cell
NTRK3	0	7.30779674663537	0.395	0.009	0	Stromal Cell
SYNPO2	0	5.96215360093924	0.4	0.019	0	Stromal Cell
NFASC	0	3.79373704728103	0.434	0.054	0	Stromal Cell
A2M	0	4.9655806105454	0.404	0.031	0	Stromal Cell
DMD	0	4.02887974030415	0.505	0.133	0	Stromal Cell
PRKG1	0	2.69841418958031	0.897	0.533	0	Stromal Cell
AKAP12	0	2.57096011144892	0.48	0.118	0	Stromal Cell
PDGFRB	0	5.5695354728054	0.381	0.02	0	Stromal Cell
AFF3	0	3.01952694834015	0.426	0.076	0	Stromal Cell
ZFPM2-AS1	0	3.85721469966006	0.383	0.036	0	Stromal Cell
FILIP1	0	5.01676436215408	0.37	0.023	0	Stromal Cell
ROBO1	0	4.63467929039925	0.44	0.099	0	Stromal Cell
RFTN1	0	3.35609799837608	0.386	0.06	0	Stromal Cell
FLNA	0	3.2802939859577	0.4	0.083	0	Stromal Cell
CDH11	0	5.01578861446482	0.333	0.023	0	Stromal Cell
ACTN1	0	3.46740222898636	0.384	0.078	0	Stromal Cell
MICAL2	0	4.57638496323762	0.335	0.032	0	Stromal Cell
NR2F2-AS1	0	2.12666496038066	0.821	0.518	0	Stromal Cell
ZFPM2	0	3.12738311818089	0.389	0.088	0	Stromal Cell
LTBP1	0	3.5939960605161	0.365	0.065	0	Stromal Cell
C7	0	7.62278126762708	0.309	0.01	0	Stromal Cell
COL25A1	0	5.82946725762604	0.314	0.023	0	Stromal Cell
COL12A1	0	6.11401502868652	0.304	0.013	0	Stromal Cell
LAMA2	0	3.98693552744206	0.345	0.057	0	Stromal Cell
TSHZ2	0	3.52513343275069	0.339	0.055	0	Stromal Cell
PRR16	0	6.86506500270438	0.291	0.007	0	Stromal Cell
ANTXR1	0	3.79224334372862	0.333	0.05	0	Stromal Cell
MRVI1	0	5.64957202636455	0.294	0.013	0	Stromal Cell
DAAM2	0	4.93006408059927	0.303	0.025	0	Stromal Cell
RUNX1T1	0	3.18946077260891	0.319	0.042	0	Stromal Cell
ACTA2	0	6.85883839254947	0.288	0.011	0	Stromal Cell
TAGLN	0	4.58600950136306	0.306	0.033	0	Stromal Cell
NEGR1	0	4.68153213441516	0.335	0.066	0	Stromal Cell
ATP10A	0	2.84972857196757	0.323	0.054	0	Stromal Cell
PDGFRA	0	6.62910107084853	0.274	0.007	0	Stromal Cell
SLC7A2	0	4.57408053808116	0.302	0.039	0	Stromal Cell

gene	p_val	avg_log2FC	pct.1	pct.2	p_val_adj	cluster
GUCY1A2	0	5.36917464256226	0.283	0.022	0	Stromal Cell
LMOD1	0	6.39222306037309	0.261	0.006	0	Stromal Cell
CRISPLD2	0	5.24396820143234	0.272	0.017	0	Stromal Cell
FILIP1L	0	4.74782683520459	0.277	0.03	0	Stromal Cell
PIEZO2	0	4.65139728639831	0.269	0.024	0	Stromal Cell
LINC00924	0	5.46841260674386	0.252	0.01	0	Stromal Cell
SVEP1	0	4.96500380189844	0.265	0.025	0	Stromal Cell
CCN1	0	4.78801352533415	0.26	0.025	0	Stromal Cell
SPARCL1	0	4.2531681264219	0.272	0.037	0	Stromal Cell
NUAK1	0	3.38654856958563	0.276	0.045	0	Stromal Cell
FSTL1	0	3.67668259311406	0.27	0.045	0	Stromal Cell
PID1	0	3.43101882391399	0.259	0.042	0	Stromal Cell
TIMP3	7.61937619914845e-295	2.81239002264412	0.573	0.213	2.20352359679373e-290	Stromal Cell
PALLD	9.55722635952413e-294	2.92790710540134	0.589	0.235	2.76394986317438e-289	Stromal Cell
SPECC1	2.95155758089542e-283	3.6799155172785	0.271	0.052	8.53590452394956e-279	Stromal Cell
TPM4	2.80874013105607e-280	3.00084836084512	0.388	0.103	8.12287645901416e-276	Stromal Cell
GRK5	1.50767851729469e-279	2.91057865976758	0.314	0.067	4.36020627201625e-275	Stromal Cell
NR2F1-AS1	2.39126184948062e-276	2.77651937575254	0.438	0.132	6.91552926869794e-272	Stromal Cell
THBS1	9.32609131407664e-266	4.60540756853562	0.276	0.057	2.69710560803096e-261	Stromal Cell
SOX5	1.14079342272125e-262	2.01802547793662	0.648	0.286	3.29917457850985e-258	Stromal Cell
ENG	4.63837776002105e-259	2.50357333717789	0.272	0.053	1.34141884819809e-254	Stromal Cell
NCALD	2.68264590448113e-255	2.65937088006548	0.347	0.087	7.75821195575942e-251	Stromal Cell
ZEB2	3.55509584351353e-249	2.80843219502351	0.43	0.137	1.02813371794411e-244	Stromal Cell
TMEM131L	1.13271496702454e-248	2.92825020172008	0.399	0.119	3.27581168463496e-244	Stromal Cell
FBN1	1.57453646902178e-248	3.29852345507935	0.26	0.052	4.553559468411e-244	Stromal Cell
SH3RF3	1.28563908444239e-242	1.88784797067471	0.367	0.096	3.7180682322074e-238	Stromal Cell
KCNT2	1.58043372251074e-239	2.32802216261552	0.28	0.06	4.57061432550106e-235	Stromal Cell
MAP3K20	2.27837091142993e-236	3.11392258303826	0.258	0.054	6.58904867585536e-232	Stromal Cell
FIGN	2.03534192069106e-231	2.57546976677195	0.421	0.135	5.88620883463855e-227	Stromal Cell
LDLRAD4	6.17685620017819e-228	1.88572081170373	0.592	0.25	1.78634681309153e-223	Stromal Cell
NID1	4.95684633826412e-220	3.03600629446769	0.276	0.064	1.43351996102598e-215	Stromal Cell
ZEB1	3.59852514548452e-215	1.68988846738859	0.53	0.21	1.04069347207412e-210	Stromal Cell
NR4A1	9.42224046664237e-215	2.95037049671722	0.488	0.194	2.72491194295297e-210	Stromal Cell
ZBTB16	1.72642031530682e-210	1.54573677184976	0.803	0.537	4.99280755186732e-206	Stromal Cell
SLCO3A1	4.30619922805124e-210	2.46647334660124	0.468	0.178	1.24535281675242e-205	Stromal Cell
UNC5C	1.55684707368104e-207	2.01075398954513	0.405	0.13	4.50240173708557e-203	Stromal Cell
INPP4B	6.38457199948899e-204	2.33453852405498	0.584	0.281	1.84641822225222e-199	Stromal Cell
SORBS1	2.20874851837675e-202	3.09269599166643	0.487	0.208	6.38770071514557e-198	Stromal Cell
PDE5A	5.85995090887193e-202	3.06122836118176	0.299	0.081	1.69469780284576e-197	Stromal Cell
RNF144A	1.01641264429719e-200	2.81792068972628	0.318	0.09	2.93946536730746e-196	Stromal Cell
LDB2	1.79304445688974e-196	0.809236967425329	0.398	0.124	5.18548456932513e-192	Stromal Cell
DGKG	8.3738628508213e-191	2.78455154553909	0.322	0.095	2.42172113645752e-186	Stromal Cell

gene	p_val	avg_log2FC	pct.1	pct.2	p_val_adj	cluster
SLC38A2	4.87141710358466e-189	2.94304005368945	0.425	0.166	1.40881382635668e-184	Stromal Cell
TRERF1	2.47471352773996e-188	2.66737425864393	0.33	0.1	7.15687152222397e-184	Stromal Cell
LIMA1	5.29795878890599e-187	1.96892038176885	0.523	0.232	1.53216968175161e-182	Stromal Cell
NEXN	7.07841722496428e-185	3.11730054154759	0.282	0.077	2.04707826145967e-180	Stromal Cell
ZBTB46	6.41974407753595e-182	2.16732166144319	0.413	0.151	1.8565899872234e-177	Stromal Cell
ENAH	1.29586290265021e-181	2.99805022222455	0.344	0.113	3.74763551446442e-177	Stromal Cell
PDLIM5	3.38495411372132e-179	2.53075798542982	0.64	0.402	9.78928729688205e-175	Stromal Cell
EYA4	2.90855787652645e-174	2.02821742128942	0.298	0.084	8.4115493789145e-170	Stromal Cell
MEG3	1.87694351232811e-172	2.14055407081138	0.255	0.064	5.42812063765289e-168	Stromal Cell
UTRN	1.76213969024596e-169	1.53131925135778	0.729	0.495	5.09610798419132e-165	Stromal Cell
ARHGAP42	1.53437954482983e-168	2.0338030105699	0.468	0.2	4.43742564364787e-164	Stromal Cell
TCF7L1	6.03535698756004e-168	1.78042271562013	0.385	0.133	1.74542524080236e-163	Stromal Cell
CNTN1	3.13917407156455e-163	2.78486496882497	0.263	0.073	9.07849141496469e-159	Stromal Cell
VCL	2.13591247957731e-162	2.04365157147682	0.519	0.253	6.17705889093758e-158	Stromal Cell
ROR1	6.54874060621117e-158	2.2351963765589	0.429	0.179	1.89389578331627e-153	Stromal Cell
GNA14	7.7579629489574e-157	2.65197008911856	0.257	0.071	2.24360288483848e-152	Stromal Cell
RBPMS	3.69801423173072e-151	1.24940013048749	0.821	0.688	1.06946571581653e-146	Stromal Cell
NRP1	5.04891021454658e-151	1.66723882294599	0.572	0.296	1.46014483404687e-146	Stromal Cell
PLSCR4	1.24861918583422e-147	2.42025782345363	0.319	0.109	3.61100668543256e-143	Stromal Cell
FOS	7.60711173721422e-144	2.82467721017452	0.32	0.112	2.19997671440235e-139	Stromal Cell
SSBP2	1.93146430547405e-142	1.64954284149764	0.586	0.337	5.58579477143095e-138	Stromal Cell
TCF4	2.46588302263312e-139	1.46960662325398	0.351	0.127	7.13133370145497e-135	Stromal Cell
CAMK1D	1.20544936195005e-136	1.94486017295764	0.271	0.083	3.48615955475955e-132	Stromal Cell

gene	p_val	avg_log2FC	pct.1	pct.2	p_val_adj	cluster
COL4A1	1.62564554542469e-132	3.18092658411862	0.492	0.265	4.7013669173682e-128	Stromal Cell
EPS8	1.56105901478726e-131	1.80404705463481	0.567	0.332	4.51458267076474e-127	Stromal Cell
EMCN	3.45282015245061e-128	0.298137290860445	0.334	0.117	9.98555588088718e-124	Stromal Cell
MYO1D	8.29601700845751e-127	1.67185855762691	0.562	0.335	2.39920811884591e-122	Stromal Cell
EPAS1	8.01691351671232e-123	1.3047306764642	0.419	0.188	2.3184913890332e-118	Stromal Cell
PIP5K1B	2.55259104732534e-121	3.20171527794287	0.401	0.2	7.38209330886489e-117	Stromal Cell
THSD4	2.68186309772378e-121	1.50016635047743	0.555	0.311	7.75594807861718e-117	Stromal Cell
RARB	3.86571122345872e-120	1.91195518264189	0.392	0.174	1.11796368582426e-115	Stromal Cell
MEF2C	7.19687085943581e-120	1.88495725706908	0.397	0.177	2.08133505254884e-115	Stromal Cell
MAP3K5	3.49113839792268e-119	1.87843303794009	0.532	0.316	1.00963722467924e-114	Stromal Cell
COL4A2	1.32726086216738e-116	1.81157795968479	0.584	0.366	3.83843841338807e-112	Stromal Cell
ROBO2	4.61452171434636e-116	3.55139646704855	0.264	0.091	1.33451967978897e-111	Stromal Cell
MYLK	1.56169499185492e-115	1.99301135201342	0.411	0.194	4.51642191644443e-111	Stromal Cell
ATP11C	3.95956200316635e-115	2.01284436188224	0.384	0.173	1.14510533131571e-110	Stromal Cell
PLPP1	4.76547235112602e-115	1.36707645344797	0.532	0.303	1.37817460394564e-110	Stromal Cell
RAPH1	1.38728007073663e-114	2.14808219598604	0.355	0.155	4.01201396457032e-110	Stromal Cell
FCHSD2	2.8860287597641e-114	1.38566779273198	0.584	0.365	8.34639517323776e-110	Stromal Cell
DOCK10	3.45913130601656e-114	1.61493403459576	0.284	0.099	1.00038077369999e-109	Stromal Cell

gene	p_val	avg_log2FC	pct.1	pct.2	p_val_adj	cluster
FRMD4A	3.82698065555838e-114	1.25371183936814	0.554	0.319	1.10676280558748e-109	Stromal Cell
CLIC4	1.01501200684496e-111	1.83681272247547	0.426	0.205	2.93541472379562e-107	Stromal Cell
ZFP36	1.9500828001417e-108	2.19577720858693	0.278	0.102	5.63963945800981e-104	Stromal Cell
TACC1	1.05850790174739e-107	1.3547632652423	0.694	0.477	3.06120485185345e-103	Stromal Cell
ARHGAP10	3.63508151073208e-105	1.72927520426425	0.463	0.24	1.05126557290372e-100	Stromal Cell
LPP	1.92682096682525e-103	1.06886403246443	0.817	0.749	5.57236623605861e-99	Stromal Cell
PSD3	3.95325234826096e-101	1.44836275370373	0.389	0.177	1.14328057911707e-96	Stromal Cell
NFIA	3.33441855614585e-100	1.35448422643961	0.632	0.433	9.64313846437378e-96	Stromal Cell
AUTS2	4.51280840710807e-99	1.38779329185165	0.606	0.418	1.30510419133565e-94	Stromal Cell
ARHGEF17	6.46487743024719e-99	1.9160113841292	0.315	0.133	1.86964255282749e-94	Stromal Cell
ROCK2	1.03855798558988e-96	1.41991108146397	0.581	0.402	3.00350969432593e-92	Stromal Cell
RCAN2	4.93158929902934e-95	1.70464601028274	0.427	0.221	1.42621562527929e-90	Stromal Cell
SNED1	1.44844628754091e-94	1.3784333096343	0.335	0.144	4.1889066635683e-90	Stromal Cell
BCL6	7.19949168656937e-93	2.39476923605879	0.271	0.108	2.08209299575586e-88	Stromal Cell
OSMR	1.36042180825197e-91	1.92990426956196	0.324	0.144	3.93433986946471e-87	Stromal Cell
PARM1	1.89840762484907e-91	1.90916244839347	0.281	0.112	5.4901948510635e-87	Stromal Cell
DIAPH2	6.2267639610746e-91	1.38790389844934	0.469	0.264	1.80078013754277e-86	Stromal Cell
MAML3	3.63361456714318e-90	1.51371168287316	0.557	0.375	1.05084133281781e-85	Stromal Cell
IGFBP7	2.81522490239514e-86	1.46404452377896	0.648	0.499	8.14163041772675e-82	Stromal Cell
SOX13	1.52269287481233e-85	1.88550580358078	0.305	0.137	4.40362779395726e-81	Stromal Cell
AKT3	2.06459982687054e-84	1.05356850450346	0.623	0.435	5.97082269930961e-80	Stromal Cell
PTEN	9.47598139556878e-84	1.60208615775971	0.527	0.368	2.74045381959849e-79	Stromal Cell
ROCK1	1.99139400951787e-83	1.29049923232663	0.573	0.409	5.75911147552568e-79	Stromal Cell
APBB2	7.2667031502939e-83	0.916866993991187	0.7	0.553	2.101530551065e-78	Stromal Cell
MIR4435-2HG	4.66574134489871e-82	1.73330847543222	0.366	0.186	1.34933239694471e-77	Stromal Cell
DST	2.89752166788762e-81	0.854659397881549	0.685	0.489	8.37963266353101e-77	Stromal Cell
CRIM1	4.03441992510855e-79	1.7874472602797	0.571	0.417	1.16675424234139e-74	Stromal Cell
KIRREL1	9.75000653370721e-77	1.53522692161352	0.285	0.124	2.81970188954812e-72	Stromal Cell
ADARB1	9.98298719769479e-77	2.07305622586479	0.274	0.121	2.88707989757333e-72	Stromal Cell
KIAA1217	1.85787190268238e-76	1.29402700420857	0.732	0.647	5.37296554255745e-72	Stromal Cell
ARHGAP17	1.27707004743838e-74	1.9457162334538	0.373	0.208	3.6932865771918e-70	Stromal Cell
NOTCH2	1.79005711319097e-73	1.78454329537981	0.306	0.147	5.1768451713483e-69	Stromal Cell
EVA1C	3.01045835218335e-69	1.54413025012926	0.286	0.132	8.70624555451426e-65	Stromal Cell
LINC00882	6.98510882601202e-68	2.02586498726452	0.301	0.15	2.02009347248268e-63	Stromal Cell
ADAMTS9-AS2	1.0165126028811e-67	0.671179328039568	0.786	0.607	2.93975444753213e-63	Stromal Cell
SGIP1	1.26203466261931e-66	1.38917918907004	0.403	0.226	3.64980424429504e-62	Stromal Cell
ST5	7.09127631471401e-66	1.92905024220836	0.314	0.163	2.05079711021529e-61	Stromal Cell
FRY	8.4320155700337e-66	1.58111591481612	0.426	0.263	2.43853890285375e-61	Stromal Cell
CEP85L	2.49664412558799e-63	1.50452162565162	0.318	0.165	7.22029481120047e-59	Stromal Cell
PPP1R12B	1.28600583359077e-62	1.70892161372747	0.511	0.382	3.71912887074451e-58	Stromal Cell
LONRF1	2.61454466205942e-60	2.03462673836606	0.295	0.156	7.56126316267583e-56	Stromal Cell
ASAP1	1.62500947922653e-59	1.23912765786355	0.473	0.322	4.69952741392313e-55	Stromal Cell

gene	p_val	avg_log2FC	pct.1	pct.2	p_val_adj	cluster
ANGPT1	1.78188035199336e-59	1.72855335687732	0.26	0.123	5.1531979779648e-55	Stromal Cell
NBEAL1	2.93935251019767e-59	1.14762589807608	0.608	0.522	8.50060745949165e-55	Stromal Cell
SSBP3	6.99480683245472e-59	1.47706140617716	0.44	0.295	2.02289813594591e-54	Stromal Cell
ITGB1	4.98008399468574e-58	1.67428812393989	0.38	0.233	1.44024029126312e-53	Stromal Cell
ZFHX3	5.66756635061126e-58	2.27464301466389	0.443	0.319	1.63906018859678e-53	Stromal Cell
MBNL1	5.73573471885907e-58	0.863239402999735	0.691	0.608	1.65877448069404e-53	Stromal Cell
PTPRG	5.96200856084695e-57	0.886382704592855	0.783	0.742	1.72421287579694e-52	Stromal Cell
KIF13A	9.14867015913395e-57	1.26449832578023	0.458	0.314	2.64579541002154e-52	Stromal Cell
MTHFD1L	1.53003770764212e-56	1.96021995436943	0.291	0.155	4.42486905050102e-52	Stromal Cell
RERG	6.89882823870103e-56	1.38418714591294	0.403	0.247	1.99514112663234e-51	Stromal Cell
SYNE1	2.82066225037908e-55	0.817512085932831	0.686	0.589	8.1573552280963e-51	Stromal Cell
FOXO3	6.77417088410218e-55	1.42650746302998	0.552	0.424	1.95909021968235e-50	Stromal Cell
DGKH	3.18231111650317e-54	1.15764490891097	0.445	0.288	9.20324374892717e-50	Stromal Cell
USP53	3.85255314776216e-54	1.83873934849248	0.538	0.428	1.11415837033282e-49	Stromal Cell
GPC6	3.87378550903347e-54	1.5401660991018	0.534	0.406	1.12029876921248e-49	Stromal Cell
ARID5B	2.25553773804599e-53	1.042441211	0.566	0.431	6.52301513842901e-49	Stromal Cell
CDC42BPA	7.19348393460196e-53	0.888245117710758	0.638	0.556	2.08035555388689e-48	Stromal Cell
ETV6	8.95700628122989e-53	1.45775164775501	0.496	0.368	2.59036621653168e-48	Stromal Cell
MKLN1	4.08858712867979e-52	0.85289474127467	0.646	0.555	1.18241939761419e-47	Stromal Cell
DLEU2	5.05500856372211e-52	1.26514051012401	0.406	0.257	1.46190847662843e-47	Stromal Cell
TBC1D1	1.01318324455169e-51	1.47322922177286	0.57	0.491	2.9301259432435e-47	Stromal Cell
RASAL2	1.72059856634879e-51	1.13591559468035	0.53	0.405	4.9759710538807e-47	Stromal Cell
RBMS1	3.45085025305083e-51	1.13919281428639	0.57	0.459	9.979858931823e-47	Stromal Cell
DUSP1	4.86319558659985e-51	1.5791267765082	0.338	0.196	1.40643616364468e-46	Stromal Cell
NAV2	3.90702939440294e-50	1.25550819882502	0.508	0.372	1.12991290086133e-45	Stromal Cell
MAP4K4	6.11163370036574e-49	1.23334165782564	0.381	0.24	1.76748446614577e-44	Stromal Cell
ABHD2	1.21077826222203e-47	1.81024917130035	0.262	0.14	3.50157073434611e-43	Stromal Cell
MYH9	1.83451286133689e-47	1.20722918456179	0.459	0.323	5.30541119498628e-43	Stromal Cell
DPYD	1.84840213890648e-47	0.980569079113184	0.513	0.381	5.34557898571754e-43	Stromal Cell
MYO1E	3.41762443767207e-46	1.0490760709388	0.535	0.408	9.88376987374764e-42	Stromal Cell
PCDH9	5.07077026863165e-46	1.40916438993591	0.496	0.369	1.46646676168827e-41	Stromal Cell
PPP1CB	5.41112974410208e-46	1.46272881945525	0.38	0.25	1.56489872199432e-41	Stromal Cell
KLHL29	7.61922308377098e-46	1.2537635277094	0.258	0.132	2.20347931582657e-41	Stromal Cell
CACNA2D1	1.16418040323101e-45	1.21331962725818	0.437	0.302	3.36680972614408e-41	Stromal Cell
PICALM	1.6579346596566e-45	0.752819368209892	0.673	0.577	4.7947470357269e-41	Stromal Cell
TLE1	3.43801166694615e-45	1.48489663457216	0.266	0.143	9.94272974080828e-41	Stromal Cell
TTC28	4.63605180750302e-44	0.701453799660532	0.639	0.528	1.34074618272987e-39	Stromal Cell
CAMK2D	7.08937475428555e-44	1.14135192735919	0.439	0.311	2.05024717893938e-39	Stromal Cell
PTPN12	2.05837806430975e-43	1.32612195684515	0.36	0.233	5.95282936198381e-39	Stromal Cell
PPP1R12A	5.7269892553712e-43	1.19811111657869	0.524	0.439	1.65624529265335e-38	Stromal Cell
PIP4K2A	8.26158856683792e-41	0.953027719300219	0.402	0.271	2.38925141352953e-36	Stromal Cell
PEAK1	1.39403180541372e-40	1.22705858923685	0.397	0.275	4.03153998125648e-36	Stromal Cell

gene	p_val	avg_log2FC	pct.1	pct.2	p_val_adj	cluster
UBC	7.09627819432282e-40	2.18126149095879	0.279	0.164	2.05224365379816e-35	Stromal Cell
TRAF3IP2-AS1	1.63416615780127e-39	1.68609881591315	0.296	0.181	4.72600852836128e-35	Stromal Cell
ACTB	3.29479062752823e-39	1.32844276520993	0.397	0.273	9.52853449481165e-35	Stromal Cell
BMPR2	6.4639729560532e-38	0.91384561694742	0.527	0.42	1.86938097889058e-33	Stromal Cell
ELL2	7.45343083204473e-38	1.50593916825503	0.392	0.278	2.15553219662734e-33	Stromal Cell
CBLB	4.95219044776457e-37	0.887700927001025	0.523	0.406	1.43217347749351e-32	Stromal Cell
PBX1	5.42027046985454e-37	0.718287196486787	0.638	0.545	1.56754221988193e-32	Stromal Cell
UACA	1.17372573525672e-36	1.02798457553799	0.5	0.393	3.39441482636244e-32	Stromal Cell
DOCK4	1.33791732252465e-36	0.913059804288405	0.462	0.346	3.8692568967413e-32	Stromal Cell
FER	6.56469646268615e-36	0.896911947420581	0.538	0.442	1.89851021700883e-31	Stromal Cell
UBA2	4.90006683734015e-35	1.52090941549896	0.279	0.175	1.41709932935877e-30	Stromal Cell
GTDC1	6.16019301249613e-35	1.36678682275045	0.329	0.217	1.78152781921388e-30	Stromal Cell
SMURF1	1.50002904937139e-34	1.15361421361086	0.418	0.311	4.33808401078206e-30	Stromal Cell
KLF9	2.57988482882056e-34	1.39594123520925	0.299	0.19	7.46102692494907e-30	Stromal Cell
GAB2	3.87958226999801e-34	1.50815625762901	0.266	0.161	1.12197519248342e-29	Stromal Cell
ANKRD36C	1.23352118976829e-33	0.814704198021677	0.408	0.284	3.56734328080988e-29	Stromal Cell
NR3C1	1.67154814265204e-33	1.12210836112577	0.352	0.237	4.8341172285497e-29	Stromal Cell
AC098829.1	4.28805165584214e-33	1.24401473521334	0.258	0.15	1.24010453886955e-28	Stromal Cell
TRPC1	2.86329207856899e-32	1.2356421688793	0.279	0.172	8.28064069122153e-28	Stromal Cell
PIK3R1	9.57329404965276e-32	1.95852745669715	0.345	0.245	2.76859663915958e-27	Stromal Cell
TLE4	1.47115276909631e-31	1.22471965683084	0.254	0.151	4.25457380822652e-27	Stromal Cell
ARGLU1	3.75850480570724e-31	0.761104992475957	0.519	0.423	1.08695958981053e-26	Stromal Cell
HIP1	1.63750819193316e-30	0.556750485543455	0.573	0.454	4.7356736910707e-26	Stromal Cell
ELOVL5	1.8292523533817e-30	1.22864307999653	0.264	0.162	5.29019780597987e-26	Stromal Cell
PTPN9	7.42759422958023e-30	1.38017005874945	0.301	0.2	2.1480602511946e-25	Stromal Cell
TNS1	3.37100980455371e-29	0.686467155991576	0.594	0.512	9.74896035476933e-25	Stromal Cell
ATP10D	4.24205638996185e-29	1.39459629382809	0.334	0.236	1.22680270797697e-24	Stromal Cell
CACNB2	4.66248390317629e-29	1.26044908368116	0.345	0.24	1.34839034479858e-24	Stromal Cell
DSTN	5.56956467739392e-28	1.38899936226477	0.299	0.203	1.61071810470232e-23	Stromal Cell
STAG1	1.19122637874524e-27	0.591636481102448	0.663	0.603	3.44502668733123e-23	Stromal Cell
JAK1	1.38519041305248e-27	0.647061226065078	0.589	0.513	4.00597067454777e-23	Stromal Cell
ARHGEF7	2.20586333219965e-27	1.32973480182865	0.451	0.38	6.37935675672138e-23	Stromal Cell
CREB3L2	2.2475163853734e-27	1.19911803297557	0.346	0.247	6.49981738649986e-23	Stromal Cell
SETBP1	2.93802966442786e-27	0.682480065436198	0.7	0.655	8.49678178952537e-23	Stromal Cell
SDK1	5.13596142674552e-27	0.898865195975429	0.497	0.407	1.48532004461481e-22	Stromal Cell
FKBP5	2.89705049892237e-26	0.496349590624656	0.65	0.526	8.37827004288349e-22	Stromal Cell
PARVA	3.67568295129586e-26	1.14559320937145	0.338	0.245	1.06300750951476e-21	Stromal Cell
MYH10	1.06714644590508e-25	1.27140569448323	0.39	0.307	3.08618752155748e-21	Stromal Cell
REV3L	1.12176187923052e-25	0.957178723193023	0.486	0.415	3.24413535473467e-21	Stromal Cell
RBFOX2	1.31999636591295e-25	0.756947318644395	0.508	0.437	3.81742949022026e-21	Stromal Cell
HIVEP3	1.86077689473853e-25	1.02128788018122	0.275	0.179	5.38136677958384e-21	Stromal Cell
IL6ST	1.31791993168847e-24	0.894593663670778	0.381	0.283	3.81142444244304e-20	Stromal Cell

gene	p_val	avg_log2FC	pct.1	pct.2	p_val_adj	cluster
CPSF6	1.34239403796251e-24	1.02772014174763	0.363	0.275	3.88220355778758e-20	Stromal Cell
SEMA5A	6.39660924431921e-24	1.06765719689201	0.329	0.232	1.84989939345712e-19	Stromal Cell
TBX2	8.05584137606128e-24	1.26764812312512	0.26	0.169	2.32974932595692e-19	Stromal Cell
ANO6	1.21392373647201e-23	0.980559262485764	0.378	0.291	3.51066744587706e-19	Stromal Cell
GHR	1.47278247646641e-23	0.851134544977804	0.451	0.359	4.25928692194087e-19	Stromal Cell
CD46	1.52944538750838e-23	1.14006114104152	0.369	0.288	4.42315606067425e-19	Stromal Cell
PLS3	1.98714727003265e-23	1.12194441002141	0.319	0.229	5.74682990493442e-19	Stromal Cell
MYO1B	2.08958110225707e-23	0.669592547500894	0.346	0.241	6.04306854772744e-19	Stromal Cell
SH3BGRL	2.93434512415278e-23	0.939506057129944	0.343	0.249	8.48612609904984e-19	Stromal Cell
PITPNC1	6.16698545227381e-23	0.875378035274084	0.416	0.33	1.78349219279759e-18	Stromal Cell
ANKRD44	6.33011796955682e-23	0.638619459636962	0.285	0.188	1.83067011679583e-18	Stromal Cell
NBEA	8.853128838084e-23	0.967283157382259	0.513	0.458	2.56032485997389e-18	Stromal Cell
EXT1	1.27207852656505e-22	0.944384590918566	0.565	0.533	3.67885109882614e-18	Stromal Cell
TJP1	2.23890156224969e-22	0.883277550468235	0.459	0.382	6.47490331802609e-18	Stromal Cell
ESYT2	2.4825984779584e-22	1.1279717083015	0.409	0.33	7.17967479825569e-18	Stromal Cell
LAMC1	3.37287713119555e-22	1.31033747933203	0.267	0.185	9.75436066341752e-18	Stromal Cell
PLEKHA2	3.98629240682955e-22	1.01388343321765	0.282	0.193	1.15283576405511e-17	Stromal Cell
FNDC3B	4.21165485464418e-22	0.525048910584979	0.686	0.627	1.2180105839631e-17	Stromal Cell
CERS6	1.75701647068926e-21	0.982171330628729	0.303	0.217	5.08129163323333e-17	Stromal Cell
MRTFA	6.89474343007739e-21	0.956622655750317	0.468	0.406	1.99395979997838e-16	Stromal Cell
NAP1L1	1.12036239948935e-20	1.0687053164072	0.303	0.221	3.24008805932321e-16	Stromal Cell
MICU3	1.81191109531435e-20	1.22032545581285	0.268	0.19	5.2400468876491e-16	Stromal Cell
FTO	2.18197441937782e-20	0.684088414354529	0.54	0.487	6.31027002084066e-16	Stromal Cell
INPP5A	2.83374290946902e-20	1.07521125477889	0.317	0.237	8.19518449418441e-16	Stromal Cell
SEPTIN7	3.45537234197061e-20	0.972143637302277	0.342	0.265	9.992936812979e-16	Stromal Cell
GSN	8.00982902575672e-20	1.30159004441332	0.251	0.172	2.31644255424884e-15	Stromal Cell
MED13L	1.27712818142552e-19	0.389171162803645	0.702	0.651	3.69345470068261e-15	Stromal Cell
RSU1	2.2035425259155e-19	1.05914887395455	0.287	0.205	6.37264498494764e-15	Stromal Cell
JAK2	4.10670576321579e-19	1.4211910088138	0.35	0.282	1.18765930672201e-14	Stromal Cell
MSN	6.35978144415078e-19	1.00991378721642	0.258	0.179	1.83924879364841e-14	Stromal Cell
PLD1	9.43330928739245e-19	1.32543029457355	0.251	0.178	2.7281130459139e-14	Stromal Cell
VMP1	1.36332916130775e-18	1.05130353777458	0.462	0.412	3.94274793450201e-14	Stromal Cell
SPRED1	1.50938986219396e-18	0.890412268192575	0.302	0.223	4.36515548146494e-14	Stromal Cell
PITPNB	1.61261984095865e-18	1.15020822879688	0.307	0.234	4.66369658005242e-14	Stromal Cell
LINC00342	2.90253907470143e-18	0.630792678619897	0.368	0.278	8.39414300403653e-14	Stromal Cell
ANKRD36	4.01230670046258e-18	0.640575182527923	0.43	0.352	1.16035909777378e-13	Stromal Cell
DAAM1	6.97512959870767e-18	1.27748693799207	0.346	0.285	2.01720747994626e-13	Stromal Cell
KLF7	8.88872960869833e-18	0.935527711548666	0.255	0.175	2.57062060283556e-13	Stromal Cell
CYP7B1	1.02327707336244e-17	1.63984786583924	0.252	0.184	2.95931729616417e-13	Stromal Cell
PDS5B	1.18713589895488e-17	0.882571487410593	0.4	0.336	3.43319701977752e-13	Stromal Cell
PHTF2	4.18361380925971e-17	1.48486546401617	0.257	0.191	1.20990111363791e-12	Stromal Cell
TANC1	1.31293415123106e-16	1.01674855961472	0.425	0.368	3.79700556536021e-12	Stromal Cell

gene	p_val	avg_log2FC	pct.1	pct.2	p_val_adj	cluster
MPDZ	2.62857315128972e-16	0.856138203856077	0.348	0.279	7.60183355352987e-12	Stromal Cell
RAP1B	5.48406310498291e-16	0.774415855804574	0.377	0.314	1.58599104996106e-11	Stromal Cell
TCF12	1.16594638859118e-15	0.425938055785437	0.675	0.654	3.37191695580568e-11	Stromal Cell
PER1	2.02274183410038e-15	0.936867670579175	0.313	0.243	5.84976938421831e-11	Stromal Cell
MID1	1.47826820332473e-14	1.08940681651852	0.252	0.185	4.27515164401512e-10	Stromal Cell
PARD3B	2.29254365121383e-14	0.356857140905634	0.719	0.686	6.63003623931039e-10	Stromal Cell
TBL1X	2.96479480677815e-14	0.707870170903353	0.503	0.472	8.5741865812024e-10	Stromal Cell
MYCBP2	1.0281565851039e-13	0.601595808858445	0.5	0.461	2.97342884412049e-09	Stromal Cell
YAP1	1.22561210194805e-13	0.787253859914181	0.45	0.407	3.54447019883377e-09	Stromal Cell
AASS	1.59405800704008e-13	0.609162733450672	0.365	0.293	4.61001575635992e-09	Stromal Cell
DOCK8	2.14237337847654e-13	0.88746141928349	0.28	0.212	6.19574381055415e-09	Stromal Cell
ZFP36L1	2.15267154178376e-13	1.19732253808011	0.296	0.236	6.22552609883864e-09	Stromal Cell
ARHGAP26	2.64634697249938e-13	0.656865569454215	0.388	0.323	7.65323544446821e-09	Stromal Cell
STAT3	3.93008075971354e-13	0.733225870727118	0.46	0.42	1.13657935570916e-08	Stromal Cell
PALM2-AKAP2	5.58333698056482e-13	0.728466283486225	0.367	0.31	1.61470105477935e-08	Stromal Cell
SPRED2	7.73580409202933e-13	0.906182975231705	0.338	0.283	2.23719454341488e-08	Stromal Cell
PDZRN3	8.37736783423359e-13	1.01289395453488	0.395	0.342	2.42273477766035e-08	Stromal Cell
FOXN3	8.57428884212148e-13	0.56381258576543	0.463	0.417	2.47968433314153e-08	Stromal Cell
AKAP13	1.1710437458506e-12	0.519100255825532	0.609	0.592	3.38665851299993e-08	Stromal Cell
GULP1	1.89393561191733e-12	0.638726838682909	0.438	0.383	5.47726178966492e-08	Stromal Cell
SENP6	2.29079960144642e-12	0.473923492657819	0.549	0.522	6.62499244738306e-08	Stromal Cell
PCGF5	2.79656132722984e-12	0.715010196941091	0.381	0.324	8.0876553583487e-08	Stromal Cell
RAPGEF2	3.29219382669365e-12	0.690239533929178	0.5	0.477	9.52102454679803e-08	Stromal Cell
CCNH	3.32404816361147e-12	0.767933149934638	0.427	0.389	9.61314728916439e-08	Stromal Cell
MBNL2	4.15588874595003e-12	0.697666454050282	0.497	0.477	1.20188302532875e-07	Stromal Cell
PBX3	4.22276468419775e-12	0.84264691289929	0.279	0.215	1.22122354666999e-07	Stromal Cell
SPTBN1	5.11037500341507e-12	0.522002042082981	0.516	0.486	1.47792045098764e-07	Stromal Cell
HIVEP2	5.92733071648356e-12	0.634357586741872	0.422	0.371	1.71418404320705e-07	Stromal Cell
PHF14	8.16191916944632e-12	0.635605514013479	0.387	0.337	2.36042702380388e-07	Stromal Cell
HERC1	9.15003990099868e-12	0.479740957457572	0.551	0.529	2.64619153936882e-07	Stromal Cell
FTX	1.48216707177308e-11	0.276438097555626	0.866	0.867	4.28642717156774e-07	Stromal Cell
ZFAND5	2.03100123388171e-11	1.27108896459906	0.371	0.335	5.87365556838591e-07	Stromal Cell
PTPRD	2.06365260886748e-11	0.353459162229374	0.419	0.333	5.96808334484476e-07	Stromal Cell
GNAS	2.07742936708534e-11	1.53868633525411	0.325	0.283	6.00792572961079e-07	Stromal Cell
UGGT2	2.354502932143e-11	0.633007833861842	0.448	0.413	6.80922247975754e-07	Stromal Cell
CAMSAP2	2.80036495329902e-11	0.768152603959597	0.341	0.291	8.09865544494076e-07	Stromal Cell
SESN1	2.96689499498059e-11	0.92730422410586	0.277	0.224	8.58026032548387e-07	Stromal Cell
INPP4A	2.98149512857258e-11	0.935982814014133	0.257	0.2	8.62248391183191e-07	Stromal Cell
TNRC6C	5.65380392437204e-11	0.794546866606262	0.298	0.247	1.63508009492839e-06	Stromal Cell
PAWR	8.46846423737008e-11	0.718004287271141	0.385	0.341	2.44907985744743e-06	Stromal Cell
PLCL1	1.12038863136483e-10	0.784128389502094	0.694	0.719	3.24016392190709e-06	Stromal Cell
UBR3	1.69190936619508e-10	0.459752732546408	0.521	0.493	4.89300188703618e-06	Stromal Cell

gene	p_val	avg_log2FC	pct.1	pct.2	p_val_adj	cluster
SAMD4A	1.96225442579931e-10	1.06080177026743	0.357	0.307	5.6748397994116e-06	Stromal Cell
BMPR1A	2.73698962729699e-10	0.810765778276415	0.357	0.318	7.91537400214289e-06	Stromal Cell
ZNF532	3.05992366666141e-10	0.898891432050163	0.284	0.233	8.84929924398479e-06	Stromal Cell
RTN4	4.59221647448053e-10	0.566272004260298	0.415	0.369	1.32806900441977e-05	Stromal Cell
ACTN4	4.96684848269377e-10	0.610205595596505	0.458	0.427	1.43641258119504e-05	Stromal Cell
STIM1	6.92769195556217e-10	0.613280439413257	0.255	0.197	2.00348851354858e-05	Stromal Cell
SBF2	7.44663933610245e-10	0.33852083188772	0.683	0.682	2.15356809600083e-05	Stromal Cell
ASCC3	7.7319983282307e-10	0.599937290744979	0.387	0.344	2.23609391652432e-05	Stromal Cell
ALG13	8.40926615848749e-10	0.770220561112307	0.254	0.202	2.43195977303458e-05	Stromal Cell
LRCH3	9.27195191413413e-10	0.804313340010062	0.343	0.302	2.68144849356759e-05	Stromal Cell
UBR1	9.63272752503615e-10	0.777454856611038	0.36	0.321	2.78578480024045e-05	Stromal Cell
RORA	1.02713068935190e-09	0.307294586674033	0.804	0.812	2.97046195360568e-05	Stromal Cell
EGFR	1.21514977441058e-09	0.728731847202908	0.387	0.343	3.5142131475954e-05	Stromal Cell
PJA2	1.62085445166834e-09	0.69446677217241	0.295	0.247	4.68751107422483e-05	Stromal Cell
ACTR3	3.33257008693001e-09	0.767519221900388	0.3	0.254	9.63779269140158e-05	Stromal Cell
FAM214A	3.65528632616257e-09	0.829492208631947	0.343	0.305	0.000105710880552622	Stromal Cell
PIK3CA	3.95416745878486e-09	0.982108538624979	0.251	0.204	0.000114354522908058	Stromal Cell
LINC01266	5.31422039052117e-09	0.790713744277832	0.267	0.216	0.000153687253693872	Stromal Cell
NCOA1	5.73915328871087e-09	0.444331598989126	0.573	0.562	0.000165976313109519	Stromal Cell
CCND3	6.22289496315343e-09	0.566684501741954	0.275	0.221	0.000179966122334397	Stromal Cell
KMT2E	7.1635345798947e-09	0.498434526338958	0.527	0.516	0.000207169420050555	Stromal Cell
PAM	8.8712959749788e-09	0.561996015620595	0.453	0.426	0.000256557879596387	Stromal Cell
SPECC1L	9.17204439916977e-09	0.55884439089491	0.395	0.355	0.00026525552402399	Stromal Cell
KLF12	1.15306080108069e-08	0.540208384974325	0.492	0.47	0.000333465183672535	Stromal Cell
UBR5	1.36223438292135e-08	0.468021006411172	0.5	0.486	0.000393958183540854	Stromal Cell
NCOR2	1.77601983924219e-08	0.76160064308182	0.334	0.297	0.000513624937508842	Stromal Cell
FRYL	2.55967285127709e-08	0.537070142759239	0.465	0.447	0.000740257388589334	Stromal Cell
PTBP2	2.69967149660301e-08	0.660460222744392	0.389	0.358	0.00078074499681759	Stromal Cell
IQGAP1	2.78250961328495e-08	0.654642243239572	0.336	0.298	0.000804701780162009	Stromal Cell
FUS	4.10398449905519e-08	0.640378179687078	0.368	0.327	0.00118687231712676	Stromal Cell
MIR99AHG	4.61780312807848e-08	1.00391690575958	0.477	0.488	0.0013354686646403	Stromal Cell
MYOF	5.22793975561451e-08	0.969407236287795	0.255	0.213	0.00151192017732372	Stromal Cell
TMEM165	7.68462996536932e-08	0.735713566049059	0.298	0.26	0.00222239498598481	Stromal Cell
SMC5	7.77748491108981e-08	0.711977952422831	0.349	0.317	0.00224924863628717	Stromal Cell
CDC27	1.37118841354806e-07	0.722590495927869	0.26	0.219	0.00396547689198098	Stromal Cell
STT3B	1.60517682229481e-07	0.798121972935834	0.306	0.27	0.0046421713700766	Stromal Cell
CHIC2	1.94229398176987e-07	1.00667615326708	0.255	0.22	0.00561711419527845	Stromal Cell
TGFBR3	3.38257840032887e-07	0.425772140959892	0.256	0.205	0.00978241673375109	Stromal Cell
DLG2	3.52314111985251e-07	0.253928568557524	0.675	0.62	0.0101889241186135	Stromal Cell
RABGAP1	4.23961970567786e-07	0.596716278449315	0.42	0.406	0.0122609801888204	Stromal Cell
PTPRK	4.56265533842376e-07	0.699914182325743	0.492	0.504	0.0131951992387215	Stromal Cell
SH3D19	4.71795471405355e-07	0.75287214412838	0.4	0.382	0.0136443250330429	Stromal Cell

gene	p_val	avg_log2FC	pct.1	pct.2	p_val_adj	cluster
EHBP1	5.41388856354056e-07	0.609342081982402	0.55	0.56	0.0156569657257593	Stromal Cell
ST3GAL3	1.36523935905199e-06	0.734365074622197	0.252	0.216	0.0394827222637835	Stromal Cell
ZNF280D	1.49226967705798e-06	0.509244676834971	0.344	0.313	0.0431564390605169	Stromal Cell
BCKDHB	1.72845058888718e-06	0.306488211520042	0.3	0.4	0.0499867910306173	Stromal Cell
PPARG	1.74964378594962e-06	1.09029189593779	0.299	0.271	0.050599698289663	Stromal Cell
CSNK1A1	2.55456384057295e-06	0.50014054701082	0.489	0.493	0.0738779862693696	Stromal Cell
ANKRD10	3.1025397681363e-06	0.613481674022589	0.349	0.324	0.0897254500945018	Stromal Cell
FNBP1	3.79743085767177e-06	0.431298652539219	0.326	0.289	0.109821700403867	Stromal Cell
MIR100HG	4.79113373378485e-06	0.557974034845797	0.327	0.28	0.138559587581058	Stromal Cell
AOPEP	5.56579433862919e-06	0.320114145468294	0.619	0.623	0.160962772273156	Stromal Cell
CAPZB	5.63744586703916e-06	0.581202508316927	0.368	0.347	0.163034934474773	Stromal Cell
ADAMTS9	5.65670757902643e-06	0.330406370738002	0.256	0.342	0.163591983185444	Stromal Cell
AHNAK	6.31790462062997e-06	0.584774009349061	0.274	0.236	0.182713801628619	Stromal Cell
EPN2	8.69172720011701e-06	0.674575306002099	0.317	0.289	0.251364750627384	Stromal Cell
FERMT2	1.23297377419136e-05	0.572986096558372	0.38	0.356	0.356576015496142	Stromal Cell
HIPK3	1.27667966313148e-05	0.631291279890187	0.373	0.354	0.369215758577625	Stromal Cell
STARD13	1.28839607210425e-05	0.379720698462714	0.439	0.412	0.372604144052548	Stromal Cell
CTNNB1	1.29518869465601e-05	0.71811190359783	0.268	0.237	0.374568570494519	Stromal Cell
UVRAG	1.37772685729255e-05	0.541071905415956	0.417	0.405	0.398438607129005	Stromal Cell
KLC1	1.52871492542013e-05	0.533990586315355	0.364	0.343	0.442104356431501	Stromal Cell
SP100	1.84909754926258e-05	0.474361520658162	0.311	0.281	0.534759011246739	Stromal Cell
VPS13B	2.25688376178996e-05	0.274683000126382	0.605	0.607	0.652690783909658	Stromal Cell
MT-CYB	2.26436192047888e-05	0.537461852170792	0.34	0.311	0.654853467402493	Stromal Cell
ACYP2	2.38823303610567e-05	0.497066592248871	0.427	0.418	0.690676994041761	Stromal Cell
WAC	2.60670520296223e-05	0.369938674694164	0.461	0.452	0.753859144696676	Stromal Cell
VPS45	2.74219725388317e-05	0.671401871540937	0.271	0.243	0.793043445823013	Stromal Cell
EPC1	2.94357729223472e-05	0.484631330863315	0.398	0.381	0.85128255291428	Stromal Cell
PPP6R3	3.16892350505348e-05	0.353045195209563	0.532	0.529	0.916452677661467	Stromal Cell
TANK	3.27094876805218e-05	0.858198276521168	0.282	0.258	0.945958383720691	Stromal Cell
RIC1	3.59961873841709e-05	0.65245758949256	0.321	0.3	1	Stromal Cell
CLMN	4.41516530807502e-05	0.765802655235505	0.491	0.524	1	Stromal Cell
LENG8	4.50928962158553e-05	0.65117417805099	0.306	0.28	1	Stromal Cell
PHF21A	4.68129314013585e-05	0.533519369147405	0.369	0.354	1	Stromal Cell
CEP112	4.82697106225653e-05	0.503915251392049	0.356	0.331	1	Stromal Cell
PARP14	5.73672006107972e-05	0.419417010259587	0.274	0.24	1	Stromal Cell
RB1	6.06391856272983e-05	0.435826888680364	0.388	0.368	1	Stromal Cell
ITSN1	6.12327557667141e-05	0.691558461433095	0.4	0.398	1	Stromal Cell
OSBPL8	6.37737517271755e-05	0.854336912291451	0.403	0.397	1	Stromal Cell
TRPM3	6.88869286980962e-05	0.292832112346362	0.46	0.547	1	Stromal Cell
PKD2	7.11567843804497e-05	0.638749845816828	0.371	0.362	1	Stromal Cell
ATP2A2	7.63768552351642e-05	0.739733899674928	0.288	0.264	1	Stromal Cell
ARID4B	0.000100446304154167	0.354710714233671	0.495	0.493	1	Stromal Cell

gene	p_val	avg_log2FC	pct.1	pct.2	p_val_adj	cluster
ZDHHC14	0.00010768585389796	0.464561123905784	0.36	0.329	1	Stromal Cell
KAT6B	0.000122930601020134	0.48999780976273	0.416	0.409	1	Stromal Cell
TCF7L2	0.000135379968640649	0.49226171196378	0.434	0.428	1	Stromal Cell
KANSL1	0.000170406307041942	0.261457368984891	0.621	0.634	1	Stromal Cell
NEK7	0.000183097806731787	0.610551779979056	0.363	0.352	1	Stromal Cell
GAB1	0.000199264600090393	0.601502848694197	0.373	0.36	1	Stromal Cell
HNRNPU	0.000214802471816639	0.557933271479739	0.43	0.43	1	Stromal Cell
LARGE1	0.000242357839278604	0.370767139524552	0.425	0.416	1	Stromal Cell
ZSWIM6	0.000266689866228901	0.266370431013359	0.562	0.561	1	Stromal Cell
TRIO	0.000278222694903641	0.394204947657705	0.534	0.543	1	Stromal Cell
CTBP2	0.000309761338822471	0.470112305761385	0.476	0.474	1	Stromal Cell
SMCHD1	0.000342886924673015	0.392571624741221	0.408	0.401	1	Stromal Cell
EPS15	0.000344705915360004	0.40124589050649	0.423	0.421	1	Stromal Cell
ARHGAP21	0.000399673367327626	0.638785620864399	0.324	0.311	1	Stromal Cell
RAP1A	0.000442318868509386	0.450924757448777	0.327	0.31	1	Stromal Cell
MAP4	0.000505646052673628	0.313608572133665	0.525	0.529	1	Stromal Cell
MT-ND5	0.00056385454112233	0.375051365040038	0.331	0.307	1	Stromal Cell
HIF1A	0.000664450600503261	0.510353751569156	0.369	0.36	1	Stromal Cell
STXBP5	0.000677281064426897	0.483465493714229	0.336	0.322	1	Stromal Cell
KIAA1328	0.000703792031919978	0.275807160244731	0.403	0.387	1	Stromal Cell
CLASP2	0.000721130549936968	0.454105796465164	0.414	0.417	1	Stromal Cell
RNF180	0.000723639103730419	0.546219823834133	0.33	0.317	1	Stromal Cell
ATF7	0.000773207089713509	0.438653276234843	0.261	0.236	1	Stromal Cell
NEO1	0.000877882199846102	0.696325982373511	0.315	0.301	1	Stromal Cell
METTL15	0.000967341960873613	0.51334114096035	0.401	0.399	1	Stromal Cell
PPP3CC	0.00105099228307637	0.499773347255025	0.284	0.267	1	Stromal Cell
ZRANB2	0.00123549268366816	0.320094915081376	0.495	0.5	1	Stromal Cell
KDM6A	0.00128876332452767	0.383382685258868	0.446	0.451	1	Stromal Cell
FLNB	0.00144322201771958	0.472111789441214	0.264	0.335	1	Stromal Cell
POU2F1	0.00149723769880051	0.425143681393144	0.358	0.343	1	Stromal Cell
PSPC1	0.00169025457809654	0.471237816508108	0.351	0.345	1	Stromal Cell
TBL1XR1	0.00224769456311409	0.388590645745517	0.449	0.454	1	Stromal Cell
MT-ND4	0.0025466410865468	0.457493125398781	0.377	0.366	1	Stromal Cell
NUP98	0.00278692549359449	0.550234408021182	0.298	0.286	1	Stromal Cell
EEF1A1	0.00283884525954733	0.409856726317646	0.287	0.266	1	Stromal Cell
DYRK1A	0.00337596925542879	0.303680423162868	0.498	0.509	1	Stromal Cell
SETD5	0.00453890060835865	0.330612087568547	0.424	0.43	1	Stromal Cell
RNF115	0.0045578110932801	0.516448706861343	0.347	0.343	1	Stromal Cell
TNKS	0.00461343039108146	0.320248425678264	0.479	0.492	1	Stromal Cell
MAP4K5	0.00465905098892725	0.433890123261486	0.297	0.285	1	Stromal Cell
FBXO11	0.00493529639634723	0.41193149753769	0.402	0.406	1	Stromal Cell
MON2	0.00614318254370162	0.273283781218677	0.512	0.526	1	Stromal Cell

gene	p_val	avg_log2FC	pct.1	pct.2	p_val_adj	cluster
AEBP2	0.00728556431336587	0.438987919820649	0.437	0.442	1	Stromal Cell
SRGAP1	0.00758804753193708	0.358554506173313	0.295	0.278	1	Stromal Cell
GNB1	0.00775380305738473	0.350755848681099	0.387	0.387	1	Stromal Cell
NCKAP1	0.00885051117114638	0.422825437901873	0.349	0.347	1	Stromal Cell
PAFAH1B1	0.0089826074274962	0.289160647413184	0.47	0.483	1	Stromal Cell
TSC22D1	0.00953351016998968	0.338508333441032	0.442	0.439	1	Stromal Cell

Supplementary Table S2. Sensitivity of nuclei retention and OXR1 expression to mitochondrial QC thresholds in GSE195460.

Cohort-level nuclei retention

Group	Libraries (n)	Nuclei after gene filter	Retained at MT <5%	Retained at MT <10%	Additional at 10%	Excluded at 5% (%)	Excluded at 10% (%)
Control	6	38,926	38,777	38,863	86	0.38	0.16
DKD	5	24,383	23,015	23,768	753	5.61	2.52
Overall	11	63,309	61,792	62,631	839	2.40	1.07

Individual-library nuclei retention

Library	Group	Nuclei after gene filter	Retained at MT <5%	Retained at MT <10%	Additional at 10%	Excluded at 5% (%)	Excluded at 10% (%)
Control1	Control	6,118	6,086	6,115	29	0.52	0.05
Control2	Control	4,316	4,218	4,262	44	2.27	1.25
Control3	Control	6,537	6,537	6,537	0	0.00	0.00
Control4	Control	4,659	4,657	4,659	2	0.04	0.00
Control5	Control	4,969	4,969	4,969	0	0.00	0.00
Control6	Control	12,327	12,310	12,321	11	0.14	0.05
DN1	DKD	4,942	4,938	4,942	4	0.08	0.00
DN2	DKD	3,697	3,692	3,695	3	0.14	0.05
DN3	DKD	3,690	3,675	3,686	11	0.41	0.11
DN4	DKD	6,481	6,009	6,237	228	7.28	3.76
DN5	DKD	5,573	4,701	5,208	507	15.65	6.55

OXR1 expression sensitivity comparison

MT cutoff	Control mean expression	DKD mean expression	log2FC (DKD vs Control)	P value	Direction	Independent-donor test
<5%	1.14	0.69	-0.724	0.021	Lower in DKD	Not performed
<10%	1.13	0.70	-0.691	0.028	Lower in DKD	Not performed

Notes: MT denotes the percentage of mitochondrial transcripts per nucleus. Additional at 10% = nuclei with 5% ≤ MT <10%; Removed at ≥5%, % = 100 × (after gene filter − retained at MT <5%)/(after gene filter)

Supplementary Table S3. Significantly differentially expressed genes in DKD podocytes identified by exploratory cell-level analysis.

Gene_Symbol	p_val	avg_log2FC	pct.1	pct.2	p_val_adj	Significance
MT-CO2	2.26051935282773e-43	1.52873837694816	0.726	0.518	6.53742196837779e-39	Up-regulated in DKD
LINC02740	1.95548783102729e-41	3.04188889659262	0.231	0.036	5.65527080733091e-37	Up-regulated in DKD
THSD7A	1.91639654597751e-40	0.768097847149128	0.847	0.781	5.54221881096696e-36	Up-regulated in DKD
ST6GALNAC3	4.12223370179812e-40	0.740291474540733	0.815	0.742	1.19214998656002e-35	Up-regulated in DKD
PLA2R1	6.71674762245569e-40	0.725349609380157	0.824	0.748	1.94248341241418e-35	Up-regulated in DKD
LINC02745	6.68257941842618e-37	2.83885976992153	0.226	0.041	1.93260196780885e-32	Up-regulated in DKD
CNTN4	9.05110971648689e-37	1.29403041596515	0.579	0.348	2.61758093000801e-32	Up-regulated in DKD
PARD3B	9.68929235853493e-35	0.566039214005419	0.855	0.817	2.8021433500883e-30	Up-regulated in DKD
DPP4	5.00330131362826e-34	2.72773415804327	0.217	0.042	1.44695473990129e-29	Up-regulated in DKD
SRGAP2B	5.88107180636914e-34	0.92454576123019	0.719	0.548	1.70080596640196e-29	Up-regulated in DKD
NEBL	7.64120969347446e-33	0.605387340430874	0.857	0.772	2.20983784335281e-28	Up-regulated in DKD
IQCJ-SCHIP1	1.36302921422399e-32	0.876463786393155	0.761	0.577	3.94188048753579e-28	Up-regulated in DKD
NPNT	2.24744326131739e-32	0.833921241359222	0.746	0.638	6.49960591172989e-28	Up-regulated in DKD
SPOCK2	2.59103304641797e-31	1.30316374558151	0.533	0.324	7.49326757024076e-27	Up-regulated in DKD
UNC5D	4.83996649795033e-31	2.10832212351589	0.412	0.214	1.39971831120723e-26	Up-regulated in DKD
CRIM1	5.47975389151695e-31	0.803623397394533	0.793	0.69	1.5847448254267e-26	Up-regulated in DKD
ST3GAL6	1.44516873173269e-30	0.653911760575185	0.777	0.649	4.17942797217093e-26	Up-regulated in DKD
BGN	3.25101789917033e-30	1.57643853859441	0.206	0.044	9.40194376440058e-26	Up-regulated in DKD
MGAT5	7.52179966769773e-30	0.780321949002424	0.778	0.642	2.17530446389818e-25	Up-regulated in DKD
ARRDC4	2.11740115018999e-29	1.28407681413738	0.593	0.389	6.12352412634945e-25	Up-regulated in DKD
SRGAP2	2.66948589014107e-29	0.734653024182378	0.749	0.624	7.72015319428797e-25	Up-regulated in DKD
SGIP1	1.13450140635926e-28	0.852436744608877	0.726	0.572	3.28097806719099e-24	Up-regulated in DKD
CBLB	3.38568763723827e-28	0.645103287012325	0.786	0.711	9.79140864689308e-24	Up-regulated in DKD
CDC14A	2.0006175386856e-27	0.680139596565857	0.774	0.633	5.78578592187875e-23	Up-regulated in DKD
PTPRD	7.05394834424745e-27	0.54874272200419	0.804	0.72	2.04000186115636e-22	Up-regulated in DKD
ADAMTS9-AS2	8.08006766408771e-27	0.798995269153142	0.822	0.712	2.33675556845416e-22	Up-regulated in DKD
NTNG1	2.74822599451977e-26	0.650842305697093	0.791	0.667	7.94786957615119e-22	Up-regulated in DKD
MT-ND4L	8.38774565825654e-26	0.544863022922503	0.428	0.199	2.42573604436779e-21	Up-regulated in DKD
DST	1.23570171801112e-25	0.483941133490627	0.864	0.769	3.57364936848817e-21	Up-regulated in DKD
DACH1	8.51578152125914e-25	0.604612316934672	0.82	0.761	2.46276401594814e-20	Up-regulated in DKD
PLCE1	2.08428201054658e-24	0.555360201911511	0.795	0.694	6.0277435745007e-20	Up-regulated in DKD
CLIP4	2.32468138815011e-24	0.783725868914874	0.685	0.518	6.72297857453012e-20	Up-regulated in DKD
CPNE4	3.07561795666498e-24	1.34441875290813	0.349	0.165	8.89468713067511e-20	Up-regulated in DKD
KLF7	5.74423380803746e-24	0.794153784431268	0.687	0.532	1.66123241728443e-19	Up-regulated in DKD
SNX25	1.23938454760801e-23	0.775104344208645	0.701	0.545	3.58430011168238e-19	Up-regulated in DKD
NPAS3	5.15437347369982e-23	0.516366227962305	0.805	0.708	1.49064480859399e-18	Up-regulated in DKD
CD47	1.01671698973588e-22	0.917068951080464	0.504	0.309	2.94034553431615e-18	Up-regulated in DKD
COL4A3	1.36336021567932e-22	0.468451337972142	0.825	0.732	3.94283774374459e-18	Up-regulated in DKD
CR1	2.65206264749142e-22	0.737086617069411	0.712	0.568	7.66976517654517e-18	Up-regulated in DKD
FMN2	2.55898918194027e-21	0.561850677710647	0.761	0.646	7.40059671417127e-17	Up-regulated in DKD

Gene_Symbol	p_val	avg_log2FC	pct.1	pct.2	p_val_adj	Significance
PODXL	4.75141903487219e-21	0.680078215344993	0.768	0.692	1.37411038488504e-16	Up-regulated in DKD
MAGI2-AS3	8.24806154668252e-21	0.505560185235293	0.77	0.669	2.38533939930059e-16	Up-regulated in DKD
DACH2	9.23686360122594e-21	0.826310743676118	0.659	0.521	2.67130095347454e-16	Up-regulated in DKD
FRY	3.92911294154065e-20	0.564565340167312	0.782	0.669	1.13629946269356e-15	Up-regulated in DKD
TMEM150C	4.04345808126564e-20	1.02671995342523	0.531	0.357	1.16936807710202e-15	Up-regulated in DKD
UNC5C	4.57984387914789e-20	0.760984864714341	0.698	0.575	1.32449084984957e-15	Up-regulated in DKD
TPPP3	7.5463830713322e-20	0.85959833294551	0.207	0.069	2.18241398422927e-15	Up-regulated in DKD
TARID	7.97690109958704e-20	0.676209269754005	0.73	0.59	2.30691979800057e-15	Up-regulated in DKD
SRGAP2C	8.04293634082314e-20	0.653280278790084	0.708	0.562	2.32601718976605e-15	Up-regulated in DKD
ITGB3	8.14386841844676e-20	1.07838685993639	0.495	0.327	2.3552067466148e-15	Up-regulated in DKD
UACA	1.29909843176643e-19	0.612411732509904	0.794	0.695	3.75699266466851e-15	Up-regulated in DKD
NBPF14	1.98596408410949e-19	0.923803858177462	0.42	0.24	5.74340813124463e-15	Up-regulated in DKD
AKT3	5.35597187073036e-19	0.513228049759015	0.765	0.652	1.54894706501522e-14	Up-regulated in DKD
ITGB8	1.44337512100777e-18	0.591428446447181	0.758	0.637	4.17424084995446e-14	Up-regulated in DKD
MME	1.74607864878467e-18	0.48462000772166	0.791	0.744	5.04965945228528e-14	Up-regulated in DKD
ARL17B	1.94670015881279e-18	0.632037019993029	0.328	0.16	5.62985685928659e-14	Up-regulated in DKD
VTI1A	2.14849846048671e-18	0.529110872952388	0.795	0.737	6.21345754772757e-14	Up-regulated in DKD
PTPRO	3.09322701554394e-18	0.406670373693028	0.818	0.748	8.94561252895307e-14	Up-regulated in DKD
CNGB3	5.66276507111792e-18	1.87023443037845	0.162	0.048	1.6376716585673e-13	Up-regulated in DKD
SERINC5	7.66946004398946e-18	0.718728355662566	0.672	0.54	2.21800784472175e-13	Up-regulated in DKD
MT-ND4	1.02131665126364e-17	0.53405139722659	0.585	0.407	2.95364775545446e-13	Up-regulated in DKD
VIM	2.13847853893401e-17	0.84785302084093	0.438	0.276	6.18447993459716e-13	Up-regulated in DKD
FGF1	2.3227134444065e-17	0.575392203556195	0.749	0.624	6.71728728122361e-13	Up-regulated in DKD
CDC42EP3	2.38601119153775e-17	0.896213910875138	0.502	0.345	6.90034436592718e-13	Up-regulated in DKD
ZDHHC6	4.49885716193344e-17	0.546773792143601	0.724	0.59	1.30106949123115e-12	Up-regulated in DKD
RBMS1	7.94922966058576e-17	0.553448047347707	0.744	0.651	2.2989172178414e-12	Up-regulated in DKD
SULF1	8.07294699777078e-17	0.652910655379756	0.656	0.519	2.33469627175531e-12	Up-regulated in DKD
MYH9	8.42078605766509e-17	0.54595878101798	0.752	0.648	2.43529132787674e-12	Up-regulated in DKD
ARMH4	1.07275995082334e-16	0.549048464348618	0.739	0.657	3.1024217777811e-12	Up-regulated in DKD
ROBO2	1.60220901686863e-16	0.50168347040669	0.661	0.503	4.63358847678409e-12	Up-regulated in DKD
GRK3	1.66459945144844e-16	0.923283999521668	0.514	0.342	4.81402161358888e-12	Up-regulated in DKD
PLTP	1.91581471920776e-16	0.940277544262321	0.124	0.03	5.54053616794885e-12	Up-regulated in DKD
SPOCK1	2.7492506805613e-16	0.487070004103963	0.753	0.642	7.95083296818329e-12	Up-regulated in DKD
MCC	3.15898128876753e-16	0.701350194386473	0.614	0.469	9.13577388711568e-12	Up-regulated in DKD
MT-CYB	3.76061359004302e-16	0.470818292797579	0.545	0.362	1.08756945024044e-11	Up-regulated in DKD
PRDM5	4.67248317012358e-16	0.750260130807029	0.582	0.433	1.35128213279974e-11	Up-regulated in DKD
NPHS1	6.21704697497655e-16	0.522099191566589	0.796	0.685	1.79796998516322e-11	Up-regulated in DKD
DCN	1.06380610002714e-15	0.866067290610778	0.392	0.242	3.07652724127848e-11	Up-regulated in DKD
CCDC91	1.5370958463067e-15	0.456015342968208	0.784	0.705	4.44528118751897e-11	Up-regulated in DKD
HPGD	2.62411128325731e-15	0.86496918055634	0.412	0.255	7.58892983118013e-11	Up-regulated in DKD
CDK6	2.78775437470786e-15	0.735810800405198	0.572	0.427	8.06218565165512e-11	Up-regulated in DKD
AL024495.1	3.01366664131963e-15	1.05413667295865	0.473	0.327	8.71552392669637e-11	Up-regulated in DKD

Gene_Symbol	p_val	avg_log2FC	pct.1	pct.2	p_val_adj	Significance
IQGAP2	3.34819339323775e-15	0.423983694207285	0.799	0.718	9.68297529324356e-11	Up-regulated in DKD
UTRN	3.82872990504502e-15	0.485211633927972	0.786	0.684	1.10726868853902e-10	Up-regulated in DKD
TANC1	4.56963126450147e-15	0.541927764035196	0.716	0.604	1.32153736169382e-10	Up-regulated in DKD
SEL1L	4.8693700217517e-15	0.54824569298394	0.339	0.185	1.40822181029059e-10	Up-regulated in DKD
LINC00839	5.30569856450341e-15	0.742830304877951	0.575	0.421	1.53440802485439e-10	Up-regulated in DKD
NORAD	6.12975287009832e-15	0.63262695072539	0.265	0.129	1.77272453003243e-10	Up-regulated in DKD
THBS1	6.71106053310097e-15	0.903513027079374	0.264	0.131	1.9408387061728e-10	Up-regulated in DKD
AC092813.2	7.63271045126907e-15	0.620877453020403	0.663	0.53	2.20737986250702e-10	Up-regulated in DKD
SLC7A14-AS1	9.54563965046368e-15	0.960578500745621	0.383	0.235	2.7605989869141e-10	Up-regulated in DKD
TMEM130	1.16659605894038e-14	2.48922207351352	0.103	0.023	3.37379580245557e-10	Up-regulated in DKD
TMEM30A	1.28647686348385e-14	0.738248252425348	0.266	0.132	3.7204910891953e-10	Up-regulated in DKD
WT1	1.60494762448587e-14	0.547741650795512	0.716	0.571	4.64150853001314e-10	Up-regulated in DKD
NEDD9	1.60629349586744e-14	0.573093037366118	0.642	0.504	4.64540079004865e-10	Up-regulated in DKD
GULP1	1.69649110862376e-14	0.549503825460667	0.737	0.643	4.9062522861399e-10	Up-regulated in DKD
PPP2R2B	2.61563548186449e-14	0.886882368442915	0.548	0.419	7.56441781355211e-10	Up-regulated in DKD
APBB2	3.09995025277719e-14	0.49704342663566	0.804	0.718	8.96505613103163e-10	Up-regulated in DKD
MAML3	3.37146349786017e-14	0.51027737406307	0.736	0.629	9.7502724358116e-10	Up-regulated in DKD
AC104596.1	3.75076496823041e-14	1.07157116449784	0.261	0.132	1.08472122881223e-09	Up-regulated in DKD
NOTCH2	3.79227778393316e-14	0.603236006440519	0.561	0.401	1.09672673511347e-09	Up-regulated in DKD
PDE4B	4.92321340360168e-14	1.11462721915926	0.277	0.145	1.42379331632161e-09	Up-regulated in DKD
NLK	5.45472460295977e-14	0.453030082612595	0.748	0.637	1.57750635517597e-09	Up-regulated in DKD
TJP1	5.89331789939325e-14	0.398877335111792	0.794	0.715	1.70434753650453e-09	Up-regulated in DKD
SRGAP1	6.20775025944679e-14	0.391370273220357	0.762	0.663	1.79528137503201e-09	Up-regulated in DKD
BST2	7.61422199584353e-14	1.01839587599394	0.165	0.062	2.20203300119795e-09	Up-regulated in DKD
SPATA13	9.36438756546861e-14	0.447882597537967	0.812	0.721	2.70818088393352e-09	Up-regulated in DKD
SYNPO	9.44525243488728e-14	0.852290660460973	0.431	0.294	2.7315670041694e-09	Up-regulated in DKD
IL1RAP	1.30723568194197e-13	0.836837296947948	0.363	0.217	3.78052559217616e-09	Up-regulated in DKD
CALHM5	1.40260453796291e-13	1.49870671315432	0.153	0.055	4.05633232378874e-09	Up-regulated in DKD
PTGDS	1.42106652737027e-13	0.658041516966255	0.554	0.393	4.10972439715481e-09	Up-regulated in DKD
DOCK4	1.50762463074013e-13	0.499851851027563	0.697	0.589	4.36005043210045e-09	Up-regulated in DKD
BACH1	1.87267165557799e-13	0.51396697879926	0.696	0.588	5.41576642793154e-09	Up-regulated in DKD
PCOLCE2	2.02497745881419e-13	0.57492151674149	0.597	0.469	5.85623481089063e-09	Up-regulated in DKD
COL4A4	2.12317930402515e-13	0.460659801967488	0.751	0.652	6.14023454724074e-09	Up-regulated in DKD
FOS	2.40278563142805e-13	1.40789843237169	0.223	0.107	6.94885604608993e-09	Up-regulated in DKD
AL139383.1	2.51200974014596e-13	0.775299642322554	0.474	0.327	7.26473216850213e-09	Up-regulated in DKD
TENM2	2.53796634020247e-13	0.46225182106082	0.7	0.572	7.33979865586553e-09	Up-regulated in DKD
ARHGAP28	4.61798453862441e-13	0.424252839541575	0.748	0.633	1.33552112857018e-08	Up-regulated in DKD
ZNRF3	5.13555130419469e-13	0.453505629101321	0.726	0.613	1.48520143717311e-08	Up-regulated in DKD
EFCAB13	5.22837800571738e-13	0.807774797853551	0.58	0.453	1.51204691925347e-08	Up-regulated in DKD
HMGCLL1	6.220256898316e-13	1.18541611053218	0.141	0.049	1.79889829499299e-08	Up-regulated in DKD
RIPOR2	6.8367083786552e-13	0.852904915915519	0.334	0.198	1.97717606310708e-08	Up-regulated in DKD
SH3BGRL2	7.48905830497838e-13	0.716161568554475	0.407	0.268	2.16583566179975e-08	Up-regulated in DKD

Gene_Symbol	p_val	avg_log2FC	pct.1	pct.2	p_val_adj	Significance
SEMA3G	1.00851606609364e-12	1.09208518366228	0.157	0.061	2.91662846314279e-08	Up-regulated in DKD
MRGPRF	1.14836450263598e-12	1.32492208423628	0.126	0.04	3.32107014162327e-08	Up-regulated in DKD
NAPEPLD	1.33851547907233e-12	0.618990487654791	0.518	0.381	3.87098676547719e-08	Up-regulated in DKD
GOLIM4	1.91905705306575e-12	0.651374979400074	0.617	0.508	5.54991299746616e-08	Up-regulated in DKD
ZNF736	1.95861411528462e-12	0.755932037054935	0.398	0.261	5.66431202140312e-08	Up-regulated in DKD
TUSC3	2.05689357872047e-12	0.526983457243239	0.601	0.457	5.9485362296596e-08	Up-regulated in DKD
ANKRD36	2.17858762935518e-12	0.462234680170395	0.617	0.465	6.30047542409517e-08	Up-regulated in DKD
WWOX	2.24431974940288e-12	0.44992044777864	0.838	0.844	6.49057271527314e-08	Up-regulated in DKD
VASN	2.4207700478874e-12	0.790773770620424	0.167	0.069	7.00086697849037e-08	Up-regulated in DKD
FBXL7	2.5803512142791e-12	0.511686875848196	0.673	0.563	7.46237571169515e-08	Up-regulated in DKD
DPP6	2.66367135141111e-12	0.351921936082184	0.802	0.714	7.70333754828093e-08	Up-regulated in DKD
RBFOX1	2.72160441831254e-12	0.418702993104197	0.733	0.637	7.87087997775987e-08	Up-regulated in DKD
BCAM	2.99290850531509e-12	0.599288089249801	0.54	0.417	8.65549139737124e-08	Up-regulated in DKD
CCBE1	3.14412675064556e-12	0.505417650458811	0.695	0.585	9.09281456286697e-08	Up-regulated in DKD
AOPEP	3.74184592866583e-12	0.452070295713728	0.765	0.685	1.08214184257016e-07	Up-regulated in DKD
AC009271.1	4.13726524756007e-12	1.33556563348662	0.195	0.091	1.19649710959437e-07	Up-regulated in DKD
MPP5	4.69434165176077e-12	0.491958282638321	0.708	0.593	1.35760360568921e-07	Up-regulated in DKD
TRAM2	5.4036790946023e-12	0.746450851976734	0.46	0.327	1.56274399415899e-07	Up-regulated in DKD
ARHGEF37	5.80119255717214e-12	0.896806260887745	0.348	0.223	1.67770488753418e-07	Up-regulated in DKD
NBPF26	6.94659440510128e-12	0.342139108603852	0.315	0.182	2.00895510195529e-07	Up-regulated in DKD
CLDN5	7.30442963064796e-12	1.30364904493094	0.168	0.073	2.11244104918339e-07	Up-regulated in DKD
FNDC3B	8.15600068414671e-12	0.377198526569472	0.792	0.746	2.35871539785523e-07	Up-regulated in DKD
NDRG2	9.58116917650022e-12	0.769720861618431	0.293	0.166	2.77087412584386e-07	Up-regulated in DKD
CLIC5	9.93184537671773e-12	0.350348852341892	0.804	0.721	2.87228968294677e-07	Up-regulated in DKD
SIRPA	1.03723888581663e-11	0.745812438950193	0.303	0.179	2.9996948577817e-07	Up-regulated in DKD
PCED1B	1.03899049711184e-11	0.462401618456034	0.676	0.545	3.00476051764745e-07	Up-regulated in DKD
MYO1B	1.11076712161747e-11	0.461350842709464	0.712	0.605	3.21233851571771e-07	Up-regulated in DKD
ADAMTS19-AS1	1.19777772539974e-11	0.8706085878998	0.354	0.224	3.46397318185606e-07	Up-regulated in DKD
KCNT2	1.21968447132028e-11	0.747355890528434	0.335	0.202	3.52732749105824e-07	Up-regulated in DKD
CLDN11	1.34878178029168e-11	0.964374116785776	0.229	0.117	3.90067690860355e-07	Up-regulated in DKD
DLC1	1.36553214807605e-11	0.808583957466931	0.382	0.253	3.94911897223594e-07	Up-regulated in DKD
ADAM28	1.61194485362429e-11	0.821568103431759	0.317	0.19	4.66174451668145e-07	Up-regulated in DKD
AC245297.3	1.8955482383944e-11	0.418253123958162	0.705	0.585	5.4819255054366e-07	Up-regulated in DKD
EML4	2.04629089927214e-11	0.48566779869326	0.729	0.613	5.91787328069504e-07	Up-regulated in DKD
FYN	2.29914043964826e-11	0.45263379532722	0.711	0.591	6.64911415146276e-07	Up-regulated in DKD
MT-ND2	3.33170155815999e-11	0.297125803016826	0.553	0.386	9.63528090619868e-07	Up-regulated in DKD
SEMA5A	3.79008666090859e-11	0.357909802193269	0.771	0.692	1.09609306233477e-06	Up-regulated in DKD
SOX6	4.32437473605144e-11	0.376396037962039	0.779	0.721	1.25060917366608e-06	Up-regulated in DKD
SERPINI2	4.42039760985431e-11	0.778844564578475	0.379	0.254	1.27837898876987e-06	Up-regulated in DKD
MYO1D	4.50094148566185e-11	0.358540028296664	0.744	0.656	1.30167227765341e-06	Up-regulated in DKD
CABCOCO1	4.62239844824384e-11	1.42602490000775	0.171	0.077	1.33679763123212e-06	Up-regulated in DKD
WDR49	6.32114943816871e-11	0.633202754831287	0.557	0.431	1.82807641751839e-06	Up-regulated in DKD

Gene_Symbol	p_val	avg_log2FC	pct.1	pct.2	p_val_adj	Significance
APOD	7.50123846168882e-11	0.934008790127558	0.132	0.051	2.16935816312041e-06	Up-regulated in DKD
FRMD3	8.83635007664635e-11	0.44081905832763	0.746	0.651	2.55547244216612e-06	Up-regulated in DKD
ZNF608	9.65030950714322e-11	0.524738124056558	0.515	0.382	2.79086950946582e-06	Up-regulated in DKD
MYADM	9.97568970003449e-11	1.1291205446515	0.109	0.036	2.88496946124997e-06	Up-regulated in DKD
RYR3	1.07990690271579e-10	0.375719392194982	0.722	0.588	3.12309076265407e-06	Up-regulated in DKD
WIPF3	1.13604551746752e-10	0.513000318360659	0.606	0.491	3.28544363651608e-06	Up-regulated in DKD
NOS1AP	1.19488617379969e-10	0.486199577851616	0.629	0.508	3.45561081462871e-06	Up-regulated in DKD
PAMR1	1.20966990400861e-10	1.31904081167513	0.149	0.062	3.49836536239289e-06	Up-regulated in DKD
AC009154.1	1.35703220423016e-10	1.37660113553032	0.141	0.058	3.92453713463363e-06	Up-regulated in DKD
PDLIM5	1.45661860002805e-10	0.404068752845983	0.745	0.669	4.21254099128111e-06	Up-regulated in DKD
AL049838.1	1.50050231495403e-10	0.870621676703132	0.252	0.142	4.33945269484707e-06	Up-regulated in DKD
HLA-E	1.50531178294021e-10	0.582298500954724	0.203	0.1	4.35336167626309e-06	Up-regulated in DKD
CD81	1.57729244843317e-10	0.360372679610797	0.22	0.114	4.56152976086872e-06	Up-regulated in DKD
MMP16	1.78058354567562e-10	0.736276472128977	0.224	0.116	5.1494476140939e-06	Up-regulated in DKD
LY75	1.79874886981614e-10	2.07468008670964	0.145	0.062	5.20198173150829e-06	Up-regulated in DKD
ZEB2	1.96237730274416e-10	0.668196681701299	0.474	0.35	5.67519515953612e-06	Up-regulated in DKD
G0S2	2.74086771146458e-10	0.966111974969012	0.116	0.041	7.92658942155556e-06	Up-regulated in DKD
AC064875.1	3.03418734120966e-10	1.08582944069322	0.227	0.125	8.77486979077833e-06	Up-regulated in DKD
GRIK2	3.39768262475433e-10	0.508901510203604	0.56	0.425	9.82609815078951e-06	Up-regulated in DKD
SBSPON	3.54882036378609e-10	0.638887401486505	0.462	0.345	1.02631884920694e-05	Up-regulated in DKD
AC046134.2	3.79295255546454e-10	0.61050067779424	0.468	0.331	1.09692187904034e-05	Up-regulated in DKD
ZSWIM6	3.83930730507766e-10	0.306060011248534	0.822	0.754	1.11032767262846e-05	Up-regulated in DKD
F5	4.01412866532899e-10	0.613348947174096	0.409	0.281	1.16088601001315e-05	Up-regulated in DKD
ZBTB7C	4.755306217016e-10	0.440305790917117	0.65	0.504	1.37523455796103e-05	Up-regulated in DKD
DDN	4.86851360623999e-10	0.800012501340841	0.296	0.183	1.40797413492461e-05	Up-regulated in DKD
PDE7B	5.0834182128079e-10	0.61701402551027	0.714	0.627	1.47012454714405e-05	Up-regulated in DKD
NDNF	6.07115137701303e-10	0.695087185341679	0.517	0.408	1.75577697823217e-05	Up-regulated in DKD
ANXA2	6.41782263251113e-10	0.544165037847794	0.571	0.467	1.85603430532222e-05	Up-regulated in DKD
LBH	6.61494676524199e-10	0.882832817220584	0.198	0.101	1.91304260450798e-05	Up-regulated in DKD
MYLK3	6.64722995536736e-10	0.840137061163086	0.278	0.167	1.92237890309224e-05	Up-regulated in DKD
INTS12	8.89499438548216e-10	0.504557326906652	0.66	0.54	2.57243237628144e-05	Up-regulated in DKD
APOL1	9.11364486159274e-10	0.902069454973641	0.116	0.043	2.63566609397262e-05	Up-regulated in DKD
FAM180A	9.38540731431552e-10	0.556830800889256	0.206	0.109	2.71425979530005e-05	Up-regulated in DKD
STON2	1.00475984263522e-09	0.469610178026594	0.406	0.276	2.90576546490106e-05	Up-regulated in DKD
MCTP2	1.05766032577668e-09	0.765256989158999	0.433	0.312	3.05875366214617e-05	Up-regulated in DKD
ITGAV	1.32376662188978e-09	0.482164497052546	0.73	0.639	3.82833307050524e-05	Up-regulated in DKD
UTY	1.34478055271624e-09	0.583657041616009	0.315	0.196	3.88910535845536e-05	Up-regulated in DKD
LRP11	1.38176310720177e-09	0.422107848721748	0.349	0.225	3.99605890602753e-05	Up-regulated in DKD
PAM	1.39350334393827e-09	0.454921582995443	0.68	0.586	4.03001167066948e-05	Up-regulated in DKD
EFNB1	1.45601409166859e-09	0.698962374552233	0.188	0.094	4.21079275310555e-05	Up-regulated in DKD
FKBP8	1.535028058439e-09	1.35481924129333	0.104	0.037	4.43930114500559e-05	Up-regulated in DKD
NOTCH2NLC	1.66623279286339e-09	1.02306211025062	0.221	0.124	4.81874523696093e-05	Up-regulated in DKD

Gene_Symbol	p_val	avg_log2FC	pct.1	pct.2	p_val_adj	Significance
SYNE1	1.90262334036758e-09	0.341605011072574	0.755	0.688	5.50238670034304e-05	Up-regulated in DKD
EFNB2	2.00129256917091e-09	1.06878931889181	0.22	0.123	5.78773811004227e-05	Up-regulated in DKD
ARHGEF12	2.10785793258828e-09	0.325153955655743	0.782	0.711	6.0959251410453e-05	Up-regulated in DKD
CCN2	2.29286965626049e-09	0.731261302777491	0.455	0.347	6.63097904590532e-05	Up-regulated in DKD
MT-CO3	2.37078453031717e-09	0.424820301906495	0.648	0.544	6.85630886167727e-05	Up-regulated in DKD
RERE	2.56823044905837e-09	0.298295823244218	0.817	0.791	7.4273224586768e-05	Up-regulated in DKD
TNNI3K	2.78714776801381e-09	0.428592781215947	0.32	0.202	8.06043134509594e-05	Up-regulated in DKD
SH3RF1	2.82946864028617e-09	0.384758070473967	0.735	0.661	8.1828233077076e-05	Up-regulated in DKD
RERG	3.37735357196015e-09	0.404734761107469	0.71	0.613	9.76730653010875e-05	Up-regulated in DKD
CTNND1	3.43598591100726e-09	0.400279812554621	0.738	0.667	9.93687125463298e-05	Up-regulated in DKD
FAM157C	3.5287240517223e-09	0.823387082048821	0.266	0.161	0.000102050699575809	Up-regulated in DKD
AL365295.1	4.07741283165113e-09	0.646904918829347	0.491	0.382	0.000117918779091351	Up-regulated in DKD
TGFBR3	4.33091310104026e-09	0.367803839544672	0.748	0.673	0.000125250006882084	Up-regulated in DKD
PARP14	5.103804080276e-09	0.534942710358129	0.327	0.21	0.000147602014001582	Up-regulated in DKD
IGFBP2	5.53828684800563e-09	0.510238872037263	0.432	0.332	0.000160167255644323	Up-regulated in DKD
LRRC1	5.6397791593132e-09	0.569309683425793	0.398	0.282	0.000163102413287338	Up-regulated in DKD
STAT1	5.79098132840724e-09	0.670735336712758	0.366	0.25	0.000167475180017537	Up-regulated in DKD
LOX	5.86414355323912e-09	1.26209317382268	0.12	0.05	0.000169591031559675	Up-regulated in DKD
PTBP3	5.93348558972087e-09	0.331554189578862	0.691	0.577	0.000171596403254728	Up-regulated in DKD
MAGI2	6.75411080199623e-09	0.276605568659413	0.876	0.862	0.000195328884393731	Up-regulated in DKD
PARVA	6.78953624595324e-09	0.355768832098428	0.655	0.541	0.000196353388232968	Up-regulated in DKD
SLC6A6	7.60976989758993e-09	0.699908472473501	0.383	0.267	0.000220074545438301	Up-regulated in DKD
AGRN	7.84013164569997e-09	0.805504051389776	0.127	0.055	0.000226736607193643	Up-regulated in DKD
CYTOR	7.90797429472801e-09	0.683240776335931	0.203	0.11	0.000228698616603534	Up-regulated in DKD
SPATA18	8.12113887999139e-09	0.540057256023858	0.329	0.213	0.000234863336409351	Up-regulated in DKD
FBXL4	8.12693234713012e-09	0.393326721707495	0.433	0.309	0.000235030883479003	Up-regulated in DKD
GRK5	8.43823499998099e-09	0.435268074977593	0.612	0.499	0.00024403375619945	Up-regulated in DKD
KIAA1211	8.53680046809291e-09	0.407375086119367	0.545	0.419	0.000246884269537247	Up-regulated in DKD
XKR4	1.034306703651e-08	0.648872871509915	0.451	0.332	0.000299121498695871	Up-regulated in DKD
FOSB	1.10761075560049e-08	0.533004583070728	0.116	0.047	0.000320321030519662	Up-regulated in DKD
DTNA	1.1490769154983e-08	0.650398678564989	0.374	0.261	0.000332313043962108	Up-regulated in DKD
FSTL1	1.14915118600257e-08	0.421656671480509	0.274	0.169	0.000332334522991944	Up-regulated in DKD
PJA2	1.20364328589643e-08	0.439944840561081	0.448	0.333	0.000348093638281247	Up-regulated in DKD
DAPK1	1.31777544444648e-08	0.460754097824383	0.66	0.562	0.000381100658533922	Up-regulated in DKD
SSBP2	1.33589381394905e-08	0.398566647913313	0.688	0.589	0.000386340490994065	Up-regulated in DKD
NCK2	1.3656120550512e-08	0.543442501493956	0.433	0.316	0.000394935006320806	Up-regulated in DKD
BBS9	1.74033945071964e-08	0.43051455540269	0.678	0.579	0.00050330616914812	Up-regulated in DKD
CXCL14	2.64589816593003e-08	1.69824265387216	0.106	0.043	0.000765193749586966	Up-regulated in DKD
AC087477.2	2.74188902673259e-08	1.02186877992079	0.171	0.089	0.000792954306531066	Up-regulated in DKD
HTRA1	3.40697876232751e-08	0.570816414812263	0.48	0.379	0.000985298258065115	Up-regulated in DKD
PRKG1	3.556551531304e-08	0.274415872410328	0.836	0.796	0.00102855470285312	Up-regulated in DKD
SEPTIN11	4.65571564124228e-08	0.398278652262929	0.659	0.548	0.00134643296344727	Up-regulated in DKD

Gene_Symbol	p_val	avg_log2FC	pct.1	pct.2	p_val_adj	Significance
RAMP3	5.08190952318033e-08	0.561946675861723	0.233	0.14	0.00146968823410375	Up-regulated in DKD
LINC02398	5.09961619766319e-08	0.656615481807051	0.273	0.172	0.0014748090043642	Up-regulated in DKD
WT1-AS	5.3616399070342e-08	0.442483021936212	0.536	0.413	0.00155058626111429	Up-regulated in DKD
F2R	5.50446382414059e-08	0.83920167119149	0.173	0.093	0.00159189093794146	Up-regulated in DKD
ITIH5	6.06027263627426e-08	0.502229428764903	0.483	0.371	0.00175263084641052	Up-regulated in DKD
TRPC6	6.08048539117569e-08	0.394560067760199	0.491	0.375	0.00175847637512801	Up-regulated in DKD
MSRB3	6.41710300126927e-08	0.427359806632511	0.575	0.457	0.00185582618796707	Up-regulated in DKD
PLXNA4	6.68947291944576e-08	0.828963997815488	0.181	0.099	0.00193459556830371	Up-regulated in DKD
FHL1	7.56856766720252e-08	0.995932735445344	0.182	0.101	0.00218882976935497	Up-regulated in DKD
ADAMTS19	7.69169686128166e-08	0.28834621923505	0.724	0.621	0.00222443873228266	Up-regulated in DKD
LINC02021	7.7211787371653e-08	0.583869715117856	0.145	0.071	0.0022329648907882	Up-regulated in DKD
PDGFRB	7.99558094674813e-08	0.525510081591573	0.385	0.275	0.00231232200979956	Up-regulated in DKD
TMOD3	8.01617904984976e-08	0.407396876143525	0.542	0.446	0.00231827898121655	Up-regulated in DKD
RUNX1T1	8.07924282326452e-08	0.646054507777312	0.187	0.103	0.0023365170244881	Up-regulated in DKD
CSRP1	8.15858380617623e-08	0.753067855204301	0.242	0.15	0.00235946243674617	Up-regulated in DKD
TLE4	8.18727084070248e-08	0.518315065550782	0.517	0.395	0.00236775872713116	Up-regulated in DKD
TMEM98	8.69883599703251e-08	0.460066113255024	0.134	0.063	0.0025157033703418	Up-regulated in DKD
NQO1	8.7070858679953e-08	0.715519963522784	0.145	0.072	0.00251808923302424	Up-regulated in DKD
ROBO1	9.01033686375111e-08	0.468038521439404	0.563	0.448	0.00260578942099682	Up-regulated in DKD
NUTM2A-AS1	9.25266924633038e-08	0.347043552	0.482	0.363	0.00267587194603875	Up-regulated in DKD
RCAN1	9.27613288314577e-08	0.747565299763691	0.325	0.226	0.00268265762980576	Up-regulated in DKD
DAAM2	9.95224351602409e-08	0.575557792056128	0.235	0.143	0.00287818882483417	Up-regulated in DKD
GLS	1.01481354768279e-07	0.313521757882654	0.756	0.697	0.00293484077989863	Up-regulated in DKD
IGF1	1.18560990642554e-07	0.295642446400558	0.214	0.123	0.00342878384938267	Up-regulated in DKD
AC024901.1	1.2097494128126e-07	0.790862525777347	0.255	0.162	0.00349859530185404	Up-regulated in DKD
LOXL1	1.23622981366014e-07	0.804772309139922	0.202	0.116	0.00357517662110512	Up-regulated in DKD
LINC01006	1.24171190090508e-07	0.491961961689552	0.336	0.231	0.0035910308174175	Up-regulated in DKD
ATP8A1	1.40119360594376e-07	0.531993969234348	0.291	0.19	0.00405225190838934	Up-regulated in DKD
MIR4435-2HG	1.55911279574911e-07	0.409711412191706	0.402	0.29	0.00450895420530643	Up-regulated in DKD
NPIPB13	1.72280727411097e-07	0.735732909382013	0.19	0.109	0.00498235863672892	Up-regulated in DKD
REPS1	1.72420538794995e-07	0.399073949641924	0.578	0.473	0.00498640198195125	Up-regulated in DKD
BCAR3	1.75016352784929e-07	0.341398707895067	0.583	0.479	0.00506147292254015	Up-regulated in DKD
MTSS1	1.75143901187716e-07	0.284492601532328	0.771	0.702	0.00506516162234874	Up-regulated in DKD
FGFR1	1.75544529484767e-07	0.560791132359895	0.325	0.218	0.00507674779269946	Up-regulated in DKD
EGR1	1.85612507946242e-07	0.92174301202533	0.174	0.095	0.00536791372980533	Up-regulated in DKD
AC112722.1	2.00043379693616e-07	0.621536273398571	0.4	0.302	0.00578525454073937	Up-regulated in DKD
GPRC5A	2.00661837591293e-07	0.465302067359884	0.183	0.103	0.00580314034314018	Up-regulated in DKD
SCART1	2.03948791338518e-07	0.898366421142527	0.137	0.069	0.00589819904550994	Up-regulated in DKD
TVP23C	2.06858653311042e-07	0.468333728353091	0.454	0.34	0.00598235225375534	Up-regulated in DKD
HSPA5	2.10137218192451e-07	0.925330830660348	0.182	0.103	0.00607716835012569	Up-regulated in DKD
ZNF462	2.12066809915732e-07	0.437368279573054	0.555	0.449	0.00613297214276298	Up-regulated in DKD
S100A6	2.16048676418635e-07	0.256955068488039	0.343	0.239	0.00624812772202693	Up-regulated in DKD

Gene_Symbol	p_val	avg_log2FC	pct.1	pct.2	p_val_adj	Significance
CA10	2.22999825618484e-07	0.357825555904398	0.677	0.585	0.00644915495688655	Up-regulated in DKD
ARAP2	2.49460543462039e-07	0.498022296787321	0.481	0.374	0.00721439891692216	Up-regulated in DKD
ARHGAP19	2.70379413243473e-07	0.646672215981427	0.369	0.268	0.00781937263100125	Up-regulated in DKD
LINC01197	2.77102309070447e-07	0.992924630426207	0.237	0.151	0.00801379877831732	Up-regulated in DKD
ITGA3	2.77712159509282e-07	0.385335117764985	0.55	0.439	0.00803143565300845	Up-regulated in DKD
ADAMTS9-AS1	3.28120430973577e-07	0.443875988863223	0.549	0.455	0.00948924286375583	Up-regulated in DKD
ARNTL	3.36376630794745e-07	1.01871446992768	0.15	0.079	0.00972801216258404	Up-regulated in DKD
LRRC37A2	3.48428230995638e-07	0.59890528720603	0.209	0.128	0.0100765444403938	Up-regulated in DKD
AL391840.1	3.64071795836549e-07	1.3683285796841	0.132	0.067	0.010528956335593	Up-regulated in DKD
USP46	3.85301242761663e-07	0.479649136921449	0.53	0.413	0.0111429119406673	Up-regulated in DKD
AC013652.1	3.86624853475643e-07	0.744160512745483	0.278	0.188	0.0111811907625156	Up-regulated in DKD
JAZF1	4.06542693854849e-07	0.498896118769677	0.547	0.451	0.0117572147062822	Up-regulated in DKD
NT5E	4.23643902955556e-07	0.719264953466896	0.157	0.085	0.0122517816734747	Up-regulated in DKD
HFM1	4.51092133931561e-07	0.772302797339664	0.309	0.218	0.0130455845133007	Up-regulated in DKD
FNBP1	4.89165716799476e-07	0.392524824712302	0.615	0.517	0.0141466725298408	Up-regulated in DKD
LRRC2	5.20087985322068e-07	0.379862306144062	0.614	0.5	0.0150409445355142	Up-regulated in DKD
TRERF1	5.41760466907481e-07	0.929531795271567	0.132	0.067	0.0156677127029643	Up-regulated in DKD
RAI14	5.45079942380998e-07	0.361476444799711	0.653	0.555	0.0157637119336585	Up-regulated in DKD
ITGB5	5.6927889531755e-07	0.400962317131365	0.499	0.394	0.0164635456525835	Up-regulated in DKD
RORA	5.89128431285335e-07	0.322982180619027	0.783	0.745	0.0170375942327719	Up-regulated in DKD
RFX3-AS1	6.25370796006952e-07	0.353971123347983	0.416	0.308	0.0180857234205211	Up-regulated in DKD
ERAP2	6.42489601711446e-07	0.915260640211315	0.1	0.044	0.018580799281495	Up-regulated in DKD
SLC6A16	6.6536075250981e-07	0.498819279862584	0.212	0.13	0.0192422329625837	Up-regulated in DKD
RFC1	6.82542984621972e-07	0.514588768344803	0.46	0.354	0.0197391431152674	Up-regulated in DKD
F3	7.92710499965018e-07	0.406493310623532	0.383	0.28	0.0229251876589883	Up-regulated in DKD
RHOQ	8.15541989005062e-07	0.417039323810847	0.332	0.23	0.0235854743220264	Up-regulated in DKD
TPM4	8.21082316242607e-07	0.548308401297239	0.264	0.178	0.0237457005857362	Up-regulated in DKD
GMDS	9.32771498859647e-07	0.33087044203042	0.71	0.638	0.026975751747021	Up-regulated in DKD
NFASC	9.74124006731404e-07	0.298365909894043	0.731	0.629	0.0281716662746722	Up-regulated in DKD
LPCAT2	1.02054266461605e-06	0.479474763879358	0.385	0.278	0.0295140938606962	Up-regulated in DKD
ANKRD36B	1.03726280891589e-06	0.55417551004329	0.317	0.224	0.0299976404338477	Up-regulated in DKD
PTPN7	1.06290737157795e-06	1.06230653725669	0.167	0.097	0.0307392811860344	Up-regulated in DKD
AC124852.1	1.08732254440384e-06	0.774973229318116	0.327	0.24	0.031445367984159	Up-regulated in DKD
ATXN7	1.20583044449456e-06	0.354782353324485	0.443	0.339	0.0348726164547826	Up-regulated in DKD
USP9Y	1.25276493764248e-06	0.325378918593187	0.279	0.185	0.0362299619966207	Up-regulated in DKD
FAM126A	1.27807090596623e-06	0.492962324565584	0.365	0.268	0.0369618106005433	Up-regulated in DKD
CTDSPL	1.28921103602246e-06	0.25573199181711	0.759	0.674	0.0372839831617695	Up-regulated in DKD
MACF1	1.32619716999283e-06	0.253845887272308	0.864	0.805	0.0383536221561928	Up-regulated in DKD
GPC6	1.36306732958413e-06	0.329440380635149	0.741	0.682	0.039419907171573	Up-regulated in DKD
GAS1	1.40125719111785e-06	1.04526959770441	0.106	0.05	0.0405243579671282	Up-regulated in DKD
KIRREL1	1.42309416311215e-06	0.280024759143573	0.689	0.581	0.0411558831972033	Up-regulated in DKD
MED21	1.43105738286876e-06	0.6547502209304	0.241	0.158	0.0413861795125647	Up-regulated in DKD

Gene_Symbol	p_val	avg_log2FC	pct.1	pct.2	p_val_adj	Significance
VPS13B-DT	1.44019044037652e-06	0.915232151331234	0.198	0.122	0.041650307535689	Up-regulated in DKD
KLHL42	1.68149151939361e-06	0.34999886655654	0.542	0.444	0.0486287347408632	Up-regulated in DKD
MEIS1	1.71603820570238e-06	0.712016355242514	0.268	0.186	0.0496278249089128	Up-regulated in DKD
LINC00486	1.41328716372473e-72	-4.667481588	0.106	0.438	4.08722647749191e-68	Down-regulated in DKD
SLC8A1	2.3763253922693e-57	-0.938096508	0.262	0.616	6.87233303444282e-53	Down-regulated in DKD
WDR72	3.51432521636257e-47	-1.689354765	0.155	0.452	1.01634285257206e-42	Down-regulated in DKD
MTRNR2L1	9.66493684589525e-47	-11.30657309	0	0.198	2.79509973583291e-42	Down-regulated in DKD
FGD4	4.56357538949873e-44	-1.375110917	0.263	0.553	1.31978600264303e-39	Down-regulated in DKD
AC019197.1	2.58312806770725e-41	-0.829511437	0.271	0.571	7.47040637180936e-37	Down-regulated in DKD
SORCS1	1.30731700193476e-39	-2.313796834	0.053	0.274	3.78076076959531e-35	Down-regulated in DKD
SLC4A4	7.78639814368742e-38	-1.460586023	0.18	0.44	2.2518263431544e-33	Down-regulated in DKD
PTH2R	8.73922333891863e-37	-0.628107066	0.347	0.647	2.52738338961527e-32	Down-regulated in DKD
PKHD1	1.89048967470673e-34	-0.800175915	0.376	0.651	5.46729613925188e-30	Down-regulated in DKD
MECOM	2.28892125639735e-34	-0.953219177	0.351	0.611	6.61956027350114e-30	Down-regulated in DKD
RNF152	2.98440313491074e-34	-1.442945546	0.128	0.364	8.63089386616186e-30	Down-regulated in DKD
GRAMD1B	6.16284194127575e-34	-2.032269166	0.079	0.288	1.78229388941695e-29	Down-regulated in DKD
RAB11FIP3	8.52781653034017e-33	-1.31902766	0.259	0.497	2.46624454057438e-28	Down-regulated in DKD
CCSER1	1.85717677186388e-32	-0.831316952	0.352	0.619	5.37095522423035e-28	Down-regulated in DKD
ACSM2B	1.94133467912303e-32	-1.392969139	0.108	0.336	5.61433989202379e-28	Down-regulated in DKD
ARHGAP6	1.95621365137347e-32	-1.279701464	0.198	0.448	5.65736987977208e-28	Down-regulated in DKD
RALYL	3.47387650281551e-32	-1.287908639	0.143	0.377	1.00464508461424e-27	Down-regulated in DKD
MAGI1	3.54872544511045e-32	-0.88023588	0.283	0.551	1.02629139872594e-27	Down-regulated in DKD
GPC5	9.05883630796583e-31	-1.129134229	0.193	0.429	2.61981546026372e-26	Down-regulated in DKD
SPP1	1.51364917958392e-30	-0.973986721	0.079	0.276	4.37747342735671e-26	Down-regulated in DKD
ESRRG	2.79918671320395e-30	-0.930301883	0.535	0.73	8.09524797458581e-26	Down-regulated in DKD
SLC12A3	7.78164091922281e-30	-1.321549083	0.083	0.285	2.25045055383924e-25	Down-regulated in DKD
GPX3	1.13815315938206e-29	-2.266753106	0.193	0.402	3.29153893693291e-25	Down-regulated in DKD
ZBTB16	3.13018962335233e-29	-1.115454871	0.46	0.648	9.05250839073494e-25	Down-regulated in DKD
ACSM2A	4.11380897141849e-29	-1.041327662	0.168	0.401	1.18971355453423e-24	Down-regulated in DKD
SLC5A12	7.67163269869579e-29	-1.771182779	0.094	0.291	2.21863617646282e-24	Down-regulated in DKD
AC138305.1	8.50045433119292e-28	-1.185504334	0.084	0.279	2.45833139258099e-23	Down-regulated in DKD
CUBN	7.13271865613982e-27	-0.947564688	0.388	0.598	2.06278223535563e-22	Down-regulated in DKD
LRP2	1.01623937963329e-26	-1.075401193	0.235	0.459	2.93896428589949e-22	Down-regulated in DKD
AC011246.1	7.12167145730683e-26	-2.660741386	0.039	0.191	2.05958738545314e-21	Down-regulated in DKD
GLDC	8.26861614011488e-26	-1.567980196	0.06	0.227	2.39128378772122e-21	Down-regulated in DKD
EPB41L3	1.72005487075329e-25	-1.720722167	0.098	0.278	4.97439868621851e-21	Down-regulated in DKD
PPARGC1A	1.932366808063e-25	-0.883539533	0.137	0.339	5.58840480891818e-21	Down-regulated in DKD
SLC13A3	6.2052577228418e-25	-1.486313011	0.123	0.31	1.79456053344585e-20	Down-regulated in DKD
CALB1	1.78459295802974e-24	-1.84252367	0.057	0.217	5.161042834622e-20	Down-regulated in DKD
RBM47	4.69552907795052e-24	-1.256772885	0.185	0.383	1.35794700934329e-19	Down-regulated in DKD
PTGER3	8.40945412202281e-24	-0.894753121	0.136	0.333	2.432014132089e-19	Down-regulated in DKD
CA12	8.76167936494353e-24	-0.879844792	0.173	0.383	2.53387767234167e-19	Down-regulated in DKD

Gene_Symbol	p_val	avg_log2FC	pct.1	pct.2	p_val_adj	Significance
AC003044.1	9.06234942829393e-24	-1.662917448	0.071	0.233	2.62083145466261e-19	Down-regulated in DKD
SLC17A1	1.01370181039658e-23	-1.377513305	0.098	0.274	2.9316256356669e-19	Down-regulated in DKD
SLC16A9	3.20286808057923e-23	-1.636160688	0.079	0.245	9.26269448903514e-19	Down-regulated in DKD
SNTG1	3.59327366672635e-23	-1.265551352	0.04	0.183	1.03917474441726e-18	Down-regulated in DKD
INSR	4.30000711175221e-23	-0.870225326	0.504	0.655	1.24356205671874e-18	Down-regulated in DKD
SLIT2	6.20832609130628e-23	-1.098874267	0.133	0.322	1.79544790560578e-18	Down-regulated in DKD
SLC12A1	1.41703887263935e-22	-0.477877614	0.165	0.369	4.09807641967299e-18	Down-regulated in DKD
COBLL1	2.24185952780479e-22	-0.687648006	0.364	0.578	6.48345775441144e-18	Down-regulated in DKD
TFCP2L1	2.24345996637373e-22	-1.050424021	0.126	0.311	6.48808622275282e-18	Down-regulated in DKD
SLC4A1	4.48450350811428e-22	-2.473894573	0.028	0.154	1.29691841454665e-17	Down-regulated in DKD
SNX29	7.97689567329072e-22	-1.064856051	0.437	0.594	2.30691822871568e-17	Down-regulated in DKD
PAX8	2.15853591627619e-21	-0.51264108	0.195	0.401	6.24248586987075e-17	Down-regulated in DKD
DHFR	2.27747269921502e-21	-0.874631485	0.362	0.6	6.58645104612984e-17	Down-regulated in DKD
LINC01320	3.25428488239537e-21	-0.832764018	0.119	0.298	9.41139187988742e-17	Down-regulated in DKD
ATP1B1	3.32231934752291e-21	-0.75283049	0.378	0.565	9.60814755303624e-17	Down-regulated in DKD
EGF	3.43666973532515e-21	-0.728843229	0.187	0.379	9.93884887456032e-17	Down-regulated in DKD
TRABD2B	3.62152117769533e-21	-1.282253267	0.118	0.287	1.04734392458949e-16	Down-regulated in DKD
APOE	3.84601205239808e-21	-1.935219163	0.028	0.151	1.11226668555353e-16	Down-regulated in DKD
DEFB1	8.65706264559248e-21	-0.948153154	0.115	0.284	2.50362251710535e-16	Down-regulated in DKD
MALAT1	9.95178366965016e-21	-0.250337981	1	1	2.87805583726283e-16	Down-regulated in DKD
ALDOB	1.28772156715335e-20	-1.719041826	0.072	0.22	3.72409077220748e-16	Down-regulated in DKD
ASS1	2.77720739345198e-20	-1.955282587	0.087	0.235	8.03168378186312e-16	Down-regulated in DKD
KCNJ16	5.17262807900727e-20	-1.212743265	0.146	0.317	1.4959240404489e-15	Down-regulated in DKD
NHS	8.16963382584514e-20	-1.038993575	0.227	0.419	2.36265810243441e-15	Down-regulated in DKD
FXYD2	8.32858038678757e-20	-1.219920503	0.156	0.332	2.40862544785896e-15	Down-regulated in DKD
PCK1	1.1056794126385e-19	-1.44766919	0.041	0.167	3.19762486135054e-15	Down-regulated in DKD
PDZD2	1.42961263779216e-19	-1.291127454	0.223	0.392	4.13443974849493e-15	Down-regulated in DKD
PPARA	4.97039564443716e-19	-1.424038132	0.189	0.354	1.43743842037123e-14	Down-regulated in DKD
SLC16A12	7.51830911463099e-19	-1.830055209	0.076	0.217	2.17429499595128e-14	Down-regulated in DKD
BICC1	1.62551197867565e-18	-0.395921849	0.341	0.55	4.70098064232997e-14	Down-regulated in DKD
GLIS3	2.19599739883357e-18	-0.457015673	0.238	0.433	6.35082447742669e-14	Down-regulated in DKD
SLC13A1	3.15974013894529e-18	-1.29569508	0.056	0.185	9.13796848182979e-14	Down-regulated in DKD
HPN	4.19409469111795e-18	-1.623003561	0.062	0.194	1.21293218467131e-13	Down-regulated in DKD
LIN52	5.1401840708343e-18	-1.341083318	0.151	0.306	1.48654123328528e-13	Down-regulated in DKD
SETBP1	5.40711418127031e-18	-0.942272157	0.217	0.391	1.56373742122337e-13	Down-regulated in DKD
FKBP5	1.17682641939569e-17	-0.803257837	0.708	0.761	3.40338200489234e-13	Down-regulated in DKD
TEX41	1.23236104379279e-17	-1.104595437	0.09	0.233	3.56398813864875e-13	Down-regulated in DKD
SLC2A9	1.33918004193622e-17	-1.563815896	0.087	0.223	3.87290868127956e-13	Down-regulated in DKD
LRP1B	1.66904022213692e-17	-1.059133261	0.12	0.273	4.82686432241998e-13	Down-regulated in DKD
CLNK	1.94077157189001e-17	-0.594626058	0.068	0.203	5.61271138590592e-13	Down-regulated in DKD
KCNIP4	2.42672265519337e-17	-0.558486739	0.246	0.432	7.01808191881922e-13	Down-regulated in DKD
CUX1	4.24855868016852e-17	-0.564349958	0.742	0.762	1.22868317030474e-12	Down-regulated in DKD

Gene_Symbol	p_val	avg_log2FC	pct.1	pct.2	p_val_adj	Significance
SLC22A6	8.30129906732423e-17	-1.596823533	0.071	0.199	2.40073569027017e-12	Down-regulated in DKD
ANK3	8.63638046332798e-17	-0.567701596	0.435	0.614	2.49764122999445e-12	Down-regulated in DKD
LINC01060	9.68318856222563e-17	-1.817804619	0.054	0.172	2.80037813219565e-12	Down-regulated in DKD
SMIM2-AS1	1.21306197632396e-16	-1.803923686	0.055	0.173	3.5081752355289e-12	Down-regulated in DKD
PLEKHA6	2.66617040939674e-16	-1.911043154	0.046	0.158	7.71056482397536e-12	Down-regulated in DKD
ALDH6A1	3.35398780843471e-16	-2.036813404	0.057	0.173	9.69973274199317e-12	Down-regulated in DKD
TMEM52B	3.88390810736574e-16	-1.474176743	0.035	0.142	1.12322622465017e-11	Down-regulated in DKD
AQP2	4.33465073189199e-16	-1.950517793	0.026	0.123	1.25358099166316e-11	Down-regulated in DKD
PCDH15	5.48412891867248e-16	-1.582557689	0.083	0.208	1.58601008328008e-11	Down-regulated in DKD
LINC00871	5.97935037127429e-16	-1.181144086	0.089	0.22	1.72922812737252e-11	Down-regulated in DKD
BNC2	6.62333867490408e-16	-1.168364856	0.112	0.251	1.91546954478226e-11	Down-regulated in DKD
RHCG	7.27842141464826e-16	-1.482688783	0.029	0.129	2.10491947311628e-11	Down-regulated in DKD
WWC1	1.11436355082119e-15	-1.035162768	0.109	0.247	3.22273938897488e-11	Down-regulated in DKD
SDK1	1.28880959893354e-15	-1.038938417	0.114	0.255	3.72723736011579e-11	Down-regulated in DKD
MIOX	1.91284794955553e-15	-1.357170772	0.063	0.182	5.53195627011459e-11	Down-regulated in DKD
SH3BP2	2.10293811588544e-15	-2.831607306	0.025	0.116	6.08169703114071e-11	Down-regulated in DKD
NLGN1	2.49821895659529e-15	-0.711084267	0.159	0.313	7.22484922247358e-11	Down-regulated in DKD
CALCR	2.59087372111744e-15	-2.707043475	0.017	0.103	7.49280680147164e-11	Down-regulated in DKD
CKB	3.69887610299898e-15	-1.4765779	0.108	0.24	1.06971496898731e-10	Down-regulated in DKD
ATP6V0A4	5.41739674008411e-15	-0.783035686	0.062	0.179	1.56671113723232e-10	Down-regulated in DKD
HECW1	8.01092480613197e-15	-2.525713331	0.023	0.11	2.31675945393336e-10	Down-regulated in DKD
PAH	9.19364330768849e-15	-0.882655491	0.073	0.192	2.65880164458351e-10	Down-regulated in DKD
PLEKHA7	9.99229066776743e-15	-0.860651761	0.227	0.376	2.88977046111834e-10	Down-regulated in DKD
ERC1	1.26923853792835e-14	-0.580787204	0.711	0.711	3.67063785168879e-10	Down-regulated in DKD
LDHB	1.76418712325824e-14	-1.88398678	0.055	0.159	5.10202916046282e-10	Down-regulated in DKD
TMPRSS2	1.87582968530942e-14	-1.410812152	0.025	0.114	5.42489944991483e-10	Down-regulated in DKD
SULT1C2	1.91005367220383e-14	-1.182467405	0.121	0.252	5.52387522001348e-10	Down-regulated in DKD
PLG	1.96099258801589e-14	-2.922912544	0.032	0.124	5.67119056454194e-10	Down-regulated in DKD
GNGT1	2.54757791866911e-14	-1.577089264	0.072	0.186	7.36759534079108e-10	Down-regulated in DKD
EFNA5	2.57599396678557e-14	-0.932887098	0.148	0.291	7.44977455194386e-10	Down-regulated in DKD
HSP90AA1	3.43689915636979e-14	-1.119626036	0.277	0.414	9.93951236022143e-10	Down-regulated in DKD
KCNIP1	3.87732089558531e-14	-1.652820042	0.059	0.166	1.12132120300327e-09	Down-regulated in DKD
AC087762.1	4.27756742055976e-14	-2.534788982	0.03	0.12	1.23707249802588e-09	Down-regulated in DKD
AC096577.1	4.62501812085049e-14	-1.865043901	0.076	0.19	1.33755524054996e-09	Down-regulated in DKD
CCDC198	4.76247733695655e-14	-0.568170491	0.095	0.224	1.37730844584783e-09	Down-regulated in DKD
PIK3C2G	5.36567000975974e-14	-0.79674199	0.08	0.201	1.55175176682252e-09	Down-regulated in DKD
MITF	5.61670784110137e-14	-0.942947882	0.131	0.262	1.62435190764652e-09	Down-regulated in DKD
AL110292.1	6.40411092664574e-14	-1.548626351	0.059	0.165	1.85206887998595e-09	Down-regulated in DKD
SCNN1A	1.14528994395237e-13	-0.702137182	0.064	0.176	3.31217851791025e-09	Down-regulated in DKD
ACMSD	1.23071925645308e-13	-1.765892733	0.038	0.131	3.55924008966232e-09	Down-regulated in DKD
SUGCT	1.29149512793968e-13	-1.010918459	0.105	0.227	3.73500391000156e-09	Down-regulated in DKD
TINAG	1.62308267321095e-13	-0.887306229	0.085	0.204	4.69395509092607e-09	Down-regulated in DKD

Gene_Symbol	p_val	avg_log2FC	pct.1	pct.2	p_val_adj	Significance
PDZRN3	1.63308672533842e-13	-1.272686851	0.053	0.154	4.72288680967872e-09	Down-regulated in DKD
OXR1	3.0838756893276e-13	-0.716643561	0.413	0.541	8.91856849353541e-09	Down-regulated in DKD
PDE1A	3.94430476148606e-13	-0.427677227	0.177	0.327	1.14069293702177e-08	Down-regulated in DKD
L3MBTL4	4.83911652365333e-13	-0.994358698	0.186	0.322	1.39947249864054e-08	Down-regulated in DKD
TRPM3	5.50091051569191e-13	-0.546231505	0.273	0.431	1.5908633211381e-08	Down-regulated in DKD
SLC36A2	6.72176534222239e-13	-2.256090651	0.025	0.105	1.94393453697072e-08	Down-regulated in DKD
TOX3	6.94993582512556e-13	-1.197773157	0.065	0.17	2.00992144062631e-08	Down-regulated in DKD
ABTB2	7.05485941179519e-13	-0.967832087	0.088	0.202	2.04026534189117e-08	Down-regulated in DKD
ATP6V0D2	8.17324480926599e-13	-0.840740171	0.065	0.17	2.36370239883973e-08	Down-regulated in DKD
PLPPR1	8.28828569743194e-13	-1.193733231	0.064	0.167	2.39697222369732e-08	Down-regulated in DKD
FHIT	9.73877700335246e-13	-0.514954483	0.694	0.772	2.81645430936953e-08	Down-regulated in DKD
KIAA1217	1.02776263852299e-12	-0.601564512	0.351	0.5	2.97228955060848e-08	Down-regulated in DKD
ADGRF5	1.17369575176139e-12	-0.299328962	0.084	0.2	3.39432811409393e-08	Down-regulated in DKD
SCN2A	1.43860151864026e-12	-0.390012789	0.083	0.199	4.16043559190764e-08	Down-regulated in DKD
TACC1	1.44365504782015e-12	-1.137094283	0.216	0.342	4.17505039829589e-08	Down-regulated in DKD
LINC01762	1.9560314099094e-12	-1.337031806	0.069	0.173	5.656842837458e-08	Down-regulated in DKD
AGXT2	2.56756426718334e-12	-1.421907364	0.059	0.157	7.42539586069423e-08	Down-regulated in DKD
PLCL1	2.57314099773935e-12	-0.310534478	0.424	0.579	7.44152376546221e-08	Down-regulated in DKD
PDE1C	2.61996653910543e-12	-0.967455766	0.072	0.178	7.57694323109292e-08	Down-regulated in DKD
CYP3A5	2.72775809774765e-12	-1.405160892	0.055	0.151	7.88867641868619e-08	Down-regulated in DKD
ALDH1L1	2.82899096867444e-12	-1.726354607	0.057	0.152	8.18144188140649e-08	Down-regulated in DKD
SLC28A1	3.66224922585017e-12	-1.759172182	0.053	0.145	1.05912247611587e-07	Down-regulated in DKD
SAMD5	4.38554222762079e-12	-1.133606496	0.1	0.212	1.26829881222793e-07	Down-regulated in DKD
GCNT2	4.49143462059143e-12	-1.351429613	0.072	0.173	1.29892289227504e-07	Down-regulated in DKD
DPYS	4.49240062878647e-12	-1.382254363	0.036	0.121	1.29920226184505e-07	Down-regulated in DKD
TNS1	5.0810235371111e-12	-1.408674346	0.127	0.24	1.46943200693253e-07	Down-regulated in DKD
HIP1	5.08891326311094e-12	-0.938668181	0.339	0.453	1.47171371569168e-07	Down-regulated in DKD
CACNA1D	5.67099000842970e-12	-0.94463392	0.074	0.177	1.64005031043787e-07	Down-regulated in DKD
ITPR2	5.87627789917401e-12	-0.629168741	0.294	0.435	1.69941956844112e-07	Down-regulated in DKD
ADGRL2	7.06929646919324e-12	-0.597136668	0.094	0.21	2.04444053889069e-07	Down-regulated in DKD
KL	7.11837282339073e-12	-1.164537473	0.033	0.115	2.0586334205246e-07	Down-regulated in DKD
BICDL1	7.40962595470682e-12	-0.844974934	0.094	0.204	2.14286382610121e-07	Down-regulated in DKD
THRB	8.30342213160943e-12	-0.876522578	0.134	0.254	2.40134968046145e-07	Down-regulated in DKD
PAQR5	8.74464717484592e-12	-0.678903888	0.054	0.149	2.52895196296544e-07	Down-regulated in DKD
EFHD1	9.64029334292141e-12	-1.079051914	0.095	0.203	2.78797283477287e-07	Down-regulated in DKD
Z93403.1	9.71458120588002e-12	-1.329425365	0.073	0.173	2.8094568847405e-07	Down-regulated in DKD
TAF1D	1.1485623835625e-11	-0.893277216	0.274	0.4	3.32164241326276e-07	Down-regulated in DKD
SLC4A9	1.53239041638044e-11	-0.317975877	0.031	0.112	4.43167308417223e-07	Down-regulated in DKD
PPARG	1.70883537892879e-11	-0.78513668	0.054	0.145	4.94195191586206e-07	Down-regulated in DKD
NRG1	1.89734130945681e-11	-1.117828665	0.04	0.123	5.48711106694908e-07	Down-regulated in DKD
AC098588.3	1.92940156253083e-11	-1.148308889	0.176	0.298	5.57982931883916e-07	Down-regulated in DKD
KCNJ3	2.07078475348651e-11	-1.08248795	0.069	0.167	5.98870950708299e-07	Down-regulated in DKD

Gene_Symbol	p_val	avg_log2FC	pct.1	pct.2	p_val_adj	Significance
PTPRJ	2.19133151971147e-11	-0.582096651	0.219	0.347	6.33733075500557e-07	Down-regulated in DKD
AL589669.1	2.34531523647441e-11	-1.443146343	0.054	0.143	6.782651663884e-07	Down-regulated in DKD
TMEM213	2.35183940145473e-11	-0.902542077	0.062	0.158	6.80151954900707e-07	Down-regulated in DKD
KCTD1	2.39302529457019e-11	-1.330949376	0.07	0.165	6.92062915189699e-07	Down-regulated in DKD
CLDN10	2.63581938036805e-11	-1.018272519	0.067	0.163	7.62278964802439e-07	Down-regulated in DKD
RGL3	2.69563393668351e-11	-0.774374852	0.087	0.191	7.79577334488872e-07	Down-regulated in DKD
RHOBTB1	2.78821651066943e-11	-0.75082261	0.072	0.17	8.063522148856e-07	Down-regulated in DKD
LINC00970	3.60308233770189e-11	-0.992747397	0.071	0.168	1.04201141206339e-06	Down-regulated in DKD
CADPS2	3.87101746731762e-11	-0.458649413	0.289	0.426	1.11949825154825e-06	Down-regulated in DKD
NR6A1	4.01067084320406e-11	-1.132533797	0.099	0.204	1.15988600785462e-06	Down-regulated in DKD
OGDH	4.1677174698555e-11	-1.054285956	0.242	0.356	1.20530389228221e-06	Down-regulated in DKD
CACNA2D3	4.21222128147502e-11	-0.820219941	0.104	0.216	1.21817439460258e-06	Down-regulated in DKD
SLC1A1	4.59184545902531e-11	-1.0093713	0.068	0.162	1.32796170675012e-06	Down-regulated in DKD
GALNT14	4.68354618327705e-11	-1.226253392	0.085	0.185	1.35448155620372e-06	Down-regulated in DKD
MYO3B	5.02585567938017e-11	-0.859380438	0.055	0.145	1.45347746247674e-06	Down-regulated in DKD
LAMB1	5.72428686783125e-11	-0.728172031	0.105	0.216	1.6554637621768e-06	Down-regulated in DKD
CLCNKB	6.04530196605331e-11	-0.651535982	0.097	0.207	1.74830132858262e-06	Down-regulated in DKD
DHRS3	8.36236679492434e-11	-0.67827232	0.513	0.585	2.41839647709212e-06	Down-regulated in DKD
BHMT2	8.63854724629751e-11	-1.339905641	0.079	0.175	2.49826786362924e-06	Down-regulated in DKD
CRYL1	9.50915251293888e-11	-1.280936871	0.146	0.255	2.75004690674192e-06	Down-regulated in DKD
CLMN	9.94213170467357e-11	-0.864244459	0.118	0.227	2.8752644889916e-06	Down-regulated in DKD
AL391117.1	1.1822915440288e-10	-1.485342134	0.069	0.159	3.41918714533128e-06	Down-regulated in DKD
HNF1A-AS1	1.28187791251403e-10	-0.857367125	0.082	0.182	3.70719092299059e-06	Down-regulated in DKD
DDAH1	1.32661252233908e-10	-1.260190416	0.085	0.181	3.83656341460462e-06	Down-regulated in DKD
KCNJ15	1.38352352156354e-10	-1.185781861	0.097	0.197	4.00115002436174e-06	Down-regulated in DKD
RHEX	1.39086636497742e-10	-1.647189391	0.041	0.119	4.02238552751471e-06	Down-regulated in DKD
ELOVL7	1.48198317417469e-10	-1.467522192	0.04	0.117	4.28589533971321e-06	Down-regulated in DKD
PLIN5	1.51118919531931e-10	-0.612543133	0.062	0.154	4.37035915286345e-06	Down-regulated in DKD
HS6ST2	2.16539033982923e-10	-1.334328962	0.03	0.102	6.26230886278613e-06	Down-regulated in DKD
DHTKD1	2.3294166959894e-10	-1.395500103	0.106	0.203	6.73667308480134e-06	Down-regulated in DKD
ABLIM1	2.66823035409557e-10	-0.921440815	0.21	0.326	7.71652218404438e-06	Down-regulated in DKD
CYFIP2	2.88304134344834e-10	-0.752459058	0.179	0.294	8.33775556525261e-06	Down-regulated in DKD
CACNB4	3.05733776726449e-10	-1.108273524	0.1	0.198	8.84182082292891e-06	Down-regulated in DKD
CPM	3.30268181797985e-10	-1.315406372	0.067	0.155	9.55135581759773e-06	Down-regulated in DKD
EMCN	3.53793234268744e-10	-0.540083043	0.052	0.136	1.02317003350521e-05	Down-regulated in DKD
AK4	3.95876552925782e-10	-1.646452077	0.073	0.161	1.14487499106136e-05	Down-regulated in DKD
PCAT1	4.33710435628065e-10	-0.51912042	0.12	0.23	1.25429057983636e-05	Down-regulated in DKD
LINC02532	4.43036841233129e-10	-0.546800323	0.133	0.246	1.28126254484621e-05	Down-regulated in DKD
CASR	4.4455230173408e-10	-0.313306669	0.074	0.169	1.28564525661496e-05	Down-regulated in DKD
ANPEP	4.89397270492883e-10	-1.738330973	0.035	0.107	1.41533690626542e-05	Down-regulated in DKD
ADGRG1	6.10524654350743e-10	-1.11590057	0.046	0.124	1.76563730038235e-05	Down-regulated in DKD
EPS8	6.51514461498348e-10	-0.727647872	0.118	0.222	1.88417982265322e-05	Down-regulated in DKD

Gene_Symbol	p_val	avg_log2FC	pct.1	pct.2	p_val_adj	Significance
PDE4D	6.85450154801529e-10	-0.333223256	0.909	0.921	1.98232184768602e-05	Down-regulated in DKD
NCKAP5	6.87000137813496e-10	-0.800184358	0.255	0.376	1.98680439855663e-05	Down-regulated in DKD
SAFB2	6.96952065089994e-10	-0.625725101	0.188	0.296	2.01558537224026e-05	Down-regulated in DKD
ATP1A1	7.86707198045985e-10	-0.998691363	0.37	0.481	2.27515721674899e-05	Down-regulated in DKD
MTRNR2L8	9.66947871296063e-10	-1.063864602	0.033	0.104	2.79641324378821e-05	Down-regulated in DKD
DSP	1.0760107171338e-09	-1.054740738	0.046	0.123	3.11182299395096e-05	Down-regulated in DKD
KCNJ1	1.11923958027281e-09	-1.3062782	0.038	0.11	3.23684086614896e-05	Down-regulated in DKD
LNX1	1.15833785158024e-09	-1.543592061	0.034	0.104	3.34991306677007e-05	Down-regulated in DKD
PPA1	1.26271662487272e-09	-1.305008082	0.133	0.231	3.6517764791319e-05	Down-regulated in DKD
KAZN	1.2715759626902e-09	-0.337928196	0.298	0.427	3.67739768410005e-05	Down-regulated in DKD
RASSF4	1.32956366996482e-09	-1.022544212	0.142	0.244	3.84509813353825e-05	Down-regulated in DKD
NMT1	1.51496424368952e-09	-0.996067465	0.121	0.219	4.38127659275009e-05	Down-regulated in DKD
LINC01781	1.62257356385233e-09	-1.773494466	0.033	0.101	4.69248274666093e-05	Down-regulated in DKD
PEPD	1.81032883714194e-09	-1.47796706	0.073	0.157	5.2354709970145e-05	Down-regulated in DKD
SORBS2	1.84430103286336e-09	-0.495460306	0.175	0.289	5.33371858704084e-05	Down-regulated in DKD
FHOD3	1.85262174770107e-09	-0.93208795	0.186	0.294	5.35778209435151e-05	Down-regulated in DKD
FNIP2	1.90268953994168e-09	-0.814342764	0.161	0.266	5.50257814951134e-05	Down-regulated in DKD
ATP6V0C	2.16572762128391e-09	-1.366873082	0.097	0.188	6.26328428075308e-05	Down-regulated in DKD
CHP1	2.1765961510432e-09	-1.356611985	0.082	0.168	6.29471606881693e-05	Down-regulated in DKD
LINC01606	2.44140590566314e-09	-0.766654194	0.053	0.132	7.06054587917779e-05	Down-regulated in DKD
FTCD	2.53058691504767e-09	-0.681216808	0.099	0.194	7.31845735831786e-05	Down-regulated in DKD
RAMP1	2.56328760707599e-09	-0.992227048	0.093	0.185	7.41302775966375e-05	Down-regulated in DKD
ARHGAP10	2.71356060920666e-09	-1.273667783	0.067	0.148	7.84761728182566e-05	Down-regulated in DKD
PRKAG2	3.44830305132717e-09	-0.724398861	0.151	0.256	9.97249242443817e-05	Down-regulated in DKD
ZHX3	3.99994773344163e-09	-1.076603955	0.152	0.251	0.000115678488451132	Down-regulated in DKD
CLIC4	4.13024517768794e-09	-1.038521686	0.087	0.173	0.000119446690538735	Down-regulated in DKD
SLC6A13	4.17979008671283e-09	-0.714114817	0.084	0.173	0.000120879529307735	Down-regulated in DKD
LINC00671	4.50691796687322e-09	-1.540839685	0.052	0.126	0.000130340067601974	Down-regulated in DKD
MPC1	4.81997307633004e-09	-0.993285978	0.12	0.214	0.000139393621367465	Down-regulated in DKD
CLCN5	4.99111651158164e-09	-0.734430956	0.119	0.219	0.000144343089514941	Down-regulated in DKD
PNISR	5.03022074146221e-09	-0.361188069	0.72	0.734	0.000145473983843087	Down-regulated in DKD
SLC3A1	5.3593002463945e-09	-1.117980861	0.074	0.157	0.000154990963125729	Down-regulated in DKD
FAM107B	5.59916972820566e-09	-0.579316824	0.087	0.176	0.000161927988539708	Down-regulated in DKD
TBC1D4	5.73619114777603e-09	-0.844540622	0.121	0.219	0.000165890647993683	Down-regulated in DKD
HGD	6.01657891760489e-09	-1.435505695	0.036	0.102	0.000173999462297133	Down-regulated in DKD
FARP1	6.46818576372432e-09	-0.552279221	0.527	0.611	0.000187059932286907	Down-regulated in DKD
PRLR	6.75292981895174e-09	-0.826033034	0.06	0.138	0.000195294730364084	Down-regulated in DKD
AFMID	7.51934312961273e-09	-1.343342634	0.082	0.165	0.0002174594033084	Down-regulated in DKD
LIMCH1	8.71761544210622e-09	-0.608887215	0.2	0.31	0.000252113438585712	Down-regulated in DKD
GLYAT	8.74632605536201e-09	-1.414256614	0.065	0.143	0.000252943749521069	Down-regulated in DKD
TRHDE	1.06250619966944e-08	-0.714718896	0.08	0.166	0.000307276792944403	Down-regulated in DKD
BHMT	1.08860917446088e-08	-1.592510098	0.038	0.103	0.000314825773254087	Down-regulated in DKD

Gene_Symbol	p_val	avg_log2FC	pct.1	pct.2	p_val_adj	Significance
RP1	1.11098937691812e-08	-1.495030553	0.043	0.112	0.000321298127804719	Down-regulated in DKD
VAV3	1.24571664679218e-08	-0.694834104	0.1	0.19	0.0003602612542523	Down-regulated in DKD
SLC22A23	1.253771832937e-08	-0.886824987	0.143	0.238	0.000362590814085382	Down-regulated in DKD
DPF3	1.31252284959936e-08	-1.857208624	0.054	0.124	0.000379581608104135	Down-regulated in DKD
AC009060.1	1.40916844527159e-08	-0.980827782	0.046	0.116	0.000407531514372544	Down-regulated in DKD
SLC39A14	1.48899722362442e-08	-1.506956279	0.077	0.156	0.000430617997072183	Down-regulated in DKD
PRDM16	1.64559138811994e-08	-0.406710504	0.063	0.143	0.000475905029444287	Down-regulated in DKD
SLC27A2	2.02235062485692e-08	-1.225668505	0.04	0.105	0.00058486380070862	Down-regulated in DKD
BDP1	2.17549520910037e-08	-0.474866148	0.411	0.504	0.000629153214471828	Down-regulated in DKD
BACE2	2.18048056057204e-08	-0.954975236	0.089	0.173	0.000630594978117433	Down-regulated in DKD
SLC26A4	2.37126674924069e-08	-2.070784488	0.042	0.107	0.000685770343880407	Down-regulated in DKD
RCAN2	2.43473934860085e-08	-0.25642757	0.083	0.169	0.000704126619615366	Down-regulated in DKD
EPB41L1	2.72675971011357e-08	-0.838358169	0.127	0.217	0.000788578908164844	Down-regulated in DKD
TLN2	2.83690867698863e-08	-0.953859341	0.122	0.213	0.000820433989385113	Down-regulated in DKD
TAOK3	2.94802284290902e-08	-0.742207531	0.274	0.374	0.00085256820616929	Down-regulated in DKD
ARHGAP8	3.04836730275713e-08	-0.686321192	0.061	0.136	0.000881587823957363	Down-regulated in DKD
RAB3IP	3.2056264444486e-08	-1.102702701	0.148	0.239	0.000927067167734535	Down-regulated in DKD
MPPED2	3.73684834068964e-08	-0.906649558	0.079	0.16	0.00108069654012744	Down-regulated in DKD
TMTC1	3.82618149627896e-08	-0.855660453	0.101	0.188	0.00110653168872387	Down-regulated in DKD
LMO7	3.85383004348142e-08	-0.600245229	0.384	0.487	0.00111452764857483	Down-regulated in DKD
SIPA1L3	4.21788861784952e-08	-0.753301596	0.276	0.375	0.00121981338828208	Down-regulated in DKD
PRPF38B	4.27301857530467e-08	-0.573006979	0.17	0.266	0.00123575697197811	Down-regulated in DKD
CDH16	4.92956455795412e-08	-0.617121556	0.087	0.173	0.00142563007016033	Down-regulated in DKD
CHRM3	5.13365848646838e-08	-0.813739242	0.044	0.111	0.00148465403428666	Down-regulated in DKD
UMOD	5.573432487781e-08	-0.370973323	0.102	0.191	0.00161183667546627	Down-regulated in DKD
MAL	5.67388698358407e-08	-0.506449528	0.06	0.134	0.00164088811565251	Down-regulated in DKD
MIA2	6.01607241824749e-08	-0.732569407	0.264	0.365	0.00173984814335717	Down-regulated in DKD
FAM153A	6.35836046791146e-08	-0.756731608	0.088	0.169	0.00183883784731999	Down-regulated in DKD
ACAT1	6.42569464632808e-08	-1.021769659	0.074	0.151	0.00185831089171808	Down-regulated in DKD
PIK3R3	6.47086564788992e-08	-1.019147611	0.039	0.101	0.00187137434536976	Down-regulated in DKD
CMTM4	6.88381461651066e-08	-0.804328592	0.196	0.295	0.00199079918709488	Down-regulated in DKD
CDH1	6.93388130425778e-08	-1.008349378	0.042	0.106	0.00200527847319135	Down-regulated in DKD
SGK2	7.55171807172442e-08	-0.938871542	0.102	0.185	0.0021839568663427	Down-regulated in DKD
LINC01099	7.66520014857151e-08	-0.675818507	0.039	0.101	0.00221677588296688	Down-regulated in DKD
CYP4A22-AS1	8.78213716590101e-08	-1.752987776	0.075	0.148	0.00253979406837857	Down-regulated in DKD
SCIN	1.03585536517988e-07	-0.636256494	0.06	0.131	0.00299569371610023	Down-regulated in DKD
ACSF2	1.04738994974485e-07	-0.723319976	0.09	0.171	0.00302905173466211	Down-regulated in DKD
SOX5	1.14643010814037e-07	-0.438795211	0.114	0.203	0.00331547587274194	Down-regulated in DKD
CHCHD10	1.16214959014439e-07	-1.325708845	0.047	0.111	0.00336093661469757	Down-regulated in DKD
KCNMB2	1.18398801485566e-07	-0.710185618	0.107	0.192	0.00342409333896255	Down-regulated in DKD
DDX5	1.21747397850602e-07	-0.443099774	0.597	0.633	0.0035209347458394	Down-regulated in DKD
ASPA	1.32641810969074e-07	-1.155717996	0.042	0.103	0.00383600117322563	Down-regulated in DKD

Gene_Symbol	p_val	avg_log2FC	pct.1	pct.2	p_val_adj	Significance
ZNF804B	1.33516614075144e-07	-0.723301069	0.053	0.12	0.00386130047905316	Down-regulated in DKD
PFKFB2	1.53674730011193e-07	-1.627183652	0.045	0.107	0.00444427319192371	Down-regulated in DKD
SLC16A7	1.72028159907802e-07	-0.733521463	0.132	0.222	0.00497505438453363	Down-regulated in DKD
ESRRB	1.76393530670603e-07	-1.452796207	0.046	0.108	0.00510130090699384	Down-regulated in DKD
HSP90AB1	1.89035836843675e-07	-1.097823854	0.149	0.232	0.00546691640151908	Down-regulated in DKD
ATP6V1A	2.04781145193617e-07	-0.752744108	0.204	0.298	0.00592227071899941	Down-regulated in DKD
SLC19A2	2.29564287740923e-07	-1.054542261	0.049	0.113	0.00663899920146749	Down-regulated in DKD
SLC2A11	2.32826554983889e-07	-0.960424103	0.048	0.111	0.00673334397013408	Down-regulated in DKD
GDA	2.53234356610295e-07	-1.149436809	0.05	0.114	0.00732353759316974	Down-regulated in DKD
MYO10	2.61918725904159e-07	-0.532560281	0.131	0.219	0.00757468955314826	Down-regulated in DKD
LARP4	2.99158962615281e-07	-0.800017409	0.313	0.401	0.00865167719883392	Down-regulated in DKD
POR	3.06793569020045e-07	-1.1684548	0.099	0.175	0.00887247001605971	Down-regulated in DKD
EPAS1	3.23015237586371e-07	-1.484953897	0.062	0.129	0.00934160067099784	Down-regulated in DKD
PPP2R3A	3.2915568436233e-07	-0.532607513	0.38	0.477	0.00951918239175858	Down-regulated in DKD
TOX	3.55537086373286e-07	-0.355912227	0.086	0.164	0.0102821325379154	Down-regulated in DKD
CHKA	3.71574869915392e-07	-0.916178376	0.153	0.235	0.0107459452379531	Down-regulated in DKD
MYO6	4.0790294496083e-07	-0.534300964	0.568	0.624	0.0117965531682672	Down-regulated in DKD
SHISA6	4.09597668522698e-07	-0.985510763	0.1	0.174	0.0118455645736764	Down-regulated in DKD
FMN1	4.15384076833318e-07	-0.805148185	0.069	0.138	0.0120129075020196	Down-regulated in DKD
CTSH	4.94689657439636e-07	-1.265990725	0.043	0.101	0.0143064248931543	Down-regulated in DKD
MUC1	5.57103965441701e-07	-0.61379767	0.061	0.129	0.016111446680574	Down-regulated in DKD
TSPYL2	5.59247822705534e-07	-0.875076282	0.076	0.146	0.016173447032644	Down-regulated in DKD
SLC26A6	5.80737367898162e-07	-1.756804673	0.046	0.104	0.0167949246796149	Down-regulated in DKD
DSCAML1	6.09077665639113e-07	-1.206886418	0.063	0.129	0.0176145260902831	Down-regulated in DKD
UGT2B7	6.0938188612162e-07	-1.523228627	0.055	0.116	0.0176233241466372	Down-regulated in DKD
RBM6	6.23740943095833e-07	-0.44811051	0.605	0.643	0.0180385880743315	Down-regulated in DKD
AC079352.1	6.51435661323786e-07	-1.343632075	0.064	0.129	0.0188395193254839	Down-regulated in DKD
TMEM178B	6.89987382040737e-07	-1.247176081	0.046	0.105	0.0199544350886181	Down-regulated in DKD
PLPP3	7.25600745839956e-07	-0.54328027	0.266	0.357	0.0209843735696915	Down-regulated in DKD
COL7A1	7.54651497404287e-07	-0.702719077	0.152	0.239	0.021824521304932	Down-regulated in DKD
PNN	8.18247903411719e-07	-0.659207213	0.344	0.422	0.0236637293666669	Down-regulated in DKD
MAP3K13	8.59673034728684e-07	-0.484524294	0.223	0.317	0.0248617441643535	Down-regulated in DKD
LLGL2	8.88973932120532e-07	-0.630038134	0.148	0.232	0.0257091261169258	Down-regulated in DKD
BCR	9.01903879375802e-07	-0.863735126	0.087	0.158	0.0260830601915482	Down-regulated in DKD
BMPR1B	9.63466429858074e-07	-0.406076825	0.122	0.205	0.0278634491514955	Down-regulated in DKD
MAOA	1.00990985316828e-06	-1.005897122	0.126	0.203	0.0292065929536266	Down-regulated in DKD
FGF13	1.05416772081146e-06	-0.3653989	0.238	0.346	0.0304865304858675	Down-regulated in DKD
AFM	1.08342735505216e-06	-0.790360832	0.054	0.114	0.0313327191081085	Down-regulated in DKD
ENOX1	1.11417585271684e-06	-0.807687125	0.105	0.18	0.0322219656605711	Down-regulated in DKD
TMTC2	1.11599667578236e-06	-0.299747029	0.102	0.179	0.032274623863626	Down-regulated in DKD
AP3D1	1.27422559937121e-06	-0.655272753	0.165	0.245	0.0368506043338154	Down-regulated in DKD
ABLIM2	1.30106319777979e-06	-0.623100252	0.703	0.644	0.0376267476797915	Down-regulated in DKD

Gene_Symbol	p_val	avg_log2FC	pct.1	pct.2	p_val_adj	Significance
STK32B	1.32353720582979e-06	-0.672291896	0.07	0.136	0.0382766959925977	Down-regulated in DKD
FREM1	1.42073440303642e-06	-0.485091584	0.114	0.191	0.0410876389358132	Down-regulated in DKD
PDZK1	1.43786363605468e-06	-0.820999455	0.075	0.142	0.0415830163547012	Down-regulated in DKD
RNF130	1.53467243371705e-06	-0.650436485	0.27	0.355	0.0443827267830972	Down-regulated in DKD
UQCRH	1.58122301133002e-06	-1.147522623	0.055	0.114	0.0457289694876641	Down-regulated in DKD
FAM160A1	1.6542983657016e-06	-0.430704499	0.149	0.235	0.0478423087360903	Down-regulated in DKD
ACSS2	1.71206371009414e-06	-1.123257809	0.152	0.23	0.0495128824959226	Down-regulated in DKD

Supplementary Table S4. Patient-level pseudobulk differential expression in DKD podocytes.

Gene	baseMean	log2FC	SE	Wald z	P value	BH-FDR	Direction
NDRG2	56.3	+1.030	0.194	+5.309	1.10E-07	0.0006	Up
SLC16A9	41.7	-1.730	0.329	-5.258	1.45E-07	0.0006	Down
DHRS3	226.1	-0.860	0.168	-5.119	3.07E-07	0.0007	Down
FGD4	129.7	-1.350	0.265	-5.094	3.50E-07	0.0007	Down
MT-CO2	921.5	+1.320	0.270	+4.889	1.01E-06	0.0016	Up
TMEM52B	27.9	-1.970	0.417	-4.724	2.31E-06	0.0031	Down
ACSM2B	83.1	-1.160	0.249	-4.659	3.18E-06	0.0034	Down
CDK6	259.8	+1.050	0.226	+4.646	3.38E-06	0.0034	Up
GRIK4	12.7	+1.780	0.395	+4.506	6.60E-06	0.0059	Up
ALDH6A1	89.2	-1.260	0.283	-4.452	8.50E-06	0.0068	Down
OXR1	178.4	-0.735	0.168	-4.375	1.21E-05	0.0086	Down
MT-ND4	565.7	+0.650	0.149	+4.362	1.29E-05	0.0086	Up
ACSM2A	127.9	-0.940	0.218	-4.312	1.62E-05	0.0093	Down
GRAMD1B	47.4	-1.530	0.355	-4.310	1.63E-05	0.0093	Down
GLDC	66.1	-1.200	0.285	-4.211	2.55E-05	0.0132	Down
FHIT	112.1	-0.580	0.138	-4.203	2.64E-05	0.0132	Down
CYP3A5	39.2	-1.250	0.299	-4.181	2.91E-05	0.0137	Down
NOX4	138.8	+0.760	0.185	+4.108	3.99E-05	0.0177	Up

Note: Raw podocyte counts from the GSE195460 discovery cohort were aggregated within each donor (six control and five DKD donors), with each donor treated as one independent biological replicate. Differential expression was analyzed with DESeq2 using disease status as the design factor (DKD vs. control). This table lists 18 genes meeting BH-FDR < 0.05 and |log2FC| > 0.25 (six upregulated and 12 downregulated in DKD).

Supplementary Table S5. Kyoto Encyclopedia of Genes and Genomes (KEGG) pathway enrichment analysis of podocyte DEGs.

category	subcategory	ID	Description	GeneRatio	BgRatio	RichFactor	FoldEnrichment	zScore	pvalue	p.adjust	qvalue	geneID	Count
NA	NA	hsa04517	IgSF CAM signaling	34/334	299/9375	0.1137123745819 4	3.1917769811547 5	7.4030756174506 8	1.59654718520 27e-09	4.96526174598039e-07	4.01657660277 31e-07	MAGI1/UNC5D/SRGAP2/PTPRD/EPB41L3/SLIT2/UNC5C/ITGB3/AKT3/MYH9/ANK3/ROBO2/NPHS1/NLGN1/ERC1/TJP1/SRGAP1/IL1RAP/FYN/MYLK3/MAGI2/VAV3/NCK2/EPB41L1/LMO7/TRPC6/PIK3R3/PLXNA4/CDH1/ROBO1/MTSS1/FGFR1/NFASC/KIRREL1	34
Cellular Processes	Cell motility	hsa04820	Cytoskeleton in muscle cells	28/334	233/9375	0.1201716738197 42	3.3730821618565 4	7.0501377114675	1.42154927745469e-08	2.21050912644204e-06	1.78815935427195e-06	NEBL/BGN/COL4A3/FMN2/ATP1B1/ITGB3/ITGB8/VIM/MYH9/ANK3/DCN/THBS1/COL4A4/PDLIM5/ATP1A1/DSP/ITGAV/SYNE1/AGRN/DTNA/TLN2/FHL1/TMOD3/CSRP1/DAAM2/ITGA3/ITGB5/TPM4	28
NA	NA	hsa04518	Integrin signaling	21/334	154/9375	0.1363636363636 36	3.8275721284703 3	6.8000564023303 4	1.12037061800122e-07	1.16145087399 46e-05	9.39538869130851e-06	NPNT/SPP1/COL4A3/EGF/ITGB3/AKT3/ITGB8/THBS1/COL4A4/LAMB1/ITGAV/PARVA/NCK2/TLN2/PIK3R3/CDH1/PDGFRB/IGF1/ITGA3/ITGB5/FREM1	21
Organismal Systems	Development and regeneration	hsa04360	Axon guidance	22/334	184/9375	0.1195652173913 04	3.3560596198906 5	6.2035641545162 3	5.79807621383937e-07	4.50800425626011e-05	3.64668477659897e-05	UNC5D/SRGAP2/NTNG1/SLIT2/UNC5C/ROBO2/EFNA5/SRGAP1/SEMA3G/FYN/SEMA5A/ABLIM1/EFNB1/EFNB2/ARHGEF12/NCK2/TRPC6/PIK3R3/PLXNA4/ROBO1/BMP	22

category	subcategory	ID	Description	GeneRatio	BgRatio	RichFactor	FoldEnrichment	zScore	pvalue	p.adjust	qvalue	geneID	Count
												R1B/ABLIM2	
Environmental Information Processing	Signaling molecules and interaction	hsa04512	ECM-receptor interaction	14/334	89/9375	0.15730337078651 7	4.41532665007064	6.2221486 73	2.742397179132 18e-06	0.00017057710454 2021	0.00013798587911 844	NPNT/SPP1/CD47/COL4A3/ITGB3/ITGB8/THBS1/COL4A4/LAMB1/ITGAV/AGRN/ITGA3/ITGB5/FREM1	14
Environmental Information Processing	Signal transduction	hsa04151	PI3K-Akt signaling pathway	31/334	362/9375	0.08563535911602 21	2.40368710093625	5.23500839 09695	4.972862814517 37e-06	0.00025776005588 5817	0.00020851126538 0641	MAGI1/SPP1/INSR/COL4A3/EGF/ITGB3/PCK1/AKT3/ITGB8/FGF1/CDK6/THBS1/GNGT1/EFNA5/PPP2R2B/HSP90AA1/COL4A4/LAMB1/ITGAV/PRLR/MAGI2/F2R/PIK3R3/SGK2/PDGFRB/IGF1/FGFR1/HSP90AB1/ITGA3/PPP2R3A/ITGB5	31
Environmental Information Processing	Signal transduction	hsa04015	Rap1 signaling pathway	21/334	212/9375	0.09905660377358 49	2.78040616879449	5.03959516 878917	2.092525550039 3e-05	0.00092967920866 0317	0.00075205053602 9162	MAGI1/PLCE1/INSR/EGF/ITGB3/AKT3/FGF1/THBS1/EFNA5/DOCK4/CTNND1/MAGI2/VAV3/TLN2/SIPA1L3/F2R/PIK3R3/CDH1/PDGFRB/IGF1/FGFR1	21
Organismal Systems	Endocrine system	hsa04919	Thyroid hormone signaling pathway	15/334	122/9375	0.12295081967213 1	3.45108962403063	5.23751523 352271	2.674882782093 95e-05	0.00103986068153 902	0.00084118024331 6385	PLCE1/ATP1B1/ITGB3/FXYD2/AKT3/NOTCH2/THRB/ATP1A1/ITGAV/TBC1D4/STAT1/RCAN2/PIK3R3/RCAN1/PFKFB2	15
Cellular Processes	Cellular community - eukaryotes	hsa04510	Focal adhesion	20/334	203/9375	0.09852216748768 47	2.76540515029055	4.88751224 580168	3.560755100399 13e-05	0.00123043870691 57	0.00099534557777 2388	SPP1/COL4A3/EGF/ITGB3/AKT3/ITGB8/THBS1/COL4A4/FYN/LAMB1/MYLK3/ITGAV/PARVA/VAV3/TLN2/PIK3R3/PDGFRB/IG	20

category	subcategory	ID	Description	GeneRatio	BgRatio	RichFactor	FoldEnrichment	zScore	pvalue	p.adjust	qvalue	geneID	Count
												F1/ITGA3/ITGB5	
Organismal Systems	Excretory system	hsa04960	Aldosterone-regulated sodium reabsorption	8/334	38/9375	0.2105263157894774	5.90923416325244	5.8281314717954	4.57812490039424e-05	0.00129738052801324	0.00104949719477124	INSR/ATP1B1/FXYD2/SCNN1A/ATP1A1/KCNJ1/PIK3R3/IGF1	8
Human Diseases	Cardiovascular disease	hsa05410	Hypertrophic cardiomyopathy	13/334	99/9375	0.131313131313131	3.68581019778625	5.16345051426454	4.58880572609183e-05	0.00129738052801324	0.00104949719477124	SLC8A1/ITGB3/ITGB8/CACNA1D/CACNA2D3/CACNB4/ITGAV/PRKAG2/DTNA/IGF1/ITGA3/ITGB5/TPM4	13
Human Diseases	Infectious disease: viral	hsa05165	Human papillomavirus infection	27/334	333/9375	0.0810810810810811	2.27585369800939	4.55636194053552	5.43429603946482e-05	0.00140838839022797	0.00113929539774745	MAGI1/SPP1/COL4A3/EGF/ITGB3/AKT3/ITGB8/CDK6/ATP6V0A4/THBS1/PPP2R2B/MAML3/NOTCH2/COL4A4/ATP6V0D2/LAMB1/HLA-E/ITGAV/ATP6V0C/STAT1/PIK3R3/PDGFRB/ATP6V1A/ITGA3/PPP2R3A/ITGB5/LLGL2	27
Cellular Processes	Cell motility	hsa04810	Regulation of actin cytoskeleton	21/334	232/9375	0.0905172413793103	2.54071598182944	4.567196461	7.94454885812526e-05	0.00190058053452073	0.00153744710695703	EGF/ITGB3/AKT3/ITGB8/FGF1/MYH9/IQGAP2/SPATA13/CYFIP2/MYLK3/ITGAV/ARHGEF12/VAV3/CHRM3/F2R/PIK3R3/PDGFRB/SCIN/FGFR1/ITGA3/ITGB5	21
Organismal Systems	Excretory system	hsa04964	Proximal tubule bicarbonate reclamation	6/334	23/9375	0.260869565217391	7.32231189794324	5.83465335828897	0.00011801372220045	0.00262159054315814	0.00212069771472261	SLC4A4/ATP1B1/FXYD2/PCK1/ATP1A1/GLS	6
Human Diseases	Cardiovascular disease	hsa05412	Arrhythmogenic right ventricular cardiomyopathy	11/334	86/9375	0.127906976744186	3.59020331430163	4.63795010615553	0.00022286871023365	0.00462081125883843	0.00373793836812451	SLC8A1/ITGB3/ITGB8/CACNA1D/CACNA2D3/CACNB4/DSP/ITGAV/DTNA/ITGA3/ITGB5	11

category	subcategory	ID	Description	GeneRatio	BgRatio	RichFactor	FoldEnrichment	zScore	pvalue	p.adjust	qvalue	geneID	Count
Human Diseases	Cancer: specific types	hsa05218	Melanoma	10/334	73/9375	0.136986301369863	3.84504962677385	4.69019157407673	0.000242832899165006	0.00472006447751981	0.00381822782239714	EGF/AKT3/FGF1/CDK6/MITF/PIK3R3/CDH1/PDGFRB/IGF1/FGFR1	10
Organismal Systems	Digestive system	hsa04974	Protein digestion and absorption	12/334	103/9375	0.116504854368932	3.27015871170281	4.45261961304685	0.000284683704416234	0.00520803718079111	0.00421296627588111	SLC8A1/DPP4/COL4A3/ATP1B1/FXYD2/MME/COL4A4/SLC36A2/SLC1A1/ATP1A1/SLC3A1/COL7A1	12
Human Diseases	Cardiovascular disease	hsa05414	Dilated cardiomyopathy	12/334	105/9375	0.114285714285714	3.20786997433704	4.37276108319194	0.000340930739404194	0.00589052555303912	0.00476505536360247	SLC8A1/ITGB3/ITGB8/CACNA1D/CACNA2D3/CACNB4/ITGAV/DTNA/IGF1/ITGA3/ITGB5/TPM4	12
Organismal Systems	Excretory system	hsa04966	Collecting duct acid secretion	6/334	28/9375	0.214285714285714	6.01475620188195	5.10763896980287	0.000378855405091749	0.00620126478860705	0.00501642336935889	SLC4A1/ATP6V0A4/ATP6V0D2/CLCNKB/ATP6V0C/ATP6V1A	6
Organismal Systems	Endocrine system	hsa04918	Thyroid hormone synthesis	9/334	75/9375	0.12	3.36826347305389	3.95773441590594	0.00131234488108445	0.0204069629008632	0.0165079171883781	GPX3/LRP2/PAX8/ATP1B1/FXYD2/ITPR2/ATP1A1/SLC26A4/HSPA5	9
Human Diseases	Infectious disease: bacterial	hsa05130	Pathogenic Escherichia coli infection	16/334	200/9375	0.08	2.24550898203593	3.42204958852087	0.0020782919211434	0.0307785136893141	0.0248978330402642	MYH9/TJP1/FOS/CLDN5/MYO1B/CLDN11/FYN/CLDN10/MYO1D/WIPF3/CYFIP2/ARHGEF12/NCK2/F2R/MYO10/MYO6	16
Metabolism	Metabolism of cofactors and vitamins	hsa00670	One carbon pool by folate	6/334	39/9375	0.153846153846154	4.31828650391525	3.99110575808639	0.00235977023441396	0.0333585701319428	0.0269849323456907	GLDC/DHFR/ALDH1L1/BHMT2/FTCD/BHMT	6
Organismal Systems	Circulatory system	hsa04260	Cardiac muscle contraction	9/334	87/9375	0.103448275862069	2.90367540780508	3.4286163824048	0.00369276230087413	0.0499325685031241	0.0403922283711175	SLC8A1/ATP1B1/FXYD2/CACNA1D/CACNA2D3/CACNB4/ATP1A1/TPM4/UQCRH	9

Supplementary Table S6. Gene Ontology (GO) enrichment analysis of podocyte DEGs.

ID	Description	GeneRatio	BgRatio	RichFactor	FoldEnrichment	zScore	pvalue	p.adjust	qvalue	geneID	Count
GO:0001822	kidney development	37/568	322/18986	0.114906832298137	3.84088225002187	9.0289456861796	2.41500108163471e-12	1.09713499138665e-08	8.90245661882606e-09	PKHD1/NPNT/ZBTB16/LRP2/CALB1/PLCE1/SLIT2/COL4A3/PAX8/PODXL/ASS1/ITGB3/BICC1/MME/PTPRO/FGF1/SULF1/SLC22A6/ROBO2/AQP2/NPHS1/HPGD/WT1/NOTCH2/COL4A4/EFNB2/STAT1/MAGI2/UMOD/ACAT1/PDGFRB/MTSS1/FGFR1/EGR1/ITGA3/FMN1/CTSH	37
GO:0072001	renal system development	37/568	332/18986	0.111445783132530	3.72519302562362	8.79708035036096	6.06069330662625e-12	1.37668648460015e-08	1.11708147156869e-08	PKHD1/NPNT/ZBTB16/LRP2/CALB1/PLCE1/SLIT2/COL4A3/PAX8/PODXL/ASS1/ITGB3/BICC1/MME/PTPRO/FGF1/SULF1/SLC22A6/ROBO2/AQP2/NPHS1/HPGD/WT1/NOTCH2/COL4A4/EFNB2/STAT1/MAGI2/UMOD/ACAT1/PDGFRB/MTSS1/FGFR1/EGR1/ITGA3/FMN1/CTSH	37
GO:0072006	nephron development	24/568	160/18986	0.15	5.01390845070423	8.95378498940751	7.62237410166076e-11	1.15428151812816e-07	9.36615933474245e-08	NPNT/CALB1/PLCE1/COL4A3/PAX8/PODXL/ITGB3/PTPRO/FGF1/SULF1/SLC22A6/NPHS1/WT1/NOTCH2/COL4A4/STAT1/MAGI2/UMOD/ACAT1/PDGFRB/MTSS1/EGR1/ITGA3/FMN1	24
GO:0032970	regulation of actin filament-based process	38/568	395/18986	0.0962025316455696	3.21567124264575	7.81467151890899	2.4993211291 26e-10	2.83860397240485e-07	2.30332173531559e-07	ARHGAP6/SLIT2/CD47/ITGB3/CDC42EP3/MYH9/NPHS1/IQGAP2/NEDD9/EFNA5/PDE4B/TJP1/BST2/SYNPO/ARHGAP28/DLC1/SEMA5A/MYO3B/MYADM/ZEB2/CYFIP2/EPS8/MYLK3/PDE4D/	38

ID	Description	GeneRatio	BgRatio	RichFactor	FoldEnrichment	zScore	pvalue	p.adjust	qvalue	geneID	Count
										DSP/CCN2/LIMCH1/NCK2/PDGFRB/TMOD3/DAAM2/SCIN/MTSS1/ARAP2/FMN1/RHOQ/FGF13/KIRREL1	
GO:0031589	cell-substrate adhesion	34/568	361/18986	0.0941828254847645	3.14816043072842	7.23653067036762	3.87271697765089e-09	3.5187506458936e-06	2.85521154857545e-06	PKHD1/ARHGAP6/NPNT/SPOCK2/SRGAP2/ITGB3/ITGB8/PTPRO/SPOCK1/CDK6/UTRN/THBS1/NEDD9/PLG/EFNA5/BCAM/GCNT2/DLC1/PTPRJ/LAMB1/APOD/MYADM/NDNF/ANXA2/ITGAV/CCN2/PARVA/LIMCH1/ITGA3/FMN1/ITGB5/BCR/MACF1/FREM1	34
GO:0043954	cellular component maintenance	15/568	77/18986	0.194805194805195	6.51156941649899	8.51029466378916	7.29726782035924e-09	5.52524795131533e-06	4.48333893103475e-06	INSR/APOE/ITGB3/TANC1/PLEKHA7/ERC1/NEDD9/TJP1/FYN/F2R/PLXNA4/CHCHD10/MTSS1/ITGA3/KIRREL1	15
GO:0050919	negative chemotaxis	12/568	47/18986	0.25531914893617	8.53431225651783	9.08180675469392	9.20125687097895e-09	5.97161570926534e-06	4.84553406949899e-06	DPP4/SLIT2/UNC5C/ITGB3/ROBO2/EFNA5/SEMA3G/NRG1/SEMA5A/ITGAV/PLXNA4/ROBO1	12
GO:0032835	glomerulus development	14/568	72/18986	0.194444444444444	6.49951095461659	8.21025907680697	2.37585767248446e-08	1.31525065583786e-05	1.0672307417413e-05	PLCE1/COL4A3/PODXL/ITGB3/PTPRO/SULF1/NPHS1/WT1/NOTCH2/COL4A4/MAGI2/PDGFRB/MTSS1/EGR1	14
GO:0032956	regulation of actin cytoskeleton organization	32/568	352/18986	0.0909090909090909	3.0387323943662	6.78011006609956	2.60560332435411e-08	1.31525065583786e-05	1.0672307417413e-05	ARHGAP6/SLIT2/CD47/ITGB3/CDC42EP3/NPHS1/IQGAP2/NEDD9/EFNA5/TJP1/BST2/SYNPO/ARHGAP28/DLC1/SEMA5A/MYO3B/MYADM/CYFIP2/EPS8/MYLK3/CCN2/LIMCH1/NCK2/PDGFRB/TMOD3/DAAM2/SCIN/MTSS1/ARAP2/FMN1/RHOQ/KIRREL1	32

ID	Description	GeneRatio	BgRatio	RichFactor	FoldEnrichment	zScore	pvalue	p.adjust	qvalue	geneID	Count
GO:0072073	kidney epithelium development	20/568	153/18986	0.130718954248366	4.36941912915401	7.34850905452297	3.23748032709027e-08	1.47078731259711e-05	1.19343748478633e-05	NPNT/CALB1/SLIT2/PAX8/PODXL/PTPRO/FGF1/SLC22A6/ROBO2/NPHS1/WT1/NOTCH2/EFNB2/STAT1/MAGI2/UMOD/ACAT1/MTSS1/FGFR1/FMN1	20
GO:0031346	positive regulation of cell projection organization	32/568	363/18986	0.0881542699724518	2.94664959453692	6.57619236836872	5.36721077900222e-08	2.21665805172792e-05	1.79865762182448e-05	RAB11FIP3/PTPRD/PLCE1/SLIT2/APOE/CDC42EP3/CUX1/ROBO2/NLGN1/TMEM30A/NEDD9/EFNA5/HSP90AA1/TENM2/RIPOR2/FYN/SEMA5A/MYO3B/NDNF/EPS8/MAGI2/AGRN/RP1/RAB3IP/PLXNA4/ROBO1/FGFR1/ITGA3/TOX/RHOQ/MAP3K13/MACF1	32
GO:0015711	organic anion transport	37/568	462/18986	0.0800865800865801	2.67697853789403	6.40824843893909	5.99531456216029e-08	2.26972617132452e-05	1.84171856111275e-05	PLA2R1/SLC4A4/SLC5A12/LRP2/SLC13A3/SLC17A1/SLC16A9/CD47/CR1/SLC4A1/APOE/PPARA/SLC16A12/SERINC5/SLC2A9/SLC22A6/THBS1/CALHM5/SLC36A2/SLC4A9/PPARG/SLC1A1/CACNB4/CASR/SLC6A13/MPC1/SLC3A1/SLC6A6/SLC39A14/SLC27A2/SLC26A4/PRKG1/UMOD/SLC16A7/SLC19A2/SLC26A6/LLGL2	37
GO:0007015	actin filament organization	37/568	465/18986	0.0795698924731183	2.6597077086173	6.36332501508821	7.07417630322501e-08	2.47215253427317e-05	2.00597290800761e-05	NEBL/ARHGAP6/SLIT2/CD47/FMN2/CDC42EP3/AQP2/NPHS1/IQGAP2/NEDD9/TJP1/SYNPO/ARHGAP28/HIP1/MYO1B/DLC1/RHOBTB1/SEMA5A/MYO1D/MYADM/WIPF3/ZEB2/CYFIP2/EPS8/CCN2/LIMCH1/NCK2/T	37

ID	Description	GeneRatio	BgRatio	RichFactor	FoldEnrichment	zScore	pvalue	p.adjust	qvalue	geneID	Count
										MOD3/DAAM2/SCIN/MTSS1/MYO6/FMN1/ITGB5/RHOQ/TPM4/KIRREL1	
GO:0046847	filopodium assembly	13/568	70/18986	0.185714285714286	6.20769617706237	7.66546033416196	1.33437597859302e-07	4.33005005053436e-05	3.51352231355847e-05	FGD4/SRGAP2/SRGAP2C/NLGN1/SPATA13/TENM2/RIPOR2/MYO3B/AGRN/RAB3IP/DAAM2/MYO10/RHOQ	13
GO:0045785	positive regulation of cell adhesion	38/568	499/18986	0.0761523046092184	2.54547122413842	6.14378783411689	1.46065840242735e-07	4.4238474148183e-05	3.58963208793023e-05	DPP4/NPNT/MAGI1/SPOCK2/ZBTB16/CD47/CR1/PODXL/ITGB3/PCK1/ANK3/CDK6/UTRN/NEDD9/TJP1/GCNT2/SIRPA/NRG1/PTPRJ/FYN/LAMB1/MYADM/HLA-E/CD81/NDNF/ADGRG1/ITGAV/EFNB1/EFNB2/IGFBP2/VAV3/NCK2/IGF1/MYO10/ITGA3/FMN1/PLPP3/AP3D1	38
GO:0010810	regulation of cell-substrate adhesion	23/568	223/18986	0.103139013452915	3.44753047432578	6.45635845754414	2.63055111963389e-07	7.46912108531046e-05	6.06065132957754e-05	PKHD1/ARHGAP6/NPNT/SPOCK2/ITGB3/PTPRO/SPOCK1/CDK6/UTRN/THBS1/NEDD9/PLG/EFNA5/GCNT2/DLC1/PTPRJ/APOD/MYADM/NDNF/LIMCH1/ITGA3/FMN1/MACF1	23
GO:0046209	nitric oxide metabolic process	14/568	89/18986	0.157303370786517	5.25803133407185	7.07075287970475	3.80454729586797e-07	0.000101670931559578	8.24986045209266e-05	INSR/CD47/APOE/ASS1/PPARA/HSP90AA1/AGXT2/SIRPA/NOS1AP/DDAH1/NQO1/HSP90AB1/POR/RORA	14
GO:0141091	transforming growth factor beta receptor superfamily signaling pathway	32/568	397/18986	0.0806045340050378	2.6942916947529	5.99119776998359	4.12243420260121e-07	0.000104045658791207	8.44255238450845e-05	NPNT/CRIM1/LRP2/SNX25/PPARA/ITGB8/SULF1/HPGD/THBS1/NOTCH2/NLK/FOS/VASN/GCNT2/CLDN5/PPARG/ZEB2/FKBP8/TGFBR3/LOX/MAGI2/FSTL1/PRDM	32

ID	Description	GeneRatio	BgRatio	RichFactor	FoldEnrichment	zScore	pvalue	p.adjust	qvalue	geneID	Count
										16/HTRA1/DDX5/EGR1/HSP90AB1/HSPA5/ITGA3/ITGB5/BMPR1B/USP9Y	
GO:2001057	reactive nitrogen species metabolic process	14/568	91/18986	0.153846153846154	5.14247020585049	6.95608492916672	5.04024740041658e-07	0.00012051496810575	9.77891767105755e-05	INSR/CD47/APOE/ASS1/PPARA/HSP90AA1/AGXT2/SIRPA/NOS1AP/DDAH1/NQO1/HSP90AB1/POR/RORA	14
GO:0009261	ribonucleotide catabolic process	17/568	135/18986	0.125925925925926	4.20920709441836	6.571366136	5.96088966849814e-07	0.000135401608819935	0.000109868608521476	SLC4A4/INSR/SLC4A1/ALDOB/PPARA/PDE4B/PDE1A/DPYS/OGDH/DHTKD1/PDE7B/PDE4D/PRKAG2/IGF1/PFKFB2/GDA/NT5E	17
GO:0006809	nitric oxide biosynthetic process	13/568	81/18986	0.160493827160494	5.36467570857242	6.91296541936882	7.81140585534e-07	0.00016898674667052	0.00013712051782155 7	INSR/CD47/APOE/ASS1/HSP90AA1/AGXT2/SIRPA/NOS1AP/DDAH1/NQO1/HSP90AB1/POR/RORA	13
GO:0072009	nephron epithelium development	16/568	123/18986	0.130081300813008	4.3481048894996	6.54190845901527	8.19698808124284e-07	0.000169267803877665	0.00013734857540915	NPNT/CALB1/PAX8/PODXL/PTPRO/FGF1/SLC22A6/NPHS1/WT1/NOTCH2/STAT1/MAGI2/UMOD/ACAT1/MTSS1/FMN1	16
GO:0003018	vascular process in circulatory system	25/568	276/18986	0.0905797101449275	3.02772249438661	5.95915114176751	9.41248842064506e-07	0.00018334273241424	0.000148769361519671	SLC8A1/SLC4A4/LRP2/SLC13A3/INSR/SLIT2/APOE/SLC16A12/TJP1/DOCK4/CLDN5/PTPRJ/SLC1A1/DDAH1/ZEB2/CASR/MYLK3/SLC6A13/SLC6A6/PRKG1/F2R/KCNMB2/ATP8A1/SLC16A7/BCR	25
GO:0035725	sodium ion transmembrane transport	20/568	188/18986	0.106382978723404	3.55596344021576	6.18494687683825	9.68572656381636e-07	0.00018334273241424	0.000148769361519671	SLC8A1/SLC4A4/SLC12A3/SLC13A3/SLC17A1/SLC12A1/ATP1B1/FXYD2/SLC13A1/ANK3/UTRN/SCNN1A/SCN2A/SLC4A9/ATP1A1/CHP1/SLC6A13/SLC6A6/SLC6A16/	20

ID	Description	GeneRatio	BgRatio	RichFactor	FoldEnrichment	zScore	pvalue	p.adjust	qvalue	geneID	Count
										FGF13	
GO:0007160	cell-matrix adhesion	23/568	241/18986	0.0954356846473029	3.19003857167904	6.00863463791021	1.04013282736601e-06	0.000189012937388951	0.000153370322586769	PKHD1/ARHGAP6/NPNT/ITGB3/ITGB8/CDK6/UTRN/THBS1/EFNA5/BCAM/DLC1/PTPRJ/APOD/ANXA2/ITGAV/CCN2/LIMCH1/ITGA3/FMN1/ITGB5/BCR/MACF1/FREM1	23
GO:0040013	negative regulation of locomotion	32/568	416/18986	0.0769230769230769	2.57123510292524	5.69036495245793	1.14912137710463e-06	0.000200786862161013	0.000162924010632405	DPP4/SRGAP2/DACH1/SLIT2/APOE/PTPRO/SULF1/ROBO2/MCC/DCN/THBS1/NEDD9/MITF/SRGAP1/BST2/RIPOR2/CLDN5/DLC1/PPARG/PTPRJ/SEMA5A/APOD/ZEB2/ADGRG1/CLIC4/TGFBR3/MAGI2/LIMCH1/PRKG1/CDH1/ROBO1/BCR	32
GO:0032535	regulation of cellular component size	29/568	361/18986	0.0803324099722992	2.68519566150365	5.67693637570004	1.5428596687934e-06	0.000246624421559154	0.000200117873489615	SPP1/SLC12A3/SLIT2/SLC12A1/APOE/AKT3/CDC42EP3/NPHS1/EFNA5/HSP90AA1/ARHGAP28/SEMA3G/SEMA5A/MYO3B/MYADM/CYFIP2/EPS8/VAV3/NCK2/PLXNA4/CDH1/TMOD3/DAAM2/SCIN/FMN1/MAP3K13/FGF13/MACF1/KIRREL1	29
GO:0009154	purine ribonucleotide catabolic process	16/568	129/18986	0.124031007751938	4.14586745277869	6.29589112433783	1.56413024355447e-06	0.000246624421559154	0.000200117873489615	SLC4A4/INSR/SLC4A1/ALDOB/PPARA/PDE4B/PDE1A/OGDH/DHTKD1/PDE7B/PDE4D/PRKAG2/IGF1/PFKFB2/GDA/NT5E	16
GO:0006820	monoatomic anion transport	19/568	177/18986	0.107344632768362	3.5881077425002	6.07497319788029	1.5743139390745e-06	0.000246624421559154	0.000200117873489615	SLC4A4/SLC12A3/SLC17A1/SLC12A1/SLC4A1/SLC13A1/SLC22A6/CLIC5/SLC4A9/SLC1A1/CLC	19

ID	Description	GeneRatio	BgRatio	RichFactor	FoldEnrichment	zScore	pvalue	p.adjust	qvalue	geneID	Count
										NKB/CASR/APOL1/CLIC4/CLCN5/SLC39A14/SLC26A4/SLC26A6/PDZK1	
GO:0009166	nucleotide catabolic process	18/568	161/18986	0.111801242236025	3.7370746216429	6.12477751950712	1.64010361524528e-06	0.000248366357468643	0.000201531328441718	SLC4A4/INSR/SLC4A1/ALDOB/PPARA/PDE4B/PDE1A/FHIT/DPYS/OGDH/DHTKD1/PDE7B/PDE4D/PRKAG2/IGF1/PFKFB2/GDA/NT5E	18
GO:0007178	cell surface receptor protein serine/threonine kinase signaling pathway	32/568	425/18986	0.0752941176470588	2.51678541839271	5.55361998111749	1.82026826296634e-06	0.00024958972346799	0.00020252401433067	NPNT/CRIM1/LRP2/SNX25/PPARA/ITGB8/SULF1/HPGD/THBS1/NOTCH2/NLK/FOS/VASN/GCNT2/CLDN5/PPARG/ZEB2/FKBP8/TGFBR3/LOX/MAGI2/FSTL1/PRDM16/HTRA1/DDX5/EGR1/HSP90AB1/HSPA5/ITGA3/ITGB5/BMPR1B/USP9Y	32
GO:0051489	regulation of filopodium assembly	10/568	50/18986	0.2	6.68521126760563	7.06880045400134	1.85494667612 83e-06	0.00024958972346799	0.00020252401433067	SRGAP2C/NLGN1/TENM2/RIPOR2/MYO3B/AGRN/RAB3IP/DAAM2/MYO10/RHOQ	10
GO:1901888	regulation of cell junction assembly	24/568	268/18986	0.0895522388059701	2.99337817952491	5.77145893308196	1.89705959136905e-06	0.00024958972346799	0.00020252401433067	SRGAP2B/ARHGAP6/SRGAP2/PTPRD/SRGAP2C/ROBO2/NLGN1/THBS1/EFNA5/TJP1/IL1RAP/DOCK4/CLDN5/DLC1/PTPRJ/APOD/PDLIM5/CYFIP2/FARP1/AGRN/LIMCH1/FGFR1/FMN1/MACF1	24
GO:0071560	cellular response to transforming growth factor beta stimulus	26/568	306/18986	0.0849673202614379	2.84012243395011	5.69871885464746	1.91312387640955e-06	0.00024958972346799	0.00020252401433067	NPNT/SNX25/PPARA/ITGB8/HPGD/THBS1/NLK/FOS/WWOX/VASN/GCNT2/CLDN5/PPARG/FYN/SOX6/ZEB2/TGFBR3/LOX/PRDM16/HTRA1/SOX5/HSP90AB1/HSPA5/	26

ID	Description	GeneRatio	BgRatio	RichFactor	FoldEnrichment	zScore	pvalue	p.adjust	qvalue	geneID	Count
										ITGA3/ITGB5/USP9Y	
GO:0055007	cardiac muscle cell differentiation	16/568	131/18986	0.122137404580153	4.08257176647672	6.21718528000333	1.92287922548528e-06	0.00024958972346799	0.000202524001433067	SLC8A1/NEBL/PPARA/WT1/NRG1/SOX6/PDLIM5/MYLK3/SORBS2/FHOD3/SYNE1/EFNB2/PRKG1/PDGFRB/IGF1/MEIS1	16
GO:0006814	sodium ion transport	23/568	251/18986	0.0916334661354582	3.06294540149262	5.77771320229039	2.09726961557912e-06	0.00026466377398822	0.000214755502741464	SLC8A1/SLC4A4/SLC12A3/SLC5A12/SLC13A3/SLC17A1/SLC12A1/ATP1B1/FXYD2/SLC13A1/ANK3/UTRN/SCNN1A/SCN2A/SLC4A9/CLCNKB/ATP1A1/CHP1/SLC6A13/SLC6A6/UMOD/SLC6A16/FGF13	23
GO:0031032	actomyosin structure organization	21/568	216/18986	0.0972222222222222	3.24975547730829	5.83966980594275	2.2677890275761e-06	0.000278447717629141	0.000225940175663485	NEBL/ARHGAP6/EPB41L3/CD47/MYH9/IQGAP2/TJP1/SYNPO/ARHGAP28/DLC1/FRMD3/ZEB2/MYLK3/FHOD3/CCN2/LIMCH1/EPB41L1/PDGFRB/TMOD3/CSRP1/ITGB5	21
GO:0007411	axon guidance	22/568	236/18986	0.0932203389830508	3.11598830269754	5.74416441617081	2.62837646937888e-06	0.000314229323694428	0.000254974359993486	CNTN4/UNC5D/KLF7/SLIT2/UNC5C/PTPRO/ROBO2/EFNA5/APBB2/NOTCH2/SEMA3G/PTPRJ/FYN/SEMA5A/CYFIP2/EFNB1/EFNB2/PLXNA4/ROBO1/DSCAML1/BMPR1B/NFASC	22
GO:0097485	neuron projection guidance	22/568	237/18986	0.0928270042194093	3.10284067272835	5.72070721509338	2.81788517330708e-06	0.000320140442910328	0.000259770805535867	CNTN4/UNC5D/KLF7/SLIT2/UNC5C/PTPRO/ROBO2/EFNA5/APBB2/NOTCH2/SEMA3G/PTPRJ/FYN/SEMA5A/CYFIP2/EFNB1/EFNB2/PLXNA4/ROBO1/DSCAML1/BMPR1B/NFASC	22
GO:19	nucleoside phosphate	19/568	184/18986	0.10326086	3.45160364360073	5.8683	2.8187	0.0003	0.0002	SLC4A4/INSR/SLC4A1/	19

ID	Description	GeneRatio	BgRatio	RichFactor	FoldEnrichment	zScore	pvalue	p.adjust	qvalue	geneID	Count
01292	catabolic process			9565217		415090478	5802694544e-06	20140442910328	59770805535867	ALDOB/PPARA/PDE4B/PDE1A/FHIT/DPYS/OGDH/DHTKD1/PDE7B/PDE4D/PRKAG2/ACAT1/IGF1/PFKFB2/GDA/NT5E	
GO:0071559	response to transforming growth factor beta	26/568	313/18986	0.0830670926517572	2.77660531881384	5.5656297579585	2.90589318024341e-06	0.000321987139459654	0.000261269266167199	NPNT/SNX25/PPARA/ITGB8/HPGD/THBS1/NLK/FOS/WWOX/VASN/GCNT2/CLDN5/PPARG/FYN/SOX6/ZEB2/TGFBR3/LOX/PRDM16/HTRA1/SOX5/HSP90AB1/HSPA5/ITGA3/ITGB5/USP9Y	26
GO:0001656	metanephros development	13/568	92/18986	0.141304347826087	4.72324709124311	6.28654532836771	3.44075440226166e-06	0.000372174934511303	0.000301993030494244	CALB1/PAX8/SLC22A6/ROBO2/AQP2/WT1/STAT1/UMOD/ACAT1/PDGFRB/EGR1/FMN1/CTSH	13
GO:0046942	carboxylic acid transport	28/568	357/18986	0.0784313725490196	2.62165147749241	5.43194161212687	3.70562143452021e-06	0.000391503213419193	0.000317676530322883	PLA2R1/SLC5A12/LRP2/SLC13A3/SLC16A9/APOE/PPARA/SLC16A12/SERINC5/SLC22A6/THBS1/SLC36A2/PPARG/SLC1A1/CACNB4/CASR/SLC6A13/MPC1/SLC3A1/SLC6A6/SLC27A2/SLC26A4/PRKG1/UMOD/SLC16A7/SLC19A2/SLC26A6/LLGL2	28
GO:0006195	purine nucleotide catabolic process	16/568	138/18986	0.115942028985507	3.87548479281486	5.9535528888574	3.83553118193362e-06	0.000394674902654883	0.000320250126648841	SLC4A4/INSR/SLC4A1/ALDOB/PPARA/PDE4B/PDE1A/OGDH/DHTKD1/PDE7B/PDE4D/PRKAG2/IGF1/PFKFB2/GDA/NT5E	16
GO:0015849	organic acid transport	28/568	358/18986	0.0782122905027933	2.61432842867259	5.41512552008101	3.90939260829182e-06	0.000394674902654883	0.000320250126648841	PLA2R1/SLC5A12/LRP2/SLC13A3/SLC16A9/APOE/PPARA/SLC16A12/SERINC5/SLC22A6/THBS1/SLC36A2/PPARG/SLC1A1/CACNB4/CASR/SL	28

ID	Description	GeneRatio	BgRatio	RichFactor	FoldEnrichment	zScore	pvalue	p.adjust	qvalue	geneID	Count
										C6A13/MPC1/SLC3A1/SLC6A6/SLC27A2/SLC26A4/PRKG1/UMOD/SLC16A7/SLC19A2/SLC26A6/LLGL2	
GO:0098739	import across plasma membrane	20/568	206/18986	0.0970873786407767	3.24524818815807	5.68994941060683	4.02278777511253e-06	0.00039729401874644	0.00032237534984997 9	SLC8A1/SLC12A3/LRP2/SLC12A1/ATP1B1/KCNJ16/FXYD2/THBS1/SCNN1A/SLC28A1/CACNA1D/KCNJ3/FYN/SLC1A1/KCNJ15/ATP1A1/KCNJ1/SLC6A13/SLC6A6/SLC39A14	20
GO:0072523	purine-containing compound catabolic process	17/568	155/18986	0.109677419354839	3.66608359836438	5.85276591805146	4.13602091563123e-06	0.00039978602169601 5	0.00032439742993371 9	SLC4A4/INSR/SLC4A1/ALDOB/PPARA/PDE4B/PDE1A/OGDH/DHTKD1/PDE7B/PDE4D/PRKAG2/ACAT1/IGF1/PFKFB2/GDA/NT5E	17
GO:0003012	muscle system process	33/568	465/18986	0.0709677419354839	2.37217174011813	5.2609111312528	4.50981394734615e-06	0.00042082513363466 3	0.00034146914697883 1	SLC8A1/NPNT/PTGER3/ATP1B1/PPARA/SULF1/UTRN/APBB2/HSP90AA1/KCNIP1/PDE4B/DOCK4/SCN2A/CACNA1D/PPARG/KCNJ3/RYR3/NOS1AP/PDLIM5/PDE4D/ATP1A1/DSP/SORBS2/TNNI3K/DTNA/PRKG1/CHRM3/F2R/TMOD3/IGF1/TPM4/FGF13/MEIS1	33
GO:0034762	regulation of transmembrane transport	34/568	487/18986	0.0698151950718686	2.33364664372271	5.23587614865216	4.61228893726688e-06	0.00042082513363466 3	0.00034146914697883 1	SLC8A1/INSR/ATP1B1/KCNJ16/ITGB3/FXYD2/KCNIP4/ANK3/NLGN1/UTRN/THBS1/KCNIP1/PDE4B/DPP6/CACNA1D/KCNJ3/FYN/NOS1AP/KCNJ15/CACNB4/PDE4D/KCNJ1/CHP1/PRKAG2/DAPK1/CHRM3/F2R/TRPC6/FHL1/IGF1/SLC26A6/RHOQ/FGF13/PDZK1	34

ID	Description	GeneRatio	BgRatio	RichFactor	FoldEnrichment	zScore	pvalue	p.adjust	qvalue	geneID	Count
GO:0030048	actin filament-based movement	16/568	140/18986	0.114285714285714	3.82012072434608	5.88139514638378	4.63157752184308e-06	0.000420825133634663	0.000341469146978831	MYH9/PDE4B/SCN2A/CACNA1D/MYO1B/KCNJ3/MYO1D/NOS1AP/ZEB2/PDE4D/ATP1A1/DSP/PARVA/LIMCH1/MYO6/FGF13	16
GO:0030336	negative regulation of cell migration	29/568	383/18986	0.0757180156657963	2.53095465744861	5.31530826182374	4.96791815278665e-06	0.000436795009726093	0.0003544275459204 5	DPP4/SRGAP2/DACH1/SLIT2/APOE/SULF1/MCC/DCN/THBS1/NEDD9/MITF/SRGAP1/BST2/RIPOR2/CLDN5/DLC1/PPARG/PTPRJ/APOD/ZEB2/ADGRG1/CLIC4/TGFBR3/MAGI2/LIMCH1/PRKG1/CDH1/ROBO1/BCR	29
GO:0015698	inorganic anion transport	18/568	174/18986	0.103448275862069	3.4578678970374	5.71970869267926	4.99963471401207e-06	0.000436795009726093	0.00035442754592045	SLC4A4/SLC12A3/SLC17A1/SLC12A1/SLC4A1/SLC13A1/SLC22A6/CLIC5/SLC4A9/SLC1A1/CLCNKB/CASR/APOL1/CLIC4/CLCN5/SLC26A4/FGFR1/SLC26A6	18
GO:0007264	small GTPase-mediated signal transduction	34/568	491/18986	0.0692464358452139	2.31463526576977	5.18295085416015	5.50067325065463e-06	0.000469197879675833	0.00038072013036244 7	FGD4/ARHGAP6/SRGAP2/PLCE1/SLIT2/APOE/CDC42EP3/NOTCH2/SPATA13/DOCK4/ARHGAP28/RIPOR2/DLC1/RGL3/RHOBTB1/ADGRG1/EPS8/ITGAV/ARHGEF12/CHP1/ARHGAP10/RERG/FARP1/VAV3/ARHGAP8/SIPA1L3/F2R/ROBO1/IGF1/BCAR3/ARHGAP19/ITGA3/RHOQ/BCR	34
GO:0007044	cell-substrate junction assembly	13/568	96/18986	0.135416666666667	4.52644512910798	6.08296058627867	5.57708243506383e-06	0.000469197879675833	0.00038072013036244 7	ARHGAP6/DST/ITGB3/THBS1/EFNA5/TNS1/DLC1/PTPRJ/APOD/LIMCH1/FMN1/BCR/MACF1	13
GO:0007179	transforming growth factor beta receptor	22/568	250/18986	0.088	2.94149295774648	5.4265726212	6.7085001408	0.0005541221	0.0004496299	NPNT/SNX25/PPARA/ITGB8/HPGD/THBS1/NLK	22

ID	Description	GeneRatio	BgRatio	RichFactor	FoldEnrichment	zScore	pvalue	p.adjust	qvalue	geneID	Count
	signaling pathway					8033	2597e-06	11632225	99869331	/FOS/VASN/GCNT2/CLDN5/PPARG/ZEB2/TGFBR3/LOX/PRDM16/HTRA1/HSP90AB1/HSPA5/ITGA3/ITGB5/USP9Y	
GO:1990573	potassium ion import across plasma membrane	9/568	46/18986	0.195652173913043	6.53988058787508	6.60613685428951	7.38948535022984e-06	0.000599471999037396	0.000486428152189942	SLC12A3/SLC12A1/ATP1B1/KCNJ16/FXYD2/KCNJ3/KCNJ15/ATP1A1/KCNJ1	9
GO:0007409	axonogenesis	32/568	459/18986	0.0697167755991285	2.33035686888214	5.06675824158353	9.04727160149312e-06	0.00072108341904532	0.000585107020284929	CNTN4/UNC5D/SPP1/NTNG1/KLF7/SLIT2/APOE/UNC5C/PTPRO/ANK3/ROBO2/EFNA5/APBB2/NOTCH2/SEMA3G/PTPRJ/FYN/SEMA5A/CYFIP2/EFNB1/EFNB2/PRKG1/PLXNA4/CDH1/ROBO1/S100A6/DSCAML1/MAP3K13/BMPR1B/NFASC/FGF13/MACF1	32
GO:0032836	glomerular basement membrane development	5/568	11/18986	0.454545454545455	15.193661971831	8.26909807121983	9.36857982567873e-06	0.000733818243932042	0.000595440409247312	COL4A3/SULF1/NPHS1/WT1/COL4A4	5
GO:0006898	receptor-mediated endocytosis	23/568	275/18986	0.0836363636363636	2.7956338028169	5.2673703431449	9.64708030030954e-06	0.000742825183123834	0.000602748888700874	PLA2R1/DPP4/SGIP1/CBLB/CUBN/LRP2/INSR/EGF/APOE/ITGB3/LRP1B/GRK3/HIP1/NRG1/CD81/ANXA2/STON2/ITGAV/SYNE1/EFNB2/RAMP1/MAGI2/RAMP3	23
GO:0003014	renal system process	15/568	132/18986	0.113636363636364	3.79841549295775	5.66572700787118	9.81169961648592e-06	0.000742909189294926	0.000602817053630416	SLC12A3/SLC4A1/PTPRO/SULF1/SLC22A6/AQP2/ATP6V0A4/ADGRF5/CLCNKB/CLCN5/F2R/UMOD/ITGA3/BCR/KIRREL1	15
GO:2000146	negative regulation of cell motility	29/568	398/18986	0.0728643216080402	2.43556691910256	5.08284516603687	1.03852729877766e-05	0.000773447462024082	0.000627596652341563	DPP4/SRGAP2/DACH1/SLIT2/APOE/SULF1/MCC/DCN/THBS1/NEDD9/MITF/SRGAP1/BST2/RI	29

ID	Description	GeneRatio	BgRatio	RichFactor	FoldEnrichment	zScore	pvalue	p.adjust	qvalue	geneID	Count
										POR2/CLDN5/DLC1/PPARG/PTPRJ/APOD/ZEB2/ADGRG1/CLIC4/TGFBR3/MAGI2/LIMCH1/PRKG1/CDH1/ROBO1/BCR	
GO:0034331	cell junction maintenance	9/568	48/18986	0.1875	6.26738556338028	6.41662864863908	1.06656890751874e-05	0.000775894866034314	0.000629582543613001	ITGB3/PLEKHA7/ERC1/TJP1/F2R/PLXNA4/CHCHD10/MTSS1/KIRREL1	9
GO:0098661	inorganic anion transmembrane transport	15/568	133/18986	0.112781954887218	3.7698559779731	5.62925612428308	1.07597130883033e-05	0.000775894866034314	0.000629582543613001	SLC12A3/SLC17A1/SLC12A1/SLC4A1/SLC13A1/CLIC5/SLC4A9/SLC1A1/CLCNKB/CASR/APOL1/CLIC4/CLCN5/SLC26A4/SLC26A6	15
GO:0006936	muscle contraction	27/568	358/18986	0.0754189944134078	2.520959556	5.10192768807915	1.12780958089367e-05	0.000789302540916759	0.000640461901662591	SLC8A1/NPNT/PTGER3/ATP1B1/SULF1/UTRN/APBB2/HSP90AA1/KCNIP1/PDE4B/DOCK4/SCN2A/CACNA1D/KCNJ3/RYR3/NOS1AP/PDE4D/ATP1A1/DSP/TNNI3K/DTNA/PRKG1/CHRM3/F2R/TMOD3/TPM4/FGF13	27
GO:0035051	cardiocyte differentiation	17/568	167/18986	0.101796407185629	3.40265244159568	5.47657640144848	1.12931246224058e-05	0.000789302540916759	0.000640461901662591	SLC8A1/NEBL/PPARA/WT1/NRG1/SOX6/PDLIM5/MYLK3/SORBS2/FHOD3/SYNE1/EFNB2/TGFBR3/PRKG1/PDGFRB/IGF1/MEIS1	17
GO:0150115	cell-substrate junction organization	13/568	103/18986	0.12621359223301	4.2188226446055	5.75225854631082	1.22211035099617e-05	0.000832451680340394	0.000675474306232158	ARHGAP6/DST/ITGB3/THBS1/EFNA5/TNS1/DLC1/PTPRJ/APOD/LIMCH1/FMN1/BCR/MACF1	13
GO:0098656	monoatomic anion transmembrane transport	16/568	151/18986	0.105960264900662	3.54183378416193	5.50694885907691	1.22769673305759e-05	0.000832451680340394	0.000675474306232158	SLC4A4/SLC12A3/SLC12A1/SLC4A1/SLC13A1/CLIC5/SLC4A9/SLC1A1/CLCNKB/CASR/APOL1/CLIC4/CLCN5/SLC39A14/SLC26A4/SLC26A6	16
GO:00	regulation of cellular	28/568	381/18986	0.07349081	2.4565080773354	5.0433	1.2580	0.0008	0.0006	NPNT/CRIM1/LRP2/SN	28

ID	Description	GeneRatio	BgRatio	RichFactor	FoldEnrichment	zScore	pvalue	p.adjust	qvalue	geneID	Count
90287	response to growth factor stimulus			3648294		4621256919	8447927419e-05	37655533382802	79696856449268	X25/SLIT2/ITGB3/PPARA/FGF1/SULF1/DCN/THBS1/NOTCH2/VASN/CCBE1/PPARG/ZEB2/CYFIP2/ADGRG1/FKBP8/TGFBR3/LOX/FSTL1/PRDM16/HTRA1/ROBO1/HSP90AB1/HSPA5/ITGA3	
GO:0035850	epithelial cell differentiation involved in kidney development	9/568	49/18986	0.183673469387755	6.13947973555619	6.32586388286015	1.27224811365647e-05	0.000837655533382802	0.000679696856449268	PAX8/PODXL/PTPRO/NPHS1/WT1/NOTCH2/STAT1/MAGI2/MTSS1	9
GO:0051056	regulation of small GTPase mediated signal transduction	24/568	300/18986	0.08	2.67408450704225	5.13260764603046	1.31033989052929e-05	0.000850410588953512	0.000690046661147908	FGD4/ARHGAP6/SRGAP2/SLIT2/APOE/NOTCH2/SPATA13/ARHGAP28/RIPOR2/DLC1/ADGRG1/EPS8/ITGAV/ARHGEF12/ARHGAP10/VAV3/ARHGAP8/SIPA1L3/F2R/ROBO1/IGF1/ARHGAP19/ITGA3/BCR	24
GO:0072207	metanephric epithelium development	7/568	28/18986	0.25	8.35651408450704	6.8408971536953	1.4160610419818e-05	0.000906079621651171	0.000735218053227615	CALB1/PAX8/SLC22A6/WT1/STAT1/UMOD/ACAT1	7
GO:0043114	regulation of vascular permeability	9/568	50/18986	0.18	6.01669014084507	6.23758377234849	1.51080374815385e-05	0.000953275198314296	0.000773513848835493	SLIT2/APOE/TJP1/CLDN5/PTPRJ/DDAH1/ZEB2/MYLK3/BCR	9
GO:2000651	positive regulation of sodium ion transmembrane transporter activity	5/568	12/18986	0.416666666666667	13.9275234741784	7.86655791253266	1.56650864216342e-05	0.000974883391965537	0.000791047334514247	ATP1B1/FXYD2/ANK3/CHP1/FGF13	5
GO:0006885	regulation of pH	13/568	106/18986	0.122641509433962	4.09942200372044	5.61941212000869	1.67433816638301e-05	0.00102790787701054	0.000834072867521094	WDR72/SLC4A4/SLC4A1/RHCG/ATP6V0A4/ATP6V0D2/SLC4A9/ATP6V0C/CHP1/CLIC4/SLC26A4/ATP6V1A/SLC26A6	13
GO:0010975	regulation of neuron projection development	31/568	452/18986	0.0685840707964602	2.29249501433379	4.8839560693904	1.71889672915907e-	0.0010411930454092	0.0008448528204231	SPP1/PTPRD/NTNG1/SLIT2/APOE/PTPRO/VIM/CUX1/ROBO2/SPOCK1/	31

ID	Description	GeneRatio	BgRatio	RichFactor	FoldEnrichment	zScore	pvalue	p.adjust	qvalue	geneID	Count
							05	9	65	NLGN1/HECW1/TMEM30A/EFNA5/SEMA3G/FYN/SEMA5A/PDLIM5/NDNF/SYNE1/EFNB2/MAGI2/PLXNA4/CDH1/ROBO1/FGFR1/ITGA3/TOX/MAP3K13/FGF13/MACF1	
GO:0042692	muscle cell differentiation	30/568	432/18986	0.0694444444444444	2.32125391236307	4.8782900402579	1.85283423000693e-05	0.00110755604038441	0.000898701589125246	SLC8A1/NEBL/NPNT/PPARA/MYH9/NPHS1/TANC1/WT1/NOTCH2/RIPOR2/NRG1/SOX6/LAMB1/PDLIM5/CD81/CACNB4/MYLK3/SORBS2/FHOD3/SYNE1/EFNB2/LOX/PRKG1/PDGFRB/TMOD3/CSRP1/RCAN1/IGF1/RORA/MEIS1	30
GO:0072337	modified amino acid transport	8/568	40/18986	0.2	6.68521126760563	6.32085854835884	2.01741420466573e-05	0.00119027438075278	0.000965821537216594	LRP2/SLC13A3/SLC16A9/SLC16A12/SLC6A13/SLC3A1/SLC19A2/PDZK1	8
GO:0048738	cardiac muscle tissue development	21/568	252/18986	0.0833333333333333	2.78550469483568	5.01076739938309	2.46460201846209e-05	0.00142623018913707	0.00115728260304645	SLC8A1/NEBL/LRP2/PPARA/WT1/NOTCH2/NRG1/SOX6/PDLIM5/MYLK3/DSP/SORBS2/FHOD3/SYNE1/EFNB2/TGFBR3/PRKG1/PDGFRB/IGF1/FGFR1/MEIS1	21
GO:0045428	regulation of nitric oxide biosynthetic process	10/568	66/18986	0.151515151515152	5.06455399061033	5.80874795775541	2.48012733748246e-05	0.00142623018913707	0.00115728260304645	INSR/CD47/APOE/ASS1/HSP90AA1/AGXT2/SIRPA/NOS1AP/DDAH1/HSP90AB1	10
GO:0055006	cardiac cell development	12/568	95/18986	0.126315789473684	4.22223869532987	5.5290468717425	2.60859847115749e-05	0.00148135785680856	0.00120201471657809	SLC8A1/NEBL/PPARA/PDLIM5/MYLK3/SORBS2/FHOD3/TGFBR3/PRKG1/PDGFRB/IGF1/MEIS1	12
GO:0110053	regulation of actin filament organization	22/568	273/18986	0.0805860805860806	2.69367486973121	4.94991672503703	2.66644445297553e-	0.0014955132283787	0.0012135007763899	ARHGAP6/SLIT2/CD47/CDC42EP3/NPHS1/TJP1/SYNPO/ARHGAP28/DL	22

ID	Description	GeneRatio	BgRatio	RichFactor	FoldEnrichment	zScore	pvalue	p.adjust	qvalue	geneID	Count
							05	4		C1/SEMA5A/MYADM/CYFIP2/EPS8/CCN2/LIMCH1/NCK2/TMOD3/DAAM2/SCIN/MTSS1/FMN1/KIRREL1	
GO:0007229	integrin-mediated signaling pathway	13/568	111/18986	0.117117117117117	3.9147633549042	5.40853375302972	2.75784305492117e-05	0.00151603942771055	0.00123015630196656	DST/CD47/ITGB3/ITGB8/MYH9/NEDD9/LAMB1/ITGAV/CCN2/VAV3/ITGA3/ITGB5/PLPP3	13
GO:0051146	striated muscle cell differentiation	24/568	314/18986	0.0764331210191083	2.55485780927604	4.8788595740589	2.76978367818569e-05	0.00151603942771055	0.00123015630196656	SLC8A1/NEBL/PPARA/MYH9/NPHS1/TANC1/WT1/RIPOR2/NRG1/SOX6/PDLIM5/CD81/MYLK3/SORBS2/FHOD3/SYNE1/EFNB2/PRKG1/PDGFRB/TMOD3/CSRP1/RCAN1/IGF1/MEIS1	24
GO:0098659	inorganic cation import across plasma membrane	14/568	128/18986	0.109375	3.65597491197183	5.29467578766342	2.9805007157181e-05	0.00159298997076557	0.00129259609986932	SLC8A1/SLC12A3/SLC12A1/ATP1B1/KCNJ16/FXYD2/SCNN1A/CACNA1D/KCNJ3/FYN/KCNJ15/ATP1A1/KCNJ1/SLC39A14	14
GO:0099587	inorganic ion import across plasma membrane	14/568	128/18986	0.109375	3.65597491197183	5.29467578766342	2.9805007157181e-05	0.00159298997076557	0.00129259609986932	SLC8A1/SLC12A3/SLC12A1/ATP1B1/KCNJ16/FXYD2/SCNN1A/CACNA1D/KCNJ3/FYN/KCNJ15/ATP1A1/KCNJ1/SLC39A14	14
GO:1902476	chloride transmembrane transport	13/568	112/18986	0.116071428571429	3.87981011066398	5.36783456302941	3.03624885268829e-05	0.0016039161090422	0.00130146187051584	SLC12A3/SLC12A1/SLC4A1/CLIC5/SLC4A9/SLC1A1/CLCNKB/CASR/APOL1/CLIC4/CLCN5/SLC26A4/SLC26A6	13
GO:0080164	regulation of nitric oxide metabolic process	10/568	68/18986	0.147058823529412	4.91559652029826	5.68032276806775	3.23966992002953e-05	0.00169170349962002	0.00137269498607906	INSR/CD47/APOE/ASS1/HSP90AA1/AGXT2/SIRPA/NOS1AP/DDAH1/HSP90AB1	10
GO:0120034	positive regulation of plasma membrane bounded cell	13/568	113/18986	0.115044247787611	3.84547550791475	5.32760279254019	3.33889427530199e-	0.0017237041696246	0.0013986612143669	RAB11FIP3/PLCE1/CDC42EP3/NLGN1/HSP90AA1/TENM2/RIPOR2/MY	13

ID	Description	GeneRatio	BgRatio	RichFactor	FoldEnrichment	zScore	pvalue	p.adjust	qvalue	geneID	Count
	projection assembly						05	5	3	O3B/EPS8/AGRN/RP1/RAB3IP/RHOQ	
GO:0071805	potassium ion transmembrane transport	19/568	219/18986	0.0867579908675799	2.89997749051386	4.96628136034411	3.42934893663889e-05	0.00172781729364916	0.00140199871690614	SLC12A3/SLC12A1/ATP1B1/KCNJ16/FXYD2/HPN/KCNIP4/ANK3/KCNIP1/DPP6/CACNA1D/KCNT2/KCNJ3/NOS1AP/KCNJ15/ATP1A1/KCNJ1/FHL1/KCNMB2	19
GO:0006883	intracellular sodium ion homeostasis	6/568	22/18986	0.272727272727273	9.11619718309859	6.6889383166963	3.46095913982111e-05	0.00172781729364916	0.00140199871690614	SLC8A1/ATP1B1/FXYD2/SCNN1A/ATP1A1/UMOD	6
GO:0072234	metanephric nephron tubule development	6/568	22/18986	0.272727272727273	9.11619718309859	6.6889383166963	3.46095913982111e-05	0.00172781729364916	0.00140199871690614	CALB1/PAX8/SLC22A6/STAT1/UMOD/ACAT1	6
GO:1901606	alpha-amino acid catabolic process	12/568	98/18986	0.122448979591837	4.0929864903708	5.3908383366936	3.57431963585327e-05	0.0017650145767045	0.00143218162068171	GLDC/ALDH6A1/PAH/ACMSD/HMGCLL1/AGXT2/DDAH1/FTCD/HGD/AFMID/ACAT1/GLS	12
GO:0097205	renal filtration	7/568	32/18986	0.21875	7.31194982394366	6.27547094462424	3.62828580820481e-05	0.0017723981103951	0.00143817282403319	PTPRO/SULF1/ADGRF5/F2R/UMOD/ITGA3/KIRREL1	7
GO:0006935	chemotaxis	31/568	472/18986	0.0656779661016949	2.19535539508236	4.61823629299136	3.93954550977131e-05	0.00190397396286075	0.00154493710808725	DPP4/SLIT2/DEFB1/UNC5C/ITGB3/PTPRO/FGF1/ROBO2/THBS1/NEDD9/EFNA5/PDE4B/PIK3C2G/DOCK4/RIPOR2/SEMA3G/NRG1/PTPRJ/SEMA5A/CASR/ITGAV/EFNB2/LOX/PARVA/VAV3/CXCL14/PLXNA4/PDGFRB/ROBO1/FGFR1/F3	31
GO:0042330	taxis	31/568	474/18986	0.0654008438818565	2.18609229214952	4.59239481516 18	4.26591242286097e-05	0.00203614108905995	0.00165218116799424	DPP4/SLIT2/DEFB1/UNC5C/ITGB3/PTPRO/FGF1/ROBO2/THBS1/NEDD9/EFNA5/PDE4B/PIK3C2G/DOCK4/RIPOR2/SE	31

ID	Description	GeneRatio	BgRatio	RichFactor	FoldEnrichment	zScore	pvalue	p.adjust	qvalue	geneID	Count
										MA3G/NRG1/PTPRJ/SEMA5A/CASR/ITGAV/EFNB2/LOX/PARVA/VAV3/CXCL14/PLXNA4/PDGFRB/ROBO1/FGFR1/F3	
GO:0046434	organophosphate catabolic process	20/568	242/18986	0.0826446280991736	2.76248399487836	4.84573836127903	4.30265341293761e-05	0.00203614108905995	0.00165218116799424	SLC4A4/INSR/SLC4A1/ALDOB/PPARA/PDE4B/PDE1A/FHIT/NAPEPLD/DPYS/OGDH/DHTKD1/PDE7B/PDE4D/PRKAG2/ACAT1/IGF1/PFKFB2/GDA/NT5E	20
GO:0061318	renal filtration cell differentiation	6/568	23/18986	0.260869565217391	8.71984078383344	6.50544445597942	4.56461662256164e-05	0.00209465185013106	0.00169965841703465	PODXL/PTPRO/NPHS1/WT1/NOTCH2/MAGI2	6
GO:0072112	podocyte differentiation	6/568	23/18986	0.260869565217391	8.71984078383344	6.50544445597942	4.56461662256164e-05	0.00209465185013106	0.00169965841703465	PODXL/PTPRO/NPHS1/WT1/NOTCH2/MAGI2	6
GO:0097062	dendritic spine maintenance	6/568	23/18986	0.260869565217391	8.71984078383344	6.50544445597942	4.56461662256164e-05	0.00209465185013106	0.00169965841703465	INSR/APOE/TANC1/NEDD9/FYN/ITGA3	6
GO:0120032	regulation of plasma membrane bounded cell projection assembly	18/568	205/18986	0.0878048780487805	2.93497080041223	4.89159197871285	4.68339693192995e-05	0.00212766722617578	0.0017264480058546	RAB11FIP3/PLCE1/SLIT2/PODXL/SRGAP2C/CDC42EP3/NLGN1/HSP90AA1/TENM2/RIPOR2/MYO3B/EPS8/AGRN/RP1/RAB3IP/DAAM2/MYO10/RHOQ	18
GO:0072080	nephron tubule development	12/568	101/18986	0.118811881188119	3.97141263422117	5.25803402592602	4.836054770242e-05	0.00217526701200093	0.00176507178795075	NPNT/CALB1/PAX8/FGF1/SLC22A6/WT1/NOTCH2/STAT1/UMOD/ACAT1/MTSS1/FMN1	12
GO:0061005	cell differentiation involved in kidney development	9/568	58/18986	0.155172413793103	5.1868018455561	5.60792600173204	5.19218001076395e-05	0.00231255625381379	0.00187647207406557	PAX8/PODXL/PTPRO/NPHS1/WT1/NOTCH2/STAT1/MAGI2/MTSS1	9
GO:00	regulation of cell	18/568	207/18986	0.08695652	2.90661359461115	4.8436	5.3190	0.0023	0.0019	RAB11FIP3/PLCE1/SLIT	18

ID	Description	GeneRatio	BgRatio	RichFactor	FoldEnrichment	zScore	pvalue	p.adjust	qvalue	geneID	Count
60491	projection assembly			17391304		1774226242	538648317e-05	4503752771279	0282827763944	2/PODXL/SRGAP2C/CDC42EP3/NLGN1/HSP90AA1/TENM2/RIPOR2/MYO3B/EPS8/AGRN/RP1/RAB3IP/DAAM2/MYO10/RHOQ	
GO:0030032	lamellipodium assembly	10/568	72/18986	0.138888888888889	4.64250782472613	5.43792587688065	5.36834476958243e-05	0.00234503752771279	0.00190282827763944	SRGAP2/PLCE1/SLIT2/PTPRO/HSP90AA1/SPATA13/ABLIM1/VAV3/NCK2/ABLIM2	10
GO:0030111	regulation of Wnt signaling pathway	25/568	349/18986	0.0716332378223496	2.39441664312523	4.61716614772294	5.53303788973811e-05	0.00239396106029336	0.00194252618444741	GPC5/EGF/TRABD2B/APOE/BICC1/PTPRO/SULF1/MCC/HECW1/NLK/ZNRF3/WWOX/SEMA5A/ZEB2/ATP6V0C/CTNND1/IGFBP2/TLE4/DAAM2/EGR1/ITGA3/PPP2R3A/SHISA6/PLPP3/MACF1	25
GO:0043542	endothelial cell migration	22/568	287/18986	0.0766550522648084	2.56227609559798	4.68325472014721	5.67522241690185e-05	0.00243231466414954	0.00197364735888682	DPP4/SLIT2/EGF/APOE/ITGB3/AKT3/FGF1/MYH9/DCN/THBS1/PLG/CCBE1/PPARG/SEMA5A/ZEB2/EFNB2/TGFBR3/FSTL1/PIK3R3/ROBO1/FGFR1/PLPP3	22
GO:0010640	regulation of platelet-derived growth factor receptor signaling pathway	6/568	24/18986	0.25	8.35651408450704	6.33277203620356	5.9330511494998e-05	0.00247283040111721	0.00200652294790423	CBLB/HIP1/PTPRJ/APOD/LOX/F3	6
GO:0072170	metanephric tubule development	6/568	24/18986	0.25	8.35651408450704	6.33277203620356	5.9330511494998e-05	0.00247283040111721	0.00200652294790423	CALB1/PAX8/SLC22A6/STAT1/UMOD/ACAT1	6
GO:0072311	glomerular epithelial cell differentiation	6/568	24/18986	0.25	8.35651408450704	6.33277203620356	5.9330511494998e-05	0.00247283040111721	0.00200652294790423	PODXL/PTPRO/NPHS1/WT1/NOTCH2/MAGI2	6
GO:1901605	alpha-amino acid metabolic process	18/568	209/18986	0.0861244019138756	2.87879911045219	4.79621474704214	6.02999348645935e-05	0.00247353987974534	0.00200709863847636	GLDC/ASS1/SERINC5/ALDH6A1/PAH/ACMSD/HMGCLL1/AGXT2/BHMT2/DDAH1/FTCD/HG	18

ID	Description	GeneRatio	BgRatio	RichFactor	FoldEnrichment	zScore	pvalue	p.adjust	qvalue	geneID	Count
										D/AFMID/GLYAT/BHMT/ACAT1/GLS/ASPA	
GO:0006813	potassium ion transport	20/568	248/18986	0.0806451612903226	2.69564970467969	4.72018038658818	6.06877492197976e-05	0.00247353987974534	0.00200709863847636	SLC12A3/SLC12A1/ATP1B1/KCNJ16/FXYD2/HPN/KCNIP4/ANK3/KCNIP1/DPP6/CACNA1D/KCNT2/KCNJ3/NOS1AP/KCNJ15/ATP1A1/KCNJ1/CHP1/FHL1/KCNMB2	20
GO:0010232	vascular transport	11/568	88/18986	0.125	4.17825704225352	5.24784097273279	6.20698979288947e-05	0.00247353987974534	0.00200709863847636	SLC4A4/LRP2/SLC13A3/INSR/APOE/SLC16A12/SLC1A1/SLC6A13/SLC6A6/ATP8A1/SLC16A7	11
GO:0055013	cardiac muscle cell development	11/568	88/18986	0.125	4.17825704225352	5.24784097273279	6.20698979288947e-05	0.00247353987974534	0.00200709863847636	SLC8A1/NEBL/PPARA/PDLIM5/MYLK3/SORBS2/FHOD3/PRKG1/PDGFRB/IGF1/MEIS1	11
GO:0150104	transport across blood-brain barrier	11/568	88/18986	0.125	4.17825704225352	5.24784097273279	6.20698979288947e-05	0.00247353987974534	0.00200709863847636	SLC4A4/LRP2/SLC13A3/INSR/APOE/SLC16A12/SLC1A1/SLC6A13/SLC6A6/ATP8A1/SLC16A7	11
GO:0046031	ADP metabolic process	12/568	104/18986	0.115384615384615	3.85685265438787	5.13025259803283	6.46611027413769e-05	0.0025543946935137	0.00207270646956798	SLC4A4/INSR/SLC4A1/ALDOB/PPARA/OGDH/DHTKD1/AK4/PRKAG2/IGF1/PFKFB2/NT5E	12
GO:0006163	purine nucleotide metabolic process	29/568	440/18986	0.0659090909090909	2.20308098591549	4.4838833718189	6.53706435460671e-05	0.00256016235887744	0.00207738651268899	SLC4A4/INSR/SLC4A1/ATP1B1/ALDOB/PPARA/LDHB/PDE4B/ACMSD/PDE1A/FHIT/ALDH1L1/OGDH/DHTKD1/CACNB4/AK4/PDE7B/PDE4D/ATP6V0C/PRKAG2/AFMID/NQO1/IGF1/PFKFB2/ATP6V1A/GDA/NT5E/RORA/RHOQ	29
GO:0055078	sodium ion homeostasis	8/568	47/18986	0.170212765957447	5.68954150434522	5.65274019187834	6.86292724953691e-05	0.00266481012774754	0.00216230060529719	SLC8A1/SLC12A3/SLC12A1/ATP1B1/FXYD2/SCNN1A/ATP1A1/UMOD	8
GO:0034765	regulation of monoatomic ion	25/568	354/18986	0.0706214689265537	2.36059719901329	4.5379499337	6.9590557598	0.0026678549	0.0021647712	SLC8A1/ATP1B1/KCNJ16/ITGB3/FXYD2/KCNIP	25

ID	Description	GeneRatio	BgRatio	RichFactor	FoldEnrichment	zScore	pvalue	p.adjust	qvalue	geneID	Count
	transmembrane transport					2882	8906e-05	6287413	6869219	4/ANK3/UTRN/KCNIP1/PDE4B/DPP6/CACNA1D/KCNJ3/FYN/NOS1AP/KCNJ15/CACNB4/PDE4D/KCNJ1/CHP1/F2R/TRPC6/FHL1/FGF13/PDZK1	
GO:0170039	proteinogenic amino acid metabolic process	16/568	174/18986	0.0919540229885057	3.07366035292213	4.82561864312132	7.05797696997374e-05	0.00266785496287413	0.00216477126869219	GLDC/ASS1/SERINC5/PAH/ACMSD/HMGCLL1/AGXT2/BHMT2/DDAH1/FTCD/HGD/AFMID/GLYAT/BHMT/GLS/ASPA	16
GO:0030832	regulation of actin filament length	15/568	156/18986	0.0961538461538462	3.21404387865655	4.87620097602883	7.10426746455292e-05	0.00266785496287413	0.00216477126869219	SLIT2/CDC42EP3/NPHS1/ARHGAP28/SEMA5A/MYO3B/MYADM/CYFIP2/EPS8/NCK2/TMOD3/DAAM2/SCIN/FMN1/KIRREL1	15
GO:0061326	renal tubule development	12/568	105/18986	0.114285714285714	3.82012072434608	5.08871452483332	7.10566697133546e-05	0.00266785496287413	0.00216477126869219	NPNT/CALB1/PAX8/FGF1/SLC22A6/WT1/NOTCH2/STAT1/UMOD/ACAT1/MTSS1/FMN1	12
GO:0072243	metanephric nephron epithelium development	6/568	25/18986	0.24	8.02225352112676	6.1698431573957	7.6103689071644e-05	0.00282901704906255	0.00229554264068886	CALB1/PAX8/SLC22A6/STAT1/UMOD/ACAT1	6
GO:0002090	regulation of receptor internalization	10/568	75/18986	0.133333333333333	4.45680751173709	5.26752754976301	7.65945624113346e-05	0.00282901704906255	0.00229554264068886	CBLB/INSR/EGF/ITGB3/HIP1/NRG1/ANXA2/SYNE1/EFNB2/MAGI2	10
GO:0031623	receptor internalization	14/568	141/18986	0.0992907801418440	3.31889921086805	4.85346153968344	8.68827697219442e-05	0.00318313244231284	0.00258288166015491	CBLB/INSR/EGF/ITGB3/GRK3/HIP1/NRG1/CD81/ANXA2/SYNE1/EFNB2/RAMP1/MAGI2/RAMP3	14
GO:0043491	phosphatidylinositol 3-kinase/protein kinase B signal transduction	23/568	317/18986	0.0725552050473186	2.42523437152886	4.49379881061657	8.97921087201353e-05	0.0032634043993246	0.00264801654516138	WDR72/PKHD1/LRP2/INSR/EGF/PPARA/DCN/CALCR/THBS1/PIK3C2G/GCNT2/HIP1/PTPRJ/SEMA5A/LOX/MAGI2/VAV3/RAMP3/F2R/PIK3R3/PDGFRB/IGF1/FGFR1	23
GO:00	extracellular matrix	24/568	339/18986	0.07079646	2.36644646640907	4.4580	9.3061	0.0033	0.0027	WDR72/DPP4/NPNT/SP	24

ID	Description	GeneRatio	BgRatio	RichFactor	FoldEnrichment	zScore	pvalue	p.adjust	qvalue	geneID	Count
30198	organization			01769911		6318853326	487799956e-05	5538364345397	226510465785	OCK2/COL4A3/ITGB3/HPN/SULF1/PRDM5/NPHS1/WT1/PLG/APBB2/COL4A4/PPARG/LAMB1/MMP16/NDNF/ANXA2/TGFBR3/LOX/ADAMTS19/LOXL1/COL7A1	
GO:0001952	regulation of cell-matrix adhesion	13/568	125/18986	0.104	3.47630985915493	4.87793343981027	9.61121234593969e-05	0.00342317602883981	0.00277765966217478	PKHD1/ARHGAP6/ITGB3/CDK6/UTRN/THBS1/EFNA5/DLC1/PTPRJ/APOD/LIMCH1/FMN1/MACF1	13
GO:0099149	regulation of postsynaptic neurotransmitter receptor internalization	6/568	26/18986	0.230769230769231	7.71370530877573	6.01572557171655	9.64487192805404e-05	0.00342317602883981	0.00277765966217478	CBLB/ITGB3/HIP1/NRG1/SYNE1/EFNB2	6
GO:0043062	extracellular structure organization	24/568	340/18986	0.0705882352941176	2.35948632974317	4.44201169642769	9.73867205009009e-05	0.00342967342043095	0.00278293188253297	WDR72/DPP4/NPNT/SPOCK2/COL4A3/ITGB3/HPN/SULF1/PRDM5/NPHS1/WT1/PLG/APBB2/COL4A4/PPARG/LAMB1/MMP16/NDNF/ANXA2/TGFBR3/LOX/ADAMTS19/LOXL1/COL7A1	24
GO:0051893	regulation of focal adhesion assembly	9/568	63/18986	0.142857142857143	4.7751509054326	5.27069433579613	0.000101073802900083	0.00350517776011508	0.0028441981338376	ARHGAP6/THBS1/EFNA5/DLC1/PTPRJ/APOD/LIMCH1/FMN1/MACF1	9
GO:0090109	regulation of cell-substrate junction assembly	9/568	63/18986	0.142857142857143	4.7751509054326	5.27069433579613	0.000101073802900083	0.00350517776011508	0.0028441981338376	ARHGAP6/THBS1/EFNA5/DLC1/PTPRJ/APOD/LIMCH1/FMN1/MACF1	9
GO:0045229	external encapsulating structure organization	24/568	341/18986	0.0703812316715543	2.35256701499319	4.42601643265592	0.000101890426304947	0.00350672883866193	0.00284545672184948	WDR72/DPP4/NPNT/SPOCK2/COL4A3/ITGB3/HPN/SULF1/PRDM5/NPHS1/WT1/PLG/APBB2/COL4A4/PPARG/LAMB1/MMP16/NDNF/ANXA2/TGFBR3/LOX/ADAMTS19/LOXL1/COL7A1	24
GO:01	L-amino acid	16/568	181/18986	0.08839779	2.95478951054393	4.6404	0.0001	0.0038	0.0031	GLDC/ASS1/SERINC5/P	16

ID	Description	GeneRatio	BgRatio	RichFactor	FoldEnrichment	zScore	pvalue	p.adjust	qvalue	geneID	Count
70033	metabolic process			00552486		5806900311	12642625833876	4763495611504	2207736976839	AH/ACMSD/HMGCLL1/AGXT2/BHMT2/DDAH1/FTCD/HGD/AFMID/GLYAT/BHMT/GLS/ASPA	
GO:0072010	glomerular epithelium development	6/568	27/18986	0.2222222222222222	7.42801251956182	5.86960825718451	0.000120891165810491	0.00409192123414718	0.00332029800896311	PODXL/PTPRO/NPHS1/WT1/NOTCH2/MAGI2	6
GO:0009063	amino acid catabolic process	12/568	111/18986	0.108108108108108	3.61362771221926	4.8497569024283	0.000122091190132919	0.00409192123414718	0.00332029800896311	GLDC/ALDH6A1/PAH/ACMSD/HMGCLL1/AGXT2/DDAH1/FTCD/HGD/AFMID/ACAT1/GLS	12
GO:0006821	chloride transport	13/568	128/18986	0.1015625	3.39483384683099	4.77409207712702	0.000122496431398639	0.00409192123414718	0.00332029800896311	SLC12A3/SLC12A1/SLC4A1/CLIC5/SLC4A9/SLC1A1/CLCNKB/CASR/APOL1/CLIC4/CLCN5/SLC26A4/SLC26A6	13
GO:0051017	actin filament bundle assembly	15/568	164/18986	0.0914634146341463	3.05726125042941	4.64661531781721	0.000125172728881789	0.00415080078328443	0.00336807450283538	ARHGAP6/CD47/FMN2/NEDD9/TJP1/SYNPO/ARHGAP28/MYO1B/DLC1/ZEB2/EPS8/CCN2/LIMCH1/MTSS1/ITGB5	15
GO:0043269	regulation of monoatomic ion transport	30/568	480/18986	0.0625	2.08912852112676	4.24425730835712	0.000127247871220587	0.00418903680402265	0.00339910026708234	SLC8A1/ATP1B1/EGF/KCNJ16/ITGB3/FXYD2/KCNIP4/ANK3/UTRN/KCNIP1/PDE4B/DPP6/CACNA1D/KCNJ3/FYN/CLDN10/NOS1AP/KCNJ15/CACNB4/CASR/PDE4D/ATP1A1/KCNJ1/CHP1/F2R/TRPC6/FHL1/PDGFRB/FGF13/PDZK1	30
GO:0072203	cell proliferation involved in metanephros development	4/568	10/18986	0.4	13.3704225352113	6.8713240851188	0.000144174106680576	0.00463832478386038	0.00376366495431469	WT1/STAT1/PDGFRB/EGR1	4
GO:0045217	cell-cell junction maintenance	5/568	18/18986	0.277777777777778	9.28501564945227	6.17557000894625	0.000145984203486642	0.00463832478386038	0.00376366495431469	PLEKHA7/TJP1/F2R/MTSS1/KIRREL1	5
GO:00	relaxation of cardiac	5/568	18/18986	0.27777777	9.28501564945227	6.1755	0.0001	0.0046	0.0037	SLC8A1/ATP1B1/PDE4B	5

ID	Description	GeneRatio	BgRatio	RichFactor	FoldEnrichment	zScore	pvalue	p.adjust	qvalue	geneID	Count
55119	muscle			7777778		7000894625	45984203486642	3832478386038	6366495431469	/PDE4D/ATP1A1	
GO:0010812	negative regulation of cell-substrate adhesion	9/568	66/18986	0.136363636363636	4.5580985915493	5.084960853	0.000146000537990762	0.00463832478386038	0.00376366495431469	ARHGAP6/PTPRO/SPOCK1/THBS1/PLG/EFNA5/GCNT2/DLC1/APOD	9
GO:0072348	sulfur compound transport	9/568	66/18986	0.136363636363636	4.5580985915493	5.084960853	0.000146000537990762	0.00463832478386038	0.00376366495431469	SLC13A3/SLC13A1/SLC1A1/SLC6A13/SLC3A1/SLC6A6/SLC26A4/SLC19A2/SLC26A6	9
GO:0003156	regulation of animal organ formation	6/568	28/18986	0.214285714285714	7.16272635814889	5.7307818238601	0.000149999499098857	0.00473227586393129	0.00383989945792541	FGF1/SULF1/ROBO2/WT1/ROBO1/FGFR1	6
GO:0061572	actin filament bundle organization	15/568	168/18986	0.0892857142857143	2.98446931589537	4.53701830242588	0.000163708422541742	0.00512915423177333	0.00416193753714105	ARHGAP6/CD47/FMN2/NEDD9/TJP1/SYNPO/ARHGAP28/MYO1B/DLC1/ZEB2/EPS8/CCN2/LIMCH1/MTSS1/ITGB5	15
GO:0046032	ADP catabolic process	11/568	98/18986	0.112244897959184	3.7519042828399	4.79635819717601	0.000166084266821319	0.00514320943048429	0.00417334231392564	SLC4A4/INSR/SLC4A1/ALDOB/PPARA/OGDH/DHTKD1/PRKAG2/IGF1/PFKFB2/NT5E	11
GO:1903522	regulation of blood circulation	20/568	267/18986	0.0749063670411985	2.50382444479612	4.34579947235616	0.000166421260462512	0.00514320943048429	0.00417334231392564	SLC8A1/ATP1B1/HSP90AA1/KCNIP1/PDE4B/DOCK4/CACNA1D/THRB/KCNJ3/SLC1A1/NOS1AP/CASR/PDE4D/ATP1A1/DSP/TNNI3K/F2R/KCNMB2/EPAS1/FGF13	20
GO:0070252	actin-mediated cell contraction	12/568	115/18986	0.104347826086957	3.48793631353337	4.69947080767364	0.000171365588639465	0.00526022884587224	0.00426829510252779	PDE4B/SCN2A/CACNA1D/KCNJ3/NOS1AP/ZEB2/PDE4D/ATP1A1/DSP/PARVA/LIMCH1/FGF13	12
GO:0031345	negative regulation of cell projection organization	16/568	188/18986	0.0851063829787234	2.84477075217261	4.46399542442961	0.000175166253073774	0.00534080730095	0.00433367868784427	SPP1/SLIT2/APOE/SRGAP2C/PTPRO/VIM/SPOCK1/NLGN1/SEMA3G/FYN/SEMA5A/EFNB2/RAB3IP/CDH1/ITGA3/FGF13	16

ID	Description	GeneRatio	BgRatio	RichFactor	FoldEnrichment	zScore	pvalue	p.adjust	qvalue	geneID	Count
GO:0022604	regulation of cell morphogenesis	19/568	248/18986	0.0766129032258065	2.56086721944571	4.34498635830435	0.000180161887564966	0.00542241252500025	0.0043998954232772	FGD4/PTPRD/NTNG1/EPB41L3/HPN/CDC42EP3/CUX1/MYH9/EFNA5/DLC1/FYN/EPS8/PARVA/PLXNA4/MYO10/LARP4/RHOQ/MAP3K13/MACF1	19
GO:0150116	regulation of cell-substrate junction organization	9/568	68/18986	0.132352941176471	4.42403686826843	4.96722133669867	0.000184365134930277	0.00542241252500025	0.0043998954232772	ARHGAP6/THBS1/EFNA5/DLC1/PTPRJ/APOD/LIMCH1/FMN1/MACF1	9
GO:0003094	glomerular filtration	6/568	29/18986	0.206896551724138	6.91573579407479	5.59862266114124	0.00018438524235933	0.00542241252500025	0.0043998954232772	PTPRO/SULF1/ADGRF5/F2R/UMOD/KIRREL1	6
GO:0061448	connective tissue development	21/568	290/18986	0.0724137931034483	2.42050752792618	4.28081463910819	0.000184386895576455	0.00542241252500025	0.0043998954232772	BGN/ZBTB16/PPARGC1A/KLF7/ITGB3/ITGB8/SULF1/WT1/PPARG/SOX6/CASR/CCN2/LOX/SLC39A14/ACAT1/PDGFRB/SCIN/SOX5/FGFR1/EGR1/BMPR1B	21
GO:0009135	purine nucleoside diphosphate metabolic process	12/568	116/18986	0.103448275862069	3.4578678970374	4.66293987898782	0.000186049635622673	0.00542241252500025	0.0043998954232772	SLC4A4/INSR/SLC4A1/ALDOB/PPARA/OGDH/DHTKD1/AK4/PRKAG2/IGF1/PFKFB2/NT5E	12
GO:0009179	purine ribonucleoside diphosphate metabolic process	12/568	116/18986	0.103448275862069	3.4578678970374	4.66293987898782	0.000186049635622673	0.00542241252500025	0.0043998954232772	SLC4A4/INSR/SLC4A1/ALDOB/PPARA/OGDH/DHTKD1/AK4/PRKAG2/IGF1/PFKFB2/NT5E	12
GO:0001764	neuron migration	16/568	189/18986	0.0846560846560847	2.82971905507117	4.43945109932228	0.000186197744640114	0.00542241252500025	0.0043998954232772	UNC5D/SRGAP2/NTNG1/UNC5C/SRGAP2C/SPOCK1/APBB2/HSP90AA1/FYN/NDNF/ADGRG1/SH3RF1/PRKG1/FGFR1/ITGA3/FGF13	16
GO:1902903	regulation of supramolecular fiber organization	26/568	400/18986	0.065	2.17269366197183	4.16274532236031	0.000192141251892367	0.00555985800857977	0.00451142248828072	ARHGAP6/SLIT2/CD47/APOE/CDC42EP3/NPHS1/TJP1/SYNPO/ARHGAP28/DLC1/SEMA5A/MYADM/CYFIP2/EPS8/MY	26

ID	Description	GeneRatio	BgRatio	RichFactor	FoldEnrichment	zScore	pvalue	p.adjust	qvalue	geneID	Count
										LK3/CCN2/LIMCH1/NCK2/TMOD3/DAAM2/SCIN/MTSS1/FMN1/FGF13/ATXN7/KIRREL1	
GO:0014706	striated muscle tissue development	27/568	424/18986	0.0636792452830189	2.1285460403933	4.12712915290084	0.000202922084817821	0.00583465209700861	0.00473439800820792	SLC8A1/NEBL/LRP2/PPARA/NPHS1/WT1/NOTCH2/FOS/NRG1/SOX6/PDLIM5/MYLK3/DSP/SORBS2/FHOD3/SYNE1/EFNB2/TGFBR3/LOX/PRKG1/PDGFRB/RCAN1/IGF1/DDX5/FGFR1/EGR1/MEIS1	27
GO:0060537	muscle tissue development	28/568	447/18986	0.0626398210290828	2.09380218672212	4.109676442	0.000204249989953998	0.0058358975117045	0.00473540857212117	SLC8A1/NEBL/LRP2/PPARA/NPHS1/WT1/NOTCH2/FOS/NRG1/SOX6/PDLIM5/MYLK3/DSP/SORBS2/FHOD3/SYNE1/EFNB2/TGFBR3/LOX/PRKG1/PDGFRB/CSRP1/RCAN1/IGF1/DDX5/FGFR1/EGR1/MEIS1	28
GO:0008064	regulation of actin polymerization or depolymerization	14/568	153/18986	0.0915032679738562	3.05859339040781	4.48967361706382	0.00020752562559676	0.00587205431067761	0.00476474719834864	SLIT2/CDC42EP3/NPHS1/ARHGAP28/SEMA5A/MYADM/CYFIP2/EPS8/NCK2/TMOD3/DAAM2/SCIN/FMN1/KIRREL1	14
GO:0007162	negative regulation of cell adhesion	22/568	314/18986	0.0700636942675159	2.34195299183637	4.21080317640512	0.000208789469705915	0.00587205431067761	0.00476474719834864	ARHGAP6/CBLB/CR1/PODXL/ASS1/PPARA/PTPRO/SPOCK1/THBS1/PLG/EFNA5/RIPOR2/GCNT2/DLC1/SEMA5A/APOD/MYADM/HLA-E/PRKG1/PLXNA4/CDH1/MUC1	22
GO:0030833	regulation of actin filament polymerization	13/568	135/18986	0.0962962962962963	3.21880542514345	4.5433599031616	0.000209393087900016	0.00587205431067761	0.00476474719834864	SLIT2/CDC42EP3/NPHS1/ARHGAP28/MYADM/CYFIP2/EPS8/NCK2/TMOD3/DAAM2/SCIN/FMN1/KIRREL1	13
GO:00	pyridine nucleotide	15/568	172/18986	0.08720930	2.91506305273501	4.4306	0.0002	0.0058	0.0047	SLC4A4/INSR/SLC4A1/	15

ID	Description	GeneRatio	BgRatio	RichFactor	FoldEnrichment	zScore	pvalue	p.adjust	qvalue	geneID	Count
19362	metabolic process			23255814		2471142128	1214233577214	7660141105386	6843684129676	ALDOB/PPARA/LDHB/ACMSD/ALDH1L1/OGDH/DHTKD1/PRKAG2/AFMID/NQO1/IGF1/PFKFB2	
GO:0046496	nicotinamide nucleotide metabolic process	15/568	172/18986	0.0872093023255814	2.91506305273501	4.43062471142128	0.00021214233577214	0.00587660141105386	0.00476843684129676	SLC4A4/INSR/SLC4A1/ALDOB/PPARA/LDHB/ACMSD/ALDH1L1/OGDH/DHTKD1/PRKAG2/AFMID/NQO1/IGF1/PFKFB2	15
GO:0009137	purine nucleoside diphosphate catabolic process	11/568	101/18986	0.108910891089109	3.6404615813694	4.67240284270709	0.000217373939532874	0.00591719407602276	0.00480137485182101	SLC4A4/INSR/SLC4A1/ALDOB/PPARA/OGDH/DHTKD1/PRKAG2/IGF1/PFKFB2/NT5E	11
GO:0009181	purine ribonucleoside diphosphate catabolic process	11/568	101/18986	0.108910891089109	3.6404615813694	4.67240284270709	0.000217373939532874	0.00591719407602276	0.00480137485182101	SLC4A4/INSR/SLC4A1/ALDOB/PPARA/OGDH/DHTKD1/PRKAG2/IGF1/PFKFB2/NT5E	11
GO:0043410	positive regulation of MAPK cascade	29/568	472/18986	0.0614406779661017	2.05371956314156	4.07102854026109	0.000219077836313076	0.00591719407602276	0.00480137485182101	NPNT/INSR/EGF/APOE/ITGB3/FGF1/WWC1/CALCR/THBS1/NOTCH2/GCNT2/KL/NRG1/PTPRJ/CD81/CASR/CCN2/SH3RF1/PJA2/TAOK3/ARHGAP8/RAMP3/F2R/PDGFRB/ROBO1/IGF1/BCAR3/FGFR1/MAP3K13	29
GO:0072015	podocyte development	4/568	11/18986	0.363636363636364	12.1549295774648	6.49876028023429	0.000221205836311947	0.00591719407602276	0.00480137485182101	PODXL/NPHS1/NOTCH2/MAGI2	4
GO:0048041	focal adhesion assembly	10/568	85/18986	0.117647058823529	3.93247721623861	4.75838910699071	0.000221422626661648	0.00591719407602276	0.00480137485182101	ARHGAP6/THBS1/EFNA5/DLC1/PTPRJ/APOD/LIMCH1/FMN1/BCR/MACF1	10
GO:0086001	cardiac muscle cell action potential	10/568	85/18986	0.117647058823529	3.93247721623861	4.75838910699071	0.000221422626661648	0.00591719407602276	0.00480137485182101	SLC8A1/ATP1B1/ANK3/SCN2A/CACNA1D/KCNJ3/NOS1AP/ATP1A1/DSP/FGF13	10
GO:00	positive regulation of	7/568	42/18986	0.16666666	5.57100938967136	5.2078	0.0002	0.0059	0.0048	INSR/APOE/ASS1/HSP9	7

ID	Description	GeneRatio	BgRatio	RichFactor	FoldEnrichment	zScore	pvalue	p.adjust	qvalue	geneID	Count
45429	nitric oxide biosynthetic process			6666667		5819578335	24488585504041	6404470143192	3939074444538	0AA1/AGXT2/DDAH1/HSP90AB1	
GO:0048588	developmental cell growth	18/568	232/18986	0.0775862068965517	2.59340092277805	4.28825235929964	0.000226347459899061	0.00597846808326416	0.00485109427519285	SPP1/SLIT2/APOE/PPARA/EFNA5/SEMA3G/SEMA5A/PDLIM5/CYFIP2/SORBS2/PRKG1/PLXNA4/CDH1/IGF1/MAP3K13/FGF13/MACF1/MEIS1	18
GO:0086065	cell communication involved in cardiac conduction	8/568	56/18986	0.142857142857143	4.7751509054326	4.96833941560987	0.00024488547982371	0.00642638868648353	0.00521454943523647	SLC8A1/ATP1B1/CACNA1D/KCNJ3/PDE4D/ATP1A1/DSP/TNNI3K	8
GO:0097305	response to alcohol	20/568	275/18986	0.0727272727272727	2.43098591549296	4.19770042249213	0.000246135071857393	0.00642638868648353	0.00521454943523647	GRAMD1B/SLC12A3/SLIT2/PCK1/PPARA/FKBP5/HPGD/EFNA5/SCNN1A/FOS/ITPR2/CLDN5/FYN/EPS8/SH3RF1/TGFBR3/FOSB/CDH1/NQO1/USP46	20
GO:0003184	pulmonary valve morphogenesis	5/568	20/18986	0.25	8.35651408450704	5.78039384322023	0.000251385303348888	0.0064370501278913	0.00522320042352615	SLIT2/ROBO2/NOTCH2/ADAMTS19/ROBO1	5
GO:0043116	negative regulation of vascular permeability	5/568	20/18986	0.25	8.35651408450704	5.78039384322023	0.000251385303348888	0.0064370501278913	0.00522320042352615	SLIT2/APOE/CLDN5/PTPRJ/DDAH1	5
GO:0043171	peptide catabolic process	5/568	20/18986	0.25	8.35651408450704	5.78039384322023	0.000251385303348888	0.0064370501278913	0.00522320042352615	MME/ANPEP/TRHDE/CTSH/ERAP2	5
GO:0016055	Wnt signaling pathway	29/568	476/18986	0.0609243697478992	2.03646141555214	4.0217182366973	0.000252211076989798	0.0064370501278913	0.00522320042352615	GPC5/EGF/TRABD2B/APOE/BICC1/PTPRO/SULF1/MCC/HECW1/NLK/MITF/ZNRF3/WWOX/NDRG2/SEMA5A/ZEB2/ATP6V0C/CTNND1/IGFBP2/MAGI2/GRK5/TLE4/DAAM2/EGR1/ITGA3/PPP2R3A/SHISA6/PLPP3/	29

ID	Description	GeneRatio	BgRatio	RichFactor	FoldEnrichment	zScore	pvalue	p.adjust	qvalue	geneID	Count
										MACF1	
GO:0071456	cellular response to hypoxia	14/568	156/18986	0.0897435897435897	2.99977428674612	4.40429452318971	0.000254084321230658	0.00644863168352447	0.00523259801793452	FMN2/MALAT1/PCK1/GNGT1/FOS/SCN2A/VASN/PPARG/DDAH1/AK4/CASR/NDNF/EPAS1/RORA	14
GO:0009117	nucleotide metabolic process	30/568	500/18986	0.06	2.00556338028169	4.00157730519536	0.000257502472673408	0.00649907629641829	0.00527353017135833	SLC4A4/INSR/SLC4A1/ATP1B1/ALDOB/PPARA/LDHB/PDE4B/ACMSD/PDE1A/FHIT/ALDH1L1/DPYS/OGDH/DHTKD1/CACNB4/AK4/PDE7B/PDE4D/ATP6V0C/PRKAG2/AFMID/NQO1/IGF1/PFKFB2/ATP6V1A/GDA/NT5E/RORA/RHOQ	30
GO:1904407	positive regulation of nitric oxide metabolic process	7/568	43/18986	0.162790697674419	5.44145103177203	5.12027115621284	0.000261303705236078	0.00655857863473757	0.00532181201358967	INSR/APOE/ASS1/HSP90AA1/AGXT2/DDAH1/HSP90AB1	7
GO:0003298	physiological muscle hypertrophy	6/568	31/18986	0.193548387096774	6.46955929123126	5.35216534109633	0.000271649715198329	0.00667083597916762	0.00541290072617098	PPARA/PDLIM5/SORBS2/PRKG1/IGF1/MEIS1	6
GO:0003301	physiological cardiac muscle hypertrophy	6/568	31/18986	0.193548387096774	6.46955929123126	5.35216534109633	0.000271649715198329	0.00667083597916762	0.00541290072617098	PPARA/PDLIM5/SORBS2/PRKG1/IGF1/MEIS1	6
GO:0051491	positive regulation of filopodium assembly	6/568	31/18986	0.193548387096774	6.46955929123126	5.35216534109633	0.000271649715198329	0.00667083597916762	0.00541290072617098	NLGN1/TENM2/RIPOR2/MYO3B/AGRN/RHOQ	6
GO:0061049	cell growth involved in cardiac muscle cell development	6/568	31/18986	0.193548387096774	6.46955929123126	5.35216534109633	0.000271649715198329	0.00667083597916762	0.00541290072617098	PPARA/PDLIM5/SORBS2/PRKG1/IGF1/MEIS1	6
GO:0007266	Rho protein signal transduction	13/568	139/18986	0.0935251798561151	3.12617793089472	4.41818732200464	0.000279544621001026	0.00682780222154656	0.00554026747450816	ARHGAP6/APOE/CDC42EP3/RIPOR2/DLC1/RHOBTB1/ADGRG1/EPS8/ARHGEF12/F2R/ROBO1/ITGA3/BCR	13

ID	Description	GeneRatio	BgRatio	RichFactor	FoldEnrichment	zScore	pvalue	p.adjust	qvalue	geneID	Count
GO:0009191	ribonucleoside diphosphate catabolic process	11/568	104/18986	0.105769230769231	3.53544826652221	4.55308398090643	0.000281467378441351	0.00683800160566341	0.00554854353673859	SLC4A4/INSR/SLC4A1/ALDOB/PPARA/OGDH/DHTKD1/PRKAG2/IGF1/PFKFB2/NT5E	11
GO:0050920	regulation of chemotaxis	17/568	216/18986	0.0787037037037037	2.63075443401148	4.23293443454481	0.000283433806576406	0.00684914778338624	0.00555758785347466	DPP4/SLIT2/PTPRO/FGF1/ROBO2/THBS1/NEDD9/RIPOR2/PTPRJ/SEMA5A/CASR/EFNB2/PLXNA4/PDGFRB/ROBO1/FGFR1/F3	17
GO:0071453	cellular response to oxygen levels	15/568	178/18986	0.0842696629213483	2.81680250039563	4.27665922827257	0.000307874126433623	0.00736143240204184	0.00597326975496148	FMN2/MALAT1/PCK1/GNGT1/FOS/SCN2A/VASN/PPARG/DDAH1/AK4/CASR/NDNF/ATP6V1A/EPAS1/RORA	15
GO:0072524	pyridine-containing compound metabolic process	15/568	178/18986	0.0842696629213483	2.81680250039563	4.27665922827257	0.000307874126433623	0.00736143240204184	0.00597326975496148	SLC4A4/INSR/SLC4A1/ALDOB/PPARA/LDHB/ACMSD/ALDH1L1/OGDH/DHTKD1/PRKAG2/AFMID/NQO1/IGF1/PFKFB2	15
GO:0098742	cell-cell adhesion via plasma-membrane adhesion molecules	20/568	280/18986	0.0714285714285714	2.3875754527163	4.10774421952244	0.000311452534985435	0.00740800453632897	0.00601105967219066	CNTN4/UNC5D/PTPRD/NTNG1/ROBO2/PCDH15/SDK1/NLGN1/EFNA5/IL1RAP/CLDN5/CLDN11/CLDN10/MYADM/CDH16/UMOD/CDH1/ROBO1/ITGA3/DSCAML1	20
GO:0009259	ribonucleotide metabolic process	24/568	368/18986	0.0652173913043478	2.17996019595836	4.01405194187708	0.000319116829696664	0.00754078145507548	0.00611879853462801	SLC4A4/INSR/SLC4A1/ATP1B1/ALDOB/PPARA/PDE4B/PDE1A/DPYS/OGDH/DHTKD1/CACNB4/AK4/PDE7B/PDE4D/ATP6V0C/PRKAG2/IGF1/PFKFB2/ATP6V1A/GDA/NT5E/RORA/RHOQ	24
GO:0033627	cell adhesion mediated by integrin	10/568	89/18986	0.112359550561798	3.75573666719418	4.57608961666284	0.000323458711032951	0.00754078145507548	0.00611879853462801	DPP4/NPNT/PODXL/ITGB3/ITGB8/ITGAV/ITGA3/MUC1/ITGB5/PLPP3	10
GO:00	glomerular mesangial	4/568	12/18986	0.33333333	11.1420187793427	6.1715	0.0003	0.0075	0.0061	ITGB3/WT1/PDGFRB/E	4

ID	Description	GeneRatio	BgRatio	RichFactor	FoldEnrichment	zScore	pvalue	p.adjust	qvalue	geneID	Count
72110	cell proliferation			3333333		4385656715	23976211257103	4078145507548	1879853462801	GR1	
GO:0072310	glomerular epithelial cell development	4/568	12/18986	0.333333333333333	11.1420187793427	6.17154385656715	0.000323976211257103	0.00754078145507548	0.00611879853462801	PODXL/NPHS1/NOTCH2/MAGI2	4
GO:0008361	regulation of cell size	15/568	179/18986	0.0837988826815642	2.80106617357778	4.25162211275625	0.000326994045047297	0.00754078145507548	0.00611879853462801	SPP1/SLC12A3/SLC12A1/APOE/AKT3/EFNA5/HSP90AA1/SEMA3G/SEMA5A/VAV3/PLXNA4/CDH1/MAP3K13/FGF13/MACF1	15
GO:0015718	monocarboxylic acid transport	15/568	179/18986	0.0837988826815642	2.80106617357778	4.25162211275625	0.000326994045047297	0.00754078145507548	0.00611879853462801	PLA2R1/SLC5A12/SLC16A9/APOE/PPARA/SLC16A12/SLC22A6/THBS1/PPARG/CASR/SLC6A13/MPC1/SLC27A2/SLC16A7/SLC26A6	15
GO:0009150	purine ribonucleotide metabolic process	23/568	348/18986	0.0660919540229885	2.20919337866278	3.99801079980155	0.000350200058819917	0.00800864627625553	0.0064984369844751	SLC4A4/INSR/SLC4A1/ATP1B1/ALDOB/PPARA/PDE4B/PDE1A/OGDH/DHTKD1/CACNB4/AK4/PDE7B/PDE4D/ATP6V0C/PRKAG2/IGF1/PFKFB2/ATP6V1A/GDA/NT5E/RORA/RHOQ	23
GO:0060411	cardiac septum morphogenesis	9/568	74/18986	0.121621621621622	4.06533117624667	4.6396237917222	0.000352869326422518	0.00800864627625553	0.0064984369844751	LRP2/SLIT2/ROBO2/NOTCH2/DHRS3/TGFBR3/PARVA/ADAMTS19/ROBO1	9
GO:0048008	platelet-derived growth factor receptor signaling pathway	8/568	59/18986	0.135593220338983	4.53234662210552	4.77206842765931	0.000353441340686675	0.00800864627625553	0.0064984369844751	CBLB/ITGB3/HIP1/PTPRJ/APOD/LOX/PDGFRB/F3	8
GO:0051453	regulation of intracellular pH	10/568	90/18986	0.111111111111111	3.71400625978091	4.53216167392375	0.000354333678522421	0.00800864627625553	0.0064984369844751	SLC4A4/SLC4A1/ATP6V0A4/ATP6V0D2/SLC4A9/ATP6V0C/CHP1/CLIC4/ATP6V1A/SLC26A6	10
GO:0003015	heart process	19/568	262/18986	0.0725190839694656	2.42402698634555	4.0759400088	0.0003615291	0.0081308254	0.0065975765	SLC8A1/ATP1B1/HSP90AA1/KCNIP1/PDE4B/SC	19

ID	Description	GeneRatio	BgRatio	RichFactor	FoldEnrichment	zScore	pvalue	p.adjust	qvalue	geneID	Count
						8928	10134012	8187533	6951178	N2A/CACNA1D/THRB/KCNJ3/FYN/SLC1A1/NOS1AP/PDE4D/ATP1A1/DSP/TNNI3K/RAMP3/EPAS1/FGF13	
GO:0030178	negative regulation of Wnt signaling pathway	15/568	181/18986	0.0828729281767956	2.77011516613493	4.20206122523648	0.000368314645783476	0.00824262776253365	0.00668829603076864	TRABD2B/APOE/BICC1/PTPRO/MCC/HECW1/NLK/ZNRF3/WWOX/CTNND1/IGFBP2/TLE4/EGR1/PPP2R3A/SHISA6	15
GO:0097581	lamellipodium organization	10/568	91/18986	0.10989010989011	3.67319300417892	4.4888572868577	0.000387626153932965	0.00863228243783069	0.00700447260615709	SRGAP2/PLCE1/SLIT2/PTPRO/HSP90AA1/SPATA13/ABLIM1/VAV3/NCK2/ABLIM2	10
GO:0009134	nucleoside diphosphate catabolic process	11/568	108/18986	0.101851851851852	3.4045057381325	4.40066124822073	0.00039110285513286	0.00866722083350529	0.00703282258626587	SLC4A4/INSR/SLC4A1/ALDOB/PPARA/OGDH/DHTKD1/PRKAG2/IGF1/PFKFB2/NT5E	11
GO:0036294	cellular response to decreased oxygen levels	14/568	163/18986	0.0858895705521472	2.87094962412512	4.21278901678711	0.000398822193559381	0.00874933946480703	0.00709945591384182	FMN2/MALAT1/PCK1/GNGT1/FOS/SCN2A/VASN/PPARG/DDAH1/AK4/CASR/NDNF/EPAS1/RORA	14
GO:0006090	pyruvate metabolic process	12/568	126/18986	0.0952380952380952	3.18343393695506	4.31829909124968	0.00040222428924489	0.00874933946480703	0.00709945591384182	SLC4A4/INSR/SLC4A1/ALDOB/PCK1/PPARA/LDHB/OGDH/DHTKD1/PRKAG2/IGF1/PFKFB2	12
GO:0009185	ribonucleoside diphosphate metabolic process	12/568	126/18986	0.0952380952380952	3.18343393695506	4.31829909124968	0.00040222428924489	0.00874933946480703	0.00709945591384182	SLC4A4/INSR/SLC4A1/ALDOB/PPARA/OGDH/DHTKD1/AK4/PRKAG2/IGF1/PFKFB2/NT5E	12
GO:0036376	sodium ion export across plasma membrane	5/568	22/18986	0.227272727272727	7.59683098591549	5.43675745610868	0.000406363774394515	0.00874933946480703	0.00709945591384182	SLC8A1/SLC4A4/ATP1B1/FXYD2/ATP1A1	5
GO:0072111	cell proliferation involved in kidney development	5/568	22/18986	0.227272727272727	7.59683098591549	5.43675745610868	0.000406363774394515	0.00874933946480703	0.00709945591384182	ITGB3/WT1/STAT1/PDGFRB/EGR1	5
GO:1902307	positive regulation of sodium ion	5/568	22/18986	0.227272727272727	7.59683098591549	5.4367574561	0.0004063637	0.0087493394	0.0070994559	ATP1B1/FXYD2/ANK3/CHP1/FGF13	5

ID	Description	GeneRatio	BgRatio	RichFactor	FoldEnrichment	zScore	pvalue	p.adjust	qvalue	geneID	Count
	transmembrane transport					0868	74394515	6480703	1384182		
GO:0044282	small molecule catabolic process	24/568	375/18986	0.064	2.1392676056338	3.91304427852828	0.000419194458465931	0.00895546737582081	0.00726671379916458	GLDC/APOE/ALDOB/PCK1/PPARA/ALDH6A1/MIOX/PAH/ACMSD/HMGCLL1/AGXT2/ALDH1L1/CRYL1/DDAH1/PLIN5/FTCD/HGD/AFMID/SLC27A2/ACAT1/GLS/PFKFB2/GDA/ACSS2	24
GO:0042060	wound healing	27/568	444/18986	0.0608108108108108	2.03266558812333	3.86661682233661	0.000419879936396617	0.00895546737582081	0.00726671379916458	DST/SLC4A1/APOE/ITGB3/PPARA/FGF1/MYH9/THBS1/PLG/NOTCH2/PPARG/NRG1/PTPRJ/F5/NDNF/ANXA2/DSP/ITGAV/VAV3/PRKG1/F2R/CSRP1/IGF1/ITGB5/PLPP3/F3/MACF1	27
GO:0036293	response to decreased oxygen levels	22/568	331/18986	0.0664652567975831	2.22167141823752	3.93757662190369	0.000432893791305325	0.00918989015841165	0.00745693092548573	DPP4/FMN2/MALAT1/PCK1/PPARA/THBS1/GNGT1/FOS/SCN2A/VASN/ITPR2/PPARG/SLC1A1/DDAH1/ZEB2/AK4/CASR/NDNF/TGFBR3/EGR1/EPAS1/RORA	22
GO:0019693	ribose phosphate metabolic process	24/568	376/18986	0.0638297872340425	2.13357806412946	3.89879502719706	0.00043553465852585	0.0092029486217811	0.00746752692365987	SLC4A4/INSR/SLC4A1/ATP1B1/ALDOB/PPARA/PDE4B/PDE1A/DPYS/OGDH/DHTKD1/CACNB4/AK4/PDE7B/PDE4D/ATP6V0C/PRKAG2/IGF1/PFKFB2/ATP6V1A/GDA/NT5E/RORA/RHOQ	24
GO:0043113	receptor clustering	8/568	61/18986	0.131147540983607	4.38374509351189	4.64839308806902	0.000445483471133033	0.00936958985813596	0.00760274422957057	APOE/NLGN1/CD81/GRIK2/FARP1/MAGI2/AGRN/SHISA6	8
GO:0055075	potassium ion homeostasis	6/568	34/18986	0.176470588235294	5.89871582435791	5.02056293593219	0.000459866856841702	0.0095396124686386	0.00774070527594156	SLC12A3/SLC12A1/ATP1B1/FXYD2/ATP1A1/UMOD	6

ID	Description	GeneRatio	BgRatio	RichFactor	FoldEnrichment	zScore	pvalue	p.adjust	qvalue	geneID	Count
GO:0098884	postsynaptic neurotransmitter receptor internalization	6/568	34/18986	0.176470588235294	5.89871582435791	5.02056293593219	0.000459866856841702	0.0095396124686386	0.00774070527594156	CBLB/ITGB3/HIP1/NRG1/SYNE1/EFNB2	6
GO:0140239	postsynaptic endocytosis	6/568	34/18986	0.176470588235294	5.89871582435791	5.02056293593219	0.000459866856841702	0.0095396124686386	0.00774070527594156	CBLB/ITGB3/HIP1/NRG1/SYNE1/EFNB2	6
GO:0048259	regulation of receptor-mediated endocytosis	12/568	128/18986	0.09375	3.13369278169014	4.25350836659063	0.000464454146490866	0.00955562010443709	0.00775369431415334	SGIP1/CBLB/INSR/EGF/ITGB3/HIP1/NRG1/ANXA2/ITGAV/SYNE1/EFNB2/MAGI2	12
GO:0055001	muscle cell development	16/568	205/18986	0.0780487804878049	2.60886293369976	4.06719368563988	0.000464845265921329	0.00955562010443709	0.00775369431415334	SLC8A1/NEBL/PPARA/PDLIM5/CACNB4/MYLK3/SORBS2/FHOD3/LOX/PRKG1/PDGFRB/TMOD3/CSRP1/RCAN1/IGF1/MEIS1	16
GO:0060048	cardiac muscle contraction	13/568	147/18986	0.0884353741496599	2.95604579860113	4.18087321410665	0.000481352930822307	0.00985038903029613	0.00799287796936804	SLC8A1/ATP1B1/HSP90AA1/PDE4B/SCN2A/CACNA1D/KCNJ3/NOS1AP/PDE4D/ATP1A1/DSP/TNNI3K/FGF13	13
GO:0050807	regulation of synapse organization	21/568	312/18986	0.0673076923076923	2.24983071505959	3.90902139524902	0.000491906644267373	0.0100212192148281	0.00813149430363154	SRGAP2B/SRGAP2/PTPRD/APOE/SRGAP2C/PTPRO/ROBO2/NLGN1/TANC1/NEDD9/EFNA5/IL1RAP/DOCK4/FYN/CACNA2D3/PDLIM5/CYFIP2/FARP1/AGRN/SEPTIN11/FGFR1	21
GO:0022029	telencephalon cell migration	8/568	62/18986	0.129032258064516	4.31303952748751	4.588536881	0.000498301680341039	0.0101061809544167	0.00820043460786804	SRGAP2/SLIT2/SRGAP2C/OGDH/LAMB1/ADGRG1/ROBO1/FGF13	8
GO:0007416	synapse assembly	19/568	269/18986	0.0706319702602231	2.36094821718415	3.94782465629637	0.000500947338283918	0.010114683368106	0.00820733370138143	SRGAP2B/SPOCK2/SRGAP2/PTPRD/SRGAP2C/ROBO2/SDK1/NLGN1/EFNA5/IL1RAP/DOCK4/PDLIM5/CYFIP2/FARP1/AGRN/PLXNA4/CDH1/F	19

ID	Description	GeneRatio	BgRatio	RichFactor	FoldEnrichment	zScore	pvalue	p.adjust	qvalue	geneID	Count
										GFR1/FGF13	
GO:0030641	regulation of cellular pH	10/568	94/18986	0.106382978723404	3.55596344021576	4.36252400930013	0.000503492109578975	0.0101210825390145	0.00821252616555925	SLC4A4/SLC4A1/ATP6V0A4/ATP6V0D2/SLC4A9/ATP6V0C/CHP1/CLIC4/ATP6V1A/SLC26A6	10
GO:0031290	retinal ganglion cell axon guidance	5/568	23/18986	0.217391304347826	7.26653398652786	5.28075512466993	0.000506563109996986	0.0101379568665917	0.00822621846143957	SLIT2/PTPRO/ROBO2/EFNA5/BMPR1B	5
GO:0030041	actin filament polymerization	14/568	167/18986	0.0838323353293413	2.80218436366703	4.10787685569847	0.000509520745865033	0.0101524243353721	0.00823795776555562	ARHGAP6/SLIT2/CDC42EP3/NPHS1/ARHGAP28/MYADM/CYFIP2/EPS8/NCK2/TMOD3/DAAM2/SCIN/FMN1/KIRREL1	14
GO:0070482	response to oxygen levels	23/568	358/18986	0.0642458100558659	2.14748406640963	3.84913636007172	0.000520325930677464	0.0103224484850119	0.008375920060825	DPP4/FMN2/MALAT1/PCK1/PPARA/THBS1/GNGT1/FOS/SCN2A/VASN/ITPR2/PPARG/SLC1A1/DDAH1/ZEB2/AK4/CASR/NDNF/TGFBR3/EGR1/ATP6V1A/EPAS1/RORA	23
GO:0022409	positive regulation of cell-cell adhesion	22/568	336/18986	0.0654761904761905	2.18861083165661	3.86036294732558	0.000530044570549438	0.0104027965129374	0.00844111667187383	DPP4/MAGI1/ZBTB16/CD47/CR1/PODXL/PCK1/ANK3/TJP1/GCNT2/SIRPA/FYN/HLA-E/CD81/EFNB1/EFNB2/IGFBP2/NCK2/IGF1/MYO10/PLPP3/AP3D1	22
GO:0016054	organic acid catabolic process	18/568	249/18986	0.072289156626506	2.41634142202613	3.95071622572158	0.000531245606647913	0.0104027965129374	0.00844111667187383	GLDC/PCK1/PPARA/ALDH6A1/PAH/ACMSD/HMGCLL1/AGXT2/ALDH1L1/CRYL1/DDAH1/PLIN5/FTCD/HGD/AFMID/SLC27A2/ACAT1/GLS	18
GO:0046395	carboxylic acid catabolic process	18/568	249/18986	0.072289156626506	2.41634142202613	3.95071622572158	0.000531245606647913	0.0104027965129374	0.00844111667187383	GLDC/PCK1/PPARA/ALDH6A1/PAH/ACMSD/HMGCLL1/AGXT2/ALDH1L1/CRYL1/DDAH1/PLIN5/FTCD/HGD/AFMID/SLC27A2/ACAT1/GLS	18
GO:00	regulation of synapse	14/568	168/18986	0.08333333	2.78550469483568	4.0821	0.0005	0.0104	0.0084	SRGAP2B/SRGAP2/PTP	14

ID	Description	GeneRatio	BgRatio	RichFactor	FoldEnrichment	zScore	pvalue	p.adjust	qvalue	geneID	Count
51963	assembly			33333333		3289802458	40958229803516	61204926195	8851087306898	RD/SRGAP2C/ROBO2/NLGN1/EFNA5/IL1RAP/DOCK4/PDLIM5/CYFIP2/FARP1/AGRN/FGFR1	
GO:0090075	relaxation of muscle	6/568	35/18986	0.171428571428571	5.73018108651911	4.91874103203469	0.000541136508398815	0.010461204926195	0.00848851087306898	SLC8A1/ATP1B1/PDE4B/PDE4D/ATP1A1/PRKG1	6
GO:2000649	regulation of sodium ion transmembrane transporter activity	6/568	35/18986	0.171428571428571	5.73018108651911	4.91874103203469	0.000541136508398815	0.010461204926195	0.00848851087306898	ATP1B1/FXYD2/ANK3/UTRN/CHP1/FGF13	6
GO:0032412	regulation of monoatomic ion transmembrane transporter activity	15/568	188/18986	0.0797872340425532	2.66697258016182	4.03375756132745	0.000550157172538632	0.0105905255713687	0.00859344521958203	ATP1B1/FXYD2/ANK3/NLGN1/UTRN/CACNA1D/NOS1AP/CACNB4/CHP1/DAPK1/CHRM3/TRPC6/FHL1/FGF13/PDZK1	15
GO:0034113	heterotypic cell-cell adhesion	8/568	63/18986	0.126984126984127	4.24457858260675	4.52993334194444	0.00056080280696088	0.0106593785451575	0.00864931442592804	CD47/ITGB3/GCNT2/SIRPA/MYADM/DSP/ITGAV/PARVA	8
GO:0007605	sensory perception of sound	14/568	169/18986	0.0828402366863905	2.76902241853488	4.05657706403092	0.00057402926567432	0.010957205688901	0.00889097960367745	CDC14A/LRP2/COL4A3/HPN/PCDH15/ATP6V0A4/RIPOR2/CACNA1D/THRB/CLIC5/MYO3B/SLC26A4/FGFR1/MYO6	14
GO:0006096	glycolytic process	10/568	96/18986	0.104166666666667	3.4818808685446	4.28113381903513	0.000595645580436906	0.0112957354857329	0.00916567203935181	SLC4A4/INSR/SLC4A1/ALDOB/PPARA/OGDH/DHTKD1/PRKAG2/IGF1/PFKFB2	10
GO:0021795	cerebral cortex cell migration	7/568	49/18986	0.142857142857143	4.7751509054326	4.64659693077639	0.000596737071665395	0.0112957354857329	0.00916567203935181	SRGAP2/SLIT2/SRGAP2C/LAMB1/ADGRG1/ROBO1/FGF13	7
GO:0010977	negative regulation of neuron projection development	12/568	132/18986	0.0909090909090909	3.0387323943662	4.12765767544185	0.000613463162568167	0.0115353897940769	0.00936013417029263	SPP1/SLIT2/APOE/PTPRO/VIM/SPOCK1/NLGN1/SEMA3G/SEMA5A/EFNB2/CDH1/FGF13	12
GO:0048545	response to steroid hormone	22/568	340/18986	0.0647058823529412	2.16286246893123	3.79955912414741	0.0006209333522040	0.0115353897940769	0.0093601341702926	SPP1/ESRRG/SLC12A3/SLIT2/ASS1/PCK1/PPARA/CALCR/THBS1/SCNN	22

ID	Description	GeneRatio	BgRatio	RichFactor	FoldEnrichment	zScore	pvalue	p.adjust	qvalue	geneID	Count
							5		3	1A/PTGDS/FOS/LBH/SAFB2/ATP1A1/SH3RF1/IGFBP2/LOX/FOSB/DDX5/ESRRB/TRERF1	
GO:0003177	pulmonary valve development	5/568	24/18986	0.208333333333333	6.9637617370892	5.1338369131594	0.000624255899076458	0.0115353897940769	0.00936013417029263	SLIT2/ROBO2/NOTCH2/ADAMTS19/ROBO1	5
GO:0009074	aromatic amino acid family catabolic process	5/568	24/18986	0.208333333333333	6.9637617370892	5.1338369131594	0.000624255899076458	0.0115353897940769	0.00936013417029263	PAH/ACMSD/FTCD/HGD/AFMID	5
GO:0009214	cyclic nucleotide catabolic process	4/568	14/18986	0.285714285714286	9.55030181086519	5.62014006225127	0.000624632597257963	0.0115353897940769	0.00936013417029263	PDE4B/PDE1A/PDE7B/PDE4D	4
GO:0010745	negative regulation of macrophage derived foam cell differentiation	4/568	14/18986	0.285714285714286	9.55030181086519	5.62014006225127	0.000624632597257963	0.0115353897940769	0.00936013417029263	ITGB3/PPARA/PPARG/ITGAV	4
GO:0050803	regulation of synapse structure or activity	21/568	318/18986	0.0660377358490566	2.20738107892639	3.81300402601956	0.000630174033901647	0.0115906098624097	0.00940494126027517	SRGAP2B/SRGAP2/PTPRD/APOE/SRGAP2C/PTPRO/ROBO2/NLGN1/TANC1/NEDD9/EFNA5/IL1RAP/DOCK4/FYN/CACNA2D3/PDLIM5/CYFIP2/FARP1/AGRN/SEPTIN11/FGFR1	21
GO:0070371	ERK1 and ERK2 cascade	21/568	319/18986	0.0658307210031348	2.2004613890238	3.79720905881215	0.000656225472576293	0.0119762358180037	0.00971784882112419	PKHD1/NPNT/EGF/APOE/ITGB3/FGF1/CALCR/NOTCH2/GCNT2/NDRG2/SIRPA/NRG1/CASR/ITGAV/CCN2/ARHGAP8/RAMP3/F2R/PDGFRB/IGF1/CTSH	21
GO:0009132	nucleoside diphosphate metabolic process	12/568	133/18986	0.0902255639097744	3.01588478237848	4.09693928034381	0.000656412660947152	0.0119762358180037	0.00971784882112419	SLC4A4/INSR/SLC4A1/ALDOB/PPARA/OGDH/DHTKD1/AK4/PRKAG2/IGF1/PFKFB2/NT5E	12
GO:0030509	BMP signaling pathway	14/568	172/18986	0.0813953488372093	2.72072551588601	3.9810119992	0.0006837292	0.0123977673	0.0100598911	CRIM1/LRP2/SULF1/NOTCH2/PPARG/FKBP8/T	14

ID	Description	GeneRatio	BgRatio	RichFactor	FoldEnrichment	zScore	pvalue	p.adjust	qvalue	geneID	Count
						4962	11670818	047974	225831	GFBR3/FSTL1/HTRA1/DDX5/EGR1/ITGA3/BMPR1B/USP9Y	
GO:0051897	positive regulation of phosphatidylinositol 3-kinase/protein kinase B signal transduction	15/568	192/18986	0.078125	2.61141065140845	3.94099122046789	0.000684974596853211	0.0123977673047974	0.0100598911225831	INSR/EGF/DCN/CALCR/THBS1/GCNT2/HIP1/PTPRJ/SEMA5A/VAV3/RAMP3/F2R/PDGFRB/IGF1/FGFR1	15
GO:0021885	forebrain cell migration	8/568	65/18986	0.123076923076923	4.11397616468039	4.41629579357413	0.000687885204617359	0.012401041605463	0.01006254798066	SRGAP2/SLIT2/SRGAP2C/OGDH/LAMB1/ADGRG1/ROBO1/FGF13	8
GO:0008016	regulation of heart contraction	16/568	213/18986	0.0751173708920188	2.51087747140118	3.89412996143274	0.000705485892085945	0.0126680727578911	0.0102792244397128	SLC8A1/ATP1B1/HSP90AA1/KCNIP1/PDE4B/CACNA1D/THRB/KCNJ3/SLC1A1/NOS1AP/PDE4D/ATP1A1/DSP/TNNI3K/EPAS1/FGF13	16
GO:0060840	artery development	11/568	116/18986	0.0948275862068965	3.16971223895095	4.11626586486897	0.000719436689196328	0.0128677199961375	0.0104412237279964	LRP2/APOE/AKT3/ROBO2/HPGD/ADGRF5/EFNB2/LOX/PDGFRB/ROBO1/LOXL1	11
GO:0010092	specification of animal organ identity	6/568	37/18986	0.162162162162162	5.42044156832889	4.72641228686847	0.000736812714240188	0.0130755475030983	0.0106098607124553	LRP2/PAX8/FGF1/ROBO2/ROBO1/FGFR1	6
GO:0015701	bicarbonate transport	6/568	37/18986	0.162162162162162	5.42044156832889	4.72641228686847	0.000736812714240188	0.0130755475030983	0.0106098607124553	SLC4A4/SLC4A1/SLC4A9/SLC39A14/SLC26A4/SLC26A6	6
GO:0019722	calcium-mediated signaling	17/568	235/18986	0.0723404255319149	2.41805513934672	3.84125436425916	0.000744897411036093	0.0131675834176536	0.0106845411978226	SLC8A1/PLCE1/ATP1B1/DEFB1/CALCR/ITPR2/PTPRJ/RYR3/GRIK2/MCTP2/CHP1/RCAN2/RAMP3/CHRM3/PDGFRB/RCAN1/IGF1	17
GO:0061387	regulation of extent of cell growth	10/568	99/18986	0.101010101010101	3.37636932707355	4.16301791204406	0.000759679668803491	0.0132744008739149	0.0107712158347602	SPP1/APOE/EFNA5/SEMA3G/SEMA5A/PLXNA4/CDH1/MAP3K13/FGF13/MACF1	10
GO:20	negative regulation of	5/568	25/18986	0.2	6.68521126760563	4.9951	0.0007	0.0132	0.0107	PPARA/PPARG/PTPRJ/R	5

ID	Description	GeneRatio	BgRatio	RichFactor	FoldEnrichment	zScore	pvalue	p.adjust	qvalue	geneID	Count
00272	signaling receptor activity					0046628981	61293951577659	744008739149	712158347602	AMP3/GPRC5A	
GO:0006941	striated muscle contraction	15/568	194/18986	0.077319587628866	2.58448889211558	3.89548733150484	0.000762301647764483	0.0132744008739149	0.0107712158347602	SLC8A1/ATP1B1/HSP90AA1/PDE4B/SCN2A/CACNA1D/KCNJ3/RYR3/NOS1AP/PDE4D/ATP1A1/DSP/TNNI3K/DTNA/FGF13	15
GO:0006518	peptide metabolic process	8/568	66/18986	0.121212121212121	4.05164319248826	4.36117374824259	0.000762627917255511	0.0132744008739149	0.0107712158347602	MME/SLC1A1/CPM/ANPEP/PAM/TRHDE/CTSH/ERAP2	8
GO:0006937	regulation of muscle contraction	14/568	174/18986	0.0804597701149425	2.68945280880686	3.93152859356338	0.000766321791555769	0.0132877858741903	0.0107820767940068	SLC8A1/NPNT/ATP1B1/HSP90AA1/PDE4B/DOCK4/PDE4D/ATP1A1/DSP/TNNI3K/PRKG1/CHRM3/F2R/FGF13	14
GO:0099072	regulation of postsynaptic membrane neurotransmitter receptor levels	11/568	117/18986	0.0940170940170940	3.14262068135308	4.08246072634108	0.000773154324372979	0.0133552855347013	0.0108368478845474	CBLB/ITGB3/HSP90AA1/HIP1/NRG1/EPS8/SYNE1/EFNB2/CTNND1/SHISA6/GPC6	11
GO:0030900	forebrain development	25/568	416/18986	0.0600961538461538	2.00877742416035	3.65337541694799	0.00080257757111285	0.013811022369567	0.0112066453510255	SRGAP2/LRP2/SLIT2/SRGAP2C/ROBO2/CDK6/FOS/SCN2A/TACC1/DLC1/FYN/SEMA5A/OGDH/LAMB1/ZSWIM6/NDNF/ADGRG1/NOTCH2NLC/PRKG1/PLXNA4/CDH1/ROBO1/FGFR1/TOX/FGF13	25
GO:0051896	regulation of phosphatidylinositol 3-kinase/protein kinase B signal transduction	19/568	280/18986	0.0678571428571429	2.26819668008048	3.75433823913522	0.000813799392767599	0.0139512854390309	0.0113204586831068	PKHD1/INSR/EGF/PPARA/DCN/CALCR/THBS1/GCNT2/HIP1/PTPRJ/SEMA5A/LOX/MAGI2/VAV3/RAMP3/F2R/PDGFRB/IGF1/FGFR1	19
GO:0019364	pyridine nucleotide catabolic process	10/568	100/18986	0.1	3.34260563380282	4.12465306676126	0.0008219811275124	0.0139859934917198	0.0113486217565492	SLC4A4/INSR/SLC4A1/ALDOB/PPARA/OGDH/DHTKD1/PRKAG2/IGF1	10

ID	Description	GeneRatio	BgRatio	RichFactor	FoldEnrichment	zScore	pvalue	p.adjust	qvalue	geneID	Count
							77			/PFKFB2	
GO:0060419	heart growth	10/568	100/18986	0.1	3.34260563380282	4.12465306676126	0.000821981127512477	0.0139859934917198	0.0113486217565492	PPARA/WT1/NRG1/PDLIM5/SORBS2/TGFBR3/PRKG1/IGF1/FGFR1/MEIS1	10
GO:0072044	collecting duct development	4/568	15/18986	0.266666666666667	8.91361502347418	5.38435481387651	0.000831726805565965	0.014099010737635	0.0114403270742027	CALB1/PAX8/AQP2/UMOD	4
GO:0032368	regulation of lipid transport	13/568	156/18986	0.0833333333333333	2.78550469483568	3.9323880703506	0.000844190386396132	0.0141975587204662	0.0115202916317927	PLA2R1/SPP1/EGF/APOE/ITGB3/PPARA/PLTP/THBS1/TMEM30A/PPARG/ANXA2/ITGAV/ATP8A1	13
GO:0008154	actin polymerization or depolymerization	15/568	196/18986	0.076530612244898	2.55811655648175	3.85055048894666	0.00084691578543833	0.0141975587204662	0.0115202916317927	ARHGAP6/SLIT2/CDC42EP3/NPHS1/ARHGAP28/SEMA5A/MYADM/CYFIP2/EPS8/NCK2/TMOD3/DAAM2/SCIN/FMN1/KIRREL1	15
GO:0050954	sensory perception of mechanical stimulus	15/568	196/18986	0.076530612244898	2.55811655648175	3.85055048894666	0.00084691578543833	0.0141975587204662	0.0115202916317927	CDC14A/LRP2/COL4A3/HPN/PCDH15/ATP6V0A4/RIPOR2/CACNA1D/THRB/CLIC5/FYN/MYO3B/SLC26A4/FGFR1/MYO6	15
GO:0050922	negative regulation of chemotaxis	7/568	52/18986	0.134615384615385	4.49966143011918	4.43776960079083	0.000860351978856939	0.0143697758821584	0.0116600333976664	DPP4/SLIT2/PTPRO/ROBO2/THBS1/SEMA5A/ROBO1	7
GO:0090092	regulation of transmembrane receptor protein serine/threonine kinase signaling pathway	21/568	326/18986	0.0644171779141104	2.15321221809384	3.68825083684127	0.00086613691674404 2	0.0144134066401765	0.0116954366008777	NPNT/CRIM1/LRP2/SNX25/PPARA/SULF1/THBS1/NOTCH2/VASN/PPARG/ZEB2/FKBP8/TGFBR3/LOX/MAGI2/FSTL1/PRDM16/HTRA1/HSP90AB1/HSPA5/ITGA3	21
GO:0016049	cell growth	28/568	490/18986	0.0571428571428571	1.91006036217304	3.58416145439135	0.000873219854220973	0.0144782401376857	0.0117480442930536	SPP1/SLIT2/APOE/PPARA/HPN/SPOCK1/WT1/EFNA5/BST2/SEMA3G/NRG1/PTPRJ/SEMA5A/P	28

ID	Description	GeneRatio	BgRatio	RichFactor	FoldEnrichment	zScore	pvalue	p.adjust	qvalue	geneID	Count
										DLIM5/CYFIP2/SORBS2/RERG/PRKG1/PLXNA4/CDH1/SGK2/FHL1/IGF1/TSPYL2/MAP3K13/FGF13/MACF1/MEIS1	
GO:0022898	regulation of transmembrane transporter activity	15/568	197/18986	0.0761421319796954	2.54513119325088	3.8282904000496	0.000892123003848501	0.0146844739365353	0.0119153880986936	ATP1B1/FXYD2/ANK3/NLGN1/UTRN/CACNA1D/NOS1AP/CACNB4/CHP1/DAPK1/CHRM3/TRPC6/FHL1/FGF13/PDZK1	15
GO:0070374	positive regulation of ERK1 and ERK2 cascade	15/568	197/18986	0.0761421319796954	2.54513119325088	3.8282904000496	0.000892123003848501	0.0146844739365353	0.0119153880986936	NPNT/APOE/ITGB3/FGF1/CALCR/NOTCH2/GCNT2/NRG1/CASR/CCN2/ARHGAP8/RAMP3/F2R/PDGFRB/IGF1	15
GO:0035023	regulation of Rho protein signal transduction	9/568	84/18986	0.107142857142857	3.58136317907445	4.16383114000353	0.000903055972757243	0.01469873606697	0.0119269607855993	APOE/RIPOR2/DLC1/ADGRG1/EPS8/F2R/ROBO1/ITGA3/BCR	9
GO:0045216	cell-cell junction organization	16/568	218/18986	0.0733944954128440	2.45328853857087	3.78991379947695	0.000904415728980988	0.01469873606697	0.0119269607855993	PKHD1/EPB41L3/PTPRO/NPHS1/PLEKHA7/TJP1/CLDN5/CLDN11/CLDN10/DSP/EFNB2/TLN2/F2R/CDH1/MTSS1/KIRREL1	16
GO:0055002	striated muscle cell development	14/568	177/18986	0.0790960451977401	2.64386886289488	3.85859487954281	0.000905931344651462	0.01469873606697	0.0119269607855993	SLC8A1/NEBL/PPARA/PDLIM5/MYLK3/SORBS2/FHOD3/PRKG1/PDGFRB/TMOD3/CSRP1/RCAN1/IGF1/MEIS1	14
GO:1902905	positive regulation of supramolecular fiber organization	14/568	177/18986	0.0790960451977401	2.64386886289488	3.85859487954281	0.000905931344651462	0.01469873606697	0.0119269607855993	CD47/APOE/CDC42EP3/NPHS1/SYNPO/SEMA5A/MYLK3/CCN2/LIMCH1/NCK2/SCIN/MTSS1/FMN1/KIRREL1	14
GO:1905953	negative regulation of lipid localization	8/568	68/18986	0.117647058823529	3.93247721623861	4.25411990532959	0.000931683486242795	0.0150627689608577	0.0122223471392481	PLA2R1/EGF/APOE/ITGB3/PPARA/THBS1/PPARG/ITGAV	8
GO:0002064	epithelial cell development	16/568	219/18986	0.0730593607305936	2.44208630780114	3.7694164200	0.0009494495	0.0152955644	0.0124112438	PKHD1/MAGI1/TFCP2L1/PODXL/VIM/NPHS1/C	16

ID	Description	GeneRatio	BgRatio	RichFactor	FoldEnrichment	zScore	pvalue	p.adjust	qvalue	geneID	Count
						3524	18714428	096441	019333	DK6/NOTCH2/TJP1/CLDN5/MYADM/PDE4D/CLIC4/MAGI2/SIPA1L3/EPAS1	
GO:0086003	cardiac muscle cell contraction	9/568	85/18986	0.105882352941176	3.53922949461475	4.12028502742208	0.000983775711110665	0.0157369473787879	0.0127693941449576	PDE4B/SCN2A/CACNA1D/KCNJ3/NOS1AP/PDE4D/ATP1A1/DSP/FGF13	9
GO:0097061	dendritic spine organization	9/568	85/18986	0.105882352941176	3.53922949461475	4.12028502742208	0.000983775711110665	0.0157369473787879	0.0127693941449576	PTPRD/INSR/APOE/NLGN1/TANC1/NEDD9/FYN/PDLIM5/ITGA3	9
GO:0045807	positive regulation of endocytosis	13/568	159/18986	0.0817610062893082	2.73294800248029	3.853468089	0.00100729018326435	0.0160004171418529	0.012983180794228	SGIP1/INSR/CD47/EGF/APOE/NLGN1/HIP1/SIRPA/PTPRJ/ANXA2/SYNE1/MAGI2/BCR	13
GO:1903825	organic acid transmembrane transport	13/568	159/18986	0.0817610062893082	2.73294800248029	3.853468089	0.00100729018326435	0.0160004171418529	0.012983180794228	SLC5A12/LRP2/SLC13A3/SLC16A9/SLC16A12/THBS1/SLC36A2/SLC1A1/SLC6A13/MPC1/SLC3A1/SLC6A6/SLC16A7	13
GO:0008360	regulation of cell shape	12/568	140/18986	0.0857142857142857	2.86509054325956	3.88966840107053	0.00103334098577833	0.0163192627799267	0.0132419009593251	FGD4/EPB41L3/HPN/CDC42EP3/MYH9/DLC1/FYN/EPS8/PARVA/PLXNA4/MYO10/RHOQ	12
GO:0072526	pyridine-containing compound catabolic process	10/568	103/18986	0.0970873786407767	3.24524818815807	4.01241370057035	0.00103454714519456	0.0163192627799267	0.0132419009593251	SLC4A4/INSR/SLC4A1/ALDOB/PPARA/OGDH/DHTKD1/PRKAG2/IGF1/PFKFB2	10
GO:0043603	amide metabolic process	27/568	472/18986	0.0572033898305085	1.91208373120076	3.52382078753083	0.00105588260021565	0.0165981821895491	0.0134682238789116	ST6GALNAC3/DPP4/ACSM2B/ACSM2A/DHFR/APOE/ALDOB/ASS1/SLC16A12/ITGB8/MME/NAPEPLD/OGDH/SLC1A1/ELOVL7/PAM/FTCD/GLYAT/SLC27A2/BACE2/ACAT1/ACSF2/IGF1/GDA/CTSH/PLPP3/ACSS2	27
GO:0017015	regulation of transforming growth factor beta receptor	14/568	180/18986	0.0777777777777778	2.59980438184664	3.78715563407246	0.0010663808007369	0.01670540681982	0.0135552289080968	NPNT/SNX25/PPARA/THBS1/VASN/PPARG/ZEB2/TGFBR3/LOX/PRDM	14

ID	Description	GeneRatio	BgRatio	RichFactor	FoldEnrichment	zScore	pvalue	p.adjust	qvalue	geneID	Count
	signaling pathway						1			16/HTRA1/HSP90AB1/HSPA5/ITGA3	
GO:0051895	negative regulation of focal adhesion assembly	4/568	16/18986	0.25	8.35651408450704	5.16959631325183	0.00108289853427856	0.0168479727439298	0.0136709108400992	ARHGAP6/THBS1/DLC1/APOD	4
GO:0150118	negative regulation of cell-substrate junction organization	4/568	16/18986	0.25	8.35651408450704	5.16959631325183	0.00108289853427856	0.0168479727439298	0.0136709108400992	ARHGAP6/THBS1/DLC1/APOD	4
GO:0052652	cyclic purine nucleotide metabolic process	6/568	40/18986	0.15	5.01390845070423	4.46269212325137	0.00112775719926854	0.0173943557969045	0.0141142611538305	PDE4B/PDE1A/CACNB4/PDE7B/PDE4D/RORA	6
GO:0099590	neurotransmitter receptor internalization	6/568	40/18986	0.15	5.01390845070423	4.46269212325137	0.00112775719926854	0.0173943557969045	0.0141142611538305	CBLB/ITGB3/HIP1/NRG1/SYNE1/EFNB2	6
GO:0048645	animal organ formation	8/568	70/18986	0.114285714285714	3.82012072434608	4.15107233890462	0.00112950362317562	0.0173943557969045	0.0141142611538305	LRP2/PAX8/FGF1/SULF1/ROBO2/WT1/ROBO1/FGFR1	8
GO:0030099	myeloid cell differentiation	26/568	451/18986	0.0576496674057650	1.92700103057369	3.49889111085252	0.00116206941804987	0.0178354100209479	0.0144721447439924	ZBTB16/SLC4A1/ITGB8/MYH9/CALCR/CDK6/NEDD9/NOTCH2/MITF/FOS/ADGRF5/PPARG/PTPRJ/RHEX/CD81/ANXA2/TGFBR3/STAT1/LOX/PTBP3/PRDM16/TMOD3/SCIN/EPAS1/TPM4/MEIS1	26
GO:0006520	amino acid metabolic process	19/568	289/18986	0.0657439446366782	2.19756079730981	3.60262254416641	0.00118266058106586	0.0180903266659334	0.0146789911568054	GLDC/ASS1/SERINC5/ALDH6A1/PAH/ACMSD/HMGCLL1/AGXT2/BHMT2/DDAH1/PEPD/FTCD/HGD/AFMID/GLYAT/BHMT/ACAT1/GLS/ASPA	19
GO:0001823	mesonephros development	10/568	105/18986	0.0952380952380952	3.18343393695506	3.93985686253683	0.0011997748672414	0.0182905275901944	0.0148414397212278	NPNT/ZBTB16/CALB1/SLIT2/PAX8/FGF1/ROBO2/WT1/FGFR1/FMN1	10

ID	Description	GeneRatio	BgRatio	RichFactor	FoldEnrichment	zScore	pvalue	p.adjust	qvalue	geneID	Count
							6				
GO:0016485	protein processing	17/568	246/18986	0.0691056910569106	2.30993072254666	3.63152737733264	0.00123309490595396	0.0186731671924961	0.0151519240724588	DPP4/MME/HPN/MYH9/THBS1/TMPRSS2/PLG/CCBE1/MMP16/CYFIP2/CPM/ANXA2/BACE2/TMEM98/CTSH/F3/GAS1	17
GO:0099173	postsynapse organization	17/568	246/18986	0.0691056910569106	2.30993072254666	3.63152737733264	0.00123309490595396	0.0186731671924961	0.0151519240724588	PTPRD/INSR/APOE/ITGB3/NLGN1/TANC1/NEDD9/IL1RAP/DOCK4/FYN/NOS1AP/PDLIM5/CYFIP2/FARP1/FGFR1/ITGA3/SHISA6	17
GO:0170040	proteinogenic amino acid catabolic process	8/568	71/18986	0.112676056338028	3.76631620710177	4.10096494524883	0.00124024987152412	0.0187191201539338	0.0151892115757212	GLDC/PAH/HMGCLL1/AGXT2/DDAH1/FTCD/HGD/GLS	8
GO:1903844	regulation of cellular response to transforming growth factor beta stimulus	14/568	183/18986	0.0765027322404372	2.55718463788194	3.7171519684433	0.00125004976883046	0.0188045566218437	0.0152585370876413	NPNT/SNX25/PPARA/THBS1/VASN/PPARG/ZEB2/TGFBR3/LOX/PRDM16/HTRA1/HSP90AB1/HSPA5/ITGA3	14
GO:0032409	regulation of transporter activity	16/568	225/18986	0.0711111111111111	2.37696400625978	3.64874984531882	0.00126157812731952	0.0189084217303962	0.0153428161080315	ATP1B1/FXYD2/ANK3/NLGN1/UTRN/CACNA1D/NOS1AP/CACNB4/CHP1/DAPK1/CHRM3/TRPC6/SGK2/FHL1/FGF13/PDZK1	16
GO:0050678	regulation of epithelial cell proliferation	24/568	406/18986	0.0591133004926108	1.9759245125928	3.49071685239513	0.00126738094004566	0.0189084217303962	0.0153428161080315	PKHD1/COL4A3/EGF/APOE/ITGB3/AKT3/HPN/FGF1/SULF1/MCC/CDK6/THBS1/NOTCH2/PPARG/SEMA5A/LAMB1/EFNB2/TGFBR3/STAT1/ROBO1/IGF1/MTSS1/FGFR1/F3	24
GO:0018108	peptidyl-tyrosine phosphorylation	15/568	204/18986	0.0735294117647059	2.45779826014913	3.67620589774477	0.00126944059603144	0.0189084217303962	0.0153428161080315	INSR/EGF/ITGB3/NEDD9/EFNA5/NRG1/PTPRJ/FYN/PARP14/PRLR/PDGFRB/IGF1/FGFR1/GPRC5A/PLPP3	15
GO:00	cyclic nucleotide	6/568	41/18986	0.14634146	4.89161800068705	4.3805	0.0012	0.0190	0.0154	PDE4B/PDE1A/CACNB4	6

ID	Description	GeneRatio	BgRatio	RichFactor	FoldEnrichment	zScore	pvalue	p.adjust	qvalue	geneID	Count
09187	metabolic process			3414634		9457902225	8817790152391	006240474777	176316170087	/PDE7B/PDE4D/RORA	
GO:0072210	metanephric nephron development	6/568	41/18986	0.146341463414634	4.89161800068705	4.38059457902225	0.00128817790152391	0.0190006240474777	0.0154176316170087	PAX8/WT1/STAT1/PDGFRB/EGR1/FMN1	6
GO:1904861	excitatory synapse assembly	6/568	41/18986	0.146341463414634	4.89161800068705	4.38059457902225	0.00128817790152391	0.0190006240474777	0.0154176316170087	SRGAP2/PTPRD/SRGAP2C/NLGN1/IL1RAP/FGFR1	6
GO:0034698	response to gonadotropin	5/568	28/18986	0.178571428571429	5.96893863179074	4.6206664940249	0.00130787680057678	0.0192287517961822	0.0156027407788107	PAX8/WT1/EFNA5/TGFBR3/ITGA3	5
GO:0071772	response to BMP	14/568	184/18986	0.0760869565217391	2.54328689528475	3.69412672868582	0.00131686910709151	0.0192364512974815	0.0156089883670146	CRIM1/LRP2/SULF1/NOTCH2/PPARG/FKBP8/TGFBR3/FSTL1/HTRA1/DDX5/EGR1/ITGA3/BMPR1B/USP9Y	14
GO:0071773	cellular response to BMP stimulus	14/568	184/18986	0.0760869565217391	2.54328689528475	3.69412672868582	0.00131686910709151	0.0192364512974815	0.0156089883670146	CRIM1/LRP2/SULF1/NOTCH2/PPARG/FKBP8/TGFBR3/FSTL1/HTRA1/DDX5/EGR1/ITGA3/BMPR1B/USP9Y	14
GO:0030865	cortical cytoskeleton organization	7/568	56/18986	0.125	4.17825704225352	4.18278901467075	0.00134583248244252	0.0195339200247169	0.0158503627157011	EPB41L3/NLGN1/IQGAP2/FHOD3/EPB41L1/RHOQ/LLGL2	7
GO:0048260	positive regulation of receptor-mediated endocytosis	7/568	56/18986	0.125	4.17825704225352	4.18278901467075	0.00134583248244252	0.0195339200247169	0.0158503627157011	SGIP1/INSR/EGF/HIP1/ANXA2/SYNE1/MAGI2	7
GO:0010959	regulation of metal ion transport	24/568	408/18986	0.0588235294117647	1.9662386081193	3.46476052897016	0.00135396120976353	0.0195893177578207	0.0158953139677904	SLC8A1/ATP1B1/EGF/ITGB3/FXYD2/KCNIP4/ANK3/UTRN/KCNIP1/PDE4B/DPP6/CACNA1D/FYN/NOS1AP/CACNB4/CASR/PDE4D/ATP1A1/CHP1/F2R/TRPC6/FHL1/PDGFRB/FGF13	24

ID	Description	GeneRatio	BgRatio	RichFactor	FoldEnrichment	zScore	pvalue	p.adjust	qvalue	geneID	Count
GO:0072109	glomerular mesangium development	4/568	17/18986	0.235294117647059	7.86495443247722	4.97276728408773	0.00138284204597292	0.0199436552852539	0.0161828332330732	ITGB3/WT1/PDGFRB/EGR1	4
GO:0018212	peptidyl-tyrosine modification	15/568	206/18986	0.0728155339805825	2.43393614111856	3.63390744241007	0.0013991304925971	0.0201051622829538	0.0163138844758053	INSR/EGF/ITGB3/NEDD9/EFNA5/NRG1/PTPRJ/FYN/PARP14/PRLR/PDGFRB/IGF1/FGFR1/GPRC5A/PLPP3	15
GO:1990778	protein localization to cell periphery	22/568	362/18986	0.0607734806629834	2.03141778849895	3.47945044297667	0.0014028915790659	0.0201051622829538	0.0163138844758053	WDR72/RAB11FIP3/EPB41L3/SLC4A1/ATP1B1/KCNIP4/ANK3/DPP6/SLC1A1/MYADM/CD81/RAMP1/RAMP3/UMOD/MAL/CDH1/ITGA3/RHOQ/FGF13/MACF1/GPC6/PDZK1	22
GO:0010811	positive regulation of cell-substrate adhesion	11/568	126/18986	0.0873015873015873	2.91814777554214	3.79362785189534	0.00142382415039692	0.020340984639161	0.0165052372548494	NPNT/SPOCK2/ITGB3/CDK6/UTRN/NEDD9/PTPRJ/MYADM/NDNF/ITGA3/FMN1	11
GO:0001666	response to hypoxia	20/568	317/18986	0.0630914826498423	2.10889945350335	3.49638708186729	0.00146181924254557	0.0207765689442737	0.0168586824015771	DPP4/FMN2/MALAT1/PCK1/PPARA/THBS1/GNGT1/FOS/SCN2A/VASN/ITPR2/PPARG/DDAH1/AK4/CASR/NDNF/TGFBR3/EGR1/EPAS1/RORA	20
GO:0001763	morphogenesis of a branching structure	15/568	207/18986	0.072463768115942	2.42217799550929	3.61294317378918	0.00146803403722471	0.0207765689442737	0.0168586824015771	PKHD1/NPNT/SLIT2/PAX8/EGF/FGF1/SULF1/WT1/CASR/RERE/CLIC4/FGFR1/CTSH/MAP3K13/FGF13	15
GO:0032271	regulation of protein polymerization	15/568	207/18986	0.072463768115942	2.42217799550929	3.61294317378918	0.00146803403722471	0.0207765689442737	0.0168586824015771	SLIT2/TPPP3/CDC42EP3/NPHS1/HSP90AA1/ARHGAP28/MYADM/CYFIP2/EPS8/NCK2/TMOD3/DAAM2/SCIN/FMN1/KIRREL1	15
GO:1901379	regulation of potassium ion transmembrane	9/568	90/18986	0.1	3.34260563380282	3.9119539361328	0.0014797265503608	0.0208770115474818	0.016940184306517	ATP1B1/FXYD2/KCNIP4/ANK3/KCNIP1/DPP6/CACNA1D/NOS1AP/FH	9

ID	Description	GeneRatio	BgRatio	RichFactor	FoldEnrichment	zScore	pvalue	p.adjust	qvalue	geneID	Count
	transport						1			L1	
GO:0050770	regulation of axonogenesis	12/568	146/18986	0.0821917808219178	2.74734709627629	3.72197451877301	0.00148570933255473	0.0208965247609788	0.0169560178673836	SPP1/SLIT2/ROBO2/EFNA5/SEMA3G/SEMA5A/PLXNA4/CDH1/ROBO1/MAP3K13/FGF13/MACF1	12
GO:1904062	regulation of monoatomic cation transmembrane transport	20/568	318/18986	0.0628930817610063	2.10226769421561	3.48104772317014	0.00151763435721965	0.0212796693976817	0.0172669120174894	SLC8A1/ATP1B1/ITGB3/FXYD2/KCNIP4/ANK3/UTRN/KCNIP1/PDE4B/DPP6/CACNA1D/FYN/NOS1AP/CACNB4/PDE4D/CHP1/F2R/FHL1/FGF13/PDZK1	20
GO:0033002	muscle cell proliferation	17/568	252/18986	0.0674603174603175	2.25493237200984	3.52179091787607	0.00159853457934889	0.0222912360395202	0.0180877251550447	PPARGC1A/APOE/ITGB3/HPGD/THBS1/FOS/PDE1A/NDRG2/PPARG/NRG1/APOD/TGFBR3/STAT1/PRKG1/PDGFRB/IGF1/FGFR1	17
GO:0051591	response to cAMP	9/568	91/18986	0.0989010989010989	3.30587370376103	3.87205037628045	0.00159959122801752	0.0222912360395202	0.0180877251550447	ASS1/PCK1/WT1/FOS/ITPR2/PDE4D/STAT1/FOSB/SLC26A6	9
GO:1905952	regulation of lipid localization	14/568	188/18986	0.074468085106383	2.48917440815103	3.60351969822529	0.00161478267447383	0.0224341213765584	0.0182036662675272	PLA2R1/SPP1/EGF/APOE/ITGB3/PPARA/PLTP/THBS1/TMEM30A/PPARG/PLIN5/ANXA2/ITGAV/ATP8A1	14
GO:0170038	proteinogenic amino acid biosynthetic process	7/568	58/18986	0.120689655172414	4.0341792132103	4.06406921830734	0.00165760375747329	0.0229321796204714	0.0186078044952653	ASS1/SERINC5/PAH/AGXT2/BHMT2/BHMT/GLS	7
GO:0030866	cortical actin cytoskeleton organization	6/568	43/18986	0.13953488372093	4.66410088437602	4.22411278150223	0.00166072795402489	0.0229321796204714	0.0186078044952653	EPB41L3/IQGAP2/FHOD3/EPB41L1/RHOQ/LLGL2	6
GO:0060047	heart contraction	17/568	253/18986	0.0671936758893281	2.24601959583588	3.50380316604633	0.00166753388590674	0.0229563831626494	0.0186274439184861	SLC8A1/ATP1B1/HSP90AA1/KCNIP1/PDE4B/SCN2A/CACNA1D/THRB/KCNJ3/SLC1A1/NOS1AP/PDE4D/ATP1A1/DSP/	17

ID	Description	GeneRatio	BgRatio	RichFactor	FoldEnrichment	zScore	pvalue	p.adjust	qvalue	geneID	Count
										TNNI3K/EPAS1/FGF13	
GO:0003206	cardiac chamber morphogenesis	11/568	129/18986	0.0852713178294574	2.85028387378535	3.70301228304488	0.00172113109093942	0.0235130653102423	0.0190791512023052	LRP2/SLIT2/ROBO2/NOTCH2/NRG1/DHRS3/DSP/TGFBR3/PARVA/ADAMTS19/ROBO1	11
GO:0007613	memory	11/568	129/18986	0.0852713178294574	2.85028387378535	3.70301228304488	0.00172113109093942	0.0235130653102423	0.0190791512023052	CALB1/INSR/APOE/FOS/SCN2A/SLC1A1/PJA2/RCAN1/ITGA3/FGF13/ACSS2	11
GO:0055017	cardiac muscle tissue growth	9/568	92/18986	0.0978260869565217	3.26994029393754	3.83269823377803	0.00172712118869796	0.0235130653102423	0.0190791512023052	PPARA/NRG1/PDLIM5/SORBS2/TGFBR3/PRKG1/IGF1/FGFR1/MEIS1	9
GO:0032148	activation of protein kinase B activity	4/568	18/18986	0.222222222222222	7.42801251956182	4.79137795419389	0.001736230000969	0.0235130653102423	0.0190791512023052	INSR/FGF1/NRG1/IGF1	4
GO:0033631	cell-cell adhesion mediated by integrin	4/568	18/18986	0.222222222222222	7.42801251956182	4.79137795419389	0.001736230000969	0.0235130653102423	0.0190791512023052	DPP4/NPNT/PODXL/PLPP3	4
GO:0090257	regulation of muscle system process	17/568	254/18986	0.0669291338582677	2.23717699900189	3.48589944532775	0.00173902486115814	0.0235130653102423	0.0190791512023052	SLC8A1/NPNT/ATP1B1/PPARA/HSP90AA1/PDE4B/DOCK4/PPARG/PDE4D/ATP1A1/DSP/TNNI3K/PRKG1/CHRM3/F2R/IGF1/FGF13	17
GO:0090090	negative regulation of canonical Wnt signaling pathway	12/568	149/18986	0.0805369127516778	2.69203138292844	3.64127863921771	0.00176681685716241	0.0238179495017473	0.0193265426636975	APOE/BICC1/PTPRO/MCC/HECW1/ZNRF3/CTNND1/IGFBP2/TLE4/EGR1/PPP2R3A/SHISA6	12
GO:0170035	L-amino acid catabolic process	8/568	75/18986	0.106666666666667	3.56544600938967	3.9092594788707	0.00177258637792485	0.0238250293340609	0.019332287435356	GLDC/PAH/HMGCLL1/AGXT2/DDAH1/FTCD/HGD/GLS	8
GO:0072012	glomerulus vasculature development	5/568	30/18986	0.166666666666667	5.57100938967136	4.40005013585103	0.00180538446909936	0.0241942821330926	0.0196319093643408	ITGB3/WT1/NOTCH2/PDGFRB/EGR1	5
GO:0003279	cardiac septum development	10/568	111/18986	0.0900900900900901	3.01135642684938	3.73220320122547	0.00182872335322251	0.0243510655795485	0.0197591277870127	LRP2/SLIT2/PAX8/ROBO2/NOTCH2/DHRS3/TGFBR3/PARVA/ADAMTS19/ROBO1	10

ID	Description	GeneRatio	BgRatio	RichFactor	FoldEnrichment	zScore	pvalue	p.adjust	qvalue	geneID	Count
GO:0006892	post-Golgi vesicle-mediated transport	10/568	111/18986	0.0900900900900901	3.01135642684938	3.73220320122547	0.00182872335322251	0.0243510655795485	0.0197591277870127	SORCS1/RAB11FIP3/VTI1A/ANK3/CCDC91/MYO1B/RAB3IP/LLGL2/AP3D1/MACF1	10
GO:0170034	L-amino acid biosynthetic process	7/568	59/18986	0.118644067796610	3.96580329434233	4.00668954032117	0.00183316408280994	0.0243510655795485	0.0197591277870127	ASS1/SERINC5/PAH/AGXT2/BHMT2/BHMT/GLS	7
GO:0014074	response to purine-containing compound	12/568	150/18986	0.08	2.67408450704225	3.61482186154804	0.00186969635181833	0.0247639373944917	0.0200941433913389	SLC8A1/ASS1/PCK1/WT1/FOS/ITPR2/RYR3/PDE4D/STAT1/FOSB/NT5E/SLC26A6	12
GO:0030038	contractile actin filament bundle assembly	10/568	112/18986	0.0892857142857143	2.98446931589537	3.69896020008016	0.00195560553500605	0.0257516404218333	0.0208955929323911	ARHGAP6/CD47/TJP1/SYNPO/ARHGAP28/DLC1/ZEB2/CCN2/LIMCH1/ITGB5	10
GO:0043149	stress fiber assembly	10/568	112/18986	0.0892857142857143	2.98446931589537	3.69896020008016	0.00195560553500605	0.0257516404218333	0.0208955929323911	ARHGAP6/CD47/TJP1/SYNPO/ARHGAP28/DLC1/ZEB2/CCN2/LIMCH1/ITGB5	10
GO:1901136	carbohydrate derivative catabolic process	17/568	258/18986	0.0658914728682171	2.20249208428868	3.41510934209193	0.0020513282627171	0.0269340586633636	0.0218550397810627	SLC4A4/INSR/SLC4A1/ALDOB/PPARA/PDE4B/PDE1A/DPYS/OGDH/DHTKD1/PDE7B/PDE4D/PRKAG2/IGF1/PFKFB2/GDA/NT5E	17
GO:0043406	positive regulation of MAP kinase activity	8/568	77/18986	0.103896103896104	3.4728370221328	3.81825395338026	0.00209900351752775	0.0274190083616202	0.0222485413724744	INSR/FGF1/THBS1/TAOK3/PDGFRB/ROBO1/FGFR1/MAP3K13	8
GO:0003338	metanephros morphogenesis	5/568	31/18986	0.161290322580645	5.39129940935938	4.29704830859586	0.00210033346023417	0.0274190083616202	0.0222485413724744	CALB1/PAX8/WT1/STAT1/FMN1	5
GO:0001502	cartilage condensation	4/568	19/18986	0.210526315789474	7.03706449221646	4.62340063137681	0.00214768625932504	0.0277885135414968	0.0225483680902538	SOX6/CCN2/SOX5/BMPR1B	4
GO:0055003	cardiac myofibril assembly	4/568	19/18986	0.210526315789474	7.03706449221646	4.62340063137681	0.0021476862593250	0.0277885135414968	0.0225483680902538	NEBL/MYLK3/FHOD3/PDGFRB	4

ID	Description	GeneRatio	BgRatio	RichFactor	FoldEnrichment	zScore	pvalue	p.adjust	qvalue	geneID	Count
							4				
GO:0055064	chloride ion homeostasis	4/568	19/18986	0.210526315789474	7.03706449221646	4.62340063137681	0.00214768625932504	0.0277885135414968	0.0225483680902538	SLC12A3/CA12/SLC12A1/UMOD	4
GO:0030308	negative regulation of cell growth	13/568	173/18986	0.0751445086705202	2.51178458031426	3.50785933186669	0.00215761135841036	0.0277885135414968	0.0225483680902538	SPP1/SLIT2/PPARA/WT1/BST2/SEMA3G/PTPRJ/SEMA5A/RERG/CDH1/FHL1/TSPYL2/FGF13	13
GO:0106027	neuron projection organization	9/568	95/18986	0.0947368421052632	3.16667902149741	3.71780957234576	0.00215922194148104	0.0277885135414968	0.0225483680902538	PTPRD/INSR/APOE/NLGN1/TANC1/NEDD9/FYN/PDLIM5/ITGA3	9
GO:0010595	positive regulation of endothelial cell migration	11/568	133/18986	0.0827067669172932	2.76456105051361	3.58616699903073	0.00219447734080052	0.02816245920694	0.0228517979407774	EGF/ITGB3/AKT3/FGF1/THBS1/PLG/CCBE1/SEMA5A/TGFBR3/FGFR1/PLPP3	11
GO:0060560	developmental growth involved in morphogenesis	16/568	238/18986	0.0672268907563025	2.24712983785063	3.40001560498819	0.0022428100137674	0.0287016504015361	0.0232893125818041	SPP1/SLIT2/APOE/FGF1/EFNA5/SEMA3G/SEMA5A/CYFIP2/PRKG1/PLXNA4/CDH1/PPP2R3A/FMN1/MAP3K13/FGF13/MACF1	16
GO:0055074	calcium ion homeostasis	20/568	329/18986	0.060790273556231	2.03197910869472	3.31593633048691	0.00226128690767751	0.028856815790952	0.0234152180682632	SLC8A1/PKHD1/CALB1/PLCE1/ATP1B1/APOE/ITGB3/ITPR2/KL/RYR3/CACNB4/GRIK2/CASR/PDE4D/F2R/UMOD/TRPC6/FGFR1/NT5E/TMTC2	20
GO:0048660	regulation of smooth muscle cell proliferation	13/568	174/18986	0.0747126436781609	2.49734903674923	3.48448356878441	0.00226995150977129	0.0288862456831679	0.0234390982963852	PPARGC1A/APOE/ITGB3/HPGD/THBS1/PDE1A/NDRG2/PPARG/APOD/STAT1/PRKG1/PDGFRB/IGF1	13
GO:0050772	positive regulation of axonogenesis	8/568	78/18986	0.102564102564103	3.42831347056699	3.77387479718239	0.00227901222142452	0.0289205377707587	0.0234669238442478	SLIT2/ROBO2/EFNA5/SEMA5A/PLXNA4/ROBO1/MAP3K13/MACF1	8
GO:0016358	dendrite development	16/568	239/18986	0.0669456066945607	2.23772762095586	3.38155439477191	0.0023392508252419	0.0296022743706804	0.0240201037677683	SRGAP2/PTPRD/KLF7/APOE/SRGAP2C/CUX1/SDK1/NLGN1/HECW1/F	16

ID	Description	GeneRatio	BgRatio	RichFactor	FoldEnrichment	zScore	pvalue	p.adjust	qvalue	geneID	Count
							7			YN/PDLIM5/SYNE1/RERE/FARP1/NCK2/PRKG1	
GO:0030100	regulation of endocytosis	19/568	307/18986	0.0618892508143322	2.06871358443823	3.31521684713308	0.00235925646421566	0.0297725058803659	0.0241582343207112	SGIP1/CBLB/INSR/CD47/EGF/APOE/ITGB3/NLGN1/HIP1/SIRPA/NRG1/PTPRJ/ANXA2/STON2/ITGAV/SYNE1/EFNB2/MAGI2/BCR	19
GO:0050680	negative regulation of epithelial cell proliferation	13/568	175/18986	0.0742857142857143	2.48307847082495	3.46126975441885	0.00238705334027841	0.0300398430052211	0.0243751590542499	COL4A3/APOE/HPN/SULF1/MCC/CDK6/THBS1/PPARG/EFNB2/TGFBR3/STAT1/ROBO1/MTSS1	13
GO:0010644	cell communication by electrical coupling	5/568	32/18986	0.15625	5.2228213028169	4.19841619932438	0.0024289201855065	0.0301887402196314	0.0244959783702282	SLC8A1/ATP1B1/ANK3/PDE4D/ATP1A1	5
GO:0061437	renal system vasculature development	5/568	32/18986	0.15625	5.2228213028169	4.19841619932438	0.0024289201855065	0.0301887402196314	0.0244959783702282	ITGB3/WT1/NOTCH2/PDGFRB/EGR1	5
GO:0061440	kidney vasculature development	5/568	32/18986	0.15625	5.2228213028169	4.19841619932438	0.0024289201855065	0.0301887402196314	0.0244959783702282	ITGB3/WT1/NOTCH2/PDGFRB/EGR1	5
GO:0090313	regulation of protein targeting to membrane	5/568	32/18986	0.15625	5.2228213028169	4.19841619932438	0.0024289201855065	0.0301887402196314	0.0244959783702282	ANK3/FYN/SLC1A1/CHP1/PDZK1	5
GO:0009410	response to xenobiotic stimulus	25/568	451/18986	0.0554323725055432	1.85288560632085	3.21914838075564	0.00243211070226394	0.0301887402196314	0.0244959783702282	ACSM2B/ASS1/ITGB3/THBS1/HSP90AA1/PDE4B/FOS/CYP3A5/SLC1A1/ATP1A1/IGFBP2/STAT1/LOX/GLYAT/FOSB/VAV3/UMOD/CDH1/NQO1/BCAR3/ITGA3/POR/MYO6/RORA/UGT2B7	25
GO:0046683	response to organophosphorus	11/568	135/18986	0.0814814814814815	2.723604591	3.5293567866159	0.00246795365592173	0.0305036686154729	0.0247515199766873	ASS1/PCK1/WT1/FOS/ITPR2/RYR3/PDE4D/STAT1/FOSB/NT5E/SLC26A6	11
GO:0055117	regulation of cardiac muscle contraction	8/568	79/18986	0.10126582278481	3.38491709752184	3.73021406149457	0.00247091130321242	0.0305036686154729	0.0247515199766873	SLC8A1/ATP1B1/HSP90AA1/PDE4B/PDE4D/DSP/TNNI3K/FGF13	8

ID	Description	GeneRatio	BgRatio	RichFactor	FoldEnrichment	zScore	pvalue	p.adjust	qvalue	geneID	Count
GO:1905039	carboxylic acid transmembrane transport	12/568	156/18986	0.0769230769230769	2.57123510292524	3.46048161751148	0.00259522016796696	0.031951450469035	0.0259262901960357	SLC5A12/LRP2/SLC13A3/SLC16A9/SLC16A12/THBS1/SLC36A2/SLC1A1/SLC6A13/MPC1/SLC3A1/SLC16A7	12
GO:0032332	positive regulation of chondrocyte differentiation	4/568	20/18986	0.2	6.68521126760563	4.46716472383074	0.00262176149831907	0.0321909796942258	0.0261206508310481	ZBTB16/SOX6/SOX5/BMPR1B	4
GO:0001508	action potential	13/568	177/18986	0.0734463276836158	2.45502108697382	3.41531921587532	0.00263614464990779	0.0322803373167954	0.0261931580762579	SLC8A1/ATP1B1/ANK3/SCN2A/CACNA1D/KCNJ3/NOS1AP/CACNB4/GRIK2/ATP1A1/DSP/KCNMB2/FGF13	13
GO:0071346	cellular response to type II interferon	9/568	98/18986	0.0918367346938776	3.0697398677781	3.60739791814083	0.00267330408031203	0.0326473667657461	0.0264909759175232	CD47/ASS1/VIM/SIRPA/PPARG/PARP14/STAT1/DAPK1/SLC26A6	9
GO:0009750	response to fructose	3/568	10/18986	0.3	10.0278169014085	5.01462706986112	0.00273134950753878	0.0326540021388123	0.0264963600426615	PCK1/PPARA/SLC26A6	3
GO:0072014	proximal tubule development	3/568	10/18986	0.3	10.0278169014085	5.01462706986112	0.00273134950753878	0.0326540021388123	0.0264963600426615	SLC22A6/NOTCH2/ACAT1	3
GO:0072205	metanephric collecting duct development	3/568	10/18986	0.3	10.0278169014085	5.01462706986112	0.00273134950753878	0.0326540021388123	0.0264963600426615	CALB1/PAX8/AQP2	3
GO:0097113	AMPA glutamate receptor clustering	3/568	10/18986	0.3	10.0278169014085	5.01462706986112	0.00273134950753878	0.0326540021388123	0.0264963600426615	APOE/NLGN1/SHISA6	3
GO:0097688	glutamate receptor clustering	3/568	10/18986	0.3	10.0278169014085	5.01462706986112	0.00273134950753878	0.0326540021388123	0.0264963600426615	APOE/NLGN1/SHISA6	3
GO:1900748	positive regulation of vascular endothelial growth factor signaling	3/568	10/18986	0.3	10.0278169014085	5.01462706986112	0.0027313495075387	0.0326540021388123	0.0264963600426615	CCBE1/ADGRG1/ROBO1	3

ID	Description	GeneRatio	BgRatio	RichFactor	FoldEnrichment	zScore	pvalue	p.adjust	qvalue	geneID	Count
	pathway						8				
GO:1903044	protein localization to membrane raft	3/568	10/18986	0.3	10.0278169014085	5.01462706986112	0.00273134950753878	0.0326540021388123	0.0264963600426615	ATP1B1/MYADM/UMOD	3
GO:1904669	ATP export	3/568	10/18986	0.3	10.0278169014085	5.01462706986112	0.00273134950753878	0.0326540021388123	0.0264963600426615	CD47/CR1/CALHM5	3
GO:0048659	smooth muscle cell proliferation	13/568	178/18986	0.0730337078651685	2.44122883367621	3.39257821929003	0.00276844207611217	0.0330105836004661	0.0267857000982037	PPARGC1A/APOE/ITGB3/HPGD/THBS1/PDE1A/NDRG2/PPARG/APOD/STAT1/PRKG1/PDGFRB/IGF1	13
GO:0030511	positive regulation of transforming growth factor beta receptor signaling pathway	5/568	33/18986	0.151515151515152	5.06455399061033	4.10382771083298	0.00279329338766409	0.0330466975524947	0.0268150039572359	NPNT/THBS1/ZEB2/TGFBR3/HSP90AB1	5
GO:0099560	synaptic membrane adhesion	5/568	33/18986	0.151515151515152	5.06455399061033	4.10382771083298	0.00279329338766409	0.0330466975524947	0.0268150039572359	PTPRD/NTNG1/EFNA5/IL1RAP/ITGA3	5
GO:1903846	positive regulation of cellular response to transforming growth factor beta stimulus	5/568	33/18986	0.151515151515152	5.06455399061033	4.10382771083298	0.00279329338766409	0.0330466975524947	0.0268150039572359	NPNT/THBS1/ZEB2/TGFBR3/HSP90AB1	5
GO:0006637	acyl-CoA metabolic process	9/568	99/18986	0.0909090909090909	3.0387323943662	3.57153215997063	0.00286466652146288	0.0337154922461292	0.0273576824602209	ACSM2B/ACSM2A/OGDH/ELOVL7/GLYAT/SLC27A2/ACAT1/ACSF2/ACSS2	9
GO:0035383	thioester metabolic process	9/568	99/18986	0.0909090909090909	3.0387323943662	3.57153215997063	0.00286466652146288	0.0337154922461292	0.0273576824602209	ACSM2B/ACSM2A/OGDH/ELOVL7/GLYAT/SLC27A2/ACAT1/ACSF2/ACSS2	9
GO:0050731	positive regulation of peptidyl-tyrosine phosphorylation	10/568	118/18986	0.0847457627118644	2.83271663881595	3.50696657697699	0.00287397526703545	0.0337376476437779	0.0273756599623505	EGF/ITGB3/NEDD9/EFNA5/NRG1/PTPRJ/FYN/PARP14/IGF1/PLPP3	10
GO:2000027	regulation of animal organ morphogenesis	8/568	81/18986	0.0987654320987654	3.30133889758303	3.64496179773304	0.0028925103743249	0.0337933651125343	0.0274208706567872	PKHD1/PAX8/FGF1/SULF1/ROBO2/WT1/ROBO1/FGFR1	8

ID	Description	GeneRatio	BgRatio	RichFactor	FoldEnrichment	zScore	pvalue	p.adjust	qvalue	geneID	Count
GO:0001558	regulation of cell growth	23/568	408/18986	0.0563725490196078	1.88431199944767	3.17098621938179	0.00289359872964469	0.0337933651125343	0.0274208706567872	SPP1/SLIT2/APOE/PPARA/HPN/SPOCK1/WT1/EFNA5/BST2/SEMA3G/NRG1/PTPRJ/SEMA5A/RERG/PLXNA4/CDH1/SGK2/FHL1/IGF1/TSPYL2/MAP3K13/FGF13/MACF1	23
GO:0050673	epithelial cell proliferation	26/568	482/18986	0.0539419087136929	1.80306527964467	3.13617142313489	0.00290953954088057	0.0338924054723601	0.0275012347426822	PKHD1/DACH1/COL4A3/EGF/APOE/ITGB3/AKT3/HPN/FGF1/SULF1/MCC/CDK6/THBS1/NOTCH2/PPARG/SEMA5A/LAMB1/ZEB2/EFNB2/TGFBR3/STAT1/ROBO1/IGF1/MTSS1/FGFR1/F3	26
GO:0001657	ureteric bud development	9/568	100/18986	0.09	3.00834507042254	3.53611659354971	0.0030666903516338	0.0355407506823274	0.028838747613914	NPNT/CALB1/SLIT2/PAX8/FGF1/ROBO2/WT1/FGFR1/FMN1	9
GO:0043648	dicarboxylic acid metabolic process	9/568	100/18986	0.09	3.00834507042254	3.53611659354971	0.0030666903516338	0.0355407506823274	0.028838747613914	DHFR/ASS1/PCK1/ACMSD/ALDH1L1/OGDH/FTCD/GLS/ASPA	9
GO:1901654	response to ketone	15/568	224/18986	0.0669642857142857	2.23835198692153	3.27406112300599	0.0031496937171396	0.0364098182111074	0.029543933031801	SPP1/SLC12A3/SLIT2/ASS1/PCK1/THBS1/EFNA5/SCNN1A/FOS/F5/SH3RF1/TGFBR3/FOSB/NQO1/TRERF1	15
GO:0055081	monoatomic anion homeostasis	4/568	21/18986	0.19047619047619	6.36686787391013	4.32127954341482	0.00316291145384124	0.0364698140477176	0.0295926153121881	SLC12A3/CA12/SLC12A1/UMOD	4
GO:0140058	neuron projection arborization	5/568	34/18986	0.147058823529412	4.91559652029826	4.01299023391527	0.0031956083067714	0.0365985471423149	0.0296970729039209	LRP2/NTNG1/NLGN1/MAP3K13/MACF1	5
GO:1901889	negative regulation of cell junction assembly	5/568	34/18986	0.147058823529412	4.91559652029826	4.01299023391527	0.0031956083067714	0.0365985471423149	0.0296970729039209	ARHGAP6/ROBO2/THBS1/DLC1/APOD	5
GO:0035265	organ growth	13/568	181/18986	0.0718232044198895	2.40076647731694	3.32526753770321	0.00319824415925578	0.0365985471423149	0.0296970729039209	PPARA/BNC2/WWC1/WT1/NRG1/PDLIM5/SORBS2/TGFBR3/PRLR/PRKG1/IGF1/FGFR1/MEIS1	13

ID	Description	GeneRatio	BgRatio	RichFactor	FoldEnrichment	zScore	pvalue	p.adjust	qvalue	geneID	Count
GO:1901607	alpha-amino acid biosynthetic process	7/568	65/18986	0.107692307692308	3.59972914409534	3.68698161974491	0.00320875305814047	0.0366265455857592	0.0297197916149376	ASS1/SERINC5/PAH/AGXT2/BHMT2/BHMT/GLS	7
GO:0032369	negative regulation of lipid transport	6/568	49/18986	0.122448979591837	4.0929864903708	3.80696345473451	0.00327596420387423	0.0369918687537425	0.0300162249326567	PLA2R1/EGF/APOE/ITGB3/THBS1/ITGAV	6
GO:0006942	regulation of striated muscle contraction	9/568	101/18986	0.0891089108910891	2.97855947566588	3.50114047626923	0.00327978187189624	0.0369918687537425	0.0300162249326567	SLC8A1/ATP1B1/HSP90AA1/PDE4B/PDE4D/ATP1A1/DSP/TNNI3K/FGF13	9
GO:0072163	mesonephric epithelium development	9/568	101/18986	0.0891089108910891	2.97855947566588	3.50114047626923	0.00327978187189624	0.0369918687537425	0.0300162249326567	NPNT/CALB1/SLIT2/PAX8/FGF1/ROBO2/WT1/FGFR1/FMN1	9
GO:0072164	mesonephric tubule development	9/568	101/18986	0.0891089108910891	2.97855947566588	3.50114047626923	0.00327978187189624	0.0369918687537425	0.0300162249326567	NPNT/CALB1/SLIT2/PAX8/FGF1/ROBO2/WT1/FGFR1/FMN1	9
GO:0051258	protein polymerization	18/568	293/18986	0.0614334470989761	2.05347786376965	3.1913758616774	0.00328147107808898	0.0369918687537425	0.0300162249326567	WDR72/ARHGAP6/SLIT2/TPPP3/CDC42EP3/NPHS1/HSP90AA1/ARHGAP28/MYADM/CYFIP2/EPS8/NCK2/TMOD3/DAAM2/SCIN/FMN1/FGF13/KIRREL1	18
GO:0032102	negative regulation of response to external stimulus	24/568	437/18986	0.0549199084668192	1.83575595449125	3.10397476361052	0.00330832734113264	0.0372023047296178	0.0301869785009028	DPP4/SPP1/SLIT2/CR1/APOE/UACA/PPARA/PTPRO/ROBO2/THBS1/PLG/SIRPA/PPARG/FYN/SEMA5A/HLA-E/ANXA2/PARP14/PRKG1/ROBO1/IGF1/NT5E/RORA/BCR	24
GO:0009308	amine metabolic process	10/568	121/18986	0.0826446280991736	2.76248399487836	3.41544814868135	0.0034473700045213	0.038574881602316	0.0313007253197656	PAH/SULT1C2/ACMSD/NAPEPLD/KL/SLC1A1/AFMID/BHMT/EPAS1/MAOA	10
GO:0034341	response to type II interferon	10/568	121/18986	0.0826446280991736	2.76248399487836	3.4154481486	0.0034473700	0.0385748816	0.0313007253	CD47/ASS1/VIM/BST2/SIRPA/PPARG/PARP14/S	10

ID	Description	GeneRatio	BgRatio	RichFactor	FoldEnrichment	zScore	pvalue	p.adjust	qvalue	geneID	Count
						8135	045213	02316	197656	TAT1/DAPK1/SLC26A6	
GO:0043470	regulation of carbohydrate catabolic process	7/568	66/18986	0.106060606060606	3.54518779342723	3.63738664348617	0.00349897129519461	0.0390560849977128	0.031691187057472	SLC4A4/INSR/SLC4A1/ALDOB/PPARA/PRKAG2/IGF1	7
GO:0032411	positive regulation of transporter activity	8/568	84/18986	0.0952380952380952	3.18343393695506	3.52195703902996	0.00362763542696519	0.0403016046488949	0.032701836134349	ATP1B1/FXYD2/ANK3/CHP1/TRPC6/SGK2/FGF13/PDZK1	8
GO:0070372	regulation of ERK1 and ERK2 cascade	18/568	296/18986	0.0608108108108108	2.03266558812333	3.1445546310971	0.00365202568679782	0.0403016046488949	0.032701836134349	PKHD1/NPNT/APOE/ITGB3/FGF1/CALCR/NOTCH2/GCNT2/NDRG2/SIRPA/NRG1/CASR/CCN2/ARHGAP8/RAMP3/F2R/PDGFRB/IGF1	18
GO:0001865	NK T cell differentiation	3/568	11/18986	0.272727272727273	9.11619718309859	4.72842248924875	0.00367265338424884	0.0403016046488949	0.032701836134349	ZBTB16/TOX/AP3D1	3
GO:0010248	establishment or maintenance of transmembrane electrochemical gradient	3/568	11/18986	0.272727272727273	9.11619718309859	4.72842248924875	0.00367265338424884	0.0403016046488949	0.032701836134349	ATP1B1/FXYD2/ATP1A1	3
GO:0015747	urate transport	3/568	11/18986	0.272727272727273	9.11619718309859	4.72842248924875	0.00367265338424884	0.0403016046488949	0.032701836134349	SLC17A1/SLC2A9/UMOD	3
GO:0021889	olfactory bulb interneuron differentiation	3/568	11/18986	0.272727272727273	9.11619718309859	4.72842248924875	0.00367265338424884	0.0403016046488949	0.032701836134349	SLIT2/ROBO2/ROBO1	3
GO:0072124	regulation of glomerular mesangial cell proliferation	3/568	11/18986	0.272727272727273	9.11619718309859	4.72842248924875	0.00367265338424884	0.0403016046488949	0.032701836134349	ITGB3/WT1/EGR1	3
GO:0046034	ATP metabolic process	15/568	228/18986	0.0657894736842105	2.19908265381764	3.19875893828262	0.00371790041204124	0.0406998110166346	0.0330249517893936	SLC4A4/INSR/SLC4A1/ATP1B1/ALDOB/PPARA/OGDH/DHTKD1/AK4/ATP6V0C/PRKAG2/IGF1/PFKFB2/ATP6V1A/NT	15

ID	Description	GeneRatio	BgRatio	RichFactor	FoldEnrichment	zScore	pvalue	p.adjust	qvalue	geneID	Count
										5E	
GO:0001759	organ induction	4/568	22/18986	0.181818181818182	6.07746478873239	4.18457659552107	0.0037754776048294	0.0411318819154435	0.0333755460611196	FGF1/ROBO2/ROBO1/FGFR1	4
GO:0060004	reflex	4/568	22/18986	0.181818181818182	6.07746478873239	4.18457659552107	0.0037754776048294	0.0411318819154435	0.0333755460611196	NPNT/HPN/SLC1A1/USP46	4
GO:0035904	aorta development	7/568	67/18986	0.104477611940299	3.49227454277906	3.58874357888327	0.00380874551694329	0.0412962550918219	0.0335089230004658	LRP2/ROBO2/EFNB2/LOX/PDGFRB/ROBO1/LOXL1	7
GO:0070613	regulation of protein processing	7/568	67/18986	0.104477611940299	3.49227454277906	3.58874357888327	0.00380874551694329	0.0412962550918219	0.0335089230004658	HPN/MYH9/THBS1/CCBE1/ANXA2/TMEM98/GAS1	7
GO:0043405	regulation of MAP kinase activity	10/568	123/18986	0.0813008130081301	2.71756555593725	3.35597509938779	0.00387760873751419	0.0419428011774452	0.0340335483678564	INSR/APOE/FGF1/THBS1/PTPRJ/TAOK3/PDGFRB/ROBO1/FGFR1/MAP3K13	10
GO:0009205	purine ribonucleoside triphosphate metabolic process	16/568	252/18986	0.0634920634920635	2.12228929130338	3.14954679749932	0.00394026104502728	0.0425192539847006	0.0345012981114778	SLC4A4/INSR/SLC4A1/ATP1B1/ALDOB/PPARA/OGDH/DHTKD1/AK4/ATP6V0C/PRKAG2/IGF1/PFKFB2/ATP6V1A/NT5E/RHOQ	16
GO:0051495	positive regulation of cytoskeleton organization	13/568	186/18986	0.0698924731182796	2.33622974405573	3.21600649030499	0.00403421552159569	0.0434299552478891	0.0352402662924123	CD47/CDC42EP3/NPHS1/SYNPO/SEMA5A/MYLK3/CCN2/LIMCH1/NCK2/SCIN/MTSS1/FMN1/KIRREL1	13
GO:0060828	regulation of canonical Wnt signaling pathway	17/568	276/18986	0.0615942028985507	2.05885129618289	3.11179410796005	0.00409796167597861	0.0440119146429571	0.0357124842336123	GPC5/EGF/APOE/BICC1/PTPRO/MCC/HECW1/ZNRF3/SEMA5A/ZEB2/CTNND1/IGFBP2/TLE4/DAAM2/EGR1/PPP2R3A/SHISA6	17
GO:0007035	vacuolar acidification	5/568	36/18986	0.138888888888889	4.64250782472613	3.84154009955201	0.0041226784890628	0.0440690079430877	0.0357588113156605	ATP6V0A4/ATP6V0D2/ATP6V0C/CLIC4/ATP6V1A	5
GO:0010765	positive regulation of sodium ion transport	5/568	36/18986	0.138888888888889	4.64250782472613	3.8415400995	0.0041226784	0.0440690079	0.0357588113	ATP1B1/FXYD2/ANK3/CHP1/FGF13	5

ID	Description	GeneRatio	BgRatio	RichFactor	FoldEnrichment	zScore	pvalue	p.adjust	qvalue	geneID	Count
						5201	890628	430877	156605		
GO:1900542	regulation of purine nucleotide metabolic process	8/568	86/18986	0.0930232558139535	3.10940058958402	3.44298888657478	0.00419279143937717	0.0447132664532641	0.0362815804810943	SLC4A4/INSR/SLC4A1/ALDOB/PPARA/ACMSD/PRKAG2/IGF1	8
GO:0002027	regulation of heart rate	9/568	105/18986	0.0857142857142857	2.86509054325956	3.36542803138859	0.00425116676959146	0.0452296267781124	0.0367005695232572	SLC8A1/CACNA1D/KCNJ3/SLC1A1/NOS1AP/PDE4D/DSP/TNNI3K/EPAS1	9
GO:0048638	regulation of developmental growth	19/568	325/18986	0.0584615384615385	1.95413867822319	3.04680008942361	0.00439502777445219	0.0466509606993839	0.0378538791592021	SPP1/INSR/APOE/PPARA/WWC1/WT1/EFNA5/SEMA3G/NRG1/SEMA5A/TGFBR3/AGRN/PLXNA4/CDH1/IGF1/FGFR1/MAP3K13/FGF13/MACF1	19
GO:2001222	regulation of neuron migration	6/568	52/18986	0.115384615384615	3.85685265438787	3.62265153836591	0.00442384929470177	0.0468474297105597	0.0380132995461798	UNC5D/SRGAP2/NTNG1/UNC5C/SRGAP2C/ADGRG1	6
GO:0010888	negative regulation of lipid storage	4/568	23/18986	0.173913043478261	5.81322718922229	4.05606579336043	0.00446367013005727	0.0469408643538199	0.0380891149986856	ITGB3/PPARA/PPARG/ITGAV	4
GO:0046058	cAMP metabolic process	4/568	23/18986	0.173913043478261	5.81322718922229	4.05606579336043	0.00446367013005727	0.0469408643538199	0.0380891149986856	PDE4B/CACNB4/PDE7B/PDE4D	4
GO:0098743	cell aggregation	4/568	23/18986	0.173913043478261	5.81322718922229	4.05606579336043	0.00446367013005727	0.0469408643538199	0.0380891149986856	SOX6/CCN2/SOX5/BMPR1B	4
GO:0030193	regulation of blood coagulation	7/568	69/18986	0.101449275362319	3.391049194	3.49417907944834	0.00449026731370003	0.0471009595179286	0.0382190206406121	APOE/THBS1/PLG/ANXA2/PRKG1/F2R/F3	7
GO:0006140	regulation of nucleotide metabolic process	8/568	87/18986	0.0919540229885057	3.07366035292213	3.40436465092707	0.00449962941465573	0.0471009595179286	0.0382190206406121	SLC4A4/INSR/SLC4A1/ALDOB/PPARA/ACMSD/PRKAG2/IGF1	8
GO:0006835	dicarboxylic acid transport	9/568	106/18986	0.0849056603773585	2.83806138719107	3.3325001965	0.0045259034	0.0471586683	0.0382658471	LRP2/SLC13A3/SLC22A6/SLC1A1/SLC3A1/SLC	9

ID	Description	GeneRatio	BgRatio	RichFactor	FoldEnrichment	zScore	pvalue	p.adjust	qvalue	geneID	Count
						2153	5296089	183516	566128	26A4/PRKG1/SLC19A2/SLC26A6	
GO:0110020	regulation of actomyosin structure organization	9/568	106/18986	0.0849056603773585	2.83806138719107	3.33250019652153	0.00452590345296089	0.0471586683183516	0.0382658471566128	ARHGAP6/CD47/TJP1/SYNPO/ARHGAP28/DLC1/MYLK3/CCN2/LIMCH1	9
GO:0051494	negative regulation of cytoskeleton organization	12/568	168/18986	0.0714285714285714	2.3875754527163	3.17236208922196	0.00473161039090553	0.0491319153788672	0.0398669943711651	IQCJ-SCHIP1/ARHGAP6/SLIT2/TJP1/ARHGAP28/DLC1/MYADM/EPS8/TMOD3/SCIN/FGF13/ATXN7	12
GO:0046415	urate metabolic process	3/568	12/18986	0.25	8.35651408450704	4.47652980060164	0.00478887910857942	0.0491319153788672	0.0398669943711651	SLC17A1/SLC16A9/SLC2A9	3
GO:0048755	branching morphogenesis of a nerve	3/568	12/18986	0.25	8.35651408450704	4.47652980060164	0.00478887910857942	0.0491319153788672	0.0398669943711651	RERE/MAP3K13/FGF13	3
GO:0072017	distal tubule development	3/568	12/18986	0.25	8.35651408450704	4.47652980060164	0.00478887910857942	0.0491319153788672	0.0398669943711651	CALB1/PAX8/UMOD	3
GO:0097090	presynaptic membrane organization	3/568	12/18986	0.25	8.35651408450704	4.47652980060164	0.00478887910857942	0.0491319153788672	0.0398669943711651	PTPRD/APOE/NLGN1	3
GO:1902043	positive regulation of extrinsic apoptotic signaling pathway via death domain receptors	3/568	12/18986	0.25	8.35651408450704	4.47652980060164	0.00478887910857942	0.0491319153788672	0.0398669943711651	THBS1/MAL/BMPR1B	3
GO:0051347	positive regulation of transferase activity	18/568	304/18986	0.0592105263157895	1.97917438843588	3.0223404777661	0.00481277837907958	0.0491319153788672	0.0398669943711651	ARRDC4/INSR/APOE/ITGB3/SERINC5/FGF1/THBS1/NEDD9/HSP90AA1/NRG1/PRKAG2/PRLR/TAOK3/PDGFRB/ROBO1/IGF1/FGFR1/MAP3K13	18
GO:0032231	regulation of actin filament bundle assembly	9/568	107/18986	0.0841121495327103	2.81153744899302	3.29995445791292	0.00481426916889892	0.0491319153788672	0.0398669943711651	ARHGAP6/CD47/TJP1/SYNPO/ARHGAP28/DLC1/CCN2/LIMCH1/MTSS1	9

ID	Description	GeneRatio	BgRatio	RichFactor	FoldEnrichment	zScore	pvalue	p.adjust	qvalue	geneID	Count
GO:0043266	regulation of potassium ion transport	9/568	107/18986	0.0841121495327103	2.81153744899302	3.29995445791292	0.00481426916889892	0.0491319153788672	0.0398669943711651	ATP1B1/FXYD2/KCNIP4/ANK3/KCNIP1/DPP6/CACNA1D/NOS1AP/FHL1	9
GO:0032370	positive regulation of lipid transport	8/568	88/18986	0.0909090909090909	3.0387323943662	3.36629267216428	0.00482342818819608	0.0491319153788672	0.0398669943711651	PLA2R1/SPP1/APOE/PLTP/TMEM30A/PPARG/ANXA2/ATP8A1	8
GO:0032330	regulation of chondrocyte differentiation	6/568	53/18986	0.113207547169811	3.78408184958809	3.56425239940597	0.00486526104764446	0.0494471609383642	0.0401227933329822	ZBTB16/SOX6/CCN2/SCIN/SOX5/BMPR1B	6
GO:0010594	regulation of endothelial cell migration	15/568	235/18986	0.0638297872340425	2.13357806412946	3.07065752610419	0.00491288459856057	0.0498197203822782	0.0404250983650355	SLIT2/EGF/APOE/ITGB3/AKT3/FGF1/DCN/THBS1/PLG/CCBE1/PPARG/SEMA5A/TGFBR3/FGFR1/PLPP3	15
GO:0009199	ribonucleoside triphosphate metabolic process	16/568	258/18986	0.062015503875969	2.07293372638934	3.04716017702958	0.00493610886343021	0.0498327612590299	0.0404356800929418	SLC4A4/INSR/SLC4A1/ATP1B1/ALDOB/PPARA/OGDH/DHTKD1/AK4/ATP6V0C/PRKAG2/IGF1/PFKFB2/ATP6V1A/NT5E/RHOQ	16
GO:0099003	vesicle-mediated transport in synapse	16/568	258/18986	0.062015503875969	2.07293372638934	3.04716017702958	0.00493610886343021	0.0498327612590299	0.0404356800929418	SGIP1/CBLB/ITGB3/NLGN1/ATP6V0A4/HIP1/NRG1/CADPS2/CACNB4/STON2/SYNE1/EFNB2/ATP6V0C/ATP8A1/ATP6V1A/AP3D1	16

Supplementary Table S7. Gene Set Enrichment Analysis (GSEA) of KEGG pathways in DKD podocytes.

ID	Description	setSize	enrichmentScore	NES	pvalue	p.adjust	qvalue	rank	leading_edge	core_enrichment
hsa01100	Metabolic pathways	500	-0.520106519	-1.96752445	1e-10	2.85e-08	2.29473684210526e-08	1076	tags=36%, list=19%, signal=32%	SMPD4/DGKH/PLCB1/PDE1A/CMPK1/GLUL/GPAT3/AASS/PRDX6/GANC/LPIN1/GOT1/SDHB/RPIA/MAN1C1/PRODH2/ATP6V1B2/GLUD1/ATP5F1C/IDH2/NUDT9/ATP6V0E1/DGLUCY/NADSYN1/NME3/SELENOI/MGST2/AHCYL2/ADH5/PIGU/FKTN/FHIT/AMD1/ALDH2/MTMR8/AGPAT3/ACSF3/RBKS/VKORC1L1/PLPP3/NDUFV2/DAD1/MTHFS/NNT/ATP6V1D/MAT2A/PPCDC/MTMR14/ATP6V0A1/DUT/DMGDH/ME3/ELOVL6/ACER3/PKM/GALNT18/NDUFB2/COX6C/HK1/ST3GAL4/SUCLG1/HNMT/GLCE/BBOX1/ACSS3/MAOB/IP6K3/PGK1/SLC33A1/GYS1/DHRS3/FTCD/XYLB/MCEE/UQCRC2/ATP6V1E1/GPI/MGLL/HADHB/SAT1/ACO1/ARSB/ATP6V1A/ACO2/SGPP2/AOX1/HPRT1/PDE8B/UPP2/NDUFV3/ATP6V0A4/PIK3C2G/AHCYL1/ATP5F1A/MIF/HAGH/L2HGDH/ADCY9/NDUFA13/PCCB/ATP6V0D2/EHHADH/BCAT2/CNDP2/MTMR4/DLST/DHFR/CA12/PAH/PLA2G12A/ABAT/ACADSB/PDE7A/MDH1/CHKA/B4GALT5/ITPK1/GART/PISD/HMGCS1/MECOM/PTGES3/PDE1C/HADH/GSR/ATP5F1B/MAOA/MSMO1/NDUFB1/ACAT1/ACSM2A/ATP6V0B/P4HA1/DBT/OGDH/ACSS2/ATP5F1E/UQCRH/GDA/ASPA/KL/GALNT14/CRYL1/ATP5ME/BHMT2/AFMID/GCNT2/MIOX/ATP6V0C/CA2/DPYS/ACSM2B/DHTKD1/CYP3A5/AGXT2/HGD/PCK1/ELOVL7/ENTPD5/CKB/GAPDH/UGT2B7/ATP5MG/ENO1/GLDC/BHMT/CS/PFKFB2/AK4/NDUFA4/ALDOB/ALDH1L1/ANPEP/ACMSD/LDHB/ASS1/ALDH6A1/GPX3
hsa03010	Ribosome	63	-0.71597672	-2.305337767	4.83583270601825e-09	6.891061606076e-07	5.54848173637883e-07	1178	tags=76%, list=20%, signal=61%	RPS29/RPS9/DAP3/RPS3A/RPL13A/RPLP1/RPL31/RPL13/RPL35/RPL30/RPL5/RPS8/MRPS25/RPS16/RPL18/RPL32/MRPL33/RPL34/RPL26/RPS24/RPL37/MRPL48/RPL14/RPS27A/RACK1/RPL7A/RPS3/RPL10/RPS6/RPL11/RPL3/RPL9/RPL36/RPS15/RPL28/MRPS21/RPL6/RPL21/RPL17/RPL29/RPLP2/RPL8/RPS25/RPL41/MRPL42/RPL37A/RPL12/RPS4X
hsa00190	Oxidative phosphorylation	44	-0.709368391	-2.167740713	1.61503520304267e-06	0.000153428344289054	0.000123536026057299	968	tags=64%, list=17%, signal=53%	SDHB/ATP6V1B2/ATP5F1C/ATP6V0E1/NDUFV2/ATP6V1D/ATP6V0A1/NDUFB2/COX6C/UQCRC2/ATP6V1E1/ATP6V1A/NDUFV3/ATP6V0A4/ATP5F1A/NDUFA13/ATP6V0D2/LHPP/ATP5F1B/NDUFB1/ATP6V0B/ATP5F1E/UQCRH/ATP5ME/PPA1/ATP6V0C/ATP5MG/NDUFA4
hsa01200	Carbon metabolism	50	-0.687623133	-2.142923405	4.17504116203522e-06	0.00029747168279501	0.000239515519295705	988	tags=56%, list=17%, signal=47%	GOT1/SDHB/RPIA/GLUD1/IDH2/ADH5/ME3/PKM/HK1/SUCLG1/PGK1/MCEE/GPI/ACO1/ACO2/PCCB/DLST/MDH1/ACAT1/OGDH/ACSS2/AGXT2/GAPDH/ENO1/GLDC/CS/ALDOB/ALDH6A1
hsa051	Corona	86	-	-	1.6303	0.0009	0.0007	1275	tags=5	IL6R/RPS12/RPS29/RPS9/RPS3A/RPL13A/RPLP1/RPL31/TRAF6/RPL13/

ID	Description	setSize	enrichmentScore	NES	pvalue	p.adjust	qvalue	rank	leading_edge	core_enrichment
71	virus disease - COVID-19		0.586986948	1.963576375	4585985582e-05	2929714011782	48242941996989		8%, list=22%, signal=46%	RPL35/RPL30/RPL5/RPS8/RPS16/RPL18/RPL32/RPL34/RPL26/RPS24/RPL37/RPL14/RPS27A/NRP1/RPL7A/RPS3/RPL10/NFKBIA/RPS6/RPL11/RPL3/RPL9/RPL36/RPS15/RPL28/RPL6/RPL21/RPL17/STAT2/RPL29/RPLP2/RPL8/RPS25/PIK3R3/RPL41/MAVS/RPL37A/RPL12/TMPRSS2/RPS4X
hsa00630	Glyoxylate and dicarboxylate metabolism	17	-0.815327843	-2.053852739	2.46308489125739e-05	0.00116996532334726	0.00094202194086686	499	tags=65%, list=9%, signal=59%	MCEE/ACO1/ACO2/PCCB/MDH1/ACAT1/ACSS2/AFMID/AGXT2/GLDC/CS
hsa01230	Biosynthesis of amino acids	20	-0.760582978	-2.001287874	8.40576778772502e-05	0.00299455477437704	0.00241112812858428	1034	tags=80%, list=18%, signal=66%	GLUL/GOT1/RPIA/IDH2/MAT2A/PKM/PGK1/ACO1/ACO2/BCAT2/PAH/GAPDH/ENO1/CS/ALDOB/ASS1
hsa00010	Glycolysis / Gluconeogenesis	17	-0.79294744	-1.997475355	7.94452650596469e-05	0.00299455477437704	0.00241112812858428	869	tags=71%, list=15%, signal=60%	ADH5/ALDH2/PKM/HK1/PGK1/GPI/ACSS2/PCK1/GAPDH/ENO1/ALDOB/LDHB
hsa00670	One carbon pool by folate	19	-0.76079814	-1.970902678	0.000200964882548975	0.00636388794738421	0.00512401688838323	870	tags=63%, list=15%, signal=54%	AHCYL2/MTHFS/MAT2A/DMGDH/FTCD/AHCYL1/DHFR/GART/BHMT2/GLDC/BHMT/ALDH1L1
hsa04820	Cytoskeleton in muscle cells	85	0.392919983423859	1.76176621779686	0.000341452652713708	0.00973140060234069	0.0078354398201672	976	tags=44%, list=17%, signal=37%	BGN/ITGB3/FHL1/THBS1/DCN/VIM/AGRN/CSRP1/DSC2/DTNA/OBSCN/NEBL/ITGB8/ITGA9/DAAM2/FMN2/LMNA/TPM4/MYH9/TMOD2/ITGAV/COL4A3/COL4A4/TMOD3/PDLIM5/ITGB5/ITGA3/SGCB/MYL3/SYNE1/ITGA1/ITGB1/TMOD1/SNTB1/SGCE/ACTB/COL4A5
hsa00620	Pyruvate metabolism	20	-0.723014274	-1.902435029	0.000465764772631575	0.0120675418363635	0.00971643257738597	869	tags=50%, list=15%,	ADH5/ALDH2/ME3/PKM/HAGH/MDH1/ACAT1/ACSS2/PCK1/LDHB

ID	Description	setSize	enrichmentScore	NES	pvalue	p.adjust	qvalue	rank	leading_edge	core_enrichment
									signal=43%	
hsa00280	Valine, leucine and isoleucine degradation	32	-0.665259137	-1.924033153	0.000563039551006824	0.0123435593874573	0.00993867385582896	809	tags=50%, list=14%, signal=43%	ALDH2/ACSF3/MCEE/HADHB/AOX1/PCCB/EHHADH/BCAT2/ABAT/ACADSB/HMGCS1/HADH/ACAT1/DBT/AGXT2/ALDH6A1
hsa00270	Cysteine and methionine metabolism	17	-0.750856418	-1.891445907	0.000545259240601342	0.0123435593874573	0.00993867385582896	988	tags=65%, list=17%, signal=54%	GOT1/AHCYL2/AMD1/MAT2A/AHCYL1/BCAT2/MDH1/BHMT2/AGXT2/BHMT/LDHB
hsa04966	Collecting duct acid secretion	13	-0.827662477	-1.970013001	0.00102511695201563	0.0194772220882969	0.0156824909150461	930	tags=92%, list=16%, signal=78%	ATP6V1B2/ATP6V0E1/ATP6V1D/ATP6V0A1/CLCNKB/ATP6V1E1/ATP6V1A/ATP6V0A4/ATP6V0D2/ATP6V0C/CA2/SLC4A1
hsa05415	Diabetic cardiomyopathy	67	-0.555400127	-1.806345866	0.000998130057430832	0.0194772220882969	0.0156824909150461	1141	tags=40%, list=20%, signal=33%	MTOR/CAMK2G/PLCB1/SDHB/ATP5F1C/NDUFV2/TGFBR2/NDUFB2/COX6C/GYS1/UQCRC2/NDUFV3/ATP5F1A/NDUFA13/TBC1D4/INSR/VDAC1/GSR/ATP5F1B/MPC1/PIK3R3/NDUFB1/ATP5F1E/UQCRH/PPARA/GAPDH/NDUFA4
hsa00380	Tryptophan metabolism	18	-0.780737573	-2.002503225	0.00111669609951646	0.0198911492726369	0.0160157730062229	809	tags=61%, list=14%, signal=53%	ALDH2/MAOB/AOX1/EHHADH/DLST/HADH/MAOA/ACAT1/AFMID/DHTKD1/ACMSD
hsa00640	Propanoate metabolism	24	-0.714061282	-1.939713989	0.00124374647592325	0.0208510438610662	0.0167886521208216	596	tags=42%, list=10%, signal=38%	SUCLG1/ACSS3/MCEE/PCCB/EHHADH/ABAT/DBT/ACSS2/LDHB/ALDH6A1
hsa047	Therm	92	-	-	0.0013	0.0218	0.0175	1487	tags=4	GNAS/PRKAA2/COX7A2L/MAPK14/SMARCC1/NDUFAF5/COX10/NDU

ID	Description	setSize	enrichmentScore	NES	pvalue	p.adjust	qvalue	rank	leading_edge	core_enrichment
14	ogenesis		0.509393181	1.726172295	774071841331	089470821074	599278445038		5%, list=26%, signal=34%	FAF7/ACSL4/MTOR/PRDM16/KDM3A/SDHB/ATP5F1C/MAP2K3/MAP3K5/NDUFV2/SMARCB1/NDUFB2/COX6C/SMARCA4/RPS6/COX19/UQCRC2/PRKAG2/MGLL/NDUFV3/PPARG/ATP5F1A/ADCY9/NDUFA13/COX20/PPARGC1A/ATP5F1B/NDUFB1/ATP5F1E/UQCRH/ATP5ME/ATP5MG/NDUFA4/DPF3
hsa00650	Butanoate metabolism	11	-0.775032395	-1.76895011	0.0018131176199303	0.0271967642989545	0.0218980410606541	352	tags=73%, list=6%, signal=68%	L2HGDH/EHHADH/ABAT/HMGCS1/HADH/ACAT1/ACSM2A/ACSM2B
hsa00330	Arginine and proline metabolism	12	-0.804465309	-1.871289604	0.00214355646513759	0.0303355647447727	0.0244253115950525	988	tags=83%, list=17%, signal=69%	GOT1/PRODH2/AMD1/ALDH2/MAOB/SAT1/CNDP2/MAOA/P4HA1/CKB
hsa05012	Parkinson disease	92	-0.499368704	-1.692202514	0.00223525213908852	0.0303355647447727	0.0244253115950525	1487	tags=43%, list=26%, signal=33%	GNAS/COX7A2L/GNAI3/UBE2G2/CALM1/VDAC2/SLC39A9/CAMK2G/KLC1/MCU/SDHB/UBA1/ATP5F1C/UBE2L3/MAP3K5/NDUFV2/RPS27A/ITPR2/NDUFB2/UBE2J2/COX6C/EIF2S1/MAOB/SLC39A11/BCL2L1/UBE2G1/UBC/UQCRC2/TRAP1/NDUFV3/ATP5F1A/NDUFA13/VDAC1/ATP5F1B/MAOA/NDUFB1/ATP5F1E/UQCRH/SLC39A14/NDUFA4
hsa00020	Citrate cycle (TCA cycle)	17	-0.701736927	-1.767711385	0.0030103893652141	0.0389982258675463	0.0314002335701758	968	tags=59%, list=17%, signal=49%	SDHB/IDH2/SUCLG1/ACO1/ACO2/DLST/MDH1/OGDH/PCK1/CS
hsa04972	Pancreatic secretion	30	-0.624121035	-1.777131533	0.00333459174616873	0.0408258944887659	0.0328718190158854	615	tags=37%, list=11%, signal=33%	ITPR2/KCNQ1/ATP1B1/TPCN2/CHRM3/ADCY9/PLA2G12A/ATP1A1/FXYD2/CA2/SLC4A4
hsa04721	Synaptic vesicle cycle	29	-0.62385614	-1.763801582	0.00343797006221186	0.0408258944887659	0.0328718190158854	1206	tags=55%, list=21%, signal=	AP2A2/UNC13B/DNM2/ATP6V1B2/ATP6V0E1/ATP6V1D/ATP6V0A1/SLC6A13/ATP6V1E1/ATP6V1A/ATP6V0A4/STX3/ATP6V0D2/SLC1A1/ATP6V0B/ATP6V0C

ID	Description	setSize	enrichmentScore	NES	pvalue	p.adjust	qvalue	rank	leading_edge	core_enrichment
									44%	
hsa04978	Mineral absorption	12	-0.784040109	-1.823777966	0.00425476494577289	0.048504320381811	0.0390542635022522	486	tags=58%, list=8%, signal=54%	FTH1/FTL/ATP1B1/SLC8A1/ATP1A1/FXYD2/SLC26A6

Supplementary Table S8. Mitochondrial-related DEGs identified by intersection with the MitoCarta 3.0 mitochondrial gene inventory.

Gene_Symbol	p_val	avg_log2FC	pct.1	pct.2	p_val_adj	Significance
MT-CO2	2.26051935282773e-43	1.52873837694816	0.726	0.518	6.53742196837779e-39	Up-regulated in DKD
MT-ND4L	8.38774565825654e-26	0.544863022922503	0.428	0.199	2.42573604436779e-21	Up-regulated in DKD
MT-ND4	1.02131665126364e-17	0.53405139722659	0.585	0.407	2.95364775545446e-13	Up-regulated in DKD
MT-CYB	3.76061359004302e-16	0.470818292797579	0.545	0.362	1.08756945024044e-11	Up-regulated in DKD
MT-ND2	3.33170155815999e-11	0.297125803016826	0.553	0.386	9.63528090619868e-07	Up-regulated in DKD
FKBP8	1.535028058439e-09	1.35481924129333	0.104	0.037	4.43930114500559e-05	Up-regulated in DKD
MT-CO3	2.37078453031717e-09	0.424820301906495	0.648	0.544	6.85630886167727e-05	Up-regulated in DKD
FBXL4	8.12693234713012e-09	0.393326721707495	0.433	0.309	0.000235030883479003	Up-regulated in DKD
MSRB3	6.41710300126927e-08	0.427359806632511	0.575	0.457	0.00185582618796707	Up-regulated in DKD
GLS	1.01481354768279e-07	0.313521757882654	0.756	0.697	0.00293484077989863	Up-regulated in DKD
ACSM2B	1.94133467912303e-32	-1.392969139	0.108	0.336	5.61433989202379e-28	Down-regulated in DKD
ACSM2A	4.11380897141849e-29	-1.041327662	0.168	0.401	1.18971355453423e-24	Down-regulated in DKD
GLDC	8.26861614011488e-26	-1.567980196	0.06	0.227	2.39128378772122e-21	Down-regulated in DKD
ALDH6A1	3.35398780843471e-16	-2.036813404	0.057	0.173	9.69973274199317e-12	Down-regulated in DKD
LDHB	1.76418712325824e-14	-1.88398678	0.055	0.159	5.10202916046282e-10	Down-regulated in DKD
SUGCT	1.29149512793968e-13	-1.010918459	0.105	0.227	3.73500391000156e-09	Down-regulated in DKD
OXR1	3.0838756893276e-13	-0.716643561	0.413	0.541	8.91856849353541e-09	Down-regulated in DKD
FHIT	9.73877700335246e-13	-0.514954483	0.694	0.772	2.81645430936953e-08	Down-regulated in DKD
AGXT2	2.56756426718334e-12	-1.421907364	0.059	0.157	7.42539586069423e-08	Down-regulated in DKD
ALDH1L1	2.82899096867444e-12	-1.726354607	0.057	0.152	8.18144188140649e-08	Down-regulated in DKD
EFHD1	9.64029334292141e-12	-1.079051914	0.095	0.203	2.78797283477287e-07	Down-regulated in DKD
OGDH	4.1677174698555e-11	-1.054285956	0.242	0.356	1.20530389228221e-06	Down-regulated in DKD
DHTKD1	2.3294166959894e-10	-1.395500103	0.106	0.203	6.73667308480134e-06	Down-regulated in DKD
AK4	3.95876552925782e-10	-1.646452077	0.073	0.161	1.14487499106136e-05	Down-regulated in DKD
MPC1	4.81997307633004e-09	-0.993285978	0.12	0.214	0.000139393621367465	Down-regulated in DKD
GLYAT	8.74632605536201e-09	-1.414256614	0.065	0.143	0.000252943749521069	Down-regulated in DKD
ACAT1	6.42569464632808e-08	-1.021769659	0.074	0.151	0.00185831089171808	Down-regulated in DKD
ACSF2	1.04738994974485e-07	-0.723319976	0.09	0.171	0.00302905173466211	Down-regulated in DKD
CHCHD10	1.16214959014439e-07	-1.325708845	0.047	0.111	0.00336093661469757	Down-regulated in DKD
MAOA	1.00990985316828e-06	-1.005897122	0.126	0.203	0.0292065929536266	Down-regulated in DKD
UQCRH	1.58122301133002e-06	-1.147522623	0.055	0.114	0.0457289694876641	Down-regulated in DKD

Supplementary Table S9. Mitochondrial functional module scores in Control and DKD podocytes.

Pathway	N_Cells_Control	Mean_Control	SE_Control	N_Cells_DKD	Mean_DKD	SE_DKD	Exact_P_Value	Significance
FAO	1136	0.0078	0.0074	932	-0.0341	0.0068	1.28e-03	**
Mito_Dynamics	1136	0.0043	0.006	932	-4.00E-04	0.0065	8.69e-01	ns
OXPHOS	1136	0.1769	0.0194	932	0.3689	0.0188	8.88e-17	****
TCA_Cycle	1136	0.0173	0.0057	932	-0.0247	0.0048	6.92e-05	****

Supplementary Table S10. Gene Ontology (GO) enrichment analysis of mitochondrial-related DEGs in DKD podocytes.

GO_ID	Description	Count	Fold_Enrichment	pvalue	p.adjust	GeneRatio	BgRatio	Enriched_Genes
GO:0006637	acyl-CoA metabolic process	6	47.94	2.15e-09	4.04e-07	6/24	99/18986	ACSF2/ACAT1/ACSM2A/OGDH/ACSM2B/GLYAT
GO:0035383	thioester metabolic process	6	47.94	2.15e-09	4.04e-07	6/24	99/18986	ACSF2/ACAT1/ACSM2A/OGDH/ACSM2B/GLYAT
GO:1901606	alpha-amino acid catabolic process	5	40.36	1.30e-07	1.63e-05	5/24	98/18986	GLS/ACAT1/AGXT2/GLDC/ALDH6A1
GO:1901605	alpha-amino acid metabolic process	6	22.71	1.89e-07	1.78e-05	6/24	209/18986	GLS/ACAT1/GLYAT/AGXT2/GLDC/ALDH6A1
GO:0009063	amino acid catabolic process	5	35.63	2.43e-07	1.82e-05	5/24	111/18986	GLS/ACAT1/AGXT2/GLDC/ALDH6A1
GO:0006544	glycine metabolic process	3	182.56	5.03e-07	2.49e-05	3/24	13/18986	GLYAT/AGXT2/GLDC
GO:0016054	organic acid catabolic process	6	19.06	5.29e-07	2.49e-05	6/24	249/18986	GLS/ACAT1/AGXT2/GLDC/ALDH1L1/ALDH6A1
GO:0046395	carboxylic acid catabolic process	6	19.06	5.29e-07	2.49e-05	6/24	249/18986	GLS/ACAT1/AGXT2/GLDC/ALDH1L1/ALDH6A1
GO:0006520	amino acid metabolic process	6	16.42	1.26e-06	5.27e-05	6/24	289/18986	GLS/ACAT1/GLYAT/AGXT2/GLDC/ALDH6A1
GO:0006091	generation of precursor metabolites and energy	7	11.3	1.73e-06	6.50e-05	7/24	490/18986	ACAT1/OGDH/UQCRH/CHCHD10/DHTKD1/AK4/ALDH1L1
GO:0006790	sulfur compound metabolic process	6	14.04	3.13e-06	1.07e-04	6/24	338/18986	ACSF2/ACAT1/ACSM2A/OGDH/ACSM2B/GLYAT
GO:0044282	small molecule catabolic process	6	12.66	5.68e-06	1.78e-04	6/24	375/18986	GLS/ACAT1/AGXT2/GLDC/ALDH1L1/ALDH6A1
GO:0009069	serine family amino acid metabolic process	3	74.16	8.59e-06	2.49e-04	3/24	32/18986	GLYAT/AGXT2/GLDC
GO:0006163	purine nucleotide metabolic process	6	10.79	1.41e-05	3.80e-04	6/24	440/18986	FHIT/OGDH/DHTKD1/AK4/ALDH1L1/LDHB
GO:0043603	amide metabolic process	6	10.06	2.10e-05	5.27e-04	6/24	472/18986	ACSF2/ACAT1/ACSM2A/OGDH/ACSM2B/GLYAT
GO:0009117	nucleotide metabolic process	6	9.49	2.91e-05	6.84e-04	6/24	500/18986	FHIT/OGDH/DHTKD1/AK4/ALDH1L1/LDHB
GO:0019362	pyridine nucleotide metabolic process	4	18.4	6.00e-05	1.24e-03	4/24	172/18986	OGDH/DHTKD1/ALDH1L1/LDHB
GO:0046496	nicotinamide nucleotide metabolic process	4	18.4	6.00e-05	1.24e-03	4/24	172/18986	OGDH/DHTKD1/ALDH1L1/LDHB
GO:0170039	proteinogenic amino acid metabolic process	4	18.19	6.27e-05	1.24e-03	4/24	174/18986	GLS/GLYAT/AGXT2/GLDC
GO:0072524	pyridine-containing compound metabolic process	4	17.78	6.85e-05	1.29e-03	4/24	178/18986	OGDH/DHTKD1/ALDH1L1/LDHB

GO_ID	Description	Count	Fold_Enrichment	pvalue	p.adjust	GeneRatio	BgRatio	Enriched_Genes
GO:0170033	L-amino acid metabolic process	4	17.48	7.31e-05	1.31e-03	4/24	181/18986	GLS/GLYAT/AGXT2/GLDC
GO:1901292	nucleoside phosphate catabolic process	4	17.2	7.80e-05	1.33e-03	4/24	184/18986	FHIT/ACAT1/OGDH/DHTKD1
GO:0170040	proteinogenic amino acid catabolic process	3	33.43	9.59e-05	1.57e-03	3/24	71/18986	GLS/AGXT2/GLDC
GO:0009060	aerobic respiration	4	15.82	1.08e-04	1.69e-03	4/24	200/18986	OGDH/UQCRH/CHCHD10/AK4
GO:0170035	L-amino acid catabolic process	3	31.64	1.13e-04	1.70e-03	3/24	75/18986	GLS/AGXT2/GLDC
GO:0046434	organophosphate catabolic process	4	13.08	2.24e-04	3.24e-03	4/24	242/18986	FHIT/ACAT1/OGDH/DHTKD1
GO:0045333	cellular respiration	4	12.71	2.50e-04	3.48e-03	4/24	249/18986	OGDH/UQCRH/CHCHD10/AK4
GO:0043648	dicarboxylic acid metabolic process	3	23.73	2.65e-04	3.56e-03	3/24	100/18986	GLS/OGDH/ALDH1L1
GO:0046031	ADP metabolic process	3	22.82	2.97e-04	3.85e-03	3/24	104/18986	OGDH/DHTKD1/AK4
GO:0009083	branched-chain amino acid catabolic process	2	71.92	3.48e-04	4.37e-03	2/24	22/18986	ACAT1/ALDH6A1
GO:0009135	purine nucleoside diphosphate metabolic process	3	20.46	4.10e-04	4.81e-03	3/24	116/18986	OGDH/DHTKD1/AK4
GO:0009179	purine ribonucleoside diphosphate metabolic process	3	20.46	4.10e-04	4.81e-03	3/24	116/18986	OGDH/DHTKD1/AK4
GO:0006090	pyruvate metabolic process	3	18.84	5.22e-04	5.77e-03	3/24	126/18986	OGDH/DHTKD1/LDHB
GO:0009185	ribonucleoside diphosphate metabolic process	3	18.84	5.22e-04	5.77e-03	3/24	126/18986	OGDH/DHTKD1/AK4
GO:0009081	branched-chain amino acid metabolic process	2	56.51	5.67e-04	6.10e-03	2/24	28/18986	ACAT1/ALDH6A1
GO:0009132	nucleoside diphosphate metabolic process	3	17.84	6.11e-04	6.38e-03	3/24	133/18986	OGDH/DHTKD1/AK4
GO:0046394	carboxylic acid biosynthetic process	4	9.56	7.32e-04	7.30e-03	4/24	331/18986	GLS/ACSM2A/ACSM2B/AGXT2
GO:0042219	cellular modified amino acid catabolic process	2	49.44	7.42e-04	7.30e-03	2/24	32/18986	AGXT2/ALDH1L1
GO:0016053	organic acid biosynthetic process	4	9.47	7.57e-04	7.30e-03	4/24	334/18986	GLS/ACSM2A/ACSM2B/AGXT2
GO:0006119	oxidative phosphorylation	3	15.93	8.50e-04	7.99e-03	3/24	149/18986	UQCRH/CHCHD10/AK4
GO:0072523	purine-containing compound catabolic process	3	15.31	9.52e-04	8.59e-03	3/24	155/18986	ACAT1/OGDH/DHTKD1
GO:0015980	energy derivation by oxidation of organic compounds	4	8.89	9.60e-04	8.59e-03	4/24	356/18986	OGDH/UQCRH/CHCHD10/AK4
GO:0009166	nucleotide catabolic process	3	14.74	1.06e-03	9.29e-03	3/24	161/18986	FHIT/OGDH/DHTKD1
GO:0035337	fatty-acyl-CoA metabolic process	2	38.59	1.22e-03	1.04e-02	2/24	41/18986	ACAT1/ACSM2A
GO:0006631	fatty acid metabolic process	4	7.81	1.55e-03	1.29e-02	4/24	405/18986	ACSF2/ACAT1/ACSM2A/ACSM2B
GO:0170038	proteinogenic amino acid biosynthetic process	2	27.28	2.42e-03	1.98e-02	2/24	58/18986	GLS/AGXT2

GO_ID	Description	Count	Fold_Enrichment	pvalue	p.adjust	GeneRatio	BgRatio	Enriched_Genes
GO:0170034	L-amino acid biosynthetic process	2	26.82	2.51e-03	2.01e-02	2/24	59/18986	GLS/AGXT2
GO:0046034	ATP metabolic process	3	10.41	2.87e-03	2.25e-02	3/24	228/18986	OGDH/DHTKD1/AK4
GO:1901607	alpha-amino acid biosynthetic process	2	24.34	3.03e-03	2.33e-02	2/24	65/18986	GLS/AGXT2
GO:0009205	purine ribonucleoside triphosphate metabolic process	3	9.42	3.81e-03	2.86e-02	3/24	252/18986	OGDH/DHTKD1/AK4
GO:0008652	amino acid biosynthetic process	2	21.38	3.91e-03	2.88e-02	2/24	74/18986	GLS/AGXT2
GO:0009199	ribonucleoside triphosphate metabolic process	3	9.2	4.06e-03	2.91e-02	3/24	258/18986	OGDH/DHTKD1/AK4
GO:0009144	purine nucleoside triphosphate metabolic process	3	9.16	4.11e-03	2.91e-02	3/24	259/18986	OGDH/DHTKD1/AK4
GO:1901568	fatty acid derivative metabolic process	2	20.03	4.45e-03	3.10e-02	2/24	79/18986	ACAT1/ACSM2A
GO:0009141	nucleoside triphosphate metabolic process	3	8.66	4.81e-03	3.29e-02	3/24	274/18986	OGDH/DHTKD1/AK4
GO:0006096	glycolytic process	2	16.48	6.50e-03	4.36e-02	2/24	96/18986	OGDH/DHTKD1
GO:0046032	ADP catabolic process	2	16.14	6.76e-03	4.46e-02	2/24	98/18986	OGDH/DHTKD1
GO:0019364	pyridine nucleotide catabolic process	2	15.82	7.03e-03	4.49e-02	2/24	100/18986	OGDH/DHTKD1
GO:0009137	purine nucleoside diphosphate catabolic process	2	15.67	7.17e-03	4.49e-02	2/24	101/18986	OGDH/DHTKD1
GO:0009181	purine ribonucleoside diphosphate catabolic process	2	15.67	7.17e-03	4.49e-02	2/24	101/18986	OGDH/DHTKD1
GO:0072526	pyridine-containing compound catabolic process	2	15.36	7.44e-03	4.59e-02	2/24	103/18986	OGDH/DHTKD1
GO:0009191	ribonucleoside diphosphate catabolic process	2	15.21	7.58e-03	4.60e-02	2/24	104/18986	OGDH/DHTKD1
GO:0009134	nucleoside diphosphate catabolic process	2	14.65	8.15e-03	4.87e-02	2/24	108/18986	OGDH/DHTKD1

Supplementary Table S11. Machine-learning feature importance and network centrality metrics of 31 mitochondrial-related differentially expressed genes in DKD podocytes.

Gene	Degree	Betweenness	Closeness	PageRank	RF Score	SVM Score	LASSO
MT-CO2	9	0	0.122351189726072	0.0455410084353887	53.9200802228053	0.690638125	Yes
FKBP8	0	0	NA	0.00497512437810945	4.54202612635177	NA	Yes
MT-ND4L	7	0	0.120723219192066	0.0309967622037072	19.6638571732717	0.6144825	Yes
MT-ND4	7	0	0.11658974310087	0.0385878005737716	25.2056112888429	0.616119375	Yes
MT-CYB	11	13	0.143279598664589	0.0465185166722592	18.6735563349224	0.596745625	No
MSRB3	21	30	0.181431059973166	0.0528388074246937	22.301740379055	0.569638541666667	Yes
MT-CO3	12	2	0.148843623439555	0.0503602155138957	21.2695640772494	0.59241125	Yes
FBXL4	7	5	0.150566406046321	0.0186159240443462	16.6512229875138	NA	Yes
GLS	15	30	0.165152027864079	0.0396102353788149	27.8682571824066	0.5751375	Yes
MT-ND2	12	1	0.144312961861117	0.0458715580734973	16.3114043952042	0.570995833333333	Yes
FHIT	16	17	0.172971471999545	0.0392273926835822	30.7858189241974	0.596819375	Yes
OXR1	16	50	0.188061538422045	0.0360542353975874	26.5416110272755	0.59345	Yes
ACSF2	15	24	0.162403148927106	0.0424629014909405	8.1190085290246	NA	Yes
MPC1	11	21	0.178670920529171	0.0252436900195341	9.1070227740083	NA	Yes
MAOA	3	1	0.116400115243182	0.00922048891519019	11.462926796963	NA	Yes
SUGCT	15	16	0.167542615106761	0.0394600503988262	8.65658949080596	0.568015625	No
ACAT1	5	0	0.146706451821078	0.0136754604165808	6.18553307821144	NA	No
ACSM2A	21	20	0.167372065153164	0.0673281931909511	24.140096181167	0.615373125	Yes
OGDH	6	1	0.139644571251327	0.0168832743202899	18.0708126647735	0.57662125	Yes
EFHD1	5	0	0.146249051135018	0.0135595012400899	6.82243761069586	NA	Yes
UQCRH	2	0	0.125662127743497	0.00791060784512942	6.07117653075589	NA	Yes
CHCHD10	10	39	0.177394405235479	0.0201931177619525	5.69932613120211	NA	Yes
ACSM2B	16	0	0.145901268730596	0.0569161847240245	21.9384257653416	0.614639375	Yes
DHTKD1	7	1	0.148762183117864	0.018833350858015	7.99895567489694	NA	Yes
GLYAT	12	10	0.168118498824515	0.0329985661516107	5.5117928501223	NA	No
AGXT2	13	5	0.158267659894302	0.0385702979692056	6.50758990511084	NA	Yes
GLDC	16	22	0.166121638325432	0.042485158510543	12.7306690801797	0.57652125	Yes
AK4	9	0	0.137061571436721	0.0258722608809499	6.99488493158403	NA	Yes
ALDH1L1	14	12	0.163811197179059	0.0342110414223083	9.76545038307917	NA	Yes
LDHB	8	10	0.162268616967323	0.0186879715462022	10.6555696891906	NA	Yes
ALDH6A1	11	11	0.157501109196905	0.0262903015580026	8.03895213122505	NA	Yes

Supplementary Table S12. Consensus machine-learning candidate genes and their network-topological characteristics in DKD podocytes.

Gene	avg_log2FC	Adjusted P-value	RF importance	RF rank	SVM importance	SVM rank	LASSO retained	Degree	Betweenness	Closeness	PageRank
ACSM2A	-1.0413	1.19E-24	24.1401	6	0.615373	3	Yes	21	20	0.167372	0.067328
ACSM2B	-1.3930	5.61E-28	21.9384	8	0.614639	4	Yes	16	0	0.145901	0.056916
FHIT	-0.5150	2.82E-08	30.7858	2	0.596819	6	Yes	16	17	0.172971	0.039227
OXR1	-0.7166	8.92E-09	26.5416	4	0.593450	8	Yes	16	50	0.188062	0.036054
MT-CO2	1.5287	6.54E-39	53.9201	1	0.690638	1	Yes	9	0	0.122351	0.045541
MT-CO3	0.4248	6.86E-05	21.2696	9	0.592411	9	Yes	12	2	0.148844	0.050360
MT-ND4	0.5341	2.95E-13	25.2056	5	0.616119	2	Yes	7	0	0.116590	0.038588
MT-ND4L	0.5449	2.43E-21	19.6639	10	0.614483	5	Yes	7	0	0.120723	0.030997

Note: Eight mitochondrial-related candidate genes were identified by intersecting the top 10 genes ranked by Random Forest (RF) importance, the top 10 genes ranked by support vector machine–recursive feature elimination (SVM-RFE), and genes with nonzero coefficients in LASSO regression at lambda.1se. The table presents the original differential expression statistics (avg_log2FC and adjusted P value), RF importance (MeanDecreaseGini) and rank, SVM-RFE importance and rank, LASSO selection status, and four co-expression network centrality measures (degree, betweenness, closeness, and PageRank). RF and SVM-RFE rankings were determined among the 31 mitochondrial-related differentially expressed genes.

Abbreviations: DKD, diabetic kidney disease; RF, Random Forest; SVM-RFE, support vector machine–recursive feature elimination; LASSO, least absolute shrinkage and selection operator; avg_log2FC, average log2 fold change.

Supplementary Table S13. Clinical characteristics of donors included in the GSE209781 single-cell RNA-sequencing cohort.

Donor	Group	Sex	Age (years)	Scr (umol/L)	eGFR	24-h proteinuria (g)	Pathological diagnosis	Tissue collection
DKD01	DKD	Male	47	166	41	0.64	DKD, diffuse and nodular glomerulosclerosis	Needle biopsy
DKD02	DKD	Female	30	54	123	9.4	DKD, diffuse and nodular glomerulosclerosis	Needle biopsy
DKD03	DKD	Female	41	110.1	54	9.46	DKD, diffuse and nodular glomerulosclerosis	Needle biopsy
NM01	Reference	Male	63	60	102	0.11	Clear cell carcinoma	Wedge biopsy from nephrectomy specimen
NM02	Reference	Male	42	81	95	0.05	Angiomyolipoma	Wedge biopsy from nephrectomy specimen
NM03	Reference	Female	64	52	97	0.09	Clear cell carcinoma	Wedge biopsy from nephrectomy specimen

Note: Clinical data were extracted from Supplementary Table S1 of Lv et al. Diabetes type, disease duration, HbA1c levels, and glucose-lowering medications were not reported specifically for the three DKD donors included in the single-cell RNA-sequencing cohort. Scr, serum creatinine; eGFR, estimated glomerular filtration rate; DKD, diabetic kidney disease.

Supplementary Table S14. External validation of candidate mitochondrial hub genes in GSE195460 and GSE209781.

Dataset	Clinical_Group	Hub_Gene	Average_Expression	Percent_Expressed	Scaled_Expression
GSE195460 (Discovery)	Control	MT-CO2	1.73767605633803	42.0774647887324	-0.707106781
GSE195460 (Discovery)	DKD	MT-CO2	3.60622317596567	64.9141630901288	0.707106781186548
GSE209781 (Validation)	Control	MT-CO2	189.801871287591	99.5211674393859	-0.707106781
GSE209781 (Validation)	DKD	MT-CO2	202.729560483952	99.8768472906404	0.707106781186548
GSE195460 (Discovery)	Control	FHIT	3.31338028169014	75.0880281690141	0.707106781186547
GSE195460 (Discovery)	DKD	FHIT	1.55793991416309	61.9098712446352	-0.707106781
GSE209781 (Validation)	Control	FHIT	0.14691085784713	1.13247700843657	0.707106781186548
GSE209781 (Validation)	DKD	FHIT	0.0638645373484225	4.74137931034483	-0.707106781
GSE195460 (Discovery)	Control	OXR1	1.13908450704225	48.2394366197183	0.707106781186547
GSE195460 (Discovery)	DKD	OXR1	0.457081545064378	25.2145922746781	-0.707106781
GSE209781 (Validation)	Control	OXR1	0.713438919168847	6.94687238732234	0.707106781186547
GSE209781 (Validation)	DKD	OXR1	0.504259383854834	28.8793103448276	-0.707106781
GSE195460 (Discovery)	Control	ACSM2A	1.08010563380282	40.580985915493	0.707106781186548
GSE195460 (Discovery)	DKD	ACSM2A	0.370171673819742	12.0171673819742	-0.707106781
GSE209781 (Validation)	Control	ACSM2A	0.410225662025512	2.28775556737858	-0.707106781
GSE209781 (Validation)	DKD	ACSM2A	0.447805354233521	14.2241379310345	0.707106781186549
GSE195460 (Discovery)	Control	MT-ND4	1.09154929577465	33.4507042253521	-0.707106781
GSE195460 (Discovery)	DKD	MT-ND4	1.20922746781116	47.4248927038627	0.707106781186548
GSE209781 (Validation)	Control	MT-ND4	187.052020659817	99.5667705403967	0.707106781186547
GSE209781 (Validation)	DKD	MT-ND4	167.458349149831	99.7536945812808	-0.707106781
GSE195460 (Discovery)	Control	MT-ND4L	0.421654929577465	16.637323943662	-0.707106781
GSE195460 (Discovery)	DKD	MT-ND4L	0.440987124463519	26.931330472103	0.70710678118655
GSE209781 (Validation)	Control	MT-ND4L	47.9565718109106	86.1062552253553	0.707106781186547
GSE209781 (Validation)	DKD	MT-ND4L	23.8596857819578	95.1354679802956	-0.707106781
GSE195460 (Discovery)	Control	MT-CO3	2.04049295774648	47.887323943662	0.707106781186548
GSE195460 (Discovery)	DKD	MT-CO3	1.8519313304721	52.1459227467811	-0.707106781
GSE209781 (Validation)	Control	MT-CO3	250.339773780404	99.7947860454511	-0.707106781
GSE209781 (Validation)	DKD	MT-CO3	263.492410188091	99.9384236453202	0.707106781186547
GSE195460 (Discovery)	Control	ACSM2B	0.855633802816901	36.3556338028169	0.707106781186548
GSE195460 (Discovery)	DKD	ACSM2B	0.227467811158798	7.93991416309013	-0.707106781
GSE209781 (Validation)	Control	ACSM2B	0.405400011305881	2.52337158926807	-0.707106781
GSE209781 (Validation)	DKD	ACSM2B	0.499283837664773	15.2709359605911	0.707106781186547

Supplementary Table S15. Donor-level podocyte characteristics and OXR1 expression in GSE271188.

GEO accession	Sample ID	Group	Podocytes (n)	OXR1-positive (%)	Mean OXR1 expression	OXR1 CPM	≥10 podocytes
GSM8371526	kidney_snRNA_14	Control	34	52.9	1.110	364.5	Yes
GSM8371527	kidney_snRNA_15	Control	1	0.0	0.000	0.0	No
GSM8371528	kidney_snRNA_16	Control	410	70.7	1.291	531.7	Yes
GSM8371529	kidney_snRNA_17	Control	79	84.8	1.403	495.1	Yes
GSM8371530	kidney_snRNA_18	Control	205	57.1	0.793	239.0	Yes
GSM8371532	kidney_snRNA_20	Control	215	64.2	1.136	342.4	Yes
GSM8371535	kidney_snRNA_23	Control	592	55.6	0.978	341.1	Yes
GSM8371531	kidney_snRNA_19	DKD	55	47.3	0.540	181.1	Yes
GSM8371534	kidney_snRNA_22	DKD	4	25.0	0.340	41.1	No
GSM8371537	kidney_snRNA_25	DKD	95	45.3	0.650	158.4	Yes
GSM8842282	kidney_snRNA_29	DKD	61	45.9	0.710	184.5	Yes

Supplementary Table S16. ChEA3 transcription factor enrichment analysis of podocyte DEGs.

Rank	TF	Score	Overlapping_Genes
1	HNF1B	9.333	CPM,ARHGAP8,THRB,GDA,WWC1,MIA2,DEFB1,TFCP2L1,AQP2,SLC4A4,PCAT1,PNN,SULT1C2,MECOM,CDH1,ANPEP,CTSH,CHP1,LINC00970,TINAG,TNS1,LINC01781,DSP,CADPS2,CUBN,CCDC198,HGD,LIMCH1,PAQR5,KAZN,WDR72,ANK3,ATP1B1,KIAA1217,TOX3,BACE2,CLDN10,DDAH1,PAX8,MYO3B,HNF1A-AS1,ADGRF5,SCNN1A,PPARG,PLPP3,PPARA,CDH16,DSCAML1,SLC27A2,SGK2,PFKFB2,RBM47,EPAS1,SLC1A1,HPN,GLIS3,CLCNKB,SLC3A1,TMPRSS2,UMOD,LRP2,EFNA5,CYP3A5,PKHD1,ABLIM1,ADGRG1,EPB41L1,AFMID,SPP1,LINC02532,KCNJ1,SLC12A1,SLC16A12,KCNJ16,LAMB1,ESRRG,DHRS3,BICC1,SLC2A9,FXYD2,CLNK,RGL3
2	ARID3C	11.5	ALDH1L1,GLDC,DPYS,HPN,AFM,ACSM2A,PLG,GLYAT,EFNA5,ACSM2B,FTCD,ACMSD,DHTKD1,AFMID,PCK1,HGD,AGXT2,BHMT2,BHMT,ALDH6A1,PAH,MPPED2,SLC28A1,SLC27A2,UGT2B7
3	TFCP2L1	12	CPM,ARHGAP8,WWC1,DEFB1,AQP2,SIPA1L3,SLC4A4,CDH1,ANPEP,RNF152,TINAG,TNS1,DSP,CADPS2,GPX3,WDR72,LMO7,ANK3,ATP1B1,KIAA1217,PAX8,SCNN1A,PPARG,PLPP3,CDH16,LLGL2,FKBP5,RBM47,SLC22A6,EPAS1,MAOA,CLCNKB,TMPRSS2,AK4,UMOD,ATP1A1,LRP2,PKHD1,ADGRG1,MUC1,CLMN,COBLL1,ATP6V0A4,APOE,CKB,PPARGC1A,KCNJ1,SLC12A3,CMTM4,ZBTB16,ZNF804B,KCNJ16,PLEKHA6,SORBS2,ACSF2,ASS1,PLEKHA7,RGL3
4	MLXIPL	16	ALDH1L1,THRB,DPYS,ACSM2A,SLC4A4,ACSM2B,FTCD,DHTKD1,ANPEP,CTSH,TNS1,CADPS2,HGD,GPX3,BHMT2,ATP1B1,SLC39A14,POR,PPARG,ALDOB,PLPP3,PPARA,SLC27A2,SGK2,PLIN5,FKBP5,RBM47,ACSS2,EPAS1,HPN,AFM,PLG,GLYAT,CYP3A5,ACMSD,AFMID,APOE,CKB,PCK1,PPARGC1A,AGXT2,ZBTB16,PLEKHA6,CHCHD10,ASS1,DHRS3,BHMT,PAH,SLC28A1
5	NKX21	17	PFKFB2,CPM,DDX5,ARHGAP8,EPAS1,MAOA,PDE1A,GLIS3,PIK3R3,TFCP2L1,LRP2,ATP1A1,HS6ST2,MUC1,MECOM,RASSF4,CTSH,ZNF804B,KCNJ16,WDR72,LMO7,KIAA1217,PAX8,DDAH1,MPPED2,SCNN1A,ADGRF5,RGL3,PLPP3,SLC26A4,ADGRL2
6	HNF4A	18.67	CYFIP2,FHOD3,ALDH1L1,GLDC,GDA,WWC1,FAM107B,EPS8,RASSF4,CDH1,MPC1,ANPEP,DPF3,CHP1,SLC16A9,NCKAP5,TNS1,SOX5,MAGI1,CADPS2,PNISR,HGD,SLC6A13,KCTD1,KAZN,SMIM2-AS1,MITF,LMO7,PDZK1,ATP1B1,PRLR,KIAA1217,BCR,POR,CACNB4,PPA1,HNF1A-AS1,SCNN1A,HECW1,MALAT1,TLN2,PLPP3,SLC27A2,ATP6V0D2,FREM1,LLGL2,FKBP5,PFKFB2,PDE1C,EPAS1,MAOA,PDE1A,HPN,CLCNKB,GLIS3,ITPR2,CACNA1D,FHIT,ACMSD,PKHD1,ADGRG1,PDZD2,SCIN,EPB41L1,SNTG1,COBLL1,AFMID,PRDM16,SAFB2,APOE,PCK1,LINC01762,PPARGC1A,RAB11FIP3,TRPM3,SLC19A2,KL,LINC00671,CHKA,AGXT2,ZBTB16,INSR,LARP4,ACSF2,LRP1B,ABTB2,DHFR,SLC2A9,SDK1,ALDH6A1,BHMT,PAH,CRYL1,CPM,CLIC4,HSP90AB1,THRB,BNC2,DPYS,MIA2,ACSM2A,PRKAG2,PTPRJ,FMN1,PEPD,SIPA1L3,ACSM2B,FTCD,SULT1C2,MECOM,CTSH,ERC1,RNF152,TINAG,LIN52,RBM6,DSP,VAV3,SLC13A3,PDE4D,BHMT2,SLC39A14,SLC22A23,TOX3,ARHGAP10,SETBP1,PAX8,MYO3B,DDAH1,TBC1D4,MPPED2,PPARG,ALDOB,PPARA,CDH16,BDP1,SGK2,ATP6V0C,PDZRN3,DSCAML1,L3MBTL4,UGT2B7,PLIN5,RBM47,ACSS2,PLPPR1,AFM,TMPRSS2,SLC3A1,PLG,LRP2,ATP1A1,CYP3A5,FGD4,SNX29,TAF1D,ABLIM1,ABLIM2,CUX1,CLMN,SH3BP2,GCNT2,GPC5,SLC17A1,RNF130,FNIP2,FARP1,SLC12A3,MYO10,EGF,CMTM4,KCNIP4,ZNF804B,AP3D1,PLEKHA6,SORBS2,LAMB1,OXR1,ASS1,DHRS3,PLEKHA7,BICC1,NR6A1,TAOK3,LNX1,SLC28A1,RAMP1,ADGRL2
7	FOXI1	22.33	CHRM3,GALNT14,ARHGAP8,WWC1,BICDL1,ACSM2A,DEFB1,TFCP2L1,AQP2,PEPD,ACSM2B,CALB1,MECOM,ANPEP,EFHD1,RNF152,TINAG,SOX5,VAV3,SLC13A1,CUBN,MIOX,CADPS2,SLC13A3,CCDC198,TMEM52B,PAQR5,SLC6A13,WDR72,PTH2R,MITF,PDZK1,TOX3,CLDN10,TMEM213,SCNN1A,PPARG,ALDOB,CDH16,RHCG,ATP6V0D2,UGT2B7,SLC22A6,PDE1C,EPAS1,HPN,CLCNKB,SLC5A12,SLC3A1,TMPRSS2,UMOD,AK4,GLYAT,LRP2,ACMSD,PKHD1,ADGRG1,MUC1,SCIN,PRDM16,SPP1,ATP6V0A4,SLC17A1,LINC00871,RAB11FIP3,LINC01762,KCNJ1,PPARGC1A,CA12,KL,SLC12A3,LINC00671,LINC01320,AGXT2,EGF,ZBTB16,SLC12A1,ESRRB,KCNJ15,ESRRG,ACSF2,ASS1,SLC2A9,BHMT,SLC4A9,CLCN5,FXYD2,CRYL1,CLNK,SLC28A1
8	HNF4G	23	HSP90AB1,THRB,GDA,PEPD,SLC4A4,FTCD,MECOM,RASSF4,CDH1,ANPEP,TINAG,NCKAP5,SOX5,SLC13A1,DSP,MAGI1,CUBN,CADPS2,HGD,MITF,LMO7,ANK3,PDZK1,ATP1B1,SLC39A14,BCR,TOX3,POR,HNF1A-AS1,MALAT1,PPARG,ALDOB,PPARA,TOX,ATP6V0C,SGK2,SLC27A2,LLGL2,RBM47,MAOA,EPAS1,SLC1A1,HPN,SLC5A12,SLC3A1,TMPRSS2,ATP1A1,LRP2,EFNA5,CYP3A5,ABLIM1,SCIN,SH3BP2,AFMID,APOE,SLIT2,PCK1,PPARGC1A,LINC00671,CHKA,AGXT2,SORBS2,ESRRG,PLEKHA7,DHRS3,ACSF2,ASS1,LRP1B,ALDH6A1,TAOK3,COL7A1,CRYL1,BMPR1B,SLC28A1,ADGRL2

Rank	TF	Score	Overlapping_Genes
9	MYRFL	24	SLC13A1,CUBN,RBM47,MAOA,GDA,SLC1A1,RAB3IP,SLC5A12,SLC3A1,PDZK1,ATP1A1,PEPD,CYP3A5,SCIN,CDH1,ANPEP,ALDOB,PCK1,TINAG,SLC28A1
10	NR1H4	26	THRB,GLDC,EPAS1,DPYS,HPN,SLC1A1,AFM,ACSM2A,SLC3A1,PLG,PEPD,SLC4A4,ACSM2B,FTCD,ACMSD,ANPEP,CTSH,APOE,PCK1,PPARGC1A,CUBN,HGD,AGXT2,GPX3,BHMT2,WDR72,ANK3,PDZK1,ATP1B1,ASS1,BHMT,PAH,PPARG,ALDOB,PLPP3,PPARA,SLC28A1,SLC27A2,ASPA
11	PROX1	26.75	GDA,DPYS,ACSM2A,AQP2,SIPA1L3,SLC4A4,ACSM2B,FTCD,SLC8A1,DHTKD1,MECOM,CDH1,NCKAP5,TNS1,SOX5,DSP,MAGI1,CADPS2,HGD,TMEM52B,LIMCH1,BHMT2,LMO7,ANK3,ATP1B1,SLC39A14,BACE2,PAX8,SETBP1,SCNN1A,MALAT1,PLPP3,SLC27A2,DSCAML1,PDZRN3,PFKFB2,EPAS1,HPN,AFM,GLIS3,TMPRSS2,PLG,ATP1A1,GLYAT,EFNA5,CYP3A5,ACMSD,ACAT1,ADGRG1,ABLIM1,EPB41L1,CLMN,AFMID,PRDM16,SLIT2,PCK1,LINC01762,LINC02532,PPARGC1A,ZBTB16,SORBS2,ESRRG,ABTB2,ALDH6A1,BHMT,PAH,FXYD2,MAP3K13,SLC28A1,ADGRL2
12	NR1I3	31.33	ALDH1L1,GLDC,EPAS1,DPYS,HPN,AFM,ACSM2A,PLG,GLYAT,CYP3A5,ACSM2B,FTCD,ACMSD,DHTKD1,ANPEP,AFMID,APOE,PCK1,PPARGC1A,SLC36A2,HGD,BHMT2,ESRRG,ASS1,BHMT,ALDH6A1,TMEM213,PAH,PPARG,ALDOB,PPARA,SLC28A1,SLC27A2,ASPA,FKBP5,PLIN5
13	NR1I2	34.75	CHRM3,ALDH1L1,CLIC4,THRB,DPYS,WWC1,ACSM2A,PRKAG2,PTPRJ,ACSM2B,FTCD,DHTKD1,MECOM,RASSF4,CDH1,ANPEP,ERC1,TNS1,DSP,HGD,BHMT2,SLC6A13,ANK3,ATP1B1,SLC39A14,POR,MYO3B,PPA1,PPARG,ALDOB,PPARA,SGK2,SLC27A2,PDZRN3,FKBP5,NLGN1,EPAS1,MAOA,PDE1A,HPN,AFM,TMPRSS2,SLC3A1,PLG,GLYAT,EFNA5,CYP3A5,FHIT,TAF1D,ABLIM1,PDZD2,SNTG1,COBLL1,APOE,PCK1,PPARGC1A,AGXT2,ZBTB16,INSR,SLC16A12,ESRRG,BHMT,PAH,SLC28A1
14	HNF1A	41.67	THRB,EPAS1,DPYS,HPN,AFM,MIA2,ACSM2A,TMPRSS2,SLC3A1,PLG,GLYAT,LRP2,CYP3A5,ACSM2B,FTCD,ACMSD,DHTKD1,MECOM,ANPEP,AFMID,APOE,PCK1,PPARGC1A,TNS1,DSP,CUBN,HGD,AGXT2,BHMT2,ZBTB16,ANK3,ATP1B1,ASS1,BHMT,PAX8,PAH,HNF1A-AS1,PPARG,ALDOB,PPARA,SGK2,SLC28A1,SLC27A2,UGT2B7
15	AR	45.2	CHRM3,ALDH1L1,GLDC,GDA,WWC1,SLC4A4,SLC8A1,CDH1,ANPEP,CHP1,SLC16A7,TNS1,MAGI1,CADPS2,HGD,LIMCH1,PAQR5,CACNA2D3,KAZN,MITF,WDR72,LMO7,ANK3,ATP1B1,PRLR,KIAA1217,EMCN,SCNN1A,MALAT1,TLN2,PLPP3,TOX,FKBP5,PFKFB2,EPAS1,MAOA,HPN,SLC1A1,PCDH15,TMTC2,ITPR2,AK4,TMTC1,GLYAT,EFNA5,RHOBTB1,PDZD2,EPB41L1,COBLL1,AFMID,MYO6,KCNMB2,APOE,LINC01762,PPARGC1A,SLC19A2,CHKA,ZBTB16,ESRRB,ESRRG,LRP1B,DHFR,ABTB2,SDK1,RALYL,PAH,OGDH,CCSER1,BMPR1B,THRB,BNC2,DPYS,ACSM2A,PTPRJ,FMN1,ARHGAP6,SIPA1L3,ACSM2B,FTCD,DHTKD1,MECOM,GPX3,BHMT2,PDE4D,ELOVL7,SLC22A23,RP1,SETBP1,MYO3B,DDAH1,PPARG,ALDOB,PPARA,PDZRN3,UGT2B7,RBM47,AFM,TMPRSS2,PLG,FGD4,CUX1,CLMN,GPC5,CKB,SLIT2,RNF130,FNIP2,KCNJ3,CA12,MYO10,CMTM4,PLEKHA6,SORBS2,ENOX1,SLC28A1,RAMP1
16	GLIS3	47	PFKFB2,BNC2,EPAS1,WWC1,ITPR2,LRP2,EFNA5,SLC4A4,EPS8,ADGRG1,MECOM,RASSF4,PRDM16,SLIT2,TNS1,CA12,FARP1,CADPS2,CASR,MYO10,LIMCH1,PDE4D,ZNF804B,KCNJ16,WDR72,SORBS2,LMO7,NRG1,LAMB1,SLC39A14,BICC1,KIAA1217,SDK1,PAX8,DDAH1,FXYD2,RGL3,PPARG,PLPP3,SLC26A4,ADGRL2
17	CREB3L3	47.67	ALDH1L1,GLDC,EPAS1,MAOA,GDA,DPYS,HPN,SLC1A1,AFM,MIA2,SLC5A12,PLG,PEPD,SLC4A4,ACSM2B,FTCD,ACMSD,ANPEP,AFMID,CTSH,APOE,PCK1,SLC13A1,CUBN,HGD,AGXT2,GPX3,PDZK1,SLC39A14,DHRS3,ASS1,BHMT,PAH,ALDOB,PPARA,SGK2,SLC28A1,SLC27A2
18	PAX8	48	ARHGAP8,BNC2,GLDC,WWC1,DEFB1,TFCP2L1,AQP2,GNGT1,MECOM,RASSF4,CDH1,ANPEP,ERC1,TINAG,TNS1,VAV3,DSP,CUBN,MIOX,HSP90AA1,GPX3,PDE4D,WDR72,MITF,NRG1,PDZK1,ATP1B1,KIAA1217,POR,TBC1D4,MPPED2,SCNN1A,PPARG,PLPP3,CDH16,TRABD2B,FKBP5,SLC22A6,RBM47,EPAS1,MAOA,HPN,GLIS3,CLCNKB,AK4,UMOD,LRP2,ATP1A1,PKHD1,ADGRG1,MUC1,CALCR,AFMID,PRDM16,APOE,SLC17A1,KCNJ1,FARP1,CMTM4,ZBTB16,ZNF804B,SLC12A1,KCNJ15,KCNJ16,SORBS2,LAMB1,ACSF2,ASS1,NR6A1,FXYD2,LNX1,CRYL1,RGL3,SLC26A4
19	KLF15	59.33	CPM,CLIC4,ALDH1L1,MAOA,EPAS1,DPYS,HPN,AFM,ACSM2A,TMTC1,PLG,SLC4A4,FTCD,ACAT1,DHTKD1,PKHD1,PNN,APOE,CKB,PCK1,PPARGC1A,TNS1,SLC19A2,ZHX3,GPX3,INSR,BHMT2,ZBTB16,ANK3,ATP1B1,DHRS3,ASS1,ARHGAP10,ALDH6A1,PAH,TACC1,PPARG,PPARA,PLPP3,PLIN5,FKBP5
20	FOXA3	60	RBM47,GLDC,DPYS,HPN,AFM,MIA2,TMPRSS2,PLG,TFCP2L1,LRP2,CYP3A5,FTCD,DHTKD1,MUC1,MECOM,ANPEP,AFMID,APOE,C

Rank	TF	Score	Overlapping_Genes
			KB,PCK1,LINC02532,DSP,CCDC198,HGD,BHMT2,ATP1B1,SLC39A14,DHRS3,ASS1,TOX3,BHMT,PAH,HNF1A-AS1,PPARG,ALDOB,SGK2,SLC27A2,SLC28A1,LLGL2,PLIN5
21	FOXA2	60.83	CYFIP2,WWC1,HS6ST2,SLC8A1,FAM107B,PNN,RASSF4,CDH1,MPC1,ANPEP,DPF3,SLC16A9,NCKAP5,SOX5,MAGI1,CADPS2,HGD,CACNA2D3,KCTD1,KAZN,ANK3,ATP1B1,KIAA1217,BCR,POR,CACNB4,HNF1A-AS1,SCNN1A,HECW1,MALAT1,TLN2,PLPP3,TOX,LLGL2,PFKFB2,EPAS1,PDE1A,HPN,PCDH15,GLIS3,ITPR2,CACNA1D,FHIT,RHOBTB1,EPB41L1,COBLL1,AFMID,PRDM16,APOE,PCK1,LINC01762,PPARGC1A,RAB11FIP3,LINC00671,CHKA,ZBTB16,INSR,SLC16A12,CHCHD10,ESRRG,ACSF2,ABTB2,SLC2A9,SDK1,ALDH6A1,FXYD2,PAH,NMT1,CRYL1,MAP3K13,BMPR1B,CPM,CLIC4,HSP90AB1,HIP1,BNC2,MIA2,PIK3C2G,PEPD,SIPA1L3,DHTKD1,SULT1C2,MECOM,CTSH,ERC1,LINC00970,LIN52,DSP,VAV3,HSP90AA1,SLC13A3,PDE4D,NRG1,SLC39A14,SLC22A23,TOX3,DDAH1,MYO3B,TBC1D4,MPPED2,PPARG,BDP1,SGK2,ASPA,DSCAML1,UGT2B7,PLIN5,SAMD5,GRAMD1B,RBM47,PLPPR1,AFM,TMPRSS2,PLG,CYP3A5,FGD4,MUC1,SNX29,TAF1D,ABLIM1,ABLIM2,CUX1,CLMN,GCNT2,LINC02532,FNIP2,FARP1,MYO10,KCNIP4,PLEKHA6,KCNJ16,LAMB1,SORBS2,OXR1,DHRS3,BICC1,NR6A1,LNX1,ADGRL2
22	DMRT2	63	CPM,DEFB1,AQP2,PEPD,SLC4A4,CALB1,MECOM,TINAG,SOX5,TNS1,SLC13A1,CUBN,MIOX,CADPS2,CASR,SLC13A3,CCDC198,SLC36A2,TMEM52B,PAQR5,GPX3,SLC6A13,CACNA2D3,PTH2R,PDZK1,EMCN,TMEM213,ADGRF5,PPARG,TLN2,CDH16,PLPP3,ATP6V0D2,UGT2B7,SLC22A6,EPAS1,PTGER3,CLCNKB,SLC5A12,SLC3A1,UMOD,GLYAT,LRP2,PKHD1,SNX29,PDZD2,SPP1,ATP6V0A4,SLIT2,SLC17A1,LINC00871,LINC01762,KCNJ1,PPARGC1A,CA12,KL,SLC12A3,LINC01320,AGXT2,ZBTB16,KCNJ16,ESRRG,ASS1,SLC4A9,CLCN5,FXYD2,LNX1,CRYL1,CLNK,FGF13
23	ESRRG	68.33	FHOD3,CHRM3,THRB,PIK3C2G,SLC4A4,SLC8A1,SULT1C2,MECOM,MPC1,CHP1,NCKAP5,SOX5,VAV3,CADPS2,LIMCH1,PDE4D,CACNA2D3,SORCS1,LMO7,NRG1,ANK3,ATP1B1,KIAA1217,TOX3,TMEM213,DDAH1,HNF1A-AS1,MPPED2,PLPP3,TOX,PDZRN3,ASPA,PLIN5,NLGN1,EPAS1,GLIS3,EFNA5,LDHB,MUC1,PRDM16,GCNT2,SLIT2,PPARGC1A,TRPM3,CMTM4,ZBTB16,ESRRB,SORBS2,LRP1B,SDK1,RCAN2,OGDH,BMPR1B,MAP3K13,FGF13,ADGRL2
24	GATA4	68.6	FHOD3,HSP90AB1,THRB,ARHGAP8,WWC1,PRKAG2,MIA2,FMN1,TFCP2L1,SLC8A1,FTCD,EPS8,SULT1C2,MECOM,MPC1,RASSF4,CDH1,CTSH,LINC00970,TNS1,DSP,SLC13A1,CASR,HGD,GPX3,MITF,NRG1,WDR72,LMO7,PDZK1,ELOVL7,ATP1B1,SLC39A14,PRLR,KIAA1217,PPA1,SCNN1A,HNF1A-AS1,MALAT1,PPARG,PDZRN3,FREM1,TRABD2B,UGT2B7,PLIN5,PFKFB2,RBM47,ACSS2,PDE1C,EPAS1,HPN,TMPRSS2,LRP2,ATP1A1,EFNA5,UQCRH,LDHB,MUC1,ADGRG1,CUX1,EPB41L1,COBLL1,PRDM16,APOE,CKB,LINC01762,PPARGC1A,FNIP2,SLC19A2,CHKA,MYO10,ZBTB16,RAB3IP,CHCHD10,LAMB1,ESRRG,SORBS2,LARP4,DHRS3,PLEKHA7,ABTB2,SLC2A9,PAH,MAP3K13
25	RARB	69	CHRM3,CPM,THRB,BNC2,PRKAG2,SLC4A1,SLC4A4,SLC8A1,MECOM,SLC16A7,ERC1,SOX5,CADPS2,ZHX3,LIMCH1,PDE4D,CACNA2D3,SLC6A13,MITF,LMO7,NRG1,WDR72,ANK3,KIAA1217,ARHGAP10,PAX8,MPPED2,HECW1,SCNN1A,PPARG,MALAT1,TLN2,ALDOB,PLPP3,TOX,CDH16,PDZRN3,ASPA,PLIN5,FKBP5,PDE1C,EPAS1,GLIS3,TMTC2,EFNA5,RHOBTB1,LDHB,SNX29,PDZD2,CUX1,PRDM16,SPP1,GCNT2,SLIT2,PPARGC1A,PLCL1,ZBTB16,SORBS2,ESRRG,LAMB1,DHRS3,BICC1,ENOX1,NR6A1,RCAN2,TACC1,RGL3,ADGRL2
26	PGR	69.75	THRB,BNC2,WWC1,PTPRJ,AQP2,DHTKD1,MECOM,CDH1,ANPEP,EFHD1,TNS1,DSP,VAV3,CADPS2,ZHX3,GPX3,MITF,PDZK1,PRLR,BCR,SETBP1,SCNN1A,MAL,MALAT1,PPARG,PLPP3,FREM1,PDZRN3,LLGL2,FKBP5,EPAS1,PTGER3,AK4,ATP1A1,LDHB,MUC1,CUX1,EPB41L1,CLMN,AFMID,NHS,CKB,SLIT2,LINC01762,PPARGC1A,KCNJ3,CA12,CHKA,ZBTB16,PLEKHA6,SORBS2,ESRRG,ACSF2,LRP1B,RGL3,RAMP1
27	ZNF804B	71	ARHGAP8,THRB,TFCP2L1,SLC8A1,MECOM,RASSF4,NCKAP5,SOX5,CADPS2,PDE4D,CACNA2D3,WDR72,SORCS1,NRG1,ANK3,KIAA1217,PAX8,SETBP1,MPPED2,HECW1,PLPP3,PDZRN3,NLGN1,GLIS3,PCDH15,LRP2,ATP1A1,FHIT,TRHDE,PKHD1,ADGRG1,TAF1D,PDZD2,SNTG1,PRDM16,GPC5,SLIT2,TRPM3,KCNIP4,KCNJ16,SORBS2,ESRRG,OXR1,LRP1B,SDK1,RALYL,RGL3,CCSER1,SLC26A4,SCN2A,FGF13,ADGRL2
28	ONECUT1	71.25	CYFIP2,CPM,DPYS,ACSM2A,DEFB1,PIK3C2G,SLC4A4,ACSM2B,FTCD,DHTKD1,MECOM,CHP1,SOX5,DSP,CADPS2,CCDC198,HGD,SLC2A11,BHMT2,NRG1,WDR72,PDZK1,ATP1B1,SLC39A14,CLDN10,PPARG,ALDOB,PPARA,SLC27A2,ASPA,UGT2B7,EPAS1,MAOA,HPN,AFM,ITPR2,PLG,AK4,ATP1A1,CYP3A5,ACMSD,PKHD1,ABLIM2,CUX1,CLMN,COBLL1,AFMID,PRDM16,PCK1,LINC02532,KCN

Rank	TF	Score	Overlapping_Genes
			J1,FNIP2,PPARGC1A,SLC19A2,FARP1,LINC00671,ZBTB16,ESRRG,SORBS2,BHMT,TAOK3,PAH,TACC1,SLC28A1
29	MECOM	72.33	CYFIP2,GRAMD1B,CPM,RBM47,BNC2,PTGER3,TMPRSS2,PRKAG2,MIA2,ITPR2,PIK3C2G,CACNA1D,CYP3A5,FAM107B,ACAT1,SULT1C2,FGD4,MUC1,ADGRG1,MECOM,COBLL1,GCNT2,CHP1,FNIP2,RNF130,KCNJ3,TNS1,SLC19A2,VAV3,CMTM4,ESRRB,ESRRG,ASS1,SLC2A9,TOX3,BACE2,EMCN,TAOK3,HNF1A-AS1,SCNN1A,ADGRF5,PPARA,SLC26A6,LLGL2
30	ELF3	72.5	ARHGAP8,HSP90AB1,WWC1,PTPRJ,TFCP2L1,AQP2,PCAT1,CDH1,RASSF4,CHP1,DPF3,CTSH,LINC00970,DSP,PAQR5,LIMCH1,GPX3,ATP1B1,SLC22A23,KIAA1217,TOX3,PPA1,SCNN1A,ADGRF5,MALAT1,PPARG,DSCAML1,LLGL2,SAMD5,PFKFB2,RBM47,EPAS1,TMPRSS2,ATP1A1,EFNA5,RHEX,PKHD1,MUC1,ABLIM1,ADGRG1,SCIN,EPB41L1,CLMN,MYO6,SH3BP2,CKB,PPARGC1A,CHKA,CMTM4,LAMB1,PLEKHA7,DHRS3,BICC1,ASS1,FXYD2,CLNK,BMPR1B

Supplementary Table S17. Predicted transcription factor-target regulatory network.

Source	Target
HNF1B	EPAS1,ANPEP, HPN, HGD, PPARG, ATP1B1, SLC27A2, AFMID, CYP3A5, MECOM
ARID3C	ACSM2A, ACSM2B, HPN, SLC28A1, HGD, FTCD, PCK1, SLC27A2, AFM, AFMID, BHMT, BHMT2, DPYS
TFCP2L1	EPAS1, ANPEP, PPARG, PPARGC1A, ATP1B1, APOE
MLXIPL	ACSM2A, ACSM2B, EPAS1, ANPEP, HPN, SLC28A1, HGD, PPARG, PPARGC1A, ALDOB, ATP1B1, FTCD, PCK1, SLC27A2, AFM, AFMID, APOE, BHMT, BHMT2, CYP3A5, DPYS
NKX21	EPAS1,MECOM
HNF4A	OXR1, ACSM2A, ACSM2B, FHIT, EPAS1, ANPEP, HPN, SLC28A1, HGD, PPARG, PPARGC1A, ALDOB, ATP1B1, FTCD, PCK1, SLC27A2, AFM, AFMID, APOE, BHMT, BHMT2, CYP3A5, DPYS, MECOM
FOXI1	ACSM2A, ACSM2B, EPAS1, ANPEP, HPN, SLC28A1, PPARG, PPARGC1A, ALDOB, BHMT, MECOM
HNF4G	EPAS1, ANPEP, HPN, SLC28A1, HGD, PPARG, PPARGC1A, ALDOB, ATP1B1, FTCD, PCK1, SLC27A2, AFMID, APOE, CYP3A5, MECOM
MYRFL	ANPEP, SLC28A1, ALDOB, PCK1, CYP3A5
NR1H4	ACSM2A, ACSM2B, EPAS1, ANPEP, HPN, SLC28A1, HGD, PPARG, PPARGC1A, ALDOB, ATP1B1, FTCD, PCK1, SLC27A2, AFM, APOE, BHMT, BHMT2, DPYS
PROX1	ACSM2A, ACSM2B, EPAS1, HPN, SLC28A1, HGD, PPARGC1A, ATP1B1, FTCD, PCK1, SLC27A2, AFM, AFMID, BHMT, BHMT2, CYP3A5, DPYS, MECOM
NR1I3	ACSM2A, ACSM2B, EPAS1, ANPEP, HPN, SLC28A1, HGD, PPARG, PPARGC1A, ALDOB, FTCD, PCK1, SLC27A2, AFM, AFMID, APOE, BHMT, BHMT2, CYP3A5, DPYS
NR1I2	ACSM2A, ACSM2B, FHIT, EPAS1, ANPEP, HPN, SLC28A1, HGD, PPARG, PPARGC1A, ALDOB, ATP1B1, FTCD, PCK1, SLC27A2, AFM, APOE, BHMT, BHMT2, CYP3A5, DPYS, MECOM
HNF1A	ACSM2A, ACSM2B, EPAS1, ANPEP, HPN, SLC28A1, HGD, PPARG, PPARGC1A, ALDOB, ATP1B1, FTCD, PCK1, SLC27A2, AFM, AFMID, APOE, BHMT, BHMT2, CYP3A5, DPYS, MECOM
AR	ACSM2A, ACSM2B, EPAS1, ANPEP, HPN, SLC28A1, HGD, PPARG, PPARGC1A, ALDOB, ATP1B1, FTCD, AFM, AFMID, APOE, BHMT2, DPYS, MECOM

Supplementary Table S18. Podocyte subcluster characteristics and reference-root selection for pseudotime analysis.

Cluster	Nuclei	Control	DKD	Marker score	Reference root	NPHS1	NPHS2	WT1	PTPRO	PODXL
C0	453	216	237	1.0935	Yes	3.405	3.308	3.273	3.54	3.355
C1	399	173	226	0.2572	No	3.013	3.026	2.964	3.093	3.078
C2	332	198	134	−0.5862	No	2.796	2.747	2.68	2.609	2.751
C3	258	137	121	−1.3867	No	2.422	2.387	2.491	2.534	2.384
C4	132	86	46	0.6222	No	3.189	3.166	3.149	3.308	3.083

Notes: The reference root cluster (C0) was selected based on the highest composite expression score of five canonical podocyte markers: NPHS1, NPHS2, WT1, PTPRO, and PODXL. With C0 as the reference root, two transcriptional lineages were inferred: Lineage 1 (C0 → C1 → C2 → C3; 1,409 nuclei with assigned pseudotime) and Lineage 2 (C0 → C4; 579 nuclei with assigned pseudotime). Using C4 as an alternative root resulted in a single lineage (C4 → C0 → C1 → C2 → C3). Lineage-specific nucleus counts may overlap and should not be summed.

Abbreviations: DKD, diabetic kidney disease; C0–C4, podocyte subclusters; NPHS1, nephrin; NPHS2, podocin; WT1, Wilms tumor 1; PTPRO, protein tyrosine phosphatase receptor type O; PODXL, podocalyxin.

Supplementary Table S19. Trajectory sensitivity analyses and OXR1 expression patterns along inferred pseudotime.

**Leave-one-library-out trajectory sensitivity analysis**				
Excluded library	Remaining nuclei	Number of lineages	Lineage paths	Status
Control1	1,470	2	L1 + L2	Success
Control2	1,532	2	L1 + L2	Success
Control3	1,367	2	L1 + L2	Success
Control4	1,493	2	L1 + L2	Success
Control5	1,472	2	L1 + L2	Success
Control6	1,300	2	L1 + L2	Success
DN1	1,449	2	L1 + L2	Success
DN2	1,506	2	L1 + L2	Success
DN3	1,456	2	L1 + L2	Success
DN4	1,422	2	L1 + L2	Success
DN5	1,273	2	L1 + L2	Success
OXR1 expression along inferred pseudotime				
Lineage	Group	Nuclei	Spearman ρ	Mean OXR1
Lineage 1	All	1,409	−0.0829	0.7609
Lineage 1	Control	712	−0.1175	0.8689
Lineage 1	DKD	697	−0.0813	0.6505
Lineage 2	All	579	−0.0658	0.7882
Lineage 2	Control	299	−0.0576	0.9125
Lineage 2	DKD	280	−0.1164	0.6554

Note: A total of 1,574 podocyte nuclei from the GSE195460 discovery cohort were included in the trajectory analysis. Podocyte subclusters C0–C4 were analyzed using Slingshot. With C0 as the reference root, two lineages were inferred: Lineage 1 (C0 → C1 → C2 → C3) and Lineage 2 (C0 → C4). Using C4 as the alternative root yielded a single inferred lineage (C4 → C0 → C1 → C2 → C3). Leave-one-library-out sensitivity analysis excluded one of the 11 source libraries at a time and re-inferred the trajectories; two lineages were obtained in each of the 11 analyses. The association of OXR1 expression with inferred pseudotime is summarized by Spearman's ρ and mean OXR1 expression. Nuclei may be assigned to more than one lineage; therefore, lineage-specific counts must not be summed to obtain the total number of unique nuclei.

Abbreviations: DKD, diabetic kidney disease; GSE, Gene Expression Omnibus Series; DN1–DN5, DKD sample library identifiers; OXR1, oxidation resistance 1; C0–C4, podocyte subclusters; L1, Lineage 1; L2, Lineage 2; ρ, Spearman rank correlation coefficient.

Table S20. In silico OXR1 perturbation results.

Gene	Regulatory distance	Z-score	BH-FDR
OXR1	2.77×10^{-4}	7.335	0*
CERS6	1.75×10^{-5}	4.469	0*
ZDHHC6	2.06×10^{-6}	2.775	6.64×10^{-7}
SRGAP2B	1.97×10^{-6}	2.745	2.56×10^{-6}
CBLB	1.79×10^{-6}	2.678	5.12×10^{-5}
MAGI2	1.32×10^{-6}	2.471	0.044

Note: In silico OXR1 perturbation was performed using scTenifoldKnk. Among 4,057 evaluated genes, six showed significant regulatory displacement after Benjamini–Hochberg correction (FDR < 0.05). Regulatory distance represents the network-based displacement associated with computational OXR1 perturbation.

Abbreviations: DKD, diabetic kidney disease; OXR1, oxidation resistance 1; FDR, false discovery rate; BH, Benjamini–Hochberg.

Table S21. Gene sets used for mitochondrial functional module scoring in podocytes.

Mitochondrial program	Full name	No. of predefined genes	Gene symbols
OXPHOS	Oxidative phosphorylation	9	MT-CO1; MT-CO2; MT-CO3; MT-ND4; ATP6V1A; ATP6V1B2; COX6C; NDUFA4; UQCRH
TCA cycle	Tricarboxylic acid cycle	7	MDH2; FH; SUCLG1; IDH2; ACO2; CS; SDHB
FAO	Fatty acid oxidation	6	CPT1A; ACADM; HADHA; ACAT1; ECHS1; ACSL1
Mitochondrial dynamics	Mitochondrial dynamics	6	DNM1L; FIS1; MFN1; MFN2; OPA1; MIEF1

Note: Before AddModuleScore calculation, each predefined gene set was intersected with genes present in the podocyte expression matrix; therefore, the genes actually scored were the available subset of each predefined list.
